# Recovering the causal kinetic–thermodynamic relationship in asynchronous reactions

**DOI:** 10.1039/d6sc05228b

**Published:** 2026-09-22

**Authors:** Eduardo Garcia-Padilla, Guanqi Qiu

**Affiliations:** a Max-Planck-Institut für Kohlenforschung Kaiser-Wilhelm-Platz 1 45470 Mülheim an der Ruhr Germany garciapadilla@kofo.mpg.de gqiu@kofo.mpg.de

## Abstract

Kinetic–thermodynamic relationships underpin the interpretation and design of reactivity, yet strongly asynchronous reactions often display weak, erratic, or even inverted responses. Such behaviour is often interpreted as anomalous or even ‘anti-thermodynamic’, appearing to violate the expected monotonic relationship between reaction energy and barrier. We show that the standard practice of directly correlating activation barriers with overall reaction energies in asynchronous systems conflates energetically distinct phases into a single apparent step; the resulting negative or arbitrarily large gradients are artefacts of representation rather than evidence of exotic reactivity. By decoupling the reaction phases, we recover the underlying energy-barrier relationships in concerted asynchronous reactions, rendering them directly comparable to synchronous cases. We rationalise these spurious gradients by showing how differences in the energetic responses of the primary and secondary stages distort the overall energy-barrier relationship. Across representative systems, including 1,2-hydride shifts, a (3 + 2) ynolate-nitrone cycloaddition and a gold(i)-mediated 6-*endo*-dig cyclisation, we recover physically meaningful kinetic–thermodynamic relationships by isolating reaction phases through constrained internal coordinates. This framework shows that even highly asynchronous reactions obey well-defined thermodynamic relationships once their constituent processes are properly resolved. The resulting diagnostic provides a practical means to assess synchronicity experimentally and to extract the relative energetics of constituent reaction stages.

## Abstract 2

Kinetic–thermodynamic relationships guide reactivity, yet strongly asynchronous reactions often display erratic or inverted responses. Partitioning the reaction into phases recovers the interpretable energetic relationship.

## Graphical abstract

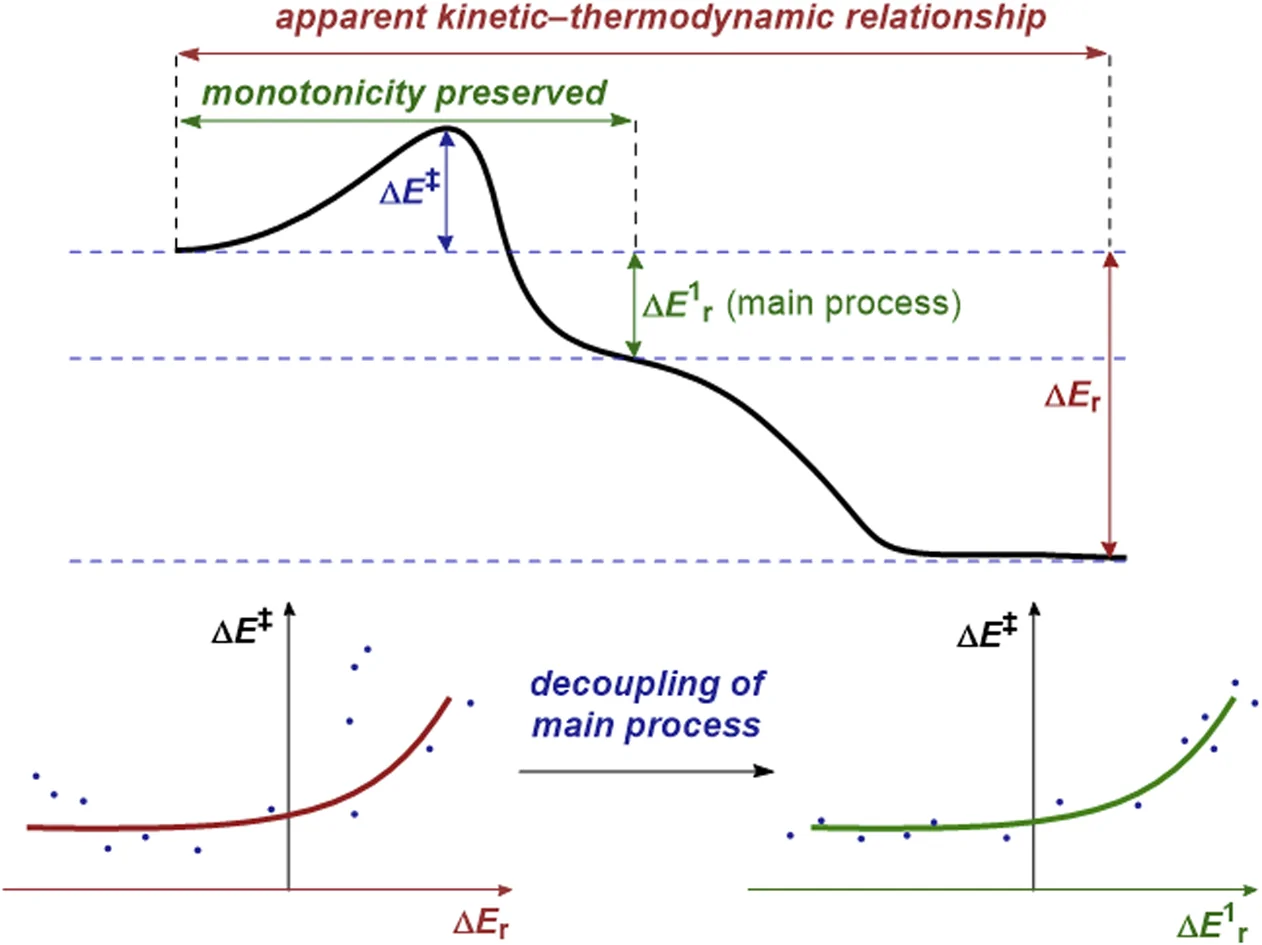

## 1 Introduction

A feature common to a wide range of organic and organometallic reactions is asynchronicity, where, in a reaction involving a single transition state (TS), two or more chemically independent processes occur in succession. In the most asynchronous cases, secondary processes may occur only after the kinetically relevant main process. Such asynchronous reactions have sparked broad interest: caldera regions,^1^ bifurcations and selectivity in some pericyclic reactions and carbocationic rearrangements.^2,3^ In synthetic chemistry, concerted asynchronous reactions are often classified alongside synchronous processes rather than stepwise mechanisms, primarily based on the absence of a trappable intermediate. Commonly, the distinction hinges on whether a step contributes to observed kinetic patterns. Stepwise reactions consist of distinct minima linked by different TSs broadly corresponding to the reaction stages, and so each with a particular kinetic signature. Concerted asynchronous reactions, while undergoing two or more chemical processes sequentially, have a single TS and no intermediate minima, with all reaction stages barrierlessly connecting to either the initial reactant or the final product of the overall chemical process. Considering the latter to be effectively identical to a concerted synchronous reaction can often be a seemingly practical simplification as, after all, there is only one kinetically relevant stage. Nevertheless, concerted asynchronous processes can drive unusually large changes in reactivity that lead to improved synthetic outcomes,^4^ explaining why many reactions exhibit lower barriers than close analogues. However, if no true intermediates are formed, how can we understand the effects of asynchronicity across very different reactions?

This question can be addressed in physical organic chemistry by examining another equally accessible dimension for concerted asynchronous reactions: the relationship between energy barriers and reaction energies. Their relationship is an important element with which to frame questions in mechanistic understanding and is generally understood to be a consequence of the interplay between molecular distortion and favourable interactions. This concept has been reflected in chemistry, most notably in the Bell–Evans–Polanyi principle and Marcus theory through the separation into thermodynamic-independent and thermodynamic-dependent contributions towards an energy barrier.^5–7^ We recently reported the derivation of the kinetic–thermodynamic relationship (KTR) from minimal assumptions,^8^ whereby the transition state arises as an energy-weighted interpolation of the minima beyond *ad hoc* fittings. Severe deviations have been documented even in early reports, with Bordwell and coworkers demonstrating that “anomalous” Brønsted slopes (*α* < 0 and *α* > 1) could be measured in the deprotonation of nitroalkanes, which was termed the nitroalkane anomaly.^9,10^ This behaviour was interpreted as violating the expected trends between the reaction energy and the barrier and was proposed by Bordwell to show limitations in interpreting *α* as a measure of the position of the TS. Other rationalisations, such as Shaik and Shurki's work with Valence Bond diagrams, showed that a simple mixing of reactant and product could be insufficient for describing a TS for reactions where structural and electronic changes do not occur synchronously, including geometrically smooth reaction pathways with multireference character.^11^

When examining the kinetic–thermodynamic relationship, how the transition state energy depends on the reaction energy, the relevant distinction becomes one of direct elementary interpolation between the minima and the transition state. Such energy relationships appear to break down in some formally concerted reactions. From a fundamentally logical, deductive standpoint, the causality of this relationship requires the core assumption of energy relationships to hold. The geometries and energy trends of the minima and the TS must interpolate smoothly and monotonically and in a similar fashion for the chosen set of reactions, as this is what enables there to be a logical link between their energies. Therefore, for KTR to operate with its conceptual grounding, Hammond's postulate must apply, and it is within these limits that we can consider the relationship to be causal within the idealised model given the interpolative assumptions. If the prerequisite does not hold, we can describe the resulting observed patterns as exclusively correlational and acausal specifically when they are analysed with this model: it would no longer track the same physical behaviour and so the results should not be interpreted as though the premise still held true. Mathematically, a correctly interpolative behaviour would require the local response gradient to be between 0 and 1,^8^ and so observed slopes outside of this range must indicate a breakdown of the core assumption. For this to happen, either the observed rate includes non-classical effects (such as quantum tunneling), or the TS is no longer geometrically or electronically connected to one or both of the minima. In the specific case of the nitroalkane anomaly, the p*K*_a_-derived reaction energy provides an equilibrium measure of deprotonation, which may combine several energetic contributions to the endpoint states whose expression at the proton-transfer transition state is not captured by a single interpolation between those endpoints, providing another possible source of apparent slopes outside this range.

While these scenarios represent established origins of apparent deviations from monotonic kinetic–thermodynamic relationships, concerted asynchronous reactions introduce another origin of apparent breakdown, arising from the conflation of energetically independent reaction phases. The reactions that preserve the validity of this framework are concerted synchronous reactions (Fig. 1a) as well as non-classical asynchronous, for which the TS remains interpolative despite spatial separation (Fig. 1c). Concerted asynchronous reactions are a notable exception, as the transition state may be geometrically unrelated to one of the minima (Fig. 1b). From this perspective, they resemble stepwise mechanisms: the TS no longer interpolates smoothly between reactant and product minima because multiple chemically independent processes are involved. As a result, direct kinetic–thermodynamic plots become purely correlational, often exhibiting spurious or noisy trends that obscure interpretable chemical meaning, with thermodynamic-independent parameters changing with the degree of asynchronicity.^12^ This affects any model that assumes a direct link between reaction energy and energy barrier conflating additional reaction stages. We address how directly correlating activation energies with overall reaction energies blends the responses of energetically independent stages into a single apparent step. Far from implying exotic reactivity, in the absence of other confounding factors (such as quantum tunnelling or non-trivial variations in entropy), it can be a practical diagnostic of a representation of an asynchronous process. To understand how the barrier responds to chemical modifications, the question thus arises: can the underlying kinetic–thermodynamic relationship of the constituent processes be extracted?

**Fig. 1:**
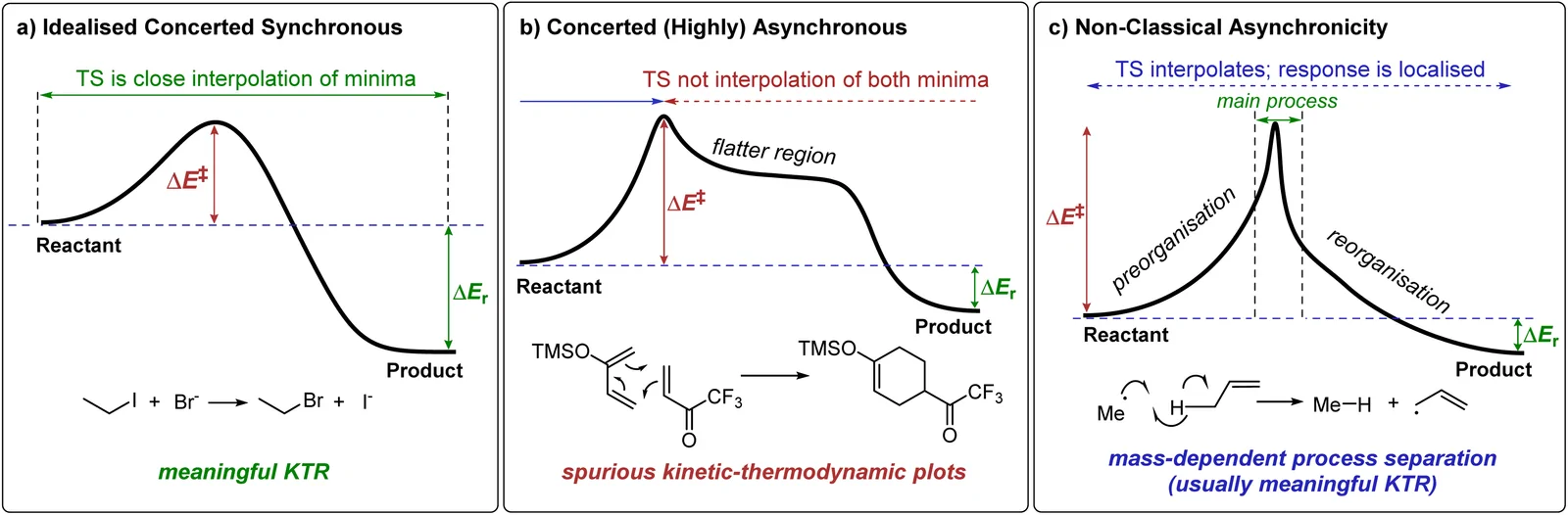
Reaction energy profiles according to their synchronicity: (a) concerted synchronous reactions, Hammond's postulate preserved; (b) concerted asynchronous reactions, defined by at least one non-stationary gradient minimum (hidden intermediate) and/or two or more curvature minima; and (c) non-classical asynchronicity, where spatial separation occurs but interpolation is largely preserved. We envisioned decoupling the individual subprocesses to determine the directly linked kinetic–thermodynamic relationship of the partial reaction, ensuring the validity of Hammond's postulate and, in this way, revealing the intrinsic causality and restoring a physical meaning to the energy analysis (Fig. 2). In this way, the actual responsiveness of the barrier and so the origin and behaviour of the kinetic–thermodynamic response at the TS can be explored for any reaction regardless of their synchronicity. Being able to study reaction phases independently also paves the way to understanding how to best modulate the energy of each stage in the reaction.

Here, we present the extraction of the kinetic–thermodynamic relationship of reactions with different types of asynchronicity, hitherto inaccessible, by studying the reaction phases separately. We show how to detect, interpret, and recover meaning from challenging cases, exemplified in three different sets of reactions and linking back to the general energy response framework. The approach is general to any concerted asynchronous chemical reactions and is not limited to the reaction classes explored in this work. Decoupling the relevant processes allows the extraction of meaningful thermodynamic-independent parameters, extending the response of a barrier to reaction energy to ubiquitous asynchronous processes. Practically, this framework provides a means to assess the synchronicity of a presumed reaction step and to resolve the relative energetics of its underlying stages.

## 2 Results and discussion

Solving the question of asynchronicity in a general manner must limit modulating the observed kinetic–thermodynamic responses with additional fitted coefficients. In addition, there may be fundamental distinctions in how different classes of asynchronicity are treated. In particular, classical asynchronicity, as defined by presenting hidden intermediates^19^ that may also include broad caldera regions,^1^ is very likely to severely distort the observed kinetic–thermodynamic response from the overall reaction energy. To approach this question, we calculated several families of reactions that displayed varying degrees of asynchronicity using density functional theory (DFT). Computational models allow a direct determination of energies and a facile characterisation of asynchronicity and its decoupling, while avoiding the contributions from extraneous factors such as quantum tunnelling, variable conformational entropy or additional asynchronous phenomena. The reported energies were calculated in ORCA 6.0 (ref. 13) using the ωB97X-D3(BJ) functional^14^ and the def2-TZVPPD basis set,^15^ with the RIJCOSX approximation^16^ with the corresponding def2/J auxiliary basis set. For the separation of reaction phases, we performed intrinsic reaction coordinate (IRC) calculations with the local quadratic approximation algorithm.^17,18^

Our initial hypothesis was that the energy response becomes less well defined with asynchronicity due to the gradual weakening of the core assumption of the kinetic–thermodynamic relationship: the factors stabilising the transition state must be defined by the minima it connects. The validity of Hammond's postulate, making the TS to respond exclusively to the energies of these connected minima, guarantees a monotonic response between the activation energy and the reaction energy. The processes with hidden intermediates in concerted asynchronous reactions are essentially independent of each other. This can compromise the causality in the total kinetic–thermodynamic response and, therefore, its monotonicity.

How can we best decouple the processes within an asynchronous reaction? Kraka and Cremer's analysis of reaction phases demonstrated that chemically independent stages of a reaction can be identified along the intrinsic reaction coordinate (IRC).^19^ In their work, the changes in curvature along the minimum energy pathway (MEP) are used as diagnostic tools to differentiate between constituent processes.^20^ These approaches have been applied in various mechanistic investigations.^21–23^ In most cases, asynchronicity can be diagnosed directly from the gradient and curvature of the MEP, as they implicitly capture the changes in the coupling of the normal modes to the IRC. In work by Jaque and co-workers, the deviation of reactions with respect to the BEP principle was found to be dependent on the degree of asynchronicity of the reaction, as defined from descriptors from the spatial derivatives of the reaction path.^24^ The application of More O'Ferrall–Jencks plots to reactions with varying degrees of asynchronicity enabled the determination of how quickly the kinetics become decoupled from the thermodynamics with increasing reaction stage separation, constituting a robust approach to rationalise deviations from linear energy relationships due to asynchronicity especially at the interphase between concerted synchronous and concerted asynchronous processes.^12^ For increasingly asynchronous reactions, the interoperability between reactants and products gradually ceases to be an acceptable approximation. Each subprocess will show separate changes in energy dependence, which are precisely the object of study in kinetic–thermodynamic relationships and suggests that a complementary approach would be valuable to decouple the energy responses of each stage in a reaction through IRC analysis. This is feasible if the reaction path is partitioned into chemically sound sections that preserve Hammond's postulate (Fig. 2). Such partitioning is not arbitrary: they have been known to relate to single chemical processes.^20,21,25^ The presence of inflection points on the MEP show the presence of so-called hidden intermediates, often showing a well-defined geometry and electronic structure. The absence of a true energy minimum should be understood as a direct consequence of the fusion with a secondary barrierless process. This, however, does not diminish the chemical identity of the non-stationary geometry. Decoupling these asynchronous processes should recover the underlying stepwise-like reactions, rather than generating arbitrary partitions, and this makes it a meaningful protocol to study a given reaction. Indeed, these inflection points on the MEP bracket a region where the reaction's geometric and energetic trends are monotonically interpolative between the effective minima and the transition state. Within this partitioned region, the causal link required for Hammond's postulate to apply is fully preserved.

**Fig. 2:**
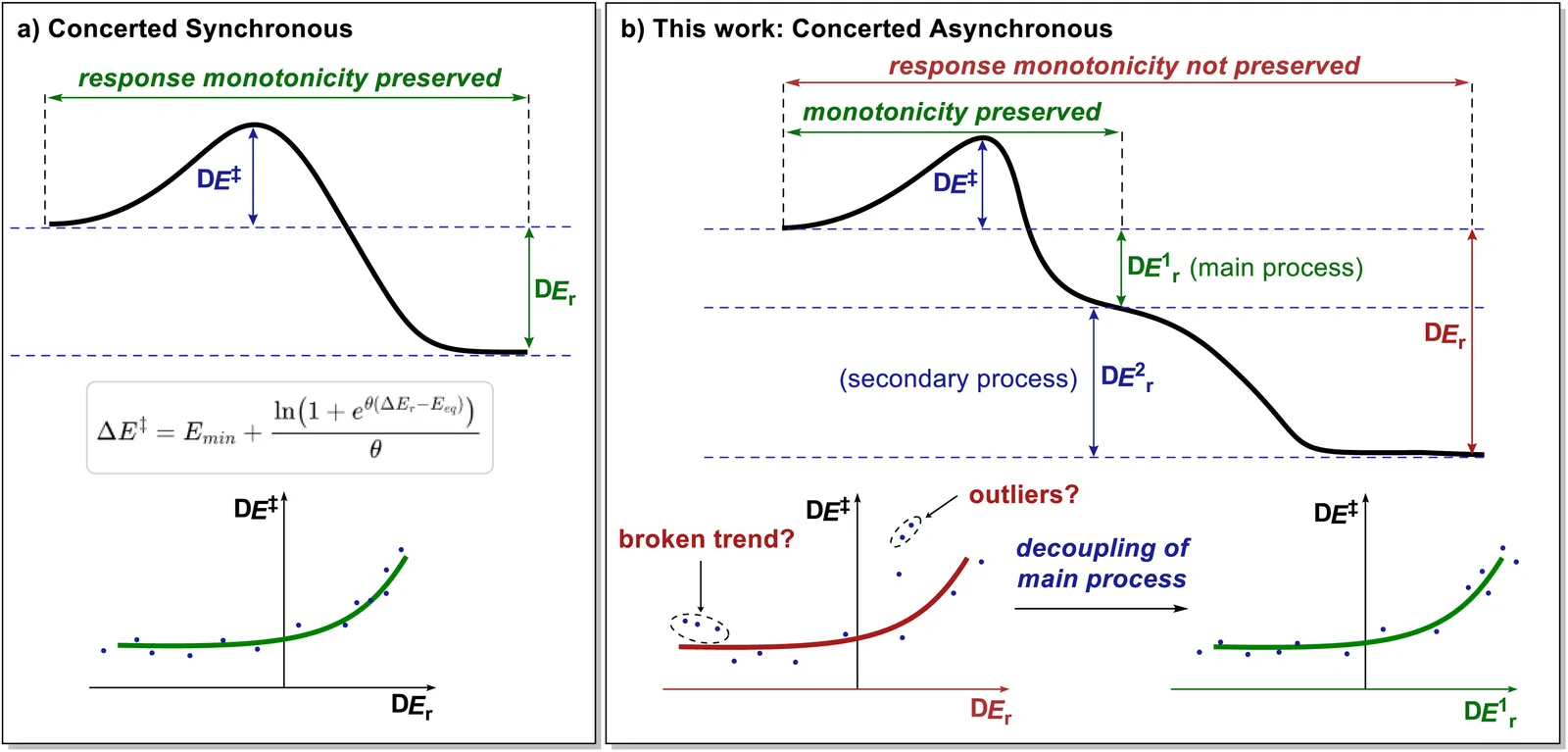
Schematic representation of how an asynchronous reaction can be partitioned to extract the kinetic–thermodynamic relationship of the constituent process. (a) Concerted synchronous reactions exhibit a direct, derivable non-linear^8^ relationship between stationary points; (b) this work: in concerted asynchronous reactions, recoverable meaningful kinetic–thermodynamic relationships obtained by isolating the chemically independent synchronous stages.

### 2.1 Asynchronicity and chemical meaning in apparent kinetic–thermodynamic responses

To explore these effects, we decided to explore a reaction family with presumed asynchronicity, 1,2-hydride shifts in protonated stilbenes, and determining what patterns appear when the core assumptions of the kinetic–thermodynamic relationship break down (Scheme 1). We used a wide range of substituted aryl rings, including several with very close electronic properties, in order to gauge the spread of the points in a kinetic–thermodynamic plot to ensure that the observed trends are not a misread cause of single coincidental outliers. The geometries were optimised using ωB97X-D3(BJ)/def2-TZVP(-f), followed by single-point energy determination with the same functional and def2-TZVPPD. We first plotted the activation energies against the reaction energies of 1,2-hydride shifts in protonated stilbenes (Fig. 3a). The plot showed a shallow curve in the range between approximately −4.7 and 4.7 kcal mol^−1^, displaying a very large negative gradient for more exothermic reactions. This observation would initially seem at odds with the expected values for the gradient of the non-linear kinetic–thermodynamic relationship: the influence of the reaction energy on the transition state has to be in the same direction (monotonic response). It therefore becomes apparent that, in this reaction, the TS cannot be an interpolation of the factors in the minima, and so would break down the Hammond postulate for the overall reaction compromising the monotonic response.

**Scheme 1:**
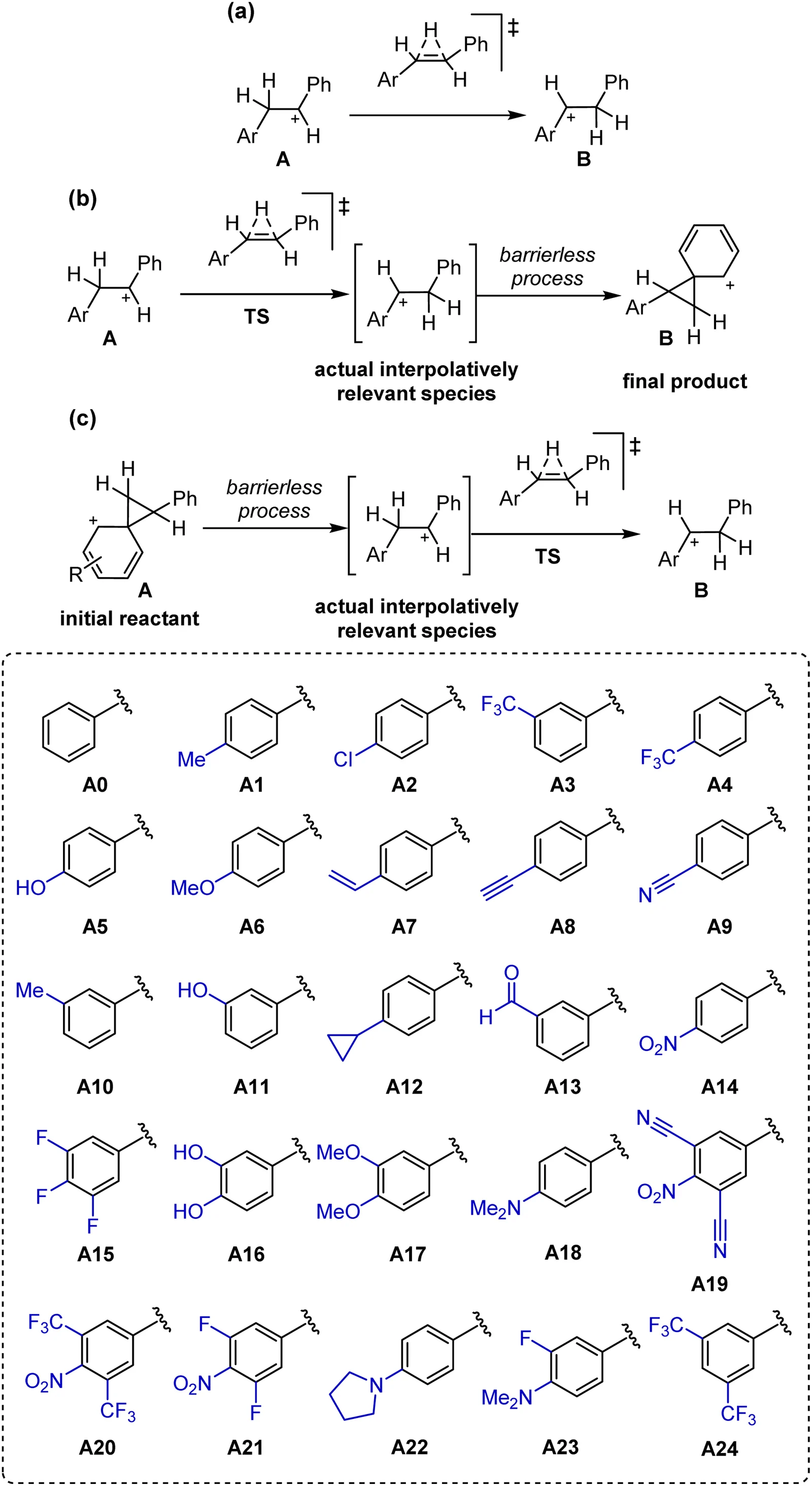
(a) General synchronous process expected, *e.g.*A0, A2; (b) aryl with strongly electron-withdrawing substituents, *e.g.*A19, A24; (c) aryl with strongly electron-donating substituents, *e.g.*A17, A22.

**Fig. 3:**
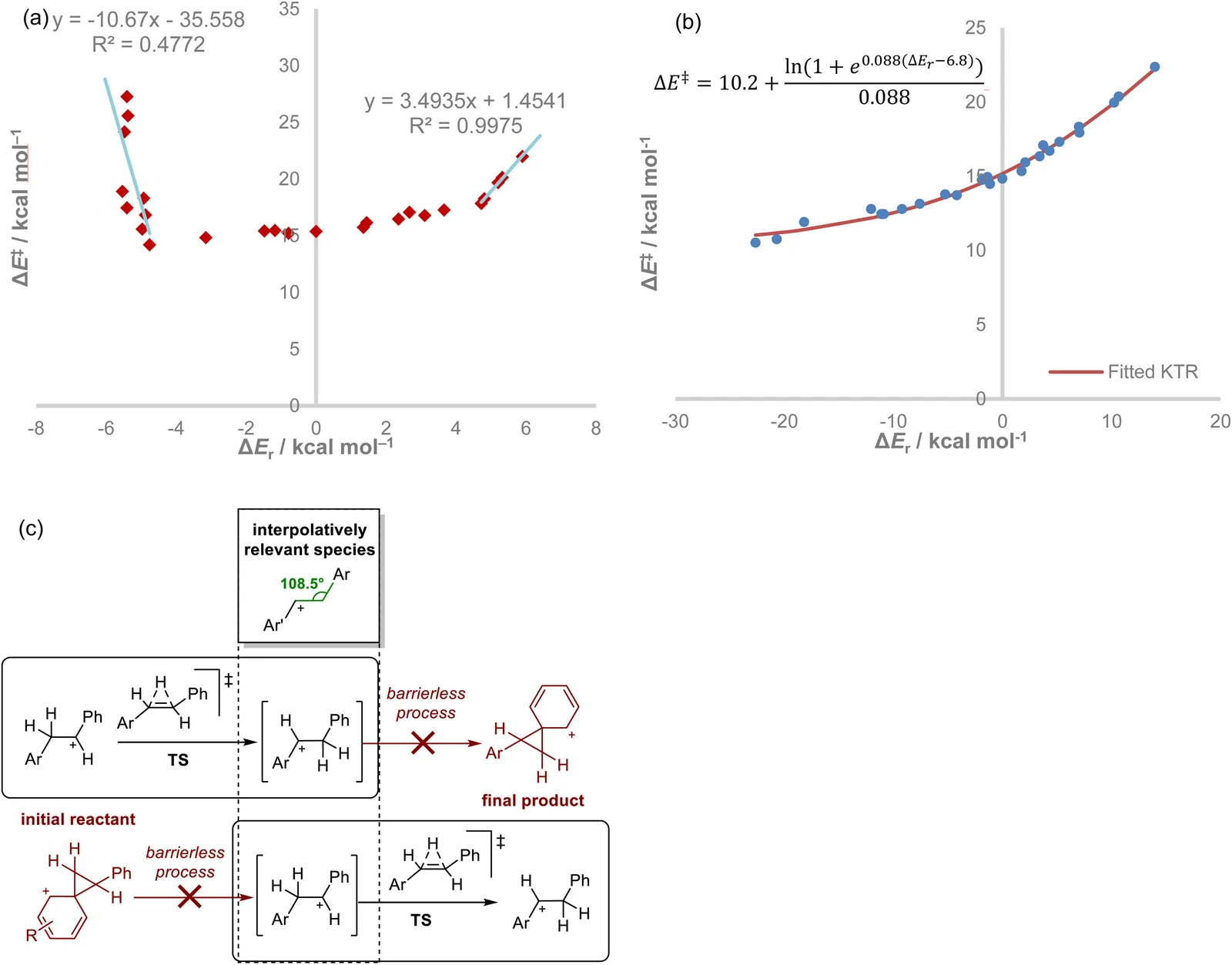
(a) Uncorrected kinetic–thermodynamic plot of the 1,2-hydride shift in protonated stilbenes, obtained by directly correlating activation energies with overall reaction energies, without separating asynchronous reaction phases. The local gradients at the extremes are inconsistent with the origin of the KTR. (b) Corrected kinetic–thermodynamic relationship for the 1,2-hydride shift on protonated stilbenes, revealing the TS interpolation. *R*^2^ = 0.991. (c) Asynchronous regimes of the 1,2-hydride shift leading to the excessively positive and negative gradients at the uncorrected driving force extremes, and the corrected reference energies by constraining the CCC angle to 108.5° to prevent barrierless spirocyclisation.

We suspected that a concerted asynchronous process could be causing this observed pattern and be responsible for the apparent “inverted region” as well as the local gradient increasing beyond 1 for the more endergonic reactions which is similarly indicative of a spurious effect in how the TS relates to the minima. Simple observation of the products showed that not all reactions connected to the same minima: while many reactions resembled the well-behaved case (Scheme 1a), when a given carbocation was particularly destabilised, a spirocyclic arenium product formed instead of the benzylic cation (Scheme 1b and 1c). To confirm whether this was a case of asynchronicity or a different reaction mechanism, we relaxed the TS through IRC analysis, revealing a concerted asynchronous process. After the 1,2-hydride shift common to all reactions, the most destabilised carbocations underwent an additional process (process 2, Scheme 1b) ultimately leading to chemically different products. In this barrierless process, the vicinal phenyl ring acts as a nucleophile stabilising the carbocation through *ipso* attack, as is the case with B14, B21 or B24.

The pitfalls in studying such highly asynchronous reaction can be understood due to the divergent nature of the two processes (1,2-hydride shift and *ipso*-electrophilic attack). As the factors that affect this barrierless reaction follow different stabilisation patterns to the 1,2-hydride shift, plotting the activation energy against the overall reaction energy is, in effect, analogous to plotting those for a stepwise reaction. This is known not to be chemically meaningful, as the observed behaviour is unrelated to the underlying energetic responses of the transition state. In fact, this can lead to arbitrary patterns depending on whether the energies of each step are more or less correlated to each other. Even if a strong correlation is seen, this would not constitute the causal relationship established in the kinetic–thermodynamic relationship.^8^ The 1,2-hydride shift of stilbenes constitutes a very clear example of how it is asynchronicity that hampers the kinetic–thermodynamic response analysis by limiting or fully eliminating the validity of the Hammond postulate.

These patterns can be understood from the variable asynchronous nature of the processes. In the more endergonic range, when the substituted aryl ring has strongly electron-withdrawing substituents, the final spirocyclic minimum is more stable than it would have been without spirocyclisation, while the activation energy is still the same (as it feels a higher energy species than the product). This compresses the kinetic–thermodynamic relationship along the *x*-axis. Conversely, for the more exergonic reactions, the substituted aryl ring has strongly electron-donating substituents, and the initial minimum is also spirocyclic as it barrierlessly stabilises the presumed benzyl cation reactant geometry (Scheme 1c). The barrier thus appears larger than it should be, as it has to overcome both the spirocycle ring-opening and the 1,2-hydride shift, with the reaction energy also being affected, leading to increasingly negative local gradients.

The IRC calculations on the transition states showed that the hydrogen migration occurred in a highly localised section of the entire process around the TS. In all cases, the non-stationary geometries where the migration started and ended were well-defined benzylic carbocations. An extended relaxation of the IRC showed that the cyclopropyl ring only formed at much larger displacements along the reaction coordinate. This is consistent with a concerted highly asynchronous mechanism. Given the extreme separation of the hydrogen migration and the cyclopropyl ring formation, this is conceptually equivalent to two separate processes, the latter of which happens to be barrierless. Through this conceptual re-evaluation, it is apparent that a concerted asynchronous process is, from the point of view of kinetic–thermodynamic correlations and reactivity theory, much closer to a stepwise sequence of processes than to a concerted synchronous process. When the processes of asynchronous reactions where the processes are of an entirely unrelated nature, this leads to the observed outliers and erratic patterns in the plot. Any direct correlation for such asynchronous reactions would have been coincidental or, at best, the result of error cancellation, which would constitute a statistical correlation instead of representing the underlying kinetic–thermodynamic response.

In that case, how can we analyse the more meaningful kinetic–thermodynamic relationship? This would require energetic decoupling of the main process, the 1,2-hydride shift contributing to the TS, and the secondary process, the subsequent barrierless spirocyclisation. Hammond's postulate should still hold perfectly for the 1,2-hydride shift process itself, if it could somehow be separated from the ulterior nucleophilic interception of the carbocation. We hindered the spirocyclisation by optimising constrained reactants and products keeping the Ar–CH_2_-cation angle fixed at a value of 108.5° (the average of well-behaved products). This treatment results in a removal of the confounding spirocyclisation so only the benzylic carbocations are reached for all reactions. In this way, the same electronic structure is preserved across all minima and the underlying energy relationship can be approximated. The resulting plot presents a typical curved kinetic–thermodynamic relationship with the local KTR gradient between 0 and 1, as expected from a truly interpolative TS, throughout and an *R*^2^ value of 0.99 (Fig. 3b and c).

This result exemplifies how the initially observed non-monotonic plot and gradients above unity were the direct and exclusive result of asynchronicity. Indeed, by preventing the barrierless secondary process, Hammond's postulate holds well and so the kinetic–thermodynamic relationship is well defined again. Being able to partition asynchronous reactions into their constituent processes is key. In principle, for all asynchronous reactions, it should always be possible to find a region in the reaction coordinate corresponding to a synchronous process where the TS interpolates between the extremes.^26^ It is worth noting that some rare chemical processes may be so heavily asynchronous near the TS that constraining an appropriate reaction coordinate is very complicated. In addition, processes with high asynchronicity both before and after the TS may be energetically dominated by the other reaction stages, the response around the TS no longer being as informative (and this is true for any chemical model reliant on interpolation). However, the model itself would theoretically hold also for these cases within a given restricted range of reaction coordinates, and so a reduction in applicability would appear rather than, or at least significantly precede, any breakdown of the model. PES bifurcations are often coupled to asynchronous processes or decomposable through dimensionality reduction,^27^ and so mostly analysable.

To evaluate how often this can be observed, we modelled the responsiveness of a (3 + 2) cycloaddition between a lithium ynolate and a nitrone (Scheme 2), which had been thoroughly explored mechanistically and found to involve a concerted asynchronous process,^21,28^ as well as developed previously as experimental methodology.^29^ In this reaction, an initial rate-determining nucleophilic attack of the ynolate onto the nitrone is followed by the ring closing through attack of the oxygen-centred anion onto the ketene. The latter process seemed to be responsible for most of the stabilisation of the reaction energy, while decoupled from the TS geometry. This would coincide with the requirements for an apparent inverted (negative-gradient) responsiveness, given our insights so far. In our modified system, we studied substituted phenylethynolates (Scheme 2) as a handle to modulate the reaction and activation energy. We calculated this reaction at ωB97X-D3(BJ)/def2-TZVPPD//ωB97X-D3(BJ)/def2-SVP+ma-def2-SVP[O] using SMD implicit solvation for tetrahydrofuran.^30^ When plotting the kinetic–thermodynamic response, the result was an observed negative slope of −0.5 (Fig. 4a).

**Scheme 2:**
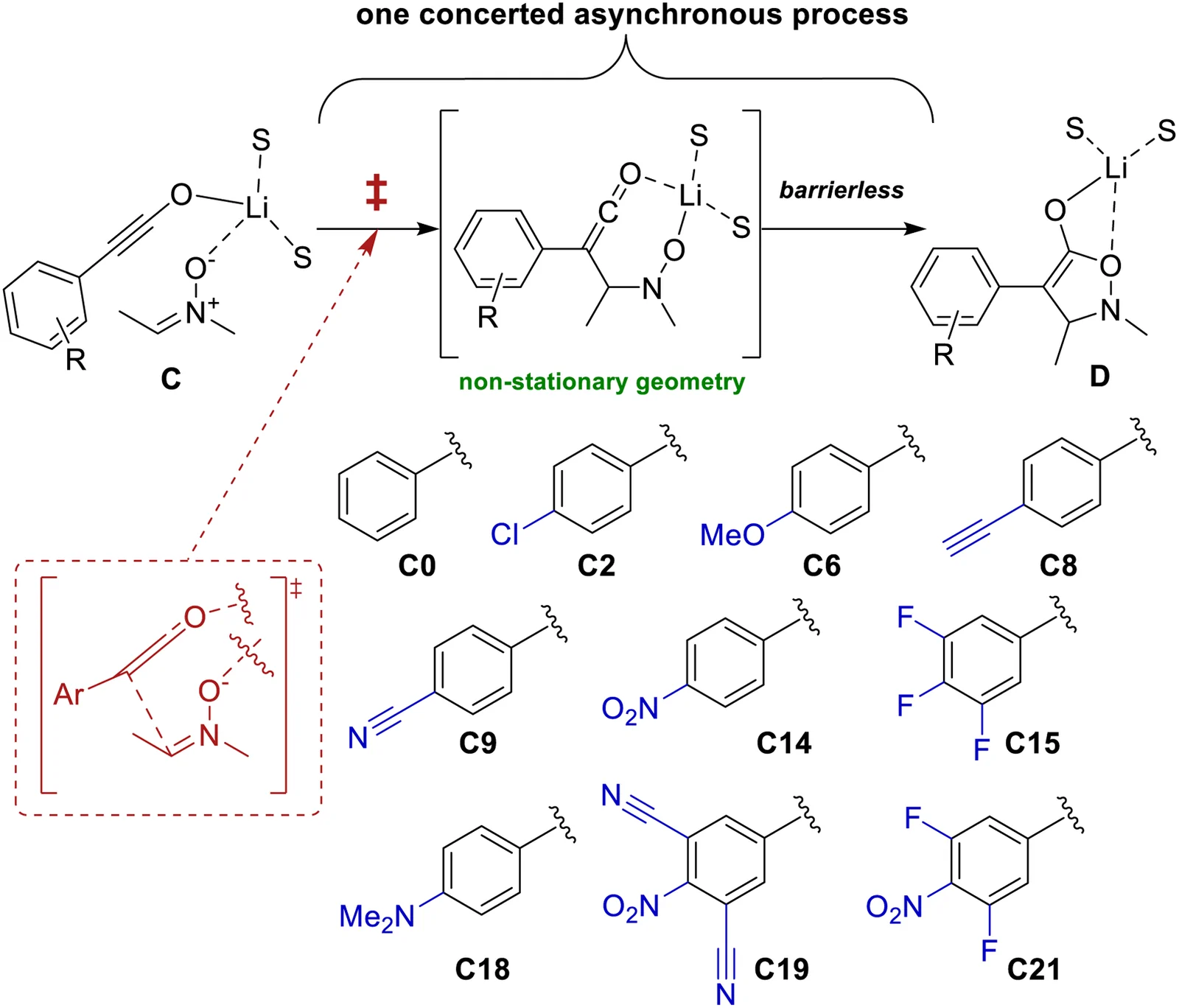
Concerted asynchronous (3 + 2) cycloaddition of an ynolate and a nitrone showing the key ketene non-stationary geometry (see Fig. 5). S = Me_2_O as model ether.

**Fig. 4:**
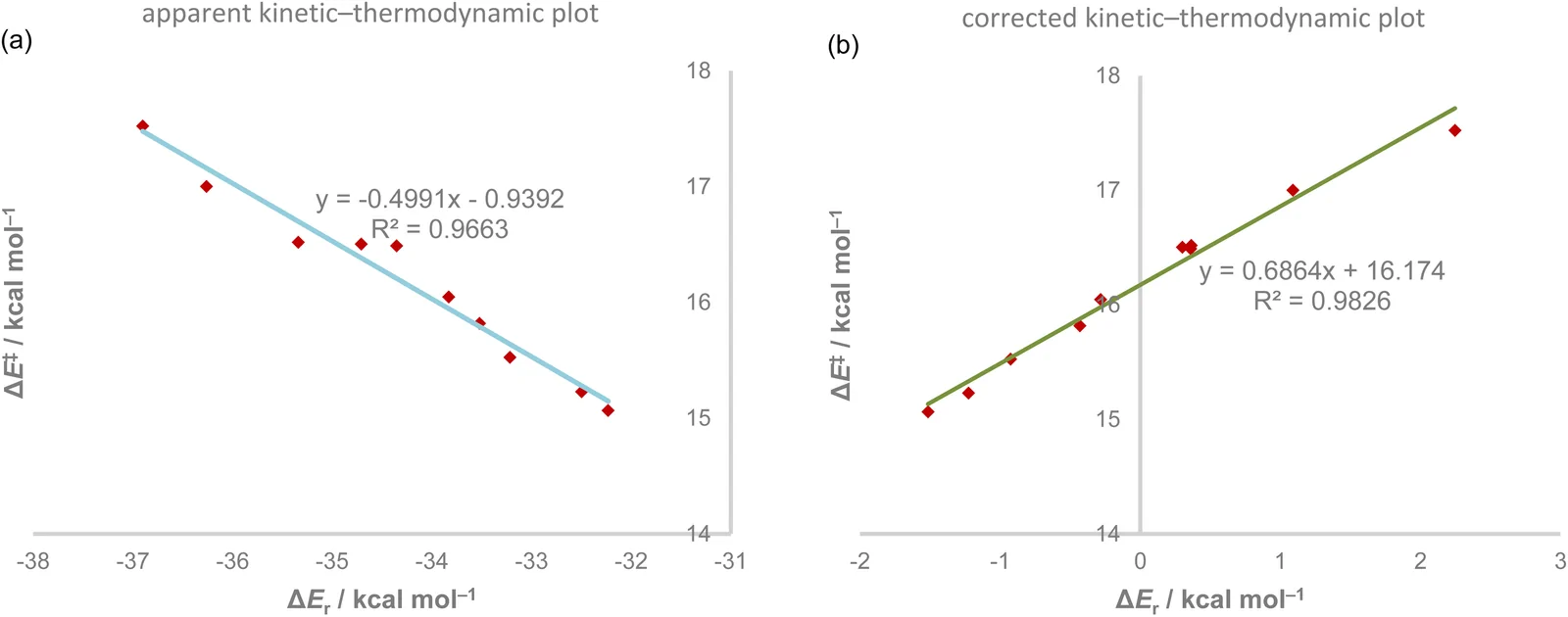
(a) Apparent kinetic–thermodynamic plot of the full (3 + 2) cycloaddition using the overall reaction energy, showing an apparently inverted response. (b) Corrected kinetic–thermodynamic plot of the initial nucleophilic attack of the lithium ynolate onto the nitrone forming a (non-stationary) ketene, recovering the monotonic relationship between barrier and energy.

In order to obtain accurate interpolatively correct geometries for the ketene non-stationary geometry, the ketene CCO angle was frozen to 180° from a geometry prior to the gradient minimum of the IRC close to the hidden intermediate followed by constrained optimisation. Using the energies of these geometries as the reference for the reaction energy, the resulting plot showed the expected positive slope (Fig. 4b). In this case, due to the shorter range of reaction energies, the curvature is not directly visible, but the same rationale can be extracted: the transition state interpolates between the reactants and the ketene hidden intermediate, and is not linked to the ultimate (3 + 2) cycloaddition product.

With a local gradient of above 0.5 in the corrected plot, this suggests that the transition state is more product-like than reactant-like throughout the observed energy range. This is captured in the kinetic–thermodynamic response by the equal-response reaction energy, *E*_eq_ < 0 kcal mol^−1^ (the reaction energy at which the influence from the reactant and product is equal).^8^ For this reason, the transition state actually responds more sharply to the reaction energy than in a symmetric case, suggesting that it is more ketene-like in the nature of its stabilisation than ynolate-like. As such, modifications that stabilise the ketene or, presumably, the formal oxoanion fragment, would be highly effective in lowering the energy barrier. This conclusion emerges only from analysis of the decoupled kinetic–thermodynamic response and would not be evident from the overall reaction, which is dominated by the second chemical process. Indeed, the large overall stabilisation is primarily a result of the cyclisation rather than of the initial nucleophilic attack, which is roughly thermoneutral for the studied reactions. Learning that the activation energy can be easily modulated with a large local gradient is practical, suggesting that including electron-rich substituents on the ketene will not only stabilise the ketene but also the transition state and lower the barrier. Note that this is at odds with any attempted interpretation of the misleading direct plotting of reaction energy and activation energy for the overall process: as the second process is barrierless, we can disregard any factors that may stabilise it and, instead, focus on the initial nucleophilic attack exclusively. The thermodynamics of that initial process are what causally lead to a higher or lower barrier and the local responsiveness will vary directly if small variations of the system are performed. The original conceptually flawed plot would not only be at odds with intuition for interpolative relationships, but, being an acausal correlation in nature, also more likely to vary in erratic ways for additional modifications on the system. The linear outcome also exemplifies how addressing asynchronicity is also critical for the reinterpretation of KTR also with classical approaches such as the Bell–Evans–Polanyi principle. As a linear approximation in the studied range of reaction energies, the premises underpinning its interpretability are the same. Classical models therefore benefit from this reaction stage decoupling much like in non-linear examples.

The intrinsic reaction coordinate analysis shows a clear separation of the two reaction phases and, importantly, the reversal in the effect that the substituents have on the energy (Fig. 5). As the ordering of the substituted reactions inverts between the transition state and the ultimate product of the two-stage reaction, this is responsible for the observed negative slope. When looking only at the first process, the ordering between the TS and the hidden intermediate is the same, which coincides with the findings that the monotonicity of the response is preserved. The apparent inverted response is an artefact from the inclusion of the two unrelated processes as a single reaction, when they effectively behave as two separate stepwise steps. The clearest examples are visible in the IRC analysis of TSCD18 and TSCD19, respectively, the most and least stable ketene species, which however form upon cyclisation the least (D18) and most (D19) stable final products of the set of reactions. In situations in which reaction energies and kinetics are experimentally obtainable but the transition state is elusive, such patterns in local gradient outside of the range between 0 and 1 would hint that the process happens through a concerted asynchronous reaction, or is otherwise stepwise and involves an additional unidentified minimum. This provides tools to interpret apparent energy responses and gain information about the synchronicity of the mechanism.

**Fig. 5:**
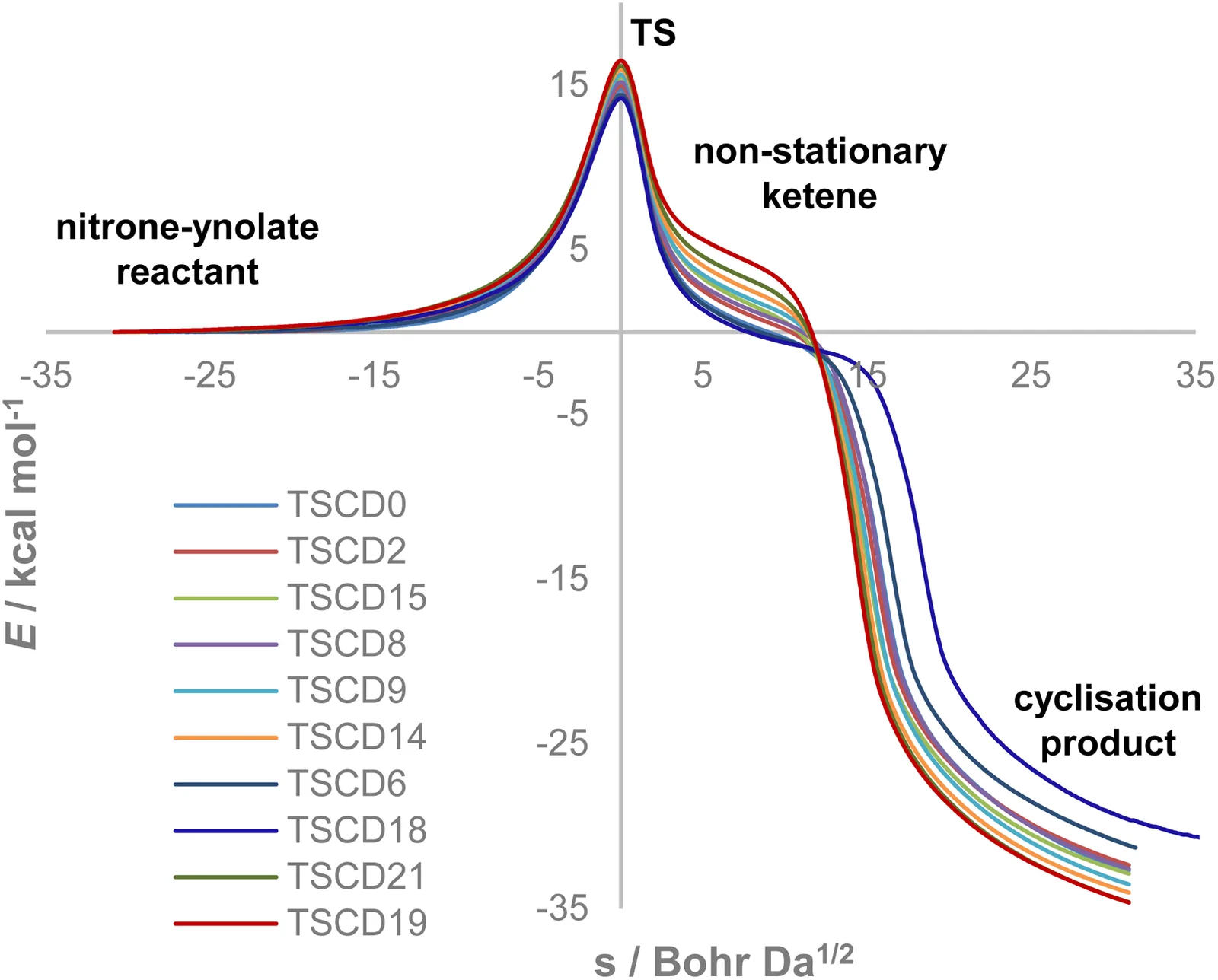
Overlaid IRCs for eight formal (3 + 2) cyclisations. Notice the crossover in the relative ordering of the reactions after the second process, causing the apparent negative gradient in Fig. 4a.

In these IRC traces for each reaction within the family, the two reaction stages remain spatially separated along the reaction coordinate. It is this sequential nature of the barrierless processes that defines classical asynchronicity and that causes a decoupling between the final product and the transition state. These independent reaction phases appear as shoulders on the minimum energy pathway, present for each of the processes in the reaction. Hidden intermediates correspond to the non-stationary geometries between the barrierless processes, and are identifiable through the presence of inflection points on the IRC.

Spurious responses arise when the secondary processes also exhibit variations in energy dependent on the same chemical modifications. Where previous approaches aimed to account for these patterns with asynchronicity descriptors, tracking the onset of asynchronicity and its effects on energy responses,^12,25^ our complementary protocol extracts the KTR of the main reaction stage itself. The degree of divergence from the expected gradient depends on the response of the secondary process: when the energy varies in the opposite direction to that of the main process, the apparent gradient will be larger than it would have otherwise been. No longer being the outcome of a causal interpolation (as interpreted with the KTR model), it may exceed the usual limits of 0 and 1. The underlying kinetic–thermodynamic response can be obtained by isolating the processes. In fact, given these insights, observations such as inverted responsiveness should be reasonably easy to anticipate: given two steps with opposite response to the same chemical modification, if the secondary step is stabilising enough, negative gradients of the apparent kinetic–thermodynamic relationship should frequently arise.

### 2.2 Kinetic–thermodynamic relationships at the transition between concerted asynchronous and stepwise processes

Some stepwise reactions, given a large enough thermodynamic driving force, can become concerted asynchronous processes. Several aspects of these systems are of significant interest and should serve to validate the methodology used so far: does the decoupling of reaction stages allow the direct comparison of the concerted asynchronous reactions with the stepwise reactions, following the same trend? In addition, the secondary step of the stepwise reactions should show its own responsiveness, barrierlessly blending with the main process only beyond a given reaction energy. Understanding this fusion of reaction stages, and acknowledging that the secondary process should still show a responsiveness of some kind (even when barrierless) shines light on how we should interpret asynchronicity more generally.

With the aim of studying one such reaction, we turned our attention to gold(i)-catalysed 6-*endo*-dig cyclisations. While a related 5-*exo*-dig cyclisation that we studied previously is highly synchronous and forms a single type of product, not requiring any particular treatment of the energies to extract the KTR,^8^ the gold(i)-mediated 6-*endo*-dig cyclisation of similar 1,5-enynes can lead to either α-cyclopropylcarbene complexes or carbocationic vinylgold(i) complexes (Scheme 3). However, when the latter are formed, there also exists a transition state that links these species to the α-cyclopropylcarbene isomers.^31^ Because of this, we suspected that in cyclisations where the cyclopropyl ring is reached directly, the reaction would likely be concerted asynchronous, proceeding through a non-stationary vinylgold(i) hidden intermediate (Fig. 6a). If this were the case, this would constitute a well-defined case study to identify when a stepwise reaction becomes concerted asynchronous, and should thus allow the analysis of the kinetic–thermodynamic relationship not only of the main process but also of the second barrierless process.

**Scheme 3:**
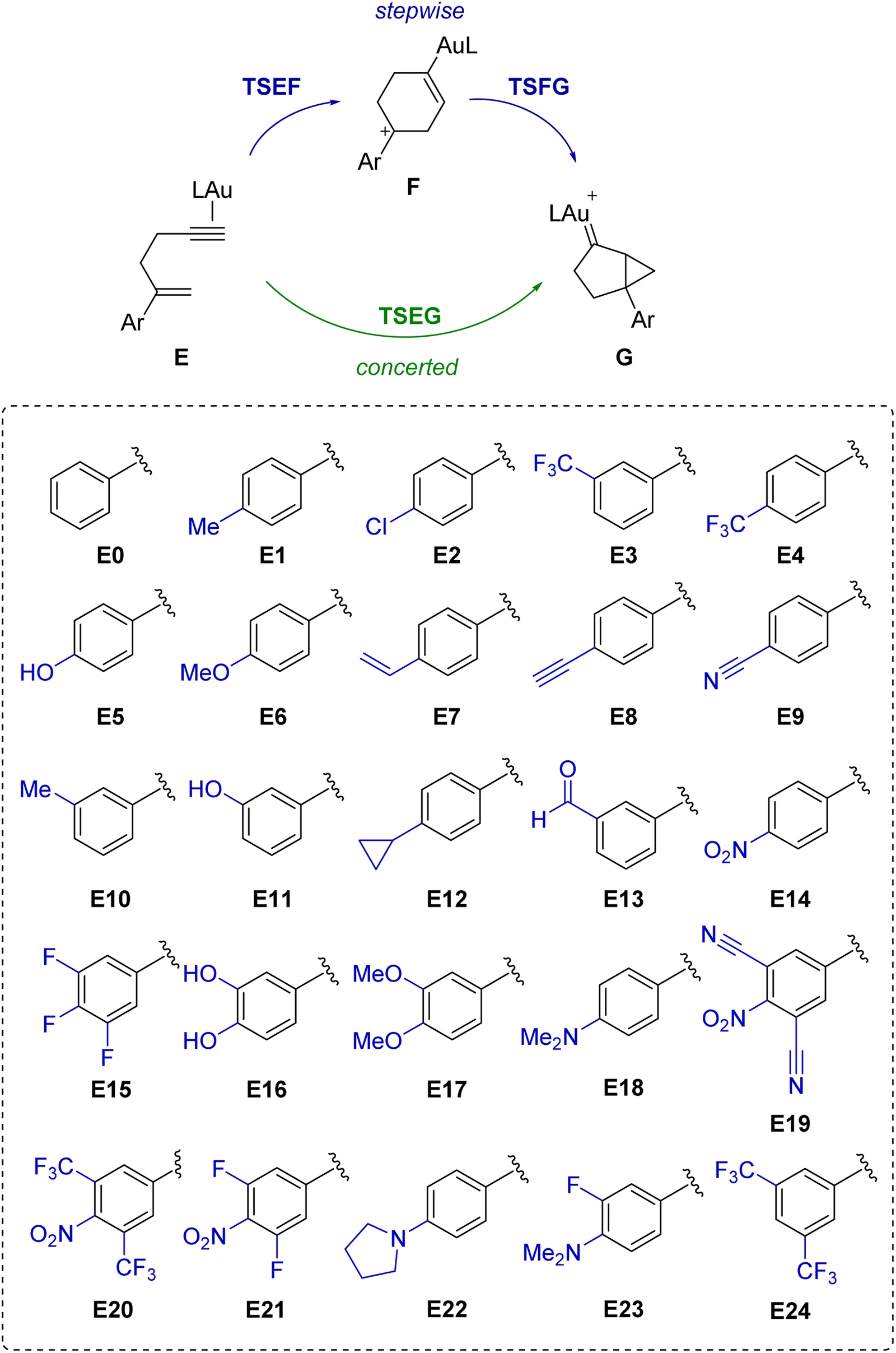
Coexistence of concerted (asynchronous) formation of α-cyclopropylcarbene complexes with stepwise pathways *via* vinylgold(i) intermediates dependent on substitution.

**Fig. 6:**
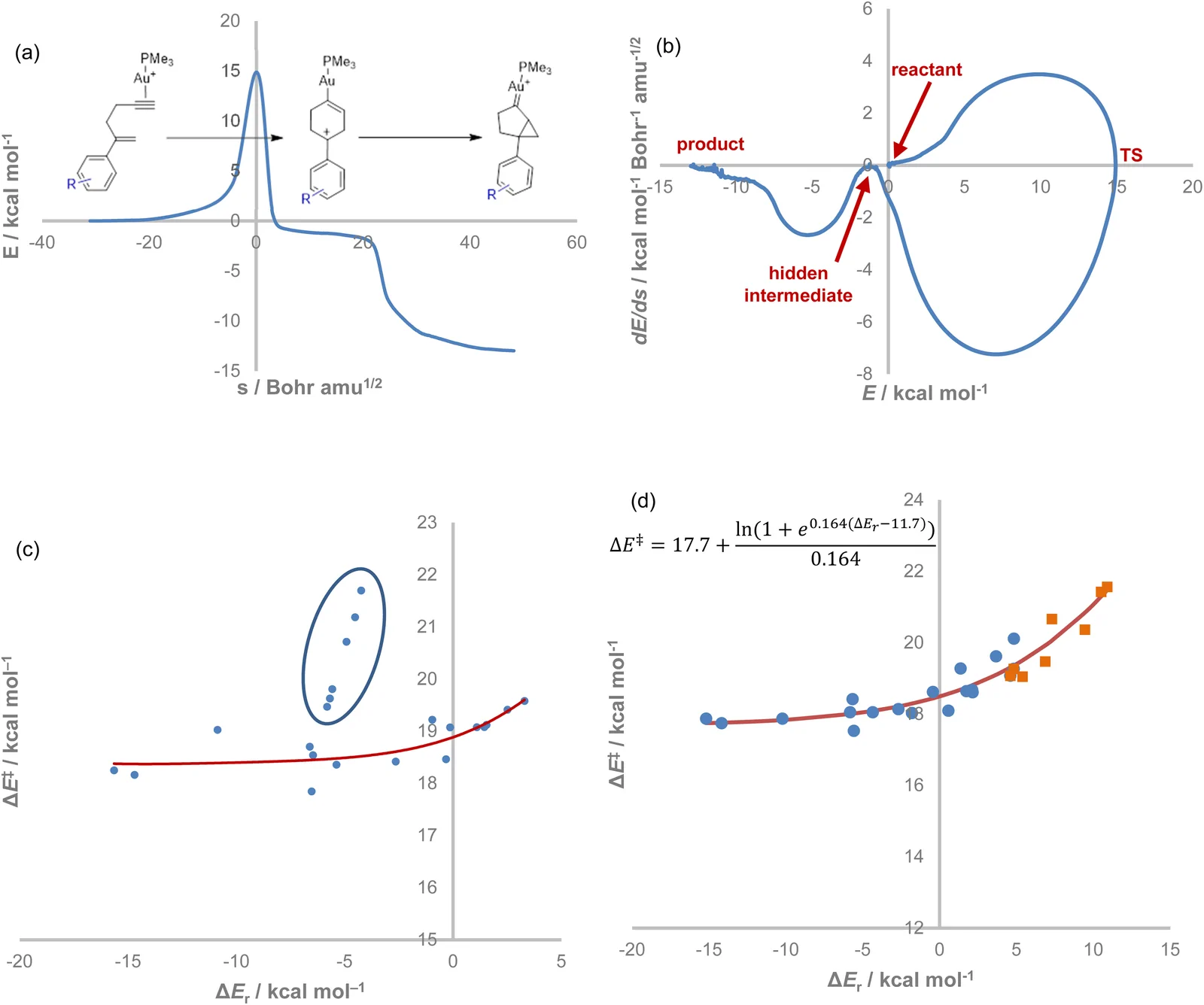
(a) IRC for the cyclisation of the 4-cyanophenyl substrate E9 (R = CN) with the representative reaction stages overlaid. (b) Mass-weighted reaction coordinate force—energy plot of the reaction from E9 to G9. The hidden intermediate of the relaxation from TSEG9 to G9 is located near −1.6 kcal mol^−1^. (c) The KTR of the 6-*endo*-dig cyclisations shows a clear spurious second trend (circled) when the cyclopropane forms directly instead of the vinylgold(i) intermediate due to the collapse of a stepwise mechanism into a concerted asynchronous process. *R*^2^ = 0.633 for the synchronous reactions. The same substitution patterns were used as in the 1,2-hydride shifts. (d) Plot of the kinetic–thermodynamic relationship of the initial 6-*endo*-dig cyclisation including both true minima (circles) and energies extracted from decoupling the asynchronous reactions (squares) *R*^2^ = 0.90.

By calculating a set of 25 reactions, again with several electronically close examples to prevent misjudging local trends as single outliers, at ωB97X-D3(BJ)/def2-TZVPPD//ωB97X-D3(BJ)/def2-TZVP(-f) with SMD implicit solvation for dichloromethane, we found that the 8 least favourable presented a concerted asynchronous mechanism leading directly to the α-cyclopropylcarbene complex, with a hidden intermediate corresponding to the vinylgold(i) species. This occurs in changing from 3-(trifluoromethyl)phenyl (TSEG3) to 3,4,5-trifluorophenyl, (TSEF15) a change of under 0.1 kcal mol^−1^ in energy barrier that constitutes the tipping point for becoming an asynchronous process. The latter, alongside the more endothermic reactions, no longer has a benzyl cation minimum, instead falling barrierlessly to the α-cyclopropylcarbene structure (Fig. 6a and b). Such patterns in a greater decoupling between reaction stages for more endothermic cases have been described and known to cause significant deviations from the expected energy relationships.^32^

Instead, if using the energy of the minima without further inspection, the apparent trend in the KTR changes abruptly (Fig. 6c). This can be found to correspond to the α-cyclopropylcarbene structures, which also exist for all other substitution patterns but are only reached after a second transition state in a stepwise process. Cases where stepwise processes collapse unto concerted asynchronous processes are known, a good example being S_N_Ar and the nature of the Meisenheimer complex geometry,^33^ but their effects on energy barriers have been only rarely explored, presumably due to the infrequent focus of studies on reaction classes as opposed to individual reactions. The relatively low correlation presented by all synchronous reactions is attributable to the proximity to the less-responsive (asymptotic) exergonic regime, where any slight non-thermodynamic differences between the reactions magnify the differences in energy barrier.^8^ The IRC analysis of all presumed asynchronous reactions showed a well-defined hidden intermediate geometry corresponding to the vinylgold(i) species, which could be observed as the shallowest gradient in the relaxation from the TS, as shown for the 4-cyanophenyl example (TSEG9) in Fig. 6b.

We firstly sought to solve the asynchronous reactions by constraining the geometries, with the aim of showing that these recovered decoupled processes should present a similar reorganisation energy to those of the truly concerted synchronous processes, and so should fit on the same plot together. Constraining the CCC angle from the benzylic cation through the three carbons that would eventually form the cyclopropane ring optimally restricts the reaction coordinate. Using a single value, ideally that of the least favoured true minimum, F3 (*meta*-CF_3_ substitution), already yields tractable data.^34^ However, closer inspection shows that the angle of the real intermediates varies significantly with substitution, from 102.1° to 111.4°, suggesting that a changing value might be better. We performed IRC calculations on the 8 transition states and, for each, noted the maximum value of the CCC angle reached immediately after the TS, in all cases found at the beginning of the region corresponding to the hidden intermediate. By using these values as references for each of the constrained optimisations, all 8 missing benzylic cation structures were obtained. The specific method to obtain the angles is explained in more detail in the SI. Nevertheless, the robustness lies not in the particular reaction coordinate or reference points (as all truly interpolative geometries should respond monotonically) but rather in the decoupling itself through the use of the IRC analysis. When the obtained energies were plotted together with those from the unconstrained synchronous processes, the points were found to follow the same trend, allowing the determination of the KTR (Fig. 6d). This shows that decoupled energies from an asynchronous reaction behave in the same way as those corresponding to minima on the PES. Alternative partitions that superficially seem more robust, such as dividing the reaction at the gradient minimum along the IRC, may not always yield data directly comparable to stepwise reactions because they would lack part of the reorganisation energy that is not reached in the concerted asynchronous process. However, for highly asynchronous reactions where the hidden intermediate undergoes practically full geometric relaxation, this can be a valid method.

We envisioned using this system to understand the kinetic–thermodynamic relationship of the secondary step, that is, the step that becomes barrierless in concerted asynchronous reactions. We studied the kinetic–thermodynamic response of the second process of all stepwise reactions (Fig. 7). The asymptote of the KTR appeared to be negative and so, in practice, unreachable, fitted in this case at around −2.5 kcal mol^−1^. After this point, the relaxation along the IRC of the first reaction blends with the second barrier by reaching a geometry close to what was the second TS, becoming concerted asynchronous.

**Fig. 7:**
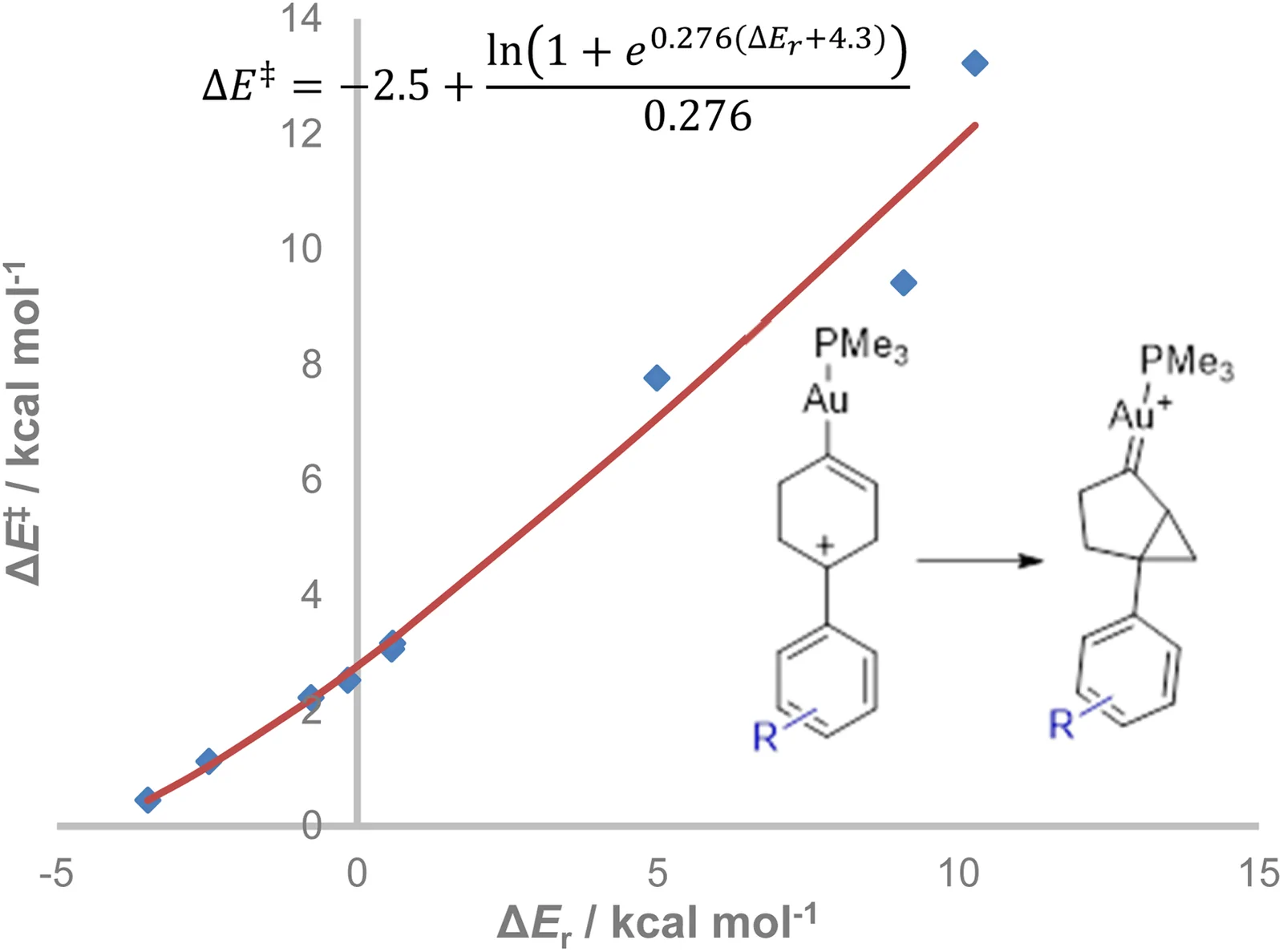
KTR plot of the formation of the cyclopropyl ring (TSH), the second step of all stepwise reactions. *R*^2^ = 0.972.

In the second reaction of the process, the cyclopropane ring formation, despite the proximity to Δ*E*^‡^ = 0, the local gradient is still above 0.6, suggesting that the ring closing eventually becomes barrierless. The negative minimum preorganisation of −2.5 kcal mol^−1^ (*E*_min_) represents the disappearance of the real minimum after a particular driving force in becoming a concerted asynchronous process. Instead of corresponding to a stable minimal torsion, in this case it captures what actually happens to the minimum: its position on the MEP and resulting energy becomes defined by the merging of the two chemical processes until there is no longer a stationary point. These examples show how harnessing both the kinetic–thermodynamic relationships can be used to probe the degree of asynchronicity in a reaction step. The difference between the real and the apparent asynchronicity, which may be experimentally derived, allows the comparison of the relative energetics of the constituent stages. The exact appearance and disappearance of asynchronicity can be visualised with a More O'Ferrall–Jencks-like diagram tracking the two newly formed carbon–carbon bonds for every point along the reaction coordinate (Fig. 8a). The benzylic carbocation appears as a stable product for the stepwise reaction and as some sort of attractor warping the MEP towards it for concerted asynchronous reactions (Fig. 8b). If such a representation is conceived as a three-dimensional representation, with the third dimension represented by reaction energy of an idealised reaction family, the switch between the concerted asynchronous and stepwise process is a cusp catastrophe (coalescence and disappearance of a minimum-TS) on the resulting surface. For reactions in which the disappearing minimum is not completely merged with the catastrophe point at any reaction energy, this could in principle enable some reactions to become concerted asynchronous even when the structure still technically exists as a minimum, not unlike the formation of oxbow lakes. The relaxation of the first process thus merges with the preorganised structure of the second one, leading to a concerted asynchronous reaction and no longer connecting to what was a minimum on the PES for other substitution patterns.

**Fig. 8:**
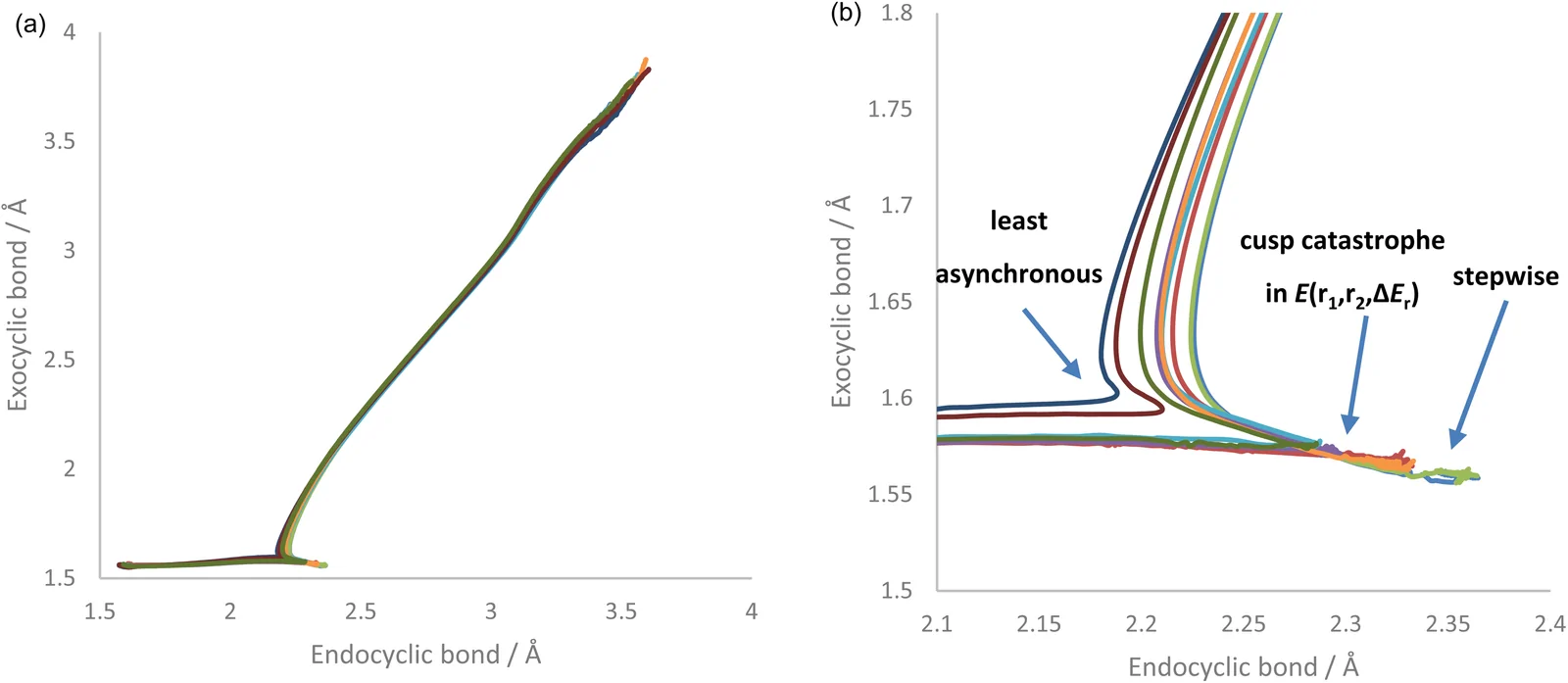
(a) Lengths between the C–C bonds corresponding to the inner (endocyclic) and outer (exocyclic) cyclopropyl bonds along the MEP for all concerted asynchronous reactions and three stepwise reactions (TSEG2, TSEG8 and TSEG15). (b) Zoomed view of the asynchronous area. The position of the catastrophe point in *E* (*r*_1_, *r*_2_, Δ*E*_r_) is not perfect due to the natural idiosyncratic differences between the substituted substrates.

Even when present, the disruption in the kinetic–thermodynamic relationship caused by asynchronicity may, however, not always be observed. In cases where secondary reaction phases happen not to change in energy with substitution, the energy of the secondary process is constant for all reactions and becomes captured as part of the thermodynamically independent pre- or reorganisation (primarily consisting of rotational and torsional relaxations before and after the main chemical process at the TS, respectively). The cases where the relative energy differences are variable, yet smaller than the main process may be more problematic: the correlation may appear smooth and show reasonable gradients while masking an underlying disruption. Solvation, strongly involved in relaxation processes and a possible direct cause of some apparent inverse kinetic–thermodynamic relationships, can modulate the degree of asynchronicity of the reactions by stabilising the hidden intermediates. The specific breakdown of the synchronicity is, thus, dependent on the relative energies of the geometries involved. In addition, many real chemical systems have extensive conformational flexibility, leading to several chemically analogous pathways, each with slightly different KTR behaviour. When combining this with the effects of Gibbs energy rather than potential energy (with which KTR behaves best, as a description of the PES) the apparent trends may be obscured even if still fundamentally well-behaved, particularly for smaller ranges of reaction energies. Often, the conformations can be treated together with little issue, but representative conformations can be used in problematic cases. In such situations, the observations can be rationalised as a weighted distribution of multiple KTR-describable barriers.

What is the situation with non-classical asynchronicity? Its nature and effects are somewhat different: it is characterised by the absence of a gradient minimum and having only one single curvature minimum, both of which result in chemically independent stages, excluding those of the ultimate minima. While these observations typically appear together, some asynchronous reactions where an interpolative relationship is disrupted or weakened can show no minimum in the gradient of the IRC but still show two or more minima in the IRC curvature. These arise in reactions with very large energy gradients or with highly spatially condensed asynchronous processes. One such example, the concerted α-halide elimination-ring opening step in a Doering–LaFlamme synthesis, is shown for reference in the SI. Primarily caused or enhanced by higher mass pre- and reorganisation and the lower mass process at the TS (such as a hydrogen transfer), the non-classical asynchronous transition state geometry typically remains interpolative between the connected minima, and Hammond's postulate is therefore preserved. Thus, there is no severe breakdown of the kinetic–thermodynamic response. Very often, the observed features in the more physically meaningful mass-weighted IRC are seen to weaken or disappear if following Cartesian coordinates instead:^31^ the separate stages are less sequential in nature than processes with large flat regions, but mass differences increase their separation. Geometric stabilising effects developed in these stages far after the TS, such as fragment reorientation, are generally unaffected by substitution and more localised as thermodynamic-independent preorganisation, impacting the TS less strongly. This has been referred to as the principle of non-perfect synchronisation, whereby earlier or later developments of stabilising factors are ascribed to the energy response of the reactions,^35,36^ matching the insights from the kinetic–thermodynamic relationship: the thermodynamic-independent pre-and reorganisation define the energy of the equal-influence point, *E*_eq_.^8^ Along with other factors, this has been linked to the development of conjugation and re-hybridisation of orbitals. This does not compromise the KTR, as no subprocesses respond to different chemical factors and there is no shoulder on the MEP. Instead, other related critical points of higher derivatives diagnose this spatial decoupling. The presence of sharp peaks in the second spatial derivative of the mass-weighted MEP (the change of reaction force constant) has been explored by Jaque and coworkers and found to correspond to this phenomenon.^37–39^ In this case, the separation of the subprocesses is attributable to mass differences in the moving fragments of the preorganisation and the TS itself. Apparent mismatches due to non-perfect synchronisation, although in reaction sets with large steric differences, have been addressed through statistical corrections.^40^ Nevertheless, these are reliant on empirical fittings or expanded parameter sets, do not account for non-linearity and no longer reflect the physical meaning of the direct kinetic–thermodynamic relationship.

The mass-related decoupling explains the effects of non-classical asynchronicity, as the TS hardly involves any other atomic movement other than the low-mass process, making the responsiveness almost exclusively dependent on the light atom movement. While the low-mass and high-mass stages become separated, this very rarely shows competing stabilisation patterns. Indeed, this is what allows the main process at the TS to respond similarly, with steric effects localised in the thermodynamic-independent preorganisation rather than introducing chemically distinct secondary processes. Conjugation primarily shifts the reactions to a significantly more exergonic regime of the same KTR where the responsiveness is consequently lower. This may explain most observations of suspected KTR changes, which often assumed a linear free energy relationship: reactions with sharper TSs change more rapidly in responsiveness and so have a more pronounced curvature, superficially resembling two different linear regimes. Other than this, the reaction sets of such processes should behave just like concerted synchronous ones, as long as the preorganisation energies can be captured as a constant value. This can be partly obscured when reactions with different steric hindrance are plotted together due to their inherent differences in preorganisation (through *E*_min_). It is, in any case, envisioned that by separating the mass-weighted preorganisation, the comparison of very diverse reactions can be enabled even where factors such as steric effects could otherwise preclude it, as well as extracting the difference in thermodynamic-independent preorganisation between the reactions. This analysis lies outside the scope of this work, as the responsiveness for each of these reactions is, in principle, valid without further refinement.

## 3 Conclusion

The kinetic–thermodynamic relationship of reactions displaying asynchronicity is hindered by the extent to which Hammond's postulate is preserved. We distinguish the impacts of classical asynchronicity, with a hidden intermediate characterised by the presence of a minimum in the energy gradient, from non-classical asynchronicity, characterised by a maximum in the gradient of the reaction force constant as well as by the absence of a hidden intermediate. The direct kinetic–thermodynamic analysis of the former without any asynchronicity treatment is severely compromised because the transition state cannot be understood as an interpolation of the minima, leading to overall trends being at best statistical in nature. Conversely, non-classical asynchronicity can still preserve the validity of Hammond's postulate, and the overall kinetic–thermodynamic responses can be analysed as long as different classes of reorganisation do not appear in the same set of reactions. We propose a systematic way of analysing all asynchronous reactions of either class by segmenting the minimum energy pathway into its chemically sequential constituent processes, extracting energies corresponding to the effective reactant and effective product. The isolated section follows the required interpolative relationship between the transition state and the minima, guaranteeing a meaningful measurable kinetic–thermodynamic relationship. Thus, our exploration enables the understanding of the way energy barriers respond to changes in local driving forces for all reactions regardless of their asynchronicity. This complements other explored mechanistic approaches in reaction force analysis and asynchronicity descriptors by showing the stage-specific response once disconnected from the reaction asynchronicity itself. Through the tracking of catastrophe points and in conjunction with reaction force analysis for asynchronicity quantification, a comprehensive picture is accessible. Concerted asynchronous reactions are not featureless from an energy response perspective, but instead a collection of complex sequential processes that respond to stabilising factors in a way that is as independent and varied as many stepwise reactions. Their behaviour is indistinguishable from that of concatenated stepwise reactions. Ultimately, our distinction and study of the energetic consequences bring asynchronicity from a peripheral curiosity to a tractable feature in physical organic chemistry. The interpolative kinetic–thermodynamic responses can be recovered even in the most elusive asynchronous reactions. Practically, this enables the modulation of asynchronous reactions directly using the underlying KTR rather than relying on the transferability of correlational patterns; furthermore, it provides insights into the secondary components of correlational patterns by comparing the apparent and the decoupled KTRs. Our approach shows that segmentation of the reaction stages provides a robust and generalisable mechanistic understanding of their chemistry: the recovered energy relationships are indistinguishable from well-behaved synchronous processes and abnormally large or negative local gradients not only show that there is asynchronicity, but also of the expected relative energy trends of the decoupled reaction phases. This allows understanding and applying the effects of thermodynamics correctly: an intuitively major bond-forming event may not be, in fact, kinetically dominant and can be detected through the trends in the kinetic–thermodynamic relationship.

## Author contributions

E. G.-P.: conceptualization, methodology. G. Q.: supervision. Both authors: writing – original draft; review & editing.

## Conflicts of interest

The authors declare no conflicts of interest.

## Supplementary Material

SC-017-D6SC05228B-s001

## Data Availability

All computed data are available within the paper and its supplementary information (SI). A collection of the computational data underlying this work has been made available in the ioChem-BD repository at https://doi.org/10.19061/iochem-bd-6-615.^41^ Supplementary information is available. See DOI: https://doi.org/10.1039/d6sc05228b.
