# Supplementary material for: Recovering the causal kinetic–thermodynamic relationship in asynchronous reactions
Source: Chem Sci. 2026 Sep 22;17(42):20714–27. doi: 10.1039/d6sc05228b (PMC13595108; doi:10.1039/d6sc05228b)
Supplement: Supplementary file 1 [file SC-017-D6SC05228B-s001.pdf]

Supplementary Information (SI) for Chemical Science.
This journal is © The Royal Society of Chemistry 2026

Supporting Information

Recovering the Causal Kinetic–Thermodynamic Relationship in Asynchronous Reactions

Eduardo Garcia-Padilla* and Guanqi Qiu*

*Eduardo Garcia-Padilla − Max-Planck-Institut für Kohlenforschung,
Kaiser-Wilhelm-Platz 1,
45470, Mülheim an der Ruhr, Germany;
orcid.org/0000-0001-9715-7992; Email: garciapadilla@kofo.mpg.de
*Guanqi Qiu − Max-Planck-Institut für Kohlenforschung,
Kaiser-Wilhelm-Platz 1,
45470, Mülheim an der Ruhr, Germany;
orcid.org/0000-0003-3818-3896; Email: gqiu@kofo.mpg.de

Table of Contents

1. Methods and Additional Calculations 3

1.1. General Methods 3

1.2. Constrained Optimisations 3

1.3. Asynchronicity without Gradient Minima and Mass-Dependent Features . 7

1.4. Reaction Energies and Activation Energies 9

2. Computed Structures and Energies 13

3. References 19

4. Coordinates of Stationary Points 20

1. Methods and Additional Calculations

1.1. General Methods

All calculations were carried out with ORCA 6.1.[1] Unless otherwise specified, calculations were run with TightSCF and, in the case of geometries, TightOpt convergence criteria. Single point calculations were performed at ωB97X-D3(BJ)/def2-TZVPPD with the tightened grid DefGrid3. The RIJCOSX approximation[2] was used and, in all cases, def2/J was used as auxiliary basis set. All stationary points were confirmed as minima or transition states through frequency analysis. Gibbs energies are reported at 298 K and 1 atm.

Geometry optimisations of the 1,2-hydride shifts on α-benzylbenzyl cations were performed with the ωB97X-D3(BJ) functional[3] and the def2-TZVP(-f) basis set.[4]

Reactions with lithium ynolates were optimised with ωB97X-D3(BJ) using ma-def2-SVP on oxygen and def2-SVP on all other atoms, a mixed basis set approach to minimise geometry error due to treatment of charge. The solvent, tetrahydrofuran, was modelled both in the optimisations and single point calculations with the implicit SMD model.[5] In some cases, where small imaginary frequencies were present, frequencies were also recalculated using a tightened grid (DefGrid3) to confirm the nature of the stationary points.

Gold(I)-mediated cyclisations were optimised with ωB97X-D3(BJ), with the def2-TZVP(-f) basis set on all non-metals and def2-TZVP (with def2-ECP) on gold.[6] The SCF convergence criteria, both in the optimisation and in the single point calculations, were increased to VeryTightSCF. The SMD model for dichloromethane was used both in the optimisations and subsequent single point calculations.

IRC calculations on the (3+2) cyclisations were carried out in ORCA 6.0 (current version as of January 2026) with the same level of theory as the aforementioned geometry optimisations using the local quadratic approximation algorithm following mass-weighted reaction coordinates.[7,8] The step size was 0.07 Bohr amu$^{1/2}$, the LQA Hessian updated with the Bofill method,[9] which is known to perform well,[10] a Bulirsch-Stoer corrector step with a threshold of 7×10^{-8} and tightened convergence criteria (tolerance of RMS gradient = 1×10^{-5}; tolerance of maximum gradient = 8×10^{-5}, strict convergence enforced). Solvation (SMD, tetrahydrofuran) was used.

IRC calculations on the gold(I)-mediated cyclisations were carried out on separately optimised TS geometries, at a ωB97X-D3(BJ)/def2-SVP + def2-TZVP[Au] level of theory with def2-TZVP(-f) on the 4 carbon atoms initially part of the alkene or alkyne, to ensure geometric proximity to the original calculation. The IRC calculation, performed in ORCA 6.1 (current version as of January 2026), used the local quadratic approximation algorithm following mass-weighted reaction coordinates. The step size was 0.19 Bohr amu$^{1/2}$, the LQA Hessian updated with the Bofill method, a Bulirsch-Stoer corrector step with a threshold of 2×10^{-6} and tightened convergence criteria (tolerance of RMS gradient = 5×10^{-6}; tolerance of maximum gradient = 7×10^{-5}, strict convergence enforced). Solvation (SMD, dichloromethane) was used.

All computational data has been included in a collection in ioChem-BD and is openly accessible at https://doi.org/10.19061/iochem-bd-6-615.[11]

1.2. Constrained Optimisations

Constrained optimisations were carried out at the same level of theory as the corresponding unconstrained geometries.

For the 1,2-hydride shifts (from protonated stilbenes), the Ph-C_{sp3}-C_{sp2} angles of well-behaved benzyl cations were found to reach around 108.5°, with some marginal variation presumably due to changes in hyperconjugation. Hence, we performed a constrained optimisation on all reactants and products of the reaction set keeping this angle fixed at 108.5°, which prevents spirocyclisation. This allows the extraction of the kinetic–thermodynamic relationship (Figure S1).

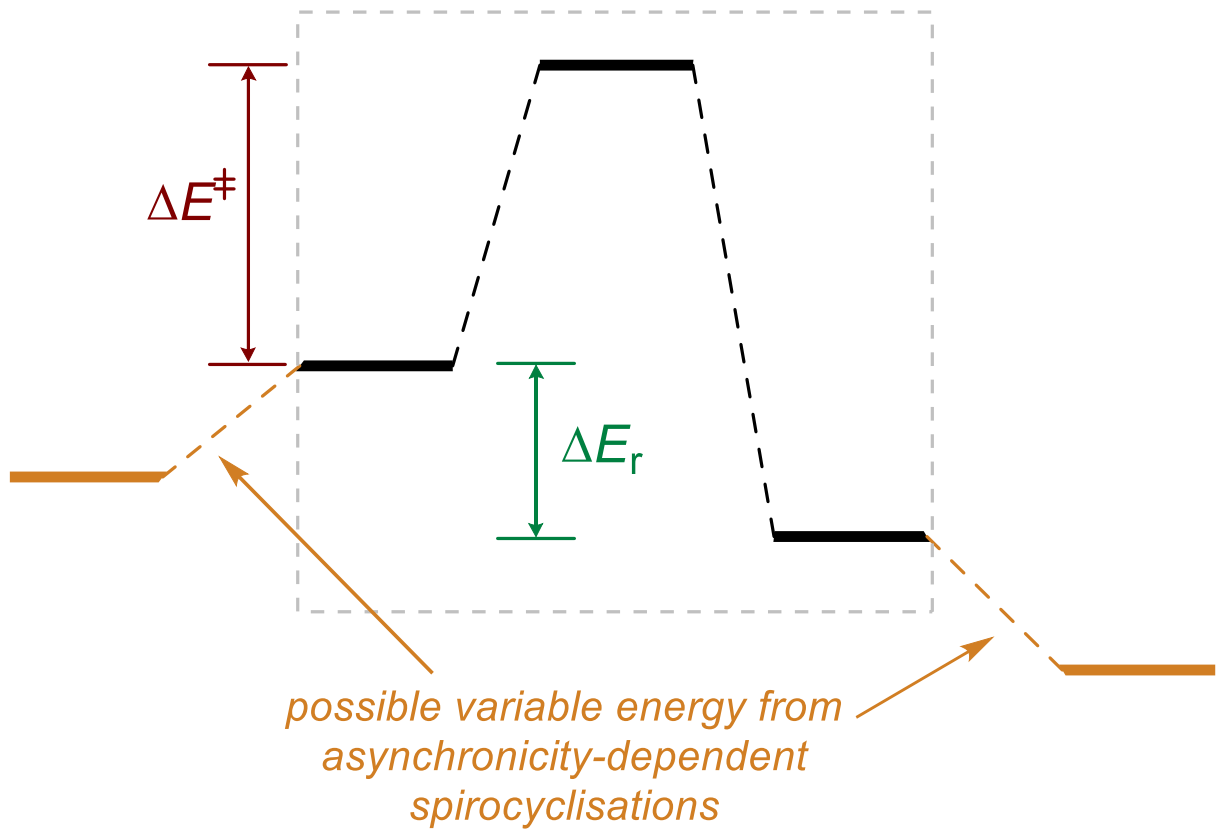

Figure S1. Conceptual representation of the main objective of decoupling: isolating the single synchronous process at the TS to study the kinetic–thermodynamic relationship; the energy changes of other processes are variable and independent from the transition state.

With the 6-*endo*-dig cyclisations, we aimed to obtain energies from the decoupled asynchronous reactions that were directly comparable to the synchronous ones (and so can be plotted together). In the manuscript, we show the better approximation for these non-stationary geometry references, which is also discussed below in more detail. The simplest way of accomplishing this is to constrain the key CCC angle (benzylic cation, CH_2, CH) to 102.1°, that of the least favoured true minimum with *meta*-CF_3 substitution. This would be analogous to the approach for the1,2-hydride shifts and indeed allows the optimisation of all 8 missing benzylic cation structures and the determination of the KTR (Figure 2). However, since the angles are seen to change significantly for all the different substitution pattern in the real minima, this is probably a worse approximation than the one used in the manuscript as it does not account for the variation in the CCC angle.

However, for all practical purposes, this alternative decoupling may have additional errors but is, however, successful in approximating the KTR by removing the secondary process (cyclopropane ring formation). The most important concept is not the evaluation of which approximation method is best, itself a worthwhile pursuit that may bear interest. Instead, it is that spurious energy responses from asynchronicity can be corrected by decoupling through intuitive constraints, and that this strategy is robust even when the constraints are not optimal.

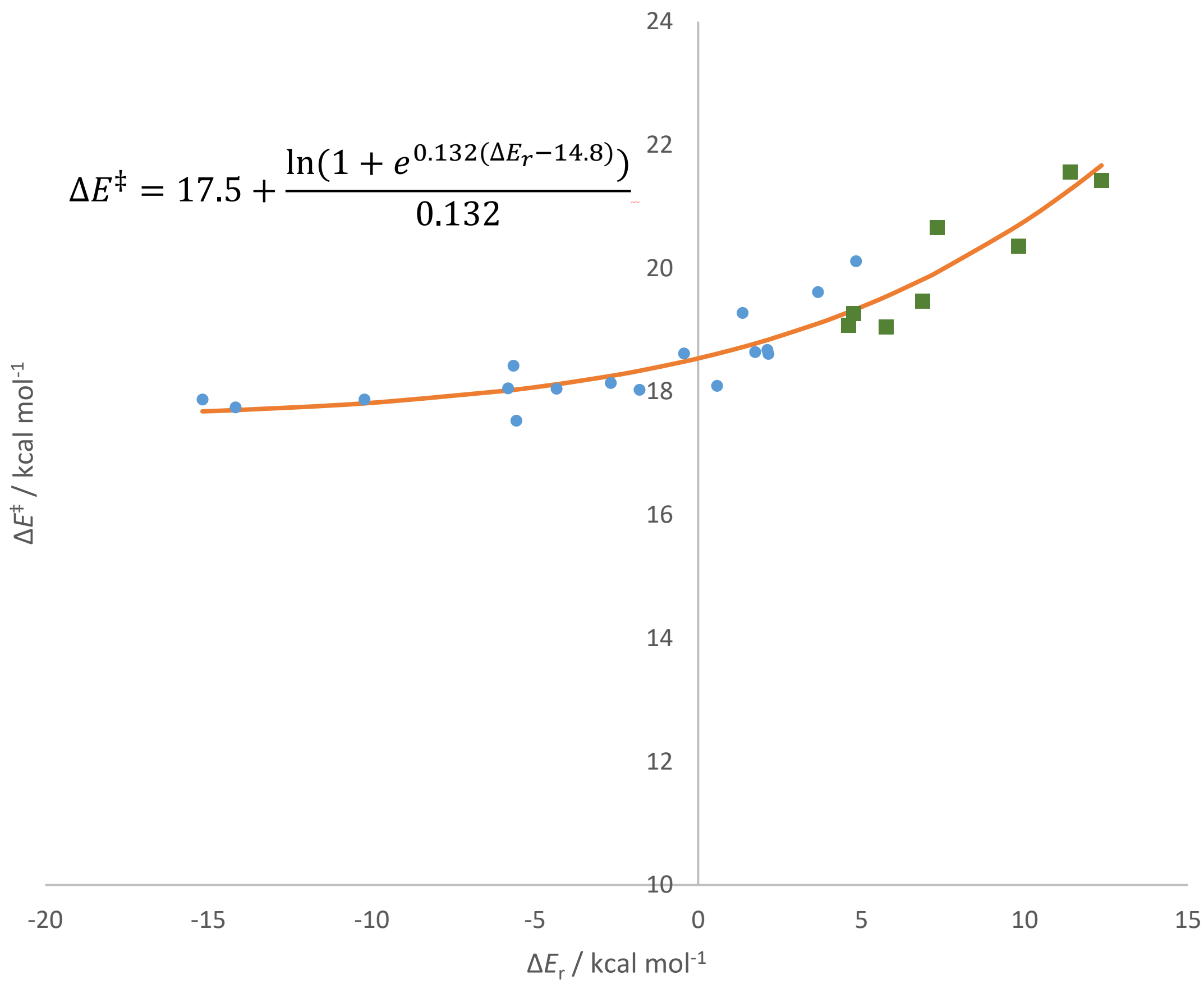

Figure S2. Plot of the kinetic-thermodynamic relationship of the initial 6-*endo*-dig cyclisation including both true minima (circles) and energies extracted from decoupling the concerted asynchronous reactions by freezing all CCC angles to 102.1° (squares). $R^2 = 0.89$.

For the IRC-based determination of the hidden intermediate (benzyl cation) angles in the eight concerted asynchronous 6-*endo*-dig cyclisations, IRC calculation were performed with the LQA algorithm at a slightly lower level of theory as detailed in the Computational Methods section. After carrying out the IRCs, plotting the key bond angle against the mass-weighted reaction coordinate showed that different reactions showed a more or less pronounced asynchronicity with large variations in the spatial extent of the hidden intermediate “plateau” (Figure S3). This is characteristic of reactions with rapidly changing degrees of asynchronicity (as is, in this case, the change between stepwise and concerted asynchronous). Note that the energies of IRC points cannot be directly plotted together with the real reactions because this would omit the minor reorganisational energy associated (from slight angle or torsional relaxations); while this is mainly thermodynamic-independent, the reaction energies would all appear higher than they should actually be.

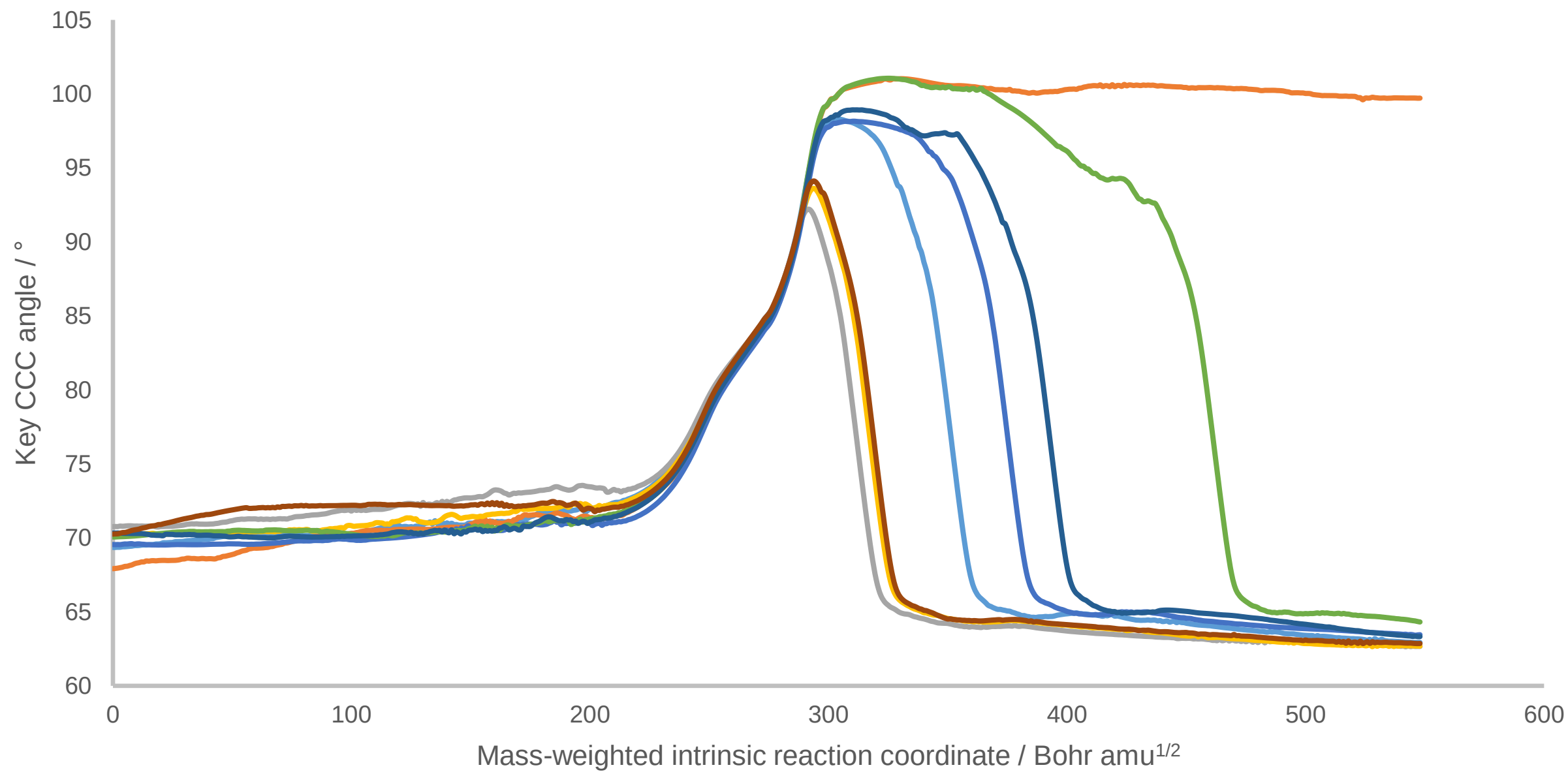

Figure S3. Change in the key bond angle over the IRC for different substitution patterns. Slowest change, orange: 3,4,5-trifluorophenyl (**TSEF15**); fastest change, grey: 3,5-dicyano-4-nitrophenyl (**TSEF19**).

The largest angles reached immediately after the transition state were selected. In order to compensate for systematic changes in the bond angles between the original triple-zeta optimisations and the IRC mixed basis set approach, we also optimised all real benzyl cation minima with the latter level of theory and compared the resulting bond angles to obtain a linear correction (HLT = 0.8602 LLT + 15.292) (Figure S4). Note that for all real minima, the discrepancies were < 1.5° in all cases.

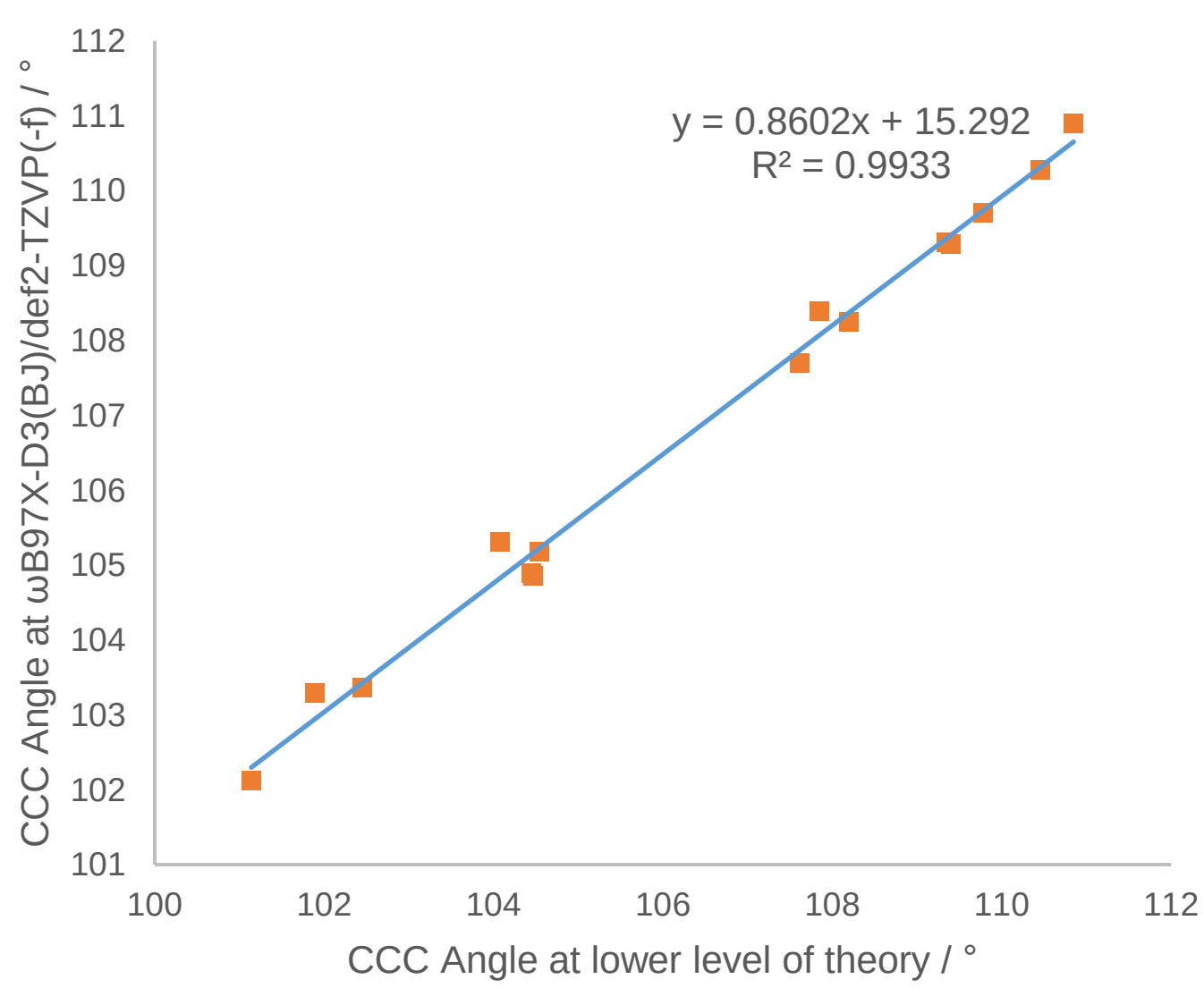

Figure S4. Linear correction used for angles extracted from the IRC.

The resulting angles used for the optimisation, obtained after the linear correction, are listed in Table S1. The angles were then constrained at the corrected value and optimised at the same level of theory as all unconstrained geometries of the 6-*endo*-dig cyclisations.

Table S1. Angles extracted from the IRC analysis and angles used for the constrained optimisations of benzyl cationic geometries.

	IRC angle / °	Corrected angle / °
**constrF4**	101.0579	102.2265
**constrF9**	98.9220	100.3891
**constrF14**	98.2957	99.8503
**constrF15**	101.0281	102.2009
**constrF19**	92.1964	94.6034
**constrF20**	94.1151	96.2540
**constrF21**	93.6110	95.8203
**constrF24**	98.1541	99.7285

For all (3+2) cycloadditions, the geometry was constrained to a linear ketene, with the CCO angle frozen at 180°. Choosing smaller angles would lead to increasingly larger reaction progression towards the final (3+2)-cyclised product. In order to evaluate the sensitivity of the observed linear energy relationship to the ketene angle, KTR plots were also obtained for 179.5°, 179.0°, 178.5° and 178.0°. The overlaid plots are shown in Figure S5.

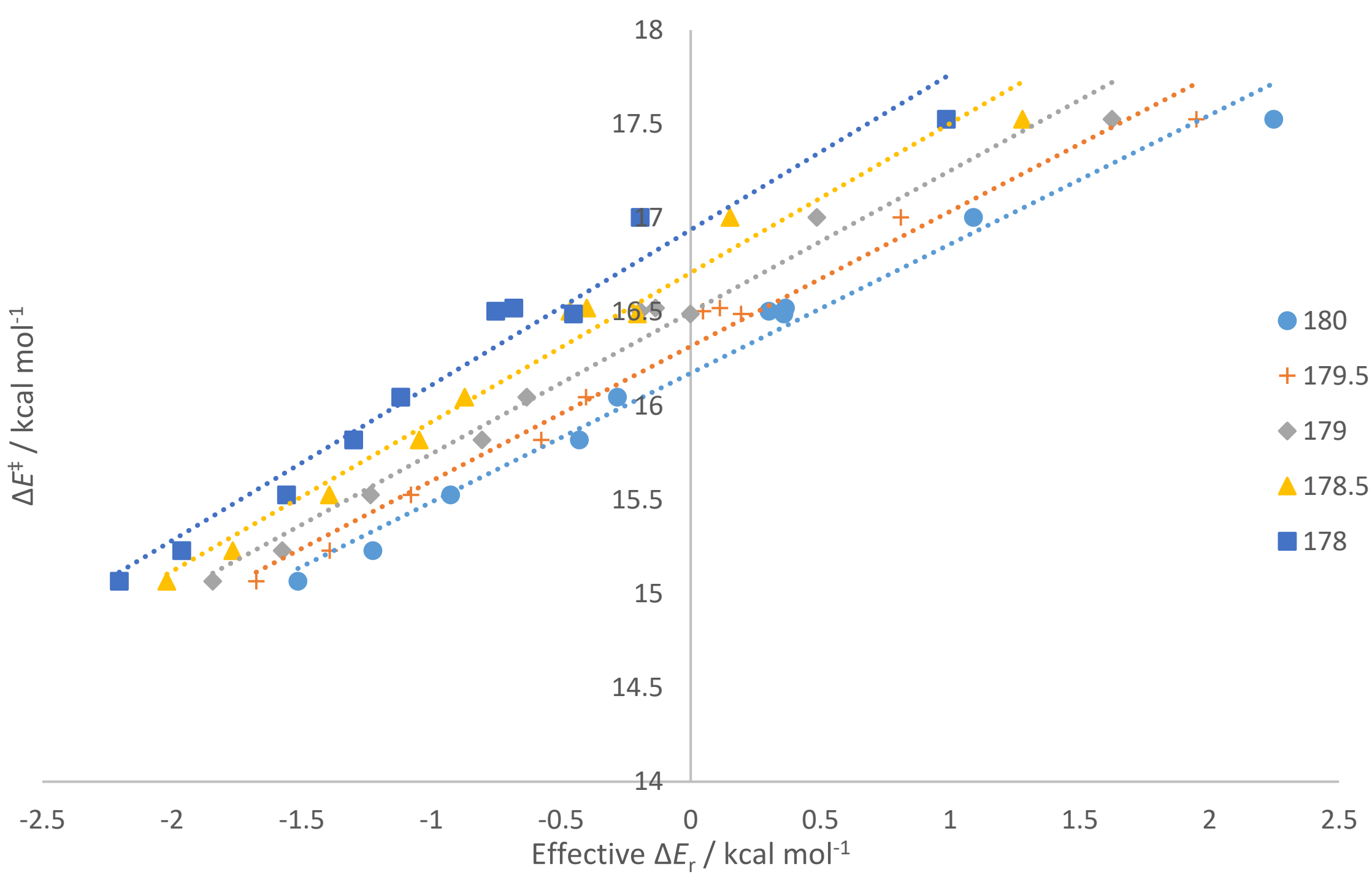

Figure S5. Linear fitting of KTR for the initial attack of the ynolate onto the nitrone, freezing the CCO angle of the ketene at one of five values.

Table S2. Variation of slopes and intercepts with ketene angle.

CCO Angle	Slope	Intercept / kcal mol^{-1}	R^2 of linear fit
180.0	0.6864	16.174	0.9826
179.5	0.7176	16.317	0.9810
179.0	0.7533	16.498	0.9795
178.5	0.7944	16.708	0.9756
178.0	0.8270	16.937	0.9650

The response of the slope is around 0.07 degree^{-1}, intercept around -0.28 kcal mol^{-1} degree^{-1} and R^2 sensitivity around 0.0028 degree^{-1}. The approximation correctly recovers the expected positive kinetic–thermodynamic response. The variation for this particular reaction is greater with geometric variation than other cases. For example, comparing with the local slopes with 6-*endo*-dig process: Figure S2: 0.323x+17.58 (R^2 = 0.86); Figure 6d: 0.383x + 17.24 (R^2 = 0.87). Both are very close in spite of the large differences in many of the used angles in the two different constrained approximations. The reason for the higher sensitivity in this (3+2) reaction is that the second process results in a rapid stabilisation upon distortion. More exothermic cyclisations (second process) lead to a decreased asynchronicity and thus to a greater change in energy with respect to geometric variation (Figure S6), which makes the KTR slope increase.

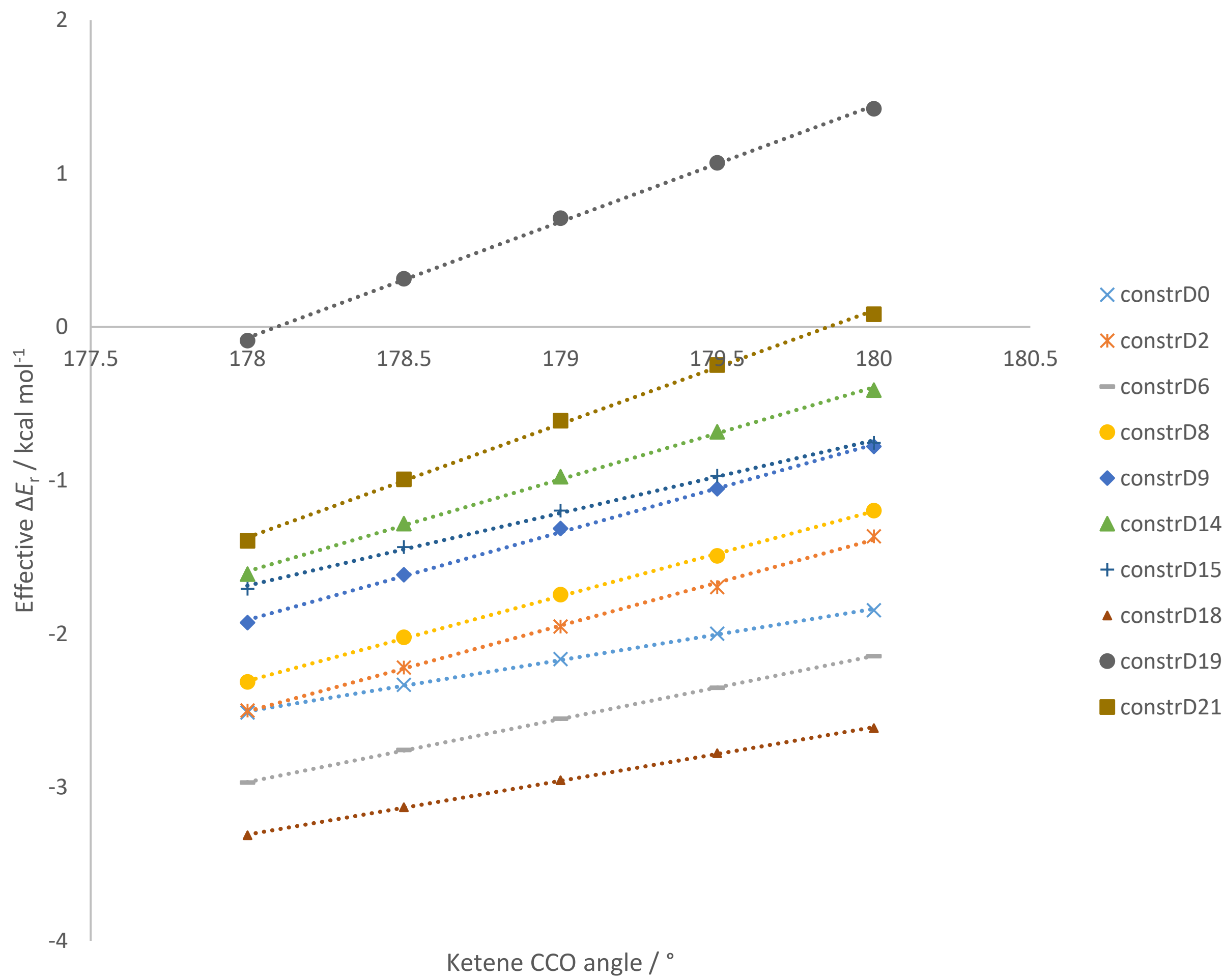

Figure S6. Variation of effective reaction energies for different frozen ketene CCO angles.

Table S3. Slopes and intercepts of effective reaction energy as a function of CCO angle as shown in Figure S6.

Pseudominimum	Slope	Intercept
**constrD0**	0.3336	-61.883
**constrD2**	0.5591	-102.030
**constrD6**	0.4110	-76.132
**constrD8**	0.5526	-100.680
**constrD9**	0.5715	-103.640
**constrD14**	0.5996	-108.320

**constrD15**	0.4734	-85.953
**constrD18**	0.3500	-65.616
**constrD19**	0.7562	-134.670
**constrD21**	0.7395	-133.000

The intercepts listed in Table S3 are closely linearly proportional to the slope (intercept = -172.69*slope – 4.863, R^2 = 0.9996). This is because of the energy-dependent degree of asynchronicity[12] and because all reactions respond in a similar way (i.e. they belong to the same reaction family). The angle-dependent equation is therefore effectively dependent on a single parameter, the slope listed in Table S3. With this slope as *s*, the energy at a given angle for a particular reaction can be linearly correlated in the following way:

$$E(\alpha) = s(\alpha - 172.69) - 4.863 \qquad \text{(Eq. S1)}$$

As the slope *s* is itself dependent on asynchronicity and the exothermicity of the second process changes the degree of asynchronicity (see Figure 5 in the manuscript), the reason for this angular dependence can be attributed to the degree to which the stabilisation cause by a Hammond-like interpolation from the final product has reached the distorted ketene at a given angle. While this is a correlational feature, it is therefore indicative of the influence of the secondary process KTR, as expected from how decreasing the angle enables the partial progression of the cyclisation. Although Equation S1 should not be linearly extrapolated because it is not analytically linear outside of this range, it would result in all reactions having an energy of -4.863 kcal mol^{-1} at 172.69°. Although the real geometric dependence is not linear, the common crossing point in Figure 5 (in the manuscript) between all IRC plots likely arises from a KT balancing phenomenon that is similar in nature.

1.3. Asynchronicity without Gradient Minima and Mass-Dependent Features

The presence of a gradient minimum is invariably linked to classical asynchronicity: there is a non-stationary region with positive curvature, a feature characteristic of minima. These cases reach a positive local maximum in curvature (d^2E/ds^2), followed by a negative local minimum, crossing the x-axis. However, there are some cases where classical asynchronous reactions do not need a gradient minimum and, yet, are separated into chemically independent reaction stages that compromise the interpolative assumption of KTR. A good example is the concerted halide abstraction—ring-opening step in the Doering-LaFlamme allene synthesis (Figure S5), calculated at TPSS-D3(BJ)/def2-SV(P), SMD (THF). There is no gradient minimum in the IRC, but clear wobbliness in the force—energy plot, corresponding to the chemically intuitive stages, shows there is some asynchronicity.

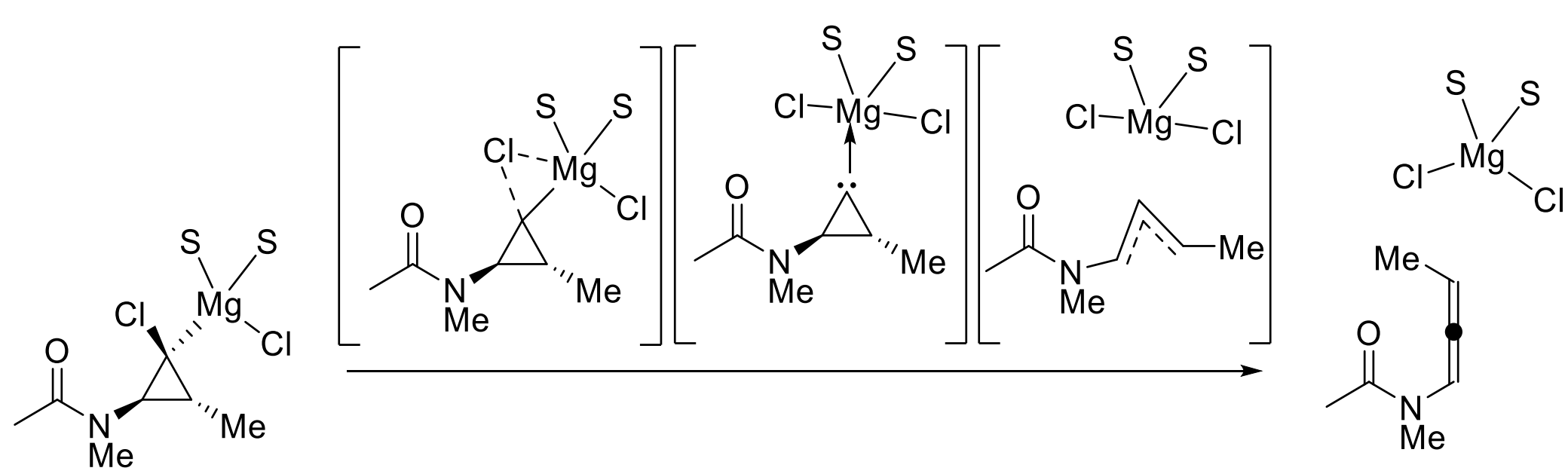

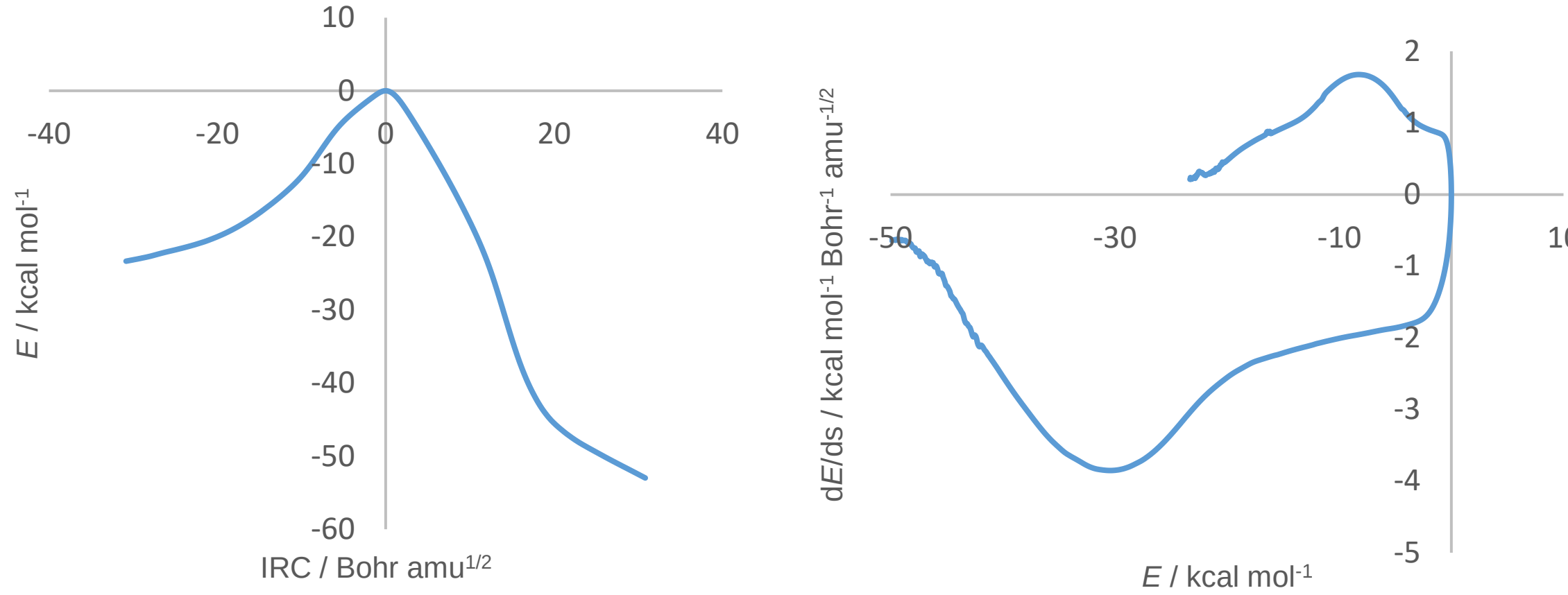

Figure S7. IRC and mass-weighted reaction coordinate force—energy plot for a Doering-LaFlamme allene synthesis ring opening step. S = THF.

When inspecting the curvature, the origin becomes clearer: two additional minima for the reaction force constant, usually attributable to a TS, appear on either side of the main TS region (the latter centred between approximately –2 and 3 Bohr amu$^{1/2}$) (see Figure 6). This is indicative of more reaction stages, which, in this particular reaction, are chemically independent and likely show different responsiveness patterns to substitution. If the curvature had increased slightly more, enough to cross the x-axis, a gradient minimum would have formed.

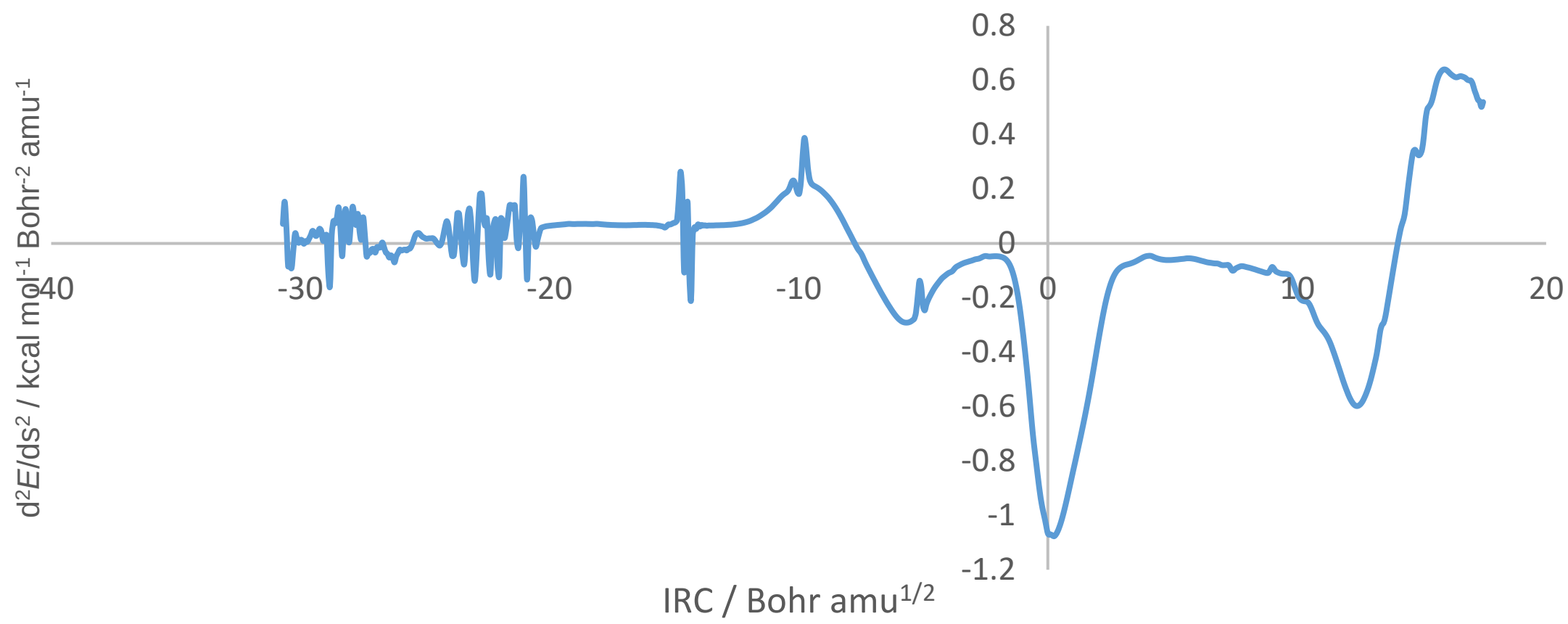

Figure S8. Evolution of reaction force constant (d^2E/ds^2) along a section of the IRC, showing additional curvature minima near –4.9 Bohr amu$^{1/2}$ and 12.4 Bohr amu$^{1/2}$.

Conversely, for reactions with strictly no gradient minima nor more than one curvature minimum, mass can become a defining feature of non-classical asynchronous processes, where the degree of asynchronicity can only be seen in an unusually rapid change in the curvature, indicating a change in the type of chemical relaxation. Most commonly, this happens before reaching a minimum, although its extent can be limited in strained systems. As detailed in the manuscript, it consists of processes minimally affected by the substitution pattern (hindered rotations, torsions, partial reorientations…) and so mostly captured in thermodynamic-independent components E_{min} and E_{eq}. In the very rare case that the reorganisation energy significantly depended on the given substitutions without breaking the validity of the reaction family, it could weaken Hammond's postulate enough to worsen the KTR correlation or could even hypothetically warrant asynchronicity analysis; however, in our experience, we have not observed this pattern in any studied systems.

The mass-related separation of the main process and the reorganisation is best visualised by plotting the IRC spatial derivative of energy against the energy. When Cartesian coordinates are used to track the relaxation of the TS, which is practical for this matter but an otherwise discouraged choice, the features practically disappear, suggesting that mass differences are key, as can be seen for an HAT reaction on acetonitrile (Figure S7). This has consequences on the kinetic–thermodynamic response, as it causes the TS to respond much less strongly to any structures that require rearrangement of polyatomic fragments, quickly reaching its “earliest” or “latest” TS character (generally high θ). Notice the features on the mass-weighted reaction coordinate plot near (–7.1, –8.8) and, slightly less visible, near (–1.1, 3.7), whereas no well-defined features can be observed for the IRC following Cartesian coordinates.

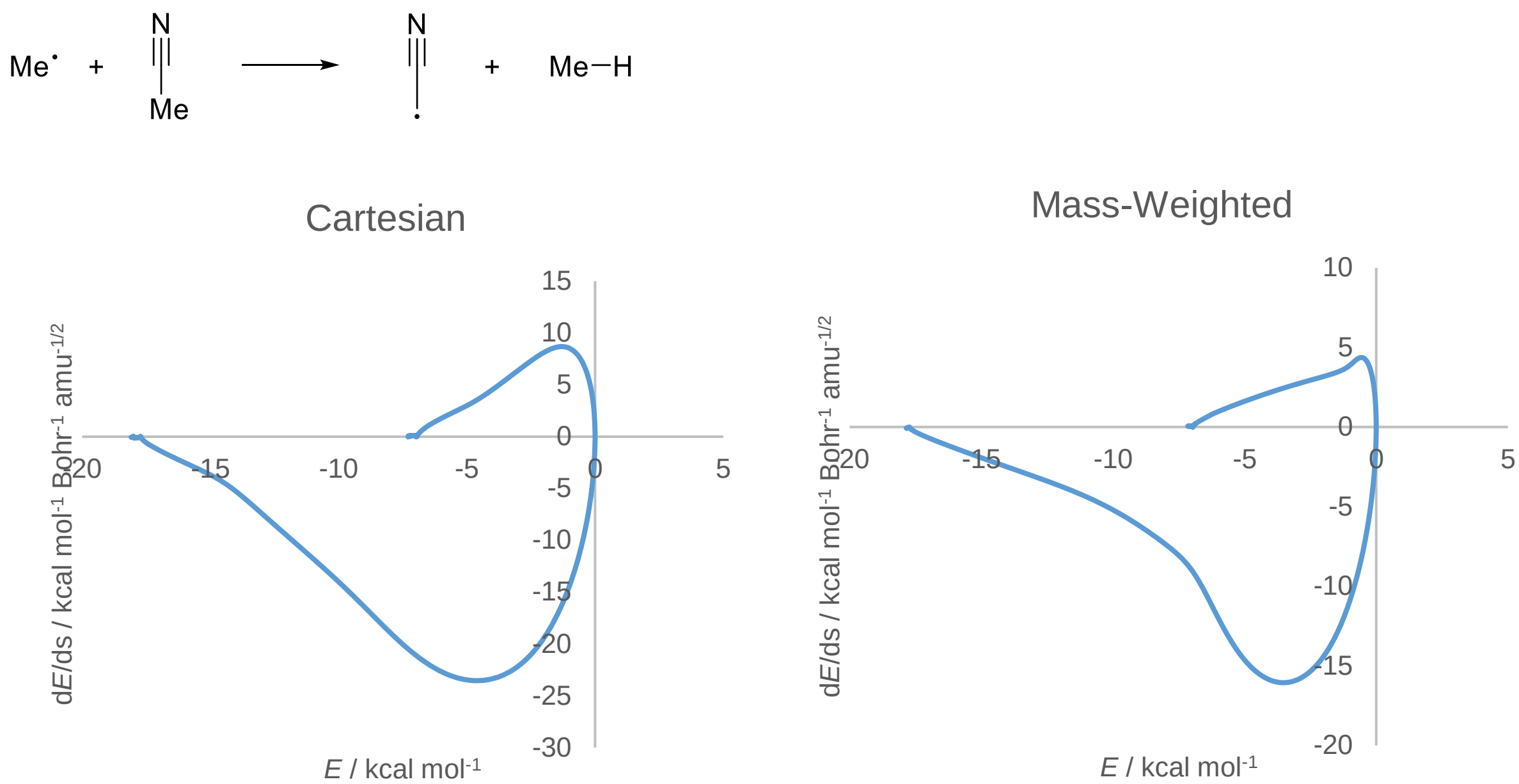

Figure S9. Comparison of the IRC reaction coordinate force—energy plot when following Cartesian coordinates and mass-weighted coordinates for an HAT reaction of a methyl radical with MeCN.

1.4. Reaction Energies and Activation Energies

The energies of all reactions shown in the figures of the manuscript, as well as the Gibbs energies where relevant, are tabulated here.

Table S4. Reaction energies and activation energies for the 1,2-hydride shifts on protonated stilbenes.

Reactant	ΔE_r / kcal mol^{-1}	$\Delta E^‡$ / kcal mol^{-1}	ΔG_r / kcal mol^{-1}	$\Delta G^‡$ / kcal mol^{-1}
**A0**	0.0	15.4	0.0	13.0
**A1**	-3.1	14.8	-3.3	12.3
**A2**	-1.2	15.5	-1.2	13.1
**A3**	2.4	16.5	2.5	14.2
**A4**	3.1	16.8	3.1	14.3
**A5**	-4.9	16.8	-5.4	13.7
**A6**	-4.9	18.3	-5.5	15.3
**A7**	-4.8	14.2	-4.7	11.9
**A8**	-1.5	15.4	-1.6	13.0
**A9**	3.7	17.3	3.7	14.9
**A10**	-0.8	15.2	-0.7	12.8
**A11**	1.4	15.8	1.3	13.4

**A12**	-5.0	15.6	-5.4	12.8
**A13**	1.4	16.2	1.4	13.8
**A14**	4.7	17.9	4.9	15.5
**A15**	2.7	17.1	2.8	14.7
**A16**	-5.4	17.5	-6.0	14.1
**A17**	-5.5	18.9	-6.2	15.8
**A18**	-5.4	25.6	-6.1	22.2
**A19**	5.9	22.0	6.4	19.4
**A20**	5.3	20.1	5.7	17.6
**A21**	5.2	19.7	5.5	17.0
**A22**	-5.4	27.3	-6.3	23.7
**A23**	-5.5	24.2	-6.3	20.5
**A24**	4.8	18.3	5.2	15.9

Table S5. Reaction energies and activation energies for the 1,2-hydride shifts on protonated stilbenes with constrained CCC angles (prevention of barrierless spirocyclisation).

Reactant	ΔE_r / kcal mol^{-1}	$\Delta E^‡$ / kcal mol^{-1}
**Constrained A0**	0.0	14.3
**Constrained A1**	-4.2	13.2
**Constrained A2**	-1.5	14.4
**Constrained A3**	3.4	15.8
**Constrained A4**	4.3	16.2
**Constrained A5**	-9.1	12.4
**Constrained A6**	-11.1	12.0
**Constrained A7**	-5.3	13.3
**Constrained A8**	-2.0	14.3
**Constrained A9**	5.1	16.8
**Constrained A10**	-1.2	14.0
**Constrained A11**	1.8	14.9
**Constrained A12**	-7.6	12.7
**Constrained A13**	2.1	15.4
**Constrained A14**	7.0	17.5
**Constrained A15**	3.7	16.6
**Constrained A16**	-10.7	12.0
**Constrained A17**	-12.0	12.3
**Constrained A18**	-20.5	10.3
**Constrained A19**	14.1	22.0
**Constrained A20**	10.6	19.9
**Constrained A21**	10.4	19.6
**Constrained A22**	-22.5	10.1
**Constrained A23**	-18.2	11.4
**Constrained A24**	6.9	17.8

Table S6. Reaction energies and activation energies for the (3+2) cyclisation of lithium ynolates with nitrones, and reaction energies when using the non-stationary ketene hidden intermediate. All energies in kcal mol^{-1}.

Reactant	ΔE_r	$\Delta E^‡$	ΔG_r	$\Delta G^‡$	ΔE_r(ketene)	ΔG_r(ketene)
**C0**	-33.2	15.5	-28.8	16.9	-0.9	1.1
**C2**	-33.5	15.8	-32.3	13.8	-0.4	-0.9
**C6**	-32.5	15.2	-29.3	14.7	-1.2	0.5
**C8**	-33.8	16.0	-31.4	14.7	-0.3	1.3
**C9**	-34.7	16.5	-31.2	16.1	0.3	2.4

**C14**	-35.3	16.5	-30.4	17.4	0.4	3.8
**C15**	-34.4	16.5	-29.9	17.1	0.4	3.1
**C18**	-32.2	15.1	-27.9	14.9	-1.5	0.6
**C19**	-36.9	17.5	-33.7	17.7	2.2	3.8
**C21**	-36.3	17.0	-32.4	16.4	1.1	2.1

In the gold(I)-mediated 6-*endo*-dig cyclisation of a 1,5-enyne, some of the initial reactions lead to a benzyl cation intermediate whereas other reactions are concerted asynchronous leading directly to the α-cyclopropylcarbene complex. For clarity, in Table S4, we show the energies corresponding to the benzyl cations, also for the concerted asynchronous reactions (where applicable, the values are indicated as constrained geometries, obtained from the IRC-derived angles). Table S5, shows the energies corresponding to the second process, with relative energies referenced to the corresponding benzyl cation.

Table S7. Reaction energies and activation energies for the initial 6-*endo*-dig cyclisation; products used for energies are benzyl cations in all cases (constrained are marked).

Reactant	ΔE_r / kcal mol^{-1}	$\Delta E^‡$ / kcal mol^{-1}	ΔG_r / kcal mol^{-1}	$\Delta G^‡$ / kcal mol^{-1}
**E0**	0.6	18.1	2.4	17.6
**E1**	-1.8	18.0	0.1	17.2
**E2**	1.7	18.6	4.2	18.6
**E3**	4.8	20.1	5.3	18.7
**E4**[a]	4.6	19.1	7.2	18.4
**E5**	-5.6	17.5	-3.2	17.0
**E6**	-5.8	18.1	-3.7	17.3
**E7**	-0.4	18.6	1.7	17.7
**E8**	2.1	18.7	4.0	18.5
**E9**[a]	5.4	19.1	6.9	18.9
**E10**	1.4	19.3	3.0	18.1
**E11**	2.2	18.6	4.0	18.1
**E12**	-2.7	18.1	-0.2	18.4
**E13**	3.7	19.6	4.5	17.9
**E14**[a]	6.9	19.5	7.8	18.0
**E15**[a]	4.8	19.3	7.2	19.0
**E16**	-4.3	18.0	-2.0	17.4
**E17**	-5.7	18.4	-3.8	17.6
**E18**	-14.2	17.7	-11.6	16.8
**E19**[a]	10.5	21.4	11.4	20.2
**E20**[a]	10.9	21.6	12.1	20.2
**E21**[a]	9.5	20.4	12.3	19.8
**E22**	-15.2	17.9	-12.4	16.9
**E23**	-10.2	17.9	-8.0	17.2
**E24**[a]	7.3	20.7	8.1	19.7

[a]These reactions are concerted asynchronous; the energies used for the product benzyl cation consequently correspond to the IRC-derived constrained geometry optimisation as explained in the previous section.

Table S8. Reaction energies using the alternative constrained products, with the CCC angle fixed at 102.1° for all product geometries.

Product	ΔE_r / kcal mol^{-1}	ΔG_r / kcal mol^{-1}	$\Delta\alpha^a$ / °	$\Delta\Delta E_r^a$ / kcal mol^{-1}
**alt-constrF4**	4.6	6.6	-0.13	0.0
**alt-constrF9**	5.8	7.7	1.71	0.4
**alt-constrF14**	6.9	8.1	2.25	0.0
**alt-constrF15**	4.8	6.9	-0.10	-0.1

**alt-constrF19**	12.4	12.7	7.50	1.9
**alt-constrF20**	11.4	12.1	5.85	0.5
**alt-constrF21**	9.8	11.4	6.28	0.4
**alt-constrF24**	7.3	8.5	2.37	0.0

[a]Difference with respect to angles and reaction energies of the IRC-derived constrained model of the hidden intermediate.

Table S9. Reaction energies and activation energies for the subsequent formation of the cyclopropane; reactants used for energies are benzyl cations in all cases (constrained are marked).

**Product**	**ΔE_r / kcal mol^{-1}**	**Δ$E^‡$ / kcal mol^{-1}**	**ΔG_r / kcal mol^{-1}**	**Δ$G^‡$ / kcal mol^{-1}**
**G0**	-6.0	—[a]	-6.5	—[a]
**G1**	-3.5	0.5	-3.6	0.1
**G2**	-6.6	—[a]	-7.0	—[a]
**G3**	-8.3	—[a]	-8.2	—[a]
**G4[b]**	-9.3	—[b]	-10.5	—[b]
**G5**	-0.2	2.5	-0.7	1.6
**G6**	0.6	3.1	-0.1	2.5
**G7**	-4.3	—[a]	-4.6	—[a]
**G8**	-7.0	—[a]	-6.9	—[a]
**G9[b]**	-10.1	—[b]	-9.7	—[b]
**G10**	-5.5	—[a]	-6.1	—[a]
**G11**	-7.0	—[a]	-7.1	—[a]
**G12**	-2.5	1.1	-2.6	1.0
**G13**	-7.6	—[a]	-7.8	—[a]
**G14[b]**	-11.3	—[b]	-11.5	—[b]
**G15[b]**	-9.4	—[b]	-10.0	—[b]
**G16**	-0.8	2.2	-1.5	1.7
**G17**	0.6	3.2	0.1	2.8
**G18**	9.1	9.4	8.1	8.3
**G19[b]**	-13.7	—[b]	-13.5	—[b]
**G22[b]**	-13.6	—[b]	-13.9	—[b]
**G21[b]**	-13.3	—[b]	-14.5	—[b]
**G22**	10.3	13.2	8.7	12.0
**G23**	5.0	7.8	4.3	6.7
**G24[b]**	-10.5	—[b]	-10.3	—[b]

[a]Low-lying torsional TS not found. [b]These reactions come from the barrierless concerted asynchronous process; reaction energies are referenced to the constrained benzyl cations

2. Computed Structures and Energies

Table S10. Hydride shifts on unconstrained protonated stilbenes. Low level of theory potential and free energies reported at ωB97X-D3(BJ)/def2-TZVP(-f). HLT energies reported at ωB97X-D3/def2-TZVPPD.

Code	E / Ha	G / Ha	E_{HLT} / Ha
A0	-541.48262	-541.29036	-541.50065
A1	-580.84043	-580.62294	-580.85979
A2	-1001.09365	-1000.91273	-1001.11463
A3	-878.69813	-878.50618	-878.72403
A4	-878.69777	-878.50561	-878.72366
A5	-616.75353	-616.55681	-616.77584
A6	-656.09377	-655.87045	-656.11650
A7	-618.94605	-618.72358	-618.96727
A8	-617.68088	-617.48184	-617.70153
A9	-633.77098	-633.58264	-633.79173
A10	-580.83957	-580.62181	-580.85890
A11	-616.74799	-616.55285	-616.76995
A12	-658.29598	-658.04356	-658.31922
A13	-654.86862	-654.66989	-654.88990
A14	-746.08236	-745.89106	-746.10921
A15	-839.30495	-839.14064	-839.33006
A16	-692.01853	-691.81876	-692.04459
A17	-770.69111	-770.43748	-770.71799
A18	-675.59273	-675.32988	-675.61713
A19	-930.64021	-930.45702	-930.67249
A20	-1420.49323	-1420.30072	-1420.53559
A21	-944.62094	-944.44858	-944.65236
A22	-753.09528	-752.79517	-753.12149
A23	-774.86095	-774.60698	-774.88742
A24	-1215.91349	-1215.72156	-1215.94723
B0	-541.48262	-541.29036	-541.50065
B1	-580.84547	-580.62816	-580.86481
B2	-1001.09537	-1000.91448	-1001.11650
B3	-878.69428	-878.50212	-878.72027
B4	-878.69275	-878.50059	-878.71871
B5	-616.76181	-616.56594	-616.78361
B6	-656.10220	-655.87975	-656.12434
B7	-618.95420	-618.73169	-618.97485
B8	-617.68308	-617.48421	-617.70389
B9	-633.76499	-633.57657	-633.78590
B10	-580.84085	-580.62302	-580.86015
B11	-616.74579	-616.55073	-616.76779
B12	-658.30447	-658.05271	-658.32713
B13	-654.86625	-654.66753	-654.88759
B14	-746.07433	-745.88278	-746.10168
B15	-839.30063	-839.13612	-839.32581
B16	-692.02766	-691.82884	-692.05320
B17	-770.70043	-770.44789	-770.72680
B18	-675.60188	-675.34025	-675.62570
B19	-930.63022	-930.44621	-930.66309
B20	-1420.48419	-1420.29110	-1420.52710
B21	-944.61207	-944.43920	-944.64408
B22	-753.10444	-752.80576	-753.13010
B23	-774.87024	-774.61750	-774.89614

**B24**	-1215.90531	-1215.71271	-1215.93956
**TSAB0**	-541.45734	-541.26888	-541.47612
**TSAB1**	-580.81607	-580.60262	-580.83613
**TSAB2**	-1001.06819	-1000.89109	-1001.08996
**TSAB3**	-878.67113	-878.48286	-878.69778
**TSAB4**	-878.67025	-878.48206	-878.69691
**TSAB5**	-616.72636	-616.53468	-616.74901
**TSAB6**	-656.06440	-655.84587	-656.08730
**TSAB7**	-618.92321	-618.70444	-618.94461
**TSAB8**	-617.65551	-617.46032	-617.67694
**TSAB9**	-633.74265	-633.55815	-633.76418
**TSAB10**	-580.81460	-580.60076	-580.83464
**TSAB11**	-616.72214	-616.53081	-616.74485
**TSAB12**	-658.27101	-658.02303	-658.29437
**TSAB13**	-654.84211	-654.64715	-654.86414
**TSAB14**	-746.05317	-745.86569	-746.08074
**TSAB15**	-839.27695	-839.11644	-839.30284
**TSAB16**	-691.99022	-691.79576	-692.01674
**TSAB17**	-770.66070	-770.41213	-770.68783
**TSAB18**	-675.55175	-675.29429	-675.57637
**TSAB19**	-930.60440	-930.42534	-930.63744
**TSAB20**	-1420.46037	-1420.27195	-1420.50348
**TSAB21**	-944.58879	-944.42072	-944.62099
**TSAB22**	-753.05156	-752.75721	-753.07802
**TSAB23**	-774.82219	-774.57406	-774.84890
**TSAB24**	-1215.88361	-1215.69549	-1215.91812

Table S11. Hydride shifts on constrained protonated stilbenes. Low level of theory potential and free energies reported at ωB97X-D3(BJ)/def2-TZVP(-f). HLT energies reported at ωB97X-D3/def2-TZVPPD.

Code	E / Ha	E_{HLT} / Ha
**constrA0**	-541.48098	-541.49901
**constrA1**	-580.83793	-580.85726
**constrA2**	-1001.09201	-1001.11300
**constrA3**	-878.69716	-878.72303
**constrA4**	-878.69687	-878.72276
**constrA5**	-616.74674	-616.76874
**constrA6**	-656.08423	-656.10646
**constrA7**	-618.94516	-618.96580
**constrA8**	-617.67901	-617.69968
**constrA9**	-633.77023	-633.79098
**constrA10**	-580.83766	-580.85695
**constrA11**	-616.74660	-616.76859
**constrA12**	-658.29195	-658.31459
**constrA13**	-654.86750	-654.88880
**constrA14**	-746.08175	-746.10861
**constrA15**	-839.30416	-839.32933
**constrA16**	-692.01004	-692.03598
**constrA17**	-770.68108	-770.70752
**constrA18**	-675.56888	-675.59286
**constrA19**	-930.64004	-930.67230
**constrA20**	-1420.49283	-1420.53523
**constrA21**	-944.62058	-944.65202
**constrA22**	-753.06832	-753.09416

**constrA23**	-774.84117	-774.86716
**constrA24**	-1215.91281	-1215.94653
**constrB0**	-541.48098	-541.49901
**constrB1**	-580.84458	-580.86396
**constrB2**	-1001.09417	-1001.11532
**constrB3**	-878.69171	-878.71769
**constrB4**	-878.68998	-878.71593
**constrB5**	-616.76140	-616.78321
**constrB6**	-656.10191	-656.12408
**constrB7**	-618.95353	-618.97422
**constrB8**	-617.68201	-617.70284
**constrB9**	-633.76190	-633.78279
**constrB10**	-580.83946	-580.85881
**constrB11**	-616.74379	-616.76578
**constrB12**	-658.30402	-658.32669
**constrB13**	-654.86414	-654.88548
**constrB14**	-746.07050	-746.09739
**constrB15**	-839.29820	-839.32338
**constrB16**	-692.02737	-692.05292
**constrB17**	-770.70026	-770.72662
**constrB18**	-675.60185	-675.62564
**constrB19**	-930.61775	-930.65031
**constrB20**	-1420.47581	-1420.51826
**constrB21**	-944.60425	-944.63579
**constrB22**	-753.10440	-753.13005
**constrB23**	-774.87013	-774.89609
**constrB24**	-1215.90163	-1215.93552

Table S12. Lithium ynolate (3+2) cyclisations. Low level of theory potential and free energies reported at ωB97X-D3(BJ)/def2-SVP+ma-def2-SVP[O]. HLT energies reported at ωB97X-D3(BJ)/def2-TZVPPD. Implicit solvation (tetrahydrofuran, SMD) is included in all energies.

Code / Filename	E / Ha	G / Ha	E_{HLT} / Ha
**C0**	-948.92240	-948.60113	-949.95681
**C2**	-1408.38294	-1408.06792	-1409.57685
**C6**	-1063.39916	-1063.04621	-1064.56089
**C8**	-1025.04641	-1024.71503	-1026.16301
**C9**	-1041.12824	-1040.80924	-1042.26752
**C14**	-1153.32111	-1153.00104	-1154.58604
**C15**	-1246.39287	-1246.10023	-1247.79789
**C18**	-1082.86339	-1082.47297	-1084.04137
**C19**	-1337.70249	-1337.38713	-1339.17695
**C21**	-1351.62852	-1351.32480	-1353.13618
**D0**	-948.97945	-948.65116	-950.00975
**D2**	-1408.44047	-1408.12350	-1409.63027
**D6**	-1063.45520	-1063.09720	-1064.61268
**D8**	-1025.10449	-1024.76924	-1026.21692
**D9**	-1041.18750	-1040.86289	-1042.32283
**D14**	-1153.38152	-1153.05357	-1154.64236
**D15**	-1246.45186	-1246.15209	-1247.85264
**D18**	-1082.91884	-1082.52152	-1084.09273
**D19**	-1337.76517	-1337.44467	-1339.23575
**D21**	-1351.69079	-1351.38094	-1353.19396
**TSCD0**	-948.89886	-948.57533	-949.93207

**TSCD2**	-1408.35892	-1408.04704	-1409.55165
**TSCD6**	-1063.37603	-1063.02401	-1064.53662
**TSCD8**	-1025.02201	-1024.69284	-1026.13744
**TSCD9**	-1041.10312	-1040.78469	-1042.24121
**TSCD14**	-1153.29555	-1152.97404	-1154.55972
**TSCD15**	-1246.36793	-1246.07430	-1247.77162
**TSCD18**	-1082.84075	-1082.45059	-1084.01736
**TSCD19**	-1337.67540	-1337.35971	-1339.14902
**TSCD21**	-1351.60232	-1351.29955	-1353.10908
**constrD0**	-948.92534	-948.60082	-949.95829
**constrD2**	-1408.38511	-1408.07086	-1409.57754
**constrD6**	-1063.40258	-1063.04693	-1064.56285
**constrD8**	-1025.04832	-1024.71435	-1026.16346
**constrD9**	-1041.12948	-1040.80708	-1042.26704
**constrD14**	-1153.32177	-1152.99625	-1154.58546
**constrD15**	-1246.39407	-1246.09698	-1247.79732
**constrD18**	-1082.86756	-1082.47381	-1084.04378
**constrD19**	-1337.70022	-1337.38236	-1339.17337
**constrD21**	-1351.62838	-1351.32298	-1353.13444

Table S13. LLT electronic energies (in Ha) for distorted ketene pseudominima at different angles (other than 180.0°, listed in Table S12). Energies reported at ωB97X-D3(BJ)/def2-SVP+ma-def2-SVP[O]. Implicit solvation (tetrahydrofuran, SMD) is included in all energies.

Structure	179.5°	179.0°	178.5°	178.0°
**constrD0**[a]	-948.92534	-948.92559	-948.92585	-948.92612
**constrD2**[a]	-1408.38511	-1408.38564	-1408.38605	-1408.38648
**constrD6**[a]	-1063.40258	-1063.40291	-1063.40323	-1063.40356
**constrD8**[a]	-1025.04832	-1025.04879	-1025.04920	-1025.04964
**constrD9**[a]	-1041.12948	-1041.12992	-1041.13033	-1041.13081
**constrD14**[a]	-1153.32177	-1153.32220	-1153.32267	-1153.32316
**constrD15**[a]	-1246.39407	-1246.39442	-1246.39478	-1246.39516
**constrD18**[a]	-1082.86756	-1082.86782	-1082.86810	-1082.86838
**constrD19**[a]	-1337.70022	-1337.70079	-1337.70136	-1337.70199
**constrD21**[a]	-1351.62838	-1351.62891	-1351.62949	-1351.63010

[a]Named in repository suffixed with ketene angle using a hyphen instead of a point as decimal separator, e.g. constrD0_179-5.

Table S14. HLT electronic energies (in Ha) for distorted ketene pseudominima at different angles (other than 180.0°, listed in Table S12). Energies reported at ωB97X-D3(BJ)/def2-TZVPPD. Implicit solvation (tetrahydrofuran, SMD) is included in all energies.

Structure	179.5°	179.0°	178.5°	178.0°
**constrD0**[a]	-949.95829	-949.95853	-949.95878	-949.95903
**constrD2**[a]	-1409.57754	-1409.57777	-1409.57814	-1409.57852
**constrD6**[a]	-1064.56285	-1064.56311	-1064.56340	-1064.56371
**constrD8**[a]	-1026.16346	-1026.16365	-1026.16402	-1026.16440
**constrD9**[a]	-1042.26704	-1042.26744	-1042.26783	-1042.26826
**constrD14**[a]	-1154.58546	-1154.58586	-1154.58626	-1154.58668
**constrD15**[a]	-1247.79732	-1247.79758	-1247.79790	-1247.79822
**constrD18**[a]	-1084.04378	-1084.04404	-1084.04430	-1084.04458
**constrD19**[a]	-1339.17337	-1339.17384	-1339.17436	-1339.17491
**constrD21**[a]	-1353.13444	-1353.13488	-1353.13540	-1353.13593

[a]Named in repository suffixed with ketene angle using a hyphen instead of a point as decimal separator, e.g. constrD0_179-5.

Table S15. Gold(I)-catalysed 6-*endo*-dig cyclisations. Low level of theory potential and free energies reported at ωB97X-D3(BJ)/def2-TZVP(-f). HLT energies reported at ωB97X-D3(BJ)/def2-TZVPPD. Implicit solvation (dichloromethane, SMD) is included in all energies. Geometries labelled "alt-constr" correspond to those with the angle constrained to a constant 102.1° (instead of the preferable variable constrain extracted from the IRC). Note that transition states referred to as **TSEG** in the manuscript are listed here as **TSEF** as no disambiguation is needed.

Code	E / Ha	G / Ha	E_{HLT} / Ha
**E0**	-1061.80424	-1061.53235	-1061.82906
**E1**	-1101.16039	-1100.86297	-1101.18649
**E2**	-1521.42043	-1521.16028	-1521.44828
**E3**	-1399.02927	-1398.75595	-1399.06203
**E4**	-1399.02715	-1398.75500	-1399.06001
**E5**	-1137.07270	-1136.79773	-1137.10115
**E6**	-1176.40687	-1176.10468	-1176.43577
**E7**	-1139.27089	-1138.96849	-1139.29820
**E8**	-1138.00721	-1137.72867	-1138.03464
**E9**	-1154.10764	-1153.83951	-1154.13536
**E10**	-1101.16211	-1100.86383	-1101.18818
**E11**	-1137.07352	-1136.79894	-1137.10197
**E12**	-1178.61772	-1178.28744	-1178.64705
**E13**	-1175.20227	-1174.92179	-1175.23072
**E14**	-1266.41870	-1266.14623	-1266.45265
**E15**	-1359.63511	-1359.39136	-1359.66731
**E16**	-1212.33946	-1212.06173	-1212.37152
**E17**	-1291.00437	-1290.67185	-1291.03733
**E18**	-1195.88929	-1195.54831	-1195.91979
**E19**	-1451.00003	-1450.73469	-1451.03967
**E20**	-1940.83968	-1940.56581	-1940.88928
**E21**	-1464.96203	-1464.70934	-1465.00070
**E22**	-1273.38992	-1273.01262	-1273.42251
**E23**	-1295.16555	-1294.83323	-1295.19808
**E24**	-1736.24939	-1735.97636	-1736.29019
**F0**	-1061.80447	-1061.52967	-1061.82814
**F1**	-1101.16439	-1100.86398	-1101.18934
**F2**	-1521.41873	-1521.15474	-1521.44550
**F3**	-1399.02254	-1398.74841	-1399.05432
**constrF4**	-1399.02089	-1398.74465	-1399.05268
**F5**	-1137.08272	-1136.80392	-1137.11003
**F6**	-1176.41726	-1176.11169	-1176.44504
**F7**	-1139.27269	-1138.96696	-1139.29889
**F8**	-1138.00489	-1137.72342	-1138.03126
**constrF9**	-1154.10013	-1153.82956	-1154.12676
**F10**	-1101.16105	-1100.86024	-1101.18600
**F11**	-1137.07100	-1136.79342	-1137.09854
**F12**	-1178.62316	-1178.28892	-1178.65131
**F13**	-1175.19757	-1174.91586	-1175.22486
**constrF14**	-1266.40885	-1266.13482	-1266.44171
**constrF15**	-1359.62839	-1359.38091	-1359.65963
**F16**	-1212.34725	-1212.06576	-1212.37844
**F17**	-1291.01427	-1290.67881	-1291.04634
**F18**	-1195.91314	-1195.56805	-1195.94237
**constrF19**	-1450.98433	-1450.71750	-1451.02293

**constrF20**	-1940.82322	-1940.54747	-1940.87190
**constrF21**	-1464.94794	-1464.69065	-1464.98564
**F22**	-1273.41558	-1273.03385	-1273.44671
**F23**	-1295.18291	-1294.84709	-1295.21436
**constrF24**	-1736.23863	-1735.96430	-1736.27855
**G0**	-1061.81351	-1061.53948	-1061.83771
**G1**	-1101.16945	-1100.86924	-1101.19490
**G2**	-1521.42883	-1521.16537	-1521.45607
**G3**	-1399.03531	-1398.76102	-1399.06757
**G4**	-1399.03526	-1398.76092	-1399.06752
**G5**	-1137.08223	-1136.80431	-1137.11029
**G6**	-1176.41569	-1176.11118	-1176.44414
**G7**	-1139.27902	-1138.97371	-1139.30575
**G8**	-1138.01558	-1137.73399	-1138.04237
**G9**	-1154.11584	-1153.84453	-1154.14291
**G10**	-1101.16926	-1100.86951	-1101.19473
**G11**	-1137.08167	-1136.80416	-1137.10975
**G12**	-1178.62650	-1178.29248	-1178.65525
**G13**	-1175.20922	-1174.92781	-1175.23703
**G14**	-1266.42632	-1266.15262	-1266.45970
**G15**	-1359.64280	-1359.39628	-1359.67459
**G16**	-1212.34760	-1212.06725	-1212.37967
**G17**	-1291.01265	-1290.67790	-1291.04541
**G18**	-1195.89802	-1195.55449	-1195.92788
**G19**	-1451.00561	-1450.73847	-1451.04469
**G20**	-1940.84442	-1940.56911	-1940.89360
**G21**	-1464.96866	-1464.71324	-1465.00689
**G22**	-1273.39841	-1273.01914	-1273.43033
**G23**	-1295.17451	-1294.83977	-1295.20642
**G24**	-1736.25498	-1735.98024	-1736.29531
**TSEF0**	-1061.77627	-1061.50509	-1061.80023
**TSEF1**	-1101.13246	-1100.83634	-1101.15776
**TSEF2**	-1521.39157	-1521.13142	-1521.41857
**TSEF3**	-1398.99791	-1398.72684	-1399.02998
**TSEF4**	-1398.99753	-1398.72641	-1399.02962
**TSEF5**	-1137.04534	-1136.77125	-1137.07321
**TSEF6**	-1176.37880	-1176.07787	-1176.40701
**TSEF7**	-1139.24205	-1138.94108	-1139.26853
**TSEF8**	-1137.97831	-1137.70013	-1138.00487
**TSEF9**	-1154.07817	-1153.81031	-1154.10500
**TSEF10**	-1101.13222	-1100.83577	-1101.15746
**TSEF11**	-1137.04446	-1136.77068	-1137.07231
**TSEF12**	-1178.58966	-1178.25888	-1178.61815
**TSEF13**	-1175.17189	-1174.89421	-1175.19946
**TSEF14**	-1266.38849	-1266.11840	-1266.42162
**TSEF15**	-1359.60509	-1359.36179	-1359.63661
**TSEF16**	-1212.31093	-1212.03421	-1212.34276
**TSEF17**	-1290.97542	-1290.64421	-1291.00798
**TSEF18**	-1195.86181	-1195.52236	-1195.89151
**TSEF19**	-1450.96676	-1450.70334	-1451.00552
**TSEF20**	-1940.80599	-1940.53427	-1940.85491
**TSEF21**	-1464.93034	-1464.67859	-1464.96825
**TSEF22**	-1273.36221	-1272.98651	-1273.39403
**TSEF23**	-1295.13785	-1294.80659	-1295.16960
**TSEF24**	-1736.21717	-1735.94563	-1736.25726

**TSFG1**	-1101.16361	-1100.86370	-1101.18862
**TSFG5**	-1137.07844	-1136.80113	-1137.10599
**TSFG6**	-1176.41218	-1176.10758	-1176.44015
**TSFG12**	-1178.62118	-1178.28716	-1178.64952
**TSFG16**	-1212.34340	-1212.06268	-1212.37488
**TSFG17**	-1291.00894	-1290.67413	-1291.04128
**TSFG18**	-1195.89764	-1195.55436	-1195.92735
**TSFG22**	-1273.39365	-1273.01397	-1273.42562
**TSFG23**	-1295.17013	-1294.83605	-1295.20197
**alt-constrF4**	-1399.02086	-1398.74557	-1399.05267
**alt-constrF9**	-1154.09952	-1153.82830	-1154.12617
**alt-constrF14**	-1266.40886	-1266.13446	-1266.44170
**alt-constrF15**	-1359.62848	-1359.38126	-1359.65973
**alt-constrF19**	-1450.98137	-1450.71554	-1451.01997
**alt-constrF20**	-1940.82245	-1940.54750	-1940.87113
**alt-constrF21**	-1464.94737	-1464.69221	-1464.98507
**alt-constrF24**	-1736.23859	-1735.96373	-1736.27853

3. REFERENCES

[1] F. Neese, “Software update: the ORCA program system, version 6.0,” *WIREs Comput. Molec. Sci.* 15 (2025): e70019.

[2] F. Neese, “An improvement of the resolution of the identity approximation for the formation of the Coulomb matrix,” *J. Comp. Chem.* 24 (2003): 1740–1747.

[3] Y.-S. Lin, G.-D. Li, S.-P. Mao, and J.-D. Chai, “Long-Range Corrected Hybrid Density Functionals with Improved Dispersion Corrections.” *J. Chem. Theory Comput.* 9 (2013): 263–272.

[4] F. Weigend and R. Ahrlichs, “Balanced basis sets of split valence, triple zeta valence and quadruple zeta valence quality for H to Rn: Design and assessment of accuracy,” *Phys. Chem. Chem. Phys.* 7 (2005): 3297–3305.

[5] A. V. Marenich, C. J. Cramer, and D. G. Truhlar, “Universal Solvation Model Based on Solute Electron Density and on a Continuum Model of the Solvent Defined by the Bulk Dielectric Constant and Atomic Surface Tensions,” *J. Phys. Chem. B* 113 (2009): 6378–6396.

[6] D. Andrae, U. Haeussermann, M. Dolg, H. Stoll, and H. Preuss, “Energy-adjusted ab initio pseudopotentials for the second and third row transition elements,” *Theor. Chim. Acta*, 77 (1990): 123-141.

[7] M. Page and J. W. McIver Jr., “On evaluating the reaction path Hamiltonian,” *J. Chem. Phys.* 88 (1988): 922–935.

[8] M. Page, C. Doubleday, and J. W. McIver Jr., “Following steepest descent reaction paths. The use of higher energy derivatives with ab initio electronic structure methods,” *J. Chem. Phys.* 93 (1990): 5634–5642.

[9] J. M. Bofill, *J. Comput. Chem.* 19 (1998): 349–362.

[10] H. P. Hratchian and H. B. Schlegel, “Using Hessian Updating To Increase the Efficiency of a Hessian Based Predictor-Corrector Reaction Path Following Method,” *J. Chem. Theory Comput.* 1 (2005): 61–69.

[11] M. Álvarez-Moreno, C. de Graaf, N. Lopez, F. Maseras, J. M. Poblet, and C. Bo, “Managing the Computational Chemistry Big Data Problem: The ioChem-BD Platform,” *J. Chem. Inf. Model.* 55 (2015): 95−103.

[12] C. Barrales-Martínez and P. Jaque, “A deeper analysis of the role of synchronicity on the Bell–Evans–Polanyi plot in multibond chemical reactions: a path-dependent reaction force constant,” *Phys. Chem. Chem. Phys.* (2022) 24: 14772–14779.

4. Coordinates of Stationary Points

A0

C	-1.03260921	-3.66921585	0.54092896
C	-0.27181064	-3.73837875	-0.62157022
C	0.63253976	-2.73079701	-0.91879540
C	0.78970199	-1.65945280	-0.03998068
C	0.00624523	-1.57771317	1.10959821
C	-0.89277920	-2.59077786	1.40691413
H	-1.73777008	-4.45910354	0.77213180
H	-0.37899876	-4.58214363	-1.29285863
H	1.23765549	-2.79145267	-1.81773373
H	0.10929619	-0.73209715	1.78201512
H	-1.48580658	-2.53845122	2.31224719
C	1.79532268	-0.54187838	-0.36102416
H	2.27270059	-0.15474826	0.53487707
H	2.54556242	-0.90886557	-1.06129089
H	0.52672223	0.08001702	-1.99847185
C	0.27779459	1.53375378	-0.47697762
C	-0.72313799	2.18722208	-1.24189327
C	0.66909409	2.08233829	0.77259601
C	-1.31215291	3.34021231	-0.77508083
H	-1.01783517	1.76445278	-2.19638585
C	0.07638657	3.23378946	1.22901737
H	1.44036412	1.60181460	1.36087591
C	-0.91121398	3.85703525	0.45679375
H	-2.07648030	3.84259432	-1.35454148
H	0.37131309	3.66419833	2.17793305
H	-1.37291804	4.76706137	0.82517568
C	0.86090666	0.38134968	-1.00743884

*

A1

C	-0.55823592	-3.33383015	0.29422651
C	0.29626541	-3.26275953	-0.80922753
C	1.13950669	-2.18129557	-0.98770005
C	1.15920912	-1.14890800	-0.04697661
C	0.28293270	-1.19152842	1.03755567
C	-0.55163235	-2.28095528	1.21184727
H	0.30120141	-4.06993702	-1.53349982
H	1.80624523	-2.15016085	-1.84379190
H	0.26233702	-0.37763493	1.75487135
H	-1.21419980	-2.31616579	2.06957760
C	2.10720649	0.04879535	-0.23138242
H	2.43702701	0.46066564	0.71772333
H	2.95809648	-0.23241042	-0.84940002
H	0.94913741	0.55176178	-1.98513958
C	0.31803771	1.88852189	-0.45878011
C	-0.69508529	2.40325045	-1.30260244
C	0.50645864	2.45297372	0.82655816
C	-1.49617537	3.43980875	-0.87545796
H	-0.83432796	1.96991216	-2.28723184
C	-0.29579051	3.48834607	1.24374353
H	1.28797974	2.08061398	1.47676146
C	-1.29490682	3.97644631	0.39490633
H	-2.27273104	3.83568200	-1.51792373
H	-0.15455472	3.93055827	2.22219878
H	-1.92288155	4.79407032	0.73227794
C	1.12405908	0.85085791	-0.95403104
C	-1.44730778	-4.52830842	0.49864977
H	-0.90621822	-5.30475070	1.04732306
H	-2.33464795	-4.26866905	1.07724304
H	-1.76192510	-4.95464267	-0.45491258

*

A10

C	-0.98029800	-3.12337196	-1.09831636
C	0.13650133	-3.37041152	-0.29388995
C	0.49226692	-2.41139775	0.64498592
C	-0.26092739	-1.24541006	0.79574058
C	-1.35211655	-1.00173116	-0.03227606
C	-1.71888256	-1.95653447	-0.97046194
H	1.34819701	-2.58684964	1.29022598
H	-1.92159997	-0.08322673	0.06208030
H	-2.58009429	-1.78756851	-1.60610159
C	0.14720547	-0.20151462	1.84964297
H	-0.71472736	0.28120596	2.30129723
H	0.76824429	-0.66777316	2.61377825
H	1.89050683	0.23273677	0.64868311
C	0.55985263	1.85920708	0.36232189
C	1.42896644	2.42078876	-0.60687410
C	-0.62555917	2.55177689	0.71951499
C	1.12348801	3.62512403	-1.20006284
H	2.33474757	1.88810969	-0.87600443
C	-0.92122351	3.75343452	0.12314778
H	-1.29216451	2.14122782	1.46721398
C	-0.04928588	4.28471552	-0.83415564
H	1.78382951	4.05768387	-1.94117868
H	-1.82082023	4.29272151	0.39271247
H	-0.29097989	5.23464850	-1.29904631
C	0.93006668	0.64545007	0.95098285
H	-1.27141777	-3.86145462	-1.83858681
C	0.91306239	-4.65224819	-0.43714385
H	1.82411842	-4.63655097	0.16210098
H	1.18922531	-4.82307638	-1.47977657
H	0.30827459	-5.50253495	-0.11262813

*

A11

C	-0.97043118	-3.25390159	-1.06559502
C	0.09612640	-3.46879452	-0.19139423
C	0.44412517	-2.48779248	0.73158089
C	-0.28841256	-1.30795024	0.78517063
C	-1.33566650	-1.07496148	-0.10345095
C	-1.67922571	-2.06352561	-1.01547673
H	1.26307307	-2.68042173	1.41567510
H	-1.88971009	-0.14345979	-0.07354690
H	-2.50577308	-1.90574539	-1.69819342
C	0.09697501	-0.22204604	1.80076989
H	-0.77685546	0.26231760	2.22785200
H	0.70926824	-0.65823810	2.59074730
H	1.85959772	0.21209521	0.62600855
C	0.55307406	1.85635608	0.35162800
C	1.45287300	2.43515137	-0.58189224
C	-0.64192057	2.54663073	0.68817668
C	1.16918605	3.65235751	-1.15728079
H	2.36396681	1.90384232	-0.83532332
C	-0.91528382	3.76098153	0.10900238
H	-1.33258377	2.12135090	1.40505290
C	-0.01216098	4.30834563	-0.81047210
H	1.85157943	4.09855587	-1.86973499
H	-1.82085628	4.29837959	0.36163568
H	-0.23676580	5.26902895	-1.26187362
C	0.89989155	0.63207430	0.92180142
H	-1.24363645	-4.02029372	-1.78405250
O	0.83023146	-4.60542689	-0.18685207
H	0.48949322	-5.22915344	-0.83756690

*

A12

C	-0.31101955	-2.48908612	0.30993008
C	0.91461347	-2.38153957	-0.38570649
C	1.64499082	-1.23096789	-0.32214770
C	1.18929866	-0.11529770	0.43517310
C	-0.04923307	-0.23027917	1.12421277
C	-0.76996298	-1.38734684	1.06604739
H	1.28080345	-3.21514052	-0.97130659
H	2.58956070	-1.15543317	-0.85179865
H	-0.42496752	0.62184335	1.67914715
H	-1.71274512	-1.46747726	1.59366118

C	2.23680967	0.94423922	0.91670141
H	1.98997234	1.37143756	1.87945540
H	3.26024976	0.60828229	0.81257057
H	1.98494008	1.19145391	-1.21018540
C	0.43059945	2.38240305	-0.25983039
C	-0.39794845	2.46051533	-1.37973417
C	0.23307735	3.25930306	0.80503893
C	-1.42366568	3.39032353	-1.42608111
H	-0.23837921	1.78674600	-2.21573244
C	-0.79294778	4.19321507	0.75520876
H	0.89116916	3.23991233	1.66632697
C	-1.62437442	4.25555418	-0.35554754
H	-2.06411132	3.44621027	-2.29840644
H	-0.93543798	4.88050473	1.58084570
H	-2.42302627	4.98728171	-0.39308099
C	1.51538002	1.37551899	-0.25104032
C	-1.12164207	-3.70228345	0.26326992
C	-1.16192962	-4.54330484	-1.00782474
C	-0.44494468	-5.06583078	0.17526728
H	-2.03796731	-3.65938616	0.83763744
H	-0.58690205	-4.18467912	-1.85225343
H	-2.12682177	-4.96372698	-1.25927519
H	-0.90538780	-5.85355583	0.75701360
H	0.63767759	-5.07762184	0.16888537

*

A13

C	-1.28606121	-2.95711719	-0.57905077
C	-0.05396416	-3.15739480	0.04204408
C	0.51671319	-2.14747639	0.80295238
C	-0.14985514	-0.93418996	0.95793402
C	-1.37000095	-0.73050749	0.31778853
C	-1.94420609	-1.74553722	-0.43800707
H	1.47079480	-2.31899791	1.29448808
H	-1.88567981	0.21928065	0.42002757
H	-2.90320283	-1.58619748	-0.91631730
C	0.47153201	0.19446530	1.79205025
H	-0.27795876	0.72787578	2.37088110
H	1.23106911	-0.21644163	2.45853928
H	1.96236011	0.53059498	0.25810703
C	0.64865421	2.18584672	0.17098798
C	1.34490474	2.69678673	-0.95841720
C	-0.44709376	2.91872335	0.70485738
C	0.96052380	3.88643474	-1.53135853
H	2.18033989	2.13428515	-1.36126707
C	-0.82173714	4.10483466	0.12580104
H	-0.97951125	2.54771487	1.57131259
C	-0.11972047	4.58322845	-0.98824484
H	1.48651981	4.28055579	-2.39176472
H	-1.65141602	4.67416191	0.52595995
H	-0.42330188	5.52270901	-1.43824655
C	1.08967372	0.98694579	0.72238384
H	-1.70856250	-3.76439207	-1.16622411
C	0.65457750	-4.46590046	-0.10789360
O	0.23241976	-5.36979651	-0.77735822
H	1.61157334	-4.55339466	0.44431206

*

A14

C	0.04032073	-2.96110502	0.25614276
C	1.20218660	-2.80648555	-0.47953952
C	1.83729663	-1.57416066	-0.46142360
C	1.31167376	-0.53009714	0.29561842
C	0.13235020	-0.70919816	1.01494781
C	-0.50921060	-1.93800387	1.00779992
H	1.59408957	-3.64069283	-1.04574207
H	2.75597303	-1.43694159	-1.02139566
H	-0.28448883	0.10600758	1.59707359
H	-1.41818052	-2.11167238	1.56764479
C	2.00064282	0.83817584	0.31078035
H	2.01065492	1.26843415	1.30926716
H	3.02621783	0.73293613	-0.04974308
H	1.31241290	1.28257548	-1.69674512
C	0.29118474	2.61832987	-0.41985041
C	-0.43958794	3.13701626	-1.52728427
C	0.06719151	3.15957453	0.87895588
C	-1.35296292	4.14710699	-1.34418680
H	-0.26514013	2.72145909	-2.51399176
C	-0.84655389	4.16731255	1.04928675
H	0.62121835	2.78195308	1.72880974
C	-1.55226050	4.65590757	-0.05917234
H	-1.91232445	4.54580603	-2.18107851
H	-1.02391264	4.58974001	2.03044689
H	-2.27258448	5.45428115	0.08681985
C	1.20673734	1.60608799	-0.66220436
N	-0.64706346	-4.27740968	0.23605423
O	-0.13941433	-5.15304088	-0.42952118
O	-1.66612976	-4.37507500	0.88486706

*

A15

C	-0.60580713	-3.11729635	0.12996652
C	0.77264774	-3.11583563	-0.05388292
C	1.53414727	-2.00011078	0.24097679
C	0.90478556	-0.86613462	0.74515304
C	-0.47631324	-0.84014823	0.91743543
C	-1.21576176	-1.96957626	0.61982338
H	2.60708403	-2.04908259	0.09749418
H	-0.99529337	0.03339122	1.29433137
C	1.72020984	0.38932778	1.06523061
H	1.40161070	0.84730238	1.99827566
H	2.77889211	0.13140288	1.12914566
H	1.93059648	0.85042510	-1.04306272
C	0.65965498	2.33661133	-0.24228724
C	0.47913626	2.89978049	-1.53755942
C	0.01932802	2.94171242	0.87671074
C	-0.30800834	4.01362362	-1.70651695
H	0.96936821	2.43541490	-2.38652350
C	-0.76364209	4.05276766	0.69600906
H	0.15434800	2.53087293	1.86913946
C	-0.92561582	4.58304356	-0.59135478
H	-0.45038904	4.44638401	-2.68871508
H	-1.25378924	4.52372000	1.53894281
H	-1.54739522	5.46251676	-0.72329326
C	1.47036720	1.21652041	-0.12663460
F	-2.53513243	-1.96970407	0.79298063
F	-1.32243563	-4.19147181	-0.15737652
F	1.35239003	-4.21717797	-0.51990170

*

A16

C	-0.33114248	-2.95973744	-0.37208266
C	0.99985357	-2.85263765	-0.80822804
C	1.71788086	-1.74567790	-0.46568507
C	1.14324191	-0.70020778	0.31502765
C	-0.21969100	-0.83122236	0.74414466
C	-0.93660265	-1.93223104	0.41429163
H	1.41691487	-3.65413324	-1.40439924
H	2.74922817	-1.64878816	-0.78851664
H	-0.67249562	-0.02488210	1.31153171
C	2.10980925	0.19052511	1.16356542
H	1.67972463	0.50261202	2.10577231
H	3.12048462	-0.19071973	1.22401925
H	2.31890219	0.75291045	-0.91340595
C	0.62541654	1.87930639	-0.12304315
C	-0.04177421	2.10086215	-1.32790462
C	0.32701974	2.66986521	0.98350885
C	-1.00885714	3.08865926	-1.41923918
H	0.19811140	1.49479219	-2.19605081
C	-0.64086674	3.66271781	0.88952390
H	0.86518825	2.53864579	1.91559161
C	-1.31242411	3.86887867	-0.30768108

H -1.52256985 3.25693215 -2.35853004
H -0.85975211 4.28360640 1.75042513
H -2.06403881 4.64634783 -0.38044824
C 1.65256043 0.80760833 -0.06047644
O -1.02815345 -4.01985565 -0.70575386
H -1.92667959 -3.95963679 -0.33837561
O -2.22868509 -2.19846790 0.73897004
H -2.63044485 -1.48291581 1.24411009
*

A17
C -0.87964466 -2.23481495 0.07746070
C 0.54568945 -2.31968940 -0.12848201
C 1.31621103 -1.24910784 0.20036619
C 0.73430318 -0.04741824 0.73935245
C -0.67482778 0.00114615 0.92276518
C -1.46441519 -1.06368814 0.60454299
H 2.39001826 -1.28252676 0.06560432
H -1.12183523 0.91349669 1.30066944
H -2.53432981 -1.00884538 0.74471611
C 1.61867516 0.83665496 1.67963595
H 1.07171418 1.29426038 2.49279991
H 2.55264342 0.36779088 1.95929236
H 2.24712521 1.13511743 -0.36737310
C 0.59653616 2.51714936 -0.00780179
C 0.15490304 2.66659021 -1.32250678
C 0.24762443 3.46915377 0.94503801
C -0.64036780 3.74492659 -1.67438174
H 0.43545015 1.93224519 -2.07132989
C -0.54820459 4.55243226 0.59052554
H 0.61310904 3.38844195 1.96262546
C -0.99649246 4.68854226 -0.71552104
H -0.97927610 3.85534084 -2.69789624
H -0.80823283 5.29528389 1.33561951
H -1.61443836 5.53510036 -0.99147633
C 1.45006496 1.34758877 0.33600554
O -1.54881518 -3.30000549 -0.26473215
C -2.98240400 -3.33939588 -0.10237598
H -3.24320925 -3.21903274 0.94997436
H -3.44924756 -2.56654279 -0.71457397
H -3.27767094 -4.32436994 -0.45194859
O 0.96425339 -3.48696253 -0.63723992
C 2.36682382 -3.63836101 -0.85807009
H 2.72630592 -2.89829746 -1.57916339
H 2.91739552 -3.54989670 0.08308873
H 2.49511479 -4.63804932 -1.26477194
*

A18
C -0.08231981 -2.53400372 0.04014075
C 1.18552000 -2.30594268 -0.60736707
C 1.80996148 -1.11770387 -0.47968010
C 1.25239752 -0.03118450 0.28785834
C -0.02155314 -0.27726813 0.91893433
C -0.65869810 -1.46016389 0.81010952
H 1.64204842 -3.08905662 -1.19392482
H 2.76744215 -0.96270054 -0.96742491
H -0.47843062 0.52884245 1.48218790
H -1.61290247 -1.59709221 1.29642196
C 2.23255795 0.95328533 0.97868911
H 1.90046643 1.30035538 1.94815676
H 3.27654655 0.67456017 0.92256887
H 2.12528216 1.41630300 -1.13823168
C 0.44820263 2.43927139 -0.18180237
C -0.47842266 2.46154360 -1.22381967
C 0.33078588 3.35663287 0.85658273
C -1.51596831 3.37983419 -1.21919591
H -0.38452709 1.75337176 -2.04148262
C -0.70980797 4.27935785 0.86063092
H 1.06165567 3.37253687 1.65741888
C -1.63511678 4.28949727 -0.17283641
H -2.23116944 3.39171467 -2.03359171
H -0.78943802 4.99613416 1.66975858
H -2.44410067 5.01078064 -0.17025991
C 1.55298728 1.43706778 -0.21711984
N -0.70273428 -3.69599765 -0.07384762
C -1.98913390 -3.93429622 0.59370934
C -0.12481367 -4.78687131 -0.86947072
H -2.30634510 -4.95201401 0.38702021
H -1.88946093 -3.81465122 1.67365393
H -2.75094789 -3.24927565 0.21751029
H -0.80202322 -5.63521877 -0.84283392
H 0.00364562 -4.47866936 -1.90829910
H 0.83692052 -5.09658094 -0.45686632
*

A19
C -0.24646144 -2.43353278 0.03887501
C 1.14178315 -2.36961451 0.07390845
C 1.75778567 -1.16968779 0.42745960
C 0.99396524 -0.06264985 0.77014534
C -0.39193318 -0.14451165 0.73541677
C -1.02426305 -1.32789834 0.36232690
H 2.84073400 -1.12795404 0.44286663
H -1.00848355 0.70659845 1.00184347
C 1.65471287 1.25892975 1.15607854
H 1.22014643 1.65941057 2.06949802
H 2.72335251 1.09470403 1.32040595
H 2.02808537 1.84054457 -0.90674188
C 0.69230603 3.27124750 -0.12782963
C 0.63672750 3.92499034 -1.39786359
C -0.07399649 3.79377927 0.95976087
C -0.14706037 5.03781256 -1.57114432
H 1.22058038 3.52561958 -2.22034785
C -0.85232031 4.90420877 0.77283005
H -0.03806044 3.31638764 1.93071771
C -0.88729541 5.52020629 -0.48805753
H -0.19621825 5.53747767 -2.53038454
H -1.43824308 5.31086948 1.58759347
H -1.50871099 6.39955444 -0.62450815
C 1.48770500 2.15050423 -0.01309631
N -0.90834347 -3.69477347 -0.35486755
O -0.55864708 -4.68796937 0.23715269
O -1.73598597 -3.60644684 -1.23253740
C -2.46406099 -1.35642686 0.34931300
N -3.60985621 -1.29131532 0.38019119
C 1.97382956 -3.49005817 -0.28109347
N 2.69984458 -4.32852916 -0.57725545
*

A2
C -0.17758476 -3.09245579 0.25076785
C 0.90637078 -2.97487380 -0.61246708
C 1.64705911 -1.80569168 -0.61351290
C 1.31957280 -0.76459940 0.25568967
C 0.21365296 -0.88817969 1.09548476
C -0.52888636 -2.05614433 1.10817165
H 1.16467491 -3.79662103 -1.26812411
H 2.50207302 -1.71609927 -1.27571983
H -0.06459027 -0.07541390 1.75822810
H -1.37613502 -2.17016921 1.77215907
C 2.14060771 0.53274653 0.25420786
H 2.21534068 0.96780315 1.24695610
H 3.13423298 0.34617184 -0.15258939
H 1.37897647 0.96935001 -1.72372352
C 0.35608320 2.26988393 -0.40052072
C -0.45360648 2.74209775 -1.46619261
C 0.19680687 2.82775321 0.89549359
C -1.38853122 3.72758498 -1.24483655
H -0.32726614 2.31405460 -2.45486852
C -0.73766922 3.81148223 1.10575854
H 0.81699093 2.48787968 1.71518722

C -1.52742105 4.25610530 0.03836728
H -2.00933402 4.09062299 -2.05426159
H -0.86367706 4.24782557 2.08884707
H -2.26270306 5.03416024 0.21450101
C 1.30250037 1.28355073 -0.68452105
Cl -1.10384447 -4.54627764 0.25500803
*

A20
C -0.23889865 -1.85681015 0.05859774
C 1.12576490 -1.84863062 0.32254417
C 1.71380975 -0.67974191 0.78498095
C 0.94772136 0.45735674 0.99953188
C -0.41307375 0.43079656 0.72643488
C -1.01943888 -0.72390111 0.25595548
H 2.77785653 -0.67311938 0.98742819
H -1.02450956 1.31032532 0.88867240
C 1.58785638 1.75244887 1.49964361
H 1.00340679 2.19356969 2.30394994
H 2.59491564 1.54077049 1.86737118
H 2.38838352 2.26439951 -0.45502379
C 0.93243716 3.72522989 -0.01719172
C 1.13962005 4.32987388 -1.29371519
C -0.02937292 4.28917367 0.87434809
C 0.41853359 5.43894214 -1.66079706
H 1.87168233 3.89741023 -1.96740505
C -0.74313753 5.39502031 0.49549891
H -0.19287990 3.84784590 1.84922008
C -0.51828949 5.96417650 -0.76706823
H 0.56859229 5.90235469 -2.62772481
H -1.47561530 5.83352503 1.16160535
H -1.08958524 6.84031082 -1.05666836
C 1.67986818 2.60678904 0.29817901
C 2.00700454 -3.07895007 0.13591074
F 3.29339084 -2.70844290 0.00347124
F 1.66955132 -3.76367434 -0.96090909
F 1.92691376 -3.89167361 1.19133986
C -2.51190049 -0.68528310 -0.04672316
F -3.09708774 0.31570961 0.63929401
F -3.12107966 -1.82070646 0.29719733
F -2.73050017 -0.45657862 -1.34657001
N -0.87603328 -3.09641188 -0.44922220
O -1.25241022 -3.06454612 -1.59700432
O -0.95662411 -4.01212362 0.33281995
*

A21
C -0.21384113 -2.61418539 0.00674894
C 1.16351633 -2.49209179 -0.09083484
C 1.80137099 -1.30415081 0.22340439
C 1.03791837 -0.23143124 0.66710253
C -0.34628314 -0.33202551 0.76805539
C -0.95779970 -1.52787283 0.43710638
H 2.87973689 -1.25082574 0.13601914
H -0.96334206 0.48986082 1.11133758
C 1.70596184 1.09939701 1.01146948
H 1.35635694 1.48080581 1.96816646
H 2.78894929 0.95900108 1.05940013
H 1.83644562 1.68348226 -1.07578415
C 0.54242237 3.07466736 -0.15954430
C 0.32849775 3.71937884 -1.41445588
C -0.11090286 3.57906890 1.00451754
C -0.49839217 4.81217561 -1.50059072
H 0.82687472 3.33165057 -2.29650689
C -0.93407123 4.66965748 0.90507462
H 0.04516791 3.10504027 1.96512072
C -1.12503866 5.28055230 -0.34311697
H -0.66638227 5.30626800 -2.44920722
H -1.43504283 5.06369430 1.78045235
H -1.77919402 6.14390952 -0.41009685
C 1.38570695 1.97964833 -0.12947671
N -0.87902465 -3.88025655 -0.34644580
O -0.61248422 -4.83604327 0.34279397
O -1.62891026 -3.83976580 -1.29480653
F -2.27600213 -1.64228637 0.54638003
F 1.87764938 -3.52730316 -0.51124080
*

A22
C 0.29285908 -1.90787238 0.10890335
C 1.51940829 -1.57130575 -0.57308781
C 2.01522147 -0.32154724 -0.49611246
C 1.35447444 0.72860377 0.24533493
C 0.12144914 0.37029800 0.90988059
C -0.38486802 -0.87616582 0.85625186
H 2.04906128 -2.33136446 -1.12916225
H 2.94635120 -0.08292470 -1.00088240
H -0.41212893 1.14538929 1.44875462
H -1.31728472 -1.10204336 1.35334935
C 2.23616252 1.82686559 0.89060340
H 1.88354872 2.17229919 1.85345819
H 3.30311947 1.65941007 0.82458051
H 2.04993744 2.21084281 -1.23863766
C 0.28985908 3.08306984 -0.28148132
C -0.67019745 2.95725431 -1.28510029
C 0.11652617 4.03931389 0.71277952
C -1.79364724 3.76834446 -1.28589731
H -0.53462123 2.21764834 -2.06840132
C -1.01031009 4.85468958 0.71147505
H 0.86894695 4.16875846 1.48286623
C -1.96698652 4.71804502 -0.28361285
H -2.53445853 3.66510162 -2.07044654
H -1.13357254 5.60261579 1.48624747
H -2.84363533 5.35541213 -0.28553352
C 1.49044761 2.19631568 -0.30948078
N -0.19795625 -3.12488832 0.04606120
C -1.40351352 -3.57827572 0.77380423
C 0.38366086 -4.22052073 -0.76007486
H -1.35859899 -3.25349807 1.81304465
H -2.29093863 -3.14605696 0.30281920
C -1.36506723 -5.09661339 0.62208331
H 0.61212803 -3.86658944 -1.76515269
H 1.30623540 -4.56528331 -0.28460296
C -0.69929808 -5.29589515 -0.73976319
H -0.75181247 -5.53462148 1.41326232
H -2.36074676 -5.53415010 0.68189612
H -1.41689815 -5.11731017 -1.54419042
H -0.28127348 -6.29297032 -0.87205447
*

A23
C 0.02247589 -2.43681840 -0.10440747
C 1.38970976 -2.15822909 -0.46954047
C 1.96384408 -0.96934789 -0.19046226
C 1.24789355 0.10298402 0.44750093
C -0.13914132 -0.14924914 0.75485138
C -0.70500874 -1.33688906 0.49679480
H 1.98142463 -2.93728674 -0.92542390
H 3.00880588 -0.81367247 -0.43910530
H -0.75571129 0.64434167 1.16172004
C 2.04652711 1.06740528 1.36396442
H 1.50132409 1.39371444 2.23952471
H 3.07556064 0.78393680 1.54139680
H 2.42333100 1.57817387 -0.71180687
C 0.57156063 2.58614482 -0.13558255
C -0.08499073 2.64760627 -1.36463481
C 0.20826487 3.46585144 0.87832121
C -1.10039395 3.56746624 -1.57067919
H 0.20221805 1.96918077 -2.16223632
C -0.80975556 4.39032199 0.67079840
H 0.73095501 3.45258663 1.82821159
C -1.46700706 4.43926361 -0.54980590

H	-1.60545163	3.60949577	-2.52878597
H	-1.08152922	5.07775188	1.46344507
H	-2.25908905	5.16116423	-0.71145641
C	1.65729348	1.58160148	0.05577419
N	-0.50901839	-3.62331357	-0.33098142
C	-1.77651786	-4.10105922	0.25325909
C	0.19707911	-4.61870564	-1.15001669
H	-1.69477296	-5.18137704	0.35805828
H	-1.93777948	-3.66956973	1.23477319
H	-2.61718278	-3.86334639	-0.39944318
H	-0.53540987	-5.33960942	-1.50570281
H	0.65767035	-4.14744947	-2.01612358
H	0.95422661	-5.14183381	-0.56080664
F	-2.01969083	-1.49284713	0.73349266

*

A24

C	-0.54015388	-2.16829420	-0.23612192
C	0.83305498	-2.25174065	-0.01850866
C	1.52603178	-1.19385730	0.54073442
C	0.84012547	-0.03421267	0.90101975
C	-0.52372333	0.06129047	0.67103992
C	-1.21062303	-1.00982909	0.11095880
H	2.59293515	-1.27902861	0.71041274
H	-1.06716184	0.96377158	0.93140782
C	1.58773951	1.15652633	1.50884046
H	1.03775599	1.58638970	2.34254448
H	2.57154716	0.83162541	1.85344201
H	2.42927784	1.72544136	-0.40901426
C	1.03899163	3.23291381	0.09192420
C	1.27327270	3.88042665	-1.15576456
C	0.10991703	3.80290777	1.01029854
C	0.60696133	5.03957682	-1.47157246
H	1.98153920	3.44244349	-1.85092345
C	-0.54977372	4.95899428	0.68342398
H	-0.07211500	3.32795486	1.96589098
C	-0.30090808	5.57123114	-0.55319282
H	0.77862749	5.53627225	-2.41817288
H	-1.25767887	5.40285002	1.37217173
H	-0.82870606	6.48618044	-0.80199386
C	1.73415353	2.06442162	0.35784846
C	1.54748091	-3.53341046	-0.39280850
F	2.87979037	-3.40853014	-0.28523000
F	1.26767381	-3.88246475	-1.65839878
F	1.16382227	-4.54416339	0.40111836
H	-1.07521499	-3.00035476	-0.67701616
C	-2.69983554	-0.86398349	-0.10921523
F	-2.96046131	0.21444053	-0.87707045
F	-3.33903070	-0.67709347	1.05894757
F	-3.23093280	-1.93283453	-0.70742522

*

A3

C	-1.70378427	-2.23894052	0.26671551
C	-0.33563519	-2.49262460	0.24791156
C	0.56547019	-1.52110754	0.64777974
C	0.09285643	-0.28375571	1.08049742
C	-1.27265972	-0.01819625	1.07588585
C	-2.17091350	-1.00143909	0.68335192
H	1.62792600	-1.73535831	0.64219167
H	-1.64124531	0.95155373	1.39480484
H	-3.23542910	-0.80162096	0.70013754
C	1.08251890	0.80551021	1.51499283
H	0.68345731	1.40907717	2.32591450
H	2.02356121	0.34708674	1.82250545
H	1.80758301	0.99296850	-0.51738552
C	0.69714603	2.74943209	-0.12789101
C	0.88721449	3.18240239	-1.46910647
C	-0.02091937	3.57885752	0.77770689
C	0.37848921	4.38947097	-1.88771422
H	1.43612705	2.54652150	-2.15545147
C	-0.52330069	4.78146480	0.34965682
H	-0.16300495	3.26775703	1.80477469
C	-0.32389874	5.18148284	-0.97836137
H	0.51926993	4.72460462	-2.90759663
H	-1.06734116	5.42395763	1.03072273
H	-0.72441947	6.13503186	-1.30650738
C	1.24544295	1.52775913	0.24656096
C	0.14490406	-3.85281084	-0.20785859
F	1.48584715	-3.92279890	-0.24571325
F	-0.31333788	-4.13353330	-1.43976683
F	-0.29289676	-4.81905554	0.61407563
H	-2.39888923	-3.00952131	-0.04616118

*

A4

C	0.21209490	-2.58557587	0.22181767
C	1.32147739	-2.36219399	-0.58547977
C	1.93190282	-1.11849722	-0.58541925
C	1.44046705	-0.10378168	0.23254562
C	0.31764751	-0.32861437	1.02354614
C	-0.29147595	-1.57441050	1.02851445
H	1.71243544	-3.16176683	-1.20274932
H	2.80730380	-0.94826932	-1.20316583
H	-0.07717350	0.46248121	1.65264759
H	-1.15187885	-1.76030498	1.65895489
C	2.10328139	1.27944597	0.23332616
H	2.14001967	1.70653423	1.23223499
H	3.11364861	1.20133059	-0.17178699
H	1.31534572	1.69615838	-1.74114301
C	0.25844710	2.95406716	-0.41151936
C	-0.55531763	3.42224457	-1.48056738
C	0.06204301	3.47946401	0.89641532
C	-1.52445967	4.37013215	-1.25166092
H	-0.40124709	3.01870476	-2.47560365
C	-0.90723202	4.42550892	1.11307330
H	0.68055580	3.14055980	1.71763829
C	-1.69616216	4.86574694	0.04185816
H	-2.14773932	4.73001816	-2.06053086
H	-1.06367117	4.83614880	2.10278120
H	-2.45970103	5.61492307	0.22394776
C	1.23066126	2.00454131	-0.70027756
C	-0.47614209	-3.93290719	0.17917375
F	-1.15469554	-4.17925592	1.30951777
F	0.40513384	-4.92794856	0.00018849
F	-1.35437778	-3.98246346	-0.83998649

*

A5

C	-0.78208209	-3.07685302	0.01606371
C	0.35942935	-3.08104598	-0.81686146
C	1.21814795	-2.02891788	-0.75913856
C	0.98236938	-0.92352626	0.11616711
C	-0.18521932	-0.95035044	0.93644352
C	-1.04729427	-2.00167407	0.89582097
H	0.52018625	-3.92431991	-1.47628574
H	2.10395887	-2.01932044	-1.38632384
H	-0.39071007	-0.10402120	1.58208535
H	-1.93598625	-2.01880565	1.51728003
C	2.20957561	-0.07239244	0.58600401
H	2.11290411	0.28873588	1.60117607
H	3.16354020	-0.52446907	0.34828630
H	1.79581981	0.41543500	-1.47583132
C	0.50768480	1.68845774	-0.26170317
C	-0.45449583	1.94733981	-1.23811912
C	0.58719011	2.50893465	0.86067908
C	-1.33894789	3.00234830	-1.08412159
H	-0.50990148	1.31785787	-2.12087199
C	-0.29818479	3.56880059	1.01182973
H	1.35434655	2.34706527	1.60955536
C	-1.26374264	3.81246765	0.04456439
H	-2.08371138	3.19924487	-1.84630734

H	-0.22444214	4.21119253	1.88148987
H	-1.95138033	4.64180365	0.16285819
C	1.43680109	0.54824902	-0.46167579
O	-1.58344641	-4.12054699	-0.08107115
H	-2.34034898	-4.05376100	0.51774891

*

A6

C	-0.21124780	-2.82022453	-0.12674880
C	1.02687696	-2.64452354	-0.80236754
C	1.71723099	-1.49034420	-0.63930913
C	1.21902750	-0.43737287	0.19682481
C	-0.03300049	-0.64250219	0.85337990
C	-0.73473026	-1.79890398	0.70720464
H	1.38572466	-3.44952327	-1.43095775
H	2.66740835	-1.35021297	-1.14488922
H	-0.43490046	0.15808331	1.46450380
H	-1.68139262	-1.92859325	1.21169278
C	2.25410555	0.53131969	0.85881695
H	1.97289241	0.84570557	1.85489161
H	3.28113165	0.20835368	0.75101436
H	2.07076711	1.03718831	-1.23382959
C	0.47724908	2.10004722	-0.18852722
C	-0.39290847	2.25832222	-1.26721040
C	0.33103835	2.90722112	0.93589387
C	-1.40752262	3.20010632	-1.21384762
H	-0.27314580	1.63964942	-2.15127163
C	-0.68558680	3.85335889	0.98727530
H	1.02250211	2.82466581	1.76688033
C	-1.55733114	3.99698858	-0.08305413
H	-2.07913184	3.31922412	-2.05603129
H	-0.78757366	4.48616707	1.86115252
H	-2.34732702	4.73789937	-0.04310672
C	1.55535870	1.07999268	-0.28099371
O	-0.80726808	-3.96478994	-0.34218877
C	-2.06890733	-4.25960438	0.29595432
H	-1.95110209	-4.24897637	1.38027574
H	-2.82661200	-3.54372242	-0.02533733
H	-2.32987202	-5.25810245	-0.04252042

*

A7

C	-0.23858122	-2.87116583	-0.11813418
C	0.95793870	-2.67496672	-0.83401525
C	1.70693861	-1.54325236	-0.64685583
C	1.28504705	-0.54703252	0.26583221
C	0.06955117	-0.74001518	0.96940245
C	-0.66608799	-1.87679266	0.78722630
H	1.28659696	-3.43411898	-1.53381778
H	2.63425509	-1.40548086	-1.19389838
H	-0.28314049	0.03493750	1.64002221
H	-1.59099905	-2.00139272	1.33388056
C	2.31000752	0.52155614	0.75547563
H	2.10936350	0.86154235	1.76304634
H	3.33673758	0.23150734	0.57109302
H	1.94908842	0.94840202	-1.32572032
C	0.43391216	2.02545743	-0.20758631
C	-0.37344509	2.25206533	-1.32448063
C	0.19073627	2.72903885	0.97367535
C	-1.42164977	3.15480784	-1.25646844
H	-0.17834213	1.71511312	-2.24728591
C	-0.85587658	3.63549480	1.03760183
H	0.83028960	2.59615569	1.83872237
C	-1.66526532	3.84421825	-0.07363509
H	-2.04528493	3.32685058	-2.12561592
H	-1.03393758	4.18970494	1.95153958
H	-2.48113168	4.55583398	-0.02092026
C	1.54221837	1.06820490	-0.32870403
C	-0.99537919	-4.10204147	-0.35563304
C	-2.09743715	-4.46417012	0.29918802
H	-0.59496823	-4.75689008	-1.12328024
H	-2.59241240	-5.39949861	0.06674103
H	-2.54364779	-3.86356346	1.08325379

*

A8

C	-0.28755397	-3.17026714	0.19065386
C	0.80723833	-3.03848663	-0.67023975
C	1.57100044	-1.88594846	-0.65221099
C	1.26392811	-0.85684208	0.23902760
C	0.15587573	-0.97369770	1.07753410
C	-0.60568651	-2.12776649	1.06689095
H	1.05382849	-3.84907369	-1.34466152
H	2.42735978	-1.79760973	-1.31295333
H	-0.10490303	-0.16714405	1.75480034
H	-1.45338075	-2.23283288	1.73245638
C	2.11201441	0.42379868	0.26183527
H	2.18130816	0.84823194	1.25943546
H	3.10582039	0.22278654	-0.13685147
H	1.37512819	0.88383917	-1.71944611
C	0.34421042	2.17769479	-0.39408453
C	-0.45412738	2.66132173	-1.46215791
C	0.17809131	2.72719009	0.90382495
C	-1.38570303	3.65051513	-1.24124042
H	-0.32266440	2.23930341	-2.45274176
C	-0.75277019	3.71482511	1.11387056
H	0.79017410	2.37847905	1.72587418
C	-1.53200359	4.17118604	0.04408683
H	-1.99812821	4.02213978	-2.05315543
H	-0.88356220	4.14489941	2.09910026
H	-2.26437731	4.95199436	0.21989368
C	1.28921859	1.18782189	-0.67807549
C	-1.08032505	-4.36527617	0.17176098
C	-1.74110096	-5.36469790	0.15537300
H	-2.32804425	-6.25575203	0.14194211

*

A9

C	-0.20844606	-3.31663649	0.25978054
C	0.90271906	-3.19025777	-0.57348658
C	1.61169853	-2.00077998	-0.59364412
C	1.22254860	-0.94320199	0.22489897
C	0.10178567	-1.06852786	1.04208224
C	-0.60908614	-2.25646376	1.07161154
H	1.20713911	-4.02368371	-1.19450709
H	2.48385053	-1.90693961	-1.23183745
H	-0.21276440	-0.24278869	1.67176633
H	-1.47196804	-2.36866559	1.71630005
C	1.99456925	0.38076940	0.19994915
H	2.08767772	0.80761695	1.19541724
H	2.98756863	0.21722027	-0.22295902
H	1.21109619	0.86280585	-1.76285879
C	0.30474431	2.22015296	-0.42202885
C	-0.47630036	2.76368040	-1.48111433
C	0.17739999	2.76339865	0.88814871
C	-1.34682500	3.79977262	-1.24074520
H	-0.37532425	2.34663970	-2.47740523
C	-0.69427224	3.79707108	1.11611457
H	0.77221981	2.36800563	1.70158512
C	-1.45233915	4.30994326	0.05447153
H	-1.94417059	4.21729726	-2.04143986
H	-0.79771148	4.22143123	2.10699059
H	-2.13847383	5.12857180	0.24569368
C	1.17517087	1.18182948	-0.72229083
C	-0.95240801	-4.55107694	0.27918233
N	-1.55041432	-5.53198273	0.29201177

*

B1

C	-0.33988926	-3.64057811	0.39830280
C	0.90700901	-3.15082538	0.81796828
C	1.35326224	-1.93279458	0.37558627

C 0.56277099 -1.14837322 -0.51157411
C -0.70203406 -1.65623536 -0.92863550
C -1.13412000 -2.87205465 -0.47954602
H 1.51172377 -3.74272372 1.49453820
H 2.31545484 -1.54979041 0.69878830
H -1.32018126 -1.08130617 -1.60634920
H -2.09479903 -3.26089035 -0.79712788
C 1.06705240 0.06628096 -0.94366370
H 2.05576850 0.34104655 -0.58017305
H 1.09208098 1.48879276 -2.50688396
H -0.50480020 0.74573620 -2.25318940
C 0.10214856 2.16828494 -0.71301278
C -0.99956588 2.02562794 0.12691785
C 0.95016981 3.26355606 -0.56845616
C -1.27531644 2.99499135 1.08058728
H -1.65353218 1.16471818 0.02753789
C 0.67519687 4.22873462 0.38919954
H 1.81065038 3.37581368 -1.22026649
C -0.43713630 4.09548498 1.21266164
H -2.14361350 2.89163243 1.72068798
H 1.32537111 5.09000829 0.48826252
H -0.65177387 4.85230695 1.95814141
C 0.40105117 1.09159445 -1.76116186
C -0.82487461 -4.97849938 0.85288084
H -1.88402681 -4.93900285 1.11476676
H -0.72221650 -5.69315632 0.02918278
H -0.25322118 -5.35109725 1.70179648
*

B10
C -0.81394454 -3.55803422 -0.35693896
C -0.06316473 -3.23285780 0.78167208
C 0.62864926 -2.04032844 0.75967525
C 0.58184162 -1.17742688 -0.36853221
C -0.18818812 -1.54724293 -1.50075642
C -0.87874228 -2.73429429 -1.48418250
H 1.22312385 -1.74235872 1.61761063
H -0.22545677 -0.90717948 -2.37262647
H -1.46942363 -3.03894665 -2.33928419
C 1.32337944 0.00123637 -0.32134841
H 1.91617660 0.16750645 0.57636245
H 2.33737569 1.51081923 -1.41087224
H 0.89923916 0.86955709 -2.25025957
C 0.46845143 2.10773690 -0.49939394
C -0.92023914 2.00732026 -0.54759341
C 1.07079120 3.08553002 0.29064607
C -1.70447054 2.91262637 0.15170688
H -1.39127183 1.23222380 -1.14366470
C 0.28392058 3.98512016 0.99352626
H 2.15261596 3.16123823 0.33325889
C -1.10250154 3.89981445 0.92306728
H -2.78467272 2.84722059 0.09668441
H 0.75204506 4.75829744 1.59121975
H -1.71540831 4.60555095 1.47161871
C 1.33227293 1.10806271 -1.28256826
H -1.36639520 -4.49330752 -0.36345910
C -0.02738988 -4.16096287 1.96555288
H 0.59560684 -3.75918913 2.76463475
H -1.03341169 -4.31747987 2.36079921
H 0.37219286 -5.13548422 1.67653688
*

B11
C -0.82367007 -3.52483984 -0.22123191
C -0.01116115 -3.14299771 0.85002097
C 0.69044292 -1.95296312 0.76426868
C 0.57724438 -1.14419977 -0.38848576
C -0.24884840 -1.54918869 -1.46434129
C -0.93767457 -2.73565437 -1.36671920
H 1.32248725 -1.64947584 1.59119353
H -0.32925353 -0.94521519 -2.35790342
H -1.57044935 -3.07043821 -2.17938857
C 1.33393819 0.03627714 -0.42849955
H 1.99531819 0.21848882 0.41612631
H 2.25322765 1.54627174 -1.61015428
H 0.77413484 0.85722553 -2.34512925
C 0.43446878 2.07357664 -0.55348122
C -0.95429237 1.95399911 -0.53170597
C 1.06733816 3.03663080 0.23345007
C -1.71078630 2.83276084 0.22799650
H -1.44512426 1.18507698 -1.11918623
C 0.30776897 3.90853110 0.99695741
H 2.14915655 3.12293417 0.22369237
C -1.07991826 3.80774598 0.99259490
H -2.79151199 2.75560179 0.22736409
H 0.79651071 4.67137915 1.59123108
H -1.67184667 4.49137812 1.59005675
C 1.27348307 1.11203651 -1.41499017
H -1.37534576 -4.45897688 -0.16412887
O 0.13251510 -3.87296260 1.97492567
H -0.38741215 -4.68388088 1.93616253
*

B12
C -0.10339053 -2.99879209 -0.28856563
C 0.70424183 -2.52956663 0.77273971
C 1.19054772 -1.25598420 0.75502622
C 0.90049745 -0.36933818 -0.32876015
C 0.08314761 -0.85536361 -1.39543758
C -0.39907765 -2.12940187 -1.36800995
H 0.93781973 -3.18213820 1.60424965
H 1.80837667 -0.89525380 1.57057844
H -0.15229435 -0.21018404 -2.23222497
H -1.01665401 -2.49660626 -2.17897599
C 1.42312932 0.90354842 -0.29967159
H 2.03850945 1.16038894 0.56093787
H 2.13691567 2.49700581 -1.48341771
H 0.70733253 1.70039553 -2.16959903
C 0.30610681 2.97963841 -0.45429069
C -1.05069178 2.70554440 -0.30319811
C 0.85625009 4.10716914 0.14812763
C -1.85950838 3.57088866 0.41958676
H -1.48192952 1.82070740 -0.76175865
C 0.04679003 4.96953740 0.87448638
H 1.91327039 4.32450197 0.03251259
C -1.31034878 4.70258722 1.01007690
H -2.91815979 3.36266802 0.52186587
H 0.47514525 5.85515772 1.32914632
H -1.94129329 5.37898930 1.57485902
C 1.18755425 2.01234933 -1.24456771
C -0.64196864 -4.35177265 -0.30900749
C 0.16758698 -5.50190607 0.28686784
C -1.03806159 -5.03352185 0.99914674
H -1.24585008 -4.59097836 -1.17461557
H 1.14287235 -5.25742137 0.68832493
H 0.12015354 -6.43362512 -0.26145833
H -1.93670286 -5.63468064 0.95373025
H -0.92024994 -4.45615601 1.90736358
*

B13
C -0.81339660 -3.19993723 -0.49645536
C 0.13823070 -2.88606208 0.48045406
C 0.81869409 -1.69235029 0.40205328
C 0.55484897 -0.78763414 -0.65635690
C -0.41133354 -1.13178408 -1.63528727
C -1.08579482 -2.32920487 -1.55146854
H 1.55983218 -1.43418575 1.15327671
H -0.61267084 -0.45815058 -2.45866147
H -1.81997876 -2.59825711 -2.30065541
C 1.28770722 0.40527744 -0.70715520
H 2.05571667 0.53523165 0.05274057

H 2.02744808 2.02237375 -1.87449317
H 0.47281032 1.36183646 -2.46463674
C 0.35677866 2.42164071 -0.54905419
C -1.01827613 2.27908424 -0.36692378
C 1.07808972 3.32242436 0.23591108
C -1.67871316 3.07602199 0.55481584
H -1.57629378 1.55934152 -0.95692379
C 0.41495865 4.11241974 1.16129481
H 2.14975832 3.42710314 0.09976583
C -0.96196086 3.99032238 1.31924497
H -2.75090835 2.98322635 0.67993681
H 0.96987802 4.82874633 1.75533106
H -1.47839019 4.61002962 2.04307357
C 1.08710257 1.55259157 -1.58941890
H -1.33383753 -4.14882328 -0.41148385
C 0.40099053 -3.85474236 1.59598642
O -0.18543504 -4.89685686 1.69134434
H 1.17187951 -3.54585854 2.32746045
*

B14
C -0.17090399 -2.61599883 0.19091151
C 0.38025095 -1.92600818 1.25651743
C 0.93010026 -0.67864172 1.01490859
C 0.91465134 -0.13759350 -0.27277771
C 0.36484219 -0.86626041 -1.32984643
C -0.18348230 -2.11722864 -1.10014501
H 0.37516313 -2.36784125 2.24372000
H 1.37043854 -0.11885167 1.83296662
H 0.38259948 -0.47941653 -2.34157217
H -0.60822790 -2.70933025 -1.89950471
C 1.52503232 1.18399049 -0.47886347
H 2.30219210 1.45348885 0.22651742
H 2.18045303 2.70164222 -1.88297584
H 0.82460855 1.64101745 -2.48627786
C 0.44221468 2.57885245 -0.51292690
C -0.93304684 2.25761704 -0.52706098
C 0.90837739 3.63682808 0.29939944
C -1.81552420 2.99541795 0.22561247
H -1.28268525 1.42232895 -1.12256349
C 0.02073788 4.36292470 1.06053248
H 1.96691793 3.87622645 0.31002835
C -1.33676216 4.04168104 1.01883714
H -2.87290735 2.76230315 0.21298305
H 0.37054522 5.17682768 1.68316054
H -2.03537849 4.61221777 1.62143194
C 1.38154309 2.01364589 -1.63640830
N -0.76701900 -3.95525232 0.44111019
O -0.73188875 -4.36620994 1.57969270
O -1.24475138 -4.52886250 -0.51274303
*

B15
C -0.47871818 -2.97234556 0.00649377
C -0.33766097 -2.42410457 1.28440758
C 0.28450949 -1.21323766 1.45024017
C 0.78098924 -0.52175132 0.31988437
C 0.64070030 -1.07919187 -0.97376791
C 0.01741517 -2.28861642 -1.11397124
H 0.38377297 -0.80023443 2.44728659
H 1.02290024 -0.58609546 -1.85752724
C 1.42841487 0.70481333 0.53618791
H 1.55375174 1.00923887 1.57271506
H 2.78739445 2.18472306 -0.16976242
H 1.93773805 1.30402787 -1.47699203
C 0.65793886 2.60989076 -0.26384603
C -0.51538469 2.38834758 -0.98550719
C 0.72630839 3.65313620 0.66193459
C -1.59300730 3.24454985 -0.82797015
H -0.57988730 1.56110510 -1.68463590
C -0.35679301 4.50205690 0.82189178
H 1.63601852 3.81844130 1.23040555
C -1.51401133 4.29866815 0.07654149
H -2.49555802 3.09018307 -1.40709200
H -0.29795964 5.32617270 1.52271958
H -2.35990320 4.96440273 0.20280238
C 1.86894500 1.67681777 -0.45920182
F -0.81650048 -3.10124870 2.31638370
F -1.06832457 -4.12791828 -0.14810049
F -0.13367209 -2.85212134 -2.30288101
*

B16
C 0.15378533 -3.44727912 0.51160995
C 1.30790483 -2.85236794 1.04477374
C 1.64060215 -1.59158102 0.64554473
C 0.84101420 -0.87347271 -0.29880870
C -0.33627849 -1.50173837 -0.82940549
C -0.66928780 -2.75492380 -0.43490511
H 1.89901315 -3.41355801 1.75713454
H 2.52670181 -1.11119676 1.04498430
H -0.95578087 -0.97676220 -1.54648790
C 1.24222500 0.38926245 -0.65460233
H 2.15214969 0.76140769 -0.18816441
H 1.27547815 1.77312188 -2.24298124
H -0.27039693 0.92188629 -2.09385491
C 0.06980181 2.46330179 -0.59808260
C -1.03018828 2.23331501 0.22432904
C 0.73950110 3.68092672 -0.53178843
C -1.47445650 3.22394421 1.08844412
H -1.55031086 1.28069658 0.18401438
C 0.29607931 4.67038664 0.33531046
H 1.59681170 3.86545284 -1.17113547
C -0.81004638 4.44312127 1.14497597
H -2.33872352 3.04545722 1.71759595
H 0.81185720 5.62278702 0.37383027
H -1.15602514 5.21785589 1.81924564
C 0.55475021 1.35848796 -1.53377580
O -0.17235085 -4.65953579 0.88881019
H -0.98681559 -4.94467522 0.43991862
O -1.73880312 -3.48524148 -0.83814939
H -2.27240620 -3.02125886 -1.49295869
*

B17
C -0.02788626 -2.87312031 -0.52577615
C -0.01672164 -2.43858577 0.84941241
C 0.39756492 -1.17304892 1.12883274
C 0.81751499 -0.27671405 0.08397022
C 0.78935605 -0.73769419 -1.26616916
C 0.37778611 -2.00261273 -1.56310765
H 0.41852333 -0.81269795 2.14891519
H 1.09908711 -0.08002063 -2.06739768
H 0.36223203 -2.34419343 -2.58815044
C 1.23266316 0.98594135 0.41447130
H 1.21941446 1.24581832 1.47163513
H 2.58385468 2.50405670 -0.13089270
H 1.75734607 1.78458198 -1.51896259
C 0.54532113 3.15381516 -0.34767481
C -0.69433053 2.94610322 -0.94685312
C 0.76657462 4.30839315 0.39574955
C -1.69770033 3.89676148 -0.82357707
H -0.87569198 2.04246406 -1.52154009
C -0.23894594 5.25778260 0.52191265
H 1.73151064 4.47675538 0.86314685
C -1.47064265 5.05323266 -0.08729604
H -2.65667885 3.73603680 -1.30244181
H -0.05729365 6.16181980 1.09148217
H -2.25329396 5.79670161 0.00926197
C 1.63351392 2.09353516 -0.48265228
O -0.43208336 -4.09742011 -0.71337645
C -0.48332526 -4.65241195 -2.04513285

H	-1.17989168	-4.08218157	-2.66120143
H	0.51511259	-4.66211424	-2.48433744
H	-0.84473134	-5.66786003	-1.91197546
O	-0.42991714	-3.36254254	1.72515097
C	-0.44484002	-2.99588880	3.10637368
H	0.56324617	-2.74615977	3.44933649
H	-1.12309807	-2.15440507	3.27394132
H	-0.80726096	-3.87124947	3.63886439

*

B18

C	0.08522652	-3.04514493	0.00163017
C	1.10929515	-2.46840553	0.83330352
C	1.47763123	-1.18079035	0.66343934
C	0.87879455	-0.33534424	-0.33915755
C	-0.14856280	-0.92613790	-1.15947903
C	-0.53001661	-2.21107371	-1.00213253
H	1.58581653	-3.06556083	1.59624834
H	2.24980196	-0.75624048	1.29621735
H	-0.62946070	-0.32680204	-1.92201885
H	-1.30382995	-2.61786554	-1.63602387
C	1.29849740	0.95234162	-0.46403950
H	2.07545761	1.28572363	0.22155650
H	1.64062410	2.43391486	-1.91948342
H	0.09078177	1.61361843	-2.12091279
C	0.13747313	3.08065476	-0.52775591
C	-1.09482167	2.83144116	0.07100784
C	0.77102345	4.29928360	-0.31085462
C	-1.69580588	3.79779548	0.86495784
H	-1.59343192	1.87982406	-0.08974864
C	0.17117341	5.26607384	0.48620722
H	1.73020420	4.50200057	-0.77663836
C	-1.06188915	5.01681940	1.07438692
H	-2.66004470	3.60114201	1.31950697
H	0.66602946	6.21767346	0.64253548
H	-1.53079372	5.77298500	1.69329302
C	0.79047844	2.00336978	-1.38269256
N	-0.28359095	-4.30638060	0.15290127
C	0.33897862	-5.15690178	1.17561625
C	-1.32564178	-4.89827489	-0.69619154
H	-0.11027335	-6.14432990	1.12944463
H	1.41079930	-5.25371275	0.99445279
H	0.17102547	-4.74505546	2.17213927
H	-1.46160167	-5.93653708	-0.40897340
H	-2.27379178	-4.37437148	-0.56373497
H	-1.03100582	-4.86470703	-1.74634036

*

B19

C	0.06729735	-2.20925995	0.06307848
C	0.31187459	-1.42869401	1.18946965
C	0.76364343	-0.12474967	1.01987113
C	0.97857407	0.38977834	-0.25539166
C	0.75615876	-0.41065534	-1.36758045
C	0.28558811	-1.71507607	-1.21585028
H	0.93920197	0.48403285	1.89958649
H	0.95756312	-0.05336783	-2.36980157
C	1.48000100	1.78295572	-0.39254405
H	2.25214204	2.06443227	0.31399896
H	2.08718188	3.34875939	-1.78217153
H	0.83335680	2.18946453	-2.43830221
C	0.34756539	2.99995612	-0.41537633
C	-1.01266815	2.61191210	-0.54534703
C	0.67979207	4.13475640	0.37214271
C	-1.99508517	3.33873632	0.07727577
H	-1.26414202	1.73554022	-1.13209493
C	-0.30856456	4.85081788	1.00039779
H	1.72036990	4.42848493	0.46762797
C	-1.63990801	4.45089087	0.84941836
H	-3.03529218	3.05447684	-0.02066242
H	-0.06382152	5.71581380	1.60386281

H	-2.42013059	5.01667567	1.34819505
C	1.35177069	2.58301138	-1.57368983
N	-0.43362106	-3.58911567	0.23291797
O	0.22701660	-4.31039829	0.94147445
O	-1.45702199	-3.85487845	-0.35354584
C	0.07334470	-1.91093568	2.52520305
N	-0.12487355	-2.21783093	3.61346098
C	0.06927756	-2.51080816	-2.39618031
N	-0.06757425	-3.08907458	-3.37839921

*

B2

C	-0.23810449	-3.16439018	0.17132770
C	0.37755393	-2.63029245	1.30936829
C	0.93572235	-1.38125638	1.22831971
C	0.89226528	-0.63868401	0.01549920
C	0.26001494	-1.21638581	-1.12120988
C	-0.29754344	-2.46299793	-1.04449303
H	0.40318513	-3.20503522	2.22583973
H	1.41669314	-0.94490080	2.09708442
H	0.22349872	-0.67454118	-2.05754525
H	-0.77842873	-2.92067843	-1.89916862
C	1.48675982	0.61659247	-0.01109175
H	1.97529322	0.94310814	0.90509454
H	2.41971694	2.11275154	-1.18640714
H	1.13488781	1.22901354	-2.05012745
C	0.42725817	2.58994805	-0.49279960
C	-0.93386793	2.32860913	-0.63359834
C	0.85270366	3.70744096	0.22306409
C	-1.86654291	3.20652751	-0.10158256
H	-1.26823821	1.44835269	-1.17368439
C	-0.08224963	4.58015769	0.75905527
H	1.91279840	3.91010358	0.33747466
C	-1.44064942	4.33077154	0.59604999
H	-2.92509055	3.01358936	-0.22975886
H	0.24877413	5.45979961	1.29835227
H	-2.16940336	5.01553070	1.01389884
C	1.45423697	1.61564493	-1.08574337
Cl	-0.93479838	-4.71497566	0.25875112

*

B20

C	0.00502609	-1.64703659	-0.00680793
C	0.18177284	-0.93078661	1.17337614
C	0.70623519	0.34836400	1.10452466
C	1.03250598	0.91842184	-0.12230761
C	0.86258969	0.18167239	-1.28730006
C	0.35045605	-1.10718231	-1.23929543
H	0.85469062	0.90526237	2.02198896
H	1.13993995	0.58939964	-2.25040215
C	1.58119533	2.29412897	-0.14551912
H	2.22818656	2.54727568	0.68608075
H	2.40747128	3.89676743	-1.36299492
H	1.24999557	2.78971127	-2.24327346
C	0.47076563	3.56316477	-0.30537771
C	-0.85923312	3.19306770	-0.62810307
C	0.70181548	4.64242443	0.58471117
C	-1.91321754	3.88970350	-0.09196071
H	-1.03478664	2.35267882	-1.28999687
C	-0.35874051	5.32757290	1.12649673
H	1.72179795	4.92185502	0.82888510
C	-1.66002200	4.94981520	0.78509844
H	-2.93226234	3.61799737	-0.33720122
H	-0.19071947	6.15178604	1.80822552
H	-2.49689388	5.48958257	1.21590271
C	1.63192278	3.14512788	-1.29512940
C	-0.19826287	-1.48773916	2.54099045
F	0.47489036	-0.83867092	3.50655175
F	0.09064168	-2.78599210	2.64469397
F	-1.50372966	-1.31732934	2.77601659
C	0.20117983	-1.87756833	-2.54687933

F 0.16021730 -1.01828251 -3.58341232
F -0.92843222 -2.58998215 -2.57017507
F 1.23305001 -2.69870851 -2.74352312
N -0.56442780 -3.01558008 0.05346085
O -1.73379979 -3.08475346 0.34872609
O 0.19430968 -3.91907872 -0.19864337
*

B21
C 0.03012241 -2.38250686 0.08074631
C 0.53916626 -1.61601057 1.11835418
C 0.98636906 -0.32769244 0.89658377
C 0.92420957 0.19733601 -0.39250036
C 0.43442207 -0.56420574 -1.44991126
C -0.01243028 -1.84945071 -1.19647285
H 1.36975059 0.24583454 1.73201644
H 0.41648457 -0.20727907 -2.47145209
C 1.41744727 1.57760174 -0.60937983
H 2.26458308 1.86623235 0.00136061
H 1.83657772 3.14829690 -2.05469362
H 0.50364097 1.99648477 -2.54317142
C 0.27956187 2.82943714 -0.48957170
C -1.07826539 2.43117024 -0.41230684
C 0.72815840 3.94308314 0.26432438
C -1.95444785 3.13576916 0.37547333
H -1.41211894 1.56223361 -0.96773515
C -0.15219525 4.63577983 1.05976826
H 1.76929639 4.24337884 0.20192200
C -1.48871014 4.23082020 1.11026867
H -2.99509915 2.84285611 0.43603943
H 0.18250715 5.48699391 1.63918979
H -2.18252592 4.77705198 1.74076783
C 1.12743039 2.38937456 -1.75110434
N -0.45665246 -3.75034970 0.33319471
O 0.37490271 -4.56123455 0.66359917
O -1.64430239 -3.92406611 0.18563020
F -0.46930036 -2.59304499 -2.19549361
F 0.57482963 -2.12483430 2.34233200
*

B22
C 0.23987206 -2.36189380 -0.01882911
C 1.13708786 -1.76648387 0.93899476
C 1.47650297 -0.46589306 0.83050813
C 0.97670829 0.37460425 -0.23206602
C 0.07882052 -0.23881747 -1.18150327
C -0.27798055 -1.53523063 -1.08356122
H 1.52180124 -2.36195478 1.75442085
H 2.14395639 -0.02209657 1.56160662
H -0.31711907 0.35409557 -1.99616442
H -0.94236269 -1.96887265 -1.81729441
C 1.36193712 1.67589227 -0.28990893
H 2.03473148 2.02577376 0.49090988
H 1.83202081 3.18453231 -1.68124449
H 0.34730147 2.31828758 -2.07846905
C 0.14596967 3.77179853 -0.48743328
C -1.13934458 3.47514960 -0.04107469
C 0.70169588 5.01314994 -0.19862465
C -1.86760733 4.41579619 0.67305112
H -1.57798666 2.50558860 -0.25900991
C -0.02575020 5.95462213 0.51875844
H 1.70160689 5.25289372 -0.54612908
C -1.31012735 5.65744164 0.95490610
H -2.87135622 4.18161908 1.00885742
H 0.41047721 6.92375197 0.73183209
H -1.87828434 6.39364279 1.51163761
C 0.93633866 2.72261469 -1.25616777
N -0.10024350 -3.62825119 0.07308947
C -1.07082517 -4.30186018 -0.81725331
C 0.44080433 -4.56619026 1.08091019
H -1.96689148 -3.69133273 -0.92647106
H -0.61500603 -4.44661410 -1.80085488
C -1.33850837 -5.63150038 -0.11703015
H 1.52490350 -4.47074286 1.13791085
H 0.00987257 -4.33033666 2.05810155
C -0.01106410 -5.93462517 0.57817879
H -2.13579536 -5.51068965 0.62029075
H -1.64110046 -6.40509200 -0.82159572
H 0.71397358 -6.32596476 -0.13948204
H -0.10604308 -6.65266088 1.39167783
*

B23
C -0.17990690 -2.85748295 -0.09990691
C 1.10179802 -2.30372506 0.28051525
C 1.44558093 -1.03287868 0.01416409
C 0.55007908 -0.14287046 -0.67929263
C -0.71475839 -0.68950768 -1.08994015
C -1.05336538 -1.97102666 -0.83090149
H 2.43091914 -0.68949484 0.30889105
H -1.42369457 -0.05848318 -1.60993217
H -2.02453283 -2.33124395 -1.13462479
C 0.93371804 1.13981050 -0.92272795
H 1.91461007 1.43909027 -0.55828644
H 0.76418100 2.64368969 -2.38439182
H -0.77834955 1.87406910 -2.00616393
C -0.09155838 3.29917663 -0.52556774
C -1.04383910 3.08290763 0.46710609
C 0.64458087 4.47893032 -0.52487168
C -1.27023758 4.04472908 1.44133144
H -1.61935371 2.16163072 0.47494930
C 0.42066510 5.44065527 0.45206778
H 1.38609853 4.65588422 -1.29750766
C -0.53621410 5.22487546 1.43526967
H -2.02071179 3.87503744 2.20486784
H 0.99067452 6.36248591 0.44077182
H -0.71290476 5.97740867 2.19496840
C 0.15827255 2.22537590 -1.57540143
N -0.55378672 -4.09267125 0.18043340
C 0.12312957 -4.97371246 1.15070582
C -1.76089883 -4.66888318 -0.42882739
H -0.63023038 -5.64829580 1.55304057
H 0.90667446 -5.55545412 0.66380327
H 0.54403176 -4.39848189 1.96780370
H -1.68200110 -5.75178162 -0.36754144
H -2.65855784 -4.34685645 0.10468215
H -1.83111938 -4.39301944 -1.47924161
F 2.00416066 -3.10981018 0.86416159
*

B24
C -0.20290096 -2.11598020 -0.01356231
C -0.06521058 -1.43267442 1.19281722
C 0.52441903 -0.18529366 1.22817393
C 0.98047373 0.40394211 0.04455720
C 0.85694561 -0.28144088 -1.15841476
C 0.26260582 -1.53661800 -1.18098405
H 0.62729857 0.33595104 2.17278048
H 1.23213016 0.13831610 -2.08426894
C 1.60982654 1.73134594 0.12051225
H 2.08720458 1.96679714 1.06432767
H 2.71097901 3.29535512 -0.90315223
H 1.66843516 2.26434457 -1.98852473
C 0.59984402 3.12058384 -0.24183909
C -0.67609509 2.81084440 -0.76480448
C 0.73471960 4.14802697 0.71961459
C -1.77462122 3.52908472 -0.35756888
H -0.78238977 2.00059104 -1.47690717
C -0.37204362 4.85351077 1.13316806
H 1.71642356 4.37899721 1.12066596
C -1.62059420 4.54346755 0.59179878
H -2.75449282 3.30529993 -0.76042741

H	-0.27627587	5.64250771	1.86850679
H	-2.49319702	5.09770192	0.92063642
C	1.88427345	2.60080724	-0.98267726
H	-0.66468708	-3.09578604	-0.03630399
C	-0.59293719	-2.07968152	2.45652331
F	-0.26566806	-1.37192318	3.54863019
F	-0.10339722	-3.31808930	2.60167073
F	-1.93234693	-2.17308084	2.41439836
C	0.16001815	-2.25799988	-2.50830507
F	-0.34295397	-1.43634076	-3.45125463
F	-0.62811056	-3.33354021	-2.43437606
F	1.37112816	-2.65386643	-2.93080192

*

B3

C	-0.85692603	-2.46911995	-1.16279377
C	-0.07812832	-2.27672176	-0.02055084
C	0.64682266	-1.11764748	0.12503622
C	0.59490775	-0.12280250	-0.87977188
C	-0.19725122	-0.33553703	-2.03196534
C	-0.91426785	-1.50324435	-2.16737265
H	1.25451446	-0.95991716	1.00845942
H	-0.23156067	0.41022380	-2.81609151
H	-1.51735294	-1.67848945	-3.04937770
C	1.37342289	1.03435080	-0.69768630
H	2.01763961	1.05032812	0.17877148
H	2.33766219	2.73013143	-1.55596941
H	0.88454646	2.19173454	-2.45678759
C	0.47956531	3.02468426	-0.46690180
C	-0.91077017	2.93708824	-0.54424917
C	1.08056868	3.77044933	0.54965151
C	-1.69633212	3.64391310	0.35137935
H	-1.37835329	2.33232920	-1.31420585
C	0.29087132	4.46913084	1.44810698
H	2.16236936	3.82803529	0.61623143
C	-1.09556082	4.40712974	1.34750378
H	-2.77620925	3.59781686	0.27725009
H	0.75473135	5.06644569	2.22387803
H	-1.71148763	4.95443137	2.05165241
C	1.35686136	2.26392342	-1.48169208
H	-1.42238912	-3.38916302	-1.26957276
C	-0.05343510	-3.36233367	1.03681145
F	0.72959616	-3.02411513	2.06940075
F	0.40521018	-4.51161359	0.52178241
F	-1.28933913	-3.58869799	1.50544478

*

B4

C	-0.03598319	-2.42483866	0.13338203
C	0.51466424	-1.83863426	1.26934231
C	1.05384120	-0.57322545	1.17385303
C	1.04060142	0.11759223	-0.05819312
C	0.48133772	-0.50371353	-1.19820981
C	-0.05081754	-1.76812570	-1.09949584
H	0.51278068	-2.37097511	2.21185746
H	1.48624313	-0.09767973	2.04737195
H	0.47986850	0.00293592	-2.15478353
H	-0.48052526	-2.25569421	-1.96521772
C	1.62645299	1.39916960	-0.10520208
H	2.12029988	1.73890944	0.80245243
H	2.47735057	2.97114679	-1.27046678
H	1.27226224	1.98081016	-2.15682995
C	0.44845116	3.18447144	-0.51496634
C	-0.88692585	2.83848906	-0.72739636
C	0.77284262	4.24709603	0.33242305
C	-1.89372296	3.59308757	-0.14855127
H	-1.13871461	1.99234049	-1.35790321
C	-0.23748979	4.99383397	0.91529896
H	1.81216595	4.50672539	0.50676065
C	-1.56866718	4.66801898	0.67309085
H	-2.93151513	3.34258923	-0.33276317
H	0.01064105	5.83298700	1.55402883
H	-2.35774151	5.25356873	1.13053899
C	1.57337962	2.37079092	-1.18946830
C	-0.59293995	-3.83825963	0.22540062
F	-1.56623695	-4.03357829	-0.67144574
F	-1.08835764	-4.08346979	1.44318680
F	0.38359951	-4.72389199	-0.01637023

*

B5

C	-0.38166636	-3.68816661	0.14893845
C	0.28370878	-3.18109973	1.28690743
C	0.83756514	-1.94107998	1.22394595
C	0.75908604	-1.14738330	0.03237939
C	0.07631727	-1.69438140	-1.10086464
C	-0.48094157	-2.93347709	-1.04652457
H	0.33311796	-3.79502827	2.17714347
H	1.35149740	-1.53207443	2.08716405
H	0.00340888	-1.11887189	-2.01469869
H	-0.99661669	-3.35247496	-1.90394354
C	1.34311479	0.09616295	0.02481775
H	1.84224792	0.40711345	0.94095373
H	2.31786412	1.52311394	-1.18275188
H	0.94495106	0.74585486	-1.99229079
C	0.40184275	2.21040893	-0.47646897
C	-0.97859142	2.02907040	-0.49311507
C	0.94208451	3.36169138	0.08849038
C	-1.81597835	3.00674111	0.02494104
H	-1.40480449	1.12791067	-0.92406178
C	0.10349045	4.33700584	0.61028733
H	2.01744234	3.50776742	0.10274291
C	-1.27460773	4.16061943	0.57854521
H	-2.89058109	2.86899370	-0.00358775
H	0.52676857	5.23935806	1.03583745
H	-1.92769172	4.92482343	0.98354311
C	1.31275301	1.11985994	-1.03891934
O	-0.90100384	-4.89487660	0.26038331
H	-1.33896535	-5.18017673	-0.55387625

*

B6

C	0.00073948	-3.30729632	0.27819433
C	0.85357881	-2.72447408	1.25293440
C	1.29626832	-1.45537412	1.07726236
C	0.92061112	-0.68778215	-0.07758354
C	0.05895819	-1.30387533	-1.04122592
C	-0.39274381	-2.57640047	-0.87552883
H	1.12565304	-3.31949027	2.11544872
H	1.94653145	-0.99846873	1.81541985
H	-0.24028906	-0.74987941	-1.92192489
H	-1.04077624	-3.02528335	-1.61467914
C	1.39848347	0.58991451	-0.21177712
H	2.04793681	0.95461310	0.58224548
H	2.01665660	2.03400293	-1.61550499
H	0.55369776	1.16618823	-2.10974122
C	0.24962169	2.65015529	-0.55063770
C	-1.08976878	2.39519490	-0.26841359
C	0.82318824	3.85277381	-0.15021229
C	-1.85815836	3.34726651	0.38623539
H	-1.54022375	1.45435193	-0.57092383
C	0.05474018	4.80318119	0.50841539
H	1.86639513	4.05699474	-0.36920382
C	-1.28517670	4.55160807	0.77656933
H	-2.90396503	3.15029895	0.59158555
H	0.50177655	5.74441174	0.80643400
H	-1.88430264	5.29600879	1.28792271
C	1.08568118	1.58667675	-1.25843628
O	-0.37717329	-4.53421962	0.52713184
C	-1.24365198	-5.23413498	-0.39264704
H	-2.19670846	-4.71092607	-0.48011003
H	-0.75642261	-5.33265015	-1.36352701

H -1.39388721 -6.21364851 0.05188119
*

B7
C -0.06736139 -3.33882216 0.29102271
C 0.76286033 -2.74493907 1.26389219
C 1.24738874 -1.47915283 1.08466455
C 0.92264106 -0.73509897 -0.08718388
C 0.08027245 -1.34606109 -1.06503884
C -0.39748666 -2.60875463 -0.87643871
H 1.01324324 -3.30527497 2.15673853
H 1.88496571 -1.02413092 1.83513060
H -0.17967053 -0.80529186 -1.96609923
H -1.03426175 -3.05360600 -1.62897004
C 1.44257974 0.53645392 -0.22694349
H 2.08549511 0.89195387 0.57642261
H 2.09272949 2.01010943 -1.59391680
H 0.66629569 1.10986125 -2.15432644
C 0.27774777 2.53990499 -0.55528693
C -1.07185410 2.25740542 -0.36007336
C 0.82031783 3.71872748 -0.05087391
C -1.88331988 3.16601234 0.30400727
H -1.49557625 1.33229028 -0.73887977
C 0.00811088 4.62390968 0.61722196
H 1.87205416 3.94120064 -0.20000927
C -1.34291976 4.34868537 0.79424278
H -2.93689999 2.95139667 0.43949577
H 0.42886950 5.54881745 0.99396814
H -1.97597190 5.05843528 1.31392318
C 1.16500994 1.52626294 -1.28210510
C -0.55332671 -4.69456092 0.53445784
C -1.30911156 -5.40175674 -0.30650414
H -0.25471641 -5.13732470 1.47935504
H -1.62427338 -6.40592452 -0.04877198
H -1.63795441 -5.02508858 -1.26790527
*

B8
C -0.18408575 -3.34700615 0.12421077
C 0.42471797 -2.80088869 1.26906151
C 0.96230081 -1.54249162 1.20960794
C 0.91115135 -0.78482054 0.00589010
C 0.29318299 -1.35765618 -1.14162393
C -0.24285639 -2.61372029 -1.08110754
H 0.45927984 -3.38279058 2.18093587
H 1.43261932 -1.10796881 2.08509411
H 0.25158005 -0.80263842 -2.06995856
H -0.71206394 -3.06267361 -1.94709548
C 1.48287499 0.48019277 -0.00075652
H 1.95735984 0.80500953 0.92345742
H 2.40482989 1.99748328 -1.15518984
H 1.13708501 1.10750624 -2.03555938
C 0.40282925 2.45508169 -0.48054889
C -0.95422122 2.17288923 -0.61929661
C 0.81113250 3.58431908 0.22628895
C -1.90005293 3.04029795 -0.09292598
H -1.27534048 1.28349459 -1.15243251
C -0.13675746 4.44683217 0.75639416
H 1.86806382 3.80366027 0.33901531
C -1.49128476 4.17594184 0.59618614
H -2.95558451 2.82999080 -0.21888205
H 0.18130165 5.33530760 1.28904014
H -2.23012681 4.85265316 1.00942899
C 1.44283096 1.49108834 -1.06547174
C -0.74498819 -4.65424426 0.17783397
C -1.21756784 -5.75559864 0.22204767
H -1.63797146 -6.73712591 0.26208428
*

B9
C -0.28978832 -3.18597158 0.20925627
C 0.31457990 -2.60531434 1.32748369
C 0.90988909 -1.36976102 1.19703258
C 0.90434789 -0.69910436 -0.04582222
C 0.29292096 -1.30978776 -1.16445335
C -0.29810509 -2.54379093 -1.03780590
H 0.30995226 -3.12745353 2.27575626
H 1.38355233 -0.90262745 2.05346332
H 0.29827664 -0.81935008 -2.12941239
H -0.76447901 -3.02889006 -1.88594245
C 1.55309452 0.55268854 -0.12651751
H 2.08485236 0.87943780 0.76410565
H 2.45138706 2.06876927 -1.33243826
H 1.18571672 1.11998684 -2.18078424
C 0.44941887 2.36986951 -0.53077369
C -0.90437643 2.08704070 -0.72344206
C 0.83624754 3.41574255 0.31226401
C -1.86532932 2.88869811 -0.13116601
H -1.20496353 1.25393479 -1.34984746
C -0.12891318 4.20948570 0.90892290
H 1.88918896 3.62546418 0.47154836
C -1.47734357 3.94703992 0.68535426
H -2.91645544 2.68842290 -0.30053473
H 0.16757437 5.03576221 1.54376368
H -2.23122146 4.56928544 1.15364485
C 1.52560337 1.50745503 -1.22544863
C -0.91518986 -4.47633913 0.33602056
N -1.41746468 -5.50485077 0.43550529
*

C0
N 0.15945808 1.05541705 -1.47912925
O 1.43329686 0.91216751 -1.25100117
C -0.50732238 2.08384136 -1.07846558
C -0.47569427 -0.05342427 -2.19424396
H -1.57293378 2.09609411 -1.31696198
C 0.15172922 3.16440523 -0.30305891
H 0.07628046 -0.21578363 -3.12970851
H -0.40137212 -0.94773015 -1.56080117
H -1.52759768 0.17820315 -2.40040065
H 1.01785831 3.56305604 -0.85637813
H -0.55643974 3.97600760 -0.09058796
H 0.53626796 2.75472743 0.64660037
C -0.25293552 0.20651163 1.62259043
C -1.39984163 -0.14021619 1.32448734
O 0.91157794 0.53691548 1.90925105
Li 2.07770829 0.20133670 0.41376555
O 2.16029823 -1.73890209 0.23973371
O 3.91200392 0.86302469 0.56029287
C 1.74978216 -2.61122889 1.27530410
H 0.76532655 -3.05763423 1.04590947
H 2.48749281 -3.42139760 1.42052240
H 1.67038629 -2.01881539 2.19632519
C 2.31701902 -2.37284059 -1.01340284
H 1.37746117 -2.86203489 -1.32934054
H 2.58372495 -1.60198962 -1.74898163
H 3.11629537 -3.13538843 -0.97151397
C 4.91966519 0.18158428 -0.16269223
H 5.92296089 0.46342484 0.20465837
H 4.77080328 -0.89592366 -0.00904202
H 4.85721908 0.40994176 -1.24248170
C 4.01695897 2.27268013 0.47480922
H 3.94413860 2.61279074 -0.57338394
H 3.19107749 2.70617562 1.05567280
H 4.97630461 2.61808026 0.90074734
C -2.71420558 -0.40373180 0.80935523
C -3.69032755 0.61651980 0.73799285
C -3.07490796 -1.68171058 0.32534833
C -4.95654174 0.37058322 0.20661594
H -3.43841734 1.61477903 1.10804560

C	-4.34200948	-1.92293969	-0.20577374
H	-2.33933846	-2.49040193	0.36943097
C	-5.29285636	-0.89991824	-0.27002031
H	-5.68990794	1.18237900	0.16444877
H	-4.58995252	-2.92355401	-0.57437505
H	-6.28608263	-1.09113175	-0.68687866

*

C1

N	-1.65398067	-0.88948271	-1.27452320
O	-0.39308214	-0.77096874	-1.58494049
C	-2.46859305	0.11026413	-1.27940631
C	-2.07421965	-2.23864598	-0.89091405
H	-3.50212775	-0.10083533	-0.99792227
C	-1.99831160	1.47478925	-1.62927655
H	-3.14931141	-2.25556577	-0.67450345
H	-1.83496118	-2.91856839	-1.71966903
H	-1.49894956	-2.52132934	0.00107889
H	-1.51540873	1.47465320	-2.62031982
H	-2.83546400	2.18472585	-1.62429225
H	-1.24049879	1.80520143	-0.90024525
C	-1.36892644	0.35783093	1.82646695
C	-2.27774413	1.13177434	2.13473423
H	-3.07669742	1.78974594	2.42416708
O	-0.44540134	-0.41759485	1.51046988
Li	0.62437266	-0.25043206	-0.03764007
O	2.21073293	-1.38438063	0.10072847
O	1.24527640	1.58716737	-0.13499996
C	3.05941502	-1.08259960	1.19217818
H	2.62865587	-1.44577239	2.14268948
H	4.05616899	-1.53854625	1.05175380
H	3.16709180	0.01025435	1.23902791
C	1.96609571	-2.76999880	-0.05631787
H	1.48419422	-3.19185605	0.84466889
H	1.29564357	-2.89036513	-0.91811274
H	2.90862192	-3.31494827	-0.24594118
C	1.67426710	2.14160642	-1.36196261
H	2.72810409	2.46927977	-1.29841003
H	1.58317120	1.36263589	-2.13167122
H	1.04889063	3.00712508	-1.64733199
C	1.26887253	2.49362553	0.95015621
H	0.58796328	3.34492192	0.76793254
H	0.93364910	1.95342544	1.84531388
H	2.29039302	2.88248987	1.11515683

*

C14

N	1.32691861	1.28489542	-1.50880307
O	2.54703474	0.85370436	-1.36144314
C	0.93557697	2.43063277	-1.06310983
C	0.41918239	0.36489340	-2.19971233
H	-0.11139307	2.68747219	-1.23815665
C	1.86805739	3.32051299	-0.32677162
H	0.84961287	0.13669533	-3.18459892
H	0.35746451	-0.55454134	-1.60310060
H	-0.57381534	0.81868887	-2.30541989
H	2.77912363	3.50014455	-0.92067612
H	1.38492902	4.27715864	-0.08853191
H	2.18501331	2.82793488	0.60848743
C	0.65351269	0.37881768	1.52785879
C	-0.55778943	0.23618325	1.32704110
O	1.86706516	0.50560534	1.71633799
Li	2.96967200	-0.08394392	0.25229318
O	2.53011229	-1.96080017	-0.00553382
O	4.89978286	0.09269201	0.47076452
C	1.93706943	-2.76913506	0.99399093
H	0.88580307	-2.99661401	0.74180468
H	2.49294294	-3.71588150	1.11248197
H	1.96696922	-2.20980201	1.93792233
C	2.59200213	-2.57382198	-1.27767661
H	1.58343324	-2.85697716	-1.62879319
H	3.02534027	-1.84795586	-1.97956079
H	3.22328493	-3.47965192	-1.24734768
C	5.72955464	-0.78039314	-0.27292741
H	6.75859744	-0.77854952	0.12891082
H	5.31287341	-1.79324225	-0.18652585
H	5.76056869	-0.48658407	-1.33816396
C	5.35790361	1.43326473	0.47538111
H	5.40949391	1.83819798	-0.55085294
H	4.64744355	2.02872217	1.06547189
H	6.35758547	1.50044643	0.94109205
C	-1.92282858	0.06977565	0.97357254
C	-2.93162181	0.94529035	1.46344885
C	-2.32485492	-0.97600617	0.10058894
C	-4.26072482	0.79104498	1.10011903
H	-2.64894845	1.75794694	2.13883945
C	-3.64994212	-1.13944657	-0.26766101
H	-1.56813586	-1.66706312	-0.28637537
C	-4.61303565	-0.25179572	0.23382310
H	-3.94626097	-1.94799371	-0.93973582
H	-5.02749860	1.47192972	1.48139398
N	-6.00745357	-0.41795724	-0.15495845
O	-6.29023002	-1.33339068	-0.91552304
O	-6.83509221	0.36523451	0.29403834

*

C15

N	1.30552781	1.21089406	-1.69546017
O	2.55693756	0.94739527	-1.44708062
C	0.76586282	2.34760715	-1.41149537
C	0.54387302	0.11339104	-2.29661533
H	-0.29196378	2.45948862	-1.65894594
C	1.55461358	3.42075669	-0.75699675
H	1.04695498	-0.18065840	-3.22746587
H	0.55483962	-0.72608741	-1.58898636
H	-0.48729990	0.42975797	-2.49472156
H	2.43386362	3.68186780	-1.36884582
H	0.93756725	4.31514389	-0.60038752
H	1.93138660	3.05724590	0.21375289
C	0.38233032	0.51683964	1.34619382
C	-0.79692535	0.21572429	1.13946766
O	1.56859377	0.81392885	1.55339951
Li	2.86824289	0.19458334	0.29864632
O	2.65440535	-1.73324329	0.13882087
O	4.71477349	0.62597237	0.75838558
C	1.99063683	-2.52620884	1.10501698
H	1.00839708	-2.86525657	0.72899031
H	2.59880495	-3.41002681	1.37037840
H	1.83979114	-1.90750923	1.99967697
C	2.95813341	-2.42416491	-1.05697765
H	2.04327045	-2.84138353	-1.51617161
H	3.41240589	-1.70618803	-1.75327623
H	3.66633772	-3.25068639	-0.86410586
C	5.70116008	0.00715638	-0.04679673
H	6.70699314	0.15277093	0.38617172
H	5.48014957	-1.06860956	-0.07993693
H	5.68990107	0.41520777	-1.07377712
C	4.92580427	2.01574660	0.93503551
H	4.91853014	2.54478417	-0.03444948
H	4.11168189	2.39821154	1.56599653
H	5.89095177	2.20163292	1.43952674
C	-2.14255874	-0.15098245	0.82239005
C	-3.24051648	0.47636739	1.45381753
C	-2.41378902	-1.15209333	-0.13847721
C	-4.53712788	0.10946301	1.12755954
H	-3.08760547	1.25492967	2.20379170
C	-3.72241679	-1.49507351	-0.44147715
H	-1.60426162	-1.66973900	-0.65652557
C	-4.80428692	-0.87617432	0.17994875
F	-3.97647968	-2.44407594	-1.35340248
F	-6.06261374	-1.21835892	-0.12403285
F	-5.57786910	0.70503311	1.72558975

*

C18
N 1.36859104 1.26271648 -1.49372229
O 2.61645200 0.92162790 -1.34163417
C 0.87589506 2.34193046 -0.98791419
C 0.54837053 0.31336366 -2.24876206
H -0.18488012 2.52439344 -1.17219042
C 1.71520631 3.25028072 -0.16761644
H 1.02276038 0.15302618 -3.22623604
H 0.52765585 -0.63090205 -1.68763362
H -0.46960281 0.70340182 -2.36953438
H 2.62301165 3.54295454 -0.71991608
H 1.15054702 4.14682074 0.12019005
H 2.04618766 2.71685900 0.74028445
C 0.75095663 0.22255755 1.53472548
C -0.44988854 0.06022028 1.30482792
O 1.97095929 0.37325249 1.75543759
Li 3.08393969 -0.03741673 0.25994337
O 2.81978788 -1.93574343 -0.11704184
O 5.00233540 0.26809487 0.49259192
C 2.25704781 -2.82897469 0.82461196
H 1.23350144 -3.11983261 0.52656067
H 2.87675895 -3.73929492 0.92013156
H 2.21681667 -2.31010051 1.79142722
C 2.94542829 -2.47696601 -1.41597109
H 1.96439877 -2.80706907 -1.80390915
H 3.34191626 -1.68903920 -2.07052107
H 3.63491200 -3.34117776 -1.41834649
C 5.89826003 -0.48506810 -0.30221743
H 6.92596835 -0.42045435 0.09864563
H 5.56910051 -1.53284917 -0.27647148
H 5.89953223 -0.12692236 -1.34808496
C 5.34423091 1.63976317 0.57609565
H 5.35766838 2.10694938 -0.42458896
H 4.58723465 2.13647713 1.19863207
H 6.33612032 1.76595365 1.04658445
C -1.84123090 -0.03489275 0.94018215
C -2.64514967 1.11018400 0.76464813
C -2.47801939 -1.27396946 0.72592375
C -3.98637908 1.03076597 0.39443713
H -2.20348688 2.09750616 0.93206615
C -3.81786938 -1.36994689 0.35576400
H -1.90223000 -2.19509579 0.85896761
C -4.61227601 -0.21593870 0.16526016
H -4.24458888 -2.36367071 0.21203173
H -4.54774479 1.95961507 0.28279212
N -5.94089719 -0.30360927 -0.22799638
C -6.76930399 0.88327605 -0.18299619
C -6.59506120 -1.59545613 -0.21374692
H -7.77488340 0.63439651 -0.54796401
H -6.86742002 1.30309277 0.83819983
H -6.37033445 1.67756788 -0.83548816
H -7.63077070 -1.47868414 -0.56017560
H -6.09861096 -2.30628153 -0.89512338
H -6.61934557 -2.05537352 0.79436803
*

C19
N 1.94394213 0.95316460 -1.76439682
O 3.14533830 0.54150959 -1.47787856
C 1.57319908 2.17882858 -1.60651245
C 1.03555591 -0.07911963 -2.27013039
H 0.54117991 2.41274412 -1.87659494
C 2.50878744 3.19460160 -1.06233005
H 1.48967430 -0.52156281 -3.16695766
H 0.93366100 -0.84726023 -1.49202837
H 0.05765218 0.35715846 -2.50735831
H 3.44736827 3.20248701 -1.63993374
H 2.05151919 4.19243504 -1.08189110
H 2.77193660 2.93506384 -0.02282270
C 1.15781176 0.72242744 1.37567498
C -0.05133925 0.53182370 1.18578625
O 2.35751916 0.91384425 1.55956254
Li 3.53158434 0.00299779 0.31291393
O 3.08866375 -1.87895232 0.49935188
O 5.45036542 0.21649533 0.56068690
C 2.54580357 -2.44857674 1.67544734
H 1.49988856 -2.76542678 1.51242342
H 3.14035684 -3.32247155 1.99776422
H 2.57194874 -1.68229163 2.46169400
C 3.13919891 -2.76958859 -0.59801995
H 2.12946196 -3.14188855 -0.85032140
H 3.54491006 -2.21885535 -1.45748891
H 3.78928206 -3.63472780 -0.37351780
C 6.28065997 -0.82160676 0.07186851
H 7.29929253 -0.73652051 0.49069639
H 5.84155036 -1.77779201 0.38746027
H 6.34259489 -0.79311786 -1.03133692
C 5.93893857 1.51249351 0.26136492
H 6.02439259 1.66158582 -0.82954747
H 5.22889766 2.24261097 0.67390488
H 6.92844575 1.67151418 0.72607516
C -1.40243556 0.28456016 0.86141559
C -2.42810933 1.20342867 1.19099626
C -1.79053598 -0.89442505 0.17905515
C -3.76165359 0.96109153 0.86579704
H -2.17119803 2.12896375 1.70904545
C -3.11717719 -1.13732208 -0.17117514
H -1.03350337 -1.63646625 -0.07918336
C -4.11627943 -0.21097732 0.17508267
N -5.50812302 -0.46399236 -0.18035706
O -5.72738686 -1.11837293 -1.18217175
O -6.37121627 -0.00651515 0.54416235
C -4.71878981 1.98612453 1.22082698
N -5.40808941 2.86406761 1.52275188
C -3.39698731 -2.38739023 -0.84362780
N -3.52630719 -3.41395345 -1.35996825
*

C2
N 1.11299886 1.21197342 -1.60582777
O 2.36840029 0.94630220 -1.38271695
C 0.56252309 2.32158101 -1.24583568
C 0.35984124 0.14819357 -2.27423340
H -0.49863582 2.43548733 -1.47743058
C 1.34106376 3.35939281 -0.52521757
H 0.86336281 -0.08105621 -3.22301517
H 0.37924408 -0.73564750 -1.62276658
H -0.67468022 0.46678535 -2.45118458
H 2.25328250 3.61838013 -1.08696086
H 0.73363990 4.26018647 -0.36737855
H 1.66411831 2.95874145 0.45089534
C 0.39009717 0.41848550 1.46981394
C -0.78179276 0.10624025 1.23910937
O 1.57291410 0.72681202 1.69459104
Li 2.80931843 0.19686385 0.33080964
O 2.64750866 -1.73871085 0.18987634
O 4.69203631 0.63188097 0.62740999
C 2.17903277 -2.54680421 1.25246850
H 1.18855714 -2.97465543 1.01410653
H 2.88664920 -3.37023508 1.45951571
H 2.09087253 -1.90914526 2.14206012
C 2.82594815 -2.43659095 -1.02579441
H 1.87919869 -2.90131065 -1.35720564
H 3.15480566 -1.71163620 -1.78266692
H 3.58908782 -3.22905640 -0.91749719
C 5.64697420 -0.15737858 -0.05713057
H 6.65694074 -0.00063179 0.36262370
H 5.36459718 -1.21112439 0.07185613
H 5.66548348 0.08733318 -1.13493129
C 4.96985348 2.01971393 0.57121760

H 4.99335161 2.37731350 -0.47346098
H 4.17307494 2.54372545 1.11702483
H 5.94025975 2.24206951 1.05052722
C -2.12966713 -0.22373674 0.87917783
C -3.21871396 0.58658520 1.27268049
C -2.42407696 -1.36825422 0.10322125
C -4.52831121 0.27640200 0.91175049
H -3.02673126 1.47900832 1.87450143
C -3.73001045 -1.68718602 -0.26337858
H -1.60585859 -2.01948672 -0.21631734
C -4.77783638 -0.86088532 0.14329517
H -3.93655152 -2.57660509 -0.86414384
H -5.35668580 0.91671332 1.22546380
Cl -6.41598403 -1.25429647 -0.31323377
*

C21
N 1.63909628 1.05049440 -1.54963974
O 2.87484285 0.69213208 -1.34979110
C 1.18952538 2.21119351 -1.21097409
C 0.78402778 0.03272947 -2.16547392
H 0.13482379 2.40310178 -1.41997689
C 2.06929334 3.20350686 -0.54431835
H 1.24907434 -0.27494678 -3.11162043
H 0.73613475 -0.82500011 -1.48109491
H -0.22041881 0.43643567 -2.34124762
H 2.98212909 3.37055661 -1.13899957
H 1.54100120 4.15483555 -0.39915040
H 2.39155796 2.81125880 0.43537863
C 1.01285649 0.39309150 1.55327411
C -0.19646527 0.23753001 1.34195729
O 2.22011708 0.53771018 1.75452551
Li 3.34681219 -0.11204068 0.32412225
O 2.98314293 -2.01671441 0.19717291
O 5.26119694 0.17364673 0.53075089
C 2.39235083 -2.77676916 1.23450190
H 1.35936403 -3.06626914 0.96943041
H 2.98174485 -3.68959512 1.43559286
H 2.37151961 -2.15138001 2.13681264
C 3.10120197 -2.71441431 -1.02710800
H 2.11267913 -3.05682589 -1.38373971
H 3.52984157 -2.02454721 -1.76662944
H 3.76174757 -3.59358291 -0.91638760
C 6.14128593 -0.70376677 -0.14738793
H 7.16650439 -0.61639249 0.25486908
H 5.78060119 -1.72927643 0.01144503
H 6.16186357 -0.48557961 -1.23076412
C 5.64383408 1.53474373 0.43724865
H 5.67710355 1.86548239 -0.61584414
H 4.89835555 2.13056712 0.98194065
H 6.63596146 1.69202870 0.89713631
C -1.55295624 0.11867367 0.95131115
C -2.43257205 1.22827334 1.01723615
C -2.06577964 -1.10921097 0.46230545
C -3.74784179 1.10226544 0.61779542
H -2.08418310 2.19903532 1.37383894
C -3.38038279 -1.20478632 0.05344356
H -1.43517436 -1.99805500 0.40644385
C -4.25834891 -0.10964255 0.12243498
N -5.63866815 -0.22484930 -0.30394110
O -5.87641056 -0.89260017 -1.29479709
O -6.48889284 0.35202045 0.35007337
F -3.83537336 -2.38008444 -0.37935456
F -4.53886435 2.17338657 0.66250969
*

C6
N 1.13967918 1.24213112 -1.65798724
O 2.39224787 0.98479691 -1.40917769
C 0.57615387 2.35082036 -1.31622622
C 0.40431939 0.16817071 -2.32958400
H -0.48114913 2.45677993 -1.56821026
C 1.33446194 3.39786722 -0.58747270
H 0.92898940 -0.07141159 -3.26423617
H 0.41191879 -0.70739394 -1.66680563
H -0.62728454 0.48085833 -2.53221111
H 2.25192259 3.66463026 -1.13700638
H 0.71628110 4.29323394 -0.44079246
H 1.64695259 3.00165728 0.39391970
C 0.36424558 0.50231165 1.42128178
C -0.80337868 0.19435128 1.16891427
O 1.54742728 0.80770102 1.66894432
Li 2.79464461 0.26069226 0.32653101
O 2.64024763 -1.67934563 0.20672897
O 4.67613211 0.69890273 0.64362191
C 2.13484844 -2.46790163 1.26703206
H 1.14600452 -2.88770868 1.00840298
H 2.82720657 -3.29651889 1.50316734
H 2.03071448 -1.81694899 2.14514475
C 2.83971843 -2.39486684 -0.99504834
H 1.89605858 -2.85349167 -1.34339486
H 3.19479488 -1.68383123 -1.75325500
H 3.59102289 -3.19431454 -0.85839459
C 5.64121835 -0.09900513 -0.01565289
H 6.64504892 0.06467931 0.41620578
H 5.35784869 -1.15106346 0.12446629
H 5.67536337 0.13012114 -1.09653198
C 4.95223756 2.08601701 0.56947991
H 4.98667120 2.42787286 -0.48016693
H 4.14845995 2.61664838 1.09833069
H 5.91688389 2.31753734 1.05617110
C -2.14903948 -0.13819109 0.78222105
C -3.24731446 0.66258279 1.14642065
C -2.43133841 -1.28544185 0.00268434
C -4.55635655 0.35155845 0.76239593
H -3.07317697 1.55837437 1.74956218
C -3.72659115 -1.60471415 -0.38592075
H -1.60693884 -1.93750415 -0.30052180
C -4.80385671 -0.78817267 -0.00954102
H -3.92526184 -2.49559911 -0.98845979
H -5.36982183 1.00935809 1.07350504
O -6.04010085 -1.17618811 -0.43440755
C -7.15444087 -0.38397274 -0.06966102
H -7.07142893 0.64059411 -0.47218724
H -8.03892088 -0.86860445 -0.50489388
H -7.27419953 -0.33499230 1.02663946
*

C8
N 0.98511264 1.16751337 -1.44091441
O 2.20954615 0.76686982 -1.25019710
C 0.53505031 2.27859382 -0.96518633
C 0.14091736 0.25085873 -2.21009182
H -0.50961168 2.51187514 -1.18157555
C 1.39925087 3.15853961 -0.13974247
H 0.62931275 0.06654151 -3.17639648
H 0.06927958 -0.68925283 -1.64696384
H -0.85623935 0.68369005 -2.35497468
H 2.32463596 3.41097307 -0.68292924
H 0.86699218 4.07900161 0.13349946
H 1.69940130 2.62183241 0.77652038
C 0.26502486 0.17623266 1.57271948
C -0.93772027 0.07684981 1.31000675
O 1.47689277 0.26800281 1.82498635
Li 2.60642398 -0.21722694 0.34801194
O 2.23184958 -2.09626547 -0.00518952
O 4.52880881 0.00201638 0.61587507
C 1.61688198 -2.95240428 0.93878836
H 0.58324716 -3.19615878 0.63316817
H 2.19087192 -3.89040147 1.04882282
H 1.59256608 -2.42410408 1.90101248
C 2.35545503 -2.66058825 -1.29496123

H	1.36724656	-2.95168520	-1.69567913
H	2.79905699	-1.90056880	-1.95238121
H	3.00513840	-3.55468498	-1.27340507
C	5.40274719	-0.81707141	-0.13776984
H	6.42222965	-0.79594979	0.28766444
H	5.01827274	-1.84532053	-0.09538265
H	5.44805070	-0.48579563	-1.19137365
C	4.94183131	1.35551936	0.67661326
H	4.99919189	1.79828446	-0.33349653
H	4.20063120	1.90579269	1.27247203
H	5.92962379	1.43918619	1.16462636
C	-2.30636998	0.03374800	0.89673481
C	-3.24250991	0.99664086	1.34199043
C	-2.77665701	-0.96354409	0.00954642
C	-4.56744538	0.96831492	0.92216655
H	-2.90989656	1.77930398	2.02923706
C	-4.10084972	-0.99332835	-0.41241401
H	-2.07894507	-1.72501175	-0.34951963
C	-5.01846107	-0.02662435	0.03645239
H	-4.43650958	-1.77491786	-1.09982750
H	-5.26896192	1.72659717	1.28118776
C	-6.39251179	-0.05551168	-0.40257792
C	-7.54579905	-0.07637169	-0.77775605
H	-8.57837036	-0.09792424	-1.08978492

*

C9

N	0.99520484	1.16078193	-1.45390786
O	2.21918708	0.76522478	-1.25128216
C	0.53977963	2.27481066	-0.99002917
C	0.15943580	0.23717749	-2.22408115
H	-0.50378776	2.50454348	-1.21551593
C	1.39785172	3.16336090	-0.16716439
H	0.65617602	0.04829162	-3.18521593
H	0.08568586	-0.69986231	-1.65605560
H	-0.83738362	0.66701501	-2.37988484
H	2.32844577	3.40760616	-0.70515450
H	0.86506510	4.08801383	0.09043698
H	1.68956119	2.63796267	0.75828738
C	0.23642415	0.16868980	1.55002815
C	-0.96743339	0.07676686	1.28466421
O	1.44568034	0.25640304	1.80151133
Li	2.60400928	-0.22667112	0.34239193
O	2.23532719	-2.10513306	-0.01089269
O	4.51964982	0.00222211	0.63519994
C	1.61380380	-2.96357023	0.92678680
H	0.58396883	-3.21024551	0.61118457
H	2.18927167	-3.90001049	1.04174322
H	1.57913826	-2.43632053	1.88928909
C	2.37773088	-2.67062304	-1.29852995
H	1.39610038	-2.96810218	-1.71052898
H	2.82480065	-1.90913122	-1.95193072
H	3.03232873	-3.56076158	-1.26766770
C	5.40641783	-0.81611633	-0.10446731
H	6.41960034	-0.79155699	0.33546405
H	5.02401554	-1.84528239	-0.06565497
H	5.46570970	-0.48653340	-1.15789167
C	4.92821547	1.35710563	0.69907086
H	4.99780283	1.79818086	-0.31096246
H	4.17768134	1.90647144	1.28401981
H	5.90911019	1.44412726	1.20013901
C	-2.33404448	0.05047705	0.87898193
C	-3.26466568	1.00187530	1.36564844
C	-2.81071755	-0.91830426	-0.03920840
C	-4.59087926	0.99181031	0.95654038
H	-2.92440502	1.75905037	2.07653196
C	-4.13574498	-0.93359473	-0.45258925
H	-2.11615322	-1.66736148	-0.42772626
C	-5.03902734	0.02356337	0.04131736
H	-4.48152844	-1.68967975	-1.16257901
H	-5.29041759	1.73724407	1.34394256
C	-6.41346947	0.01213696	-0.38777379
N	-7.51955835	0.00299535	-0.73495432

*

D0

N	0.17654626	1.40700199	-1.67027934
O	0.84172082	1.09305886	-0.44400369
C	-1.25117741	1.53900008	-1.27559009
C	0.41007300	0.28660624	-2.57302795
H	-1.85546778	1.17077095	-2.12158824
C	-1.59506381	3.00483392	-1.01334686
H	1.49176845	0.17505362	-2.73557178
H	-0.00245644	-0.66572093	-2.18948958
H	-0.06294418	0.52365102	-3.53747666
H	-1.46540481	3.61223514	-1.92430159
H	-2.63860679	3.10245262	-0.67441166
H	-0.94207128	3.41143727	-0.22412567
C	-0.08781597	0.46474636	0.43242210
C	-1.35958627	0.64849740	-0.05251088
O	0.47047864	-0.11107265	1.40286749
Li	2.14523770	-0.24371832	0.42376247
O	2.23900950	-1.89207038	-0.52930505
O	3.88583574	0.45519968	0.69603905
C	1.22840486	-2.88037160	-0.40843650
H	0.68732396	-3.00379186	-1.36320232
H	1.66738674	-3.85091487	-0.11590395
H	0.52752567	-2.54464256	0.36753902
C	3.18132647	-2.16018955	-1.55139074
H	2.67771557	-2.27747006	-2.52727173
H	3.87575372	-1.31023806	-1.60473769
H	3.75022883	-3.08049139	-1.32843435
C	5.01487519	-0.33282116	1.03290552
H	5.36583565	-0.09549285	2.05267720
H	4.71460285	-1.38922386	0.99207732
H	5.83844114	-0.16069607	0.31743986
C	4.13360918	1.85235957	0.71549560
H	4.92718815	2.11520253	-0.00611730
H	3.20150129	2.35963093	0.43095183
H	4.43680042	2.18052238	1.72532798
C	-2.59414675	0.13142558	0.51593956
C	-3.83959337	0.36507441	-0.11159653
C	-2.60598376	-0.63461620	1.70722597
C	-5.02723693	-0.14063023	0.41913905
H	-3.87913070	0.95194240	-1.03359042
C	-3.79633976	-1.13639819	2.23033823
H	-1.65877161	-0.82892668	2.21529488
C	-5.01958072	-0.89649446	1.59429641
H	-5.97257897	0.06031306	-0.09571555
H	-3.76861440	-1.72529506	3.15333153
H	-5.95142959	-1.29128122	2.00982138

*

D1

N	-1.95946963	-0.44025999	-0.79956226
O	-0.99997341	0.37583262	-0.12796703
C	-2.99545415	-0.71248730	0.23910236
C	-1.25764614	-1.64565294	-1.22223691
H	-3.36484914	-1.73784890	0.06167285
C	-4.16487656	0.26096305	0.09120989
H	-0.44296580	-1.36619916	-1.90615180
H	-0.83837534	-2.21679552	-0.37196741
H	-1.96771538	-2.28206889	-1.77131192
H	-4.65739800	0.14995103	-0.88961164
H	-4.91433763	0.08064752	0.87930013
H	-3.80988460	1.29976816	0.19143461
C	-1.07967560	0.11029769	1.28396200
C	-2.22504358	-0.57664930	1.53268579
H	-2.56302036	-0.90289555	2.51514992
O	-0.07565798	0.56100904	1.90903788
Li	0.98134017	0.59782673	0.28164151
O	1.98829653	-1.00736720	0.05351805

O	1.78761444	1.99023670	-0.73358162
C	1.96225110	-2.02413591	1.04148502
H	1.44135833	-2.92216906	0.66421465
H	2.98885158	-2.30332613	1.33950112
H	1.42152294	-1.62547351	1.91041437
C	2.60298655	-1.40187337	-1.15828906
H	2.10985412	-2.29847430	-1.57435546
H	2.50605356	-0.57363911	-1.87396701
H	3.67419584	-1.62223989	-1.00126955
C	3.18400292	2.19320430	-0.85949523
H	3.48460093	3.14546866	-0.38766945
H	3.69411895	1.36293076	-0.35130447
H	3.48273758	2.20758128	-1.92285946
C	1.00290651	2.99965996	-1.34841108
H	1.21841873	3.05327757	-2.43033492
H	-0.05296693	2.73463901	-1.20092516
H	1.20329742	3.98378993	-0.88901368

*

D14

N	1.39843670	1.72607067	-1.44787988
O	1.88205822	1.00554700	-0.30757827
C	0.01475076	2.11042281	-1.06237554
C	1.43730410	0.80014332	-2.57356028
H	-0.59514224	2.08966138	-1.98028946
C	0.00006924	3.52073982	-0.47538946
H	2.47421152	0.47177513	-2.73313577
H	0.79093062	-0.08503571	-2.42314964
H	1.10374642	1.34466444	-3.46897856
H	0.33213724	4.26187205	-1.22053028
H	-1.01513443	3.78936600	-0.14338110
H	0.66931376	3.57624440	0.39834105
C	0.78828050	0.44781269	0.38344186
C	-0.38360510	1.02830099	-0.07699816
O	1.11569438	-0.42848996	1.21316718
Li	2.81076821	-0.76401727	0.29326208
O	2.57881940	-2.12401832	-1.01538228
O	4.62711960	-0.53588957	0.76252800
C	1.37407756	-2.86314376	-1.14198939
H	0.89626792	-2.66519722	-2.11758825
H	1.57185200	-3.94596024	-1.05124789
H	0.70101254	-2.54448951	-0.33489383
C	3.52041661	-2.38704515	-2.04077599
H	3.08376996	-2.18176213	-3.03402883
H	4.38584847	-1.72820596	-1.88451360
H	3.85371180	-3.43943544	-2.00527299
C	5.54570341	-1.60426846	0.92498877
H	5.87179942	-1.68039290	1.97710324
H	5.03742051	-2.53526175	0.63726693
H	6.43043015	-1.45809452	0.28062690
C	5.15579269	0.73344059	1.11637138
H	6.03464462	0.97407962	0.49279981
H	4.37115933	1.48233197	0.94151753
H	5.44876948	0.74979857	2.18077354
C	-1.71635331	0.69277607	0.32642137
C	-2.84005994	1.35083860	-0.24806067
C	-1.98010158	-0.31358288	1.30042851
C	-4.13557498	1.02697244	0.11643812
H	-2.68545694	2.13033624	-0.99741238
C	-3.27272033	-0.64080384	1.66685874
H	-1.13924863	-0.83303604	1.76240009
C	-4.35360105	0.02906764	1.07496469
H	-3.45729671	-1.41502112	2.41354680
H	-4.98478136	1.54130282	-0.33656172
N	-5.70711492	-0.31243456	1.45863369
O	-5.87462116	-1.19099858	2.29334307
O	-6.63386854	0.28954484	0.93361658

*

D15

N	1.07231706	1.66958912	-1.55307668
O	1.65176969	1.17881041	-0.33923167
C	-0.36708253	1.84109880	-1.21827594
C	1.29844508	0.64661568	-2.56687290
H	-0.94240733	1.59962116	-2.12777002
C	-0.64975947	3.28700546	-0.81338096
H	2.38041500	0.49236590	-2.68692764
H	0.81685821	-0.31786047	-2.31787606
H	0.89286045	1.01869156	-3.51920214
H	-0.43926322	3.97908294	-1.64519135
H	-1.70445794	3.40660817	-0.51882331
H	-0.02199377	3.56986228	0.04706046
C	0.64829726	0.51830592	0.41194905
C	-0.58593053	0.83181307	-0.10884826
O	1.10999009	-0.19480142	1.33789065
Li	2.84300014	-0.32514617	0.45473039
O	2.90785149	-1.84383741	-0.68696883
O	4.58347336	0.26382209	0.91098872
C	1.84625440	-2.78297351	-0.75013245
H	1.37151644	-2.76784880	-1.74705137
H	2.21717777	-3.80276152	-0.54391161
H	1.10661151	-2.50019612	0.01092887
C	3.90918983	-2.04601332	-1.66792382
H	3.47199743	-2.02950445	-2.68190662
H	4.64184800	-1.23183961	-1.57889372
H	4.42000226	-3.01301957	-1.51258547
C	5.66396162	-0.60345137	1.21158722
H	5.96719800	-0.49647298	2.26793944
H	5.32904773	-1.63542268	1.03623632
H	6.53046985	-0.38671199	0.56215124
C	4.87814093	1.63822792	1.10822456
H	5.71894899	1.95052956	0.46406798
H	3.98147995	2.21306798	0.83880801
H	5.13629875	1.83352715	2.16389820
C	-1.86271461	0.32606933	0.34767188
C	-3.06291983	0.69648546	-0.30219497
C	-1.95402710	-0.56290586	1.44682521
C	-4.27989915	0.19434632	0.13437789
H	-3.06814255	1.37737977	-1.15547873
C	-3.18666933	-1.04394042	1.85275293
H	-1.05835225	-0.87662871	1.98490315
C	-4.37095559	-0.68073289	1.21286603
F	-3.26983618	-1.88636063	2.89415335
F	-5.55650218	-1.15547905	1.62473209
F	-5.41436729	0.54886913	-0.48778862

*

D18

N	1.57438068	1.76415653	-1.49030595
O	2.00590114	1.04793234	-0.33163650
C	0.15254169	2.08846747	-1.19795250
C	1.72367671	0.85908719	-2.62302293
H	-0.39096712	2.05537337	-2.15760525
C	0.03987413	3.49389899	-0.60739568
H	2.78156059	0.57475252	-2.71952221
H	1.10763295	-0.05470375	-2.52515023
H	1.42609501	1.40046567	-3.53325877
H	0.38522788	4.25647655	-1.32508245
H	-1.00435047	3.71702489	-0.33705274
H	0.65094121	3.56930167	0.30664696
C	0.86574617	0.43421727	0.27417412
C	-0.27237690	0.98244744	-0.25155305
O	1.18325137	-0.45742290	1.11086776
Li	2.91779010	-0.69071503	0.27697564
O	2.86157642	-2.05965239	-1.04667547
O	4.70717640	-0.42215376	0.85333289
C	1.68539376	-2.81973391	-1.27202079
H	1.27406473	-2.61345206	-2.27596361
H	1.89873015	-3.90024668	-1.18635481
H	0.95135642	-2.52765734	-0.50902542

C	3.87667205	-2.27682313	-2.00875171
H	3.50296764	-2.06643681	-3.02659067
H	4.70937616	-1.59614487	-1.78342892
H	4.23898215	-3.31968022	-1.96823666
C	5.63819485	-1.46417264	1.08893757
H	5.88229762	-1.53402711	2.16370986
H	5.18014825	-2.40811739	0.76180069
H	6.56775955	-1.29363791	0.51734993
C	5.17060458	0.86131046	1.24251538
H	6.08816320	1.12667889	0.68823132
H	4.37991492	1.58613954	1.00484053
H	5.38048349	0.88800793	2.32644503
C	-1.65314591	0.62720048	0.06975038
C	-2.74496806	1.24417535	-0.57114072
C	-1.97668888	-0.36179235	1.02311423
C	-4.06875701	0.90712300	-0.28375743
H	-2.56486317	2.01323932	-1.32826544
C	-3.29423281	-0.70717006	1.31647655
H	-1.16404934	-0.87546489	1.54285904
C	-4.38558550	-0.07193780	0.68172645
H	-3.46777168	-1.48487574	2.06208701
H	-4.86118402	1.42670080	-0.82486423
N	-5.70589274	-0.39259839	0.99791214
C	-6.76140617	0.07921344	0.12665703
C	-5.96360027	-1.58983060	1.77031467
H	-7.73151155	-0.24803695	0.52557067
H	-6.78153481	1.18033992	0.08063287
H	-6.67022460	-0.29987293	-0.91193490
H	-7.04494246	-1.68215052	1.94133124
H	-5.61383817	-2.51501737	1.26812821
H	-5.47920437	-1.54167783	2.75908105

*

D19

N	2.02327606	2.34161343	-0.20994273
O	2.39478680	1.12878947	0.46249214
C	0.56771797	2.47093463	0.06632127
C	2.32031429	2.13884228	-1.62321993
H	0.10762748	2.92786023	-0.82465352
C	0.33268164	3.36778526	1.27985809
H	3.39506854	1.94006071	-1.73871341
H	1.74224125	1.30709802	-2.06832631
H	2.08028780	3.07203097	-2.15304455
H	0.70617018	4.38716307	1.09198121
H	-0.74235043	3.42626775	1.51240793
H	0.85069503	2.95939231	2.16239408
C	1.27759545	0.29857607	0.57228880
C	0.13044680	1.03432279	0.27257041
O	1.53934096	-0.87678369	0.88809069
Li	3.40303668	-0.71501055	0.26223909
O	3.49312504	-1.18704274	-1.57143701
O	5.07628724	-0.75665881	1.12525439
C	2.41149167	-1.77927807	-2.27459674
H	2.04210407	-1.10096665	-3.06364349
H	2.72468775	-2.73231580	-2.73605267
H	1.60695271	-1.97035315	-1.55198455
C	4.59160680	-0.84448328	-2.39967629
H	4.27479487	-0.15662240	-3.20308752
H	5.34640090	-0.34643303	-1.77535042
H	5.03464097	-1.74838206	-2.85377608
C	6.08250847	-1.71935891	0.85254682
H	6.27039446	-2.34710619	1.74098911
H	5.72826056	-2.35627683	0.03000735
H	7.02252084	-1.22453233	0.55145256
C	5.40178904	0.12707785	2.18839316
H	6.32220574	0.69035777	1.95510409
H	4.56644094	0.83095887	2.30656548
H	5.54488587	-0.43289996	3.12893174
C	-1.19250914	0.52578256	0.22068800
C	-2.29144197	1.36323011	-0.11464292
C	-1.49227027	-0.84085083	0.48594080
C	-3.59256748	0.87904728	-0.17750493
H	-2.12372079	2.41795665	-0.33801121
C	-2.78732489	-1.33355754	0.40772683
H	-0.68413706	-1.51861639	0.76174461
C	-3.86459136	-0.48108933	0.07909557
N	-5.21632935	-0.99081506	0.00746877
O	-5.37131688	-2.19309018	-0.12666423
O	-6.13676623	-0.19423559	0.08372590
C	-4.60717197	1.83547649	-0.56716806
N	-5.32250910	2.67967970	-0.90408078
C	-2.95035867	-2.73597535	0.72859525
N	-2.96096119	-3.85578783	1.01810523

*

D2

N	1.03562486	1.61495017	-1.57417876
O	1.61051897	1.12567961	-0.35894616
C	-0.39455446	1.83253718	-1.22804821
C	1.22167170	0.57106185	-2.57469039
H	-0.98495084	1.59966409	-2.13011103
C	-0.62901000	3.29096961	-0.83669931
H	2.29753715	0.38301096	-2.70220986
H	0.71477879	-0.37523291	-2.30665749
H	0.81821744	0.94008254	-3.52916660
H	-0.40778735	3.96793944	-1.67826549
H	-1.67580465	3.44565545	-0.53064261
H	0.01725735	3.56378232	0.01326418
C	0.59188533	0.50054810	0.40977926
C	-0.63572659	0.84226156	-0.10497749
O	1.04820708	-0.21345307	1.33980504
Li	2.76071422	-0.39810208	0.43383382
O	2.77015572	-1.94565388	-0.67609312
O	4.53032205	0.15798160	0.82490884
C	1.67513345	-2.84689782	-0.71473286
H	1.19439996	-2.83360025	-1.70883620
H	2.01049031	-3.87539690	-0.49133960
H	0.95172956	-2.52283387	0.04537584
C	3.75455562	-2.19941086	-1.66180745
H	3.30888950	-2.18517927	-2.67219463
H	4.51549881	-1.40963951	-1.59300305
H	4.23323114	-3.18075095	-1.49415792
C	5.59319651	-0.72232131	1.14822397
H	5.92218421	-0.56975659	2.19126083
H	5.22723113	-1.75228902	1.03327381
H	6.45122891	-0.56325432	0.47134460
C	4.86835768	1.53200098	0.93629954
H	5.70082340	1.78238181	0.25523028
H	3.98132067	2.11640447	0.65601264
H	5.15914425	1.77889752	1.97253037
C	-1.93110165	0.38263668	0.36144569
C	-3.12451214	0.78802903	-0.27932236
C	-2.06036806	-0.49651522	1.46456660
C	-4.37475172	0.34359800	0.14893770
H	-3.07863574	1.46638915	-1.13524137
C	-3.30554137	-0.94480431	1.89656866
H	-1.15865213	-0.82710461	1.98424558
C	-4.46168632	-0.52324166	1.23713021
H	-3.38230797	-1.62348520	2.75026876
H	-5.28329142	0.67066305	-0.36349545
Cl	-6.02536364	-1.08362875	1.78031047

*

D21

N	1.81135283	2.14921128	-0.83104283
O	2.22080352	1.15147203	0.11466769
C	0.37273334	2.36394133	-0.52013712
C	2.02949102	1.57871507	-2.15518709
H	-0.13677311	2.57946385	-1.47344781
C	0.20500445	3.55119384	0.42594697
H	3.09743683	1.34726439	-2.27454583
H	1.43381450	0.66322709	-2.33189621

H	1.75345522	2.34018628	-2.89905971
H	0.56687282	4.48117750	-0.04164237
H	-0.85530792	3.68348993	0.69300146
H	0.77191300	3.38063551	1.35529139
C	1.10541222	0.38731374	0.49085459
C	-0.05108513	1.03630104	0.07625028
O	1.39393140	-0.67553173	1.07808700
Li	3.20935055	-0.68289389	0.33157596
O	3.23756644	-1.62391224	-1.31448508
O	4.92756279	-0.50749947	1.09374482
C	2.11989164	-2.35563531	-1.79395089
H	1.72901760	-1.90524162	-2.72335863
H	2.39956881	-3.40545556	-1.99213515
H	1.34290346	-2.32380488	-1.01843878
C	4.30202494	-1.53233478	-2.24533547
H	3.95562017	-1.07848284	-3.19049189
H	5.08433133	-0.89763645	-1.80631673
H	4.72171078	-2.53143008	-2.45859145
C	5.91903475	-1.52120997	1.05370914
H	6.15821039	-1.87164415	2.07301407
H	5.52093358	-2.36182022	0.46816278
H	6.84012933	-1.14659016	0.57350898
C	5.31134013	0.64166278	1.83429562
H	6.21454000	1.10107063	1.39563922
H	4.48119662	1.35995879	1.78979677
H	5.51173550	0.37669242	2.88716091
C	-1.38344200	0.55005404	0.22108474
C	-2.48788630	1.29774341	-0.27132026
C	-1.66138813	-0.69787504	0.84665218
C	-3.77390623	0.82241363	-0.13932311
H	-2.35087054	2.25597628	-0.77475353
C	-2.95395050	-1.15631983	0.94717061
H	-0.85638285	-1.30465169	1.26164113
C	-4.05696833	-0.41853573	0.46621057
N	-5.40612683	-0.91025837	0.58664832
O	-5.59116676	-2.11428837	0.50601970
O	-6.30284812	-0.10076539	0.76283203
F	-3.16695695	-2.31967209	1.56490279
F	-4.77163058	1.54672385	-0.64740355

*

D6

N	1.06263881	1.71418086	-1.60673656
O	1.62259087	1.21965943	-0.38750285
C	-0.38051922	1.89048540	-1.29218860
C	1.29568571	0.69261047	-2.62007457
H	-0.94291652	1.65080581	-2.21066364
C	-0.66540077	3.33868142	-0.89549535
H	2.37805215	0.52812608	-2.72319058
H	0.80119583	-0.26791870	-2.38156126
H	0.90930764	1.07004739	-3.57843502
H	-0.44258168	4.02933557	-1.72559296
H	-1.72342288	3.46132709	-0.61430750
H	-0.04807431	3.62166416	-0.02750817
C	0.59536180	0.56400308	0.35316863
C	-0.62340937	0.88446860	-0.18411907
O	1.05099924	-0.15398721	1.28554131
Li	2.75964684	-0.32377109	0.38812353
O	2.78575046	-1.85688500	-0.74366876
O	4.53953185	0.18922738	0.80213912
C	1.68249404	-2.74599077	-0.81252565
H	1.21210240	-2.70784845	-1.81095460
H	2.00535696	-3.78219377	-0.60706028
H	0.95465248	-2.42992328	-0.05323800
C	3.77780579	-2.09865922	-1.72425626
H	3.34548028	-2.04961446	-2.73937225
H	4.55023033	-1.32344964	-1.62394927
H	4.23897562	-3.09181684	-1.57839741
C	5.55707323	-0.72666421	1.16940316
H	5.77649244	-0.65524265	2.24937793
H	5.19876610	-1.74033824	0.94175329
H	6.48124917	-0.53113479	0.59729030
C	4.86689542	1.54556654	1.06266075
H	5.77862356	1.83670667	0.51188807
H	4.02388136	2.16207403	0.72157577
H	5.02712631	1.70772410	2.14337514
C	-1.92910402	0.40016972	0.25091646
C	-3.10915665	0.75535436	-0.42717411
C	-2.07447803	-0.46035140	1.36739777
C	-4.36897571	0.28730772	-0.03080153
H	-3.05720375	1.41768508	-1.29580941
C	-3.31901294	-0.93024803	1.76661795
H	-1.18145594	-0.75761550	1.92191663
C	-4.48188037	-0.56238365	1.07209536
H	-3.41103296	-1.59409136	2.63130736
H	-5.24936224	0.59755665	-0.59663000
O	-5.66168024	-1.07030272	1.53950247
C	-6.84838524	-0.74777132	0.84375959
H	-6.81266810	-1.09978306	-0.20209387
H	-7.66877647	-1.25966305	1.36509891
H	-7.04196445	0.33905811	0.85295337

*

D8

N	1.07926876	2.08175178	-0.80461594
O	1.48494450	1.14304612	0.19649901
C	-0.38491337	2.22331494	-0.58522414
C	1.40306801	1.48384729	-2.09404570
H	-0.84660692	2.37876392	-1.57459864
C	-0.67387141	3.43415435	0.30089724
H	2.48685016	1.30598986	-2.14558877
H	0.86748858	0.53234033	-2.27300561
H	1.13207415	2.20473187	-2.87937905
H	-0.34237723	4.36773112	-0.18271528
H	-1.75289523	3.51211343	0.50772738
H	-0.14974383	3.33270657	1.26501582
C	0.37598765	0.32504665	0.53431698
C	-0.77433639	0.89514004	0.03505884
O	0.70284736	-0.70621700	1.17252404
Li	2.52597592	-0.62041322	0.48488841
O	2.67775907	-1.64024527	-1.11417946
O	4.21983136	-0.37169089	1.29428537
C	1.60101653	-2.42067090	-1.60898396
H	1.23537701	-2.01637594	-2.56947341
H	1.91715217	-3.46865082	-1.75701136
H	0.79365692	-2.38217816	-0.86541867
C	3.77568931	-1.55188208	-2.00415828
H	3.45622621	-1.14667318	-2.98054863
H	4.52007421	-0.87611584	-1.56049375
H	4.23407260	-2.54470420	-2.16031022
C	5.22109776	-1.37325938	1.35872705
H	5.40480014	-1.67392272	2.40525563
H	4.86552250	-2.24459337	0.79106095
H	6.16517195	-1.01041699	0.91518758
C	4.54902049	0.81565015	1.99879582
H	5.46943906	1.26716367	1.58832274
H	3.71380952	1.51874102	1.87582123
H	4.69533689	0.60356893	3.07251793
C	-2.11434639	0.35783651	0.12238295
C	-3.21316524	1.03667641	-0.45900723
C	-2.39074392	-0.86938784	0.77830074
C	-4.50371854	0.52438306	-0.39222940
H	-3.05113959	1.98631925	-0.97539851
C	-3.68014086	-1.37974753	0.84319072
H	-1.56607007	-1.41662948	1.23926550
C	-4.76313816	-0.69391785	0.26010173
H	-3.86146551	-2.32879694	1.35629155
H	-5.33003518	1.07418963	-0.85209938
C	-6.10170839	-1.22473300	0.33281327
C	-7.22847902	-1.67121900	0.39538672
H	-8.23038514	-2.06771373	0.45087901

*

D9
N 1.05814158 2.07616323 -0.82124523
O 1.46805588 1.13872901 0.18060445
C -0.40475576 2.21924475 -0.59478982
C 1.37659276 1.47466961 -2.11046267
H -0.87107220 2.37364554 -1.58189751
C -0.68813804 3.43021327 0.29261009
H 2.46022931 1.29781752 -2.16637338
H 0.84081028 0.52234360 -2.28440693
H 1.10126620 2.19359687 -2.89597880
H -0.35722561 4.36282898 -0.19296490
H -1.76620171 3.50975644 0.50368665
H -0.16019793 3.32846443 1.25457025
C 0.36583344 0.32281182 0.52533469
C -0.79115895 0.89169200 0.02900335
O 0.69144036 -0.70461790 1.16500189
Li 2.52307993 -0.61947313 0.48546608
O 2.67867866 -1.63603499 -1.11376626
O 4.20872510 -0.35929629 1.30405634
C 1.61305849 -2.43143623 -1.60897199
H 1.24069355 -2.03089334 -2.56842306
H 1.94447823 -3.47434881 -1.75888620
H 0.80553820 -2.40643825 -0.86499859
C 3.77636803 -1.53426246 -2.00311469
H 3.45256785 -1.13272358 -2.97954257
H 4.51231952 -0.84982103 -1.55867313
H 4.24641904 -2.52154296 -2.15920188
C 5.22065597 -1.35096889 1.36102039
H 5.41113237 -1.65335509 2.40578103
H 4.87187401 -2.22388495 0.79158510
H 6.15915377 -0.97663373 0.91533799
C 4.52904812 0.82864262 2.01202514
H 5.44220339 1.29173562 1.59837268
H 3.68587061 1.52356415 1.89710075
H 4.68349526 0.61327328 3.08389171
C -2.12217130 0.35070490 0.12311470
C -3.22762848 1.02956354 -0.45314488
C -2.38868549 -0.88124667 0.78046105
C -4.51370128 0.51475282 -0.38207841
H -3.07020949 1.98028613 -0.96800664
C -3.67246775 -1.39738650 0.85191204
H -1.55923479 -1.42485424 1.23614473
C -4.75417734 -0.70685030 0.27181409
H -3.85035819 -2.34740929 1.36345066
H -5.34609302 1.05951266 -0.83589814
C -6.08623475 -1.24207732 0.34842405
N -7.16112125 -1.67418036 0.40998890
*

E0
C -1.35636869 -2.76439278 1.99803170
H -0.28381097 -2.74349907 1.79729303
H -1.62626172 -3.79548808 2.24016122
C -2.09019725 -2.39971717 0.77936344
C -2.85141479 -2.16182652 -0.14431420
H -3.64597382 -2.10748683 -0.86319374
C -2.31438089 0.52871432 2.77713911
H -2.08788294 1.57577457 2.60795666
H -3.36123524 0.25625526 2.86452135
C -1.35946435 -0.39326932 2.89947195
C -1.72312662 -1.83334059 3.16555981
H -1.21077707 -2.20439219 4.05558391
H -2.79521139 -1.90371840 3.35466372
Au -1.09090235 -0.89324169 -0.65510248
P 0.32897855 0.40749761 -1.89232313
C 0.02390199 0.16168050 -3.65748314
C 0.18896925 2.18905401 -1.63415849
C 2.06683170 -0.01180313 -1.63946835
H 2.22478688 -1.05749555 -1.90928992
H 2.69283094 0.62680041 -2.26702836
H 2.33143781 0.13012848 -0.59135634
H 0.14919465 -0.89335596 -3.90619518
H 0.72868043 0.76138306 -4.23820810
H -0.99680042 0.46534131 -3.89643975
H -0.84460299 2.49941500 -1.79612524
H 0.84041427 2.70631440 -2.34251401
H 0.48515434 2.44549401 -0.61760099
C 0.08431224 -0.04903208 2.75492132
C 1.07285425 -0.77009998 3.42914951
C 0.48279460 1.01324122 1.93806747
C 2.41383030 -0.42256106 3.31274209
H 0.80100698 -1.60373195 4.06592293
C 1.82029476 1.36588438 1.82615942
H -0.26454866 1.56370084 1.37910441
C 2.79344515 0.64961266 2.51486262
H 3.16345186 -0.99188289 3.85204390
H 2.10579533 2.19601427 1.18867203
H 3.83969586 0.92110693 2.42412883
*

E1
C -2.82404109 -1.26075564 0.54645727
H -1.95102939 -1.79860463 0.91934932
H -3.63203730 -1.98711854 0.42778014
C -2.52909063 -0.71667804 -0.78534832
C -2.46617207 -0.23701871 -1.90566728
H -2.62277035 0.13279395 -2.90040467
C -2.42695570 2.16505584 1.32733944
H -1.69979640 2.95688620 1.47085585
H -3.37407030 2.43886101 0.87369049
C -2.19277190 0.90904022 1.70795441
C -3.25188065 -0.14965638 1.51979925
H -3.49657868 -0.62491588 2.47250367
H -4.16350201 0.31957402 1.14722226
Au -0.34030655 -0.14234453 -1.23813441
P 1.93305122 0.09088490 -1.36234977
C 2.51114471 -0.27420780 -3.03612975
C 2.54981359 1.74876134 -1.00096534
C 2.83500855 -1.04403446 -0.28599920
H 2.57226848 -2.07041474 -0.54803517
H 3.90930712 -0.89589251 -0.41852421
H 2.56461936 -0.85997703 0.75379738
H 2.22932351 -1.29360394 -3.30483029
H 3.59798026 -0.17135450 -3.07962139
H 2.05057111 0.42009477 -3.74090629
H 2.08013590 2.46216370 -1.68037654
H 3.63316449 1.77130235 -1.14038759
H 2.30960705 2.02253091 0.02577634
C -0.89024954 0.49703175 2.30291310
C -0.80507943 -0.57714464 3.19383716
C 0.28931744 1.17447533 1.98886864
C 0.40697280 -0.94187624 3.76213162
H -1.69568638 -1.13393834 3.46153731
C 1.49840694 0.81366568 2.56826839
H 0.26534685 1.98964228 1.27463927
C 1.58098553 -0.25120506 3.46230232
H 0.44125086 -1.77857944 4.45376793
H 2.39968213 1.36004881 2.30829534
C 2.89731378 -0.66801344 4.06358596
H 2.83710283 -0.70234867 5.15468796
H 3.18095124 -1.66775741 3.72125904
H 3.69367580 0.02495660 3.78542715
*

E10
C -2.86939498 -0.95230265 1.45719194
H -2.18158038 -1.79846989 1.47746833
H -3.84183348 -1.31436310 1.80172557
C -3.03589597 -0.53443261 0.06139870
C -3.29219051 -0.18898862 -1.07952554
H -3.72952799 0.07979747 -2.02117041

C	-1.16684106	1.93902686	1.16880575
H	-0.28717414	2.42845512	0.76838106
H	-2.12579140	2.38537502	0.92761130
C	-1.10399183	0.84315956	1.93251014
C	-2.39384804	0.19210807	2.37760463
H	-2.29656153	-0.21868973	3.38418551
H	-3.17654097	0.95132108	2.40503677
Au	-1.04297630	-0.10464204	-1.04322463
P	1.13730480	-0.01870482	-1.72508258
C	1.32949106	-0.34170604	-3.49303462
C	1.93985881	1.57393201	-1.43962119
C	2.15957061	-1.24855201	-0.88674599
H	1.78978933	-2.24720817	-1.12577884
H	3.19707623	-1.15341342	-1.21587426
H	2.10106090	-1.09620935	0.19124964
H	0.92127198	-1.32561772	-3.72965670
H	2.38844672	-0.31007354	-3.76014554
H	0.78686762	0.41473736	-4.06258579
H	1.39045259	2.35709460	-1.96483374
H	2.96754710	1.53723108	-1.80849910
H	1.94416469	1.79484116	-0.37257237
C	0.19572675	0.20830113	2.29708185
C	0.25629649	-1.10524177	2.76598501
C	1.39674295	0.91538166	2.16918248
C	1.48107143	-1.69229885	3.06630498
H	-0.64607252	-1.68446534	2.91051340
C	2.62857614	0.33693431	2.44872417
H	1.37762389	1.95222528	1.85437339
C	2.66106575	-0.98275615	2.90262572
H	1.50930412	-2.71416033	3.42986754
H	3.61401218	-1.44949614	3.13219134
C	3.90367111	1.10284174	2.21154231
H	4.29480064	0.87857377	1.21318240
H	3.73535013	2.18021111	2.26880019
H	4.67287456	0.82957559	2.93707631

*

E11

C	-2.66788612	-1.08516871	1.25896591
H	-1.79949177	-1.74449514	1.30832758
H	-3.56056666	-1.70698865	1.36459024
C	-2.73072936	-0.45919231	-0.06787683
C	-2.94979035	0.10529918	-1.12734957
H	-3.35362890	0.57036848	-2.00554458
C	-1.54267838	2.15516886	2.00978108
H	-0.69640157	2.83195485	1.97055297
H	-2.52343046	2.57981049	1.82158865
C	-1.40862890	0.86092216	2.29961370
C	-2.62804322	-0.02540554	2.37231498
H	-2.67063598	-0.55064885	3.32848197
H	-3.52302880	0.59454710	2.30418025
Au	-0.72247186	-0.02599101	-1.11028751
P	1.45185473	-0.00323522	-1.82094630
C	1.55007298	-0.31839372	-3.59838239
C	2.32859999	1.55361367	-1.56928506
C	2.44538521	-1.29795753	-1.04733024
H	2.04582722	-2.27065895	-1.34039434
H	3.48321647	-1.21395211	-1.37778782
H	2.39402904	-1.20815470	0.03712614
H	1.08300285	-1.27789618	-3.82597265
H	2.59708180	-0.33737884	-3.90993408
H	1.02394494	0.47112984	-4.13768270
H	1.77524333	2.35935939	-2.05476848
H	3.32641899	1.47967893	-2.00842472
H	2.41870421	1.76545529	-0.50497226
C	-0.07148461	0.24436646	2.54383570
C	0.06319333	-0.89707680	3.33883441
C	1.07442263	0.81199023	1.98425320
C	1.32064401	-1.43593201	3.58212933
H	-0.80572822	-1.36365353	3.78509829
C	2.32825878	0.27371294	2.24334333
H	1.00147484	1.67857895	1.33920973
C	2.46109925	-0.85876590	3.04230080
H	1.41500119	-2.31845239	4.20582828
H	3.44355858	-1.28096368	3.23105415
O	3.40625942	0.88573539	1.66790832
H	4.21125715	0.40986290	1.91563973

*

E12

C	-0.85618406	-1.25118850	2.79806118
H	-0.17114687	-1.69922966	2.07650728
H	-1.28390845	-2.06351536	3.39071153
C	-1.95775859	-0.59912637	2.07681343
C	-2.93412237	-0.02101990	1.62771831
H	-3.87753832	0.45012542	1.43086935
C	-0.04832665	2.17548274	3.32619232
H	0.32205483	3.07674048	2.84976112
H	-0.78452144	2.29888965	4.11398617
C	0.38941452	0.96290077	2.98677441
C	-0.12497482	-0.25626810	3.71461192
H	0.69654919	-0.79610690	4.19196616
H	-0.80429863	0.06554574	4.50518787
Au	-1.56149912	-0.11170602	-0.13335348
P	-0.71246521	-0.03985555	-2.25460203
C	-1.87109123	-0.71216558	-3.46682751
C	-0.31764947	1.62416148	-2.83135447
C	0.79791647	-1.01406680	-2.42026435
H	0.57907022	-2.05891703	-2.19339711
H	1.18208098	-0.93107970	-3.43957558
H	1.54065498	-0.64373565	-1.71518305
H	-2.10480715	-1.74682958	-3.21058249
H	-1.42370456	-0.67377243	-4.46287634
H	-2.79149075	-0.12590392	-3.45715980
H	-1.22365961	2.23262358	-2.82441057
H	0.08192658	1.57458121	-3.84695561
H	0.42047260	2.07588869	-2.16873991
C	1.37762853	0.76661622	1.88979780
C	2.26316148	-0.31431326	1.89337908
C	1.46118542	1.66758718	0.82524510
C	3.21243572	-0.47217230	0.89325710
H	2.22862457	-1.04205437	2.69612659
C	2.42050726	1.51740134	-0.16504328
H	0.76331156	2.49538330	0.76560656
C	3.31468685	0.44653126	-0.15179420
H	3.88014909	-1.32631570	0.93628197
H	2.46729001	2.23602915	-0.97723911
C	4.29278893	0.29471552	-1.26335405
C	5.61765697	-0.39393801	-1.05684966
C	4.55152828	-1.06733091	-1.86392577
H	4.29356113	1.12478963	-1.96056051
H	5.82781724	-0.78213383	-0.06708059
H	6.47386889	0.01430429	-1.58044302
H	4.67847664	-1.12517114	-2.93850670
H	4.03042623	-1.90503622	-1.41429466

*

E13

C	-3.25769703	-0.89749779	-0.61709489
H	-2.72027771	-1.76833259	-0.24004961
H	-4.26136426	-1.23059375	-0.89475505
C	-2.59594442	-0.45490203	-1.84872862
C	-2.15264871	-0.08755401	-2.92357473
H	-1.97325922	0.20895432	-3.93854278
C	-1.62500778	1.93136966	0.19816504
H	-0.66557945	2.39425787	0.39689428
H	-2.24983362	2.40316736	-0.55274882
C	-2.04616237	0.83648512	0.83840015
C	-3.36786032	0.21567925	0.44665505
H	-3.86763734	-0.21409071	1.31684684
H	-4.01423848	0.99917336	0.04894660
Au	-0.32119991	-0.08636309	-1.61544717

P	1.84899953	-0.05197689	-0.90053532
C	3.03283030	-0.36713138	-2.22855120
C	2.34781038	1.52299073	-0.17097978
C	2.17058052	-1.30543440	0.35862743
H	1.98445632	-2.29567253	-0.06064139
H	3.20888296	-1.23722641	0.69171024
H	1.50294023	-1.14741330	1.20635964
H	2.82825480	-1.34193799	-2.67428826
H	4.04892601	-0.35335705	-1.82726014
H	2.93257747	0.40289600	-2.99538358
H	2.20680846	2.32336983	-0.89935021
H	3.39942458	1.47474916	0.12139417
H	1.73629126	1.72748622	0.70818109
C	-1.21902009	0.18392805	1.89273352
C	-1.41144560	-1.15132113	2.25167656
C	-0.22017799	0.89587951	2.56417872
C	-0.61606910	-1.76793281	3.21622820
H	-2.19262778	-1.73547586	1.78299389
C	0.57433077	0.28590677	3.52514533
H	-0.06281182	1.94836488	2.35541748
C	0.38516673	-1.05761126	3.85289864
H	-0.78953112	-2.80905032	3.46460535
H	1.01427134	-1.52044904	4.60447191
C	1.62861410	1.09176038	4.18542627
O	2.41094535	0.65665838	5.00179742
H	1.66797791	2.15339453	3.87628097

*

E14

C	-1.78327614	-1.31687668	2.78128526
H	-1.16890650	-1.89922550	2.09291724
H	-2.59725131	-1.96446970	3.11467740
C	-2.37018647	-0.18366036	2.05118681
C	-2.89175848	0.81968981	1.59342580
H	-3.45627523	1.70631435	1.37872475
C	0.01303204	1.40974552	4.34522933
H	0.71915675	2.20568356	4.13330746
H	-0.68139315	1.56510598	5.16463888
C	0.00805133	0.27235466	3.65336852
C	-0.95568211	-0.84062268	3.98894905
H	-0.41819673	-1.71019356	4.37676946
H	-1.62756632	-0.49432141	4.77531678
Au	-1.63644472	0.09427148	-0.11422020
P	-0.74306598	-0.25082845	-2.19043608
C	-2.03499455	-0.37733165	-3.44689130
C	0.36173488	1.05643389	-2.76107435
C	0.19419545	-1.79076171	-2.29896647
H	-0.46343603	-2.63000487	-2.06613683
H	0.59133679	-1.90574473	-3.31020041
H	1.01582840	-1.77488414	-1.58246136
H	-2.70868531	-1.19815475	-3.19545615
H	-1.58053486	-0.56194917	-4.42311307
H	-2.60409561	0.55329473	-3.47917553
H	-0.16803697	2.01044898	-2.75154894
H	0.69400563	0.83229261	-3.77743679
H	1.22807457	1.12110563	-2.10306562
C	0.94173396	0.06853418	2.51234280
C	1.50980198	-1.18431691	2.26503715
C	1.25127426	1.13054604	1.65728193
C	2.39002556	-1.37135766	1.21193564
H	1.27607695	-2.02389285	2.90868491
C	2.13139340	0.96219175	0.60195185
H	0.78898057	2.09745682	1.81419289
C	2.68939095	-0.28982090	0.39924403
H	2.83721296	-2.33787151	1.02322475
H	2.37599432	1.78498452	-0.05559527
N	3.60841649	-0.48403002	-0.73215833
O	4.14288644	-1.57063676	-0.85649139
O	3.78590309	0.45196941	-1.49182132

*

E15

C	-2.97805460	-1.04632727	-0.01766331
H	-2.17653932	-1.62277512	0.44753451
H	-3.82279727	-1.72264088	-0.17083278
C	-2.54268642	-0.58767683	-1.34310179
C	-2.35319753	-0.16681520	-2.47261942
H	-2.39781813	0.15908656	-3.49373775
C	-2.34533224	2.34928718	0.63548719
H	-1.57293976	3.08726812	0.82129182
H	-3.19340091	2.66345137	0.03630177
C	-2.28871757	1.11377323	1.13089001
C	-3.40710157	0.13606310	0.86745303
H	-3.79460609	-0.27392536	1.80250079
H	-4.22962791	0.66275097	0.38227969
Au	-0.29666569	-0.13805973	-1.60663663
P	1.98362731	-0.03648279	-1.44947154
C	2.75249527	-0.53990965	-3.00607096
C	2.65480303	1.60252724	-1.10207744
C	2.65601483	-1.15656195	-0.20249409
H	2.40057932	-2.18229357	-0.47477587
H	3.74227524	-1.04876954	-0.16152597
H	2.22780312	-0.93511120	0.77442786
H	2.44167551	-1.55586476	-3.25481688
H	3.83984386	-0.50382043	-2.90544267
H	2.43747813	0.13484533	-3.80376511
H	2.34016468	2.29024704	-1.88914639
H	3.74576588	1.54953195	-1.07879300
H	2.28843733	1.96687048	-0.14354503
C	-1.12276047	0.64969568	1.93659488
C	-1.26001015	-0.38763267	2.86034020
C	0.12807798	1.25281133	1.77751380
C	-0.17338494	-0.78599335	3.61323639
H	-2.20268938	-0.89286594	3.02532694
C	1.19115108	0.84087466	2.55065747
H	0.28909255	2.03751887	1.05065644
C	1.06349154	-0.18269185	3.47567619
F	2.40147971	1.41101649	2.40397190
F	2.11281724	-0.58468256	4.20311227
F	-0.29920194	-1.78125042	4.50812929

*

E16

C	-2.85476970	-0.96940604	-0.40725319
H	-2.08687197	-1.64316918	-0.02596154
H	-3.50965623	-1.55851266	-1.05334554
C	-2.21001921	0.06121447	-1.23280920
C	-1.80965684	0.99249369	-1.91106833
H	-1.66604266	1.83292598	-2.56180008
C	-3.53889514	1.94979728	1.63551030
H	-3.06097282	2.75682175	2.18079519
H	-4.53445681	2.13523382	1.24499843
C	-2.94714228	0.76818646	1.46531650
C	-3.66652767	-0.34865278	0.74536281
H	-3.91091014	-1.15663229	1.44057313
H	-4.60815606	0.03993343	0.35512745
Au	0.09464638	0.10365285	-1.12469384
P	2.30059845	-0.44812291	-0.89682683
C	3.00629825	-0.91543231	-2.49412674
C	3.34050501	0.89315263	-0.28371666
C	2.60471594	-1.85832977	0.18942664
H	2.00242840	-2.70771745	-0.13658309
H	3.66417077	-2.12194880	0.14418699
H	2.33970923	-1.59962365	1.21391047
H	2.46847973	-1.77838539	-2.89060686
H	4.06204340	-1.16791032	-2.37009273
H	2.90950279	-0.08388371	-3.19412935
H	3.26754534	1.74527564	-0.96194917
H	4.37826395	0.55555198	-0.23130306
H	3.00279312	1.19950247	0.70644119
C	-1.56573393	0.51023672	1.95497400
C	-1.17980355	-0.74865484	2.41055841

C	-0.61896961	1.54066677	1.96306367
C	0.10561307	-0.97034486	2.89308341
H	-1.88837057	-1.56853888	2.41345811
C	0.65653024	1.32440221	2.44982897
H	-0.87616145	2.52190513	1.57707042
C	1.02766925	0.06190493	2.92156529
H	0.40115707	-1.94802528	3.25771224
O	1.63054415	2.28705862	2.49308219
H	1.27050438	3.12965195	2.18328066
O	2.28867952	-0.16846056	3.38693589
H	2.78154585	0.66477416	3.37659493

*

E17

C	-3.05162592	-0.52785258	-0.35720837
H	-2.35261972	-1.20973813	0.12766700
H	-3.81411564	-1.14212336	-0.84157646
C	-2.33270668	0.21239470	-1.40300304
C	-1.85082764	0.91133730	-2.27851663
H	-1.63059746	1.57129632	-3.09468507
C	-3.35186823	2.82361025	1.04736703
H	-2.78535235	3.68330032	1.38851759
H	-4.35931099	3.00420532	0.68603522
C	-2.85062307	1.58886498	1.08518352
C	-3.70853206	0.41552699	0.66875630
H	-3.97093719	-0.19034264	1.54089915
H	-4.64010768	0.80230651	0.25380908
Au	-0.04618474	-0.05543249	-1.35442531
P	2.07114786	-0.85188292	-1.03943076
C	2.62668299	-1.78647784	-2.48349901
C	3.30016596	0.44693992	-0.79899194
C	2.24672693	-1.98299270	0.35682129
H	1.50958000	-2.78300385	0.27335268
H	3.25206991	-2.41117299	0.34168290
H	2.09733360	-1.44925952	1.29434729
H	1.96641980	-2.64161733	-2.63787538
H	3.64852895	-2.13843970	-2.32311764
H	2.59416882	-1.14807902	-3.36786439
H	3.32532585	1.08680480	-1.68278360
H	4.28476628	0.00120807	-0.63994646
H	3.02383567	1.04913485	0.06608854
C	-1.45637893	1.33006409	1.53721400
C	-1.12623080	0.12719957	2.17972200
C	-0.45423133	2.27010015	1.34062236
C	0.15640251	-0.10729470	2.64615857
H	-1.89257764	-0.61852324	2.34086990
C	0.83664932	2.05336869	1.82049453
H	-0.66777832	3.18702903	0.80417571
C	1.15240794	0.87930288	2.48464252
H	1.59490236	2.80894991	1.66095508
O	2.37768522	0.57742666	2.98961133
C	3.33136557	1.63582854	3.05798293
H	3.63582775	1.97258163	2.06334662
H	2.93244080	2.48204671	3.62519033
H	4.19623874	1.22415699	3.57689949
O	0.55113648	-1.24547707	3.27593367
C	-0.38334114	-2.31926314	3.32665672
H	-0.71274560	-2.60311218	2.32223760
H	0.14537767	-3.15400855	3.78520558
H	-1.25225805	-2.06117043	3.93958111

*

E18

C	-1.67383245	-1.17752667	2.49184115
H	-0.86637541	-1.70264421	1.97995663
H	-2.35324401	-1.93440295	2.89122131
C	-2.42113759	-0.37592412	1.51319806
C	-3.13080291	0.34552810	0.83158088
H	-3.89278371	0.96340818	0.39786260
C	-0.72628168	2.12689157	3.49093020
H	-0.16854297	3.00695832	3.18854777
H	-1.60754666	2.28454201	4.10453219
C	-0.34457920	0.89615802	3.14605411
C	-1.12845186	-0.30014981	3.63217255
H	-0.50410871	-0.93904405	4.26244189
H	-1.96071097	0.04826748	4.24551807
Au	-1.38133956	-0.02818979	-0.51162584
P	-0.04820257	-0.15113713	-2.36464760
C	-0.94808400	-0.79463350	-3.79362188
C	0.61372582	1.44289911	-2.89344910
C	1.36093104	-1.25756842	-2.14362568
H	0.99381077	-2.26283036	-1.92887385
H	1.96425093	-1.27464892	-3.05399397
H	1.96236727	-0.91446430	-1.30420443
H	-1.32673436	-1.79246537	-3.56597631
H	-0.27970689	-0.84494220	-4.65649522
H	-1.78944563	-0.13825457	-4.02188495
H	-0.21602510	2.10762159	-3.14112569
H	1.24558432	1.30561838	-3.77388285
H	1.19518296	1.88680333	-2.08599666
C	0.82996030	0.65305339	2.26942059
C	1.59607993	-0.50978679	2.36877549
C	1.22416598	1.58592769	1.30628368
C	2.71449845	-0.72331032	1.57848977
H	1.32857332	-1.27494875	3.08964212
C	2.34325311	1.39612700	0.51454674
H	0.63210088	2.48303782	1.15918212
C	3.13253960	0.23665029	0.63854848
H	3.26537942	-1.64578013	1.70191813
H	2.59387999	2.15254925	-0.21617308
N	4.25709784	0.04210435	-0.14135718
C	4.47602701	0.91909736	-1.27983074
C	4.81641384	-1.29701661	-0.23256619
H	5.41117072	0.63786869	-1.76219163
H	3.66885348	0.85745685	-2.02221555
H	4.56991676	1.95855236	-0.95805435
H	5.68676290	-1.26840260	-0.88663607
H	5.15038491	-1.64954910	0.74562831
H	4.09939928	-2.02432229	-0.63774125

*

E19

C	-1.05936063	-1.04015040	3.01013427
H	-0.32253788	-1.58707914	2.41818284
H	-1.29929288	-1.65081076	3.88433547
C	-2.30133620	-0.89739953	2.24029234
C	-3.42233132	-0.77832445	1.77398194
H	-4.47385882	-0.74435002	1.56358825
C	-0.73730515	2.19750659	1.85761719
H	-0.41335531	2.81444422	1.02667801
H	-1.65940314	2.48215830	2.35277101
C	-0.03259527	1.15392823	2.29436457
C	-0.51313928	0.32676957	3.45810346
H	0.29017068	0.14714196	4.17408808
H	-1.29748513	0.87126625	3.98456056
Au	-2.15509828	-0.29547180	0.00284049
P	-1.61117217	0.02679198	-2.19892798
C	-3.01321199	-0.39155461	-3.26023606
C	-1.15616632	1.70775374	-2.67837249
C	-0.26543730	-1.05398302	-2.73059715
H	-0.60065949	-2.09046942	-2.65791592
H	0.00766853	-0.82841749	-3.76369011
H	0.59999284	-0.92024395	-2.08308301
H	-3.32015254	-1.42143673	-3.07203497
H	-2.72258669	-0.28106615	-4.30769808
H	-3.84831316	0.27595032	-3.04115111
H	-1.97255495	2.38592986	-2.42371589
H	-0.98038573	1.73625435	-3.75625378
H	-0.25247934	2.02777735	-2.16158679
C	1.22775478	0.74626606	1.61298152
C	2.25880882	0.11056658	2.29976599
C	1.40115558	0.99470008	0.25351915

C 3.44765060 -0.23316892 1.65808418
H 2.16810591 -0.12121493 3.35238734
C 2.57184440 0.64231180 -0.40742700
H 0.61362536 1.46761523 -0.31318742
C 3.59995648 0.03280177 0.30289518
N 4.84233421 -0.34235552 -0.38681182
O 5.88261034 -0.18401419 0.21610326
O 4.73780417 -0.78256440 -1.51319726
C 4.46044885 -0.90016677 2.43631930
N 5.21178784 -1.44556574 3.11168639
C 2.65559666 0.95285297 -1.81192885
N 2.61997300 1.24051574 -2.92300071
*

E2
C -0.52469576 -1.28020489 2.83720967
H 0.08374747 -1.77743786 2.07982727
H -0.86973111 -2.04646769 3.53598190
C -1.71561128 -0.69905678 2.20337894
C -2.76297539 -0.18464549 1.84605318
H -3.75263176 0.21410731 1.73425479
C 0.32829462 2.14518028 2.84868677
H 0.67662228 2.96746286 2.23321765
H -0.38937967 2.37937365 3.62820040
C 0.77129204 0.89908855 2.68462286
C 0.28371359 -0.20805483 3.58665909
H 1.12220037 -0.71046431 4.07384641
H -0.33754656 0.22165016 4.37369966
Au -1.53210205 -0.09764423 -0.01215134
P -0.94032169 0.12980890 -2.20997172
C -2.32729197 -0.27433366 -3.29594275
C -0.43131518 1.79342565 -2.69135737
C 0.39364687 -0.98543306 -2.69597230
H 0.07203305 -2.01608161 -2.53599950
H 0.63069477 -0.83548535 -3.75180102
H 1.27804775 -0.79026842 -2.08965996
H -2.65417262 -1.29773583 -3.10511344
H -2.01590568 -0.17758720 -4.33873262
H -3.15610808 0.40743279 -3.09788252
H -1.24599451 2.49052538 -2.48758477
H -0.19745828 1.80691333 -3.75843054
H 0.44684090 2.09563032 -2.12174781
C 1.73638507 0.55251293 1.60317497
C 2.64387875 -0.49873482 1.75065209
C 1.75970973 1.27987370 0.40962750
C 3.56376917 -0.80270197 0.75549305
H 2.65442937 -1.09010589 2.65815004
C 2.67957012 0.99697207 -0.58812066
H 1.04252922 2.07634542 0.25072483
C 3.57431636 -0.04551993 -0.40374154
H 4.26607259 -1.61738173 0.88448867
H 2.69160766 1.56904455 -1.50773929
Cl 4.71788932 -0.42267187 -1.66754383
*

E20
C -1.06317208 -0.15692037 3.65356829
H -0.53612714 -1.00907233 3.22206303
H -1.14398684 -0.33823536 4.72865527
C -2.42795654 -0.14451812 3.11689727
C -3.60252059 -0.11843089 2.79064542
H -4.67283407 -0.09130262 2.72494582
C -1.16466983 2.40965460 1.47474177
H -1.19979685 2.70327955 0.43243032
H -1.93499416 2.81636238 2.12086282
C -0.23353228 1.59148013 1.97131941
C -0.30855734 1.16964109 3.42012203
H 0.68989398 1.05988338 3.84817072
H -0.81959625 1.95356588 3.97987195
Au -2.55970469 -0.23294102 0.80351116
P -2.13482951 -0.49444775 -1.42798316
C -3.48170391 -1.32586446 -2.29867018
C -1.88048701 1.06220996 -2.30728609
C -0.66264446 -1.49481230 -1.73154086
H -0.82119124 -2.49645461 -1.32786293
H -0.47017242 -1.55820139 -2.80484025
H 0.19349127 -1.04454580 -1.23087443
H -3.64951239 -2.30850358 -1.85485219
H -3.22167378 -1.43947330 -3.35372938
H -4.39422891 -0.73407093 -2.20911247
H -2.77192852 1.68368957 -2.20550256
H -1.69550148 0.85988965 -3.36467850
H -1.02826013 1.59196153 -1.88302927
C 0.83588811 1.02559643 1.10279088
C 1.55762413 -0.10147712 1.48716880
C 1.13997537 1.60493132 -0.12945732
C 2.51945973 -0.66568475 0.66009166
H 1.37731377 -0.56712502 2.44416857
C 2.08424379 1.05215595 -0.97708185
H 0.64113837 2.51231078 -0.43855078
C 2.76759511 -0.08984223 -0.57652551
N 3.75689416 -0.70713861 -1.48736199
O 4.92134149 -0.65633482 -1.15816746
O 3.32341865 -1.22067121 -2.49646155
C 3.25489251 -1.90233023 1.13800275
F 2.57732594 -2.53132801 2.11184685
F 4.46887797 -1.60486688 1.63242162
F 3.43304000 -2.79561150 0.14550530
C 2.30731524 1.69791156 -2.32925729
F 1.93570657 2.98744948 -2.32556686
F 1.58903275 1.08511414 -3.29037763
F 3.59762336 1.66037322 -2.70812331
*

E21
C -1.13587029 -1.21212232 2.85950199
H -0.45807068 -1.71660517 2.16838841
H -1.50498349 -1.96327620 3.56227350
C -2.29702706 -0.69994580 2.11948949
C -3.33310324 -0.24141632 1.66630696
H -4.32745462 0.10700034 1.46392938
C -0.37834526 2.22997080 2.76626875
H 0.00679181 3.03449470 2.14978436
H -1.18241487 2.47427362 3.45232327
C 0.11974625 0.99540356 2.72263918
C -0.42164168 -0.08547987 3.62516967
H 0.37955294 -0.53988800 4.21160482
H -1.12125624 0.36415098 4.33070641
Au -1.96436379 -0.15171207 -0.09322212
P -1.14721451 0.04725900 -2.22046831
C -2.36259893 -0.48473898 -3.44752171
C -0.70046761 1.73015985 -2.69522530
C 0.30278013 -0.99277165 -2.49973823
H 0.00275530 -2.03937049 -2.41955955
H 0.70892937 -0.80348743 -3.49576995
H 1.06244472 -0.78928935 -1.74707423
H -2.64269224 -1.52203995 -3.25759505
H -1.93324528 -0.39880443 -4.44855799
H -3.25181603 0.14394504 -3.37727640
H -1.59245908 2.35795890 -2.65189028
H -0.30495280 1.72846682 -3.71355165
H 0.04810418 2.13040254 -2.01331715
C 1.21724881 0.64475917 1.77751211
C 2.11741300 -0.37764271 2.08085141
C 1.35543656 1.34191600 0.57374290
C 3.14506746 -0.66626975 1.20792487
H 2.04876245 -0.95564817 2.99270253
C 2.38609497 1.02906718 -0.28203106
H 0.66420911 2.12350873 0.28893931
C 3.29917408 0.02371379 0.01407536
N 4.37043520 -0.31641101 -0.92061482
O 5.49699620 -0.39766854 -0.47621843

O 4.05818737 -0.49810690 -2.08119877
F 2.52286489 1.71429745 -1.42484093
F 3.99195596 -1.65932666 1.50220552
*

E22
C -2.72609289 -1.48560121 1.74651734
H -1.78668482 -1.95478667 1.45169800
H -3.47892120 -2.27489956 1.81218524
C -3.14466492 -0.55201209 0.69220364
C -3.61949646 0.26319669 -0.08124269
H -4.21890444 0.94705337 -0.64973821
C -2.24123712 1.64910421 3.42567572
H -1.64999514 2.55883693 3.41940287
H -3.26423087 1.72462727 3.78048259
C -1.73792193 0.47785934 3.03302858
C -2.58542547 -0.77102865 3.10229660
H -2.15365026 -1.49001425 3.80377533
H -3.57638038 -0.50735562 3.47499925
Au -1.53852085 0.00969573 -0.86530577
P 0.25420405 0.10187180 -2.28207856
C -0.21693138 -0.38681595 -3.95746466
C 0.95322962 1.75856967 -2.44736604
C 1.61730847 -0.99387101 -1.83414144
H 1.25671410 -2.02373139 -1.81094578
H 2.41227096 -0.90124331 -2.57769217
H 1.99758462 -0.72789875 -0.85008380
H -0.58925634 -1.41257035 -3.94518355
H 0.65074050 -0.32082847 -4.61817895
H -1.00520215 0.27342341 -4.32288196
H 0.18854439 2.42814750 -2.84562923
H 1.80600652 1.73262135 -3.12969922
H 1.27086666 2.12405163 -1.47123431
C -0.36133488 0.35061118 2.49076847
C 0.38955271 -0.81542977 2.65905671
C 0.24452750 1.39862607 1.79020671
C 1.68285222 -0.93248397 2.17748669
H -0.03582189 -1.65988538 3.19116090
C 1.53839368 1.30850717 1.30929575
H -0.31486525 2.31040345 1.60854733
C 2.29480929 0.13354694 1.48998788
H 2.22111690 -1.85916195 2.33087613
H 1.96610469 2.15175377 0.78245872
N 3.55884819 0.02129177 0.98877265
C 4.16012408 1.00483588 0.09050336
C 4.36493400 -1.18841793 1.11394020
H 3.43833868 1.34289463 -0.65801800
H 4.50604688 1.88370598 0.64818610
C 5.32628047 0.24490434 -0.54292849
H 4.43960383 -1.50317720 2.15794912
H 3.92274153 -2.01507313 0.54053002
C 5.71409286 -0.76992981 0.53411571
H 4.97993735 -0.27890091 -1.43853252
H 6.14505580 0.90709366 -0.82720560
H 6.31384472 -0.28417509 1.30891807
H 6.27701034 -1.61872445 0.14363040
*

E23
C -0.08205521 3.03848676 1.44413788
H -0.00364271 2.05375839 1.90744262
H -0.58111982 3.69496648 2.16052957
C -0.93308832 2.93273863 0.25015977
C -1.58308164 2.97526682 -0.78165146
H -2.14322156 3.22867731 -1.66065235
C 2.33743677 3.63241863 -1.14651379
H 2.83502539 3.18053053 -1.99784234
H 2.18023368 4.70588784 -1.17511773
C 1.95005895 2.91452236 -0.09256321
C 1.31016767 3.59340741 1.09462877
H 1.93611771 3.48252721 1.98426229
H 1.22979383 4.66081054 0.88483720
Au -1.46110524 0.78078691 -0.33558632
P -1.77140921 -1.47582870 -0.17918552
C -3.51744570 -1.93072079 -0.26676617
C -0.95338267 -2.45768908 -1.45275025
C -1.18629471 -2.08354372 1.41784263
H -1.76848363 -1.60563712 2.20808357
H -1.31185196 -3.16682234 1.47676070
H -0.13585060 -1.82313828 1.54652724
H -4.06867137 -1.41485783 0.52102711
H -3.62138706 -3.01092625 -0.13998412
H -3.92278335 -1.63678775 -1.23645248
H -1.32477898 -2.15256169 -2.43284607
H -1.16941665 -3.51667630 -1.29454933
H 0.12202843 -2.28950182 -1.41291875
C 2.12516477 1.43782505 -0.05730711
C 2.37587349 0.76471749 1.14137526
C 2.08013913 0.67700695 -1.22431020
C 2.61841353 -0.59070441 1.13424038
H 2.43623712 1.28922004 2.08809531
C 2.32176398 -0.68978190 -1.20888282
H 1.84587019 1.15632956 -2.16784314
C 2.61264876 -1.37642585 -0.02490760
H 2.27975321 -1.23601913 -2.14202825
N 2.94853920 -2.73921426 0.01991506
C 3.22316550 -3.37275059 -1.26087529
C 2.11400376 -3.59507451 0.86374898
H 3.64709107 -4.36018231 -1.06803680
H 2.32171330 -3.50349216 -1.87731820
H 3.95375312 -2.78974802 -1.82390154
H 1.15658586 -3.82690656 0.37782041
H 2.64303309 -4.53344065 1.04407848
H 1.91477371 -3.12532472 1.82343448
F 2.92812410 -1.17630647 2.32164223
*

E24
C -3.44185856 0.28264867 -0.85809810
H -3.16151104 -0.59346330 -0.27160736
H -4.52902693 0.26196715 -0.97152016
C -2.86714718 0.13406390 -2.19924789
C -2.50184577 0.03542640 -3.35842786
H -2.39740583 -0.02037160 -4.42445643
C -0.88530322 2.47237739 -0.99157299
H 0.19058891 2.60119845 -0.99371077
H -1.43263501 2.92871079 -1.80932045
C -1.53578572 1.79430668 -0.04196732
C -3.02946925 1.59868696 -0.16398083
H -3.50611305 1.61335833 0.81801551
H -3.44063301 2.42808714 -0.74052491
Au -0.58799343 -0.28033183 -2.22063183
P 1.56417016 -0.82987469 -1.67977721
C 2.35962122 -1.85974260 -2.93327819
C 2.64990931 0.59799756 -1.47085073
C 1.66122489 -1.76624372 -0.13867796
H 1.11427315 -2.70385232 -0.25307141
H 2.70566167 -1.97808012 0.10118148
H 1.20805305 -1.18885949 0.66688934
H 1.78810932 -2.77993526 -3.06442037
H 3.37742947 -2.10177746 -2.61828755
H 2.38940516 -1.32074719 -3.88176207
H 2.66762155 1.17651400 -2.39621845
H 3.66051304 0.25847772 -1.23278740
H 2.27823874 1.22932708 -0.66419536
C -0.81325565 1.16819370 1.10149252
C -1.39880068 0.14844705 1.85394016
C 0.47305286 1.58215598 1.45020997
C -0.70414268 -0.45394803 2.89262698
H -2.40465097 -0.18261717 1.64069992
C 1.15962442 0.96986754 2.48726355
H 0.94193965 2.40021438 0.92020424

C 0.58537262 -0.05959423 3.21726541
H 1.12639388 -0.53889846 4.02431288
C 2.57996445 1.37820985 2.77637569
F 2.82049209 2.66489747 2.46189053
F 3.45899536 0.63573182 2.06025209
F 2.90781582 1.21750041 4.07162268
C -1.32502450 -1.57825328 3.68036720
F -1.27440806 -1.34949818 5.00916291
F -2.61623259 -1.78357896 3.36511547
F -0.67998267 -2.74822933 3.46939033
*

E3
C -3.18491452 -0.78787489 -1.66417733
H -2.77239442 -1.69967026 -1.23057901
H -4.10415456 -1.06329085 -2.18815290
C -2.24327146 -0.29816705 -2.67614436
C -1.55551874 0.11764988 -3.59308272
H -1.13922395 0.46925084 -4.51682857
C -1.71363371 1.91299558 -0.29616487
H -0.81030544 2.32182803 0.14084121
H -2.14602451 2.46391384 -1.12452579
C -2.29489210 0.79322261 0.14480461
C -3.50968270 0.26356406 -0.58166452
H -4.21396083 -0.19284534 0.11636755
H -4.01891956 1.09952074 -1.06278773
Au -0.07022098 -0.03812664 -1.91001991
P 1.89154521 -0.14912491 -0.74194464
C 3.32618648 -0.40591830 -1.81048256
C 2.26679586 1.33610345 0.21411290
C 1.90260627 -1.52216867 0.43098804
H 1.80746926 -2.46075061 -0.11811648
H 2.83828655 -1.52160397 0.99460369
H 1.05963655 -1.42560004 1.11525473
H 3.19832792 -1.33038184 -2.37588333
H 4.23103667 -0.47045294 -1.20155660
H 3.41454864 0.42865881 -2.50822897
H 2.31941981 2.19462191 -0.45779525
H 3.22528067 1.21125875 0.72310300
H 1.48312679 1.50837855 0.95112157
C -1.73836765 0.01775289 1.29037960
C -2.09854413 -1.31188104 1.51626937
C -0.82955902 0.60724933 2.17462764
C -1.54837109 -2.04042987 2.56575408
H -2.82040691 -1.80102341 0.87584001
C -0.28043384 -0.12323125 3.21434353
H -0.56019450 1.64849001 2.06262649
C -0.62712747 -1.45557162 3.41851962
H -1.84333605 -3.07281831 2.71493821
H -0.19016652 -2.02049946 4.23351646
C 0.76481498 0.50476772 4.09461667
F 0.70728763 1.85051638 4.09761211
F 2.01678670 0.17581881 3.68694389
F 0.66738412 0.10332787 5.37784768
*

E4
C -2.77415293 -1.29982885 -1.29504697
H -2.12596060 -1.76688807 -0.55137026
H -3.41386203 -2.08238190 -1.71072040
C -1.95600166 -0.77543941 -2.39674271
C -1.43236226 -0.31719087 -3.39926017
H -1.16120431 0.02937731 -4.37761923
C -2.87969208 2.15541105 -0.59792919
H -2.32295989 2.98818234 -0.18238156
H -3.51728540 2.36249825 -1.45120059
C -2.81797932 0.92976630 -0.07884661
C -3.63712247 -0.19201300 -0.66819031
H -4.26478352 -0.65627277 0.09528955
H -4.30218885 0.21581450 -1.43058968
Au 0.20012009 -0.14194908 -1.88905311
P 2.25767943 0.13756579 -0.92885559
C 3.57955744 -0.30325693 -2.07996615
C 2.62921637 1.82380535 -0.40226665
C 2.49432347 -0.92365446 0.51258222
H 2.42654950 -1.96733130 0.20035591
H 3.47630067 -0.73547189 0.95273790
H 1.71666731 -0.72160421 1.24794561
H 3.45352615 -1.33873798 -2.40019745
H 4.54778953 -0.18773582 -1.58708721
H 3.53528870 0.34833010 -2.95431571
H 2.55626761 2.49340969 -1.26096573
H 3.64220706 1.86143574 0.00509202
H 1.91908212 2.14260472 0.35998583
C -1.93359040 0.62245365 1.08106060
C -2.26219413 -0.38115189 1.99372045
C -0.74986872 1.34062291 1.28208232
C -1.45242342 -0.64433940 3.09070659
H -3.16698872 -0.96243614 1.86599073
C 0.05712453 1.09180608 2.37832696
H -0.45325480 2.09780346 0.56671102
C -0.29723955 0.09854338 3.28573705
H -1.72920039 -1.42334783 3.79072036
H 0.96933052 1.65979278 2.52106710
C 0.62121059 -0.19930560 4.43684971
F 1.68655176 -0.94588806 4.05215546
F 0.01354643 -0.88138793 5.42555650
F 1.13002477 0.92370997 4.98743566
*

E5
C -2.68705267 -1.10518600 0.38456871
H -1.78785591 -1.67381686 0.62502485
H -3.40084031 -1.80231858 -0.06026142
C -2.34780392 -0.09330766 -0.62583799
C -2.20094143 0.81296602 -1.42891701
H -2.28954698 1.61306051 -2.13786349
C -3.15460527 1.89882142 2.35386905
H -2.63516510 2.77235155 2.73385374
H -4.22077050 1.99330620 2.17351487
C -2.52287028 0.74660657 2.13314075
C -3.28719759 -0.46350900 1.64930651
H -3.31069144 -1.23595987 2.42307409
H -4.31888144 -0.16922820 1.45167769
Au -0.09636614 0.12413201 -1.06898922
P 2.14370761 -0.26581897 -1.29940855
C 2.55029730 -0.71466100 -3.00241100
C 3.19999138 1.14556218 -0.91140955
C 2.74073716 -1.63524112 -0.28496254
H 2.17062053 -2.53558384 -0.51977647
H 3.79914409 -1.80619704 -0.49549163
H 2.61283807 -1.39529011 0.77037340
H 1.99772212 -1.61285792 -3.28323147
H 3.62312041 -0.90318667 -3.08690678
H 2.26884873 0.09995007 -3.67182447
H 2.92859809 1.98821575 -1.54958656
H 4.24450167 0.87623153 -1.08450527
H 3.06317126 1.43125023 0.13122268
C -1.05340077 0.61387145 2.32375729
C -0.48193200 -0.57769014 2.77746082
C -0.19828088 1.68176145 2.04371854
C 0.88549765 -0.69382248 2.97522278
H -1.11375592 -1.43081848 2.99904632
C 1.17098729 1.58280644 2.24064214
H -0.60839111 2.60612403 1.65256789
C 1.71501659 0.39172585 2.71162851
H 1.31849595 -1.61965696 3.33755615
H 1.81968633 2.42451317 2.01784770
O 3.05558870 0.22916019 2.91050131
H 3.50960134 1.06305668 2.72441987
*

E6

C	-2.74021321	-1.08464308	-0.95369181
H	-2.04636711	-1.65353096	-0.33311197
H	-3.24840366	-1.79494120	-1.61003237
C	-1.97577607	-0.15628818	-1.79837732
C	-1.49178498	0.68134243	-2.54182845
H	-1.26159902	1.41525232	-3.28936730
C	-3.63065016	2.04288319	0.57440703
H	-3.22764919	2.87003092	1.14953016
H	-4.45312566	2.25895933	-0.10015827
C	-3.15533209	0.80429623	0.69857049
C	-3.76482388	-0.33334065	-0.08570068
H	-4.21748822	-1.06618341	0.58769239
H	-4.55946480	0.05975217	-0.72163338
Au	0.25341854	0.09125500	-1.27202003
P	2.38730798	-0.17321612	-0.49530133
C	3.51033799	-0.69775301	-1.81038899
C	3.10886372	1.34079174	0.17261944
C	2.53497211	-1.43429986	0.78815774
H	2.22637691	-2.39703397	0.37667581
H	3.57343815	-1.49529286	1.12199901
H	1.89146365	-1.18918080	1.63159012
H	3.16961761	-1.64951841	-2.22126747
H	4.51973349	-0.81298319	-1.40834722
H	3.51718482	0.05076049	-2.60457024
H	3.15315793	2.09194427	-0.61829958
H	4.11749153	1.13701579	0.53940589
H	2.48953852	1.71858335	0.98520931
C	-1.99580791	0.49680249	1.57839393
C	-1.91239227	-0.71277078	2.27883779
C	-0.95056163	1.40484373	1.72934782
C	-0.84336282	-0.98859986	3.11075012
H	-2.70289669	-1.44935724	2.18528094
C	0.12921858	1.14762443	2.56849531
H	-0.96859665	2.33625403	1.17391997
C	0.18649158	-0.05712949	3.26301594
H	-0.78810838	-1.92627158	3.65291041
H	0.91515207	1.88458893	2.66118862
O	1.20835934	-0.42389857	4.08193532
C	2.29508338	0.48860318	4.22334982
H	2.78228077	0.67405721	3.26123545
H	1.96130011	1.43566262	4.65721791
H	3.00159088	0.00972546	4.90007793

*

E7

C	-2.72831050	-1.15849120	-0.85052857
H	-2.01047583	-1.67873606	-0.21425146
H	-3.26818199	-1.91662417	-1.42280963
C	-2.00062258	-0.31053995	-1.80566277
C	-1.54955872	0.44412955	-2.65194973
H	-1.35319143	1.09247608	-3.48368389
C	-3.43248286	2.07541735	0.48139689
H	-2.98597289	2.91607510	1.00186967
H	-4.22704121	2.29279724	-0.22533813
C	-3.04649190	0.82093884	0.71000617
C	-3.71116968	-0.32978499	-0.00578635
H	-4.17925078	-1.01114650	0.70910403
H	-4.50043042	0.06056928	-0.64997514
Au	0.22493254	0.05760936	-1.34959023
P	2.34821915	-0.06817939	-0.51228333
C	3.54540872	-0.58435564	-1.76324936
C	2.97376462	1.49624504	0.13559629
C	2.48533150	-1.27608449	0.82149595
H	2.24718588	-2.26669013	0.42980664
H	3.50308701	-1.27400156	1.21803555
H	1.77930075	-1.02821079	1.61270696
H	3.26179537	-1.56176841	-2.15685013
H	4.54027501	-0.64466412	-1.31556452
H	3.55612168	0.13942757	-2.57998090
H	3.02179245	2.22501930	-0.67565799
H	3.97231015	1.34785013	0.55311649
H	2.30348255	1.87059732	0.90844562
C	-1.93531832	0.51103358	1.65129607
C	-1.95456304	-0.63616273	2.44609664
C	-0.84251592	1.37593621	1.77221681
C	-0.93164718	-0.89440794	3.34857197
H	-2.78419312	-1.33095097	2.37819581
C	0.17533432	1.11969237	2.67561120
H	-0.79215117	2.26226862	1.14940330
C	0.15340164	-0.02611442	3.47441027
H	-0.97236000	-1.78807439	3.96361739
H	0.99494910	1.82305077	2.76650651
C	1.24907110	-0.35832292	4.40794712
C	2.49453641	0.10304627	4.32794210
H	0.99421817	-1.06532065	5.19426222
H	3.24418798	-0.19848232	5.05198669
H	2.81230139	0.78196524	3.54189424

*

E8

C	-0.45543020	-1.29992373	2.83805659
H	0.18270640	-1.79425503	2.10367892
H	-0.80339989	-2.06337102	3.53845894
C	-1.64143770	-0.75299469	2.16605760
C	-2.69331693	-0.27079568	1.77850734
H	-3.69172983	0.09534891	1.63705814
C	0.34590598	2.14184726	2.82116805
H	0.69906372	2.95805398	2.20045955
H	-0.39112555	2.38014557	3.58116097
C	0.80642445	0.89846893	2.68727416
C	0.31110594	-0.20158670	3.59372353
H	1.14354455	-0.68082434	4.11345734
H	-0.34073111	0.22979337	4.35470427
Au	-1.41626680	-0.13184820	-0.04372466
P	-0.81326989	0.14432021	-2.23380351
C	-2.20062170	-0.23877834	-3.32748237
C	-0.31024032	1.81999650	-2.67910441
C	0.52283144	-0.95461235	-2.75045185
H	0.20945631	-1.99001425	-2.60579032
H	0.74801535	-0.78435083	-3.80581839
H	1.41247791	-0.76408581	-2.15043479
H	-2.52378283	-1.26749975	-3.16031349
H	-1.89099210	-0.11671718	-4.36812419
H	-3.03159149	0.43521181	-3.11263242
H	-1.12373112	2.51104744	-2.45147292
H	-0.08608747	1.85946249	-3.74762493
H	0.57321466	2.10969616	-2.11122225
C	1.79825800	0.54571434	1.63312770
C	2.71440947	-0.49265629	1.81840316
C	1.83361528	1.24918825	0.42504500
C	3.64813843	-0.80660791	0.84277001
H	2.71481347	-1.06287930	2.73942013
C	2.76931551	0.94959686	-0.54967925
H	1.11116310	2.03547212	0.24138914
C	3.68605640	-0.08565851	-0.35137118
H	4.35335611	-1.61362177	1.00508773
H	2.78548021	1.50547887	-1.47993015
C	4.63791820	-0.41941036	-1.37830904
C	5.42451030	-0.70403612	-2.24018865
H	6.12842889	-0.95464109	-3.00520826

*

E9

C	-0.39008150	-1.29752648	2.82856660
H	0.22021510	-1.78999805	2.06942935
H	-0.71668380	-2.06457229	3.53529034
C	-1.59675240	-0.74103755	2.20217042
C	-2.65937451	-0.25050906	1.85650706
H	-3.65981878	0.12363093	1.75568597
C	0.43140578	2.13376876	2.77954992
H	0.77448083	2.94474634	2.14647678

H	-0.28864377	2.37934287	3.55327512
C	0.88602074	0.88857678	2.64554373
C	0.40698677	-0.20772156	3.56468227
H	1.24858677	-0.69492845	4.06147513
H	-0.21987164	0.22922208	4.34317145
Au	-1.45261062	-0.12193584	-0.01377970
P	-0.90638783	0.14666792	-2.21959711
C	-2.32454701	-0.21984661	-3.27850348
C	-0.39119235	1.81470692	-2.67928777
C	0.40327508	-0.96967465	-2.76560352
H	0.07974865	-2.00099126	-2.61431074
H	0.60814809	-0.80199095	-3.82550433
H	1.30811898	-0.79151454	-2.18477025
H	-2.65768814	-1.24364302	-3.10110395
H	-2.03790136	-0.10417528	-4.32641895
H	-3.14083909	0.46600330	-3.04565659
H	-1.18623810	2.51863765	-2.42732358
H	-0.19892311	1.85076567	-3.75412879
H	0.51372851	2.09037329	-2.13872975
C	1.85994211	0.53408002	1.57513151
C	2.79509394	-0.48798062	1.76056848
C	1.85345761	1.22181534	0.35697677
C	3.71355216	-0.80300402	0.77234323
H	2.82243882	-1.04198562	2.69046781
C	2.77070834	0.92464715	-0.63451510
H	1.11372542	1.99282469	0.18023203
C	3.70236932	-0.09246947	-0.42632293
H	4.43778905	-1.59392058	0.92714159
H	2.76128163	1.46382783	-1.57418118
C	4.63891073	-0.42316188	-1.46748454
N	5.37990360	-0.68927687	-2.30627006

*

F0

C	-0.99847226	-1.18119237	1.12677406
H	-0.04133353	-1.68061240	1.30685758
H	-1.59678017	-1.85619764	0.51113514
C	-0.76257254	0.12930309	0.43143911
C	-0.95200118	1.27465148	1.08574661
H	-0.75062448	2.24288738	0.63733709
C	-1.44908540	1.39135338	2.55255417
H	-0.97689417	2.24980562	3.02035140
H	-2.53369557	1.53328826	2.54737211
C	-1.11713260	0.10606919	3.17757969
C	-1.75131725	-1.02631363	2.49762275
H	-1.72226606	-1.96010725	3.05132541
H	-2.78499957	-0.76259923	2.26781110
Au	-0.12198803	0.09237225	-1.53276185
P	0.58329567	0.05779645	-3.72990179
C	-0.71276155	-0.39050988	-4.91519871
C	1.21171496	1.64944346	-4.32750041
C	1.92673902	-1.11268534	-4.06252860
H	1.59941637	-2.12318597	-3.81017390
H	2.21039487	-1.07525930	-5.11702634
H	2.79040059	-0.85957014	-3.44453509
H	-1.09420461	-1.38562818	-4.67836929
H	-0.31503732	-0.38602252	-5.93284854
H	-1.53376653	0.32553842	-4.84273996
H	0.42767273	2.40452123	-4.24335386
H	1.52594384	1.56626555	-5.37065635
H	2.06166261	1.95734201	-3.71534165
C	-0.13706090	-0.04443186	4.20042146
C	0.36274409	-1.32583992	4.52792368
C	0.36454085	1.07851436	4.89658496
C	1.33835502	-1.47039659	5.49139300
H	0.00091899	-2.20263243	4.00849495
C	1.32423396	0.92268755	5.87521761
H	-0.01740679	2.06800841	4.68628359
C	1.81496290	-0.34817337	6.16698791
H	1.72875344	-2.45341993	5.72594236
H	1.69477958	1.78622189	6.41439094
H	2.57423214	-0.46650822	6.93271875

*

F1

C	-0.74460489	-1.37540696	0.77466385
H	0.29642003	-1.59222853	1.03556231
H	-1.08958446	-2.20347759	0.15154174
C	-0.82476135	-0.06951402	0.02970146
C	-1.34189218	1.00088815	0.63429026
H	-1.40452862	1.96739689	0.14254428
C	-1.87069930	1.03240056	2.08413143
H	-1.60988438	1.98168306	2.54294190
H	-2.96331029	0.94663352	2.06522057
C	-1.32385630	-0.14282248	2.78616415
C	-1.61725790	-1.39411042	2.07277465
H	-1.42861631	-2.29713854	2.64538505
H	-2.66252671	-1.37293678	1.75712561
Au	-0.10467485	0.00619222	-1.90447639
P	0.69581867	0.09029727	-4.06769745
C	-0.52985468	0.62391400	-5.29243444
C	2.08951780	1.22608385	-4.30166513
C	1.29863438	-1.49998230	-4.69551525
H	0.48808331	-2.23098812	-4.67162474
H	1.66030322	-1.39153659	-5.72083783
H	2.11110172	-1.85714612	-4.05966978
H	-1.37610489	-0.06586179	-5.28413321
H	-0.08619941	0.64314208	-6.29076279
H	-0.88973191	1.62209782	-5.03553552
H	1.78341412	2.23754831	-4.02742006
H	2.41774158	1.21567437	-5.34377573
H	2.91717715	0.92398648	-3.65699253
C	-0.48265308	-0.06695385	3.91941295
C	0.16988226	-1.22353853	4.42154517
C	-0.25263836	1.16318357	4.58258642
C	1.00917674	-1.14183883	5.50384982
H	0.02503517	-2.18280288	3.94441635
C	0.57807342	1.22955930	5.67730300
H	-0.74302167	2.06541377	4.24513921
C	1.22542496	0.08380145	6.15303951
H	1.50967860	-2.03177468	5.86924894
H	0.73522590	2.17733687	6.17928537
C	2.13768814	0.14941873	7.33743931
H	2.17799929	1.15439538	7.75758168
H	1.80156332	-0.54791685	8.10974800
H	3.14742467	-0.15425402	7.04611343

*

F10

C	-0.81977296	-1.57323237	0.90913637
H	0.22933221	-1.81582855	1.10545300
H	-1.20892231	-2.35688830	0.25551841
C	-0.91248875	-0.22289613	0.25714068
C	-1.41420693	0.81109409	0.93213531
H	-1.45776756	1.81291160	0.51548095
C	-1.96517417	0.74567212	2.38244984
H	-1.74747454	1.68218002	2.88707200
H	-3.04831771	0.59799269	2.34425863
C	-1.31985042	-0.42620426	2.98611860
C	-1.61715409	-1.66414559	2.25960036
H	-1.35594318	-2.57388297	2.79212618
H	-2.67866868	-1.67419279	2.00658938
Au	-0.21320222	-0.02505990	-1.67706089
P	0.56859045	0.19664162	-3.83704121
C	-0.03694061	1.67475958	-4.69396732
C	2.37344837	0.30639615	-3.96462211
C	0.12097576	-1.17723791	-4.93152794
H	-0.96630654	-1.26093375	-4.98346958
H	0.52127657	-1.00996208	-5.93433709
H	0.52499065	-2.10843510	-4.52954072
H	-1.12737681	1.64718042	-4.73914894
H	0.36815608	1.71949053	-5.70770247

H 0.26805161 2.56539846 -4.14108833
H 2.72624052 1.16970626 -3.39703620
H 2.67517323 0.41012822 -5.00961536
H 2.82275955 -0.59571754 -3.54479641
C -0.35771798 -0.34527871 4.03230144
C 0.46713518 -1.45250910 4.32624642
C -0.19653155 0.84507500 4.77886716
C 1.42768619 -1.35205693 5.31142705
H 0.37189411 -2.37338223 3.76737574
C 0.74682645 0.94222018 5.78392232
H -0.83735867 1.69526262 4.58647216
C 1.55996957 -0.16958916 6.03244465
H 2.07491309 -2.19361483 5.52792654
H 2.31158916 -0.10596030 6.81383130
C 0.90679694 2.19944120 6.59553797
H 0.70889567 2.00301848 7.65252370
H 1.92792858 2.58109286 6.51647424
H 0.22013774 2.97553190 6.25439167
*

F11
C -1.48515421 -1.01661149 0.79637803
H -0.68630276 -1.74128061 0.98117623
H -2.20565219 -1.49825897 0.13197236
C -0.90306790 0.21884216 0.17569962
C -0.85304382 1.35834238 0.86426444
H -0.37614952 2.25528938 0.48135663
C -1.43701242 1.56216329 2.29611267
H -0.84048716 2.30611174 2.81503049
H -2.46995341 1.90722302 2.21336099
C -1.37958619 0.22088922 2.87665062
C -2.22099575 -0.73924952 2.16132749
H -2.39024115 -1.67265927 2.69007535
H -3.17461762 -0.26580441 1.92403983
Au -0.14867337 0.07522119 -1.74306958
P 0.69640119 -0.09598564 -3.88324592
C -0.27380123 0.78158379 -5.13769971
C 2.37633557 0.55804540 -4.07083364
C 0.80107107 -1.79765239 -4.49882389
H -0.19453836 -2.24562688 -4.50033221
H 1.20702670 -1.80981174 -5.51313131
H 1.44751676 -2.38249903 -3.84162170
H -1.29113454 0.38579639 -5.15267365
H 0.17978398 0.65691185 -6.12387856
H -0.31480388 1.84357043 -4.88798991
H 2.38671830 1.61593301 -3.80130022
H 2.71513140 0.44249809 -5.10312531
H 3.05288782 0.02029900 -3.40379531
C -0.44138176 -0.16510765 3.88694951
C -0.17751540 -1.53008217 4.12160649
C 0.24149429 0.81150873 4.63715714
C 0.76457292 -1.89308152 5.06390129
H -0.68546735 -2.29732485 3.55447433
C 1.16316934 0.43081736 5.59589160
H 0.04386144 1.86634862 4.50536791
C 1.43093435 -0.92288958 5.80339465
H 0.98505489 -2.93970684 5.23654479
H 2.16054022 -1.21520094 6.55290528
O 1.77824132 1.41294777 6.30518733
H 2.39237721 1.02056388 6.94234448
*

F12
C -0.53880314 -1.57979236 0.33679017
H 0.49205905 -1.45965205 0.68646370
H -0.55702981 -2.48810191 -0.26970448
C -0.94552959 -0.38074775 -0.47986807
C -1.81280392 0.49511835 0.03079581
H -2.12635493 1.37901809 -0.51752844
C -2.43727860 0.40802002 1.43606283
H -2.50104492 1.40358027 1.86573631
H -3.45535899 0.00911727 1.35005739
C -1.64051351 -0.53873608 2.24178218
C -1.47500029 -1.83340849 1.56016286
H -1.07776151 -2.62547766 2.18748096
H -2.44732312 -2.13671537 1.16472088
Au -0.11244748 -0.14128584 -2.35409783
P 0.82350289 0.12209659 -4.44794643
C -0.24669247 -0.40391678 -5.81352894
C 1.26606457 1.83222438 -4.85571083
C 2.35849918 -0.81089315 -4.69300320
H 2.16406794 -1.87540870 -4.54819956
H 2.74691354 -0.64616028 -5.70087258
H 3.10075608 -0.48799015 -3.96035990
H -0.49373862 -1.46077145 -5.69525422
H 0.25703307 -0.25338629 -6.77139710
H -1.17171135 0.17568043 -5.79431487
H 0.37171924 2.45738934 -4.82017923
H 1.70610356 1.88355475 -5.85448089
H 1.98371285 2.20624096 -4.12289893
C -0.99255442 -0.20053237 3.44484167
C -0.10188931 -1.11082611 4.07658837
C -1.18803059 1.06245646 4.06330601
C 0.55743573 -0.77376453 5.22915493
H 0.07908603 -2.08434316 3.64224484
C -0.53589144 1.38598020 5.22582222
H -1.86721769 1.78365753 3.63054055
C 0.35338071 0.48162316 5.83089327
H 1.23736412 -1.48760525 5.67779015
H -0.70230468 2.35183374 5.68893964
C 1.03842135 0.87483177 7.07057414
C 1.35610932 -0.16440043 8.12961738
C 2.46320554 0.41852968 7.32789834
H 0.78004156 1.86246760 7.43072428
H 1.05407140 -1.18395935 7.92297798
H 1.23905753 0.15529891 9.15766197
H 3.11584054 1.14348925 7.79829339
H 2.92532901 -0.19767685 6.56608470
*

F13
C -1.75910718 -0.72841065 0.49338093
H -1.13340168 -1.59815385 0.71616741
H -2.51726242 -1.05510993 -0.22136043
C -0.90721122 0.37087866 -0.06862965
C -0.67094483 1.47037002 0.64565243
H -0.00486958 2.25865635 0.30886284
C -1.28257463 1.77077039 2.04895133
H -0.57864587 2.36941967 2.61883999
H -2.21599284 2.32278859 1.91899603
C -1.53870326 0.43963850 2.59779157
C -2.51625397 -0.31367235 1.81291577
H -2.91261034 -1.19635995 2.30608031
H -3.33049187 0.35114558 1.52298326
Au -0.09551528 0.10736464 -1.95114655
P 0.80760448 -0.18421826 -4.05440032
C -0.01993324 0.77168219 -5.35299604
C 2.55166000 0.29188786 -4.18231596
C 0.75908435 -1.89536609 -4.64942964
H -0.27691378 -2.23746711 -4.68866464
H 1.20236424 -1.96302127 -5.64574543
H 1.31351959 -2.53567424 -3.96062426
H -1.07118671 0.48165355 -5.40404678
H 0.45524515 0.58921682 -6.31981001
H 0.03860957 1.83533580 -5.11408097
H 2.66016015 1.34657833 -3.92203923
H 2.91568174 0.12889177 -5.19948892
H 3.14338899 -0.30373129 -3.48451379
C -0.75854048 -0.14727337 3.64372923
C -0.83391829 -1.53280212 3.90135704
C 0.11339361 0.63573589 4.42695810
C -0.05499505 -2.11531290 4.88453505

H -1.49242865 -2.16059714 3.31649205
C 0.87523639 0.05100404 5.41944578
H 0.18552816 1.70572828 4.27856998
C 0.79572892 -1.32616043 5.64553295
H -0.11460904 -3.18215156 5.06154685
H 1.40545500 -1.76617976 6.42708750
C 1.77692144 0.90455660 6.23731458
O 2.48721947 0.47832527 7.11821449
H 1.75993907 1.98036911 5.98540462
*

F16
C -0.36512617 -1.58536488 0.58121637
H 0.70289568 -1.54239067 0.82083393
H -0.50264754 -2.46049225 -0.05807901
C -0.77806039 -0.32771381 -0.14147597
C -1.52561587 0.57483484 0.49755146
H -1.84270761 1.49636499 0.01699576
C -1.98858982 0.46828386 1.95831590
H -1.92436764 1.44573192 2.42905380
H -3.04071839 0.15650420 1.98021973
C -1.19744806 -0.57596676 2.64733759
C -1.17942617 -1.83916106 1.88279528
H -0.77785851 -2.68893573 2.42656170
H -2.20774793 -2.06036563 1.58461777
Au -0.14179246 -0.05941441 -2.08698725
P 0.57257516 0.22925769 -4.26351514
C -0.39017796 1.43933601 -5.21002424
C 2.28673668 0.79998663 -4.41534928
C 0.51044784 -1.27959013 -5.26690963
H -0.51616130 -1.64938801 -5.30335060
H 0.85998147 -1.07658797 -6.28204769
H 1.14190864 -2.04504404 -4.81155017
H -1.43497457 1.12457975 -5.24572461
H -0.00074144 1.52372739 -6.22738984
H -0.33487583 2.41235629 -4.71793188
H 2.39656133 1.75988679 -3.90697166
H 2.55878534 0.91260708 -5.46756825
H 2.95368620 0.07596366 -3.94316715
C -0.45800776 -0.35713811 3.81651378
C 0.39790089 -1.36808692 4.33204562
C -0.52115834 0.88878697 4.51108267
C 1.16033857 -1.15259104 5.44962219
H 0.47114381 -2.32421015 3.83461365
C 0.22422282 1.09750371 5.63224018
H -1.17356952 1.68139416 4.16942938
C 1.08290072 0.07670763 6.10924520
H 1.82366605 -1.91459036 5.83998013
O 1.82015785 0.27779526 7.19431653
H 1.66450489 1.17403373 7.53909309
O 0.23491723 2.23521931 6.37932220
H -0.36605048 2.89526647 6.00408805
*

F17
C -0.11417040 -1.34727007 0.00488462
H 0.87963290 -1.01839148 0.32857464
H 0.04202497 -2.23934118 -0.60610117
C -0.77728606 -0.25861169 -0.80350756
C -1.77123460 0.44157186 -0.25132509
H -2.28044448 1.23153874 -0.79705698
C -2.28363390 0.27442393 1.18296267
H -2.46380784 1.25428958 1.61727980
H -3.24501240 -0.25635708 1.16115962
C -1.34156612 -0.55649088 1.97158084
C -0.95107611 -1.78063277 1.23933695
H -0.40932124 -2.50815569 1.83680656
H -1.86706600 -2.24560818 0.86322273
Au -0.10809720 0.09871213 -2.72279616
P 0.63360096 0.49272702 -4.87358305
C -0.60213793 0.15492236 -6.15683425
C 1.14688407 2.20367226 -5.18475300
C 2.06873568 -0.50326735 -5.35907067
H 1.81599683 -1.56300434 -5.28783436
H 2.36599286 -0.26868949 -6.38400472
H 2.90064829 -0.29596427 -4.68310171
H -0.90894592 -0.89126749 -6.09905931
H -0.18845013 0.35841716 -7.14746577
H -1.47804253 0.78559643 -5.99231769
H 0.30606920 2.87463312 -4.99817548
H 1.48162905 2.31916558 -6.21842774
H 1.96144844 2.46760450 -4.50741961
C -0.81274732 -0.18602513 3.21007711
C 0.18162374 -0.98743909 3.85876297
C -1.24790764 0.99273104 3.86753612
C 0.70618112 -0.62750847 5.06483622
H 0.53592926 -1.88530626 3.37837162
C -0.74016063 1.35760315 5.08806832
H -2.00575963 1.62056601 3.42190109
C 0.23969853 0.56628213 5.70372214
H -1.09561069 2.25550725 5.57318772
O 0.79116322 0.83627566 6.86892656
C 0.38115655 2.02274111 7.57725108
H -0.68131803 1.96947975 7.82046324
H 0.97336707 2.02925498 8.48940149
H 0.59836511 2.91181901 6.98287197
O 1.65338542 -1.30363922 5.74839707
C 2.15033086 -2.50214139 5.15034926
H 2.89570783 -2.89103548 5.84184645
H 1.34891643 -3.23583083 5.02351823
H 2.61952740 -2.29053316 4.18502073
*

F18
C -1.06002332 -1.17928351 0.23476482
H -0.06022819 -1.53527055 0.50991087
H -1.49771405 -1.96146530 -0.39073038
C -0.94207873 0.11070955 -0.54586013
C -1.27584200 1.25860308 0.05281966
H -1.21371059 2.20550236 -0.47825388
C -1.72560982 1.40243073 1.50362677
H -1.24007640 2.26915466 1.94671245
H -2.80667594 1.59769341 1.53177128
C -1.48606098 0.14547269 2.26958913
C -1.93380239 -1.04872409 1.50285352
H -1.91882452 -1.97287370 2.07430615
H -2.96317772 -0.86087369 1.18030791
Au -0.25869984 0.03907142 -2.49111426
P 0.50175450 -0.03952721 -4.67074209
C -0.74904015 0.39232449 -5.91090768
C 1.88360391 1.08160203 -5.02041483
C 1.10402336 -1.66851048 -5.19226701
H 0.29976996 -2.40082947 -5.09781872
H 1.44446199 -1.63442522 -6.22998294
H 1.93150488 -1.97312947 -4.54842275
H -1.59111587 -0.29825695 -5.83261916
H -0.32495989 0.33594978 -6.91633697
H -1.11015026 1.40580109 -5.72525166
H 1.57659604 2.10962555 -4.81796525
H 2.19188491 0.99339023 -6.06497095
H 2.72555341 0.83396595 -4.37094212
C -0.87143113 0.09341769 3.50119772
C -0.63186926 -1.15376829 4.17972695
C -0.43278061 1.27838247 4.19182867
C -0.02973666 -1.21966372 5.38961524
H -0.93671096 -2.08086885 3.71446027
C 0.16450604 1.23368020 5.40512266
H -0.58479233 2.24842896 3.73885788
C 0.39439724 -0.02317046 6.06324630
H 0.12837829 -2.18286247 5.85239254
H 0.46964779 2.15437131 5.88078077
N 0.97490608 -0.07662906 7.25120622

C 1.20220843 -1.36057423 7.91931221
C 1.40242570 1.14721124 7.93376918
H 1.68078053 -1.17450355 8.87662111
H 0.25560497 -1.87562941 8.09416139
H 1.85463056 -1.99677516 7.31804858
H 1.86018758 0.87626126 8.88096093
H 2.13564342 1.68659606 7.33114720
H 0.54699161 1.79689794 8.12934596
*

F2
C -1.11096894 -1.36144361 0.48661768
H -0.11332988 -1.67263936 0.81173586
H -1.48791135 -2.14440708 -0.17480210
C -1.02206230 -0.04057853 -0.22249813
C -1.50787171 1.05967828 0.35113157
H -1.42081143 2.04253953 -0.10200736
C -2.22601537 1.10183972 1.72950955
H -2.00260274 2.04510961 2.21863462
H -3.30492204 1.02467344 1.56906184
C -1.73954427 -0.08327139 2.44330006
C -2.05970461 -1.32750852 1.74072181
H -1.93172332 -2.22950636 2.33150871
H -3.08348095 -1.26958967 1.36840564
Au -0.10808680 0.00859028 -2.07512145
P 0.90575913 0.05680979 -4.14742120
C -0.20421147 0.52362915 -5.50224776
C 2.28863201 1.22174173 -4.27483986
C 1.60426490 -1.53424594 -4.66350774
H 0.81236479 -2.28505338 -4.69702410
H 2.06274197 -1.44483670 -5.65124781
H 2.35803619 -1.85230503 -3.94061193
H -1.03080822 -0.18753692 -5.55641416
H 0.33636908 0.52617014 -6.45179221
H -0.61061496 1.51907676 -5.31286634
H 1.93278363 2.23226501 -4.06472963
H 2.72006768 1.18954257 -5.27817032
H 3.05406602 0.96056781 -3.54153203
C -0.87806604 -0.02698887 3.57678128
C -0.21984942 -1.19078234 4.03567964
C -0.65076997 1.18886538 4.25895184
C 0.63924128 -1.14044454 5.11010581
H -0.36597516 -2.13888355 3.53688640
C 0.19265118 1.24340666 5.34634602
H -1.15240965 2.09494567 3.94981631
C 0.83224351 0.07758298 5.75623952
H 1.15378067 -2.02852229 5.45375852
H 0.35784233 2.17272540 5.87596790
Cl 1.89590832 0.14192023 7.11568952
*

F22
C -1.28039238 0.84146793 -0.37982005
H -1.50747213 -0.17346980 -0.03261125
H -2.11051104 1.13092847 -1.02933862
C 0.01593064 0.83339603 -1.15959759
C 1.11260567 1.34234921 -0.58858633
H 2.06037159 1.36523413 -1.12149555
C 1.20044278 1.89014036 0.83190426
H 2.11027820 1.52114551 1.30082370
H 1.28589074 2.98519890 0.79525232
C -0.02694804 1.56764959 1.61736507
C -1.26116056 1.81142589 0.82125097
H -2.17981695 1.73066211 1.39595177
H -1.19457734 2.83060791 0.42572490
Au 0.03260313 0.03393936 -3.06142248
P 0.05238428 -0.85129208 -5.19470951
C 0.82607459 0.21997552 -6.43681890
C 0.94228898 -2.42426929 -5.34488107
C -1.59332482 -1.20288504 -5.87073167
H -2.17755761 -0.28113384 -5.90344010
H -1.51088212 -1.61680683 -6.87860561
H -2.10619564 -1.91861692 -5.22519057
H 0.29046405 1.17031193 -6.48117757
H 0.80268458 -0.25697878 -7.41962147
H 1.86194060 0.41584777 -6.15278778
H 1.98085609 -2.28455929 -5.03838467
H 0.91270664 -2.78234213 -6.37678360
H 0.48145458 -3.16601802 -4.68951121
C -0.01448602 1.05454077 2.89357034
C -1.22971103 0.70376699 3.58592934
C 1.21129847 0.83480785 3.62052806
C -1.23060396 0.18979171 4.83626745
H -2.18205779 0.83975167 3.09221652
C 1.23119366 0.32528841 4.87244845
H 2.15598899 1.09662421 3.16396131
C 0.00572798 -0.02556777 5.53838854
H -2.16606036 -0.07463899 5.30913096
H 2.17389787 0.19268051 5.38459120
N 0.01595488 -0.52703440 6.75475066
C 1.24228418 -0.84489304 7.51219733
C -1.19680695 -0.84623566 7.53326421
H 1.94419372 -1.38840581 6.88056137
H 1.71008984 0.08546046 7.84605343
C 0.73086359 -1.67334647 8.68638339
H -1.90256361 -0.01735784 7.48743773
H -1.66846967 -1.73927019 7.11400872
C -0.66185455 -1.09701376 8.93970420
H 0.65689899 -2.72405722 8.39424855
H 1.39166877 -1.59864995 9.54994401
H -0.58556673 -0.15068971 9.48135231
H -1.30999721 -1.76830036 9.50295592
*

F23
C -0.41677754 -1.44090800 0.03525789
H 0.62423528 -1.32684931 0.35934033
H -0.44508099 -2.34258552 -0.58167856
C -0.83104249 -0.23538815 -0.77793799
C -1.65802163 0.65986117 -0.22886217
H -1.98659924 1.53485932 -0.78449433
C -2.19346535 0.61006955 1.20021287
H -2.15610317 1.60701502 1.63348980
H -3.25058346 0.31111964 1.18295532
C -1.46159401 -0.40661864 2.00645823
C -1.32164035 -1.68743880 1.26578956
H -0.93519140 -2.50722969 1.86537638
H -2.31463576 -1.96485610 0.89815314
Au -0.08551498 -0.01749704 -2.68955352
P 0.74939706 0.23558642 -4.82817350
C -0.18097072 -0.65550253 -6.10429607
C 0.78170477 1.95116674 -5.41487806
C 2.45656333 -0.33992478 -5.03520374
H 2.51478664 -1.40162795 -4.78736171
H 2.78727001 -0.18862061 -6.06554976
H 3.11105403 0.21312599 -4.35861061
H -0.17673673 -1.72352058 -5.87779334
H 0.26626100 -0.49041437 -7.08748649
H -1.21464827 -0.30410796 -6.11160950
H -0.23229116 2.35591728 -5.40938726
H 1.18707684 1.99985241 -6.42835816
H 1.40138170 2.55212938 -4.74642689
C -0.91326105 -0.16382695 3.24782345
C -0.19257700 -1.17745703 3.96859302
C -1.03102467 1.10537387 3.90730743
C 0.36580499 -0.92569629 5.16577914
H -0.09661739 -2.18291907 3.58216890
C -0.48001689 1.34893859 5.12037516
H -1.56840084 1.91167512 3.42847950
C 0.29095365 0.35759037 5.81835036
H -0.58167395 2.33201495 5.55499893
N 0.86438322 0.63231765 6.97747815

C	1.89908705	-0.19675798	7.61123431
C	0.55699078	1.87921924	7.68513481
H	2.53002401	0.46796318	8.19901641
H	1.45079705	-0.94091162	8.27182790
H	2.51700870	-0.68312724	6.86332805
H	0.82777155	1.74451593	8.73018635
H	1.13064409	2.71240705	7.27100733
H	-0.50775387	2.09935632	7.63165315
F	0.96602602	-1.95111097	5.81507924

*

F3

C	0.45124112	-2.05559403	-0.15534106
H	1.44129164	-1.63432432	0.04404705
H	0.57596492	-2.81320540	-0.93171902
C	-0.47235033	-0.95925000	-0.59155894
C	-1.46989208	-0.56170625	0.19671147
H	-2.10501887	0.28702077	-0.03648389
C	-1.85614361	-1.23460929	1.55450049
H	-2.31185280	-0.48822909	2.19728906
H	-2.56107267	-2.04522821	1.36299641
C	-0.57130364	-1.74705573	2.01890474
C	-0.03205958	-2.79452016	1.15253167
H	0.79018678	-3.35883760	1.58276897
H	-0.83954241	-3.46848857	0.86487987
Au	-0.12545421	-0.07117517	-2.42576463
P	0.25900310	0.91017751	-4.47824219
C	-1.22617952	1.09733916	-5.49982932
C	0.95737425	2.57981054	-4.38261521
C	1.41820488	-0.00937928	-5.52488272
H	1.02753576	-1.01274359	-5.70523879
H	1.55665832	0.50390746	-6.47941000
H	2.37972684	-0.09409018	-5.01480861
H	-1.66940404	0.11615349	-5.68071730
H	-0.97281673	1.56392311	-6.45476923
H	-1.95341455	1.71853782	-4.97341740
H	0.27189642	3.22906292	-3.83447652
H	1.11548275	2.98247106	-5.38597399
H	1.90977776	2.54642424	-3.84998024
C	0.18880837	-1.13995450	3.07330246
C	1.55618917	-1.43842350	3.22743082
C	-0.41478386	-0.22803397	3.96216595
C	2.30295374	-0.82932991	4.21879251
H	2.04125884	-2.13470913	2.55686951
C	0.33634786	0.35523011	4.95849959
H	-1.46757820	0.00131471	3.88585832
C	1.69491519	0.06422787	5.08902760
H	3.35742997	-1.05308106	4.32160055
H	2.27433025	0.53357330	5.87691287
C	-0.28855974	1.31625277	5.93712712
F	0.33910231	2.51097337	5.92645526
F	-0.21373581	0.85431435	7.20333037
F	-1.58615645	1.54855178	5.67765347

*

F5

C	-1.15662242	-0.93603682	0.87326279
H	-0.28541607	-1.55813752	1.10591410
H	-1.82028350	-1.55224127	0.26231675
C	-0.72201393	0.29293278	0.11420218
C	-0.72681230	1.47446891	0.73551785
H	-0.42215427	2.38610580	0.22864828
C	-1.09745900	1.70063335	2.20735114
H	-0.41051258	2.41926063	2.64593896
H	-2.10624497	2.13061302	2.26264002
C	-1.13126588	0.40241889	2.91888316
C	-1.92543624	-0.60230082	2.18367190
H	-2.13786318	-1.50650256	2.74602800
H	-2.86968034	-0.13259279	1.89479215
Au	-0.12446817	0.08629330	-1.85094444
P	0.53743042	-0.13788776	-4.05150158
C	-0.41978117	-1.35468150	-4.99510807
C	0.40552430	1.38849094	-5.02092864
C	2.26086500	-0.65861770	-4.26522041
H	2.41090482	-1.62583954	-3.78152526
H	2.50421898	-0.74168890	-5.32717579
H	2.92234811	0.07290923	-3.79696098
H	-0.32358404	-2.33564328	-4.52556770
H	-0.05847143	-1.40760285	-6.02490793
H	-1.47329894	-1.06827108	-4.99380076
H	-0.63120427	1.73080669	-5.01963100
H	0.73012839	1.21579351	-6.04985325
H	1.02952442	2.16147969	-4.56801533
C	-0.41043765	0.12597093	4.08604042
C	-0.39269158	-1.18636168	4.64646422
C	0.33986501	1.13637928	4.75588653
C	0.32528974	-1.47259951	5.77121536
H	-0.94519024	-1.98523213	4.17244908
C	1.05107342	0.86237862	5.88976886
H	0.34600824	2.14952969	4.37937703
C	1.05249760	-0.44747007	6.40227929
H	0.34713306	-2.47040377	6.19185367
H	1.61397210	1.63736209	6.39870308
O	1.73202690	-0.77298704	7.49745442
H	2.19901674	0.00204913	7.85167161

*

F6

C	-1.04766828	-1.22859977	0.37847776
H	-0.07169164	-1.64008571	0.65842280
H	-1.51392856	-1.96491240	-0.28033837
C	-0.86066391	0.08511370	-0.33950621
C	-1.18372410	1.22000799	0.28523455
H	-1.06220142	2.18792353	-0.19354321
C	-1.70869473	1.32071382	1.72433152
H	-1.26152904	2.18183033	2.21322062
H	-2.79442482	1.47956282	1.70146742
C	-1.45787588	0.04103465	2.42728680
C	-1.94553620	-1.11290610	1.64332060
H	-1.96200409	-2.05144757	2.18948031
H	-2.95879043	-0.88108715	1.30443470
Au	-0.07912061	0.06101180	-2.24997202
P	0.80483680	0.03115029	-4.38267465
C	-0.37600679	0.46367762	-5.68884578
C	2.18603017	1.17978508	-4.62915997
C	1.46425538	-1.57992958	-4.88949278
H	0.66818387	-2.32626537	-4.85407735
H	1.86562483	-1.52436807	-5.90424298
H	2.25680947	-1.88093511	-4.20155484
H	-1.20911819	-0.24185549	-5.67390514
H	0.11019855	0.43158022	-6.66685447
H	-0.76501132	1.46788227	-5.50929964
H	1.84947762	2.19952950	-4.43187905
H	2.55926238	1.11218201	-5.65386380
H	2.99061479	0.93615766	-3.93257967
C	-0.74518666	-0.07476926	3.62328181
C	-0.43116356	-1.35381582	4.17965782
C	-0.29407887	1.07313260	4.33599148
C	0.27962249	-1.46878268	5.33462684
H	-0.74687406	-2.25691622	3.67614274
C	0.40881072	0.97122266	5.50525580
H	-0.51491038	2.06326071	3.96258979
C	0.70765545	-0.30742807	6.01743577
H	0.52645762	-2.43654576	5.75401573
H	0.72760651	1.86497784	6.02262747
O	1.38244753	-0.51796630	7.13306058
C	1.84797264	0.61224174	7.89658913
H	1.00341603	1.21699941	8.23109132
H	2.36336333	0.18598149	8.75422396
H	2.54152382	1.21055138	7.30356475

*

F7
C -1.35622481 -0.90598817 0.56940878
H -0.51678831 -1.54509536 0.86250578
H -1.99732531 -1.51015682 -0.07629978
C -0.84670642 0.30797428 -0.16084443
C -0.87898844 1.49711200 0.44190587
H -0.50996743 2.39807444 -0.03981497
C -1.38458231 1.75087833 1.87646129
H -0.75075683 2.49010349 2.35722931
H -2.40366820 2.15314804 1.82724418
C -1.43596771 0.45793378 2.58334632
C -2.20228467 -0.54913533 1.83504882
H -2.44112194 -1.44608909 2.39807237
H -3.12527806 -0.08337171 1.48211694
Au -0.09881748 0.06596738 -2.07054876
P 0.73464311 -0.20378265 -4.20604622
C -0.18403858 0.70088330 -5.48042360
C 2.44964503 0.34740605 -4.41009239
C 0.74056382 -1.92073736 -4.78792520
H -0.27952167 -2.30972561 -4.78257998
H 1.14590729 -1.97726122 -5.80097653
H 1.35076340 -2.52955238 -4.11793570
H -1.22240785 0.36375854 -5.48776139
H 0.26092251 0.52998926 -6.46361126
H -0.16458716 1.76855942 -5.25328374
H 2.52199842 1.40836518 -4.16296866
H 2.77941864 0.19063242 -5.43988449
H 3.09514922 -0.21440470 -3.73215372
C -0.71488889 0.17359414 3.76486158
C -0.67130436 -1.14246955 4.28868559
C -0.00219864 1.18597295 4.45952546
C 0.05299733 -1.42415103 5.42110677
H -1.19780140 -1.94626186 3.79339240
C 0.70590101 0.89907215 5.59860240
H -0.02042929 2.20696133 4.10509754
C 0.75048772 -0.41283194 6.10054140
H 0.08605846 -2.43769296 5.80422685
H 1.23447356 1.69524997 6.10661117
C 1.49738298 -0.77503205 7.31350261
C 2.16633891 0.07626333 8.08868244
H 1.47690608 -1.83027381 7.57068725
H 2.68940249 -0.28182811 8.96891416
H 2.21828454 1.14070763 7.88673338
*

F8
C -1.06573325 -1.36579709 0.58285226
H -0.06644487 -1.67886079 0.90091908
H -1.44848765 -2.14713132 -0.07715083
C -0.98048635 -0.04366117 -0.12399054
C -1.45879685 1.05632689 0.45621040
H -1.37349000 2.04008533 0.00467060
C -2.16475188 1.09699520 1.84113619
H -1.93282589 2.03759017 2.33136861
H -3.24524958 1.02500870 1.68893210
C -1.67755949 -0.09202044 2.54730758
C -2.00663116 -1.33280674 1.84354554
H -1.87716028 -2.23694900 2.43065114
H -3.03230083 -1.27073955 1.47714693
Au -0.08734339 0.00853418 -1.98675230
P 0.89730706 0.06085007 -4.07292717
C -0.22614832 0.55550901 -5.40661492
C 2.29481847 1.20694700 -4.21093617
C 1.56483322 -1.53537706 -4.61327185
H 0.76167421 -2.27434225 -4.64141722
H 2.00979892 -1.44378087 -5.60697395
H 2.32457926 -1.87061622 -3.90453705
H -1.06346181 -0.14346907 -5.45438345
H 0.30009051 0.55887182 -6.36418504
H -0.61552442 1.55483154 -5.20251475
H 1.95687939 2.22043579 -3.98605947
H 2.71045733 1.17799258 -5.22100467
H 3.06735293 0.92777965 -3.49184212
C -0.80689483 -0.04219227 3.67555714
C -0.14862545 -1.20943312 4.12456316
C -0.57184598 1.17023837 4.36064853
C 0.71757102 -1.16227144 5.19243063
H -0.30210854 -2.15434531 3.62192365
C 0.28051791 1.21487527 5.44065818
H -1.07478856 2.07783740 4.05801522
C 0.93472924 0.05007789 5.86050286
H 1.22967267 -2.05661579 5.52456280
H 0.44909096 2.14473094 5.96916564
C 1.82764087 0.09664887 6.98267263
C 2.57552374 0.13392743 7.92030975
H 3.24129399 0.16854352 8.75740582
*

G0
C -1.82130930 -1.11713531 0.92192579
H -1.71910363 -2.06752562 0.39624452
H -2.81182266 -0.73035424 0.63969739
C -0.82679178 -0.12260109 0.42957490
C -0.40134111 0.69423446 1.48467810
H 0.47397674 1.32547257 1.43236620
C -1.56093492 1.24971653 2.39230811
H -1.27480205 2.19724750 2.83058444
H -2.55778446 1.20090476 1.97249185
C -0.90664810 0.04237982 2.84850522
C -1.61639052 -1.23979589 2.43963739
H -0.98307295 -2.10024265 2.66026011
H -2.55282895 -1.35434453 2.98925005
Au -0.15984633 -0.01581882 -1.48920475
P 0.58581058 0.11200049 -3.68117321
C -0.49218263 1.10836079 -4.74031021
C 2.22907809 0.85172133 -3.84738203
C 0.70358349 -1.49649995 -4.50230181
H -0.27744255 -1.97517694 -4.50620080
H 1.05093301 -1.36851162 -5.53018141
H 1.40406059 -2.13243925 -3.95785751
H -1.49375379 0.67454140 -4.75089807
H -0.09564178 1.13506863 -5.75793327
H -0.55158562 2.12431349 -4.34564084
H 2.21504773 1.86360987 -3.43828829
H 2.51946107 0.88779357 -4.89991236
H 2.95433867 0.25611486 -3.28991041
C 0.02507874 0.04664900 4.01311234
C 1.12701430 -0.80972383 4.02371075
C -0.20093307 0.87817080 5.10690606
C 1.99163275 -0.82859999 5.10861956
H 1.31174429 -1.45583110 3.17133649
C 0.66355174 0.85747510 6.19653320
H -1.06075133 1.53933479 5.11167849
C 1.76079038 0.00582271 6.19903701
H 2.84907372 -1.49297946 5.10419697
H 0.47616230 1.50683259 7.04495167
H 2.43608748 -0.00960647 7.04777282
*

G1
C -2.01895266 -1.12782993 0.51546205
H -1.86693859 -2.07200805 -0.00888751
H -2.99570032 -0.75379460 0.17403141
C -1.01095805 -0.11060249 0.10034775
C -0.69057877 0.71990760 1.17843139
H 0.17040449 1.37260550 1.19110180
C -1.91306641 1.22774290 2.02713414
H -1.67913874 2.17656837 2.49273645
H -2.88142313 1.15889709 1.54737272
C -1.26008365 0.03225680 2.51026908
C -1.91023010 -1.26102032 2.04182328
H -1.27286774 -2.10921459 2.29513297

H	-2.87709944	-1.40329083	2.52904522
Au	-0.18028965	-0.00522234	-1.75429854
P	0.76045642	0.11726926	-3.86979147
C	-0.00462322	1.37541847	-4.92240806
C	2.52431627	0.52246919	-3.85164626
C	0.63838713	-1.42513377	-4.80855008
H	-0.41243101	-1.69616662	-4.92661001
H	1.09647033	-1.30391903	-5.79292119
H	1.14899612	-2.22219668	-4.26488510
H	-1.06620927	1.15206311	-5.04341432
H	0.47924704	1.38843181	-5.90180206
H	0.09866116	2.35497934	-4.45199229
H	2.67040589	1.48756690	-3.36300905
H	2.90904358	0.56887049	-4.87308038
H	3.06722109	-0.24173340	-3.29237127
C	-0.39092556	0.04406506	3.72050840
C	0.70700292	-0.81039591	3.80003635
C	-0.66390283	0.88453750	4.79821448
C	1.51571546	-0.81842191	4.92816526
H	0.93847379	-1.46770569	2.96771368
C	0.14545369	0.87076044	5.92604306
H	-1.52114910	1.54821336	4.76058052
C	1.24872434	0.02146842	6.00912172
H	2.37026332	-1.48651699	4.97001902
H	-0.08568506	1.52920298	6.75811068
C	2.12548099	0.02160120	7.23387030
H	2.89776134	-0.74667404	7.16517380
H	2.61770574	0.99044912	7.35823518
H	1.53549242	-0.16286353	8.13565723

*

G10

C	-1.87271468	-1.21580906	0.59816383
H	-1.76402529	-2.14387289	0.03544256
H	-2.86190578	-0.81930065	0.32482629
C	-0.87647588	-0.19886227	0.15658410
C	-0.47147987	0.58242201	1.24493607
H	0.40022026	1.22054462	1.22713609
C	-1.63961843	1.08922242	2.16943398
H	-1.36299668	2.01943027	2.64894679
H	-2.63334269	1.05138439	1.74112243
C	-0.98102386	-0.13144163	2.57938552
C	-1.67885142	-1.39979714	2.11106427
H	-1.04135529	-2.26444828	2.30070660
H	-2.61861530	-1.54343000	2.64815986
Au	-0.17420615	-0.02428838	-1.74477083
P	0.62033506	0.18152630	-3.91332577
C	0.02648020	1.67164632	-4.75201933
C	2.42557100	0.26893082	-4.01069374
C	0.15369470	-1.19083804	-4.99687141
H	-0.93450890	-1.25848545	-5.04966227
H	0.55814259	-1.03185241	-5.99919528
H	0.54563001	-2.12476716	-4.58971985
H	-1.06398194	1.65364837	-4.79684806
H	0.43223012	1.71806233	-5.76528384
H	0.34215589	2.55389731	-4.19215108
H	2.77950911	1.12822457	-3.43812153
H	2.74054155	0.36956501	-5.05190536
H	2.85704294	-0.63949897	-3.58626329
C	-0.05256903	-0.17147623	3.74606131
C	1.05015198	-1.02267017	3.72579827
C	-0.28798030	0.61743081	4.87059639
C	1.90485258	-1.07229271	4.81904022
H	1.24453169	-1.63540524	2.85150383
C	0.55952902	0.57401953	5.97552052
H	-1.15303571	1.27264888	4.89168639
C	1.66097419	-0.28065825	5.93462806
H	2.76770679	-1.72971129	4.80075827
C	0.29479550	1.42487463	7.19090309
H	2.33349010	-0.32585609	6.78610155
H	0.14208529	0.80124423	8.07608352
H	1.14316984	2.08313715	7.39729629
H	-0.59320398	2.04424324	7.05202509

*

G11

C	-2.11349888	-0.66940585	0.58853902
H	-2.24487681	-1.58977510	0.01770760
H	-2.96191432	-0.02732391	0.30876242
C	-0.88213488	0.05288481	0.16205253
C	-0.28806560	0.68076393	1.26423585
H	0.72204180	1.06410784	1.25798173
C	-1.28801425	1.47765464	2.18245826
H	-0.77813771	2.29783968	2.67121392
H	-2.25258564	1.70640880	1.74739504
C	-0.98157418	0.12192456	2.58442113
C	-1.99074755	-0.90900730	2.10119438
H	-1.61228119	-1.91474051	2.28844763
H	-2.93956290	-0.79696316	2.62964175
Au	-0.15727425	0.07260842	-1.73838045
P	0.66020602	0.10416197	-3.90806995
C	-0.34831572	1.09771302	-5.03581000
C	2.33231841	0.78234706	-4.04518103
C	0.74934806	-1.53505712	-4.67005084
H	-0.24683314	-1.98086147	-4.69088111
H	1.13413168	-1.45411595	-5.68935102
H	1.40973544	-2.17438584	-4.08127671
H	-1.36315199	0.69687205	-5.06526797
H	0.08192050	1.07585011	-6.03978433
H	-0.38591593	2.12831226	-4.67810054
H	2.34022203	1.80788954	-3.67137062
H	2.65724501	0.77197349	-5.08813371
H	3.01846652	0.18281512	-3.44408501
C	-0.10673622	-0.17618411	3.75584148
C	0.71621159	-1.30392439	3.73497954
C	-0.12170606	0.64996401	4.87264800
C	1.51800539	-1.58936938	4.82964129
H	0.73441046	-1.94528166	2.86070260
C	0.68545000	0.35299008	5.96886446
H	-0.76279803	1.52339959	4.91177180
C	1.50962816	-0.76692068	5.95123870
H	2.16354534	-2.46096402	4.81493907
O	0.62397651	1.19827012	7.03698700
H	2.13704817	-0.99194306	6.80848986
H	1.22322108	0.88229175	7.72775213

*

G12

C	-1.17603194	2.15256262	-0.14490731
H	-2.12069868	1.90156994	-0.62876856
H	-0.85620974	3.10398759	-0.59537714
C	-0.11807364	1.15465993	-0.47361426
C	0.74737858	0.99271626	0.61249007
H	1.44287980	0.17064235	0.70135110
C	1.20819585	2.32390605	1.31158549
H	2.17581571	2.18820760	1.77754852
H	1.08111514	3.22909417	0.73126011
C	0.05674055	1.67084980	1.89134107
C	-1.27674769	2.20096988	1.38689859
H	-2.08501777	1.55153283	1.72595369
H	-1.46123031	3.20575239	1.77264814
Au	-0.00506752	0.13472085	-2.23096742
P	0.12748416	-1.02018755	-4.23693126
C	1.12814348	-0.17004131	-5.48308380
C	0.86549908	-2.66512924	-4.07780970
C	-1.47784069	-1.29604424	-5.02593567
H	-1.95336950	-0.33464983	-5.22882897
H	-1.34611399	-1.84276094	-5.96253094
H	-2.11838823	-1.87176395	-4.35527959
H	0.69650373	0.81119265	-5.68895536
H	1.15627636	-0.75656900	-6.40435397
H	2.14338942	-0.03659172	-5.10498071

H 1.87761148 -2.57248384 -3.67954010
H 0.90085176 -3.15625527 -5.05302287
H 0.26906588 -3.26607400 -3.38887996
C 0.13414164 0.94480277 3.18914168
C -0.65855339 -0.18366307 3.40797456
C 0.96888703 1.38038606 4.21271704
C -0.61203164 -0.86014581 4.61510952
H -1.31093378 -0.54135367 2.61755282
C 1.01096970 0.70291104 5.42602000
H 1.58472553 2.26178934 4.06920086
C 0.22552805 -0.42664804 5.64853415
H -1.23463118 -1.73805028 4.75105548
H 1.66438865 1.06012276 6.21578032
C 0.30010752 -1.12772130 6.95935510
C -0.92607099 -1.76587340 7.56488229
C 0.17685465 -2.62997922 7.03852864
H 0.99094403 -0.67269358 7.65968066
H -1.84451418 -1.71128296 6.99180931
H -1.04764522 -1.68724567 8.63864223
H 0.81084626 -3.14337536 7.75157127
H 0.00543671 -3.15986464 6.10857176
*

G13
C -2.14922957 -0.73750575 0.29138148
H -2.26163931 -1.64950749 -0.29679097
H -2.99496467 -0.09650129 0.00113121
C -0.91373004 -0.00197632 -0.09711095
C -0.34280548 0.60490538 1.03063477
H 0.66663967 0.98950486 1.05207857
C -1.36473273 1.38903337 1.93421986
H -0.86843191 2.20284734 2.44720258
H -2.32003837 1.62252030 1.48174734
C -1.06450467 0.02673530 2.32274842
C -2.06037893 -0.99931367 1.80279541
H -1.68375648 -2.00709780 1.98390149
H -3.02125074 -0.89729701 2.31094122
Au -0.15294548 0.06397143 -1.98196653
P 0.70999270 0.14884448 -4.13282607
C -0.13330070 1.35009918 -5.19186476
C 2.45948206 0.60684703 -4.19410133
C 0.59989847 -1.42545518 -5.01837885
H -0.44588232 -1.73025023 -5.08879955
H 1.01784331 -1.32084780 -6.02233334
H 1.15423001 -2.19009244 -4.47102342
H -1.19130687 1.09176331 -5.26469822
H 0.31258838 1.34656737 -6.18919988
H -0.04220262 2.34658705 -4.75577107
H 2.59375070 1.59054435 -3.74039527
H 2.80439069 0.63310289 -5.23032349
H 3.04635517 -0.12313441 -3.63350818
C -0.22117358 -0.28308203 3.51297997
C 0.61677060 -1.39809356 3.50001618
C -0.26677355 0.50997912 4.65438673
C 1.40065367 -1.71459673 4.60378506
H 0.66271647 -2.01960960 2.61145346
C 0.51559124 0.19423427 5.76250317
H -0.91882190 1.37716756 4.69279756
C 1.35289261 -0.91927037 5.73886447
H 2.05039296 -2.58201489 4.57296307
C 0.43601267 1.06366502 6.96075786
H 1.95710956 -1.15058697 6.60874962
O 1.07099594 0.88676488 7.97718065
H -0.26267150 1.91682693 6.87275678
*

G14
C -1.90778368 -1.44269174 -0.04250109
H -1.59923357 -2.39687435 -0.47332472
H -2.86967647 -1.20671694 -0.52123948
C -0.96701333 -0.35942340 -0.43623745
C -0.79470061 0.53447179 0.63473710
H 0.00832895 1.25557151 0.68499323
C -2.15323424 1.01635789 1.26802438
H -2.04727186 2.01334633 1.67631688
H -3.04843547 0.83134163 0.68827516
C -1.47033662 -0.08632460 1.91795764
C -1.96542984 -1.46117952 1.49329896
H -1.29885618 -2.23234268 1.88228921
H -2.96897892 -1.64892333 1.87940806
Au -0.05679112 -0.22059771 -2.24816287
P 0.96927729 -0.05093014 -4.32142201
C 0.00287399 0.90903492 -5.51303807
C 2.58881742 0.75445162 -4.26319347
C 1.26128156 -1.64763073 -5.12118262
H 0.30980759 -2.16374425 -5.26196503
H 1.74223531 -1.49822959 -6.09064909
H 1.90439134 -2.25978018 -4.48619291
H -0.96992046 0.43555373 -5.65787683
H 0.53049233 0.95822915 -6.46840907
H -0.14954607 1.91913080 -5.12843708
H 2.47852379 1.76358010 -3.86184041
H 3.01757445 0.80598813 -5.26671302
H 3.25500772 0.18622918 -3.61145574
C -0.77925846 0.08234125 3.22916246
C 0.39615014 -0.62614127 3.48389043
C -1.30472589 0.91912649 4.21029890
C 1.04612974 -0.49924686 4.69894888
H 0.81050407 -1.27476103 2.72016201
C -0.66764082 1.05604918 5.43542903
H -2.22218358 1.46425418 4.02254053
C 0.49819055 0.34359345 5.65457153
H 1.96082389 -1.03827284 4.90434672
H -1.06910490 1.70118860 6.20500859
N 1.18409279 0.48516048 6.95153700
O 0.68627680 1.22044671 7.78358805
O 2.21361317 -0.14074628 7.12330750
*

G15
C -1.68081688 -1.64883047 0.07536147
H -1.27509946 -2.53648851 -0.41254137
H -2.69704942 -1.53284901 -0.33001345
C -0.92872512 -0.42982418 -0.32939691
C -0.83219512 0.45493179 0.75713430
H -0.14119384 1.28495070 0.78856953
C -2.19476079 0.70330008 1.50212988
H -2.21005394 1.69052466 1.94579832
H -3.09191688 0.40311245 0.97609365
C -1.31461511 -0.30487756 2.05909958
C -1.63054737 -1.72361070 1.60934316
H -0.83347974 -2.39952483 1.92246565
H -2.56667099 -2.07066595 2.05069423
Au -0.12046645 -0.13340789 -2.17086721
P 0.80630086 0.19159848 -4.27216438
C -0.42120217 0.21473646 -5.60178052
C 1.71564600 1.74739779 -4.43665402
C 1.98484280 -1.10054151 -4.73812190
H 1.48086591 -2.06880482 -4.73765969
H 2.38651990 -0.89897965 -5.73391063
H 2.80170707 -1.12722111 -4.01450597
H -0.96192250 -0.73337175 -5.61371616
H 0.07390134 0.36428705 -6.56409779
H -1.13238273 1.02443161 -5.42838210
H 1.04270697 2.58439706 -4.24125091
H 2.12578790 1.83714754 -5.44520943
H 2.52921865 1.77180078 -3.70916311
C -0.56191116 -0.07300096 3.32578735
C 0.70013058 -0.64497842 3.48465805
C -1.11716572 0.68708987 4.34991505
C 1.38816981 -0.44419010 4.66130723
H 1.15687222 -1.23588087 2.69941306

C -0.40345910 0.86944700 5.51915331
H -2.10013637 1.13262313 4.25888472
C 0.85088577 0.31236411 5.69229066
F -0.92123450 1.59594295 6.52381345
F 1.53212470 0.49846388 6.82865306
F 2.60891559 -0.97744972 4.83708118
*

G16
C 2.00791322 1.19658359 0.24844552
H 1.86607298 2.12112401 -0.31205689
H 2.98942190 0.80798474 -0.06186388
C 1.00551466 0.16384451 -0.14242261
C 0.68252748 -0.63734235 0.95592787
H -0.17594975 -1.29299441 0.98135086
C 1.89233284 -1.10227270 1.84336856
H 1.65792208 -2.03520310 2.33988992
H 2.86804557 -1.04431002 1.37710222
C 1.22690846 0.10583043 2.27434813
C 1.87581808 1.38491830 1.76698417
H 1.22969065 2.23760749 1.97918307
H 2.83434327 1.55124484 2.26317777
Au 0.18212188 0.00359978 -1.99663389
P -0.74849429 -0.18413106 -4.11181993
C -0.01218485 -1.50904970 -5.10124434
C -2.52318359 -0.53872147 -4.08912835
C -0.57670107 1.31050060 -5.11774487
H 0.48206907 1.54677850 -5.23932239
H -1.03100085 1.15732929 -6.09942356
H -1.06848799 2.14536080 -4.61502371
H 1.05617888 -1.32122368 -5.22292373
H -0.48909166 -1.55200552 -6.08319535
H -0.14667626 -2.46344137 -4.58884156
H -2.69940131 -1.47701821 -3.55991596
H -2.90272683 -0.61907108 -5.11040704
H -3.04777426 0.26411715 -3.56779738
C 0.33753146 0.13314257 3.46844666
C -0.76025759 0.98867293 3.50380050
C 0.59985270 -0.68229080 4.57010480
C -1.58941325 1.02810043 4.61690193
H -0.98057112 1.62157412 2.65105258
C -0.22382335 -0.64148773 5.68238091
H 1.45739090 -1.34788265 4.57105189
C -1.32758666 0.21631494 5.71049628
H -2.45028992 1.68680583 4.64861537
O -0.03330034 -1.40456788 6.80251696
O -2.14603096 0.26570005 6.79632046
H 0.76188450 -1.94509900 6.69964299
H -1.82114089 -0.35871135 7.46113748
*

G17
C -1.61590132 1.86323459 -0.25010669
H -2.46675769 1.39720191 -0.74769327
H -1.54177916 2.87729385 -0.67054162
C -0.33777358 1.17145674 -0.58955456
C 0.54947755 1.23360616 0.48543833
H 1.43465462 0.61872266 0.56229724
C 0.63623802 2.60882216 1.23929793
H 1.60335539 2.71673303 1.71327185
H 0.27682783 3.46871212 0.68776005
C -0.30626208 1.66372478 1.78766373
C -1.72927990 1.84252773 1.28121106
H -2.34465601 0.99822991 1.59460203
H -2.16797720 2.75460537 1.69151720
Au 0.00944296 0.20741042 -2.34879662
P 0.39589103 -0.88204276 -4.35822175
C 0.59437943 0.24791141 -5.75847372
C 1.88935388 -1.90404228 -4.35853050
C -0.94321474 -2.00074642 -4.83916398
H -1.87221025 -1.43549118 -4.93408226
H -0.70828236 -2.47904773 -5.79292380
H -1.07252702 -2.76512901 -4.07063621
H -0.30828958 0.85166188 -5.86876724
H 0.76786031 -0.31964594 -6.67571319
H 1.44180168 0.91085035 -5.57452865
H 2.75845634 -1.27701386 -4.15097139
H 2.01284993 -2.38865596 -5.32980604
H 1.80926264 -2.66456294 -3.57955889
C -0.03883313 0.93306641 3.05548166
C -0.55075455 -0.36379571 3.22166861
C 0.68485533 1.51147579 4.08036459
C -0.33410179 -1.06508202 4.39167112
H -1.10974402 -0.81671667 2.41313823
C 0.90778405 0.81416985 5.27137811
H 1.07511808 2.51717425 3.97546182
C 0.40998288 -0.46631135 5.43871073
O -0.78296903 -2.32232538 4.63462111
H 1.47164412 1.28809277 6.06364853
O 0.57477471 -1.22378981 6.54937551
C -1.54027247 -2.95164584 3.60512963
C 1.31697412 -0.65140473 7.62385985
H -1.80713223 -3.93516415 3.98988726
H -2.45180272 -2.38764093 3.38421505
H -0.94706366 -3.06666812 2.69268808
H 0.83009709 0.25280886 8.00108939
H 1.33606932 -1.40726475 8.40791131
H 2.34101574 -0.41968873 7.31606441
*

G18
C -0.17720268 2.54509629 -0.21871020
H -1.11926360 2.67536267 -0.75055304
H 0.49952121 3.32515151 -0.59706690
C 0.46308835 1.22905768 -0.52943726
C 1.24511864 0.79500793 0.53080324
H 1.61542702 -0.21615873 0.62221092
C 2.02939167 1.86296211 1.36227415
H 2.86627434 1.40387035 1.87255519
H 2.24299043 2.78597031 0.83642679
C 0.69877600 1.62505404 1.85730384
C -0.34687566 2.58063221 1.30603686
H -1.34653346 2.23688032 1.57261235
H -0.20498513 3.57599746 1.73362037
Au 0.12554269 0.16868217 -2.23784930
P -0.26416674 -1.03324094 -4.17988052
C 0.50762491 -0.30328634 -5.64562846
C 0.35313265 -2.73372958 -4.11970145
C -2.01941876 -1.18349842 -4.59665335
H -2.44743010 -0.18817723 -4.72996880
H -2.13875111 -1.75818526 -5.51808020
H -2.54473630 -1.68708311 -3.78301710
H 0.12241500 0.70717012 -5.79444387
H 0.28796953 -0.91069571 -6.52674911
H 1.58814416 -0.25086527 -5.49963067
H 1.43044197 -2.72318762 -3.94404286
H 0.14262777 -3.24402727 -5.06250411
H -0.13196521 -3.26761373 -3.30040115
C 0.43932643 0.83321890 3.08175634
C -0.75412654 0.12277190 3.23045254
C 1.35534154 0.78127683 4.13113112
C -1.02229509 -0.61643499 4.36632663
H -1.48997462 0.13657087 2.43252499
C 1.10153335 0.05204265 5.28100839
H 2.28310822 1.33977324 4.06584638
C -0.09074926 -0.68432741 5.42492942
H -1.96059470 -1.15024913 4.42755391
H 1.83834171 0.06212389 6.07209593
N -0.33474343 -1.44710224 6.54565432
C -1.69128932 -1.91028961 6.79398058
C 0.51199412 -1.26297539 7.71366162
H -1.69672568 -2.50120084 7.70868602

H	-2.40082959	-1.08008118	6.91013676
H	-2.03950902	-2.55336775	5.98334650
H	0.19383792	-1.95881918	8.48865287
H	1.55495835	-1.48487956	7.47817174
H	0.45439429	-0.24234835	8.11548405

*

G19

C	-2.30635955	-0.82537035	-0.67232291
H	-2.26860679	-1.82642772	-1.10598178
H	-3.14662184	-0.32566051	-1.17683884
C	-1.08696822	-0.05499370	-1.03035702
C	-0.68712080	0.73832343	0.06449114
H	0.29208979	1.18757683	0.14589975
C	-1.85804912	1.59816028	0.66507045
H	-1.47690553	2.51656233	1.09321839
H	-2.75549597	1.68789171	0.06690783
C	-1.54754893	0.33424732	1.31206377
C	-2.41413647	-0.83280196	0.86121644
H	-2.01630863	-1.76795218	1.25860412
H	-3.43930204	-0.71765746	1.21692240
Au	-0.12584837	-0.17406299	-2.81558081
P	0.97789904	-0.32594262	-4.85076728
C	0.15235664	0.58223848	-6.18045303
C	2.66719490	0.32060722	-4.80708406
C	1.13217261	-2.02377790	-5.45738411
H	0.13799564	-2.45669423	-5.58268129
H	1.65720408	-2.03238300	-6.41537858
H	1.68842974	-2.62055059	-4.73218178
H	-0.85744105	0.19065500	-6.31617786
H	0.71401945	0.47291351	-7.11107867
H	0.08908693	1.63909665	-5.91511192
H	2.64632925	1.37341316	-4.51964300
H	3.13223957	0.22022277	-5.79053502
H	3.25009531	-0.23509343	-4.07024265
C	-0.87432547	0.28290424	2.64124895
C	-1.15951831	1.21688055	3.62668306
C	0.04079824	-0.73035548	2.90730427
C	-0.52680494	1.15545392	4.86840058
H	-1.88253553	2.00260268	3.44689152
C	0.66558100	-0.81694365	4.14670463
H	0.28487034	-1.46072736	2.14528296
C	0.38079995	0.13537082	5.12086616
N	1.04665435	0.05853224	6.43123277
O	1.42895889	1.10066456	6.91973834
O	1.16550070	-1.04214259	6.92665318
C	-0.88239640	2.13810837	5.85999607
N	-1.22418245	2.93930768	6.60783208
C	1.62669705	-1.87081803	4.34947373
N	2.39293855	-2.72127369	4.43666768

*

G2

C	-2.05892685	-1.13433410	0.15317777
H	-1.87384561	-2.08462168	-0.34984647
H	-3.01627849	-0.77154626	-0.24962140
C	-1.03364743	-0.12214355	-0.22543313
C	-0.74640789	0.70354396	0.86995469
H	0.11920614	1.34841398	0.91773263
C	-2.01188645	1.24397130	1.63226082
H	-1.79411282	2.19775957	2.09569646
H	-2.95282385	1.17617363	1.10113121
C	-1.39595113	0.04779570	2.16647142
C	-2.03427807	-1.24679809	1.68530003
H	-1.42024209	-2.09709111	1.98545194
H	-3.02774532	-1.37252195	2.12031588
Au	-0.14930843	-0.00851896	-2.05334135
P	0.85324862	0.11699970	-4.14057912
C	0.09666039	1.35255698	-5.22529288
C	2.60824996	0.55336492	-4.07424923
C	0.78504112	-1.43480889	-5.06903051
H	-0.25705216	-1.72528210	-5.21490080
H	1.26917026	-1.31297713	-6.04079095
H	1.29373841	-2.21839969	-4.50439833
H	-0.95660484	1.10848855	-5.37553437
H	0.60907826	1.36646732	-6.19004860
H	0.16809666	2.33759724	-4.76048035
H	2.72343349	1.52444425	-3.58925816
H	3.02115763	0.59897256	-5.08468037
H	3.14797650	-0.19709053	-3.49369901
C	-0.60334321	0.06748650	3.42895220
C	0.49508469	-0.78102059	3.56962374
C	-0.95298106	0.90357929	4.48518408
C	1.23733092	-0.79367520	4.73982921
H	0.78103232	-1.43333994	2.75132068
C	-0.22035774	0.90072843	5.66635271
H	-1.81062665	1.56063572	4.39503659
C	0.86735900	0.05131045	5.77700470
H	2.09407764	-1.44776009	4.84652310
H	-0.49517964	1.54933105	6.48924421
Cl	1.79648679	0.04099880	7.25442863

*

G20

C	-2.39558505	-0.72995786	-1.43689296
H	-2.39938180	-1.72873100	-1.87683948
H	-3.20305650	-0.18590529	-1.94925852
C	-1.13573588	-0.01332225	-1.76885489
C	-0.72758774	0.76611976	-0.66922333
H	0.26662357	1.17702006	-0.56886401
C	-1.87435717	1.66601927	-0.07939749
H	-1.46431203	2.56683953	0.35926980
H	-2.75671677	1.79369333	-0.69293012
C	-1.62387269	0.38976909	0.56650775
C	-2.52695121	-0.74059213	0.09456636
H	-2.17094879	-1.69219457	0.49264316
H	-3.55231983	-0.58844715	0.43587703
Au	-0.13835161	-0.18469425	-3.53024835
P	1.00290811	-0.39353313	-5.53927503
C	0.22186938	0.50720980	-6.90057246
C	2.70360079	0.22038302	-5.47401427
C	1.13543354	-2.10416551	-6.11436852
H	0.13548752	-2.51967626	-6.25223665
H	1.67890727	-2.13929345	-7.06141347
H	1.66526656	-2.69928589	-5.36830767
H	-0.79319704	0.13419315	-7.04875189
H	0.79860220	0.36963781	-7.81816467
H	0.17571524	1.56976047	-6.65512959
H	2.69836006	1.27781932	-5.20336568
H	3.18465254	0.09531650	-6.44689943
H	3.26166857	-0.33479923	-4.71778091
C	-0.97212210	0.30755755	1.90489372
C	-1.23992699	1.24535568	2.89118387
C	-0.09536904	-0.73651491	2.17860773
C	-0.63833914	1.15501890	4.14040640
H	-1.93173946	2.05363483	2.69268336
C	0.51472132	-0.84690048	3.41747559
H	0.12524460	-1.46626060	1.40960637
C	0.23527074	0.10682792	4.39023999
C	-0.96114486	2.21038489	5.18000294
C	1.46506279	-2.00291778	3.66025530
N	0.88327020	0.00049068	5.71750640
F	-2.13028696	2.81414184	4.91382426
F	-0.02284556	3.17247393	5.22446113
F	-1.06114647	1.68884236	6.41712263
F	1.94487196	-2.49005579	2.50462058
F	0.86005988	-3.02225567	4.29509893
F	2.52664783	-1.63908079	4.40365037
O	1.68648506	0.85977006	6.00753732
O	0.56134430	-0.93838408	6.41223118

*

G21

C	-1.86225898	-1.45769686	-0.48756268
H	-1.51899544	-2.37315894	-0.97299803
H	-2.83452216	-1.23381045	-0.95090402
C	-0.96699186	-0.31922348	-0.82410384
C	-0.81468788	0.51365589	0.30117652
H	-0.03584579	1.25713860	0.39042108
C	-2.18848047	0.91578141	0.95604304
H	-2.11721163	1.89055587	1.42153544
H	-3.07931484	0.73062082	0.36989811
C	-1.46364685	-0.19852724	1.54083300
C	-1.91235076	-1.56540964	1.04494663
H	-1.21630044	-2.33222185	1.38839348
H	-2.90661856	-1.80983052	1.42267711
Au	-0.08370629	-0.04192346	-2.63237800
P	0.92032110	0.26857337	-4.70065620
C	-0.18624027	1.01334882	-5.92367203
C	2.36969661	1.35027165	-4.64674475
C	1.50105041	-1.26902227	-5.45752135
H	0.65743403	-1.94843915	-5.59252162
H	1.95944665	-1.05937987	-6.42676272
H	2.23456333	-1.74289706	-4.80258006
H	-1.05784574	0.37161841	-6.06444642
H	0.33636453	1.13270897	-6.87560547
H	-0.51990484	1.98919534	-5.56613741
H	2.07767853	2.33064749	-4.26584528
H	2.79390816	1.45934823	-5.64753769
H	3.11871927	0.92097100	-3.97867131
C	-0.78141691	-0.08063936	2.86259470
C	0.43666154	-0.72962933	3.05840797
C	-1.36803862	0.64402023	3.89405122
C	1.05964285	-0.63297423	4.28206610
H	0.90674687	-1.30215657	2.26792115
C	-0.73176044	0.70679199	5.11776886
H	-2.31340062	1.15592757	3.76454178
C	0.48668911	0.07941605	5.32959085
N	1.15675215	0.16680656	6.62834126
O	0.50758279	-0.12888481	7.61059399
O	2.31543989	0.52939558	6.63304383
F	-1.28493599	1.41171995	6.11032517
F	2.22499315	-1.25890383	4.48163493

*

G22

C	-2.46968395	-1.20405635	-0.92278515
H	-2.16480027	-2.09621823	-1.46818388
H	-3.43578462	-0.89351594	-1.34518365
C	-1.51177667	-0.06525824	-1.11441351
C	-1.56938732	0.82677457	-0.06436560
H	-0.82219922	1.58747259	0.11349823
C	-2.90163738	1.01201848	0.72321483
H	-2.88743008	1.94082438	1.27879698
H	-3.79723490	0.82538941	0.14148446
C	-2.16194115	-0.11029895	1.23277202
C	-2.52886627	-1.43678264	0.59198810
H	-1.80950762	-2.20480508	0.87485805
H	-3.51447043	-1.75117047	0.94515434
Au	-0.19789983	0.06103540	-2.67234543
P	1.28730864	0.19347424	-4.44376778
C	0.77452995	1.35854037	-5.73133493
C	2.94630949	0.73146165	-3.95818147
C	1.53744586	-1.37902163	-5.30527474
H	0.58296950	-1.73317862	-5.69926464
H	2.24556506	-1.24994710	-6.12714255
H	1.92540554	-2.12001353	-4.60381643
H	-0.19373461	1.05411104	-6.13297201
H	1.51322624	1.37767785	-6.53614793
H	0.67916135	2.35703255	-5.30062705
H	2.88703248	1.71894325	-3.49666426
H	3.59837703	0.77671491	-4.83357250
H	3.35908936	0.02794456	-3.23270869
C	-1.39218393	-0.05204480	2.48839710
C	-0.27699244	-0.87572613	2.68154807
C	-1.74557152	0.81264649	3.52789881
C	0.45731503	-0.84156210	3.84859093
H	0.04019199	-1.54367267	1.88714955
C	-1.03172709	0.85579566	4.71079306
H	-2.61545278	1.45237199	3.42438435
C	0.09588243	0.02805583	4.90398503
H	1.32504209	-1.48095780	3.94740306
H	-1.35547998	1.52328820	5.49892638
N	0.80713256	0.06421370	6.05771812
C	0.53511208	0.99703132	7.14900869
C	1.91126083	-0.84458893	6.35972230
H	0.43262440	2.01795521	6.77242567
H	-0.39523015	0.73017041	7.66614546
C	1.74897863	0.83420726	8.06243870
H	1.63949269	-1.87786273	6.12939979
H	2.79980768	-0.58672736	5.76996796
C	2.14890796	-0.62749872	7.85351835
H	2.55406009	1.49394756	7.72684832
H	1.51786459	1.07357934	9.10115481
H	1.48887743	-1.28140579	8.43034186
H	3.17966818	-0.83780623	8.14158302

*

G23

C	-1.21595730	2.17927043	-0.42957161
H	-2.14142329	1.91863950	-0.94452933
H	-0.88424431	3.12703032	-0.87922532
C	-0.14286258	1.18299383	-0.70961877
C	0.68243240	1.03447569	0.40969327
H	1.37597230	0.21510317	0.53258541
C	1.11331694	2.37265159	1.11259414
H	2.06343030	2.24500922	1.61524155
H	1.00382832	3.27304070	0.52136646
C	-0.05756193	1.72153691	1.65508128
C	-1.37272175	2.24394684	1.09699590
H	-2.19303288	1.59794773	1.41294065
H	-1.57162369	3.25260193	1.46488708
Au	0.03648893	0.14802574	-2.45232842
P	0.23784096	-1.02254489	-4.44350677
C	1.24296283	-0.16134301	-5.67825379
C	1.00926379	-2.64864607	-4.25408012
C	-1.34541867	-1.34166591	-5.26005699
H	-1.83875050	-0.39309256	-5.48002804
H	-1.18370720	-1.89297102	-6.18922832
H	-1.98517565	-1.92634232	-4.59641311
H	0.79350207	0.80802849	-5.90147554
H	1.30158300	-0.75546455	-6.59319420
H	2.24760936	-0.00182113	-5.28233864
H	2.01029626	-2.52925102	-3.83539562
H	1.07672026	-3.14742100	-5.22368719
H	0.41217599	-3.25703391	-3.57226795
C	-0.02388418	1.00823224	2.96073299
C	-0.84528833	-0.10337172	3.16505641
C	0.78125810	1.43600244	4.00698998
C	-0.83073376	-0.74449740	4.37922508
H	-1.50621031	-0.46883327	2.38674968
C	0.79047500	0.76392525	5.22603590
H	1.41388697	2.30737745	3.88094677
C	-0.01089225	-0.35723314	5.45094494
F	-1.68100122	-1.78875370	4.55170350
H	1.43637967	1.12430128	6.01567742
N	-0.06636235	-1.04785764	6.67255754
C	0.31415284	-2.46101234	6.60269942
C	0.57484295	-0.39017004	7.80193163
H	1.39901454	-2.57584431	6.46420178
H	0.03100849	-2.94879248	7.53754256
H	-0.19739615	-2.96335856	5.78505200
H	0.31963073	-0.94368415	8.70744890
H	1.67098429	-0.36285623	7.71546221

H	0.20314713	0.63049115	7.90635149

*

G24

C	-2.40273577	-0.23747466	-0.88009418
H	-2.62589845	-1.20204126	-1.33967251
H	-3.09724465	0.47403235	-1.35061414
C	-1.03158658	0.21130382	-1.24381637
C	-0.43329370	0.85279749	-0.14469109
H	0.62704645	1.04792957	-0.07449577
C	-1.35005365	1.95650070	0.50342178
H	-0.74612291	2.73988142	0.94370266
H	-2.20212581	2.28218622	-0.07937738
C	-1.35108262	0.64149046	1.11764375
C	-2.48300442	-0.26348645	0.65444151
H	-2.31778745	-1.27843487	1.01987012
H	-3.44334658	0.08665001	1.03723129
Au	-0.16372285	-0.09288893	-3.05588295
P	0.81412528	-0.42959428	-5.13223063
C	0.48121067	0.91559936	-6.29635805
C	2.61699518	-0.57117904	-5.07112723
C	0.24575122	-1.93242713	-5.96470267
H	-0.83550659	-1.88273954	-6.10575859
H	0.73612684	-2.03146771	-6.93587824
H	0.48155287	-2.80128845	-5.34743379
H	-0.59621945	1.01176851	-6.44251919
H	0.96218868	0.70777851	-7.25502272
H	0.86766731	1.85205619	-5.88996542
H	3.03887887	0.34181261	-4.64688668
H	3.01509537	-0.72390436	-6.07696466
H	2.89456041	-1.41563187	-4.43753100
C	-0.69907470	0.39547371	2.43626247
C	-0.02798033	-0.80172030	2.66388387
C	-0.77945873	1.33320128	3.45914599
C	0.55982290	-1.04760212	3.89640776
H	0.04015351	-1.54045323	1.87178120
C	-0.19356987	1.07136407	4.69122543
H	-1.30826407	2.26522619	3.29642824
C	0.48416141	-0.11618842	4.92232468
H	0.94172194	-0.31314675	5.88262098
C	-0.27366509	2.11870014	5.77086513
F	0.62865849	3.10748747	5.57619700
F	-0.03913820	1.61527208	6.99584147
F	-1.48523449	2.70984662	5.81101162
C	1.27810939	-2.35625331	4.09677874
F	1.84770128	-2.45678683	5.30942433
F	2.25811296	-2.53314608	3.18351527
F	0.44170267	-3.40978410	3.96550754

*

G3

C	-2.14224734	0.93684242	-0.33214281
H	-2.83997162	0.40002318	-0.97676475
H	-2.25196382	1.99741170	-0.60329860
C	-0.73489740	0.56853092	-0.64878136
C	0.02804951	0.54036156	0.52865616
H	1.00256672	0.07865320	0.59538116
C	-0.16518700	1.80502079	1.44553781
H	0.73444735	1.99927258	2.01552796
H	-0.63198501	2.66509844	0.98275552
C	-0.95986050	0.63582342	1.76375561
C	-2.35749394	0.65424495	1.16289483
H	-2.83053317	-0.32038192	1.29259309
H	-2.98026705	1.40472602	1.65349079
Au	-0.04703778	0.13424257	-2.51225760
P	0.74096429	-0.34954577	-4.63908830
C	0.86495863	1.10982642	-5.70239021
C	2.39444435	-1.08496696	-4.64973675
C	-0.30228718	-1.51407860	-5.55052382
H	-1.30661158	-1.09897664	-5.65317374
H	0.11937806	-1.69806940	-6.54143093
H	-0.36375986	-2.45498398	-5.00058261
H	-0.12000943	1.56807585	-5.80872679
H	1.24222805	0.82337171	-6.68684864
H	1.54421157	1.83382753	-5.24856075
H	3.10020888	-0.39831663	-4.17861608
H	2.70969683	-1.28362384	-5.67672133
H	2.38147249	-2.01939125	-4.08555775
C	-0.67290910	-0.20748460	2.95996938
C	-0.87914463	-1.58675263	2.90432462
C	-0.22809553	0.36510730	4.14469165
C	-0.63842868	-2.38179940	4.01386830
H	-1.22388670	-2.03818030	1.97982880
C	0.00571844	-0.43730124	5.25696421
H	-0.07500634	1.43614909	4.20638143
C	-0.19223310	-1.80924121	5.20009948
H	-0.79651202	-3.45275208	3.95915631
C	0.52033736	0.20026337	6.51873480
H	-0.00509619	-2.42779368	6.06941634
F	1.86357718	0.37276194	6.48562379
F	0.25552221	-0.53513849	7.61535827
F	-0.01285311	1.42257070	6.72726847

*

G4

C	-2.02770427	-1.39889371	-0.42202064
H	-1.73051522	-2.35737246	-0.85086327
H	-2.97152433	-1.13685832	-0.92280383
C	-1.05260979	-0.33351614	-0.78204748
C	-0.89998494	0.55704194	0.29256464
H	-0.08670533	1.26424304	0.36875268
C	-2.26455207	1.05528589	0.89960026
H	-2.15150451	2.04750268	1.31747842
H	-3.14758969	0.89011516	0.29563220
C	-1.61817662	-0.06202170	1.55915505
C	-2.12522101	-1.42493220	1.11142441
H	-1.48220852	-2.20984869	1.51239187
H	-3.14135947	-1.59781048	1.47092824
Au	-0.06937152	-0.21899912	-2.55776241
P	1.04524545	-0.08036381	-4.58683075
C	0.16901381	0.92284241	-5.81226262
C	2.69181318	0.65909382	-4.45604205
C	1.30672690	-1.68358199	-5.38428696
H	0.34189928	-2.15946319	-5.56926065
H	1.83468348	-1.54916313	-6.33120679
H	1.89619989	-2.32493678	-4.72648527
H	-0.81544104	0.49033680	-6.00011854
H	0.73744663	0.95431601	-6.74469527
H	0.04189473	1.93678121	-5.42853399
H	2.60544314	1.66985668	-4.05297269
H	3.16440283	0.69802128	-5.44024177
H	3.30617731	0.06107458	-3.78045911
C	-0.95473297	0.08730381	2.88680929
C	0.19696567	-0.65009584	3.17087698
C	-1.47993418	0.93263944	3.85709055
C	0.81466426	-0.53799443	4.40316369
H	0.61476402	-1.30794698	2.41647855
C	-0.86619513	1.04917826	5.09960360
H	-2.37922844	1.50117928	3.64996090
C	0.27768679	0.31398214	5.36669464
H	1.71256428	-1.10715957	4.61773428
H	-1.28478390	1.70794481	5.85022864
C	0.97903765	0.42817247	6.69238089
F	2.20213023	0.99420285	6.56655843
F	1.18038702	-0.78081878	7.26269407
F	0.29795023	1.17120562	7.58348875

*

G5

C	-1.10485827	2.03404891	0.53452922
H	-2.04474591	1.89234802	0.00034120
H	-0.72486777	3.01604723	0.21545099

C	-0.08238243	1.03107101	0.11640009
C	0.75682645	0.71769430	1.18697921
H	1.41611512	-0.13837171	1.19439929
C	1.23644878	1.92554323	2.06875765
H	2.18240988	1.69391227	2.54131597
H	1.16241365	2.90210689	1.60638355
C	0.04318406	1.25638157	2.53064917
C	-1.25121958	1.90035576	2.05731191
H	-2.09604490	1.25147403	2.29178722
H	-1.40731991	2.85775006	2.55908588
Au	0.00937784	0.18953778	-1.73492983
P	0.11272465	-0.76256983	-3.84606957
C	1.37306426	-0.01614873	-4.90942506
C	0.49986452	-2.53044350	-3.82127686
C	-1.43304071	-0.62942099	-4.77788504
H	-1.69364370	0.42354092	-4.90026188
H	-1.32150789	-1.09397784	-5.76036725
H	-2.23273953	-1.12866795	-4.22759190
H	1.16031710	1.04714386	-5.03449651
H	1.37552816	-0.50497581	-5.88644684
H	2.35410506	-0.12746883	-4.44394533
H	1.46590908	-2.68411513	-3.33684777
H	0.53703198	-2.92059077	-4.84103679
H	-0.26696235	-3.06272164	-3.25541991
C	0.05207921	0.36335344	3.72058607
C	-0.80618819	-0.73779637	3.77732001
C	0.89074662	0.60910708	4.80400803
C	-0.82428459	-1.57420687	4.87892682
H	-1.46213299	-0.95057711	2.93924401
C	0.88081045	-0.21901415	5.91921421
H	1.55707159	1.46467146	4.78828970
C	0.02263190	-1.31341078	5.95613310
H	-1.48546422	-2.43264183	4.91815435
H	1.53494507	-0.01440442	6.76140170
O	-0.03175732	-2.16200211	7.01921988
H	0.60003588	-1.87149900	7.69217677

*

G6

C	-2.20445978	-0.95606251	0.10989702
H	-2.11035713	-1.89349967	-0.43873082
H	-3.14258461	-0.49829418	-0.23790216
C	-1.11369384	-0.00145251	-0.24525110
C	-0.78981452	0.79670152	0.85297245
H	0.10886505	1.39402145	0.90892113
C	-1.99767386	1.34291859	1.69541756
H	-1.72123049	2.26098442	2.19805838
H	-2.95643909	1.34566480	1.19184147
C	-1.43309858	0.09786954	2.15924447
C	-2.14336105	-1.14007476	1.63306746
H	-1.56308621	-2.03160671	1.87470676
H	-3.12859050	-1.23967376	2.09367695
Au	-0.18339976	0.04774825	-2.05531899
P	0.86936771	0.09030604	-4.12020708
C	0.21007025	1.35268614	-5.23731809
C	2.64506165	0.42592580	-4.02095765
C	0.73218442	-1.46671967	-5.03271110
H	-0.32155355	-1.69815391	-5.19914643
H	1.24399147	-1.38555723	-5.99447597
H	1.18155889	-2.27145609	-4.44786307
H	-0.85231689	1.16868225	-5.40751861
H	0.74258717	1.32343920	-6.19080355
H	0.32925750	2.33832783	-4.78365965
H	2.80564600	1.39604860	-3.54703160
H	3.08203401	0.43222235	-5.02223419
H	3.12772370	-0.34536814	-3.41787695
C	-0.59441167	0.02250553	3.38519390
C	0.44964401	-0.90614147	3.46755245
C	-0.83609314	0.84307591	4.47898766
C	1.22496984	-1.00303337	4.60451236
H	0.66167706	-1.55276753	2.62199147
C	-0.06701276	0.75538771	5.63714019
H	-1.64693042	1.56273348	4.44384861
C	0.97022309	-0.17090076	5.70167593
H	2.03859580	-1.71722433	4.66654885
H	-0.28928082	1.40661840	6.47184859
O	1.78188592	-0.34103335	6.77444620
C	1.54743364	0.48182707	7.91621808
H	2.29201475	0.18687912	8.65441759
H	1.67644775	1.54027959	7.67185541
H	0.54567108	0.31436237	8.32251267

*

G7

C	-0.95710737	2.14415671	0.19464200
H	-1.90651586	2.05716038	-0.33521497
H	-0.49442283	3.06977670	-0.17912968
C	-0.03121544	1.03532150	-0.17261195
C	0.75061869	0.67524532	0.93123057
H	1.32061466	-0.24120648	0.98226059
C	1.36847894	1.88059888	1.73098483
H	2.28838229	1.57909055	2.21545940
H	1.39226254	2.83063896	1.21218909
C	0.11386603	1.35953925	2.22733972
C	-1.11156066	2.10670648	1.72247667
H	-2.01614498	1.55902808	1.99057209
H	-1.16829163	3.10047176	2.17124086
Au	0.02503258	0.14488225	-2.00093698
P	0.08426268	-0.86264786	-4.08844051
C	1.32194015	-0.14352209	-5.19616112
C	0.46996949	-2.62979093	-4.02874122
C	-1.48097980	-0.74892640	-4.98981163
H	-1.74242108	0.30132861	-5.13197416
H	-1.39065735	-1.23722328	-5.96291357
H	-2.26966239	-1.23333383	-4.41111282
H	1.10696720	0.91661853	-5.34234582
H	1.30277011	-0.65598223	-6.16081166
H	2.31284396	-0.24459726	-4.74968541
H	1.44596390	-2.77321514	-3.56143671
H	0.48491483	-3.04449230	-5.03933806
H	-0.28521377	-3.14697065	-3.43396811
C	0.03745418	0.54919550	3.47552102
C	-0.90051022	-0.47534278	3.58609932
C	0.87792484	0.80968114	4.55707128
C	-0.99048343	-1.22224830	4.75038705
H	-1.55727128	-0.69680717	2.75092247
C	0.78420961	0.06490642	5.72298654
H	1.60800733	1.60899694	4.49030812
C	-0.15315264	-0.96410716	5.83943862
H	-1.72062089	-2.02229090	4.82153331
H	1.45078470	0.29139678	6.54724003
C	-0.29760996	-1.78774008	7.05853007
C	0.36342363	-1.62934710	8.20210751
H	-1.02832521	-2.58977942	6.98097139
H	0.17791584	-2.29309925	9.03997495
H	1.10026726	-0.84619847	8.35001760

*

G8

C	-2.02336469	-1.14998505	0.27818362
H	-1.83630578	-2.10063805	-0.22362008
H	-2.98153998	-0.79036560	-0.12540130
C	-1.00020390	-0.13717425	-0.10354886
C	-0.70716450	0.68567351	0.99314153
H	0.15889267	1.33006912	1.03887408
C	-1.97346833	1.23093554	1.75171633
H	-1.75446185	2.18545298	2.21304351
H	-2.91402221	1.16338780	1.21994543
C	-1.35843205	0.03502992	2.28869931
C	-1.99790664	-1.26017726	1.81055681
H	-1.38412519	-2.11017075	2.11202498
H	-2.99131406	-1.38445219	2.24602602

Au	-0.13109761	-0.01557596	-1.93803307
P	0.85145227	0.12134425	-4.03409974
C	0.10019197	1.38045496	-5.09514263
C	2.61269365	0.53369725	-3.98061990
C	0.75285802	-1.41792535	-4.98038794
H	-0.29452799	-1.69244311	-5.11906493
H	1.22880835	-1.29100169	-5.95552491
H	1.25640498	-2.21504332	-4.43026956
H	-0.95783538	1.15250254	-5.23695029
H	0.60238663	1.39974745	-6.06516200
H	0.19000505	2.35845012	-4.61876185
H	2.74552953	1.49726294	-3.48525936
H	3.01597869	0.58609052	-4.99459445
H	3.14821830	-0.23074685	-3.41462206
C	-0.56886786	0.05638088	3.55325176
C	0.53123471	-0.79011515	3.69677878
C	-0.92467131	0.89196514	4.60833001
C	1.26618600	-0.79871786	4.86941876
H	0.81948317	-1.44081240	2.87792090
C	-0.19513065	0.88737943	5.78890923
H	-1.78386151	1.54631966	4.51300106
C	0.90643537	0.04251860	5.92725250
H	2.12364796	-1.45344434	4.97327995
H	-0.47949739	1.53729432	6.60832997
C	1.66701076	0.03576347	7.15021033
C	2.30007693	0.03022122	8.17056746
H	2.86410366	0.02537696	9.07910948

*

G9

C	-1.68248567	-1.64834589	0.40246233
H	-1.27447435	-2.57090500	-0.01357568
H	-2.69018669	-1.55541748	-0.02933325
C	-0.91330650	-0.46628386	-0.07334247
C	-0.82652440	0.49086946	0.95057145
H	-0.12942350	1.31623064	0.93530741
C	-2.20027199	0.80290497	1.65189397
H	-2.21256199	1.81962084	2.02320250
H	-3.09093511	0.47463731	1.13159104
C	-1.33884065	-0.17016453	2.29390573
C	-1.66004855	-1.61478315	1.93892770
H	-0.87502557	-2.27304086	2.31396101
H	-2.60715006	-1.92232059	2.38611697
Au	-0.07541613	-0.29270662	-1.91734381
P	0.88478820	-0.08326705	-4.01825515
C	0.35447213	1.40260534	-4.90517682
C	2.69137198	0.01309500	-3.96690168
C	0.51360447	-1.46006249	-5.13219237
H	-0.56652096	-1.53350904	-5.27187370
H	0.99629548	-1.30086322	-6.09921229
H	0.87659439	-2.39095845	-4.69255795
H	-0.72841589	1.37889529	-5.04033125
H	0.84258138	1.44955689	-5.88138671
H	0.61844069	2.28718286	-4.32267864
H	2.99293659	0.87474569	-3.36845766
H	3.08928876	0.11487233	-4.97923500
H	3.09123861	-0.89288149	-3.50776421
C	-0.60043496	0.14303449	3.55175277
C	0.66377941	-0.40975853	3.76279888
C	-1.16383459	0.96256396	4.52627262
C	1.36156359	-0.14493748	4.92722659
H	1.10746170	-1.04406834	3.00331270
C	-0.47637846	1.23503538	5.69965863
H	-2.14953195	1.38626343	4.37339488
C	0.78636622	0.68071587	5.89540721
H	2.34591424	-0.56708716	5.09087357
H	-0.91350841	1.87054105	6.46047454
C	1.50785041	0.96350105	7.10947947
N	2.08522115	1.18936268	8.07820481

*

TSAB0

C	-0.24505336	-4.67608220	0.08929035
C	1.01572806	-4.19614479	-0.24507971
C	1.23785186	-2.83042447	-0.29604568
C	0.19406801	-1.94057759	-0.02122453
C	-1.06985154	-2.43099241	0.32789784
C	-1.28469634	-3.79569827	0.38041110
H	-0.41892115	-5.74503025	0.13566099
H	1.82299927	-4.88538236	-0.46064252
H	2.22076257	-2.44885870	-0.55299210
H	-1.87716720	-1.75545289	0.58698708
H	-2.25851673	-4.17909783	0.65988809
C	0.48122286	-0.51131230	-0.09336455
H	1.52765393	-0.21650503	-0.05341212
H	0.08675746	0.00976251	-1.23621370
H	-1.50449403	0.23115643	-0.26397722
C	-0.18864397	1.95468424	-0.03933923
C	-1.18353141	2.84628733	-0.45420720
C	1.01394306	2.44284966	0.48535948
C	-0.97048288	4.21163806	-0.36742175
H	-2.12078150	2.46636466	-0.84782626
C	1.21951790	3.80720251	0.57275677
H	1.77680596	1.76581016	0.85259464
C	0.23112220	4.68941359	0.14211066
H	-1.73945310	4.90227976	-0.69132200
H	2.14457575	4.18883519	0.98755586
H	0.39697472	5.75805719	0.21647273
C	-0.46277511	0.52587566	-0.15689670

*

TSAB1

C	-0.11271710	-4.22336116	0.05107791
C	1.11871779	-3.69267271	-0.33081027
C	1.31153134	-2.32376847	-0.38926835
C	0.26697886	-1.45121135	-0.07356406
C	-0.96986098	-1.97600670	0.32157248
C	-1.15050849	-3.34217491	0.37906984
H	1.93491616	-4.36036088	-0.58190354
H	2.27720434	-1.92717657	-0.68657419
H	-1.78572457	-1.32304576	0.61121870
H	-2.10782933	-3.74253995	0.69435536
C	0.52111900	-0.01555859	-0.16013437
H	1.56194959	0.30039154	-0.15009327
H	0.09389873	0.47338444	-1.27259429
H	-1.48504723	0.68645398	-0.22931953
C	-0.20172580	2.43268264	-0.04260991
C	-1.24376117	3.30247731	-0.38508805
C	1.01956261	2.94875636	0.41020087
C	-1.06085998	4.67185405	-0.29852948
H	-2.19378290	2.90089324	-0.72249655
C	1.19510318	4.31675912	0.49735115
H	1.82113365	2.28852789	0.72081967
C	0.15840891	5.17609174	0.13874655
H	-1.86592376	5.34515688	-0.56629425
H	2.13449413	4.71972112	0.85590370
H	0.30138289	6.24810285	0.21284803
C	-0.44695126	1.00303271	-0.15366746
C	-0.32474104	-5.70783921	0.14236478
H	-0.37234986	-6.01532220	1.19068860
H	-1.26759049	-5.99479099	-0.32739883
H	0.48610877	-6.25691042	-0.33609824

*

TSAB10

C	-0.72160334	-4.21653759	0.25088665
C	0.57624647	-3.86004311	-0.12262380
C	0.87648503	-2.50821680	-0.20929408
C	-0.09260984	-1.53448527	0.05997205
C	-1.38091503	-1.91292177	0.44455165
C	-1.68768590	-3.25951480	0.53652727
H	1.87857340	-2.20134621	-0.49430991

H -2.13102210 -1.17379774 0.70100220
H -2.67974769 -3.56814961 0.84362861
C 0.30193699 -0.13314788 -0.05880785
H 1.36757019 0.08379344 -0.03244754
H -0.05811791 0.37298217 -1.20955055
H -1.62507308 0.74759920 -0.23278636
C -0.18610899 2.37574286 -0.06668386
C -1.12193598 3.32585937 -0.48963133
C 1.05792643 2.78994390 0.42467798
C -0.81021131 4.67409480 -0.44376624
H -2.09105727 3.00423010 -0.85710567
C 1.36233134 4.13758584 0.47089555
H 1.77690305 2.06927630 0.79724914
C 0.43172809 5.07713960 0.03256237
H -1.53356205 5.40968192 -0.77374065
H 2.31991056 4.46241789 0.85942616
H 0.67501080 6.13264569 0.07473360
C -0.56408877 0.96900119 -0.13908782
H -0.97669392 -5.26835707 0.33144009
C 1.60856006 -4.91664274 -0.41241557
H 2.56541738 -4.47360108 -0.69000872
H 1.76592909 -5.54935441 0.46391910
H 1.27963730 -5.56103818 -1.23072044
*

TSAB11
C -0.70913852 -4.24300776 0.16390412
C 0.58260219 -3.85660754 -0.18969380
C 0.89700590 -2.50370352 -0.25592488
C -0.08286488 -1.55002790 0.02150631
C -1.37640631 -1.93746759 0.38705115
C -1.67494473 -3.28689658 0.45399814
H 1.90585311 -2.21423437 -0.52870710
H -2.13119625 -1.20657852 0.64933140
H -2.66793815 -3.60664106 0.74603983
C 0.30792884 -0.14668444 -0.06834933
H 1.37328194 0.07213535 -0.04573667
H -0.07027436 0.39967011 -1.22389841
H -1.62132262 0.73004373 -0.24110625
C -0.18263140 2.36192341 -0.05641515
C -1.11013107 3.31671130 -0.48454006
C 1.05264430 2.76883590 0.45995261
C -0.79806117 4.66429781 -0.41838902
H -2.07323316 3.00043806 -0.87201382
C 1.35742460 4.11602979 0.52663419
H 1.76502363 2.04374643 0.83688105
C 0.43568415 5.06140818 0.08328182
H -1.51540564 5.40389211 -0.75257702
H 2.30867112 4.43500368 0.93510692
H 0.67895284 6.11612655 0.14126216
C -0.56014645 0.95236870 -0.15308662
H -0.95882397 -5.29773549 0.22491379
O 1.57125386 -4.73190195 -0.47553726
H 1.25754606 -5.63985148 -0.39661685
*

TSAB12
C -0.27857583 -3.42739433 0.12573938
C 0.80906740 -2.92808982 -0.59970669
C 1.01277020 -1.56869653 -0.72056420
C 0.12893288 -0.66147844 -0.12508240
C -0.95371733 -1.15147183 0.61268038
C -1.14916456 -2.51221025 0.73147681
H 1.50685131 -3.60931968 -1.07169537
H 1.86384962 -1.20125129 -1.28541149
H -1.63261662 -0.47656696 1.12235511
H -1.98397900 -2.88325741 1.31505552
C 0.38672364 0.76623744 -0.29445383
H 1.39903834 1.05344901 -0.57007231
H -0.30617952 1.25289020 -1.24786943
H -1.53858590 1.52273106 0.19890962
C -0.20796137 3.23140502 0.01960766
C -1.28138830 4.13025785 -0.01419328
C 1.10514921 3.71416989 0.10533374
C -1.04564544 5.49377972 0.01355435
H -2.29823596 3.75489553 -0.06653121
C 1.33389949 5.07646465 0.13554689
H 1.94406544 3.03160940 0.17656522
C 0.26059739 5.96395050 0.08465445
H -1.87550917 6.18918872 -0.01568320
H 2.34659164 5.45350955 0.21091454
H 0.44674896 7.03154495 0.11241271
C -0.51116429 1.81154238 -0.01251342
C -0.52371291 -4.87633949 0.28137225
C -0.18909645 -5.83289886 -0.84295004
C 0.63808190 -5.83997244 0.39565435
H -1.40234356 -5.11706045 0.86726928
H 0.23415023 -5.40710948 -1.74458914
H -0.87760484 -6.65125341 -1.00904036
H 0.52342274 -6.66318148 1.08882103
H 1.63505067 -5.41849898 0.35373836
*

TSAB13
C -0.97529506 -3.86094106 0.27770594
C 0.35339023 -3.53456287 0.01048125
C 0.73274236 -2.20595719 -0.07435040
C -0.21735056 -1.19323923 0.09765677
C -1.54687843 -1.53081558 0.38003079
C -1.92132498 -2.86121066 0.46717262
H 1.76985006 -1.95376215 -0.27925085
H -2.28756967 -0.75999601 0.56023971
H -2.94834244 -3.11968203 0.69435725
C 0.23081660 0.19006305 -0.00225954
H 1.30094884 0.36788404 0.08080334
H -0.05306807 0.72936466 -1.19334295
H -1.64497825 1.14614650 -0.31667782
C -0.14982475 2.71675348 -0.07530144
C -1.00534245 3.69948944 -0.58327828
C 1.06643019 3.08307116 0.51220004
C -0.63922581 5.03370778 -0.52536853
H -1.95453335 3.41549888 -1.02617620
C 1.42504291 4.41722962 0.57006739
H 1.72039403 2.33817004 0.95138844
C 0.57599581 5.38992051 0.04733090
H -1.30039750 5.79489169 -0.92118358
H 2.36079091 4.70549737 1.03343841
H 0.86102591 6.43445659 0.09887551
C -0.58201079 1.32292111 -0.16731011
H -1.24568841 -4.90899891 0.34481352
C 1.36580610 -4.62213019 -0.17818216
O 1.08625529 -5.78820218 -0.12290401
H 2.40133946 -4.28106312 -0.37452604
*

TSAB14
C 0.00127179 -3.44361641 0.01769597
C 1.24438747 -2.93939921 -0.31873079
C 1.40063009 -1.56440646 -0.35813867
C 0.31793316 -0.72417831 -0.07248565
C -0.92434191 -1.26885748 0.27895781
C -1.08670732 -2.64036127 0.32326982
H 2.05949032 -3.61561980 -0.53796196
H 2.36528851 -1.14067543 -0.61562500
H -1.75890086 -0.63167372 0.54547103
H -2.02844587 -3.09590057 0.59785874
C 0.54378393 0.71294747 -0.12768259
H 1.57912727 1.04805958 -0.12980164
H 0.04899957 1.28754944 -1.28913769
H -1.47434453 1.38293145 -0.25893610
C -0.21109243 3.15595621 -0.06013955
C -1.22987400 4.01901732 -0.47305032

C	0.97102645	3.67428012	0.47782276
C	-1.06016915	5.38968592	-0.36938251
H	-2.15234140	3.61539492	-0.87804404
C	1.13319831	5.04410437	0.58232641
H	1.75278038	3.01869101	0.84507415
C	0.12163362	5.90007882	0.15428663
H	-1.84861991	6.05887863	-0.69155762
H	2.04285001	5.44888137	1.00915047
H	0.25308991	6.97241154	0.24187470
C	-0.43929771	1.71289972	-0.20064839
N	-0.17520050	-4.92253342	0.06733830
O	-1.27524524	-5.32976500	0.36585721
O	0.79509181	-5.59576582	-0.19641985

*

TSAB15

C	-0.12157022	-3.66652323	0.02724575
C	1.15357806	-3.14682139	-0.17186444
C	1.36212525	-1.78350955	-0.20226189
C	0.27410582	-0.92023783	-0.03617841
C	-1.01291301	-1.42845363	0.17631340
C	-1.19494086	-2.79291124	0.20343259
H	2.36834895	-1.41260690	-0.35897172
H	-1.87367559	-0.79556577	0.35096290
C	0.53845892	0.50995015	-0.07790706
H	1.57523790	0.82049224	0.03120328
H	0.19671965	1.06700259	-1.26615424
H	-1.44076912	1.22538309	-0.40623531
C	-0.16629097	2.96734939	-0.08066711
C	-1.13024174	3.84878809	-0.57974261
C	0.97788158	3.46557615	0.55213016
C	-0.94225729	5.21614485	-0.46735282
H	-2.02384463	3.46107973	-1.05824702
C	1.15832820	4.83202387	0.66411406
H	1.71304007	2.79656679	0.98524193
C	0.20256437	5.70536538	0.15034735
H	-1.68760800	5.89930489	-0.85589186
H	2.03754571	5.22150413	1.16283117
H	0.34807596	6.77536583	0.24440212
C	-0.41144278	1.53234245	-0.23360807
F	-2.39981038	-3.30726081	0.41167719
F	-0.31358996	-4.96767640	0.06322713
F	2.16658191	-3.98811774	-0.32909913

*

TSAB16

C	0.30136825	-3.93917188	-0.03942064
C	1.53313565	-3.34356767	-0.26756110
C	1.63524867	-1.96361838	-0.29488520
C	0.50979922	-1.16322785	-0.09981489
C	-0.73831453	-1.76432507	0.14172414
C	-0.84022821	-3.13467881	0.17218486
H	2.60127269	-1.50398388	-0.47357171
H	-1.62294575	-1.16687146	0.33771178
C	0.68678539	0.28416908	-0.16363815
H	1.70135614	0.65213749	-0.03012732
H	0.38086809	0.73400876	-1.30333395
H	-1.33866225	0.89126293	-0.41791634
C	-0.17319225	2.68785641	-0.07445899
C	-1.22609570	3.51089314	-0.49617408
C	0.97626673	3.25921649	0.49055533
C	-1.12332747	4.88554529	-0.37693218
H	-2.12045461	3.06724813	-0.92158967
C	1.07167601	4.63219290	0.60919461
H	1.78123152	2.63557465	0.86160802
C	0.02599198	5.44328595	0.17169467
H	-1.93562333	5.52194278	-0.70600118
H	1.95406956	5.07699360	1.05290310
H	0.10600868	6.51984719	0.27095341
C	-0.33216892	1.25485371	-0.22212961
O	0.20706580	-5.27329522	-0.00451041
H	2.39812646	-3.97783150	-0.41555615
H	-0.70885106	-5.52922517	0.17475844
O	-1.98369262	-3.83354669	0.41222350
H	-2.73016354	-3.24869551	0.57618203

*

TSAB17

C	-0.59603139	-3.22674575	0.08506432
C	0.75048050	-2.79468447	-0.09134486
C	1.02516407	-1.44503931	-0.11670766
C	-0.01161539	-0.49906005	0.01960721
C	-1.31831596	-0.92738067	0.20385532
C	-1.60767746	-2.28547568	0.23761136
H	2.04344065	-1.10173132	-0.24817683
H	-2.12365889	-0.21922463	0.36075564
H	-2.62702112	-2.60619578	0.39989697
C	0.33987604	0.91737926	-0.02907125
H	1.35376581	1.18940321	0.25707479
H	0.26328164	1.36890357	-1.20548490
H	-1.54805225	1.70104759	-0.61369548
C	-0.29099304	3.39262917	-0.08661655
C	-1.17319447	4.30324466	-0.68334615
C	0.79480585	3.86327239	0.66571684
C	-0.96020245	5.66407386	-0.55147931
H	-2.02114079	3.93755818	-1.25326095
C	1.00057275	5.22316398	0.79596791
H	1.45956700	3.17232471	1.17099078
C	0.12752849	6.12093742	0.18416162
H	-1.64004295	6.36813238	-1.01544110
H	1.83289770	5.59096191	1.38361367
H	0.29248946	7.18682340	0.29346258
C	-0.55813568	1.97847186	-0.25684517
O	-0.77100857	-4.54838495	0.10444861
O	1.65682267	-3.77864866	-0.21601068
C	-2.09430049	-5.05505391	0.28543107
H	-2.49963920	-4.74215681	1.25126230
H	-2.74927356	-4.72560324	-0.52546222
H	-1.99756083	-6.13757677	0.26198148
C	3.02032511	-3.40593374	-0.38139985
H	3.15763257	-2.82271808	-1.29731797
H	3.37774947	-2.83821877	0.48331848
H	3.57483287	-4.33809901	-0.45816998

*

TSAB18

C	0.00780116	-3.38066684	0.09398398
C	1.27242355	-2.81183582	-0.19822318
C	1.42816306	-1.44910860	-0.26917118
C	0.35312350	-0.57450414	-0.05885729
C	-0.89690684	-1.13048898	0.24735963
C	-1.07306702	-2.48969233	0.32504232
H	2.13115417	-3.44561638	-0.36491996
H	2.41048440	-1.04581766	-0.49647985
H	-1.74907960	-0.49307291	0.45929346
H	-2.04855745	-2.87461701	0.58462775
C	0.58525012	0.86084715	-0.17485054
H	1.61280230	1.19679017	-0.05181617
H	0.30794235	1.26163222	-1.31054609
H	-1.42715856	1.53587186	-0.36968901
C	-0.19906147	3.29042676	-0.05928864
C	-1.24596180	4.14522150	-0.43562371
C	0.99185343	3.83134110	0.45089720
C	-1.09787783	5.51606727	-0.32674098
H	-2.17104694	3.72582695	-0.81745959
C	1.13293214	5.20076859	0.55840363
H	1.79359360	3.18431841	0.78656033
C	0.09184598	6.04086721	0.16602876
H	-1.90529399	6.17560273	-0.62036294
H	2.04737927	5.62135140	0.95872468
H	0.20790282	7.11494339	0.25694672
C	-0.40670943	1.86818350	-0.19057654

N	-0.16164262	-4.72014047	0.16429846
C	-1.46916857	-5.28238087	0.47151945
C	0.96791779	-5.61458469	-0.04665305
H	-1.39966795	-6.36709898	0.45445551
H	-1.81192177	-4.97829046	1.46530385
H	-2.21490727	-4.97838207	-0.26864624
H	0.62858679	-6.64235488	0.05470662
H	1.38944087	-5.49221642	-1.04869517
H	1.75609545	-5.44127016	0.69214632

*

TSAB19

C	-0.03736449	-2.81813601	0.01300937
C	1.24360699	-2.31565950	-0.18999389
C	1.42332398	-0.93772799	-0.20839027
C	0.32991232	-0.08013242	-0.05453402
C	-0.94896750	-0.60724868	0.15414783
C	-1.13710320	-1.98126718	0.19517774
H	2.42260517	-0.54215002	-0.35146809
H	-1.80767156	0.03262681	0.31383326
C	0.57770853	1.35167162	-0.08474250
H	1.61380255	1.67184912	0.01033603
H	0.17567980	1.98669590	-1.32792374
H	-1.40927497	2.05281812	-0.42969610
C	-0.14360598	3.80611811	-0.09477728
C	-1.08610427	4.69078396	-0.62526474
C	0.96963521	4.29615927	0.59405625
C	-0.90632516	6.05701142	-0.48550456
H	-1.95672911	4.30906940	-1.14900100
C	1.14127135	5.66191916	0.73365007
H	1.68659564	3.62378798	1.05259880
C	0.20800383	6.54061922	0.18970875
H	-1.63552814	6.74359612	-0.89794825
H	1.99572635	6.04534505	1.27803633
H	0.34606710	7.60941580	0.30526731
C	-0.37678840	2.36467726	-0.28380667
N	-0.23774316	-4.28151523	0.04853261
O	-0.01617313	-4.86328719	-0.98542635
O	-0.60148782	-4.73362846	1.10666510
C	-2.45932249	-2.50719264	0.41147377
N	-3.53489484	-2.87738497	0.56521380
C	2.37950306	-3.18359448	-0.35532227
N	3.31451836	-3.83853156	-0.47481429

*

TSAB2

C	-0.04276950	-3.76311370	0.02343969
C	1.18503425	-3.24686030	-0.37624212
C	1.35167091	-1.87607608	-0.42735024
C	0.29815260	-1.01878369	-0.09041309
C	-0.92639949	-1.55796101	0.32309997
C	-1.09879712	-2.92584705	0.37937521
H	1.99340659	-3.91737017	-0.63745947
H	2.30774745	-1.46587358	-0.73563698
H	-1.74382395	-0.91610759	0.63089587
H	-2.03654075	-3.35527765	0.70753443
C	0.53341113	0.41833000	-0.16815328
H	1.57037913	0.74665560	-0.18403173
H	0.06183691	0.93185115	-1.27780493
H	-1.48200456	1.09996607	-0.21156882
C	-0.21055213	2.86012260	-0.04196226
C	-1.25909838	3.72252807	-0.38077828
C	1.00709029	3.38296700	0.41077843
C	-1.08581584	5.09309710	-0.29036957
H	-2.20681264	3.31557760	-0.71834889
C	1.17290068	4.75225096	0.50201813
H	1.81390338	2.72817807	0.71978779
C	0.13003073	5.60524409	0.14705161
H	-1.89623666	5.76107890	-0.55531369
H	2.10970194	5.16054213	0.86133124
H	0.26522012	6.67800264	0.22431478
C	-0.44538355	1.42595212	-0.15929904
Cl	-0.25983363	-5.46649782	0.09696426

*

TSAB20

C	-0.05839849	-2.15353180	0.00193019
C	1.24273248	-1.67092774	-0.09300798
C	1.44012246	-0.30133367	-0.11323607
C	0.35488537	0.57470778	-0.04114919
C	-0.94192297	0.06542377	0.07128426
C	-1.15737857	-1.29885187	0.08553658
H	2.45005969	0.08324252	-0.19301247
H	-1.79525057	0.72260700	0.17522996
C	0.62750584	2.00276132	-0.05269928
H	1.65811264	2.30678355	0.12047079
H	0.34202855	2.61823055	-1.29944018
H	-1.31840426	2.73445519	-0.53179255
C	-0.05406920	4.46729216	-0.10640879
C	-0.95186839	5.36400281	-0.69189414
C	1.02407225	4.94420449	0.64530641
C	-0.76135930	6.72796357	-0.54484677
H	-1.79602591	4.99260214	-1.26406300
C	1.20657105	6.30765747	0.79180129
H	1.70362317	4.26269138	1.14516752
C	0.31868706	7.19770584	0.19293526
H	-1.45563551	7.42360431	-1.00015710
H	2.03354937	6.68082370	1.38367398
H	0.46490899	8.26481693	0.31413852
C	-0.29669952	3.03103804	-0.30313421
N	-0.28460235	-3.62000807	0.02526904
O	-0.79633346	-4.08513873	-0.96315392
O	0.06662924	-4.18909839	1.02872646
C	-2.59250643	-1.81109604	0.18782459
F	-3.38625030	-0.84955833	0.68957594
F	-2.67618025	-2.87231356	0.99096182
F	-3.06385369	-2.14017001	-1.01615363
C	2.46281112	-2.58709271	-0.15848502
F	3.50055405	-1.92407116	-0.69528057
F	2.22572854	-3.65984215	-0.91544330
F	2.82117631	-2.98792932	1.06184958

*

TSAB21

C	-0.02628994	-3.05354706	0.00851318
C	1.22469247	-2.52062597	-0.25445357
C	1.41191007	-1.15182887	-0.29188423
C	0.31693393	-0.31321019	-0.06085624
C	-0.94918288	-0.83949656	0.22090421
C	-1.10331714	-2.20946922	0.24567901
H	2.40014636	-0.76116253	-0.50374924
H	-1.80719344	-0.22069688	0.44907809
C	0.55412182	1.12143364	-0.10439158
H	1.59161201	1.44796512	-0.07183553
H	0.08733153	1.72359537	-1.30060559
H	-1.45439375	1.80521534	-0.31454601
C	-0.18535641	3.56993710	-0.06874864
C	-1.17652422	4.44236322	-0.52601883
C	0.97485776	4.07602474	0.52483634
C	-1.00016411	5.81124638	-0.40979597
H	-2.08261301	4.04820369	-0.97497103
C	1.14345686	5.44421971	0.64147107
H	1.73339716	3.41326978	0.92661945
C	0.16024942	6.31013676	0.16977098
H	-1.76727536	6.48796051	-0.76597504
H	2.03560436	5.83972875	1.11171215
H	0.29650087	7.38101554	0.26696615
C	-0.41872779	2.12720301	-0.22749057
N	-0.21452016	-4.51602112	0.04863545
O	-0.80248603	-4.99433469	-0.89088027
O	0.23826944	-5.07909954	1.01507651
F	-2.28535262	-2.73946593	0.52050279

F	2.24178077	-3.33700211	-0.48026404

*

TSAB22

C	0.09209055	-2.65924678	-0.01428412
C	1.36799180	-2.06738313	-0.18980177
C	1.50460143	-0.70195747	-0.22646636
C	0.39881375	0.15121143	-0.09600935
C	-0.86424188	-0.42994254	0.09065168
C	-1.02256360	-1.79234211	0.13307074
H	2.24643334	-2.69125232	-0.27826121
H	2.49549689	-0.27730879	-0.35786424
H	-1.74329582	0.19077479	0.23040199
H	-2.01001361	-2.20533157	0.28571416
C	0.61467608	1.59231992	-0.17250501
H	1.61988317	1.94298589	0.05242498
H	0.44218370	1.99803123	-1.31978981
H	-1.38544476	2.23879914	-0.53022188
C	-0.22278792	4.00469646	-0.07629896
C	-1.24938438	4.85011633	-0.52451339
C	0.90986011	4.55520880	0.54530341
C	-1.13669487	6.22048548	-0.37643838
H	-2.13029004	4.42331510	-0.99280808
C	1.01566138	5.92409835	0.69134902
H	1.69162982	3.91478583	0.93607642
C	-0.00345418	6.75462452	0.22722218
H	-1.92795572	6.87253780	-0.72551310
H	1.88438682	6.35158277	1.17708748
H	0.08441108	7.82835678	0.34883752
C	-0.39176002	2.58371740	-0.25200656
N	-0.05708780	-3.99454044	0.02538938
C	-1.32718174	-4.66866907	0.30728303
C	1.03646994	-4.94341751	-0.19972675
H	-1.81173795	-4.22872500	1.18191376
H	-2.00701615	-4.57624367	-0.54777008
C	-0.91454614	-6.12106029	0.54339721
H	1.60141774	-4.67439531	-1.09509457
H	1.72328299	-4.94435706	0.65481367
C	0.31901516	-6.28455276	-0.34638317
H	-0.64345988	-6.26230348	1.59271513
H	-1.71473636	-6.82034491	0.30198715
H	0.01697147	-6.43059671	-1.38658439
H	0.95116361	-7.12308408	-0.05517776

*

TSAB23

C	-0.22251740	-3.18302073	0.11987750
C	1.06989042	-2.61626207	-0.03273596
C	1.28856443	-1.26922175	-0.09820943
C	0.21830969	-0.36725317	0.00234103
C	-1.06374212	-0.88820242	0.19657377
C	-1.27676477	-2.24681121	0.25619338
H	2.31189375	-0.92526473	-0.20492094
H	-1.91776708	-0.23176096	0.32107769
H	-2.28489143	-2.60709422	0.40110584
C	0.50096392	1.05950653	-0.09121482
H	1.51931015	1.37140052	0.13100685
H	0.34304069	1.47509203	-1.25638095
H	-1.45826749	1.77886402	-0.51916435
C	-0.22482700	3.50901130	-0.09287580
C	-1.19355556	4.38577879	-0.60237721
C	0.91007499	4.02363634	0.55271277
C	-1.02041737	5.75362212	-0.49052305
H	-2.07732467	3.98680206	-1.08949412
C	1.07608774	5.39027221	0.66281620
H	1.64628100	3.36016038	0.99103991
C	0.11508563	6.25288596	0.13785950
H	-1.76716827	6.43056961	-0.88704470
H	1.94670250	5.79101500	1.16758382
H	0.24987174	7.32454211	0.23118429
C	-0.45469027	2.08912766	-0.23569158
N	-0.44374798	-4.51657279	0.17727151
C	-1.74145547	-5.01857073	0.60736540
C	0.46671949	-5.50271721	-0.41122817
H	-1.62940861	-6.06497125	0.88644500
H	-2.09086747	-4.47592516	1.48677156
H	-2.49635738	-4.94780763	-0.18444399
H	-0.13229717	-6.33920680	-0.77078255
H	0.99742791	-5.08226875	-1.26231363
H	1.19206458	-5.87267596	0.31608358
F	2.14907689	-3.42353909	-0.05730336

*

TSAB24

C	-0.16535347	-2.69119895	-0.02744358
C	1.13786396	-2.21615497	-0.08743638
C	1.38211450	-0.85489206	-0.08554589
C	0.31085030	0.04293865	-0.03356101
C	-1.00231227	-0.43600858	0.04154912
C	-1.23024990	-1.79756237	0.04045154
H	2.40197072	-0.49071236	-0.13329011
H	-1.84463413	0.23939707	0.12049729
C	0.61596543	1.46565249	-0.03350481
H	1.64526360	1.74683668	0.17991609
H	0.40222621	2.06095531	-1.26442239
H	-1.29629713	2.24033512	-0.56840357
C	-0.01057471	3.94386799	-0.09884373
C	-0.88025705	4.85807844	-0.70010849
C	1.06472004	4.40081701	0.66989191
C	-0.66442287	6.21816756	-0.55263419
H	-1.72243517	4.50232536	-1.28493290
C	1.27295923	5.76037258	0.81637793
H	1.72205883	3.70619249	1.18098926
C	0.41313403	6.66707288	0.20151358
H	-1.33680497	6.92700540	-1.02028137
H	2.09815286	6.11803873	1.42019518
H	0.57949270	7.73125205	0.32280096
C	-0.27956544	2.51509522	-0.29541073
C	-2.63930226	-2.34745002	0.14657567
F	-3.55542056	-1.38009351	-0.01893137
F	-2.83963044	-2.90443097	1.34879611
F	-2.85291870	-3.28704132	-0.78308892
C	2.28219503	-3.21030795	-0.12653204
F	3.44272469	-2.60678575	-0.42380403
F	2.05730592	-4.15902534	-1.04428642
F	2.42239965	-3.81309171	1.06265745
H	-0.35429364	-3.75937017	-0.03021914

*

TSAB3

C	-1.49393485	-3.09005438	0.30701061
C	-0.14707960	-2.86755881	0.03907274
C	0.33913743	-1.57736815	-0.05230354
C	-0.53175967	-0.49526629	0.11670731
C	-1.88354791	-0.72176561	0.39963693
C	-2.35919041	-2.01749571	0.49176987
H	1.39079978	-1.40939975	-0.25437758
H	-2.56041589	0.10588160	0.57727112
H	-3.40211841	-2.19798486	0.72080688
C	0.02901043	0.84553276	0.01431835
H	1.11042344	0.93429318	0.09495387
H	-0.21336708	1.41321494	-1.18702977
H	-1.76168151	1.94963694	-0.31184911
C	-0.14187610	3.39465846	-0.07579522
C	-0.90829912	4.44150753	-0.59700461
C	1.09513599	3.66201646	0.52076642
C	-0.43322133	5.74113601	-0.54278700
H	-1.87379329	4.23460737	-1.04738556
C	1.56265538	4.96240158	0.57509109
H	1.68158031	2.86822865	0.96968774
C	0.80216317	5.99899500	0.03939441
H	-1.02546387	6.55201250	-0.94894579

H	2.51503387	5.17458293	1.04562412
H	1.17243124	7.01656708	0.08807328
C	-0.68736562	2.03925073	-0.16566882
C	0.76906032	-4.05519219	-0.17036730
H	-1.86391038	-4.10601656	0.38661781
F	0.58479380	-4.97796127	0.78380300
F	2.06062525	-3.69046292	-0.15226252
F	0.52442007	-4.63880584	-1.35436933

*

TSAB4

C	0.05217147	-3.04574409	-0.01215935
C	1.26373800	-2.50670458	-0.41583089
C	1.41032625	-1.12895166	-0.46010603
C	0.34373657	-0.29704425	-0.11016953
C	-0.87201235	-0.85526735	0.30924971
C	-1.01476770	-2.22620280	0.35536037
H	2.08560696	-3.15538616	-0.68907277
H	2.35606142	-0.69815201	-0.77137688
H	-1.69596291	-0.22693239	0.62590648
H	-1.94658086	-2.66873342	0.68660824
C	0.55610417	1.14262434	-0.17430301
H	1.58725714	1.48543157	-0.22974330
H	0.00899336	1.69703563	-1.28815048
H	-1.47004512	1.79691461	-0.17581290
C	-0.21341508	3.57779245	-0.04094684
C	-1.26869351	4.43162702	-0.37514017
C	0.99910714	4.10750404	0.41293342
C	-1.10677256	5.80357990	-0.27783785
H	-2.21351199	4.01902253	-0.71411412
C	1.15365871	5.47839484	0.51147308
H	1.81205452	3.45870429	0.71915362
C	0.10436230	6.32466501	0.16155092
H	-1.92343495	6.46543038	-0.53911954
H	2.08735592	5.89188125	0.87300849
H	0.23031408	7.39806685	0.24435885
C	-0.43626166	2.13548887	-0.16821551
C	-0.14784991	-4.54837175	0.05677184
F	-1.15540462	-4.92240199	-0.74744940
F	-0.46404301	-4.92136436	1.30652199
F	0.94955724	-5.21765277	-0.31228344

*

TSAB5

C	-0.11609103	-4.22217726	0.05569478
C	1.11992410	-3.71244621	-0.35367061
C	1.30223066	-2.35009933	-0.41832452
C	0.26397861	-1.46702179	-0.08746407
C	-0.96366545	-1.99006884	0.33356148
C	-1.15629700	-3.35331232	0.40572112
H	1.91432114	-4.40291113	-0.60754451
H	2.26359313	-1.95716723	-0.73363621
H	-1.77265861	-1.33525751	0.63781514
H	-2.10537173	-3.75253579	0.74749855
C	0.51747322	-0.03305608	-0.18751206
H	1.55786913	0.28367558	-0.16206412
H	0.11982408	0.43341391	-1.29819652
H	-1.48889566	0.67291206	-0.25926168
C	-0.20736076	2.41261147	-0.04795019
C	-1.25072172	3.28547197	-0.38325375
C	1.01536954	2.92710376	0.40624493
C	-1.06783778	4.65386665	-0.28906165
H	-2.20104095	2.88547971	-0.72166194
C	1.19074965	4.29416021	0.50072385
H	1.81710920	2.26477336	0.71157363
C	0.15271138	5.15515456	0.14888524
H	-1.87306505	5.32908964	-0.55135510
H	2.13056533	4.69571499	0.85967386
H	0.29576190	6.22677322	0.22889165
C	-0.45157706	0.98791067	-0.16577497
O	-0.23787272	-5.55826679	0.10596117
H	-1.11885701	-5.81441180	0.40326039

*

TSAB6

C	0.14475146	-3.77023426	-0.03726125
C	1.38219675	-3.19450600	-0.36659603
C	1.51510918	-1.82869872	-0.40299074
C	0.42202957	-0.99261247	-0.12131554
C	-0.80126830	-1.57378908	0.21958678
C	-0.94868297	-2.94695694	0.26386881
H	2.21659877	-3.84998880	-0.58258723
H	2.47654000	-1.39326000	-0.65639100
H	-1.65258128	-0.95677543	0.48606625
H	-1.90215799	-3.37051304	0.54615774
C	0.61824096	0.45238425	-0.18821409
H	1.63891690	0.81401533	-0.08342454
H	0.28451063	0.91096762	-1.31699879
H	-1.40851618	1.06925636	-0.38758929
C	-0.22654833	2.86019184	-0.06129747
C	-1.28563980	3.69095257	-0.45084960
C	0.93931685	3.42202923	0.47891262
C	-1.17303753	5.06430846	-0.32495481
H	-2.19260550	3.25413934	-0.85608295
C	1.04466670	4.79371345	0.60420849
H	1.74972730	2.79158902	0.82584821
C	-0.00759422	5.61272115	0.19838763
H	-1.99026416	5.70682270	-0.62898653
H	1.93996682	5.23125527	1.02888665
H	0.08016955	6.68818409	0.30296896
C	-0.39613281	1.42908073	-0.21535020
O	0.11160227	-5.10521493	-0.02350860
C	-1.11666315	-5.75896297	0.30750993
H	-0.90660806	-6.82374433	0.24582625
H	-1.42844645	-5.50380783	1.32344431
H	-1.89936129	-5.49593304	-0.40845626

*

TSAB7

C	0.14090832	-3.86748347	0.06947540
C	1.35588168	-3.28366848	-0.30076936
C	1.49206496	-1.91010757	-0.36707603
C	0.40955858	-1.08045792	-0.06236199
C	-0.80443821	-1.65659123	0.33499640
C	-0.93205642	-3.02686346	0.40091152
H	2.20037332	-3.91962022	-0.54056841
H	2.44260738	-1.47564203	-0.65979097
H	-1.64385787	-1.03657515	0.62956639
H	-1.86701510	-3.45437512	0.74003091
C	0.60280690	0.36386412	-0.15389032
H	1.62945574	0.72310992	-0.14567170
H	0.15574283	0.82820807	-1.26635197
H	-1.43167371	0.98122662	-0.22036198
C	-0.22259090	2.77961388	-0.04036950
C	-1.30183122	3.60393567	-0.38081357
C	0.97776069	3.34826985	0.40593399
C	-1.17650876	4.97998694	-0.29875121
H	-2.23543161	3.16168958	-0.71317668
C	1.09579363	4.72263743	0.48849958
H	1.80775253	2.72348447	0.71514197
C	0.02225575	5.53644502	0.13193047
H	-2.01032652	5.61799421	-0.56493068
H	2.01881397	5.16595039	0.84191009
H	0.12020147	6.61375127	0.20243632
C	-0.40771817	1.34120850	-0.14646642
C	0.03859133	-5.33614882	0.11646530
C	-1.09539981	-6.02645736	0.17783548
H	0.98337425	-5.87010099	0.07072915
H	-1.08270702	-7.10946374	0.19747383
H	-2.07026411	-5.55179615	0.19675232

*

TSAB8

C	-0.04288249	-3.93382387	0.03152121
C	1.18369976	-3.39662874	-0.37081146
C	1.35197063	-2.02679601	-0.42306286
C	0.29810284	-1.17133752	-0.08345693
C	-0.92549957	-1.71034329	0.33266614
C	-1.09387886	-3.07796065	0.38818714
H	1.99585025	-4.06173664	-0.63548094
H	2.30633528	-1.61397141	-0.73330987
H	-1.74173051	-1.06770629	0.64217346
H	-2.03345947	-3.50233740	0.71879984
C	0.53202871	0.26657600	-0.16356413
H	1.56871578	0.59556051	-0.18281323
H	0.05781590	0.77363076	-1.27085538
H	-1.48429675	0.94469783	-0.19791825
C	-0.21570037	2.70739023	-0.03711856
C	-1.26899672	3.56684352	-0.36881348
C	1.00427181	3.23409316	0.40491708
C	-1.09844198	4.93797950	-0.28193314
H	-2.21833930	3.15689533	-0.69804960
C	1.16746086	4.60390870	0.49258583
H	1.81520219	2.58166504	0.70797537
C	0.11963033	5.45379548	0.14481796
H	-1.91262951	5.60355058	-0.54135409
H	2.10619848	5.01508385	0.84347234
H	0.25281452	6.52701153	0.21924832
C	-0.44819728	1.27281498	-0.14976884
C	-0.22254593	-5.35385138	0.09328879
C	-0.37345258	-6.54129873	0.14475037
H	-0.50745025	-7.59928537	0.18967458

*

TSAB9

C	-0.03966022	-3.93058140	0.03217093
C	1.18709674	-3.40455842	-0.37117150
C	1.34999150	-2.03269844	-0.42156866
C	0.29039222	-1.18399792	-0.08080749
C	-0.93328289	-1.72331454	0.33683822
C	-1.09881905	-3.09218024	0.39193709
H	1.99907223	-4.06965704	-0.63666801
H	2.30229756	-1.61557334	-0.73106283
H	-1.74983782	-1.08187328	0.64607937
H	-2.03612375	-3.52286929	0.72100524
C	0.52071876	0.25172630	-0.15241332
H	1.55636871	0.58120633	-0.20619929
H	-0.01911147	0.81450322	-1.27843891
H	-1.49743563	0.93304803	-0.17478835
C	-0.21711142	2.69804358	-0.04345195
C	-1.25593346	3.56466834	-0.39557516
C	0.99771591	3.21303508	0.42080795
C	-1.07504279	4.93475420	-0.30552273
H	-2.20273937	3.16370449	-0.74290365
C	1.17112184	4.58221879	0.51207966
H	1.79763317	2.55478045	0.74100544
C	0.13845225	5.44125442	0.14433298
H	-1.87889665	5.60657012	-0.58071745
H	2.10650608	4.98449471	0.88181053
H	0.27912487	6.51324393	0.22138230
C	-0.45910781	1.25755481	-0.16541526
C	-0.21643713	-5.35944517	0.09213984
N	-0.35960126	-6.49824365	0.13862612

*

TSCD0

N	0.14316320	1.17047686	-1.59548764
O	1.43411759	1.03170860	-1.22059938
C	-0.68160949	1.88824197	-0.82949049
C	-0.37464335	0.07096613	-2.37962838
H	-1.69303979	1.96562452	-1.23982744
C	-0.13760092	3.10994016	-0.14432702
H	0.27914710	-0.09118002	-3.24953380
H	-0.39582745	-0.85420434	-1.77463432
H	-1.39381367	0.29715481	-2.72605867
H	0.19785779	3.83103137	-0.90906514
H	-0.91678500	3.58144083	0.47016358
H	0.72788606	2.86746787	0.48669835
C	-0.20934285	0.54213580	1.42707658
C	-1.25654978	0.69831356	0.71805722
O	0.85823550	0.52847894	1.99321317
Li	1.97493036	0.12910433	0.30723634
O	1.79045245	-1.80022208	0.16469958
O	3.83551246	0.63795834	0.64612337
C	1.47266178	-2.70424114	1.20447616
H	0.59851041	-3.32313046	0.93226387
H	2.32713480	-3.37117891	1.42149150
H	1.23606710	-2.11510116	2.10038064
C	2.06404123	-2.42625741	-1.07376700
H	1.19170477	-3.00973224	-1.41996073
H	2.28235069	-1.63627457	-1.80507916
H	2.93302497	-3.10425280	-0.98942816
C	4.73382982	0.10321881	-0.30941996
H	5.77945549	0.32337534	-0.02869836
H	4.59304726	-0.98643002	-0.32943601
H	4.53574915	0.51642238	-1.31481782
C	3.96506921	2.04067514	0.80572037
H	3.76157068	2.56659625	-0.14347078
H	3.23439522	2.35928444	1.56220237
H	4.98068788	2.29927181	1.15564707
C	-2.51642914	-0.01478470	0.51529942
C	-3.66678966	0.65355792	0.05657885
C	-2.61203708	-1.40198727	0.75574673
C	-4.86389683	-0.03573536	-0.15298965
H	-3.62555295	1.73161637	-0.12500172
C	-3.80852785	-2.08674538	0.55086499
H	-1.72430910	-1.93964232	1.10378796
C	-4.94275069	-1.40810916	0.09175326
H	-5.74487499	0.50891074	-0.50684905
H	-3.85586351	-3.16251399	0.74720126
H	-5.88080590	-1.94658393	-0.07229193

*

TSCD1

N	-1.78172032	-0.48122030	-1.29924720
O	-0.59273599	0.14452272	-1.21892580
C	-2.76997737	-0.11883404	-0.48768336
C	-1.71504216	-1.83130085	-1.81330724
H	-3.70052550	-0.66857909	-0.65576790
C	-2.88882022	1.32644128	-0.09673608
H	-1.21032879	-1.82213349	-2.79073732
H	-1.13684155	-2.47054662	-1.11989468
H	-2.72711343	-2.24542834	-1.92881873
H	-3.07931993	1.92923082	-1.00167817
H	-3.72325171	1.46540769	0.60393155
H	-1.96266297	1.69649663	0.36264216
C	-1.33476680	-0.54051757	1.80918422
C	-2.42284519	-1.00788209	1.35768281
H	-3.10710826	-1.81884632	1.57915395
O	-0.29730459	0.03055712	2.08290849
Li	0.54532823	0.14568394	0.25186694
O	1.82699642	-1.30317302	0.15872416
O	1.63884725	1.77583434	0.20963266
C	2.73459652	-1.59738232	1.20042616
H	2.68624621	-2.66674087	1.47593025
H	3.77053239	-1.35096683	0.90296750
H	2.45370986	-0.98900076	2.07113030
C	2.06173501	-2.02244531	-1.03537022
H	1.89657243	-3.10478788	-0.88185988
H	1.35396859	-1.64710775	-1.78636518
H	3.09654622	-1.86802318	-1.39270338
C	2.40031166	1.86762906	-0.98014764
H	3.17743937	2.64777090	-0.88862465
H	2.88497594	0.89452384	-1.14115796

H	1.75534655	2.09926111	-1.84656701
C	0.95527252	2.97402994	0.52784839
H	0.26121770	3.26266346	-0.28202510
H	0.38692280	2.79845154	1.45149352
H	1.67124713	3.79903521	0.69597245

*

TSCD14

N	1.20588017	1.41320464	-1.45210965
O	2.41724840	0.91428238	-1.12483895
C	0.57897166	2.20877573	-0.58075189
C	0.43581118	0.57938181	-2.34868496
H	-0.37896484	2.58284465	-0.95469488
C	1.40161792	3.17352255	0.22691011
H	1.03290412	0.36944930	-3.24850407
H	0.18427806	-0.37966928	-1.85891514
H	-0.49391878	1.09054416	-2.63895309
H	1.88763108	3.88926594	-0.45757585
H	0.76077138	3.72989312	0.92458700
H	2.19338274	2.65958170	0.78804202
C	0.74181958	0.50420397	1.47104624
C	-0.23764561	1.00464435	0.81746212
O	1.76096675	0.16233081	2.00822228
Li	2.76218368	-0.28993837	0.23396538
O	2.10449795	-2.08310483	-0.10286681
O	4.68189867	-0.26927686	0.57855599
C	1.62975942	-3.01764113	0.84682481
H	0.62795439	-3.38879424	0.56436791
H	2.31813866	-3.87841344	0.92970806
H	1.56921767	-2.50877423	1.81798247
C	2.19136157	-2.59851928	-1.41795820
H	1.19830242	-2.92180763	-1.77897757
H	2.56698423	-1.79527870	-2.06619864
H	2.88193333	-3.46054818	-1.45836083
C	5.44089773	-0.87223484	-0.45465492
H	6.50314489	-0.95300801	-0.16237308
H	5.03964326	-1.88186753	-0.62036327
H	5.36786735	-0.29110916	-1.39153351
C	5.13880081	1.02901212	0.91997048
H	5.08055814	1.70914298	0.05223371
H	4.49301212	1.40887210	1.72396408
H	6.18110330	0.99025772	1.28415611
C	-1.61299725	0.59722608	0.59035480
C	-2.59722285	1.53385699	0.20902951
C	-2.00258071	-0.75652759	0.72724571
C	-3.91033299	1.14427000	-0.02865958
H	-2.33054616	2.58901022	0.11294052
C	-3.30965455	-1.15809623	0.49681232
H	-1.25201588	-1.49848830	1.01310984
C	-4.25434264	-0.20020987	0.11563680
H	-3.60044959	-2.20408796	0.60427265
H	-4.66582431	1.87614053	-0.31831367
N	-5.63303128	-0.61712929	-0.13679895
O	-5.91108409	-1.79773232	-0.01227703
O	-6.44398582	0.23328630	-0.46151543

*

TSCD15

N	0.92616058	1.27243943	-1.56939056
O	2.19158218	0.96737961	-1.21209184
C	0.16929806	1.98473979	-0.73083725
C	0.30899533	0.31326279	-2.45850517
H	-0.82406886	2.20692274	-1.13301403
C	0.82441616	3.06961722	0.07738661
H	0.94356704	0.18370721	-3.34787557
H	0.20449861	-0.66481094	-1.95221616
H	-0.68520503	0.66693732	-2.76886258
H	1.23996811	3.82773843	-0.60794027
H	0.08836964	3.55164697	0.73539545
H	1.65263549	2.67861247	0.68331719
C	0.49098726	0.33471131	1.34979817
C	-0.52793302	0.71340628	0.68217771
O	1.54209490	0.11592322	1.89799985
Li	2.73996277	-0.04927174	0.22764453
O	2.75991041	-1.94762459	-0.13030942
O	4.55086953	0.42432620	0.78281902
C	2.95361283	-2.92823076	0.86899410
H	2.15856753	-3.69484667	0.82709998
H	3.93525116	-3.42277627	0.75146416
H	2.91652936	-2.42469859	1.84477159
C	2.78716349	-2.45423009	-1.45051296
H	1.94710078	-3.15209043	-1.62111277
H	2.69569074	-1.59800069	-2.13190287
H	3.73625341	-2.98483096	-1.64972655
C	5.52354149	0.12330655	-0.20146200
H	6.54034578	0.16052979	0.22893910
H	5.32475222	-0.89434659	-0.56602611
H	5.46371803	0.83105081	-1.04772266
C	4.69428313	1.72234131	1.33172452
H	4.60032521	2.49660718	0.54927862
H	3.90004811	1.85901428	2.07861794
H	5.67617372	1.82967965	1.82700544
C	-1.88797259	0.23345479	0.47306248
C	-2.92211059	1.13194262	0.14991690
C	-2.19564933	-1.13955301	0.57076699
C	-4.21193291	0.66245551	-0.05983042
H	-2.74209073	2.20652746	0.07652469
C	-3.49352870	-1.57742772	0.36073324
H	-1.42248537	-1.87440197	0.80622752
C	-4.52045317	-0.69066243	0.04001084
F	-3.79278312	-2.87921683	0.45372039
F	-5.76703774	-1.12961600	-0.16395932
F	-5.20019761	1.51454151	-0.35967650

*

TSCD18

N	1.26076037	1.42963430	-1.43926063
O	2.50063114	0.96444233	-1.16677199
C	0.64409755	2.20431958	-0.54399487
C	0.48609504	0.58709345	-2.32338961
H	-0.33716057	2.55403570	-0.87895340
C	1.47045083	3.17740801	0.24850697
H	1.05554852	0.41596700	-3.24936036
H	0.28600584	-0.38940404	-1.84559037
H	-0.47153230	1.07018295	-2.56738169
H	1.94281042	3.89532762	-0.44342222
H	0.83522511	3.73000695	0.95436555
H	2.27332672	2.67145549	0.80110707
C	0.86172755	0.54356824	1.51915858
C	-0.14922352	0.97781109	0.88096544
O	1.92750209	0.25636094	2.02089098
Li	2.85916839	-0.18480782	0.25102253
O	2.25521073	-2.00398689	-0.08478492
O	4.80035924	-0.13280392	0.53089794
C	1.77649322	-2.93242382	0.86755318
H	0.78233259	-3.31679182	0.57542958
H	2.47159993	-3.78598447	0.97005890
H	1.69622755	-2.41250074	1.83143560
C	2.34740785	-2.52461091	-1.39629988
H	1.35875130	-2.86519153	-1.75409860
H	2.70823860	-1.71809550	-2.04879809
H	3.05073784	-3.37670705	-1.43443207
C	5.52439748	-0.69343867	-0.54915662
H	6.60407551	-0.73221478	-0.31781781
H	5.15711683	-1.71757101	-0.70369619
H	5.37386227	-0.10754356	-1.47363307
C	5.23134858	1.17434539	0.86927792
H	5.11888452	1.86179459	0.01263749
H	4.60678858	1.52559123	1.70245741
H	6.28838842	1.16385700	1.19122936
C	-1.53126999	0.57103418	0.61990744
C	-2.53537242	1.50525226	0.31831431

C	-1.91831320	-0.78246651	0.63936803
C	-3.84918241	1.12159436	0.04864087
H	-2.28962249	2.57188461	0.30744647
C	-3.22475514	-1.18345732	0.37645361
H	-1.16545051	-1.54389321	0.86880605
C	-4.23098899	-0.23929723	0.05813609
H	-3.45856786	-2.24827688	0.41423620
H	-4.58192798	1.89952581	-0.16987686
N	-5.52701247	-0.63258247	-0.23016036
C	-6.56346282	0.37180332	-0.34961539
C	-5.91633698	-2.01050588	-0.01365160
H	-7.51511689	-0.11953845	-0.59196464
H	-6.70477328	0.95468275	0.58181364
H	-6.34327245	1.08484637	-1.16191121
H	-6.96423665	-2.13993486	-0.31533129
H	-5.30986521	-2.70193574	-0.62235708
H	-5.82237149	-2.32204847	1.04537963

*

TSCD19

N	1.69739693	1.66492041	-1.28215347
O	2.89710857	1.10059847	-1.03003951
C	1.10971675	2.37911234	-0.31600413
C	0.88649263	0.94703393	-2.24088963
H	0.15535585	2.81556538	-0.62759407
C	1.97506550	3.23995674	0.56228741
H	1.46079389	0.81145503	-3.16920043
H	0.61477025	-0.04892614	-1.84409318
H	-0.03188079	1.51111484	-2.46056206
H	2.44405639	4.02246049	-0.05759017
H	1.36886964	3.72130428	1.34178842
H	2.78042115	2.65796922	1.02984775
C	1.30739551	0.46452798	1.54257108
C	0.32690701	1.05016337	0.95568295
O	2.31534303	0.04882077	2.03361873
Li	3.26553455	-0.26311132	0.15095732
O	2.51826973	-1.98730539	-0.31475874
O	5.18318720	-0.34702550	0.46053001
C	2.08385027	-3.01000563	0.56172722
H	1.06164058	-3.34056990	0.30281754
H	2.76199660	-3.88128423	0.51482784
H	2.08857316	-2.60419786	1.58204917
C	2.52616757	-2.37117099	-1.67786270
H	1.50946882	-2.64341158	-2.01390088
H	2.88339864	-1.51356900	-2.26396614
H	3.19720880	-3.23424134	-1.83889756
C	5.90933932	-0.84499854	-0.64999619
H	6.96104238	-1.03819913	-0.37315497
H	5.44271993	-1.79091525	-0.95921299
H	5.88330130	-0.13179043	-1.49336753
C	5.71248635	0.86368469	0.97568467
H	5.68353831	1.66323518	0.21465297
H	5.09633031	1.15804651	1.83671264
H	6.75454647	0.71914798	1.31248607
C	-1.02669415	0.62134339	0.68051279
C	-2.04279791	1.55120461	0.39112067
C	-1.37192624	-0.74702685	0.66469436
C	-3.34806622	1.14138881	0.10202877
H	-1.82291507	2.62007837	0.40007507
C	-2.66394447	-1.17306248	0.36063204
H	-0.60997671	-1.49669245	0.88703817
C	-3.66067830	-0.22440807	0.07777733
N	-5.01950475	-0.66353081	-0.24435670
O	-5.14321105	-1.70477422	-0.85831149
O	-5.94096856	0.03859469	0.12109446
C	-4.31443370	2.17184171	-0.20720145
N	-5.02019846	3.05151609	-0.46163462
C	-2.91586327	-2.59650385	0.39026879
N	-3.03314906	-3.74521507	0.45044796

*

TSCD2

N	0.91027439	1.31751917	-1.51745403
O	2.18558156	1.06259519	-1.15120883
C	0.13653747	2.04413424	-0.70720191
C	0.31725629	0.30579388	-2.36385896
H	-0.86443997	2.22166848	-1.11261712
C	0.76519668	3.17818961	0.05264321
H	0.96029056	0.14771804	-3.24238221
H	0.22822593	-0.64897629	-1.81330376
H	-0.68190332	0.62445507	-2.69555970
H	1.15760008	3.91832266	-0.66527093
H	0.01923443	3.66713952	0.69420751
H	1.60625258	2.83365355	0.66894980
C	0.50396221	0.51536620	1.45330292
C	-0.52566458	0.80842401	0.76101097
O	1.56387217	0.37100695	2.01314527
Li	2.66485690	0.01552547	0.30055229
O	2.36517208	-1.88481146	0.02106223
O	4.54622390	0.38357609	0.68648530
C	1.89178228	-2.82899354	0.96140061
H	0.95139894	-3.29232394	0.61145090
H	2.63957889	-3.62540692	1.12988654
H	1.70757811	-2.29956170	1.90554491
C	2.60697991	-2.42989152	-1.26185512
H	1.68614344	-2.87568513	-1.67960443
H	2.93857747	-1.61187592	-1.91563952
H	3.38972716	-3.20903892	-1.21709460
C	5.43762112	-0.14977314	-0.27603553
H	6.48561526	-0.02661653	0.05134761
H	5.22024027	-1.22230623	-0.37640282
H	5.30641033	0.34386448	-1.25583622
C	4.76468161	1.75999410	0.94574568
H	4.62735572	2.36136675	0.03012919
H	4.03302862	2.07703392	1.70197181
H	5.78358190	1.92367016	1.34051826
C	-1.84503223	0.22729582	0.53100815
C	-2.93626758	1.02734128	0.14492317
C	-2.06482126	-1.15936684	0.66850763
C	-4.19500250	0.47401358	-0.09441995
H	-2.80412269	2.10805617	0.04250821
C	-3.31658881	-1.72330834	0.43711488
H	-1.22922466	-1.80340410	0.95825492
C	-4.37667998	-0.89970076	0.05259391
H	-3.47182035	-2.79922648	0.54892101
H	-5.03414773	1.10880346	-0.38938704
Cl	-5.94590620	-1.59996077	-0.24624625

*

TSCD21

N	1.54973370	1.56566080	-1.35427778
O	2.75148158	1.02196764	-1.06653485
C	0.93894815	2.30536014	-0.42381990
C	0.76302143	0.81158709	-2.30533921
H	-0.01366689	2.72204277	-0.76500992
C	1.78002903	3.19701475	0.44670367
H	1.35404223	0.65649628	-3.22006764
H	0.49615594	-0.17551084	-1.88421307
H	-0.15807671	1.35823234	-2.55541720
H	2.26389606	3.95968145	-0.18654599
H	1.15292495	3.70262720	1.19367736
H	2.57405957	2.63396586	0.95502793
C	1.09885708	0.45443286	1.50233780
C	0.12421830	1.01019070	0.88289217
O	2.10914193	0.06602835	2.01708059
Li	3.09606979	-0.28811884	0.18295393
O	2.38985844	-2.03984691	-0.24483921
O	5.01278828	-0.33656782	0.52403971
C	1.94896768	-3.03520633	0.65875435
H	0.94085608	-3.39475444	0.38416477
H	2.64384391	-3.89462043	0.66618093
H	1.91635689	-2.58896278	1.66150605

C	2.43717612	-2.47284099	-1.59182760
H	1.43228207	-2.76763010	-1.94414797
H	2.80133901	-1.63329832	-2.19943482
H	3.12035196	-3.33421885	-1.70439634
C	5.75907275	-0.87467681	-0.55355773
H	6.81369375	-1.02476328	-0.26121337
H	5.31957536	-1.84803519	-0.81311696
H	5.72023236	-0.20931654	-1.43475026
C	5.50993648	0.91162095	0.97791318
H	5.47437148	1.66705638	0.17348655
H	4.87579799	1.23949123	1.81343455
H	6.55010588	0.80821453	1.33532756
C	-1.24139730	0.59948738	0.62551176
C	-2.22673554	1.54636973	0.27992313
C	-1.61121552	-0.76345039	0.69460401
C	-3.52477894	1.13992339	0.02419727
H	-1.99936055	2.61189902	0.21843127
C	-2.90882055	-1.14821612	0.42361796
H	-0.88090659	-1.53187369	0.95665939
C	-3.89631937	-0.20968248	0.08286906
N	-5.25864245	-0.62625565	-0.20360685
O	-5.41599201	-1.63040395	-0.87275045
O	-6.16596489	0.05178170	0.24067955
F	-3.23657928	-2.43586863	0.51635357
F	-4.43127499	2.05349123	-0.31621101

*

TSCD6

N	0.86973654	1.36142833	-1.48741245
O	2.15933915	1.11174095	-1.16856201
C	0.11921832	2.07615254	-0.64573230
C	0.25461096	0.35451609	-2.32359101
H	-0.89575496	2.25189015	-1.01549013
C	0.76773997	3.20338687	0.10717747
H	0.86924614	0.20851419	-3.22434038
H	0.18872693	-0.60596820	-1.77978197
H	-0.75684800	0.67048318	-2.61861007
H	1.14278586	3.94971679	-0.61365590
H	0.03870354	3.68690168	0.77192989
H	1.62361553	2.85289350	0.69923768
C	0.54103909	0.53691387	1.49065633
C	-0.50924377	0.82332729	0.83131699
O	1.62440597	0.39684653	2.01233626
Li	2.66964139	0.06224323	0.27497899
O	2.38047539	-1.83836669	-0.02617012
O	4.56050591	0.44618033	0.61332311
C	1.90597859	-2.79201660	0.90370827
H	0.95856206	-3.24158472	0.55464346
H	2.64764956	-3.59771980	1.05458604
H	1.73384086	-2.27469079	1.85680509
C	2.60443553	-2.36526503	-1.31955420
H	1.67738042	-2.80311998	-1.73202895
H	2.92886267	-1.53838464	-1.96570106
H	3.38601421	-3.14662051	-1.29623041
C	5.42352438	-0.03731834	-0.39977035
H	6.48105374	0.10114178	-0.11126465
H	5.22684784	-1.11141358	-0.52366070
H	5.24069875	0.48161936	-1.35793808
C	4.75993895	1.81875482	0.90537023
H	4.57923814	2.44292999	0.01279949
H	4.04863166	2.09806896	1.69509303
H	5.78852557	1.99278414	1.26964610
C	-1.84320747	0.26098279	0.62069594
C	-2.93972715	1.07384542	0.30222827
C	-2.07458058	-1.13010103	0.70799919
C	-4.21672412	0.54249074	0.08015883
H	-2.80425822	2.15755204	0.23556870
C	-3.33499550	-1.67076317	0.49424902
H	-1.23633503	-1.79247193	0.94568401
C	-4.42004691	-0.83712326	0.17515532
H	-3.50125713	-2.74920661	0.56592290
H	-5.03812028	1.21916120	-0.16077860
O	-5.61848625	-1.45175133	-0.02326627
C	-6.74014252	-0.65015371	-0.34625647
H	-6.58559833	-0.10238693	-1.29194481
H	-7.59042534	-1.33532405	-0.46390273
H	-6.96687427	0.06975108	0.45908267

*

TSCD8

N	0.85513168	1.35477351	-1.47430146
O	2.08129111	0.88722701	-1.15294622
C	0.21801336	2.15015739	-0.61130411
C	0.09710839	0.49471039	-2.35622863
H	-0.75108583	2.49762207	-0.98206902
C	1.02610802	3.13970118	0.18029818
H	0.69158643	0.28943160	-3.25897666
H	-0.12897144	-0.46462622	-1.85522436
H	-0.84648130	0.98168988	-2.64331722
H	1.50529069	3.84945594	-0.51526338
H	0.37641350	3.69981842	0.86672022
H	1.82258308	2.64611386	0.75288202
C	0.40548767	0.49748190	1.47613447
C	-0.58629400	0.95272391	0.81492833
O	1.44329931	0.19421786	2.01084273
Li	2.43029928	-0.26873307	0.25387058
O	1.81227500	-2.08174081	-0.06730245
O	4.35644514	-0.21512902	0.58454630
C	1.33480925	-3.00693990	0.88951274
H	0.34204513	-3.39516685	0.59803794
H	2.03228786	-3.85780496	0.99670116
H	1.25275058	-2.48311766	1.85112011
C	1.91985697	-2.61357102	-1.37380450
H	0.93595472	-2.95956657	-1.73905796
H	2.28636895	-1.81218137	-2.02946146
H	2.62577443	-3.46389769	-1.39644743
C	5.10982963	-0.80050138	-0.46243366
H	6.18041094	-0.84793152	-0.19402822
H	4.73563273	-1.82260657	-0.61413043
H	4.99767927	-0.22693110	-1.40006169
C	4.78938631	1.09335042	0.91686462
H	4.70877313	1.76910791	0.04746296
H	4.14377371	1.46242985	1.72599928
H	5.83604825	1.07788832	1.27047381
C	-1.96327549	0.53411582	0.58058375
C	-2.95651220	1.46176107	0.21235191
C	-2.34191395	-0.82122343	0.69740836
C	-4.26782285	1.05845696	-0.02851966
H	-2.69925884	2.52113209	0.12783263
C	-3.64939760	-1.22825805	0.46266694
H	-1.58418258	-1.56071530	0.97320863
C	-4.63280010	-0.29207480	0.09257487
H	-3.91936227	-2.28327445	0.56076154
H	-5.02324271	1.79755226	-0.30882866
C	-5.99113843	-0.71318463	-0.15474249
C	-7.13206035	-1.06896419	-0.35973406
H	-8.14766200	-1.38539502	-0.54176103

*

TSCD9

N	0.84273321	1.37132213	-1.47354004
O	2.06638042	0.90501128	-1.14256855
C	0.19710166	2.16165338	-0.61165026
C	0.09267216	0.51119267	-2.36218044
H	-0.76959065	2.50838626	-0.98931905
C	0.99650130	3.15257194	0.18732799
H	0.69447647	0.30715821	-3.26025938
H	-0.13637638	-0.44884358	-1.86357821
H	-0.84891644	0.99767937	-2.65660658
H	1.47177386	3.86935938	-0.50362169
H	0.34143722	3.70450023	0.87525543
H	1.79540326	2.66138426	0.75857350

C	0.38767683	0.49152481	1.46488619
C	-0.60156572	0.95548609	0.80223694
O	1.41771102	0.18314704	2.00581499
Li	2.42535720	-0.26779048	0.24327969
O	1.80708325	-2.07828226	-0.08100544
O	4.34483700	-0.20901969	0.59170246
C	1.34521389	-3.01214281	0.87534147
H	0.35448578	-3.40905495	0.58875313
H	2.05188041	-3.85631348	0.97438901
H	1.26455022	-2.49327465	1.83973176
C	1.91536954	-2.60549593	-1.38960737
H	0.93313964	-2.95767814	-1.75331920
H	2.27460786	-1.79972989	-2.04394676
H	2.62759334	-3.45033297	-1.41639134
C	5.11499699	-0.80144773	-0.43916217
H	6.17938419	-0.85682085	-0.14871928
H	4.73592794	-1.82076868	-0.59758118
H	5.02699851	-0.22813026	-1.37955262
C	4.77637181	1.09920474	0.92747625
H	4.70928117	1.77346513	0.05576224
H	4.12053050	1.47153915	1.72685514
H	5.81787470	1.08182262	1.29569905
C	-1.97182341	0.52367145	0.57144891
C	-2.97019700	1.44165515	0.18714213
C	-2.33869417	-0.83475364	0.70840315
C	-4.27640412	1.02763627	-0.05275048
H	-2.72011031	2.50112450	0.08995146
C	-3.63986711	-1.25694605	0.47573063
H	-1.57566230	-1.56323342	0.99703379
C	-4.61962818	-0.32551395	0.08862272
H	-3.90502624	-2.31142061	0.58704660
H	-5.03847511	1.75418274	-0.34592858
C	-5.97137052	-0.75955939	-0.16060012
N	-7.05772789	-1.10969254	-0.36083748

*

TSEF0

Au	0.10974070	-1.44924912	0.06995438
C	1.30523643	0.91804476	0.64973829
C	0.12022197	0.52079202	0.82727128
C	0.45790355	3.17459700	1.32763448
C	1.74789665	2.87233822	1.61500609
H	1.99696156	2.37052497	2.54300183
C	-1.07021803	1.21990088	1.36816678
H	-1.64431303	0.58327185	2.04196954
H	-1.72032023	1.47742239	0.52766036
C	-0.61433987	2.51066051	2.11762292
P	-0.03737829	-3.59618415	-0.71668578
C	1.57072396	-4.37107943	-1.00815239
C	-0.90635891	-4.70827894	0.41413275
H	2.30184710	0.83679323	0.25619635
C	-0.92213033	-3.72485901	-2.28867483
H	-0.40919919	-3.12786349	-3.04481976
H	-0.95982583	-4.76825501	-2.61042791
H	-1.93766176	-3.34393671	-2.16619649
H	2.12589039	-3.78946203	-1.74625579
H	1.43098699	-5.39020959	-1.37649246
H	2.13844478	-4.39463511	-0.07630406
H	-0.37860113	-4.74064665	1.36897592
H	-0.95104548	-5.71294717	-0.01277614
H	-1.91930134	-4.33807275	0.58215134
H	2.56854392	3.28671093	1.04024261
H	-1.49087010	3.14446475	2.24946836
H	-0.23162444	2.22721965	3.09820367
C	0.08612370	3.98154528	0.13874234
C	-1.05069225	4.79240387	0.15895398
C	0.86750850	3.94878792	-1.02053798
C	-1.38777174	5.56509490	-0.94554658
H	-1.66758371	4.84379473	1.04820932
C	0.52372940	4.71155465	-2.12669315
H	1.73892316	3.30553940	-1.06593700
C	-0.60400843	5.52578568	-2.09173609
H	-2.26717138	6.19882766	-0.90815419
H	1.13511767	4.66666457	-3.02149870
H	-0.87154011	6.12374877	-2.95621269

*

TSEF1

Au	0.00544033	-1.67646189	0.21353575
C	1.14997038	0.59006782	1.14956408
C	-0.06141235	0.24304165	1.09028678
C	0.27045352	2.85610074	1.77185070
C	1.47505900	2.49580278	2.27889512
H	1.53153756	1.94800505	3.21251157
C	-1.30574495	0.96750218	1.44498159
H	-2.01319166	0.32595219	1.97119264
H	-1.78415650	1.28953753	0.51610636
C	-0.94660459	2.20210890	2.32794806
P	-0.06988690	-3.75821695	-0.73974544
C	0.92791167	-3.90031086	-2.24154571
C	0.51549551	-5.07595761	0.35174788
H	2.19947923	0.48634875	0.94416869
C	-1.74065278	-4.24836758	-1.22836165
H	-2.13379943	-3.53024690	-1.95018561
H	-1.71973422	-5.24381718	-1.67808291
H	-2.38875302	-4.25850607	-0.35021892
H	0.57768228	-3.17719770	-2.98029475
H	0.84330081	-4.91012019	-2.65004539
H	1.97234046	-3.68826209	-2.00595348
H	1.55259902	-4.88285876	0.63177755
H	0.44861637	-6.03939037	-0.15918776
H	-0.09652072	-5.09849757	1.25519863
H	2.40167139	2.89768657	1.88490724
H	-1.81091392	2.86577305	2.34152951
H	-0.75100131	1.85923859	3.34419781
C	0.14505873	3.70815580	0.56490565
C	-0.97668111	4.51271384	0.36719832
C	1.14851392	3.72417538	-0.41139908
C	-1.08263420	5.32205361	-0.75829190
H	-1.77180652	4.53250451	1.10304464
C	1.03235677	4.52286503	-1.53522078
H	2.01821993	3.08610250	-0.30263514
C	-0.08445146	5.34100926	-1.72728944
H	-1.96015024	5.94863261	-0.88253964
H	1.81886062	4.50962836	-2.28382893
C	-0.19365589	6.21130880	-2.95071533
H	-1.14224059	6.75082984	-2.96707041
H	0.61806968	6.94385494	-2.97775802
H	-0.12233066	5.61154233	-3.86222077

*

TSEF10

Au	-0.01751893	-1.58653659	0.22680800
C	1.23573076	0.74998710	0.79051301
C	0.06905129	0.35075100	1.05994703
C	0.46324579	2.98467317	1.62293919
C	1.77265566	2.67093105	1.78033941
H	2.10335323	2.13511442	2.66275441
C	-1.06452679	1.03267960	1.72953475
H	-1.58117198	0.37325715	2.42746218
H	-1.78416106	1.32326273	0.95941433
C	-0.53613726	2.29374178	2.48204559
P	-0.24535201	-3.69670929	-0.63571778
C	0.27144660	-3.83017877	-2.36377302
C	0.71563549	-4.94696172	0.24977616
H	2.19257885	0.68095676	0.30654643
C	-1.95250603	-4.29236649	-0.60028340
H	-2.58491992	-3.61918195	-1.18177066
H	-2.00425970	-5.29836767	-1.02311641
H	-2.31012165	-4.31126953	0.43074906
H	-0.32326405	-3.14876801	-2.97471216
H	0.13029628	-4.85460867	-2.71624586

H	1.32434169	-3.55614854	-2.45085490
H	1.77543400	-4.68976438	0.20554416
H	0.55855058	-5.92799976	-0.20474201
H	0.40149267	-4.97427210	1.29468382
H	2.53891920	3.10518546	1.14809510
H	-1.39377566	2.92461594	2.71420618
H	-0.06874933	1.97351445	3.41348588
C	-0.01304998	3.83394296	0.50282943
C	-1.12961935	4.65372492	0.66060633
C	0.65408011	3.82771962	-0.72746861
C	-1.55207001	5.46194302	-0.38936250
H	-1.65944530	4.68600363	1.60512296
C	0.23298044	4.62084107	-1.78712263
H	1.50712585	3.17286731	-0.86966479
C	-0.88041484	5.44460645	-1.60203572
H	-2.41365127	6.10751967	-0.25643663
H	-1.22217898	6.07243971	-2.41945195
C	0.95245498	4.59549418	-3.11083527
H	0.27079402	4.31671845	-3.91900778
H	1.35711853	5.58271492	-3.35072703
H	1.77827108	3.88181116	-3.09698400

*

TSEF11

Au	-0.04065978	-1.59067634	0.22649639
C	1.20779660	0.74235934	0.84191728
C	0.02895133	0.34560122	1.06133298
C	0.41744798	2.97645130	1.62855265
C	1.71628020	2.65267098	1.84368907
H	2.00445678	2.12199007	2.74391862
C	-1.12773982	1.03685256	1.68010669
H	-1.67537350	0.38433856	2.36070438
H	-1.81435163	1.32559611	0.87975903
C	-0.62534334	2.30088595	2.44621899
P	-0.25066404	-3.69738808	-0.64907931
C	0.24768843	-3.81067524	-2.38399324
C	0.73636129	-4.94258299	0.21486335
H	2.18219176	0.66448167	0.39536505
C	-1.94956958	-4.31529679	-0.59900051
H	-2.59759881	-3.64439393	-1.16575725
H	-1.99378061	-5.31752753	-1.03153207
H	-2.29426817	-4.34919166	0.43605397
H	-0.36280691	-3.13235048	-2.98268808
H	0.11577652	-4.83406450	-2.74300071
H	1.29584395	-3.52215662	-2.48103798
H	1.79241617	-4.67260497	0.15841627
H	0.58476382	-5.92218629	-0.24459183
H	0.43656859	-4.98096608	1.26362256
H	2.51207580	3.07684925	1.24175052
H	-1.48767556	2.94028055	2.63272159
H	-0.20341391	1.98512986	3.40052052
C	-0.00087591	3.82602759	0.48449567
C	-1.10776821	4.67042969	0.59909414
C	0.71567035	3.79302221	-0.71170906
C	-1.47199509	5.47982442	-0.46933168
H	-1.66998022	4.71927181	1.52317365
C	0.33388037	4.59694440	-1.77822838
H	1.56254881	3.12862807	-0.83710629
C	-0.76211637	5.44886296	-1.66122101
H	-2.32340763	6.14486309	-0.37382536
H	-1.05425255	6.07566335	-2.49832121
O	1.06714412	4.50641940	-2.92410309
H	0.70525001	5.11491735	-3.58353386

*

TSEF12

Au	-0.13843267	-2.10574470	0.12934486
C	0.87879785	-0.06071780	1.57071497
C	-0.31079657	-0.31831808	1.24053790
C	-0.02042533	2.15919772	2.32810615
C	1.04839685	1.66132526	2.99700474
H	0.90244725	0.99433500	3.83886108
C	-1.57279657	0.43006827	1.45485260
H	-2.39093472	-0.22889124	1.74718811
H	-1.85185736	0.89771509	0.50675977
C	-1.34574020	1.51672089	2.55077156
P	-0.08768806	-4.03203227	-1.10950774
C	0.90621555	-3.88963834	-2.61382689
C	0.59550660	-5.44966850	-0.21796153
H	1.94384672	-0.20073274	1.55214070
C	-1.72551866	-4.55969733	-1.66576277
H	-2.17217852	-3.77411128	-2.27784415
H	-1.64095587	-5.47682099	-2.25337800
H	-2.36402648	-4.73924311	-0.79897735
H	0.50120742	-3.09524696	-3.24328351
H	0.89005462	-4.83434871	-3.16238840
H	1.93485295	-3.64027132	-2.34737989
H	1.62060291	-5.22901567	0.08501117
H	0.58678373	-6.33304287	-0.86082551
H	-0.00336744	-5.64171336	0.67406470
H	2.04814075	2.05112630	2.84260147
H	-2.17161442	2.22552017	2.49339835
H	-1.36389206	1.03494567	3.52857543
C	0.12307512	3.16377867	1.24785358
C	-0.91421827	4.04974581	0.94530374
C	1.29795462	3.24960603	0.49642987
C	-0.77557762	4.99734978	-0.05759938
H	-1.83788016	4.02074324	1.51098083
C	1.42950194	4.18956884	-0.51295008
H	2.11197924	2.55858459	0.68395586
C	0.39814000	5.08371240	-0.80798335
H	-1.59925936	5.67578010	-0.25256525
H	2.34796178	4.22839309	-1.09018972
C	0.57862518	6.07853996	-1.89885213
C	-0.58054354	6.47153482	-2.78274234
C	-0.03315455	7.45465546	-1.79675307
H	1.54652270	6.02562058	-2.38363738
H	-1.54050636	6.01722117	-2.56573314
H	-0.37162290	6.62866631	-3.83422081
H	0.55170659	8.28604199	-2.17176584
H	-0.62259530	7.66720120	-0.91236655

*

TSEF13

Au	-0.01130922	-1.75244301	0.20801904
C	1.27869023	0.56255042	0.83510057
C	0.13981891	0.12235980	1.16219144
C	0.57285337	2.70536488	1.88465569
C	1.89126923	2.38829133	1.91204231
H	2.29224362	1.79758031	2.72749522
C	-0.93725660	0.74123206	1.97218910
H	-1.39093208	0.02796217	2.66086121
H	-1.72062695	1.07958088	1.28856552
C	-0.35349682	1.94982808	2.76951439
P	-0.29419543	-3.80306458	-0.77481653
C	0.26536339	-3.86307286	-2.49368037
C	0.59650248	-5.13361612	0.06592800
H	2.18879062	0.53575940	0.26394736
C	-2.02192293	-4.33593417	-0.81567501
H	-2.61423080	-3.60861618	-1.37373117
H	-2.09812436	-5.31422677	-1.29592625
H	-2.40744716	-4.39892178	0.20351263
H	-0.28860208	-3.12966043	-3.08232801
H	0.09926572	-4.86177537	-2.90437982
H	1.32900391	-3.62246796	-2.53871802
H	1.66595124	-4.91565330	0.06018725
H	0.41455661	-6.08325931	-0.44274546
H	0.25513094	-5.20265475	1.10034398
H	2.60404936	2.87111292	1.25300179
H	-1.19016681	2.55529262	3.11740698
H	0.18988561	1.56759357	3.63366778
C	0.01050887	3.62813127	0.86680966

C	-1.10590009	4.41415357	1.15554886
C	0.58464301	3.73082756	-0.40224746
C	-1.62760853	5.29412530	0.21081588
H	-1.56780106	4.36119994	2.13410331
C	0.05759746	4.59887600	-1.34907021
H	1.43747256	3.11600023	-0.67111168
C	-1.05182175	5.38876768	-1.04383940
H	-2.48844936	5.90249563	0.46400722
H	-1.45023156	6.06221078	-1.79405016
C	0.69230857	4.65995897	-2.68797553
O	0.31969517	5.38011441	-3.58765567
H	1.55832579	3.98571863	-2.82544833

*

TSEF14

Au	0.01325105	-2.11937632	0.12762093
C	1.19667631	0.07435103	1.27036919
C	-0.01087881	-0.29610891	1.18308152
C	0.33392342	2.20282167	2.18672785
C	1.54858517	1.76095363	2.60087415
H	1.62393592	1.10425155	3.45982252
C	-1.24978505	0.37533541	1.64486670
H	-1.94882329	-0.32487167	2.10287816
H	-1.74468590	0.80657888	0.77048795
C	-0.88188631	1.49769574	2.66782581
P	-0.09475940	-4.11144648	-1.00025699
C	1.10908640	-4.25638816	-2.34219841
C	0.19444832	-5.55018820	0.05708615
H	2.23593232	-0.00786812	1.00721238
C	-1.70604186	-4.39884203	-1.76976387
H	-1.91561567	-3.60064595	-2.48410486
H	-1.70619253	-5.36100985	-2.28726707
H	-2.48113775	-4.39822072	-1.00118275
H	0.94620490	-3.45622488	-3.06638635
H	0.99907212	-5.22450527	-2.83662270
H	2.11847495	-4.16683489	-1.93677210
H	1.18790896	-5.47941409	0.50359034
H	0.12399663	-6.46674498	-0.53332856
H	-0.55090163	-5.57145483	0.85428624
H	2.46650764	2.21000261	2.23821791
H	-1.74245098	2.16003512	2.75723077
H	-0.68439762	1.03556842	3.63495012
C	0.20025268	3.23576688	1.12830409
C	-0.85632223	4.14786934	1.17076632
C	1.12630346	3.31246252	0.08366658
C	-0.98523996	5.12713774	0.19853460
H	-1.57670624	4.11478299	1.97826207
C	1.00393693	4.27766226	-0.90055201
H	1.93980418	2.59996413	0.02345094
C	-0.05290501	5.17059941	-0.82319611
H	-1.79589643	5.84206924	0.23237074
H	1.71141692	4.33396413	-1.71649336
N	-0.19293542	6.19873205	-1.87027465
O	-1.11901352	6.98253705	-1.77904790
O	0.62461168	6.20932888	-2.77145126

*

TSEF15

Au	-0.05207771	-2.03842258	0.21055229
C	1.28148311	0.19013607	1.06657861
C	0.07254254	-0.16835276	1.17493356
C	0.56079880	2.38919319	1.93376201
C	1.82008941	1.97064387	2.21683179
H	2.00716829	1.37928834	3.10540822
C	-1.08696754	0.54584470	1.76231072
H	-1.71063102	-0.11419352	2.36575475
H	-1.70434824	0.91807264	0.94027654
C	-0.57427355	1.73482249	2.63585276
P	-0.31162085	-4.06865246	-0.82057569
C	0.62513886	-4.21839038	-2.36039712
C	0.21162977	-5.46891535	0.19741977
H	2.27453836	0.08169365	0.66896510
C	-2.02794447	-4.41972746	-1.26972843
H	-2.39225618	-3.64562216	-1.94744820
H	-2.09245157	-5.39385751	-1.76007745
H	-2.64646326	-4.42245758	-0.37042533
H	0.30220117	-3.44421724	-3.05878200
H	0.45798653	-5.20290323	-2.80371741
H	1.68879274	-4.08718909	-2.15371148
H	1.26889871	-5.36268682	0.44688545
H	0.05723534	-6.40336616	-0.34722507
H	-0.36988056	-5.48449114	1.12094502
H	2.68337337	2.37611639	1.70149704
H	-1.41165505	2.41086224	2.80538526
H	-0.23686246	1.34304545	3.59516244
C	0.28012915	3.32278879	0.81357794
C	-0.82317235	4.17457147	0.87217218
C	1.11783162	3.35587731	-0.30405643
C	-1.06159375	5.05074665	-0.16729562
H	-1.49340146	4.19220509	1.72167754
C	0.84653890	4.23364168	-1.33012395
H	1.96768736	2.69251783	-0.40259955
C	-0.23912373	5.09416564	-1.27920602
F	-2.11104616	5.88735574	-0.11973208
F	-0.48772154	5.94016889	-2.28328150
F	1.63131103	4.26760254	-2.41943763

*

TSEF16

Au	-0.06256493	-1.83509652	0.21079106
C	1.17798103	0.40182075	1.07962660
C	-0.02759014	0.04557678	1.17193679
C	0.36354257	2.63470036	1.93288388
C	1.62915454	2.26705659	2.25183711
H	1.81481892	1.67076714	3.13759963
C	-1.21871598	0.73055994	1.72842630
H	-1.83952834	0.05163967	2.31377789
H	-1.82388020	1.08829335	0.89106306
C	-0.75649372	1.92726415	2.61523454
P	-0.25572912	-3.87862534	-0.80639270
C	0.35468558	-3.89614684	-2.50865996
C	0.64496888	-5.19824166	0.04068746
H	2.19141774	0.32543691	0.73139518
C	-1.96714616	-4.45483138	-0.90528205
H	-2.56176881	-3.73473699	-1.47019107
H	-2.00389350	-5.42758958	-1.40119801
H	-2.38007082	-4.54269767	0.10126660
H	-0.20131840	-3.16764535	-3.10146180
H	0.22702023	-4.89190093	-2.93981247
H	1.41242363	-3.62720537	-2.51862902
H	1.70769113	-4.95155274	0.07205269
H	0.50484720	-6.14357804	-0.48882559
H	0.27354607	-5.29392263	1.06255110
H	2.49228321	2.70338974	1.76221346
H	-1.62101881	2.56837219	2.78398481
H	-0.41656795	1.53859397	3.57534212
C	0.06118320	3.54733805	0.80639241
C	-1.11630705	4.29222626	0.78178579
C	0.96070179	3.68146162	-0.26126028
C	-1.38789334	5.16384734	-0.26704778
H	-1.82845452	4.22331956	1.59409609
C	0.68828606	4.53961562	-1.30649545
H	1.87166442	3.09304803	-0.29432199
C	-0.49376936	5.29311033	-1.31494349
H	-2.29843150	5.75241878	-0.27818347
O	-0.76990279	6.13846786	-2.34141210
H	-0.03346614	6.11562166	-2.96986105
O	1.50655511	4.71573557	-2.38865622
H	2.31550670	4.19743442	-2.27802104

*

TSEF17

Au	0.04996608	-2.34223454	0.17187213
C	1.50180728	-0.24853789	1.05670748
C	0.27007253	-0.49216641	1.16759097
C	0.91799105	2.03175064	1.96980868
C	2.15192983	1.54437486	2.24902429
H	2.30442839	0.91245163	3.11635372
C	-0.83984440	0.29076420	1.76223614
H	-1.51348835	-0.33843268	2.34470510
H	-1.42165989	0.72486066	0.94460709
C	-0.24717727	1.41580967	2.66524742
P	-0.33206448	-4.34108699	-0.87973787
C	0.22391947	-4.36813325	-2.60048900
C	0.48986852	-5.74384614	-0.08782317
H	2.49661973	-0.41097665	0.68489521
C	-2.08493158	-4.78269501	-0.93645133
H	-2.63840574	-4.00784609	-1.46985296
H	-2.21099425	-5.73942725	-1.44862284
H	-2.47387516	-4.85929869	0.08048235
H	-0.29412939	-3.58869265	-3.16206573
H	0.00970046	-5.34270302	-3.04548286
H	1.29790174	-4.17761286	-2.63907835
H	1.56881400	-5.57905346	-0.08523328
H	0.26226269	-6.66391709	-0.63132858
H	0.14311234	-5.83373252	0.94317896
H	3.03760162	1.91312636	1.74422472
H	-1.04271516	2.13182148	2.87036776
H	0.07574748	0.97259521	3.60746268
C	0.67638580	2.98838198	0.86496834
C	1.51370719	3.03317730	-0.24052996
C	-0.42095065	3.86482151	0.91450963
C	1.27826605	3.93275035	-1.27823790
H	2.34863509	2.34786879	-0.32413451
C	-0.65858390	4.77022765	-0.10332439
H	-1.07794424	3.85146576	1.77200282
C	0.20393010	4.80765038	-1.22288368
H	1.94065390	3.93652102	-2.13362398
O	-0.10394482	5.71728310	-2.17620753
O	-1.68558276	5.65752310	-0.12168262
C	0.74883000	5.78123660	-3.31767099
H	0.33891558	6.56678145	-3.95104046
H	1.77205158	6.04113061	-3.03086327
H	0.74634867	4.83382612	-3.86462439
C	-2.57694712	5.64617949	0.98970810
H	-3.07738917	4.67753671	1.08542931
H	-2.05229643	5.88462421	1.92003705
H	-3.31833437	6.41756700	0.78491730

*

TSEF18

Au	-0.10021442	-2.10409805	0.12570348
C	1.09082343	-0.09625334	1.42753518
C	-0.13529634	-0.34293340	1.29757184
C	0.28059211	2.12517444	2.39451589
C	1.45101991	1.64010556	2.87788771
H	1.44830118	0.94173884	3.70668438
C	-1.35787115	0.37045586	1.73735218
H	-2.10274334	-0.31737454	2.13888765
H	-1.79786781	0.85677682	0.86260829
C	-0.97169298	1.42852177	2.81395078
P	-0.22737123	-4.00930496	-1.13916629
C	0.61241058	-3.87778131	-2.73545520
C	0.49318522	-5.45981280	-0.33498663
H	2.14393434	-0.20691970	1.24851025
C	-1.93025301	-4.47451133	-1.53237251
H	-2.40767788	-3.66716531	-2.09055164
H	-1.93772097	-5.38737285	-2.13256667
H	-2.48511685	-4.64154439	-0.60745004
H	0.17442693	-3.06003463	-3.31046359
H	0.50460448	-4.81314133	-3.28975573
H	1.67153957	-3.66952730	-2.57374808
H	1.54976846	-5.27602256	-0.13218780
H	0.39215341	-6.33345121	-0.98323206
H	-0.02160838	-5.64451610	0.60955790
H	2.41000522	2.05428913	2.58905506
H	-1.81516336	2.10784047	2.93431129
H	-0.80505533	0.91499372	3.76119398
C	0.21402602	3.15239023	1.33688934
C	-0.95352226	3.88141787	1.09381305
C	1.31228964	3.42861049	0.51243183
C	-1.02281966	4.85037740	0.10827707
H	-1.83938104	3.70870845	1.69325012
C	1.26078823	4.38379014	-0.48260947
H	2.22967366	2.86213612	0.62738938
C	0.09102951	5.14347528	-0.70292055
H	-1.95382054	5.38365360	-0.02567822
H	2.13649894	4.53562221	-1.09849085
N	0.04343525	6.12626358	-1.66242978
C	1.10818500	6.20063608	-2.65018493
C	-1.24935528	6.66933057	-2.04771614
H	0.91880410	7.05054633	-3.30407392
H	2.07563999	6.36161313	-2.17014066
H	1.17065700	5.29355136	-3.26601986
H	-1.09448154	7.44160922	-2.79961790
H	-1.91509972	5.90278033	-2.46609607
H	-1.74809703	7.13322424	-1.19405989

*

TSEF19

Au	-0.06776288	-2.61357975	0.13773977
C	1.21847109	-0.48827569	1.35594091
C	0.00475157	-0.86135945	1.29861923
C	0.44994451	1.56864135	2.38622758
C	1.67039505	1.07310144	2.72195247
H	1.76460004	0.39381927	3.56094789
C	-1.19903587	-0.19613646	1.85304172
H	-1.88117766	-0.90398648	2.32451242
H	-1.73627629	0.27199635	1.02381424
C	-0.76558885	0.88878075	2.89455078
P	-0.25792866	-4.52244544	-1.11809436
C	0.06923684	-4.26365782	-2.87805974
C	0.87116109	-5.84092159	-0.61003828
H	2.23441028	-0.57992366	1.01305106
C	-1.91052015	-5.25341170	-1.04149062
H	-2.64407953	-4.53320540	-1.40827983
H	-1.94785213	-6.15674254	-1.65485259
H	-2.14898510	-5.50539788	-0.00658017
H	-0.63154088	-3.52633112	-3.27374639
H	-0.04612693	-5.20489177	-3.42073494
H	1.08654347	-3.89018421	-3.00829500
H	1.90175275	-5.49370739	-0.70307784
H	0.72402313	-6.72041314	-1.24136831
H	0.67870276	-6.10344458	0.43175051
H	2.58341227	1.51143750	2.33412194
H	-1.60297097	1.57213964	3.03345069
H	-0.54365257	0.39598656	3.84050818
C	0.30727992	2.64445236	1.37112766
C	-0.68883945	3.60586426	1.50702091
C	1.16339284	2.70872765	0.27490168
C	-0.81667994	4.63526377	0.57736328
H	-1.36998441	3.58535940	2.34781897
C	1.02207100	3.70870835	-0.68176101
H	1.94133295	1.96947005	0.13498881
C	0.03409219	4.67375805	-0.51983179
N	-0.11740999	5.73905583	-1.52441610
O	-0.02955695	5.41452516	-2.68963597
O	-0.31908329	6.86157278	-1.11308190
C	-1.86894350	5.59898801	0.77628901
N	-2.73616557	6.32205470	0.98322167
C	1.93593384	3.71138955	-1.79537001
N	2.70523207	3.66393832	-2.64609566

*

TSEF2

Au	-0.13854133	-1.90523774	0.15710636
C	0.93001210	0.24452038	1.43016306
C	-0.26796339	-0.05595566	1.16419954
C	0.04296405	2.47182733	2.11150607
C	1.14689211	2.00923575	2.74908306
H	1.04531359	1.38850600	3.63173113
C	-1.52781799	0.69134185	1.39553156
H	-2.32622642	0.04315799	1.75781943
H	-1.85247444	1.10696421	0.43776776
C	-1.26638985	1.83742639	2.42260885
P	-0.13172984	-3.90833358	-0.95515873
C	0.52332095	-3.79605515	-2.63728197
C	0.85646430	-5.18526247	-0.13998611
H	1.99388285	0.10939663	1.35919909
C	-1.78410063	-4.62188305	-1.13175653
H	-2.42215935	-3.92943142	-1.68373400
H	-1.72473163	-5.57110845	-1.66944244
H	-2.21558240	-4.78929588	-0.14323158
H	-0.07823109	-3.09184130	-3.21476457
H	0.49429104	-4.77847375	-3.11453482
H	1.55347916	-3.43697605	-2.60369169
H	1.89341100	-4.85214043	-0.06855818
H	0.81022123	-6.11426613	-0.71316839
H	0.46999425	-5.35750883	0.86613617
H	2.13467716	2.39755474	2.52794494
H	-2.09893949	2.53780399	2.35949840
H	-1.24085344	1.40902898	3.42458293
C	0.13310469	3.43146856	0.98347892
C	-0.90763444	4.32447350	0.72285764
C	1.26048902	3.45886707	0.15730781
C	-0.82374603	5.23426704	-0.32211888
H	-1.79084717	4.33517262	1.34942403
C	1.35280261	4.35352547	-0.89621978
H	2.07075863	2.75829686	0.32049736
C	0.30664267	5.23627562	-1.12206741
H	-1.63015661	5.93223211	-0.51084468
H	2.22469363	4.36148734	-1.53884800
Cl	0.41549104	6.36680678	-2.44509681

*

TSEF20

Au	-0.13666774	-3.27873258	-0.02821514
C	-1.72377230	-1.16927320	0.76634070
C	-1.30325379	-1.56012970	-0.36588819
C	-2.51951038	0.85511006	-0.34155573
C	-3.19473253	0.38623853	0.74033135
H	-4.00814668	-0.31701114	0.60611473
C	-1.48711889	-0.93795662	-1.69944941
H	-1.71675899	-1.67513694	-2.46920467
H	-0.54606334	-0.45645253	-1.97884152
C	-2.63481079	0.12224078	-1.62660818
P	1.16046746	-5.15095957	0.23113806
C	2.41350832	-4.98514462	1.52504687
C	0.21415068	-6.62898947	0.66792278
H	-1.70676223	-1.22676383	1.84046262
C	2.07531904	-5.59373935	-1.26487052
H	2.73145834	-4.76868149	-1.54789875
H	2.67335992	-6.48986870	-1.08356221
H	1.37318434	-5.78261558	-2.07889208
H	3.07217556	-4.14758734	1.28827161
H	3.00135532	-5.90347439	1.59398807
H	1.92536459	-4.79181831	2.48197761
H	-0.30992292	-6.45982124	1.61031225
H	0.88581192	-7.48435171	0.77225188
H	-0.52004288	-6.83482581	-0.11304650
H	-3.10506456	0.85905873	1.71197466
H	-2.54265985	0.77540578	-2.49380095
H	-3.59212024	-0.39619121	-1.67066640
C	-1.52329772	1.95035339	-0.21772354
C	-0.77259334	2.10119048	0.94552614
C	-1.32604534	2.84372707	-1.26540516
C	0.15699056	3.12066697	1.06857722
H	-0.89279619	1.40400842	1.76326395
C	-0.40627597	3.87723465	-1.15850718
H	-1.90769013	2.75750285	-2.17283922
C	0.33162215	4.00346640	0.00964991
C	0.95172526	3.22752063	2.35589972
C	-0.24177635	4.82430889	-2.33178790
N	1.32814032	5.09230228	0.12857552
F	0.97337607	2.05784623	3.01461558
F	0.42779588	4.13992450	3.19227588
F	2.23234305	3.57542000	2.13040113
F	-1.33393810	4.82707879	-3.11172412
F	0.79798035	4.47756171	-3.11062637
F	-0.03972216	6.09350007	-1.93229925
O	1.09601686	5.96348872	0.93740525
O	2.30008469	5.02691860	-0.59151572

*

TSEF21

Au	-0.46329515	-2.34349510	0.10910390
C	0.62987446	-0.25671820	1.53917246
C	-0.55842780	-0.54669563	1.20130454
C	-0.22298537	1.90337876	2.29902689
C	0.84362483	1.35751453	2.93918908
H	0.68744958	0.70437301	3.78962641
C	-1.81071590	0.21547668	1.42695949
H	-2.63525311	-0.42923698	1.73228611
H	-2.09471854	0.68296743	0.48026377
C	-1.56555551	1.30943527	2.51687430
P	-0.46832509	-4.29244551	-1.09813612
C	-1.13067932	-4.09794456	-2.76983535
C	1.17755176	-5.01032573	-1.31694572
H	1.69067952	-0.42030066	1.46767393
C	-1.46258430	-5.59935947	-0.33980037
H	-2.49596356	-5.26104499	-0.24463542
H	-1.42892768	-6.49979589	-0.95763109
H	-1.07121480	-5.82404959	0.65403255
H	-2.15933313	-3.73719172	-2.71417614
H	-1.10793441	-5.05673620	-3.29313277
H	-0.52950855	-3.36953496	-3.31692424
H	1.81894826	-4.29737868	-1.83812006
H	1.10733033	-5.93191102	-1.89963631
H	1.61209896	-5.22883809	-0.33979823
H	1.85019054	1.72561372	2.77519550
H	-2.36768849	2.04295710	2.44125984
H	-1.60439982	0.83761525	3.49831769
C	-0.04718238	2.94348356	1.25176503
C	-1.01420483	3.93501591	1.09291737
C	1.08404980	2.93416489	0.43414693
C	-0.82806551	4.91274118	0.13801836
H	-1.90213157	3.97942748	1.70942821
C	1.22826694	3.90577210	-0.53041231
H	1.84876539	2.17326224	0.52003432
C	0.28366080	4.91153357	-0.69141872
F	-1.75631938	5.86186952	-0.01549399
F	2.31298402	3.89881590	-1.31178705
N	0.45468625	5.94239150	-1.71693362
O	0.67846111	5.56712639	-2.84950245
O	0.36042762	7.09948899	-1.36308473

*

TSEF22

Au	-0.11538415	-2.62136218	0.05014085
C	1.07704101	-0.75559836	1.52910839
C	-0.15113256	-0.97550846	1.38159069
C	0.27424539	1.38351034	2.70148712
C	1.44281410	0.85141744	3.13828599
H	1.43636749	0.07685535	3.89635488
C	-1.37033374	-0.30045255	1.88576737

H	-2.11752408	-1.01994404	2.22231167
H	-1.80865461	0.26621933	1.05996702
C	-0.97941748	0.65198503	3.05514169
P	-0.24409305	-4.39867855	-1.38846600
C	0.56344230	-4.09917887	-2.97856115
C	0.51147857	-5.90887626	-0.74176633
H	2.13017740	-0.85491366	1.34454654
C	-1.94771846	-4.84975602	-1.79420155
H	-2.44781526	-3.99884811	-2.26017582
H	-1.95389013	-5.69843436	-2.48217748
H	-2.48123425	-5.11701399	-0.88029214
H	0.10389616	-3.23548140	-3.46233530
H	0.45828227	-4.97670738	-3.62084445
H	1.62245784	-3.89142451	-2.81537092
H	1.56850807	-5.72849884	-0.53823599
H	0.41243034	-6.71636912	-1.47107579
H	0.01545460	-6.19360810	0.18788431
H	2.40435427	1.28629508	2.89136522
H	-1.82125700	1.31877687	3.23988526
H	-0.81308806	0.05210263	3.95024275
C	0.20740734	2.50484653	1.74653368
C	-0.96750834	3.24163092	1.55897534
C	1.31637355	2.87557306	0.97176000
C	-1.03666085	4.30309925	0.67533661
H	-1.85600901	3.00372626	2.13178635
C	1.26836752	3.92243211	0.07561579
H	2.24000515	2.31295469	1.04960286
C	0.08511081	4.67770956	-0.09307005
H	-1.96421634	4.85359947	0.58472435
H	2.14602619	4.15292435	-0.51476877
N	0.03059770	5.71459624	-0.96624620
C	-1.17813399	6.49432467	-1.21995871
C	1.19558629	6.24006731	-1.67592709
H	-2.02476273	5.83975941	-1.44221129
H	-1.43966295	7.10382248	-0.34539755
C	-0.78893715	7.36674342	-2.41190839
H	2.04923957	6.34254441	-1.00106150
H	1.48753006	5.57010463	-2.49415115
C	0.71025496	7.58979280	-2.20417019
H	-0.96027126	6.81793877	-3.34225519
H	-1.36152447	8.29438856	-2.44823654
H	0.87127038	8.36156958	-1.44627803
H	1.23191546	7.88768967	-3.11459371

*

TSEF23

Au	-0.03367014	-2.45329406	0.13747095
C	1.20753243	-0.36770933	1.25627011
C	-0.02017477	-0.63522963	1.22217212
C	0.44683851	1.90510265	2.17793251
C	1.62138849	1.38666401	2.61607357
H	1.63403201	0.71613502	3.46748424
C	-1.22548139	0.07367696	1.70432415
H	-1.89699739	-0.60213789	2.23523920
H	-1.76888476	0.44518779	0.83291584
C	-0.80826227	1.24764236	2.65066061
P	-0.20851267	-4.41639850	-1.02948259
C	0.62551707	-4.37602524	-2.63368702
C	0.49031652	-5.83969506	-0.16013803
H	2.24634051	-0.47901947	1.00688177
C	-1.92184790	-4.86694712	-1.39224135
H	-2.38694976	-4.07777507	-1.98569840
H	-1.94934843	-5.80660041	-1.94888561
H	-2.47503820	-4.97993610	-0.45818512
H	0.20042884	-3.57781548	-3.24473871
H	0.49734154	-5.33401988	-3.14325802
H	1.68913921	-4.18074298	-2.48584297
H	1.55118078	-5.66566954	0.02836753
H	0.36988330	-6.74116013	-0.76556181
H	-0.02225578	-5.96996956	0.79461777
H	2.58047889	1.74312651	2.25967133
H	-1.64221706	1.94719398	2.71385176
H	-0.63424037	0.82689238	3.64106780
C	0.38311087	2.96812621	1.15308793
C	-0.79915560	3.21513275	0.44579764
C	1.49090727	3.76100711	0.84806620
C	-0.83468408	4.18977507	-0.52192408
H	-1.70201748	2.64225035	0.61250446
C	1.42921068	4.75252131	-0.11859788
H	2.41893296	3.63198583	1.39112135
C	0.25768523	5.00720269	-0.84249765
H	2.30946805	5.35308872	-0.30498293
N	0.16383394	5.97031646	-1.85316345
C	1.42026803	6.55605932	-2.29802189
C	-0.86034670	6.99965295	-1.65803601
H	1.22067223	7.14798794	-3.19298976
H	1.87376557	7.21634420	-1.54479444
H	2.13319808	5.77154561	-2.55649282
H	-0.55567772	7.72077001	-0.88628488
H	-1.00253869	7.53704457	-2.59758866
H	-1.81015720	6.55792965	-1.36777495
F	-1.98992732	4.33334730	-1.22220809

*

TSEF24

Au	-0.19464141	-2.81881920	0.10196986
C	-0.05721744	-0.69155283	1.81918181
C	-0.90557079	-1.02035339	0.93799112
C	-1.23886419	1.46499030	1.96407761
C	-0.63157525	0.98604342	3.07912716
H	-1.17472735	0.33804282	3.75694957
C	-2.11806338	-0.30514654	0.47156853
H	-2.96483982	-0.97879324	0.33758877
H	-1.89404050	0.13653787	-0.50321219
C	-2.48960038	0.81235046	1.49892215
P	0.49603935	-4.76650690	-0.88885758
C	2.17693529	-4.67591761	-1.55081707
C	0.49719402	-6.18814825	0.22905878
H	0.88847401	-0.81260060	2.31643056
C	-0.54108293	-5.25791939	-2.28652368
H	-0.52658259	-4.47162847	-3.04346417
H	-0.16615464	-6.18820718	-2.71954756
H	-1.56785882	-5.40209194	-1.94549755
H	2.23196174	-3.87857845	-2.29422064
H	2.44673456	-5.62739541	-2.01516831
H	2.87501306	-4.45407755	-0.74163950
H	1.16741445	-5.99098339	1.06761715
H	0.83238963	-7.08098232	-0.30405989
H	-0.51176587	-6.34963389	0.61285134
H	0.30897110	1.39498299	3.43133067
H	-3.16789612	1.50738466	1.00436683
H	-3.00373286	0.35411529	2.34352585
C	-0.58446500	2.47595456	1.09496372
C	-1.34513361	3.42056056	0.41435334
C	0.80478004	2.49688272	0.94753549
C	-0.72537073	4.37585358	-0.38391642
H	-2.42338583	3.43525952	0.51843443
C	1.40622343	3.44636284	0.14074199
H	1.41467873	1.75723833	1.45022965
C	0.64944987	4.39797742	-0.53445207
C	-1.59433348	5.38860878	-1.08347408
C	2.89970800	3.41948340	-0.05882130
H	1.12731780	5.13740645	-1.16393894
F	-0.88622585	6.25562397	-1.82614606
F	-2.31683041	6.11720893	-0.20508187
F	-2.48396062	4.79549802	-1.90948753
F	3.54356157	2.84697375	0.97604626
F	3.41384150	4.65428699	-0.21228854
F	3.24224333	2.71809600	-1.16347891

*

TSEF3

Au	0.00027723	-2.11820041	0.23481171
C	1.29527326	0.16906184	0.96302714
C	0.20820070	-0.34460440	1.35572496
C	0.64795104	2.16961088	2.28506610
C	1.96842549	1.89424565	2.14015440
H	2.46933405	1.24384631	2.84778744
C	-0.79430745	0.15525651	2.32734359
H	-1.15673657	-0.63615341	2.98394627
H	-1.65204265	0.53060338	1.76266375
C	-0.16267572	1.30191728	3.17936440
P	-0.33400433	-4.06687649	-0.92320041
C	-0.04435061	-3.90901105	-2.70158548
C	0.73915682	-5.41831323	-0.38191489
H	2.14101682	0.22535011	0.30172019
C	-2.01939939	-4.70398229	-0.76836686
H	-2.72458013	-3.96376674	-1.15063528
H	-2.11988896	-5.63155897	-1.33670142
H	-2.24137571	-4.89453441	0.28317242
H	-0.71885113	-3.15690372	-3.11471762
H	-0.22107849	-4.86825657	-3.19392913
H	0.98606181	-3.59388851	-2.87561120
H	1.78315848	-5.13024661	-0.51783484
H	0.52935564	-6.31751221	-0.96597917
H	0.56299926	-5.62094437	0.67601555
H	2.59415149	2.46299161	1.46132798
H	-0.97521549	1.84177552	3.66502412
H	0.47520607	0.85782265	3.94352806
C	-0.04102420	3.16954537	1.43080148
C	-1.11588902	3.91250734	1.92722815
C	0.37250013	3.38529963	0.11798557
C	-1.74951406	4.85832592	1.13496304
H	-1.44927458	3.77006639	2.94799708
C	-0.27582197	4.32431688	-0.67282280
H	1.18779315	2.80638675	-0.29991022
C	-1.33615798	5.06861659	-0.17434569
H	-2.57338285	5.43517703	1.53889031
H	-1.83644368	5.80001302	-0.79651713
C	0.19726769	4.49947634	-2.09069564
F	0.07969406	3.35568343	-2.80455378
F	1.50363759	4.84512536	-2.14736747
F	-0.48590127	5.44640998	-2.75701422

*

TSEF4

Au	0.28007525	-2.69454650	0.49306748
C	1.33396066	-0.38266955	1.50143652
C	0.14454616	-0.80429774	1.41679218
C	0.35690042	1.78254853	2.21318421
C	1.57616152	1.44193930	2.70046413
H	1.65482689	0.86210576	3.61282060
C	-1.13366385	-0.15217372	1.79178173
H	-1.81537974	-0.84361375	2.28769273
H	-1.62097928	0.18534107	0.87306239
C	-0.83821930	1.06278144	2.72692596
P	0.29188027	-4.75740619	-0.50494691
C	1.94058162	-5.49047076	-0.63188791
C	-0.70881988	-5.97903848	0.37656014
H	2.38461208	-0.43616710	1.28055301
C	-0.35426600	-4.73765480	-2.19399807
H	0.25343631	-4.07032628	-2.80771040
H	-0.32850167	-5.74557574	-2.61481333
H	-1.38262804	-4.37198095	-2.18570848
H	2.58581780	-4.83436244	-1.21876123
H	1.87734658	-6.46826311	-1.11531715
H	2.36594045	-5.60430676	0.36684286
H	-0.32528844	-6.09972255	1.39128430
H	-0.67058473	-6.93829900	-0.14494880
H	-1.74277375	-5.63310988	0.42799888
H	2.48411239	1.90011662	2.32446221
H	-1.72859583	1.69063781	2.74926051
H	-0.64207609	0.68931706	3.73198005
C	0.20604119	2.70090414	1.05602756
C	-0.91070871	3.53520868	0.95925063
C	1.17276180	2.74786275	0.05031601
C	-1.04830248	4.40690851	-0.10829479
H	-1.66998529	3.52466161	1.73113071
C	1.03494922	3.61101147	-1.02624211
H	2.03343295	2.09139882	0.09282728
C	-0.07467105	4.44160380	-1.10013678
H	-1.91295991	5.05847368	-0.16816526
H	1.78905643	3.62976207	-1.80306977
C	-0.26229248	5.38293981	-2.25870643
F	-1.34065725	5.05113288	-3.00569001
F	-0.46472226	6.65418931	-1.84708971
F	0.79472506	5.40537214	-3.09071291

*

TSEF5

Au	-0.11293908	-1.64303456	0.18304449
C	0.98591153	0.57940174	1.24374929
C	-0.22332735	0.27576083	1.06066771
C	0.10235962	2.87954855	1.79704875
C	1.23752303	2.48376682	2.42388514
H	1.18100050	1.92383675	3.35019430
C	-1.47814859	1.02996714	1.29465187
H	-2.25012831	0.40265332	1.74138499
H	-1.85284023	1.37024496	0.32551504
C	-1.17914811	2.24781663	2.22147722
P	-0.15228121	-3.72119777	-0.77972424
C	0.49215173	-3.74472986	-2.46927194
C	0.81409414	-4.95642676	0.12081650
H	2.04838581	0.45081292	1.15011145
C	-1.82136777	-4.40814479	-0.89428534
H	-2.44778923	-3.74314090	-1.49146528
H	-1.78697993	-5.39492954	-1.36190206
H	-2.24909765	-4.49344765	0.10627797
H	-0.09603038	-3.06798054	-3.09161960
H	0.43506437	-4.75728049	-2.87566750
H	1.53111733	-3.41037454	-2.46716858
H	1.85765780	-4.63984658	0.16568182
H	0.74703389	-5.92242141	-0.38523652
H	0.42822610	-5.04934023	1.13754614
H	2.21126513	2.86410302	2.13735787
H	-2.02656544	2.93006546	2.16100585
H	-1.09172614	1.89118317	3.24793706
C	0.11982282	3.74013219	0.59242804
C	-0.98812052	4.51846910	0.24283550
C	1.24481360	3.79508578	-0.23712821
C	-0.97159463	5.33656247	-0.87499329
H	-1.87843289	4.50966622	0.85978490
C	1.27149852	4.59895255	-1.36345311
H	2.11053877	3.18129725	-0.01667343
C	0.16047791	5.37856859	-1.68317826
H	-1.83131518	5.94599079	-1.12969196
H	2.14933134	4.61939494	-2.00214534
O	0.13088938	6.18846977	-2.77476186
H	0.97838355	6.13203747	-3.23872719

*

TSEF6

Au	-0.03211209	-1.91078099	0.17728873
C	1.18958618	0.26271613	1.20799419
C	-0.02558704	-0.07103422	1.21567830
C	0.36356689	2.47690985	2.10511783
C	1.60334647	2.07582653	2.47940260
H	1.73063816	1.43915343	3.34733326
C	-1.23545483	0.61425197	1.72982487
H	-1.90224146	-0.07560638	2.24798968
H	-1.78321598	1.01845247	0.87424441
C	-0.80516885	1.76321067	2.69268830
P	-0.19592726	-3.91076155	-0.92773557

C 0.77380766 -3.97859131 -2.45269324
C 0.35462571 -5.32530972 0.05550808
H 2.22079273 0.18142238 0.91810961
C -1.89147112 -4.30188599 -1.42035810
H -2.26824539 -3.51867171 -2.08055706
H -1.91591614 -5.26170099 -1.94161382
H -2.52549913 -4.35229114 -0.53336822
H 0.44142523 -3.18983392 -3.12997653
H 0.64406290 -4.95138112 -2.93294990
H 1.82897362 -3.82431877 -2.22004239
H 1.40238118 -5.19186953 0.33063889
H 0.24308619 -6.24615979 -0.52177356
H -0.24443684 -5.39016859 0.96554274
H 2.49820450 2.51842263 2.05684701
H -1.66625684 2.41488106 2.83830984
H -0.52875085 1.32828733 3.65337592
C 0.13888574 3.43496102 0.99945947
C -1.01893267 4.22187944 0.94776474
C 1.06819159 3.57862383 -0.03107909
C -1.22535366 5.12506937 -0.07694336
H -1.76482899 4.14857957 1.73014427
C 0.87100549 4.47259167 -1.07464083
H 1.96096580 2.96362089 -0.04221769
C -0.28197446 5.25777455 -1.09910792
H -2.11771835 5.74049043 -0.10276592
H 1.61211370 4.54163394 -1.85972940
O -0.57279525 6.16100587 -2.06548911
C 0.37363189 6.32987800 -3.12048484
H 1.33876164 6.66772536 -2.73231039
H 0.50402126 5.40169726 -3.68431495
H -0.04241729 7.09535030 -3.77399803
*

TSEF7
Au -0.04641636 -1.86939228 0.14645921
C 1.15412012 0.33721594 1.17198354
C -0.05801157 -0.01385345 1.15183133
C 0.31081640 2.53188735 2.04148044
C 1.54149506 2.12709053 2.44215703
H 1.64886172 1.49769054 3.31804031
C -1.28071718 0.67122771 1.63575901
H -1.95825189 -0.01619259 2.14298744
H -1.80976560 1.07001864 0.76597772
C -0.87478989 1.82719793 2.60178346
P -0.18395862 -3.89189141 -0.92100314
C 0.79573493 -3.98081413 -2.43851608
C 0.37543226 -5.28045124 0.09393549
H 2.18859258 0.25684219 0.89261263
C -1.87220104 -4.31135415 -1.41557386
H -2.25339049 -3.54579247 -2.09370533
H -1.88318736 -5.28171543 -1.91740957
H -2.51103154 -4.35079582 -0.53147116
H 0.45783684 -3.21116889 -3.13478246
H 0.68081327 -4.96534158 -2.89817578
H 1.84750902 -3.80848997 -2.20320213
H 1.42027071 -5.13073527 0.37178040
H 0.27645062 -6.21403900 -0.46489165
H -0.22785313 -5.33292558 1.00199588
H 2.44548662 2.56639851 2.03597546
H -1.73720950 2.48300738 2.71708414
H -0.62596798 1.39874372 3.57278346
C 0.12059110 3.49890546 0.93417669
C -1.01097154 4.31244305 0.88181657
C 1.06892383 3.61790836 -0.08985812
C -1.17698841 5.22865557 -0.14788509
H -1.76413112 4.25371766 1.65821967
C 0.89626917 4.52343270 -1.11976799
H 1.94149311 2.97472064 -0.09486535
C -0.22942771 5.35396163 -1.16325721
H -2.05922240 5.86069883 -0.16371089
H 1.64241045 4.57510039 -1.90422649
C -0.45571176 6.34562111 -2.23469214
C 0.40118699 6.67364079 -3.19811970
H -1.42145883 6.84451163 -2.19721618
H 0.13569707 7.42138101 -3.93798561
H 1.38504382 6.22386111 -3.28623206
*

TSEF8
Au -0.12721225 -1.80549407 0.13950205
C 0.93931383 0.37664934 1.36229295
C -0.25936098 0.05460493 1.12555970
C 0.03344084 2.59449281 2.04603431
C 1.15874717 2.15186996 2.66042250
H 1.08712233 1.53663084 3.54987245
C -1.52517957 0.78477570 1.37797533
H -2.30524866 0.12780062 1.76360699
H -1.87726537 1.18686878 0.42417042
C -1.25932151 1.94361240 2.38973126
P -0.12069728 -3.82388351 -0.94464634
C 0.53895890 -3.73692917 -2.62648485
C 0.86198912 -5.09168869 -0.10874005
H 2.00313003 0.25701592 1.26640933
C -1.77459586 -4.53558726 -1.11553213
H -2.40804497 -3.85064025 -1.68212638
H -1.71613654 -5.49411394 -1.63654702
H -2.21083505 -4.68447678 -0.12609955
H -0.05491538 -3.03426976 -3.21376552
H 0.50252636 -4.72463008 -3.09219234
H 1.57221859 -3.38646775 -2.59444685
H 1.89842234 -4.75770114 -0.03320724
H 0.81991033 -6.02690152 -0.67203314
H 0.46803400 -5.25296523 0.89633861
H 2.13500890 2.55356812 2.41352976
H -2.10303643 2.63133989 2.33775565
H -1.20665489 1.52394762 3.39435842
C 0.08411133 3.55139442 0.91333537
C -0.97105324 4.43637684 0.68284660
C 1.18706817 3.58521891 0.05446860
C -0.92132986 5.34210814 -0.36511024
H -1.83428603 4.44016202 1.33674283
C 1.23786368 4.47873471 -1.00052910
H 2.00620090 2.89035968 0.19676151
C 0.18268413 5.36917778 -1.21796801
H -1.74134437 6.03211206 -0.52608230
H 2.09343719 4.48809108 -1.66575125
C 0.23157750 6.30288576 -2.31263080
C 0.26828609 7.08588044 -3.22230455
H 0.30205058 7.78167824 -4.03383777
*

TSEF9
Au -0.13836329 -1.79976586 0.14909686
C 0.92854537 0.39745415 1.37614435
C -0.26630885 0.06341639 1.12591927
C 0.02901542 2.60200382 2.03119481
C 1.14483694 2.15401839 2.66033817
H 1.05797631 1.55157457 3.55706656
C -1.53301601 0.79805188 1.36185563
H -2.31790736 0.14717068 1.74795340
H -1.87693982 1.19159748 0.40151510
C -1.27329774 1.96736845 2.36420635
P -0.12611303 -3.82051536 -0.93158092
C 0.53191323 -3.73258209 -2.61406283
C 0.86259005 -5.08344642 -0.09545258
H 1.99204803 0.27159311 1.28113220
C -1.77686895 -4.53968246 -1.10081053
H -2.41512006 -3.85644489 -1.66394090
H -1.71489640 -5.49649144 -1.62456632
H -2.20994844 -4.69349580 -0.11077584
H -0.06765361 -3.03570344 -3.20239630
H 0.50242144 -4.72148775 -3.07768815

H	1.56250361	-3.37443668	-2.58371521
H	1.89809089	-4.74599767	-0.02344864
H	0.82209526	-6.01997531	-0.65669500
H	0.47193438	-5.24393084	0.91101115
H	2.12659540	2.54645105	2.42040122
H	-2.11007245	2.66163853	2.29233225
H	-1.23717439	1.55920843	3.37412752
C	0.10470091	3.55443373	0.89424403
C	-0.93074931	4.46292323	0.66546251
C	1.21109511	3.55517846	0.03907339
C	-0.86117827	5.36460491	-0.38405435
H	-1.79279547	4.48711738	1.31983173
C	1.28684528	4.44316195	-1.01828138
H	2.01230540	2.84135539	0.18590378
C	0.24785368	5.35074086	-1.22662708
H	-1.66167850	6.07499417	-0.55151151
H	2.14093057	4.43416206	-1.68471598
C	0.31974908	6.27724071	-2.32672553
N	0.37676048	7.01681279	-3.20557662

*

TSFG1

C	-2.12599860	-1.11588994	0.50011660
H	-1.89571431	-2.01467781	-0.07047112
H	-3.16972127	-0.86123668	0.27400636
C	-1.26236643	0.05831559	0.09400046
C	-1.21976640	1.05373128	1.00215413
H	-0.52548077	1.88489252	0.98107946
C	-2.22151920	1.05261720	2.19736526
H	-2.12335646	1.96085850	2.77965170
H	-3.24029736	0.86808240	1.86146924
C	-1.51420693	-0.11324144	2.67411384
C	-1.91106614	-1.38374030	1.99761400
H	-1.17003076	-2.16616758	2.14325453
H	-2.83361380	-1.71439142	2.49256007
Au	-0.26990323	0.10985548	-1.70527461
P	0.83777536	0.15335276	-3.73171556
C	0.26714741	1.45629004	-4.85369194
C	2.62341983	0.42206680	-3.58613776
C	0.67870391	-1.37855342	-4.68554096
H	-0.37636331	-1.57168334	-4.88896619
H	1.22194871	-1.29548238	-5.62984010
H	1.08219217	-2.21091041	-4.10574208
H	-0.79544367	1.31748149	-5.06196275
H	0.82922069	1.42190976	-5.78998976
H	0.40754494	2.43014071	-4.38070248
H	2.80784992	1.37741174	-3.09108940
H	3.08627154	0.42954282	-4.57581472
H	3.06340728	-0.37579687	-2.98484273
C	-0.51603624	-0.05351577	3.72455413
C	0.49522526	-1.02080521	3.82130540
C	-0.55678834	0.97012245	4.68742685
C	1.43276509	-0.95935814	4.83645168
H	0.57225827	-1.80699915	3.08110863
C	0.37260119	1.01311158	5.70682200
H	-1.33972675	1.71718484	4.65442329
C	1.38548956	0.05281404	5.79620915
H	2.21763033	-1.70630616	4.88695262
H	0.31714114	1.80071173	6.45120145
C	2.39288300	0.11673836	6.90831871
H	3.13778563	-0.67461318	6.81497378
H	2.90536135	1.08249131	6.90601614
H	1.89620764	0.01427997	7.87730300

*

TSFG12

C	-1.36933392	2.18827347	-0.14096465
H	-2.25576780	1.79925708	-0.64026985
H	-1.22905426	3.21379273	-0.50692492
C	-0.12524179	1.39734714	-0.48131963
C	0.89417864	1.57374439	0.38487843
H	1.78831212	0.96353079	0.42179397
C	0.82912370	2.72282066	1.43607907
H	1.76072832	2.79189415	1.98473318
H	0.52542079	3.66416550	0.98245096
C	-0.23634069	1.95785459	2.03979863
C	-1.56591652	2.14132452	1.38162443
H	-2.25994976	1.34923314	1.65275553
H	-1.97519923	3.08085399	1.77432411
Au	-0.02697590	0.17596829	-2.13106651
P	0.07277971	-1.19235534	-3.98964328
C	1.41385435	-0.77439821	-5.13371413
C	0.33265500	-2.94140591	-3.59538929
C	-1.42559989	-1.16753603	-5.00739680
H	-1.60848465	-0.15081910	-5.36050294
H	-1.31086756	-1.83536958	-5.86441112
H	-2.27847683	-1.48957590	-4.40684587
H	1.28262509	0.24961923	-5.48867031
H	1.41150116	-1.45817142	-5.98590963
H	2.37068584	-0.84524345	-4.61292262
H	1.26962534	-3.05450432	-3.04670373
H	0.37417413	-3.53347795	-4.51268102
H	-0.48659873	-3.29871842	-2.96859550
C	-0.03819373	1.10305912	3.19403779
C	-0.88068246	0.00498562	3.43784217
C	0.99129704	1.36669019	4.11054231
C	-0.69465386	-0.79787952	4.54403240
H	-1.66655085	-0.24356433	2.73578459
C	1.16069080	0.57001752	5.22669564
H	1.64378826	2.21904488	3.96845543
C	0.32766349	-0.52783291	5.46297225
H	-1.35167130	-1.64714308	4.69336855
H	1.95045623	0.79886598	5.93406962
C	0.55045132	-1.36187456	6.66698668
C	-0.61914182	-1.98316666	7.39646891
C	0.31250350	-2.85439891	6.62128375
H	1.37581509	-1.03092922	7.28583416
H	-1.60613914	-1.80229496	6.98712322
H	-0.56411409	-2.00492132	8.47807322
H	1.01039498	-3.47639331	7.16852615
H	-0.03870479	-3.26866225	5.68350113

*

TSFG16

C	-1.43437191	2.02051320	0.23427122
H	-2.31409023	1.72685837	-0.33675045
H	-1.24725260	3.07700048	0.00122643
C	-0.20588599	1.23107670	-0.15719919
C	0.78278252	1.26818931	0.76430800
H	1.64944018	0.61871795	0.76447068
C	0.72815390	2.30357892	1.92408098
H	1.64652151	2.28640977	2.49791875
H	0.45520598	3.29627162	1.57358534
C	-0.36399669	1.48273883	2.38730041
C	-1.67644358	1.79233707	1.73307207
H	-2.39741054	0.99194936	1.88132425
H	-2.07169393	2.68493168	2.23309452
Au	-0.08045895	0.19808188	-1.92767850
P	0.04989987	-0.95414729	-3.92623360
C	1.52692344	-0.56530097	-4.90010015
C	0.08509029	-2.75503015	-3.73326821
C	-1.33729878	-0.63652245	-5.04712253
H	-1.37848923	0.42902003	-5.28096756
H	-1.21571341	-1.20738626	-5.97072214
H	-2.27104939	-0.92573072	-4.56104544
H	1.53790949	0.50121391	-5.13295436
H	1.52969354	-1.14074532	-5.82885693
H	2.41922168	-0.80705802	-4.31950866
H	0.95032439	-3.03932875	-3.13130440
H	0.14739183	-3.23816446	-4.71124906
H	-0.82172448	-3.08393627	-3.22211332
C	-0.21645263	0.48656791	3.43156222

C	-1.09934118	-0.59672330	3.52572219
C	0.82130483	0.60066594	4.37673088
C	-0.95321253	-1.54289679	4.52381558
H	-1.88766080	-0.72716473	2.79634918
C	0.95700346	-0.33192562	5.37825066
H	1.50757733	1.43903944	4.34935553
C	0.06858904	-1.41778645	5.45611782
H	-1.62419827	-2.39119506	4.59307698
O	1.91660657	-0.29157689	6.34834464
O	0.19613813	-2.34007315	6.43034986
H	2.48145274	0.48416263	6.22455021
H	0.95268470	-2.11016086	6.99205190

*

TSFG17

C	-1.52288982	2.07252597	-0.27558847
H	-2.35967744	1.60262299	-0.79043196
H	-1.48561481	3.11410609	-0.62061717
C	-0.20067638	1.42018166	-0.61139036
C	0.78298476	1.69753600	0.27465747
H	1.73095192	1.17612467	0.32263294
C	0.60027157	2.83757783	1.31617857
H	1.51976688	3.00585011	1.86326181
H	0.18405607	3.73878291	0.87224309
C	-0.35539388	1.92105207	1.88711501
C	-1.70953689	1.97255219	1.24454017
H	-2.30311398	1.09624199	1.49512717
H	-2.22335545	2.84651944	1.66246716
Au	0.04406131	0.21411237	-2.25502292
P	0.30278526	-1.13349320	-4.11429830
C	0.34245051	-0.21439013	-5.67495271
C	1.83010982	-2.10724253	-4.11326497
C	-1.02018096	-2.35222662	-4.32979037
H	-1.97826656	-1.83682083	-4.42035608
H	-0.84144354	-2.94909805	-5.22729515
H	-1.05528993	-3.00814985	-3.45784883
H	-0.58952948	0.34170771	-5.79293259
H	0.46563985	-0.90140039	-6.51550476
H	1.17364265	0.49312681	-5.65838526
H	2.68927167	-1.43522226	-4.06882903
H	1.89058482	-2.71627550	-5.01829646
H	1.84651682	-2.75675835	-3.23595991
C	-0.05106526	1.06969772	3.02093781
C	-0.75100322	-0.14116547	3.21883434
C	0.91716701	1.44843284	3.94850824
C	-0.48026308	-0.94372187	4.30299172
H	-1.48215329	-0.45987229	2.49059379
C	1.18446005	0.65638474	5.05740185
H	1.45029418	2.38380273	3.83827376
C	0.50244932	-0.53920702	5.24633354
O	-1.08248840	-2.12822738	4.56267549
H	1.92576549	0.98120628	5.77446381
O	0.69510819	-1.37604314	6.27667767
C	-2.08145397	-2.56484063	3.64323121
C	1.67533357	-1.01520851	7.25476967
H	-2.44226532	-3.51809978	4.02671138
H	-2.91087173	-1.85271308	3.59672376
H	-1.66004184	-2.70956418	2.64381933
H	1.40922582	-0.07391296	7.74271877
H	1.66990170	-1.82120297	7.98648232
H	2.66673419	-0.93674515	6.80117436

*

TSFG18

C	-1.21124408	2.38019188	-0.28933462
H	-2.10927835	2.11479211	-0.84520077
H	-0.91297072	3.38129257	-0.62870010
C	-0.06838590	1.43695844	-0.56075752
C	0.88202345	1.47022990	0.41964811
H	1.65827331	0.72463663	0.52961568
C	0.99358520	2.68630870	1.37684626
H	1.91840502	2.64890843	1.93801607
H	0.78729277	3.63858937	0.89854101
C	-0.13034764	1.92895031	1.85739953
C	-1.44775830	2.31768606	1.22429105
H	-2.21794291	1.58192026	1.44805595
H	-1.76477204	3.27366284	1.65224395
Au	-0.01262296	0.17387610	-2.17109464
P	0.04448770	-1.24346889	-3.99831669
C	0.78857060	-0.49712769	-5.47120340
C	0.99382030	-2.75944956	-3.71556039
C	-1.58794457	-1.81087148	-4.53919388
H	-2.20182387	-0.94900284	-4.80757184
H	-1.49002395	-2.47170151	-5.40367207
H	-2.07444189	-2.34877339	-3.72330460
H	0.21834567	0.38861776	-5.75754257
H	0.78836961	-1.21302256	-6.29654935
H	1.81460441	-0.19820854	-5.24863096
H	2.02281790	-2.50064526	-3.45897351
H	0.98800193	-3.38177320	-4.61350163
H	0.55363745	-3.31476733	-2.88519920
C	-0.05199797	1.02911485	3.00502395
C	-0.96250215	-0.02578517	3.16265559
C	0.92785594	1.18524622	3.99485467
C	-0.90040617	-0.88493228	4.23732965
H	-1.72651516	-0.19637329	2.41257348
C	1.00018328	0.34410977	5.08517141
H	1.63658352	2.00298665	3.93047203
C	0.09249869	-0.73034075	5.23467346
H	-1.62233742	-1.68687957	4.30560304
H	1.76076293	0.52480700	5.83207942
N	0.16952854	-1.58212771	6.29308779
C	-0.87143031	-2.57551397	6.50321527
C	1.11395679	-1.32283329	7.36756707
H	-0.62707507	-3.15741199	7.38968514
H	-1.85398553	-2.11246475	6.65191765
H	-0.93662380	-3.26353678	5.65591219
H	1.05066778	-2.13177466	8.09278622
H	2.13918510	-1.28865871	6.98965465
H	0.90086336	-0.37777153	7.88123611

*

TSFG22

C	0.23246552	2.26035415	-0.93694796
H	-0.67314623	2.61663632	-1.43155250
H	1.05817873	2.79387160	-1.43048123
C	0.44196029	0.81374339	-1.21386144
C	0.94194334	0.17563323	-0.06553115
H	0.96722168	-0.89782093	0.04227605
C	2.18155895	0.93076087	0.54947160
H	2.84485491	0.23214521	1.04350874
H	2.65186579	1.67662373	-0.07939981
C	0.87369659	1.18175696	1.14070361
C	0.19943587	2.43187929	0.58982859
H	-0.83335860	2.47555861	0.93999123
H	0.71213426	3.33645934	0.92135750
Au	0.03759238	-0.08570111	-2.99155463
P	-0.41674370	-1.09894621	-5.02775427
C	-1.60020909	-0.18055933	-6.04324786
C	1.05030816	-1.29576122	-6.06935627
C	-1.11455192	-2.76138696	-4.87170369
H	-2.04946915	-2.71303433	-4.31026618
H	-1.30540798	-3.18188821	-5.86180691
H	-0.41248652	-3.40075750	-4.33337554
H	-2.54564022	-0.08846590	-5.50541489
H	-1.76784562	-0.70192995	-6.98851618
H	-1.20916542	0.81886936	-6.24268693
H	1.48843511	-0.31633391	-6.27007084
H	0.77718103	-1.77345020	-7.01316480
H	1.78600780	-1.91156903	-5.54878466
C	0.52746816	0.70185239	2.51343049
C	0.54686356	1.61394699	3.57016739

C 0.18182080 -0.61439826 2.80530528
C 0.23884978 1.23541651 4.86548391
H 0.80657627 2.65080803 3.37931584
C -0.12594417 -1.01632227 4.09719327
H 0.14416801 -1.35648413 2.01537872
C -0.10602515 -0.09865620 5.16444494
H 0.25507551 1.98053837 5.65098909
H -0.38508855 -2.05200483 4.27740845
N -0.41973047 -0.48011036 6.43644931
C -0.79283506 -1.84306436 6.79617949
C -0.22066234 0.37436712 7.60425007
H -1.61102472 -2.20372746 6.16692617
H 0.05549894 -2.53075434 6.67565426
C -1.20103201 -1.71595166 8.26227616
H 0.74297210 0.88936716 7.55348899
H -1.00703388 1.13682930 7.66948337
C -0.28763782 -0.60267091 8.77927446
H -2.24727068 -1.40384013 8.32775845
H -1.08654453 -2.65441533 8.80649172
H 0.70931954 -1.00408931 8.98173164
H -0.65804451 -0.12909886 9.68948622
*

TSFG23
C -1.21084708 2.40503409 -0.52321495
H -2.09835784 2.09284099 -1.07167900
H -0.95413890 3.41012297 -0.88302957
C -0.02971132 1.49844955 -0.77605934
C 0.93935158 1.60608705 0.16856781
H 1.75395485 0.90262875 0.28549844
C 0.95894140 2.80850674 1.14794800
H 1.87488654 2.82065460 1.72501773
H 0.72671459 3.75311186 0.66379997
C -0.15992978 2.06561698 1.66904654
C -1.46727992 2.37499514 0.98902724
H -2.22258638 1.62842158 1.22403420
H -1.81948877 3.33572526 1.38029732
Au 0.05220680 0.19005553 -2.35332124
P 0.13565833 -1.28370370 -4.13244758
C 0.88585531 -0.58419452 -5.62542981
C 1.09282904 -2.78344359 -3.79279709
C -1.48767457 -1.88240970 -4.66794976
H -2.10591865 -1.03511015 -4.97063189
H -1.37773330 -2.57154316 -5.50858329
H -1.97728719 -2.39594255 -3.83830843
H 0.31163525 0.28696224 -5.94658280
H 0.89691672 -1.32822102 -6.42546742
H 1.90802242 -0.27002224 -5.40603200
H 2.11870321 -2.50971887 -3.53911438
H 1.09629111 -3.43632614 -4.66879155
H 0.65112364 -3.31282302 -2.94643172
C -0.05983152 1.19000479 2.82150205
C -0.99103152 0.15735976 3.02937845
C 0.95082052 1.34836327 3.77828081
C -0.88249432 -0.66245325 4.11629165
H -1.80771259 -0.02259635 2.34152120
C 1.04546030 0.51724954 4.87530205
H 1.67584451 2.14629397 3.67737016
C 0.14380111 -0.54853151 5.08412897
F -1.85144266 -1.59935387 4.29388836
H 1.84677649 0.67839984 5.58290822
N 0.23287621 -1.37764479 6.16009116
C -0.18088079 -2.78052262 6.08731313
C 1.16159995 -1.05974704 7.23384615
H 0.55614526 -3.38061289 6.62362991
H -1.16075366 -2.94112729 6.54407239
H -0.20448967 -3.12880519 5.05626758
H 0.88779729 -1.65171847 8.10723710
H 2.20057150 -1.29027658 6.96645727
H 1.08827139 -0.00593854 7.50535428
*

TSFG5
C -2.05409614 -1.24613999 0.52807505
H -1.81560436 -2.15001928 -0.03070796
H -3.10393892 -1.00981357 0.31008857
C -1.21104956 -0.06685398 0.10008222
C -1.18239702 0.93784997 1.00558179
H -0.48883890 1.76915849 0.98010191
C -2.20610414 0.96328497 2.17560487
H -2.13413767 1.89204414 2.72793023
H -3.21536450 0.73072647 1.84415010
C -1.42623294 -0.15509108 2.64473233
C -1.80915400 -1.46718867 2.02692309
H -1.03741390 -2.21908916 2.17488986
H -2.70769040 -1.81187517 2.55262803
Au -0.20539586 -0.02167715 -1.68935676
P 0.91312505 0.02554115 -3.71071478
C 0.05514003 0.97320011 -4.99441723
C 2.56439043 0.76462270 -3.61701870
C 1.17971400 -1.60871742 -4.44509005
H 0.21602410 -2.09311556 -4.61384079
H 1.71067053 -1.51382709 -5.39522881
H 1.76670272 -2.22359047 -3.76018515
H -0.92919887 0.53578379 -5.17204376
H 0.63181313 0.96094819 -5.92232967
H -0.07411594 2.00427751 -4.65992565
H 2.48196426 1.79407449 -3.26312195
H 3.03885222 0.75476473 -4.60115044
H 3.17670251 0.19881427 -2.91232084
C -0.41065355 -0.03270151 3.67350329
C 0.63979320 -0.96190090 3.77588591
C -0.46544210 1.01182638 4.61018933
C 1.59517238 -0.85228573 4.76230946
H 0.72830528 -1.76493496 3.05511665
C 0.47726093 1.12365360 5.61137335
H -1.27302138 1.73208335 4.57574318
C 1.51448116 0.19154878 5.68812521
H 2.41199343 -1.56118204 4.83111361
H 0.41489713 1.92533071 6.34017687
O 2.46736939 0.25332918 6.64163896
H 2.30583602 1.01802162 7.21450819
*

TSFG6
C -2.51856098 -0.64347714 0.06527029
H -2.46143226 -1.54606049 -0.54146685
H -3.46888748 -0.15462364 -0.18647935
C -1.39819110 0.32536986 -0.23764412
C -1.20072013 1.24766224 0.73254205
H -0.33245746 1.89147184 0.79893946
C -2.26030166 1.44729895 1.85205533
H -2.01030229 2.30121908 2.46943130
H -3.27451843 1.47343335 1.46092003
C -1.78492246 0.15364701 2.27560722
C -2.42547444 -0.99678060 1.55593641
H -1.85853381 -1.91493029 1.69106744
H -3.40989926 -1.14821522 2.01444406
Au -0.27084733 0.21258890 -1.94992525
P 0.99431131 0.07782492 -3.87903698
C 0.19815989 0.82064142 -5.32669823
C 2.60296884 0.90270470 -3.76293555
C 1.38280300 -1.61781788 -4.38466468
H 0.45550751 -2.16771660 -4.55634642
H 1.97770718 -1.61226026 -5.30106724
H 1.94342941 -2.11432870 -3.59033653
H -0.75261797 0.31871091 -5.51593655
H 0.84244450 0.72309386 -6.20366351
H 0.00494750 1.87741932 -5.13273042
H 2.45390120 1.96031564 -3.53711860
H 3.14610290 0.80476741 -4.70588073
H 3.18635963 0.45062861 -2.95856908

C -0.82278498 -0.02649725 3.34523053
C -0.02169330 -1.18277870 3.41517658
C -0.67679192 0.93788578 4.35105108
C 0.88121503 -1.35776792 4.43571405
H -0.08734181 -1.93753178 2.64150118
C 0.21653353 0.76795690 5.39358144
H -1.29176351 1.82918173 4.34260288
C 1.00666836 -0.38457961 5.43909959
H 1.50887017 -2.24013309 4.48206870
H 0.28759459 1.52568648 6.16177982
O 1.90810355 -0.64698041 6.39955528
C 2.08131749 0.31724728 7.44378468
H 2.85059617 -0.09122558 8.09667938
H 2.41560241 1.27479731 7.03662252
H 1.15441641 0.45034569 8.00738578
*

alt-constrF14
Au 0.02319123 -2.19363348 0.16477367
C 1.34679422 0.16779700 1.43049125
C 0.16863005 -0.39420328 1.17371405
C 0.28415864 2.18425394 2.00921967
C 1.52781652 1.47734407 2.27265541
H 1.60865744 1.20732561 3.32651992
C -1.10303226 0.28161852 1.57641851
H -1.78798058 -0.39712697 2.08811362
H -1.61716633 0.65197939 0.68522160
C -0.88530269 1.52052049 2.55668040
P -0.14493338 -4.20188115 -0.95728083
C 1.01873929 -4.38030322 -2.33479914
C 0.15564687 -5.65528114 0.08248354
H 2.29168988 -0.22575907 1.06966910
C -1.77408309 -4.49561389 -1.69424855
H -1.99940727 -3.70010556 -2.40714917
H -1.78991359 -5.45968135 -2.20814571
H -2.53377649 -4.48929375 -0.91016774
H 0.84876956 -3.58328610 -3.06121373
H 0.88426149 -5.34990176 -2.82021941
H 2.04083261 -4.30048051 -1.95955632
H 1.16042532 -5.59759963 0.50548725
H 0.06161913 -6.56950542 -0.50838358
H -0.56817142 -5.67422947 0.89959423
H 2.42516233 1.99958410 1.95495508
H -1.79708044 2.11022779 2.55956874
H -0.67049304 1.11578709 3.54478973
C 0.18458424 3.28798330 1.07742764
C -0.98134003 4.06881351 1.03126689
C 1.24558363 3.57989130 0.20258830
C -1.08737699 5.12075385 0.13897264
H -1.79946028 3.88058064 1.71215037
C 1.13989750 4.61346009 -0.70681913
H 2.14561347 2.98070707 0.20764928
C -0.02503183 5.36410553 -0.71253685
H -1.97452882 5.73729266 0.10483481
H 1.94290923 4.83642203 -1.39518307
N -0.13662721 6.48652359 -1.67598276
O -1.18879062 7.09144357 -1.71633483
O 0.83058573 6.73306863 -2.36758187
*

alt-constrF15
Au 0.03065875 -2.13347321 0.20060552
C 1.56953727 0.29889221 0.99953242
C 0.37344208 -0.28363566 1.05826042
C 0.65374538 2.35915311 1.66791019
C 1.93131464 1.65861161 1.68471531
H 2.26866695 1.46607130 2.70429918
C -0.77998919 0.40661833 1.71842443
H -1.29119823 -0.24599861 2.42891785
H -1.51292900 0.69127058 0.95758261
C -0.36219539 1.71705952 2.49744465
P -0.35286334 -4.19603500 -0.75950784
C 0.51880849 -4.45286291 -2.32732974
C 0.14516967 -5.59865850 0.27416011
H 2.40677104 -0.11805717 0.44909903
C -2.09375247 -4.51419561 -1.15010897
H -2.45561925 -3.75283436 -1.84380399
H -2.20613107 -5.50199674 -1.60328360
H -2.68677385 -4.46481986 -0.23481984
H 0.20615510 -3.69252607 -3.04568124
H 0.29510493 -5.44480772 -2.72696288
H 1.59442581 -4.36048111 -2.16495520
H 1.21340502 -5.53090955 0.48894375
H -0.06371856 -6.54034574 -0.23908949
H -0.40450666 -5.56876361 1.21692653
H 2.72198387 2.13426585 1.11271596
H -1.23952817 2.32741880 2.69215506
H 0.09236304 1.40282831 3.43740019
C 0.35270139 3.42503374 0.75411070
C -0.98875483 3.76799929 0.49638681
C 1.38896428 4.11840508 0.10122852
C -1.26790062 4.75934919 -0.40909367
H -1.81701187 3.25738008 0.96845055
C 1.07571027 5.12232890 -0.78026349
H 2.43300217 3.90937415 0.28838775
C -0.24713474 5.44997048 -1.05288070
F -2.53112430 5.09514416 -0.69358489
F -0.53266553 6.41565388 -1.91088997
F 2.03647493 5.81426586 -1.40290800
*

alt-constrF19
Au 0.07258977 -2.71823588 0.17311926
C 1.80739643 -0.39321034 0.90281426
C 0.61382762 -0.94416096 1.09225005
C 1.06296103 1.59727482 1.90794640
C 2.30035381 0.88232687 1.68178934
H 2.75108202 0.55911420 2.62046567
C -0.41374398 -0.28546545 1.94676237
H -0.87069040 -0.97130837 2.66218180
H -1.21148985 0.11688372 1.31735734
C 0.15901021 0.93966872 2.82151682
P -0.55582272 -4.68924053 -0.84502520
C -0.49385619 -4.63622991 -2.65467875
C 0.46460833 -6.11541992 -0.39087059
H 2.54642632 -0.75313323 0.19506070
C -2.25197047 -5.19514679 -0.45818908
H -2.94598770 -4.41731795 -0.78242200
H -2.49237658 -6.13206078 -0.96626680
H -2.35451494 -5.32978728 0.62029035
H -1.15450645 -3.84678587 -3.01810106
H -0.80942528 -5.59551361 -3.07175729
H 0.52561284 -4.41728188 -2.97791353
H 1.50247003 -5.92366268 -0.66988398
H 0.10958502 -7.01245424 -0.90378064
H 0.41391119 -6.26960893 0.68866436
H 3.03307038 1.41389873 1.08151019
H -0.69394547 1.52369756 3.15447932
H 0.69960321 0.50072247 3.65821697
C 0.64649800 2.72127969 1.07451363
C -0.40820586 3.54247168 1.47659408
C 1.29789988 2.98480240 -0.13442555
C -0.79934359 4.62465638 0.69465770
H -0.92352295 3.37819768 2.41257750
C 0.88890798 4.03807147 -0.94073041
H 2.11288364 2.36335686 -0.48004383
C -0.15141281 4.85639949 -0.51122402
N -0.56813691 5.99494844 -1.35161264
O -0.73402280 5.77079922 -2.53015333
O -0.71037455 7.06106739 -0.79518045
C -1.89235927 5.44424252 1.15115649
N -2.77778468 6.05546377 1.55018707

C 1.58927502 4.27302048 -2.17763489
N 2.18949373 4.42599303 -3.14363565
*

alt-constrF20
Au -0.06391436 -3.37093466 -0.26534515
C -2.06175963 -1.14966259 -0.30279305
C -0.99543525 -1.64480475 -0.92402387
C -1.82434667 0.87644269 -1.46964162
C -2.86127091 0.09841668 -0.81983019
H -3.61056159 -0.23813627 -1.53665333
C -0.39628338 -0.94331413 -2.09782872
H -0.21318029 -1.61671853 -2.93712201
H 0.56413744 -0.50616196 -1.81117103
C -1.30586287 0.24543351 -2.66746998
P 0.98716898 -5.28588336 0.47405913
C 1.32289599 -5.29773468 2.25453954
C 0.05017673 -6.80731682 0.17480389
H -2.46710340 -1.54572203 0.62220752
C 2.59950625 -5.58129976 -0.29776144
H 3.26213447 -4.73913213 -0.08922667
H 3.04377274 -6.49933711 0.09407360
H 2.47462275 -5.67041492 -1.37864063
H 1.96194112 -4.45020505 2.50976317
H 1.82084304 -6.22763802 2.53937360
H 0.38311980 -5.20617948 2.80245735
H -0.91323568 -6.74707608 0.68472059
H 0.60513291 -7.67207599 0.54634383
H -0.12618285 -6.92136242 -0.89648617
H -3.34920395 0.58351963 0.01984452
H -0.68659535 0.86795323 -3.30580541
H -2.11285043 -0.21479320 -3.23524903
C -1.16970309 2.00971772 -0.83504503
C -1.51088957 2.39245120 0.46752755
C -0.16885611 2.71799061 -1.50804405
C -0.86540507 3.44363061 1.09021950
H -2.27329829 1.86030162 1.01705851
C 0.48227586 3.77834360 -0.90209688
H 0.10487535 2.45802923 -2.51978765
C 0.12636917 4.12640471 0.39431499
C -1.27188996 3.81793671 2.50488009
C 1.56683198 4.50911823 -1.67391633
N 0.81997959 5.25883989 1.05374891
F -1.87540836 2.79217356 3.12107902
F -2.12926701 4.85116624 2.51652151
F -0.21006083 4.16488882 3.25353375
F 1.42148745 4.33416835 -2.99477467
F 2.79074875 4.06565142 -1.34442234
F 1.53976833 5.83314266 -1.44183279
O 0.16684642 6.25946567 1.24144191
O 1.98036478 5.08592635 1.34866358
*

alt-constrF21
Au -0.02693828 -2.52529811 0.18017804
C 1.37014793 -0.22311271 1.46894233
C 0.17439804 -0.74377981 1.21336062
C 0.36722604 1.80687488 2.10267826
C 1.58889361 1.06440302 2.34323091
H 1.66869063 0.76303882 3.38826755
C -1.07419660 -0.03985395 1.62869618
H -1.78117851 -0.70240960 2.13115496
H -1.57552603 0.36941105 0.74773463
C -0.81819426 1.17779348 2.64156109
P -0.25278519 -4.50973566 -0.97267685
C -1.74628177 -4.59763829 -1.99460348
C 1.10774857 -4.85382421 -2.11876637
H 2.30383058 -0.62636118 1.09098402
C -0.33607137 -5.97493165 0.08970194
H -1.19016893 -5.88791121 0.76402176
H -0.44237289 -6.87667866 -0.51800344
H 0.57595315 -6.04355897 0.68574923
H -2.62779734 -4.48235829 -1.36103142
H -1.79407518 -5.55792743 -2.51353634
H -1.73258316 -3.78868781 -2.72759875
H 1.17474260 -4.05106325 -2.85568369
H 0.93995735 -5.80450261 -2.63047680
H 2.04695731 -4.90021809 -1.56418629
H 2.49869087 1.56875150 2.03080920
H -1.71438591 1.79102422 2.65102608
H -0.61832910 0.74073255 3.61857426
C 0.29575955 2.92311121 1.17062592
C -0.82732909 3.75834515 1.17488104
C 1.34278507 3.14707672 0.26594589
C -0.88050917 4.81350326 0.29025517
H -1.64569966 3.62802894 1.86909438
C 1.23846876 4.18239910 -0.63214340
H 2.21925070 2.51495444 0.22578364
C 0.13849321 5.02977143 -0.62309454
F -1.93902018 5.62481281 0.28789922
F 2.21729926 4.40303640 -1.51089286
N 0.05776393 6.14688861 -1.57302521
O 0.10438778 5.86799780 -2.75117530
O -0.04937932 7.25873509 -1.10373360
*

alt-constrF24
Au -0.33260644 -2.96409610 0.03505977
C 0.08930227 -0.68186902 1.91514275
C -0.72190522 -1.16460998 0.97669661
C -0.95559112 1.42108548 1.80406851
C -0.19103826 0.61808985 2.74440111
H -0.78132962 0.35544445 3.62340896
C -1.93543018 -0.40199592 0.54780894
H -2.82878986 -1.02932985 0.53397512
H -1.78588105 -0.02970831 -0.47000851
C -2.25274582 0.84490315 1.47312383
P 0.09156647 -4.97383639 -1.01463146
C 1.74851150 -5.09563742 -1.73815776
C -0.03637481 -6.41684078 0.07415235
H 1.03351337 -1.14329970 2.18505241
C -1.03379940 -5.33556743 -2.38799015
H -0.95416781 -4.54816635 -3.13998225
H -0.78241308 -6.29770731 -2.84059534
H -2.06065666 -5.36513443 -2.01847558
H 1.88227011 -4.30295872 -2.47682980
H 1.88082549 -6.06750986 -2.21956092
H 2.49761402 -4.97378020 -0.95339660
H 0.67998689 -6.31831629 0.89205641
H 0.17251484 -7.33252552 -0.48411148
H -1.04281098 -6.46844246 0.49390442
H 0.76067620 1.04752141 3.04343239
H -2.93280336 1.52015017 0.96117864
H -2.71534471 0.45830007 2.38146945
C -0.39191564 2.53855285 1.08813013
C -0.98175154 2.98011552 -0.10401008
C 0.76212890 3.18857376 1.56305133
C -0.41197312 4.02153764 -0.81635245
H -1.86901984 2.49637283 -0.49149204
C 1.29863238 4.24042317 0.85308044
H 1.21681523 2.88631988 2.49540107
C 0.72337873 4.66017946 -0.34448633
C -1.06428500 4.45130006 -2.10784822
C 2.52148946 4.97049672 1.35166132
H 1.16002925 5.48332860 -0.89773762
F -0.38068439 5.42504639 -2.72829558
F -2.31518592 4.91101697 -1.89969313
F -1.17022781 3.42220093 -2.97287057
F 3.00417015 4.44451419 2.48877266
F 2.25490211 6.26994696 1.59455587
F 3.51732129 4.94269353 0.44335938
*

alt-constrF4

Au	-0.03756950	-2.50816702	0.15404252
C	1.27891036	-0.22609818	1.56112322
C	0.09932460	-0.75782201	1.24589576
C	0.25335138	1.80099520	2.16849171
C	1.46947502	1.04930890	2.44823480
H	1.52098526	0.74848066	3.49572102
C	-1.17413049	-0.07834117	1.64514175
H	-1.87134853	-0.76933811	2.12322095
H	-1.67000918	0.31375498	0.75223000
C	-0.95235829	1.12891814	2.64359059
P	-0.19609044	-4.45542401	-1.07308689
C	1.39614312	-5.09425179	-1.65677390
C	-0.94093892	-5.84014958	-0.17210245
H	2.22436750	-0.62868036	1.21197890
C	-1.20106904	-4.29571301	-2.57271346
H	-0.76698697	-3.53254577	-3.22155449
H	-1.24032799	-5.24791909	-3.10706789
H	-2.21312851	-3.98998049	-2.30047284
H	1.87963940	-4.34478171	-2.28637701
H	1.24927386	-6.01190537	-2.23128099
H	2.04065721	-5.30085527	-0.80017685
H	-0.34174706	-6.06264023	0.71293606
H	-0.98974048	-6.72557734	-0.81041257
H	-1.94844660	-5.56593520	0.14666356
H	2.39085511	1.54264925	2.15497841
H	-1.84119012	1.75280036	2.66803481
H	-0.76281394	0.70138919	3.62845585
C	0.21589118	2.95340955	1.30596894
C	-1.00935191	3.39505696	0.76868988
C	1.39397272	3.64417625	0.97541600
C	-1.04728013	4.47511636	-0.08727631
H	-1.93086243	2.88106237	1.00467548
C	1.35308024	4.74065308	0.13287844
H	2.34548161	3.34316344	1.39081257
C	0.13518119	5.14381793	-0.39638682
H	-1.98894986	4.80362866	-0.51101409
H	2.26330746	5.27389299	-0.10751296
C	0.05632877	6.32930977	-1.33192443
F	-0.40190344	5.96068959	-2.54550010
F	-0.79005676	7.26751741	-0.86292711
F	1.24503060	6.92277565	-1.51944107

*

alt-constrF9

Au	0.03339574	-1.89789668	0.15156737
C	1.31423221	0.54207739	1.30727619
C	0.14551649	-0.04605445	1.06470669
C	0.22732893	2.57517986	1.77149871
C	1.47383817	1.89139642	2.08596959
H	1.54097116	1.67521068	3.15322733
C	-1.13824757	0.63533298	1.41916786
H	-1.82073748	-0.02534506	1.95723402
H	-1.64678129	0.95040660	0.50370247
C	-0.94581194	1.92505518	2.33224901
P	-0.09638749	-3.96858711	-0.85655732
C	0.31261688	-3.95800452	-2.62180705
C	1.01364472	-5.20868085	-0.13948519
H	2.26664801	0.13826184	0.97863896
C	-1.74081546	-4.72526268	-0.77330762
H	-2.46611356	-4.07350020	-1.26421616
H	-1.73492156	-5.69974905	-1.26738132
H	-2.03159210	-4.84908142	0.27166393
H	-0.37253389	-3.29211008	-3.14994432
H	0.23179414	-4.96602222	-3.03546537
H	1.33183945	-3.59056791	-2.75628867
H	2.04647297	-4.86594659	-0.22710334
H	0.90173985	-6.16310706	-0.65940638
H	0.77665844	-5.34075515	0.91798382
H	2.37106831	2.40649241	1.75693584
H	-1.86187882	2.50665105	2.29113259
H	-0.74259120	1.57717603	3.34439745
C	0.13436033	3.64054418	0.79992748
C	-1.03615856	4.41218722	0.70773776
C	1.20755610	3.91031392	-0.06878070
C	-1.13257779	5.43128231	-0.22047677
H	-1.86303743	4.24240604	1.38318259
C	1.10667230	4.90751696	-1.01597314
H	2.11171513	3.31851266	-0.02711166
C	-0.06262800	5.66770207	-1.08133623
H	-2.02641191	6.03897119	-0.28276665
H	1.92308849	5.10587278	-1.69872113
C	-0.16305781	6.72439984	-2.05821922
N	-0.24221097	7.56536344	-2.83707041

*

constrA0

C	-0.36156559	-3.97407305	1.14602058
C	-0.46680322	-4.05207196	-0.23727173
C	-0.10757835	-2.96636781	-1.02253170
C	0.37242333	-1.80481030	-0.42452255
C	0.46245461	-1.72075377	0.96201682
C	0.10343337	-2.80951444	1.74535543
H	-0.64041495	-4.82313877	1.75906802
H	-0.82335576	-4.96163854	-0.70622026
H	-0.18027370	-3.03226803	-2.10318891
H	0.82781265	-0.81332384	1.43374229
H	0.18668772	-2.74881008	2.82415856
C	0.77580322	-0.61264448	-1.28764903
H	1.77306910	-0.26087998	-1.02816055
H	0.78980820	-0.93254965	-2.33475968
H	-1.23039429	0.16279756	-1.54489709
C	-0.14521698	1.66814205	-0.53816282
C	-1.30685163	2.49092876	-0.48967935
C	1.06996303	2.14227134	0.03392570
C	-1.25473510	3.73102531	0.09967520
H	-2.22994364	2.12305113	-0.92463143
C	1.10908412	3.38059197	0.62216046
H	1.95985936	1.52690051	0.00571827
C	-0.04907593	4.16881598	0.65197384
H	-2.13324579	4.36261281	0.13906547
H	2.02640918	3.75289749	1.06084037
H	-0.00677880	5.14769511	1.11840881
C	-0.24979556	0.42906624	-1.15180180

*

constrA1

C	-0.71742178	-3.62277912	0.11442588
C	0.49849546	-3.57114921	-0.56783652
C	1.26617309	-2.41840209	-0.57281096
C	0.83693819	-1.29089305	0.12184689
C	-0.38444984	-1.32070715	0.78737704
C	-1.14634291	-2.48015007	0.78864647
H	0.85067653	-4.44892701	-1.09916451
H	2.21325300	-2.40412902	-1.10258451
H	-0.74090559	-0.44395341	1.32029177
H	-2.09146916	-2.49762434	1.32062750
C	1.68278258	-0.02193858	0.12588545
H	1.83731930	0.34315767	1.14015653
H	2.66220838	-0.25862773	-0.30215752
H	1.02147697	0.71206748	-1.80159550
C	0.39703722	2.14547091	-0.38152508
C	-0.20567988	2.92271829	-1.41069986
C	0.32591280	2.59790661	0.96644378
C	-0.84631543	4.09995768	-1.10666814
H	-0.15064544	2.57142598	-2.43547340
C	-0.31927131	3.77283892	1.25849473
H	0.77816648	2.01589432	1.75897744
C	-0.90029959	4.51827223	0.22442483
H	-1.30552118	4.69691297	-1.88451359
H	-0.38070843	4.12759309	2.27973889

H	-1.40686221	5.44698139	0.46604583
C	1.03911416	0.96927940	-0.74309872
C	-1.52991546	-4.88923646	0.14165426
H	-2.58303610	-4.68056465	0.33421230
H	-1.44914579	-5.42879359	-0.80335237
H	-1.16929112	-5.55143537	0.93376529

*

constrA10

C	-1.24894163	-3.47646796	-0.64138217
C	0.07084397	-3.66046615	-0.22398595
C	0.73343335	-2.58288720	0.35114435
C	0.09340999	-1.35764491	0.52373213
C	-1.21340426	-1.18235479	0.08609056
C	-1.88526602	-2.25403722	-0.49091366
H	1.75621413	-2.70925353	0.69341756
H	-1.71546559	-0.22743970	0.20785650
H	-2.90984123	-2.13280115	-0.82303605
C	0.83734057	-0.20276246	1.18895788
H	0.26951176	0.19604017	2.02810642
H	1.79028324	-0.58152347	1.57228722
H	1.83385154	0.49287583	-0.60374490
C	0.59118803	2.07847193	0.03043093
C	1.00210196	2.86494299	-1.08302741
C	-0.34944886	2.61557811	0.95451138
C	0.49885633	4.13109155	-1.26179455
H	1.71665285	2.44922961	-1.78531992
C	-0.84740018	3.87951924	0.76406641
H	-0.67271719	2.02771881	1.80381636
C	-0.42226381	4.63120777	-0.33903313
H	0.80785300	4.73534355	-2.10540124
H	-1.56358940	4.29929422	1.45935152
H	-0.82114922	5.63051603	-0.47969831
C	1.13386283	0.80901095	0.16889349
H	-1.78398026	-4.30648382	-1.09110776
C	0.74462018	-4.99735475	-0.38444488
H	1.78260335	-4.96351085	-0.05162315
H	0.72949548	-5.31383682	-1.42987142
H	0.22459796	-5.76128134	0.19838102

*

constrA11

C	-1.26070877	-3.57667290	-0.39806909
C	0.10356229	-3.70312566	-0.14123409
C	0.82295130	-2.61331724	0.34140908
C	0.17128692	-1.41156628	0.57716382
C	-1.18704184	-1.27039462	0.30555063
C	-1.89605125	-2.36491185	-0.17350027
H	1.87998833	-2.73721185	0.54794435
H	-1.69334505	-0.32756501	0.48342147
H	-2.95688561	-2.27454570	-0.37596311
C	0.95876819	-0.22093366	1.11346279
H	0.48950778	0.18859840	2.00643539
H	1.96432322	-0.56147392	1.38319599
H	1.67256449	0.42664407	-0.82508780
C	0.55885907	2.04158675	-0.04270200
C	0.81167563	2.80311872	-1.21932816
C	-0.22635812	2.60989909	1.00072936
C	0.30729563	4.07510091	-1.34442942
H	1.40776921	2.36363113	-2.01187929
C	-0.72699524	3.87947264	0.86323600
H	-0.42952068	2.04115790	1.89880973
C	-0.45844331	4.60602358	-0.30423615
H	0.49675988	4.66061096	-2.23523065
H	-1.32559412	4.32298525	1.64906463
H	-0.85825889	5.61006782	-0.40218411
C	1.09994206	0.76718443	0.03696120
H	-1.82349924	-4.42633176	-0.77150310
O	0.78889441	-4.85426880	-0.33586244
H	0.19664490	-5.54808413	-0.64613461

*

constrA12

C	-0.32909397	-2.94957644	0.17821064
C	0.92372591	-2.79990601	-0.42385189
C	1.58774418	-1.58675451	-0.39594416
C	1.02300389	-0.49092807	0.25264358
C	-0.23282066	-0.61935175	0.83763636
C	-0.89271592	-1.83828948	0.80763635
H	1.39372591	-3.64327330	-0.91669674
H	2.56401595	-1.49883251	-0.86190648
H	-0.69553763	0.22765744	1.33594540
H	-1.86373595	-1.93205712	1.28139662
C	1.75700819	0.84378229	0.29305032
H	1.82016390	1.22772204	1.31022400
H	2.77718407	0.68820113	-0.07280813
H	1.15308066	1.50588236	-1.67832523
C	0.32891971	2.89316348	-0.31451833
C	-0.27202127	3.61145731	-1.38614934
C	0.14286495	3.34860191	1.02099790
C	-1.02023908	4.73656145	-1.13439660
H	-0.12943542	3.25772160	-2.40158747
C	-0.60880341	4.47114133	1.26065831
H	0.59178167	2.81032845	1.84566726
C	-1.18495243	5.16009592	0.18579373
H	-1.47862632	5.28883515	-1.94504878
H	-0.75759031	4.82770973	2.27223576
H	-1.77620170	6.04766829	0.38598782
C	1.08672329	1.77136296	-0.62385235
C	-1.06167856	-4.23875919	0.16817466
C	-0.96438205	-5.16778856	-1.01935512
C	-0.31071553	-5.54607905	0.26807063
H	-2.02265439	-4.20334213	0.66745372
H	-0.33473634	-4.85371941	-1.84307090
H	-1.86528196	-5.69104413	-1.31308086
H	-0.75999318	-6.33041613	0.86350675
H	0.76929510	-5.49140401	0.33237671

*

constrA13

C	-1.25132932	-3.23789513	-0.40174380
C	0.05893987	-3.35656402	0.05495130
C	0.70718708	-2.26000251	0.60568417
C	0.04589968	-1.04060656	0.71442816
C	-1.25888538	-0.92137013	0.24540495
C	-1.90887296	-2.02142004	-0.30472595
H	1.72614348	-2.36731757	0.96782655
H	-1.78095955	0.02806613	0.32064121
H	-2.92928447	-1.92412331	-0.65600954
C	0.75657548	0.16315917	1.32591352
H	0.15489285	0.61459369	2.11308495
H	1.69732821	-0.17718448	1.77106852
H	1.80136506	0.73330129	-0.48630482
C	0.57008740	2.37048996	0.01871542
C	1.01952939	3.07935589	-1.13360162
C	-0.38093903	2.98190611	0.88696691
C	0.54366209	4.33949227	-1.40242108
H	1.74160627	2.60817540	-1.79182571
C	-0.85026377	4.23909883	0.60666190
H	-0.73213079	2.45550315	1.76506287
C	-0.38737344	4.91197683	-0.53262835
H	0.88132868	4.88451335	-2.27487501
H	-1.57255379	4.71528396	1.25787237
H	-0.76455189	5.90727361	-0.74427253
C	1.08798772	1.10668511	0.24766786
H	-1.73611957	-4.10944391	-0.82618845
C	0.77030343	-4.66757477	-0.04273136
O	0.27848652	-5.65080579	-0.52806560
H	1.80052240	-4.67946222	0.36629683

*

constrA14

C	-0.10820510	-3.08341301	0.07255423
C	1.20709428	-2.91146826	-0.31797792
C	1.78733842	-1.66371942	-0.14802952
C	1.05533005	-0.62110288	0.41338453
C	-0.27106056	-0.81836179	0.78759678
C	-0.86210834	-2.06239246	0.62409153
H	1.75628752	-3.74280023	-0.73904302
H	2.82135636	-1.51198664	-0.43768197
H	-0.84649470	-0.00771650	1.22292837
H	-1.88682131	-2.25001855	0.91491696
C	1.70206154	0.74753053	0.60282950
H	1.54519804	1.11481197	1.61547326
H	2.78054436	0.65175273	0.43807955
H	1.41363798	1.38168783	-1.45171937
C	0.38145144	2.78083032	-0.25894124
C	-0.04191351	3.48061516	-1.42807899
C	-0.02222957	3.25733876	1.02377277
C	-0.82794588	4.60122929	-1.31942294
H	0.26659936	3.11294909	-2.40088521
C	-0.80828668	4.37574986	1.11871146
H	0.29105173	2.73770562	1.92005562
C	-1.20683329	5.04216461	-0.04902304
H	-1.15287054	5.13787605	-2.20182907
H	-1.12194356	4.74844430	2.08578229
H	-1.82876678	5.92725961	0.03819040
C	1.17442452	1.66022241	-0.42588538
N	-0.73869116	-4.41543782	-0.10804940
O	-0.05167881	-5.28862064	-0.59113820
O	-1.89454505	-4.52898721	0.23899197

*

constrA15

C	-0.52355645	-3.26353242	-0.11706529
C	0.86167578	-3.19155300	-0.04709208
C	1.49991996	-2.01880570	0.31373951
C	0.73780684	-0.89782326	0.62281728
C	-0.65044551	-0.94295443	0.54376470
C	-1.26595978	-2.12803710	0.18237843
H	2.58195536	-2.01289572	0.36835562
H	-1.27254297	-0.08545183	0.77339685
C	1.42878924	0.39812646	1.02812935
H	1.02828680	0.77758419	1.96667644
H	2.49518686	0.19657216	1.17257300
H	1.80394897	1.06817632	-1.00148415
C	0.59208600	2.54754297	-0.11165217
C	0.58702984	3.29204359	-1.32872221
C	-0.12669867	3.04732199	1.01473333
C	-0.10038554	4.47759784	-1.41388389
H	1.13376084	2.90642155	-2.18259218
C	-0.81109217	4.23046382	0.91606780
H	-0.13331710	2.49390383	1.94491943
C	-0.79535072	4.93975610	-0.29343656
H	-0.10832660	5.04855609	-2.33375290
H	-1.36153242	4.62090755	1.76287206
H	-1.34039768	5.87583225	-0.36010721
C	1.30274914	1.36145605	-0.07968327
F	-2.59371980	-2.19563370	0.11284416
F	-1.12593389	-4.39146233	-0.46086293
F	1.57190414	-4.27868129	-0.33182290

*

constrA16

C	-0.09917660	-3.50952441	-0.29591452
C	1.26644308	-3.27953744	-0.35103460
C	1.78556931	-2.06150111	0.06447982
C	0.94408090	-1.07011672	0.55108716
C	-0.43731444	-1.28473034	0.58385055
C	-0.95331361	-2.49985712	0.17831829
H	1.91169791	-4.07222178	-0.70960241
H	2.85769670	-1.90076109	0.03298870
H	-1.10977512	-0.51233381	0.94826022
C	1.51436303	0.26335142	1.01408884
H	1.06249184	0.58297477	1.95194700
H	2.58862794	0.13789848	1.18487101
H	1.90586088	1.02899397	-0.97181368
C	0.51040858	2.35516330	-0.10110235
C	0.47567173	3.12480805	-1.29757377
C	-0.28272828	2.75786573	1.00981073
C	-0.31342705	4.24766891	-1.37859366
H	1.08098688	2.81208832	-2.14161629
C	-1.06751533	3.87939248	0.91728718
H	-0.26417458	2.18313192	1.92677608
C	-1.07992169	4.61896450	-0.27263703
H	-0.34300855	4.83843743	-2.28547447
H	-1.67306055	4.19809336	1.75657037
H	-1.70256250	5.50546259	-0.33480715
C	1.34205905	1.24314668	-0.06457165
O	-0.59244148	-4.69787644	-0.69398945
H	-1.55101122	-4.70143143	-0.56948385
O	-2.27985698	-2.82524250	0.18858317
H	-2.80055254	-2.14564930	0.62694195

*

constrA17

C	0.01809837	-2.74511685	0.84955518
C	0.04495362	-2.78322088	-0.57342757
C	0.30878063	-1.62505100	-1.27871432
C	0.57185205	-0.42589007	-0.60076600
C	0.51933266	-0.38416532	0.77971627
C	0.25564753	-1.54809838	1.50476587
H	0.34576459	-1.64605615	-2.35989893
H	0.70431804	0.54196055	1.31396339
H	0.23805664	-1.50644649	2.58491360
C	0.87335188	0.83684810	-1.39041548
H	1.84775063	1.24801019	-1.12917226
H	0.89688689	0.58392805	-2.45561768
H	-1.18380011	1.48329869	-1.58532116
C	-0.19257948	2.98884844	-0.47672472
C	-1.39904774	3.73458143	-0.37479664
C	0.99182730	3.49486712	0.12572573
C	-1.41810929	4.93694060	0.29164867
H	-2.30060729	3.34143834	-0.83217360
C	0.96018958	4.69443617	0.79293524
H	1.91521842	2.93378121	0.06325476
C	-0.24001097	5.41064067	0.87202998
H	-2.33289813	5.51088638	0.36982264
H	1.85583096	5.08898872	1.25639639
H	-0.25443082	6.35835758	1.40010889
C	-0.22011980	1.78874964	-1.18018800
O	-0.24129402	-3.91694733	1.44784297
C	-0.26405963	-3.94759700	2.87194103
H	0.70625457	-3.65493791	3.28341747
H	-1.04984841	-3.29525063	3.26386569
H	-0.47864300	-4.97924186	3.14063492
O	-0.18814967	-3.98804426	-1.12064188
C	-0.16616037	-4.08846550	-2.53950655
H	-0.93755269	-3.45521129	-2.98872954
H	0.81763914	-3.81846193	-2.93543107
H	-0.37521078	-5.13144047	-2.76564633

*

constrA18

C	-0.09310080	-2.93847193	0.05524684
C	1.24458398	-2.72278931	-0.35265028
C	1.83818159	-1.48909417	-0.20408535
C	1.15223235	-0.41962944	0.37472444
C	-0.17947261	-0.61110223	0.74528805
C	-0.79150133	-1.83889909	0.60705497
H	1.82332446	-3.53125938	-0.77597519
H	2.86990413	-1.36272836	-0.51738196
H	-0.74905445	0.20989133	1.17149566

H	-1.81708528	-1.94980157	0.92872536
C	1.82051776	0.93206106	0.54616655
H	1.69502630	1.31128602	1.56015218
H	2.89397849	0.81246854	0.37017543
H	1.58381115	1.62884475	-1.49090263
C	0.38428741	2.89235473	-0.28830979
C	-0.04732040	3.60754755	-1.43740165
C	-0.09790496	3.27684496	0.99162932
C	-0.91760930	4.66531753	-1.31186716
H	0.32107892	3.30808883	-2.41266073
C	-0.97021156	4.33161693	1.10539733
H	0.21973135	2.73871113	1.87531880
C	-1.37535743	5.02186048	-0.04267312
H	-1.24719689	5.21541050	-2.18432420
H	-1.34369993	4.63266638	2.07626491
H	-2.06307674	5.85497035	0.05759401
C	1.28733241	1.84539818	-0.46557902
N	-0.68309815	-4.15515232	-0.07805903
C	-2.05480857	-4.35384373	0.35866574
C	0.05066085	-5.26492741	-0.66454472
H	-2.33256302	-5.39214004	0.19369154
H	-2.16818160	-4.13820235	1.42575514
H	-2.75108461	-3.72210006	-0.20265683
H	-0.59307809	-6.14092271	-0.68935959
H	0.36065057	-5.04234590	-1.69042471
H	0.93920054	-5.51320276	-0.07544851

*

constrA19

C	-0.24016560	-2.52017951	0.03251942
C	1.14606392	-2.42864430	0.04903939
C	1.73923036	-1.20275666	0.34537046
C	0.95873957	-0.09555671	0.65009256
C	-0.42498184	-0.20411343	0.63458689
C	-1.03573426	-1.41570176	0.31686384
H	2.82115203	-1.13918832	0.34707328
H	-1.05647278	0.64400776	0.87459106
C	1.61740294	1.23650053	0.98937937
H	1.24525089	1.62001128	1.93776683
H	2.69726790	1.08415171	1.08572857
H	1.85122485	1.86935518	-1.07916060
C	0.65403275	3.33230419	-0.15214457
C	0.55166627	4.04376023	-1.38864536
C	-0.01397686	3.84358107	1.00419351
C	-0.18042503	5.20143724	-1.46380780
H	1.05991020	3.65209827	-2.26337374
C	-0.74232926	4.99892548	0.91446221
H	0.05495873	3.32020456	1.94924636
C	-0.82331206	5.67185428	-0.31493778
H	-0.26392337	5.74537024	-2.39621080
H	-1.25447895	5.39718091	1.78140732
H	-1.40434733	6.58674215	-0.37397088
C	1.39509940	2.17052969	-0.13666396
N	-0.88209330	-3.80954026	-0.29804099
O	-0.50211361	-4.77019657	0.32838202
O	-1.72527643	-3.77507799	-1.16451595
C	-2.47478295	-1.47350270	0.32295853
N	-3.62111321	-1.43155391	0.37026866
C	1.99780889	-3.54592220	-0.26690927
N	2.73900823	-4.37971901	-0.53756300

*

constrA2

C	-0.11760374	-3.40335566	0.17276111
C	1.13238657	-3.23172554	-0.40700418
C	1.73801240	-1.98682209	-0.35405099
C	1.10759391	-0.92275541	0.28503243
C	-0.15254176	-1.10578185	0.84783688
C	-0.76610810	-2.34821942	0.80169439
H	1.62395817	-4.06828471	-0.88712149
H	2.71931609	-1.85380134	-0.79723011

H	-0.65876178	-0.28223339	1.34216775
H	-1.73951898	-2.50451284	1.24887277
C	1.78204937	0.44278267	0.34683646
H	1.79119523	0.83057890	1.36422509
H	2.82062857	0.33325681	0.01734926
H	1.19936678	1.05728627	-1.64801681
C	0.32300378	2.45121963	-0.32750837
C	-0.27152480	3.13663067	-1.42624898
C	0.10124691	2.93044475	0.99596893
C	-1.04724355	4.25004110	-1.21197413
H	-0.10136035	2.76568211	-2.43127556
C	-0.67689056	4.04135215	1.19741043
H	0.54533179	2.41928613	1.84033120
C	-1.24589147	4.69602415	0.09654972
H	-1.50108625	4.77674267	-2.04194600
H	-0.85292329	4.41618485	2.19793543
H	-1.85893639	5.57503215	0.26722479
C	1.10547060	1.34039149	-0.60014790
Cl	-0.87832682	-4.95281699	0.11241648

*

constrA20

C	-0.32542333	-1.93125436	0.03556254
C	1.06169891	-1.94626662	0.10722431
C	1.72644137	-0.77928519	0.45479786
C	1.01945143	0.37982920	0.74337935
C	-0.36556192	0.37691210	0.66340379
C	-1.05019947	-0.77688999	0.30999201
H	2.80816519	-0.79069721	0.50840307
H	-0.93365485	1.27129637	0.89009198
C	1.76067718	1.65357950	1.13349157
H	1.34088023	2.08093785	2.04238765
H	2.80943646	1.40675532	1.32708822
H	2.25015888	2.24584059	-0.89998409
C	1.06268286	3.79569504	-0.10929148
C	1.13235128	4.49527125	-1.35320739
C	0.31623606	4.36257011	0.96900318
C	0.48824936	5.69682234	-1.51016644
H	1.69968379	4.06042336	-2.16919942
C	-0.32323660	5.56132997	0.79828963
H	0.25544038	3.84754661	1.91913323
C	-0.23561503	6.22242759	-0.43632075
H	0.53523916	6.23258855	-2.44981454
H	-0.89345154	6.00217237	1.60638226
H	-0.74676903	7.17187401	-0.55981251
C	1.72190602	2.58748271	-0.01041191
C	1.88620650	-3.19855671	-0.16864837
F	3.15049750	-2.85989899	-0.47923712
F	1.39134722	-3.89579501	-1.19593098
F	1.93184095	-3.99006370	0.90497272
C	-2.56989265	-0.71632165	0.22153052
F	-3.03514809	0.31015063	0.95996363
F	-3.14075085	-1.83242698	0.67767627
F	-2.96957275	-0.51280639	-1.03843963
N	-1.04985133	-3.16761926	-0.34808846
O	-1.58457177	-3.15162114	-1.43155564
O	-1.03474143	-4.06590080	0.45802290

*

constrA21

C	-0.15372640	-2.76327823	0.02663951
C	1.21609022	-2.58474449	-0.07481750
C	1.79510456	-1.35192086	0.17138574
C	0.98260223	-0.28933108	0.54777814
C	-0.39548906	-0.44574012	0.65173558
C	-0.94844565	-1.68685539	0.38804032
H	2.87023984	-1.25428349	0.08328109
H	-1.05148846	0.36407008	0.94881490
C	1.60730233	1.07072892	0.83427566
H	1.31226509	1.43420878	1.81704873
H	2.69758327	0.96812288	0.82317215

H 1.59534142 1.70217708 -1.24338821
C 0.47233376 3.14145022 -0.19022637
C 0.22408696 3.85030508 -1.40502512
C -0.07399202 3.63858915 1.03191865
C -0.52975466 4.99726877 -1.39696855
H 0.63975949 3.46716349 -2.33085293
C -0.82563729 4.78361555 1.02582207
H 0.10435089 3.11312148 1.96126361
C -1.05005318 5.45717654 -0.18418215
H -0.72268214 5.54040582 -2.31347146
H -1.24623675 5.17202271 1.94491617
H -1.64731042 6.36335357 -0.17680659
C 1.23847256 1.99474231 -0.25642269
N -0.75967485 -4.07732190 -0.24952788
O -0.44892232 -4.97744342 0.49439366
O -1.51068171 -4.12876093 -1.19641245
F -2.26078749 -1.85541326 0.50025176
F 1.97974328 -3.60836439 -0.43211513
*

constrA22
C 0.28957400 -2.24359589 0.08186114
C 1.52069104 -1.93048371 -0.54298805
C 2.02067777 -0.64888789 -0.50878931
C 1.34695728 0.37405725 0.16422801
C 0.11232721 0.08267542 0.74937961
C -0.40572289 -1.19396306 0.72767674
H 2.09102145 -2.70744681 -1.03351751
H 2.97472764 -0.44389593 -0.98466995
H -0.45010043 0.86611902 1.24940251
H -1.35781887 -1.38788976 1.20250052
C 1.91475758 1.77970058 0.20845804
H 1.92563362 2.17316248 1.22489114
H 2.95129656 1.74497435 -0.14078566
H 1.29022105 2.40571538 -1.76850345
C 0.19357514 3.58883592 -0.39604553
C -0.47996596 4.23849922 -1.46429288
C -0.10508066 3.96051239 0.94186098
C -1.40613512 5.22269332 -1.20681728
H -0.25123627 3.94860519 -2.48417967
C -1.03500144 4.94128774 1.18827914
H 0.39726117 3.47117889 1.76621711
C -1.67995347 5.56908847 0.11692309
H -1.91964211 5.72302365 -2.01824836
H -1.26943863 5.23193856 2.20480332
H -2.41117691 6.34397244 0.32157632
C 1.14658330 2.61976321 -0.71044164
N -0.20265008 -3.50072710 0.06134034
C -1.40396972 -3.91597216 0.78473702
C 0.42310201 -4.60759713 -0.66249096
H -1.38455065 -3.54312208 1.81186953
H -2.30426393 -3.52564933 0.29438944
C -1.34761859 -5.44212698 0.72143077
H 0.65960648 -4.31515811 -1.68850311
H 1.35369944 -4.91310376 -0.16882442
C -0.63184659 -5.71170463 -0.60374920
H -0.75254156 -5.82438437 1.55473331
H -2.33779466 -5.89439956 0.77513118
H -1.32972056 -5.60132772 -1.43778893
H -0.19065004 -6.70672515 -0.65933168
*

constrA23
C 0.06650083 -2.79960139 -0.09663819
C 1.43312858 -2.54234649 -0.32457539
C 1.99307318 -1.29905166 -0.09714922
C 1.22732440 -0.24963748 0.39948625
C -0.13999946 -0.45749265 0.59339147
C -0.68868361 -1.69166598 0.35166163
H 2.07302814 -3.34693472 -0.65868258
H 3.05542021 -1.16187231 -0.26968547
H -0.80204442 0.33511399 0.92601008
C 1.84694867 1.11148179 0.66430757
H 1.60068489 1.47096486 1.66308338
H 2.93474029 1.01057248 0.60560244
H 1.83256897 1.84391432 -1.37616423
C 0.46917536 3.05218603 -0.30138333
C 0.14883126 3.77432471 -1.48393556
C -0.17252028 3.39742331 0.92013805
C -0.76850427 4.79765326 -1.44614390
H 0.63872566 3.50510232 -2.41347726
C -1.09004090 4.41808256 0.94543323
H 0.06038567 2.85576226 1.82781403
C -1.38382260 5.11371952 -0.23358918
H -1.01536713 5.35179922 -2.34297651
H -1.58547378 4.68868425 1.86951813
H -2.10953089 5.91971237 -0.20289584
C 1.41222046 2.03352336 -0.38949255
N -0.49585579 -4.02105742 -0.32863809
C -1.62705722 -4.50687538 0.46490056
C 0.27282037 -5.03783625 -1.02673435
H -1.48667490 -5.57360070 0.64369087
H -1.66554118 -4.01068487 1.43335578
H -2.57957875 -4.35776745 -0.04773752
H -0.41202196 -5.81153670 -1.37221759
H 0.76116717 -4.61139627 -1.90414662
H 1.03154619 -5.50578712 -0.38606245
F -2.02405565 -1.81901128 0.50363971
*

constrA24
C -0.68690439 -2.34245048 -0.09275024
C 0.68745643 -2.46152544 0.07924581
C 1.44283560 -1.37749425 0.49048069
C 0.82088487 -0.15753023 0.74611440
C -0.54790516 -0.02772761 0.56380087
C -1.29674372 -1.12280228 0.14917592
H 2.51169575 -1.48997595 0.62826229
H -1.04473195 0.91921553 0.74706041
C 1.64698445 1.03956137 1.20571804
H 1.21187024 1.49150970 2.09541869
H 2.65468389 0.69450701 1.45810346
H 2.32782287 1.61698870 -0.77377836
C 1.20309438 3.24351378 -0.04379845
C 1.41565605 3.95110926 -1.26492530
C 0.42347613 3.84905156 0.98694302
C 0.87586178 5.20039745 -1.44555452
H 2.00763958 3.48541968 -2.04556097
C -0.11189429 5.09520324 0.79314096
H 0.25433838 3.32615874 1.91943594
C 0.11548667 5.76472334 -0.41806312
H 1.03211037 5.74348947 -2.36903948
H -0.70656880 5.56588257 1.56599548
H -0.31318904 6.75153148 -0.56043373
C 1.76366690 1.98655950 0.08191402
C 1.34420795 -3.79318862 -0.21697021
F 2.62299593 -3.81794003 0.19047812
F 1.33195473 -4.04379283 -1.53740781
F 0.69661620 -4.79672918 0.39135785
H -1.27407497 -3.19371366 -0.41518844
C -2.79451295 -0.96868660 0.00263689
F -3.10457086 0.23885423 -0.51145981
F -3.40500806 -1.05924456 1.19634370
F -3.31708412 -1.90259099 -0.79873733
*

constrA3
C -0.97649085 -2.52823178 1.32409246
C -0.01062303 -2.67811895 0.33540454
C 0.78905053 -1.60957811 -0.03415269
C 0.62678564 -0.37868071 0.59462012
C -0.35041781 -0.21942047 1.57051738

C -1.14496507 -1.29747542 1.94073480
H 1.54631454 -1.74370239 -0.79770874
H -0.48506337 0.74122426 2.05832599
H -1.89655363 -1.17686381 2.71155109
C 1.50698643 0.80142557 0.19413886
H 1.87487088 1.32899751 1.07219116
H 2.37137434 0.42090078 -0.36068463
H 0.45578713 1.20339401 -1.65867217
C 0.29499569 2.96152246 -0.50421216
C -0.47814042 3.57785176 -1.53143270
C 0.58326245 3.69219275 0.68549065
C -0.93862582 4.86267837 -1.37709542
H -0.69571961 3.01656123 -2.43382332
C 0.11676155 4.97320073 0.82837934
H 1.16966370 3.23804968 1.47369783
C -0.63961721 5.55260613 -0.20000971
H -1.52562412 5.33756478 -2.15307436
H 0.32916250 5.53979200 1.72637050
H -1.00297812 6.56772063 -0.07665512
C 0.74081358 1.66785691 -0.71512937
C 0.16829396 -4.03394799 -0.31031462
F 1.00222368 -3.98081343 -1.36293186
F -1.00656803 -4.51942290 -0.74502435
F 0.67156652 -4.92145923 0.56302358
H -1.59476342 -3.37194046 1.60770780
*

constrA4
C 0.12206225 -2.74407297 0.13531800
C 1.36242062 -2.49593076 -0.43520077
C 1.90936512 -1.22368808 -0.35948613
C 1.22190341 -0.20613971 0.29344412
C -0.02763870 -0.45790283 0.85237155
C -0.57536194 -1.72964971 0.77990426
H 1.90405893 -3.29433560 -0.92679828
H 2.88518513 -1.03305765 -0.79316109
H -0.57143124 0.33145408 1.36182228
H -1.53970429 -1.93569134 1.22795352
C 1.82752480 1.19135888 0.38051543
H 1.79701682 1.56715526 1.40177271
H 2.87595730 1.13727125 0.06858499
H 1.23935055 1.78698830 -1.61931937
C 0.30236219 3.14637860 -0.30668409
C -0.30655151 3.81020615 -1.41207265
C 0.04853373 3.61629371 1.01536442
C -1.12662678 4.89238748 -1.20550461
H -0.11166432 3.44671074 -2.41534613
C -0.77345033 4.69602234 1.20867896
H 0.50334147 3.12232931 1.86424953
C -1.35569484 5.32895337 0.10147116
H -1.59193601 5.40211688 -2.03969792
H -0.97454428 5.06354508 2.20718187
H -2.00396262 6.18355987 0.26613735
C 1.12456508 2.06499923 -0.57210174
C -0.50565804 -4.11555314 0.01782187
F -1.23201077 -4.41846898 1.10548548
F 0.41902005 -5.07345738 -0.14074536
F -1.33134367 -4.16931835 -1.04446368
*

constrA5
C -0.61155404 -3.68267862 -0.03853808
C 0.73282536 -3.60646616 -0.40773090
C 1.43093678 -2.43262844 -0.20959666
C 0.81263903 -1.32420067 0.37140105
C -0.53385382 -1.40067989 0.71480818
C -1.24476054 -2.57468527 0.52380386
H 1.20917867 -4.47918026 -0.83724143
H 2.47866470 -2.38540669 -0.48837961
H -1.03677102 -0.54458374 1.15464623
H -2.28954340 -2.63193529 0.81152127
C 1.59096893 -0.03527874 0.59543528
H 1.46558128 0.33154893 1.61316567
H 2.65544455 -0.24235404 0.44347535
H 1.43284640 0.68139074 -1.44193235
C 0.44031948 2.10781642 -0.23981939
C 0.10997257 2.87193315 -1.39420258
C 0.02545082 2.56689976 1.04193247
C -0.59779111 4.04391168 -1.27218968
H 0.42627300 2.51583129 -2.36875648
C -0.68299234 3.73680848 1.15143447
H 0.26780873 1.99501569 1.92828152
C -0.99041580 4.46994888 -0.00190495
H -0.85037094 4.63103215 -2.14614025
H -1.00332135 4.09700884 2.12109443
H -1.54960988 5.39470762 0.09588767
C 1.17428798 0.94193777 -0.41623905
O -1.24150995 -4.85845263 -0.25253321
H -2.15506530 -4.81263979 0.05045272
*

constrA6
C -0.07547596 -3.33982186 -0.07121024
C 1.26608815 -3.12449152 -0.41560532
C 1.84366059 -1.89181234 -0.21142623
C 1.10774652 -0.84727096 0.35666390
C -0.22836063 -1.05702101 0.67283074
C -0.82422065 -2.29627751 0.47604641
H 1.83159399 -3.94853690 -0.83329394
H 2.88651841 -1.74280790 -0.47202874
H -0.82146073 -0.25300751 1.09890738
H -1.86053433 -2.43681879 0.74995319
C 1.75273366 0.51144458 0.58990286
H 1.59044224 0.85738986 1.60982412
H 2.83254430 0.41182443 0.43901204
H 1.53553624 1.23113855 -1.44096828
C 0.37757591 2.52193301 -0.23225078
C -0.02580804 3.25973929 -1.37987389
C -0.09948492 2.91209876 1.05011458
C -0.86352311 4.34199497 -1.25060966
H 0.33875098 2.95591760 -2.35526068
C -0.93815375 3.99201181 1.16693468
H 0.19719866 2.35821305 1.93135311
C -1.31530723 4.70222213 0.02025741
H -1.17225971 4.90912245 -2.11971568
H -1.30752856 4.29885311 2.13750349
H -1.97727874 5.55572859 0.12363120
C 1.24071057 1.44808318 -0.41496635
O -0.54563752 -4.57733331 -0.30417851
C -1.89999164 -4.86058317 0.04024230
H -2.06628129 -4.72207983 1.11221406
H -2.58768438 -4.23089608 -0.53135941
H -2.05916122 -5.90404363 -0.22130787
*

constrA7
C -0.18255050 -3.39846472 -0.04066267
C 1.09733244 -3.18839778 -0.55825699
C 1.72240412 -1.95801564 -0.44158503
C 1.07898750 -0.90834767 0.20594858
C -0.21207460 -1.09371796 0.69642745
C -0.83140891 -2.32610311 0.57910628
H 1.61396439 -4.00703257 -1.04735948
H 2.72390494 -1.82306742 -0.83706855
H -0.73632647 -0.27527587 1.18112866
H -1.83767243 -2.45229483 0.95960585
C 1.76517989 0.44611038 0.34008831
H 1.72891355 0.80401059 1.36799788
H 2.81683809 0.33028149 0.05873072
H 1.30817453 1.14180300 -1.65971447
C 0.33127013 2.46932678 -0.33924378
C -0.19973797 3.19664672 -1.44203151

C	0.01880348	2.88556272	0.98601777
C	-1.00208068	4.29192039	-1.22953778
H	0.03932810	2.87293870	-2.44932992
C	-0.78597900	3.97830762	1.18589575
H	0.41413689	2.34088497	1.83361266
C	-1.29108362	4.67653036	0.08129190
H	-1.40791791	4.85069726	-2.06339984
H	-1.03109054	4.30505168	2.18875124
H	-1.92524961	5.54064837	0.25050342
C	1.14520604	1.37819535	-0.60879584
C	-0.80200850	-4.73202592	-0.16739825
C	-1.82196320	-5.17939784	0.55641481
H	-0.34725469	-5.38820056	-0.90456674
H	-2.21316579	-6.17825010	0.40564738
H	-2.29243506	-4.58279421	1.33053267

*

constrA8

C	-0.28435169	-3.48025088	0.10874885
C	0.96145038	-3.31898593	-0.50235012
C	1.60602532	-2.09491212	-0.45684383
C	1.02391419	-1.02078433	0.21061394
C	-0.22537546	-1.17162615	0.80645195
C	-0.87262729	-2.39564794	0.76458598
H	1.42015128	-4.16115532	-1.00563603
H	2.57819343	-1.98352847	-0.92548574
H	-0.69212551	-0.33752627	1.32163764
H	-1.83784327	-2.52209357	1.23925447
C	1.73940515	0.32429820	0.26632415
H	1.78750121	0.70006604	1.28716578
H	2.76459028	0.18779384	-0.09337975
H	1.12431830	0.98136676	-1.70483959
C	0.31175003	2.37482068	-0.34309499
C	-0.29379745	3.08947868	-1.41642722
C	0.13268757	2.83808593	0.99211154
C	-1.03959936	4.21600472	-1.16666010
H	-0.15630819	2.73041828	-2.43072143
C	-0.61599768	3.96231550	1.22918969
H	0.58560982	2.30436281	1.81758561
C	-1.19693197	4.64600229	0.15269264
H	-1.50172830	4.76491248	-1.97745340
H	-0.75953231	4.32510324	2.23930017
H	-1.78633117	5.53522547	0.35135416
C	1.06241496	1.25073154	-0.65099282
C	-0.95471799	-4.74926612	0.06461538
C	-1.51427449	-5.80801904	0.02625998
H	-2.01003262	-6.75212629	-0.00580683

*

constrA9

C	-0.27889413	-3.51477903	0.05122830
C	1.01597830	-3.34340558	-0.43429958
C	1.63914242	-2.11393145	-0.29878692
C	0.98037132	-1.05995213	0.32777867
C	-0.31763362	-1.23175399	0.80032673
C	-0.94736755	-2.45921552	0.67034414
H	1.52778967	-4.17195035	-0.90811148
H	2.65125387	-1.98407447	-0.66649834
H	-0.83849633	-0.41300706	1.28657369
H	-1.95266492	-2.60646441	1.04531263
C	1.67302628	0.29031725	0.47989451
H	1.59732675	0.65418780	1.50308384
H	2.73470814	0.16864166	0.23975515
H	1.25772601	0.94538495	-1.54558550
C	0.34960050	2.36366961	-0.27641219
C	-0.13508169	3.08249114	-1.40931538
C	0.05201563	2.84324944	1.03358898
C	-0.87805926	4.22491841	-1.24026350
H	0.09269664	2.71215519	-2.40312586
C	-0.69194589	3.98363497	1.18905382
H	0.41284456	2.30892559	1.90294774
C	-1.15249588	4.66886330	0.05563266
H	-1.24877224	4.77635463	-2.09507482
H	-0.92478571	4.35922872	2.17759710
H	-1.74001918	5.57128414	0.19060895
C	1.09902851	1.22338261	-0.50411685
C	-0.93520895	-4.79069521	-0.08857365
N	-1.46256226	-5.80490715	-0.20374663

*

constrB1

C	-0.34082153	-3.78370778	0.08206382
C	0.26839834	-3.28563486	1.24601058
C	0.78352177	-2.01714721	1.26334677
C	0.71270955	-1.18645361	0.10548944
C	0.08921133	-1.70591844	-1.06969335
C	-0.42217301	-2.97150152	-1.07074650
H	0.32380430	-3.91219878	2.12803868
H	1.25234309	-1.62601101	2.16010335
H	0.01940360	-1.09488779	-1.96045366
H	-0.89789316	-3.36751273	-1.96057852
C	1.25270981	0.08128037	0.17357328
H	1.69227963	0.37356520	1.12640849
H	2.28596111	1.50001724	-0.95414819
H	0.94433770	0.76142956	-1.83528053
C	0.36968074	2.27610495	-0.39317403
C	-1.01382969	2.12338509	-0.38345818
C	0.93952086	3.46818731	0.04352193
C	-1.82368841	3.17071580	0.03478575
H	-1.46395916	1.19268691	-0.71628144
C	0.12842915	4.51316743	0.46257727
H	2.01787294	3.58955595	0.03721109
C	-1.25281894	4.36521826	0.45800141
H	-2.90125279	3.05491541	0.02947030
H	0.57550347	5.44563014	0.78675229
H	-1.88649523	5.18230257	0.78260726
C	1.26006974	1.12964932	-0.86027848
C	-0.88937459	-5.17173199	0.04365243
H	-1.87654866	-5.18262513	-0.42341472
H	-0.23515376	-5.79651341	-0.57391315
H	-0.95113340	-5.61408707	1.03680534

*

constrB10

C	-0.76722615	-3.74966841	-0.33343122
C	0.16122733	-3.46483236	0.68004092
C	0.76104580	-2.22581720	0.64900804
C	0.45509210	-1.27665747	-0.36966995
C	-0.49278470	-1.61070336	-1.37619454
C	-1.09492608	-2.84308713	-1.34684693
H	1.48474204	-1.95265883	1.41047815
H	-0.73750916	-0.90127711	-2.15566873
H	-1.81992005	-3.12045037	-2.10198788
C	1.10581021	-0.05400625	-0.33660006
H	1.80358080	0.10378591	0.48507262
H	1.92802127	1.42283674	-1.55389497
H	0.35162113	0.85943936	-2.12087555
C	0.28709818	2.23250374	-0.44196695
C	-1.05307580	2.14884612	-0.07521768
C	1.04295860	3.33986430	-0.06860376
C	-1.64170771	3.18577959	0.63626072
H	-1.64490163	1.28278399	-0.35652777
C	0.45270314	4.37396972	0.64340384
H	2.08821854	3.40586758	-0.35241899
C	-0.88902392	4.29741046	0.99595226
H	-2.68864443	3.12612871	0.90985607
H	1.03945609	5.24280001	0.91773384
H	-1.35043640	5.10629368	1.55033005
C	0.93317241	1.09819704	-1.23185285
H	-1.25414509	-4.72090756	-0.32912565
C	0.46878101	-4.48401212	1.74315778
H	1.20917916	-4.10820629	2.44922257

H	-0.43508476	-4.74237796	2.29913062
H	0.85733177	-5.40063523	1.29410768

*

constrB11

C	-0.08505135	-3.75536828	-0.93102657
C	-0.63263255	-3.47823949	0.32705532
C	-0.34845571	-2.26276884	0.91954783
C	0.48668940	-1.32501678	0.25893341
C	1.03158927	-1.63042705	-1.01734261
C	0.73636215	-2.84102481	-1.59503852
H	-0.76900396	-2.03335232	1.89215271
H	1.66869724	-0.92211302	-1.52878612
H	1.13787176	-3.09903354	-2.56709818
C	0.74249173	-0.12215507	0.90771637
H	0.26551722	0.01900608	1.87698064
H	2.20658300	1.32108124	1.22787936
H	2.11373784	0.79154208	-0.45683327
C	0.56471830	2.18190923	0.15831323
C	-0.29104178	2.13549585	-0.93850246
C	0.53094359	3.27146850	1.02384775
C	-1.15420233	3.19448486	-1.18646733
H	-0.27591815	1.28216404	-1.61000859
C	-0.33344590	4.32717990	0.77408384
H	1.19524370	3.30687879	1.88106219
C	-1.17595042	4.28899777	-0.33027864
H	-1.81080238	3.16476877	-2.04808727
H	-0.34510265	5.18272117	1.43910471
H	-1.85014454	5.11468429	-0.52574437
C	1.52183105	1.02331895	0.42696730
H	-0.30430175	-4.70805840	-1.40552300
O	-1.42883371	-4.33315626	0.99833673
H	-1.56675948	-5.15057806	0.50591428

*

constrB12

C	-0.03835276	-3.07271736	-0.26605773
C	0.29256758	-2.61238081	1.03022129
C	0.71466639	-1.33043928	1.21643017
C	0.83683579	-0.42376815	0.11501972
C	0.49775027	-0.90173802	-1.19018975
C	0.07437439	-2.18410241	-1.36526913
H	0.20955217	-3.27922040	1.87887761
H	0.96635220	-0.97641419	2.21054487
H	0.57638407	-0.24101372	-2.04402594
H	-0.18196781	-2.54399340	-2.35451689
C	1.27172265	0.85630793	0.35882820
H	1.48783052	1.11027540	1.39559635
H	2.42871508	2.39211563	-0.46270035
H	1.33813667	1.64688026	-1.63451802
C	0.39523565	3.03998351	-0.26806259
C	-0.94990615	2.82165955	-0.55146332
C	0.79074534	4.23021177	0.33437278
C	-1.88949989	3.80025860	-0.25669887
H	-1.26633004	1.89213507	-1.01574588
C	-0.14977779	5.20704630	0.63029550
H	1.83874752	4.40412022	0.55615861
C	-1.48975136	4.99321233	0.33425160
H	-2.93461086	3.63266098	-0.48983551
H	0.16567455	6.13820119	1.08637478
H	-2.22412307	5.75704186	0.56199797
C	1.42837800	1.96943860	-0.59818501
C	-0.49240964	-4.43355287	-0.50880361
C	0.05952176	-5.58156096	0.33608481
C	-1.33779739	-5.15363732	0.54125126
H	-0.71509191	-4.66501671	-1.54219454
H	0.80198948	-5.32668762	1.08155961
H	0.24998111	-6.50033358	-0.20297146
H	-2.13502768	-5.77005411	0.14692034
H	-1.59121157	-4.59491592	1.43324057

*

constrB13

C	-0.72743843	-3.44292118	-0.73218597
C	0.18185335	-3.20787929	0.30869122
C	0.79079346	-1.98086255	0.40416546
C	0.50273087	-0.96029814	-0.54605277
C	-0.42534938	-1.22992288	-1.59167654
C	-1.03113349	-2.46101688	-1.67718493
H	1.49666728	-1.77784466	1.20467802
H	-0.65560870	-0.46371068	-2.32088246
H	-1.73764538	-2.67231166	-2.47010144
C	1.14658800	0.26225310	-0.41356882
H	1.82908972	0.36261415	0.42978124
H	1.97865656	1.83102948	-1.49804973
H	0.42029320	1.29794669	-2.14011794
C	0.30736723	2.54178671	-0.36470023
C	-1.04179009	2.42466145	-0.04233482
C	1.04884656	3.61749638	0.11541620
C	-1.65462678	3.40168730	0.73083504
H	-1.62224833	1.58235171	-0.40717813
C	0.43394580	4.59113496	0.88871780
H	2.10112500	3.70835745	-0.13329174
C	-0.91686171	4.48349580	1.19663038
H	-2.70835749	3.31841089	0.96974261
H	1.00843787	5.43767515	1.24598195
H	-1.39711605	5.24545377	1.79932689
C	0.98054755	1.47407775	-1.22324639
H	-1.19415807	-4.42225298	-0.78228695
C	0.46886007	-4.30110510	1.29786802
O	-0.05789061	-5.37618051	1.22907470
H	1.19627922	-4.05091859	2.09287569

*

constrB14

C	0.20295239	-3.13892783	0.02006461
C	0.31823890	-2.64615770	1.31187551
C	0.64724149	-1.31691658	1.46042283
C	0.86109975	-0.49700874	0.32087811
C	0.73146542	-1.05319598	-0.97960634
C	0.39959757	-2.37854985	-1.12920973
H	0.14937400	-3.30070176	2.15611652
H	0.74525494	-0.88674280	2.45101814
H	0.89132952	-0.43971955	-1.85645653
H	0.29007380	-2.84097543	-2.10077569
C	1.20125624	0.83988055	0.53300503
H	1.26325499	1.16469062	1.57126524
H	2.34600066	2.41026216	-0.20100262
H	1.51771181	1.50309120	-1.47391518
C	0.26796722	2.89170206	-0.36660302
C	-0.99548300	2.54258761	-0.83573766
C	0.47988198	4.13903189	0.21457866
C	-2.03616944	3.45860379	-0.75893626
H	-1.16724580	1.56628096	-1.27912779
C	-0.56248230	5.05032554	0.29050703
H	1.46355120	4.40872106	0.58455873
C	-1.81980654	4.71025738	-0.19505086
H	-3.01627895	3.19457084	-1.13825980
H	-0.39197346	6.02885545	0.72364908
H	-2.63348771	5.42360846	-0.13593081
C	1.41786886	1.89008020	-0.46109030
N	-0.15783355	-4.58226259	-0.15009263
O	-0.31118092	-5.22163512	0.86384577
O	-0.26423381	-4.97980750	-1.28668018

*

constrB15

C	-0.02026083	-3.36627481	-0.09665832
C	0.30109681	-2.89029890	1.18191603
C	0.72916403	-1.60157643	1.34297480
C	0.85084857	-0.75323077	0.20714906
C	0.52487515	-1.24312298	-1.08808918

C 0.09538570 -2.53145948 -1.22271943
H 0.97138915 -1.23975752 2.33557918
H 0.60648435 -0.62397206 -1.97138321
C 1.29412454 0.54602649 0.41460128
H 1.50486140 0.82746662 1.44539823
H 2.43496799 2.07419592 -0.41763945
H 1.39651130 1.26674890 -1.59776778
C 0.38542341 2.68258971 -0.29970905
C -0.94322251 2.43300922 -0.63179809
C 0.74131935 3.88637055 0.30185151
C -1.90777927 3.40325184 -0.39541542
H -1.22695250 1.49146665 -1.09278397
C -0.22520020 4.85262327 0.53847851
H 1.77635666 4.08008777 0.56371668
C -1.54884444 4.61141996 0.19043189
H -2.93962294 3.21616491 -0.66848824
H 0.05626457 5.79707528 0.98906985
H -2.30303226 5.36773915 0.37383572
C 1.44969768 1.62130764 -0.56939503
F 0.17450284 -3.71653176 2.20649505
F -0.43091617 -4.59249450 -0.24596076
F -0.22380434 -3.03829904 -2.40190653
*

constrB16
C 0.10611834 -3.54976835 0.44445366
C 1.20300510 -2.97879258 1.10992442
C 1.56218408 -1.70013482 0.80386545
C 0.84824485 -0.93854366 -0.17640288
C -0.27224656 -1.54359111 -0.84243769
C -0.63196089 -2.81431734 -0.53980982
H 1.72895499 -3.57257902 1.84652582
H 2.40441618 -1.23733377 1.30585458
H -0.82865780 -0.98480099 -1.58514546
C 1.27242719 0.33914409 -0.43196256
H 2.12598709 0.69329145 0.14301816
H 1.48371245 1.72140153 -1.98931828
H -0.08415016 0.91186749 -1.99504397
C 0.12312917 2.48551606 -0.51926141
C -1.01627154 2.29425882 0.25730999
C 0.75791444 3.72345455 -0.52064870
C -1.52981400 3.34237208 1.00885963
H -1.51301586 1.32830575 0.26905600
C 0.24448926 4.77064842 0.23218193
H 1.64615560 3.87693118 -1.12491439
C -0.89966066 4.58134081 0.99613751
H -2.42316505 3.19320624 1.60419477
H 0.73571310 5.73659180 0.21696341
H -1.30247978 5.39943063 1.58181084
C 0.68339200 1.33746711 -1.34936549
O -0.24561767 -4.77820867 0.73270228
H -1.01475252 -5.04357700 0.19921736
O -1.65583795 -3.52698477 -1.07169225
H -2.13561042 -3.03161197 -1.74498868
*

constrB17
C -0.03390246 -2.94745329 -0.58361490
C -0.05478719 -2.55413568 0.80433276
C 0.32321056 -1.28801019 1.12755080
C 0.73896293 -0.35084802 0.11642734
C 0.74256493 -0.77220783 -1.24782360
C 0.36583190 -2.03646767 -1.58859147
H 0.31921266 -0.95770370 2.15795411
H 1.04652802 -0.08292100 -2.02426457
H 0.37260503 -2.34709471 -2.62353980
C 1.11841265 0.90971172 0.49089216
H 1.07229142 1.13985946 1.55423340
H 2.49370707 2.41490517 0.01290129
H 1.67674958 1.75840729 -1.40527981
C 0.49612891 3.15047197 -0.23954195
C -0.75864132 2.99885454 -0.82276546
C 0.78994864 4.30999335 0.47081023
C -1.70371956 4.00995293 -0.71425824
H -0.99755869 2.09392884 -1.37402833
C -0.15619169 5.31995039 0.58118818
H 1.76764343 4.43377502 0.92541183
C -1.40281332 5.17140326 -0.01223555
H -2.67560113 3.89275783 -1.17962855
H 0.08366213 6.22610181 1.12529161
H -2.14067614 5.96098482 0.06988041
C 1.53536259 2.04447730 -0.36393985
O -0.40490248 -4.17427422 -0.81373158
C -0.42376505 -4.69095160 -2.16208425
H -1.12485633 -4.11903922 -2.77133355
H 0.58093976 -4.66435345 -2.58592858
H -0.76287923 -5.71796821 -2.06370619
O -0.45833688 -3.51399449 1.64492025
C -0.50219011 -3.19056247 3.03645612
H 0.49425944 -2.92603260 3.40154272
H -1.20434875 -2.37221002 3.21935793
H -0.84932349 -4.09090109 3.53648223
*

constrB18
C -0.00425022 -3.07169733 0.04368223
C 0.97971306 -2.51560574 0.93592909
C 1.37876978 -1.23389631 0.79401789
C 0.85308367 -0.37426018 -0.23746678
C -0.13505914 -0.94454598 -1.11858618
C -0.54658217 -2.22323733 -0.98969357
H 1.40133736 -3.12372494 1.72219522
H 2.12013260 -0.82510033 1.47238625
H -0.56184896 -0.33389781 -1.90400794
H -1.28961855 -2.61387012 -1.66897304
C 1.29948999 0.90681012 -0.33129786
H 2.03866714 1.22541209 0.40157178
H 1.75774407 2.37738724 -1.76119630
H 0.21206332 1.58383069 -2.06024612
C 0.17777606 3.06881857 -0.48637162
C -1.07600740 2.84772628 0.07668553
C 0.80150012 4.29933457 -0.30877810
C -1.70648962 3.85469147 0.79448551
H -1.56875205 1.88831320 -0.05275417
C 0.17217142 5.30645563 0.41108193
H 1.77779349 4.47907064 -0.74755725
C -1.08285288 5.08589215 0.96192208
H -2.68725847 3.68022155 1.22197040
H 0.66076187 6.26594683 0.53553221
H -1.57702553 5.87232781 1.52050781
C 0.86664070 1.96659839 -1.27778471
N -0.40222616 -4.32691044 0.16731595
C 0.14599066 -5.19205913 1.21989368
C -1.40357875 -4.89753852 -0.74314752
H -0.31677913 -6.17131155 1.14335742
H 1.22461052 -5.30680267 1.09996497
H -0.07174995 -4.78117175 2.20712415
H -1.57306011 -5.93457952 -0.46964190
H -2.34893473 -4.35829968 -0.66237453
H -1.04876434 -4.86451688 -1.77452320
*

constrB19
C 0.00957891 -2.52203923 -0.01815809
C 0.12799173 -2.07349592 1.29864541
C 0.55249972 -0.77497935 1.50971715
C 0.86285195 0.06622091 0.41434030
C 0.73438768 -0.42495977 -0.90722182
C 0.30427978 -1.71753944 -1.12561510
H 0.64631820 -0.40328917 2.52403821
H 0.96677437 0.20010751 -1.75933246
C 1.29612757 1.37101912 0.68768589

H 1.35023348 1.65051304 1.73954596
H 2.55257495 2.89517655 0.05568668
H 1.73255348 2.07635601 -1.28050436
C 0.51352430 3.49666569 -0.17698610
C -0.74002571 3.24077534 -0.72703898
C 0.76392932 4.70007595 0.47781855
C -1.72909791 4.21307548 -0.66362190
H -0.94249891 2.29802672 -1.22660761
C -0.22778517 5.66642663 0.53996458
H 1.73879306 4.89406468 0.91258839
C -1.47328073 5.42256689 -0.02866153
H -2.69940215 4.02524982 -1.10773986
H -0.02731577 6.61321030 1.02732976
H -2.24729495 6.17940805 0.02122847
C 1.61040728 2.43340640 -0.25887346
N -0.44897803 -3.90298728 -0.25538663
O 0.41539861 -4.74184824 -0.23660742
O -1.63351074 -4.02439566 -0.44037533
C -0.18946903 -2.93584318 2.40540768
N -0.44387312 -3.61391599 3.29583901
C 0.16349801 -2.22733312 -2.46375358
N 0.05651512 -2.61994655 -3.53697773
*

constrB2
C -0.00968179 -3.42457211 -0.04625780
C 0.56438362 -2.94707550 1.14028235
C 1.00108296 -1.65093277 1.18013361
C 0.87992962 -0.80061785 0.04046810
C 0.28915136 -1.32481154 -1.14809316
C -0.14968244 -2.61788839 -1.18990208
H 0.65075618 -3.60330245 1.99633152
H 1.44699221 -1.25494356 2.08613905
H 0.18486796 -0.69824389 -2.02438437
H -0.60012289 -3.03450498 -2.08136260
C 1.34523335 0.49709990 0.13713397
H 1.76808714 0.79235287 1.09659407
H 2.28843740 2.00275647 -0.95282756
H 0.99563612 1.20524439 -1.85500190
C 0.32833920 2.64547173 -0.37620567
C -1.04329103 2.40821408 -0.37379357
C 0.82527428 3.85784952 0.09287217
C -1.91554027 3.39384775 0.06830309
H -1.43622427 1.46109321 -0.73180391
C -0.04826954 4.84064379 0.53558912
H 1.89428855 4.04424806 0.09268498
C -1.41815958 4.60932721 0.52325878
H -2.98409729 3.21346120 0.05694597
H 0.34086864 5.79003797 0.88430855
H -2.10048911 5.37809915 0.86664928
C 1.28740851 1.56704330 -0.87013587
Cl -0.55668440 -5.03199744 -0.10773521
*

constrB20
C 0.07545592 -1.96428362 -0.01564955
C 0.36626693 -1.50695234 1.27217898
C 0.79615507 -0.20767286 1.41693321
C 0.93185369 0.64056522 0.29219066
C 0.62469368 0.13949767 -0.99558433
C 0.20072274 -1.15710940 -1.15479726
H 1.03231344 0.16960154 2.40512786
H 0.71816802 0.77119468 -1.86832884
C 1.36879294 1.95181526 0.50142091
H 1.56895508 2.23460566 1.53454178
H 2.48174194 3.50898016 -0.30465905
H 1.50028453 2.66303948 -1.50865504
C 0.41321544 4.05340954 -0.24422292
C -0.89308907 3.77069560 -0.63423943
C 0.71364030 5.25642477 0.38927068
C -1.89071512 4.71452646 -0.43059788
H -1.13284916 2.82836512 -1.11786108
C -0.28655289 6.19476513 0.59256034
H 1.73096521 5.47233420 0.69851471
C -1.58752577 5.92369256 0.18398632
H -2.90463506 4.50513361 -0.75026644
H -0.04962370 7.14083000 1.06472715
H -2.36808803 6.65844142 0.34304796
C 1.51698794 3.02160874 -0.48045843
C 0.20546092 -2.38032731 2.51745989
F 0.93647168 -1.87301020 3.51951929
F 0.62038144 -3.62676603 2.28803697
F -1.06843855 -2.41076564 2.90700563
C -0.09911153 -1.67001957 -2.56431417
F -0.32069126 -0.63125000 -3.38355946
F -1.18473603 -2.44453315 -2.57376453
F 0.93350901 -2.36182162 -3.04290254
N -0.38574037 -3.36316094 -0.18563912
O -1.50951026 -3.58500640 0.18665855
O 0.41727706 -4.11452175 -0.67681194
*

constrB21
C 0.05642967 -2.75634783 -0.00941366
C -0.19327479 -2.27886871 1.27204767
C 0.12185477 -0.98065194 1.59394657
C 0.70668499 -0.14814230 0.60680919
C 0.96162887 -0.64182606 -0.69736401
C 0.62896593 -1.93995414 -0.98511933
H -0.07931868 -0.61128724 2.59273158
H 1.40762834 -0.02956222 -1.46939252
C 1.02291970 1.16412940 0.97393444
H 0.77687787 1.45263299 1.99519851
H 2.39250912 2.69876796 0.69662674
H 1.97457505 1.86448861 -0.80573233
C 0.48876702 3.27815131 -0.08896494
C -0.56653273 3.00516951 -0.95530411
C 0.54198926 4.49047540 0.59401480
C -1.54515049 3.96616384 -1.17123499
H -0.61785260 2.05542602 -1.47928589
C -0.43861850 5.44608892 0.37661367
H 1.36148074 4.69937032 1.27369651
C -1.48173677 5.18384482 -0.50440874
H -2.35785947 3.76325954 -1.85859524
H -0.38579500 6.39866307 0.89038395
H -2.24717120 5.93206696 -0.67381450
C 1.57527634 2.22737778 0.14010500
N -0.28989634 -4.14930859 -0.34576394
O 0.57344643 -4.96199919 -0.13298940
O -1.39613254 -4.31133325 -0.79500148
F 0.84192472 -2.44870631 -2.18690354
F -0.73709767 -3.09120399 2.16155062
*

constrB22
C 0.21962515 -2.37619458 -0.04063197
C 1.19282809 -1.78804103 0.84490398
C 1.52127847 -0.48620725 0.72008431
C 0.93431106 0.36298553 -0.28996834
C -0.04149317 -0.24246030 -1.16504536
C -0.38810636 -1.54009878 -1.04910546
H 1.64406052 -2.39057814 1.62001894
H 2.24739398 -0.04770023 1.39639867
H -0.50577154 0.35795105 -1.93697588
H -1.11279967 -1.96809935 -1.72691716
C 1.31669478 1.66375358 -0.37121200
H 2.05314011 2.00681981 0.35323871
H 1.68115254 3.16365130 -1.79929879
H 0.15738547 2.31747922 -2.06344309
C 0.11309084 3.78522937 -0.47448292
C -1.11697674 3.51433126 0.11845111
C 0.69675789 5.03560231 -0.29953521

C -1.76351320 4.49071905 0.86368854
H -1.57840562 2.53921041 -0.00890385
C 0.05162228 6.01235295 0.44783271
H 1.65419679 5.25449192 -0.76152796
C -1.17985621 5.74175765 1.02854618
H -2.72590482 4.27694082 1.31452492
H 0.50921492 6.98728459 0.57032871
H -1.68671286 6.50435372 1.60844785
C 0.81928189 2.71659883 -1.29541804
N -0.10847801 -3.64453306 0.06574515
C -1.14817872 -4.31209270 -0.74805493
C 0.51849400 -4.59195407 1.01315774
H -2.05347196 -3.70586923 -0.77160887
H -0.77721170 -4.44007594 -1.76892484
C -1.34815558 -5.65316472 -0.04693734
H 1.60302335 -4.48987088 0.98297679
H 0.16714547 -4.37210583 2.02532506
C 0.03476094 -5.95643768 0.52996789
H -2.08122859 -5.54828989 0.75650289
H -1.70447331 -6.41906224 -0.73456546
H 0.69946127 -6.33181096 -0.25179979
H 0.01292673 -6.68680586 1.33771517
*

constrB23
C -0.19482645 -2.86843176 -0.06339957
C 1.13468432 -2.33518482 0.14416097
C 1.46141100 -1.07102225 -0.17027910
C 0.49780110 -0.16790565 -0.74601371
C -0.81740502 -0.69613034 -0.99029236
C -1.14044120 -1.97080837 -0.68262115
H 2.48182025 -0.74298223 -0.00644476
H -1.57729826 -0.05539225 -1.41833553
H -2.14823628 -2.31579736 -0.85731004
C 0.86367671 1.11058922 -1.03315581
H 1.88533322 1.39756023 -0.79107099
H 0.54410534 2.63064503 -2.44830048
H -0.94869320 1.85379351 -1.92375071
C -0.12875424 3.27969592 -0.51775781
C -0.92577114 3.04747164 0.59956073
C 0.53677908 4.49449139 -0.64523194
C -1.07047427 4.02938202 1.56991255
H -1.44518468 2.09977675 0.70960446
C 0.39438821 5.47626441 0.32651960
H 1.15755291 4.68241925 -1.51546274
C -0.41053259 5.24546490 1.43377065
H -1.70095662 3.84747066 2.43273044
H 0.90782343 6.42418816 0.21422586
H -0.52591774 6.01238068 2.19086650
C 0.02527529 2.20514112 -1.58452805
N -0.54986834 -4.09482057 0.27337240
C 0.23224106 -4.98146517 1.15555043
C -1.83600874 -4.65291835 -0.16823527
H -0.47404872 -5.64023324 1.65717038
H 0.93660968 -5.57990131 0.57662313
H 0.76456613 -4.40852168 1.90663869
H -1.76860399 -5.73663737 -0.10787606
H -2.65061698 -4.31092349 0.47486956
H -2.03780555 -4.38274298 -1.20295264
F 2.09134046 -3.15477164 0.61071769
*

constrB24
C -0.06550547 -2.44883353 -0.15496936
C 0.15066175 -2.04472038 1.16176537
C 0.60059896 -0.77131203 1.41652923
C 0.84744146 0.12235513 0.34106047
C 0.62337182 -0.30796810 -0.99354074
C 0.16911570 -1.58096820 -1.22644816
H 0.76926343 -0.44412518 2.43616174
H 0.80483817 0.35802106 -1.82627104
C 1.30685102 1.40284357 0.64444700
H 1.43109659 1.63418800 1.70192306
H 2.54557946 2.94130190 -0.00246956
H 1.62141571 2.19863265 -1.31470597
C 0.50359531 3.58010949 -0.06826382
C -0.78032830 3.37802433 -0.56684069
C 0.80614388 4.73932360 0.64083839
C -1.74951079 4.35639594 -0.39052678
H -1.02291569 2.47036383 -1.11153428
C -0.16550073 5.71297154 0.81687365
H 1.80588035 4.89405275 1.03313611
C -1.44236623 5.52155383 0.30213319
H -2.74458394 4.20860128 -0.79322878
H 0.07585476 6.62455862 1.35067963
H -2.20049719 6.28376372 0.43894219
C 1.57579537 2.51039838 -0.27234789
H -0.42265692 -3.45544711 -0.35322806
C -0.13341096 -3.02763555 2.28377498
F 0.22003868 -2.52180979 3.47060397
F 0.54317094 -4.16571288 2.08822673
F -1.43880798 -3.32326674 2.32137598
C -0.08502330 -2.08586578 -2.63568131
F 0.11747184 -1.12059905 -3.54034157
F -1.34683360 -2.51791552 -2.75341296
F 0.73083810 -3.10848638 -2.91806324
*

constrB3
C -0.73343283 -2.71170277 -1.42676995
C -0.01661449 -2.62109123 -0.22911402
C 0.60164567 -1.44321824 0.10393999
C 0.51261695 -0.32388911 -0.77000487
C -0.22215837 -0.43961792 -1.98147429
C -0.83762176 -1.62652133 -2.29959072
H 1.15856150 -1.35724696 1.02988547
H -0.29825738 0.40552605 -2.65331438
H -1.39984896 -1.72746819 -3.21929480
C 1.16268315 0.84658906 -0.39553945
H 1.68817897 0.82529599 0.55870390
H 2.22315930 2.47068080 -1.14415116
H 0.78411900 2.08750615 -2.09731143
C 0.40443498 3.14878381 -0.24177149
C -0.98404048 3.07479305 -0.17524657
C 1.08767276 4.12402077 0.47921270
C -1.68889962 3.99934898 0.58391141
H -1.52042054 2.30937447 -0.72829788
C 0.38049297 5.04503352 1.23768275
H 2.17019549 4.17976987 0.43041078
C -1.00686008 4.98285600 1.29034124
H -2.77073402 3.95154260 0.62442187
H 0.91253115 5.81554532 1.78313708
H -1.55882456 5.70380072 1.88197967
C 1.18081173 2.13983024 -1.08461195
H -1.21927064 -3.64905556 -1.67964319
C 0.05587695 -3.83916404 0.67168174
F 0.75087187 -3.58101589 1.78556645
F 0.64633922 -4.85796527 0.03245909
F -1.17539051 -4.23330087 1.02410879
*

constrB4
C 0.17106406 -2.74503012 0.00685521
C 0.62374575 -2.24929057 1.22683181
C 1.02284304 -0.93347216 1.29933394
C 0.97524150 -0.10224842 0.14879884
C 0.50917659 -0.64083842 -1.08246312
C 0.11114366 -1.95113687 -1.14613332
H 0.65386996 -2.89013708 2.09807668
H 1.37625145 -0.51938640 2.23735442
H 0.46569861 -0.02100630 -1.96840644
H -0.25009108 -2.37721541 -2.07398568

C 1.39487680 1.21807749 0.27978801
H 1.72237234 1.52674373 1.27206709
H 2.36789585 2.76785160 -0.70802570
H 1.22007145 1.90298682 -1.73868056
C 0.32956916 3.30912373 -0.34475036
C -1.02110589 2.99868560 -0.47646601
C 0.71648063 4.54627583 0.16281736
C -1.98209602 3.94077915 -0.13454811
H -1.32726465 2.03076055 -0.86214390
C -0.24627157 5.48436105 0.50428778
H 1.76914090 4.78686432 0.26912964
C -1.59466435 5.18188823 0.35638286
H -3.03351265 3.70559984 -0.25072904
H 0.05642647 6.45439232 0.88068753
H -2.34620030 5.91597397 0.62244045
C 1.38850304 2.27851398 -0.73059421
C -0.25450636 -4.20391182 -0.11473628
F -1.41957640 -4.29367497 -0.76635214
F -0.39235409 -4.78028540 1.08047101
F 0.67015418 -4.87898694 -0.80931165
*

constrB5
C -0.33260932 -3.80937482 0.06030370
C 0.32055496 -3.33286326 1.21939712
C 0.82649678 -2.07183924 1.21391575
C 0.71240244 -1.22490087 0.06054732
C 0.04131794 -1.74261997 -1.09517057
C -0.46939448 -3.00204292 -1.09728148
H 0.39785571 -3.98685473 2.07858231
H 1.33013667 -1.68505920 2.09328227
H -0.06291348 -1.12538820 -1.97814958
H -0.97789317 -3.39792846 -1.96983156
C 1.24834607 0.03675669 0.11077988
H 1.72556512 0.32755474 1.04545317
H 2.23751575 1.45317962 -1.06619987
H 0.84765918 0.73277679 -1.88109438
C 0.35772718 2.24374617 -0.40639139
C -1.02447579 2.10149779 -0.32390500
C 0.95589707 3.43260650 -0.00068782
C -1.80479665 3.15246110 0.13857573
H -1.49792049 1.17415890 -0.63339776
C 0.17492788 4.48205674 0.46326413
H 2.03311964 3.54775720 -0.06448249
C -1.20523147 4.34311958 0.53225916
H -2.88186155 3.04316282 0.19036691
H 0.64478282 5.41096041 0.76474971
H -1.81566071 5.16349548 0.89129222
C 1.21422869 1.09328951 -0.92077226
O -0.80528592 -5.03773227 0.11608526
H -1.23820184 -5.30081553 -0.70849238
*

constrB6
C 0.01704725 -3.41273550 0.27456360
C 0.86079908 -2.83970030 1.26389672
C 1.26993004 -1.55529637 1.12696283
C 0.86875219 -0.76033877 -0.00198408
C 0.01445012 -1.36713784 -0.97979704
C -0.40388141 -2.65476805 -0.85236715
H 1.15273003 -3.45529334 2.10522844
H 1.91330669 -1.10578188 1.87561689
H -0.30768307 -0.79161045 -1.83823952
H -1.04762343 -3.09534478 -1.60021166
C 1.31547923 0.53024910 -0.09890347
H 1.95197996 0.88871631 0.70870310
H 1.94956950 1.94297259 -1.50591832
H 0.46191766 1.12126704 -1.98047589
C 0.21031420 2.66745084 -0.48344512
C -1.11218151 2.46089740 -0.10167401
C 0.81000216 3.90172279 -0.25411224
C -1.83582607 3.49034425 0.48471982
H -1.58574733 1.49844324 -0.27292214
C 0.08638806 4.93008299 0.33394503
H 1.84037478 4.06775667 -0.55170492
C -1.23679013 4.72564857 0.70251625
H -2.86894707 3.32950580 0.77033452
H 0.55502243 5.89342580 0.49805301
H -1.80345908 5.52914433 1.15843782
C 1.00276102 1.54036395 -1.13288772
O -0.32707506 -4.65573071 0.48515957
C -1.17989638 -5.34946175 -0.45270226
H -2.14742953 -4.85022836 -0.51767035
H -0.69587769 -5.40245961 -1.42866276
H -1.30001720 -6.34663525 -0.03945796
*

constrB7
C -0.01798921 -3.47556980 0.24672778
C 0.78079544 -2.90201162 1.25927719
C 1.21207801 -1.61035714 1.15420167
C 0.86570284 -0.81573834 0.01911535
C 0.05283067 -1.40740810 -0.99798788
C -0.37336137 -2.69602512 -0.88173317
H 1.04785299 -3.50040945 2.12205877
H 1.82514591 -1.17058684 1.93362944
H -0.22888339 -0.82710383 -1.86717158
H -0.98995134 -3.12389271 -1.66038609
C 1.33017187 0.47904489 -0.04486087
H 1.93104259 0.82410952 0.79543528
H 2.03186039 1.91654527 -1.38781727
H 0.58554435 1.08172640 -1.96320477
C 0.22531694 2.60666538 -0.46484240
C -1.11975583 2.38481704 -0.18369360
C 0.80230972 3.83687185 -0.16471598
C -1.88922943 3.39937395 0.36961416
H -1.57393938 1.42437952 -0.40929684
C 0.03224109 4.84931562 0.39012357
H 1.85072588 4.01257700 -0.38295145
C -1.31347597 4.63154940 0.65654473
H -2.93948785 3.22887285 0.57586839
H 0.48218032 5.81083529 0.60813867
H -1.91596690 5.42302511 1.08681737
C 1.06926407 1.49612285 -1.07986448
C -0.44732165 -4.86065949 0.41151560
C -1.16995047 -5.54971801 -0.47327395
H -0.13440922 -5.34325845 1.33189623
H -1.44422217 -6.57905058 -0.27469843
H -1.51038459 -5.13301593 -1.41387762
*

constrB8
C -0.09039702 -3.54136077 0.03315348
C 0.43961700 -3.03523686 1.23631147
C 0.88856296 -1.74404142 1.28051679
C 0.82862756 -0.90940928 0.12441129
C 0.28743722 -1.44439461 -1.08289127
C -0.16243149 -2.73309454 -1.12406822
H 0.48224715 -3.67521221 2.10802891
H 1.29711106 -1.33801131 2.19958165
H 0.23086598 -0.82853610 -1.97103728
H -0.57528436 -3.15240770 -2.03252880
C 1.30154449 0.38497794 0.22500821
H 1.68189427 0.69002353 1.19905418
H 2.30777040 1.86917896 -0.83984430
H 1.05439267 1.06660754 -1.79162319
C 0.32667852 2.53274970 -0.36826713
C -1.04497893 2.30172558 -0.42162421
C 0.80824742 3.75189144 0.09914012
C -1.93079657 3.29911554 -0.03662716
H -1.42642775 1.34935877 -0.77805777
C -0.07880041 4.74665568 0.48469999

H	1.87717858	3.93366344	0.14259613
C	-1.44794347	4.52100203	0.41680183
H	-2.99871790	3.12276885	-0.09114814
H	0.29959030	5.70075493	0.83247289
H	-2.14081119	5.29891053	0.71567269
C	1.30092687	1.44122060	-0.79926153
C	-0.55992137	-4.88246258	-0.02005144
C	-0.95626894	-6.01367528	-0.06532652
H	-1.30890381	-7.02175135	-0.10524757

*

constrB9

C	-0.02204069	-3.54774989	-0.02224001
C	0.42572197	-3.06909648	1.21565728
C	0.86448672	-1.77017044	1.30197690
C	0.86645311	-0.93087791	0.15466645
C	0.40749339	-1.44621819	-1.08840560
C	-0.03305769	-2.74174932	-1.17351122
H	0.41874288	-3.72036169	2.08027825
H	1.21325245	-1.37327958	2.24903004
H	0.40252091	-0.81985606	-1.97081828
H	-0.38697684	-3.15350170	-2.11010514
C	1.32654203	0.37507725	0.30010226
H	1.64320321	0.66874378	1.30040023
H	2.36906071	1.89746988	-0.65838263
H	1.21780357	1.07419565	-1.71839702
C	0.34142043	2.50122143	-0.33665690
C	-1.01516203	2.23509960	-0.50156939
C	0.75607447	3.72205970	0.18851328
C	-1.95310777	3.20583689	-0.17620661
H	-1.34327109	1.27995885	-0.90085208
C	-0.18380629	4.68866659	0.51351184
H	1.81311993	3.92792698	0.32094691
C	-1.53745942	4.43077422	0.33201486
H	-3.00851692	3.00563715	-0.31866798
H	0.14090525	5.64622313	0.90325207
H	-2.27101187	5.18722365	0.58529486
C	1.37553144	1.43922104	-0.70468541
C	-0.48643760	-4.90547883	-0.12032603
N	-0.86088139	-5.98895516	-0.19955131

*

constrD0

N	0.11429047	1.48815702	-1.64611933
O	1.14819591	1.42574334	-0.72190230
C	-1.11100348	1.72172898	-0.90377861
C	0.06951993	0.25416105	-2.40896434
H	-1.95427092	1.55116219	-1.59145219
C	-1.17626807	3.15733001	-0.38820152
H	1.01496555	0.14426596	-2.96344252
H	-0.05945837	-0.64180023	-1.76563380
H	-0.75755090	0.28878650	-3.13723512
H	-1.13285341	3.86678742	-1.23058230
H	-2.10901640	3.33153553	0.17139496
H	-0.32409937	3.35289947	0.27971666
C	-0.22884853	0.45637677	1.04254449
C	-1.26214706	0.70734325	0.25098760
O	0.69294298	0.23249289	1.74868170
Li	2.08962708	0.10021221	0.04800894
O	2.09684200	-1.80869702	-0.26734512
O	3.90689600	0.54335338	0.43927977
C	1.12545137	-2.73197824	0.19160811
H	0.28517782	-2.81146101	-0.52170674
H	1.57549612	-3.73106573	0.33005292
H	0.75233407	-2.37189979	1.15915752
C	2.75408904	-2.22037879	-1.45268715
H	2.02876794	-2.40235054	-2.26532797
H	3.44108322	-1.41728370	-1.75621674
H	3.33494950	-3.14310096	-1.27522672
C	4.93773756	-0.33231114	0.85135830
H	5.39450106	0.01518261	1.79551983
H	4.49186896	-1.32396199	1.00904186
H	5.72352718	-0.40564577	0.07754231
C	4.33155940	1.87583584	0.20695127
H	5.11556477	1.90521862	-0.57139709
H	3.45171511	2.43732477	-0.13743790
H	4.72725051	2.32692402	1.13463799
C	-2.55520766	0.03123344	0.53612368
C	-3.77297479	0.57994860	0.09671971
C	-2.60023457	-1.18171138	1.25214890
C	-4.98574198	-0.06038815	0.36332912
H	-3.78156463	1.52518914	-0.45121185
C	-3.81222158	-1.81322439	1.52580824
H	-1.66783714	-1.64775306	1.58716985
C	-5.01533254	-1.25796949	1.07971002
H	-5.91842368	0.38962425	0.00934771
H	-3.81503596	-2.75462043	2.08375460
H	-5.96644385	-1.75667661	1.28738340

*

constrD1

N	-2.08951012	-0.29724176	-1.11338719
O	-0.86058471	0.28359121	-0.84018857
C	-2.99022086	0.03941819	-0.02357494
C	-1.92178457	-1.72798325	-1.27535701
H	-3.89094902	-0.58828561	-0.12516090
C	-3.40855112	1.50722055	-0.09948639
H	-1.28665737	-1.91630699	-2.15547926
H	-1.43771804	-2.20248160	-0.39425670
H	-2.89966613	-2.20818447	-1.44662673
H	-3.90556666	1.71166490	-1.06205061
H	-4.10441496	1.76087179	0.71618805
H	-2.52089462	2.15226166	-0.01811901
C	-1.10228413	-0.09534273	1.64173580
C	-2.36901469	-0.28022956	1.34630842
H	-3.00522972	-0.64880002	2.15720427
O	0.03979336	0.07135010	1.90809163
Li	0.76466334	0.04916479	-0.18605587
O	2.00337409	-1.42418124	-0.18865426
O	1.93312718	1.60608960	-0.15598741
C	2.93979483	-1.48769089	0.87208077
H	2.58379457	-2.16255532	1.67082647
H	3.91915375	-1.84436281	0.50592105
H	3.05386690	-0.47426465	1.28058543
C	1.77675248	-2.67009273	-0.82205834
H	1.33983300	-3.39880760	-0.11557554
H	1.07617623	-2.50329532	-1.65101348
H	2.72139056	-3.07924848	-1.22277010
C	3.00970990	1.68571735	-1.07154132
H	3.77588832	2.39626882	-0.71313542
H	3.45520040	0.68464152	-1.15305095
H	2.65815874	2.00861840	-2.06843775
C	1.27230590	2.84285682	0.04819601
H	0.82416144	3.21066224	-0.89200386
H	0.47602016	2.67841045	0.78643118
H	1.97706532	3.60012167	0.43578025

*

constrD14

N	1.37661975	1.88439944	-1.23554456
O	2.16875104	1.38097981	-0.21633523
C	0.06237362	2.08830264	-0.66088266
C	1.35872998	0.95118175	-2.34791080
H	-0.64710600	2.23223437	-1.49036671
C	0.03650473	3.32283713	0.23769620
H	2.38450154	0.83675408	-2.73258734
H	0.98635102	-0.05376704	-2.05796438
H	0.72554163	1.34387329	-3.16025172
H	0.29017569	4.22255980	-0.34578927
H	-0.95985291	3.46367632	0.68581292
H	0.77254347	3.20746946	1.04707923
C	0.48506839	0.14730468	0.86207276

C	-0.38659318	0.83288925	0.11845840
O	1.25484413	-0.45814474	1.51876932
Li	2.98050298	-0.18926383	0.11940699
O	3.10068657	-1.82245969	-0.88033111
O	4.58199751	-0.04010814	1.14941756
C	2.02466831	-2.68923079	-1.19257241
H	1.70212222	-2.55499485	-2.24020177
H	2.31847189	-3.74296748	-1.03906945
H	1.18834537	-2.44857584	-0.52240463
C	4.23855809	-2.01272420	-1.70285881
H	3.98539714	-1.85151031	-2.76580358
H	5.00201308	-1.28238373	-1.39978521
H	4.64479410	-3.03238120	-1.57885468
C	5.41909151	-1.08103890	1.61384691
H	5.44539128	-1.10008468	2.71821501
H	5.00929686	-2.03265382	1.24776708
H	6.44857517	-0.95749535	1.23154501
C	4.97666707	1.25567714	1.56983187
H	5.98903789	1.49481922	1.19721000
H	4.24893853	1.96412705	1.14961965
H	4.97191842	1.32579032	2.67245186
C	-1.79600580	0.39982620	0.14330603
C	-2.82042651	1.21315395	-0.38570862
C	-2.16965382	-0.84735470	0.69888679
C	-4.14957284	0.80542054	-0.36617107
H	-2.58603162	2.18903556	-0.81284680
C	-3.49090342	-1.26391533	0.72804232
H	-1.40621764	-1.51435488	1.10982825
C	-4.47549137	-0.43048724	0.19016587
H	-3.76083431	-2.22985911	1.15710009
H	-4.93112068	1.44639461	-0.77662763
N	-5.87146935	-0.86321665	0.21118706
O	-6.13202643	-1.94652280	0.70609904
O	-6.71329795	-0.12235595	-0.26691785

*

constrD15

N	0.93132891	1.61965138	-1.78518362
O	2.00907812	1.28971275	-0.97685505
C	-0.17592427	1.94321820	-0.90759413
C	0.63137601	0.51146378	-2.67162043
H	-1.08038595	2.00744018	-1.53268330
C	0.04505152	3.28634856	-0.21550162
H	1.48534321	0.35425808	-3.34921575
H	0.44870446	-0.43675204	-2.12160888
H	-0.25607470	0.74455066	-3.28305076
H	0.15233940	4.08723874	-0.96489816
H	-0.80183966	3.53332744	0.44422061
H	0.96202666	3.24642846	0.39082624
C	0.58694375	0.23416318	0.75210164
C	-0.42679645	0.82377680	0.12576375
O	1.48330955	-0.28718305	1.30591994
Li	3.03026888	-0.10816537	-0.58507993
O	3.12787177	-1.93936927	-1.12973267
O	4.52771184	0.28625878	0.54385444
C	2.97845451	-2.98334190	-0.18324088
H	1.97305664	-3.43531266	-0.25517222
H	3.74091993	-3.76613994	-0.34398855
H	3.10713628	-2.55385425	0.81919638
C	3.01610495	-2.37027039	-2.47493310
H	2.01384979	-2.79021465	-2.67306590
H	3.17923778	-1.49886182	-3.12364834
H	3.77916024	-3.13630096	-2.70056288
C	5.52328705	-0.59875364	1.01811105
H	5.28373756	-0.95315442	2.03722919
H	5.56423354	-1.45808449	0.33442484
H	6.51090770	-0.10379584	1.03420860
C	4.38726015	1.46607179	1.31875846
H	5.32025862	2.05726073	1.29865635
H	3.56948451	2.04904451	0.87242327
H	4.13899367	1.21892550	2.36631851
C	-1.80043201	0.42495701	0.51402181
C	-2.90034487	1.24730607	0.21167832
C	-2.04049797	-0.78474537	1.19774661
C	-4.18159069	0.86323232	0.58618647
H	-2.79047820	2.20308758	-0.30210733
C	-3.32857777	-1.13640211	1.56675410
H	-1.23361829	-1.47959158	1.44445473
C	-4.42148845	-0.32508217	1.26730940
F	-3.55451267	-2.28577936	2.21334476
F	-5.66048054	-0.68146688	1.62085136
F	-5.22651554	1.64776379	0.29762132

*

constrD18

N	1.49110093	1.76985932	-1.47616861
O	2.41861052	1.30325750	-0.55396514
C	0.29244940	2.13560586	-0.74265010
C	1.23699255	0.74270666	-2.46926953
H	-0.50907721	2.30652037	-1.47906244
C	0.51140769	3.41823220	0.05600446
H	2.17483117	0.52705433	-3.00544390
H	0.87054441	-0.20501937	-2.02093190
H	0.49270116	1.09852853	-3.20104197
H	0.78753365	4.24463961	-0.61918545
H	-0.40128789	3.70108863	0.60417368
H	1.32554449	3.27146084	0.78143246
C	0.68625620	0.35298707	0.93725311
C	-0.18552429	0.98470374	0.16989681
O	1.46782257	-0.21335778	1.62520132
Li	2.94864214	-0.31473471	0.02697983
O	2.61263854	-2.08549034	-0.68626469
O	4.77852660	-0.35187236	0.58485039
C	1.43067828	-2.84463486	-0.50735939
H	0.67937716	-2.59606980	-1.27870275
H	1.65275085	-3.92552123	-0.55732575
H	1.02859082	-2.60826055	0.48638740
C	3.29802063	-2.37962575	-1.89012833
H	2.64099859	-2.23506425	-2.76601550
H	4.15553726	-1.69575953	-1.96710490
H	3.66626841	-3.42119261	-1.88457893
C	5.56925225	-1.48240615	0.89226078
H	6.00062919	-1.39711931	1.90602909
H	4.92000289	-2.36777924	0.84944663
H	6.39070727	-1.59836633	0.16177527
C	5.48251477	0.87828048	0.61188050
H	6.32423951	0.86176565	-0.10417712
H	4.76787294	1.66104617	0.32071477
H	5.87507687	1.08149404	1.62453016
C	-1.62091415	0.58923045	0.23294490
C	-2.64742359	1.49922108	-0.06329993
C	-2.01662393	-0.70869319	0.59902572
C	-3.99317431	1.13999523	-0.00072626
H	-2.40171832	2.52822789	-0.33981366
C	-3.35557799	-1.08159554	0.67745153
H	-1.25419697	-1.46240314	0.82391385
C	-4.38920827	-0.16655099	0.36624872
H	-3.59222960	-2.10493696	0.97157052
H	-4.74100673	1.89731127	-0.23925640
N	-5.72175400	-0.53451450	0.41549131
C	-6.74516845	0.47920059	0.26515970
C	-6.08571475	-1.81676589	0.98198309
H	-7.73349153	0.00264814	0.30786158
H	-6.70136953	1.25251814	1.05702480
H	-6.66651900	0.99081000	-0.70890948
H	-7.17600918	-1.93640914	0.93146131
H	-5.63523565	-2.65129555	0.41831198
H	-5.77774049	-1.91950267	2.04096397

*

constrD19

N	1.95147873	2.18855163	-0.69036123

O	2.69641345	1.40189127	0.17033116
C	0.62814714	2.24786511	-0.10634183
C	1.94915021	1.59706454	-2.01770339
H	-0.06786652	2.60433049	-0.88163159
C	0.58389098	3.20201042	1.08526170
H	2.98326753	1.56720641	-2.39525991
H	1.54890221	0.56188603	-2.02252766
H	1.34795047	2.21522156	-2.70377760
H	0.84093268	4.22369583	0.76300291
H	-0.41962889	3.21947555	1.53940486
H	1.30769608	2.87774026	1.84737938
C	1.06314546	-0.03191878	0.85192062
C	0.18599610	0.82892296	0.31387167
O	1.83351733	-0.78722603	1.32521852
Li	3.57410664	-0.16930764	0.02689171
O	3.63221728	-1.47506185	-1.37209864
O	5.19949276	-0.30216721	1.01260783
C	2.54009271	-2.24389601	-1.84508918
H	2.15749391	-1.83929862	-2.79882858
H	2.84262563	-3.29540966	-1.99598596
H	1.74426548	-2.20449375	-1.08909128
C	4.72177728	-1.42222580	-2.27708104
H	4.40714530	-0.99207518	-3.24445507
H	5.49934176	-0.78320260	-1.83511919
H	5.13700854	-2.43118842	-2.44869754
C	6.06700073	-1.41608043	1.10752332
H	6.15722329	-1.75360746	2.15548761
H	5.63990561	-2.22883077	0.50397085
H	7.07163397	-1.16512800	0.72199082
C	5.60945679	0.81982324	1.77785590
H	6.59935488	1.17915283	1.44372929
H	4.85850514	1.60719826	1.62329902
H	5.66216882	0.56090915	2.85045935
C	-1.21942541	0.41563918	0.24359815
C	-2.23918173	1.33643576	-0.06494917
C	-1.61096667	-0.91990732	0.48117831
C	-3.57960897	0.94855564	-0.14256018
H	-2.00625524	2.38675595	-0.23759329
C	-2.94546519	-1.31732092	0.43282104
H	-0.86299987	-1.68469537	0.70288286
C	-3.94040657	-0.38040471	0.11182757
N	-5.34325212	-0.79117141	0.04387866
O	-5.71641856	-1.64880246	0.81922398
O	-6.04906206	-0.25041444	-0.78384447
C	-4.55155595	1.98045006	-0.43073934
N	-5.26808802	2.86160470	-0.64663496
C	-3.23207841	-2.71597598	0.66334634
N	-3.37856257	-3.84906648	0.83983045

*

constrD2

N	1.00448656	1.70746080	-1.45470183
O	1.98485156	1.31817558	-0.55367377
C	-0.16404468	2.07703263	-0.67860126
C	0.73382840	0.62156126	-2.37902235
H	-1.01337550	2.16411342	-1.37451112
C	0.04238300	3.41764437	0.02227711
H	1.65057690	0.40371340	-2.94933968
H	0.41800476	-0.31017495	-1.86391965
H	-0.05463006	0.91782374	-3.09044766
H	0.23845703	4.20863847	-0.71974291
H	-0.84804476	3.69665424	0.60781108
H	0.90403324	3.35321037	0.70337861
C	0.42820311	0.41325253	1.07086570
C	-0.52546134	0.96952201	0.33551745
O	1.27821393	-0.08255597	1.72628893
Li	2.65084785	-0.24881294	0.02190570
O	2.29823508	-2.06257251	-0.55332510
O	4.51517123	-0.21286314	0.43808390
C	1.15788229	-2.83355740	-0.21878511
H	0.32474535	-2.63381058	-0.91685631
H	1.39787448	-3.91128302	-0.24419987
H	0.85606626	-2.55966926	0.80054437
C	2.87522016	-2.41504632	-1.79819379
H	2.13860677	-2.32557104	-2.61610362
H	3.71243253	-1.72864043	-1.99063052
H	3.25815459	-3.45083400	-1.77009685
C	5.35629173	-1.31297892	0.72396007
H	5.87036197	-1.17333069	1.69197665
H	4.72675760	-2.21197019	0.77537122
H	6.11459301	-1.44461130	-0.06932545
C	5.19115809	1.03014941	0.34742728
H	5.96509677	0.99635147	-0.44071193
H	4.43719567	1.78694051	0.08886992
H	5.66813367	1.28651069	1.31042902
C	-1.93006339	0.54412776	0.56069773
C	-3.00776180	1.37771567	0.21340986
C	-2.22985142	-0.71006326	1.12880798
C	-4.32892864	0.98081037	0.42478504
H	-2.82508505	2.36351455	-0.21993723
C	-3.54316818	-1.11324948	1.35432370
H	-1.41966954	-1.39826141	1.38926212
C	-4.58978019	-0.26220827	0.99702920
H	-3.75549579	-2.09005489	1.79549072
H	-5.15466013	1.64177983	0.15054299
Cl	-6.23790893	-0.76240848	1.26562530

*

constrD21

N	1.78226830	2.09952816	-0.88099062
O	2.50613527	1.42670678	0.08853507
C	0.43771657	2.20915359	-0.35290395
C	1.82878583	1.35886511	-2.13023335
H	-0.23454423	2.45480826	-1.18921014
C	0.33782452	3.30728107	0.70344085
H	2.87618772	1.29109758	-2.46361835
H	1.42891976	0.32814244	-2.03205078
H	1.25316379	1.89159310	-2.90440061
H	0.59565490	4.28343255	0.26216673
H	-0.68301311	3.36612281	1.11328070
H	1.03512511	3.09639055	1.52790439
C	0.85996983	0.08003046	0.90309063
C	-0.00544926	0.85146928	0.23121340
O	1.62174348	-0.59901375	1.49449774
Li	3.35379681	-0.16775841	0.16203434
O	3.39266755	-1.62541840	-1.08591349
O	5.00259904	-0.20554453	1.12056034
C	2.29324868	-2.43938438	-1.45483365
H	1.90138235	-2.14639911	-2.44493132
H	2.59106424	-3.50260880	-1.48634758
H	1.50535822	-2.30892377	-0.70082073
C	4.46431278	-1.67077073	-2.01174578
H	4.12933300	-1.35203719	-3.01477461
H	5.24757805	-0.98499887	-1.65918326
H	4.88085956	-2.69159608	-2.07834671
C	5.85223929	-1.31674521	1.33338744
H	5.96459216	-1.52240593	2.41301582
H	5.39448584	-2.18906890	0.84679068
H	6.85080949	-1.13721026	0.89557070
C	5.45803544	0.99639384	1.72042567
H	6.44299785	1.28682103	1.31252929
H	4.71764105	1.77423592	1.48716029
H	5.54089117	0.87806267	2.81564643
C	-1.40455578	0.40665222	0.17182868
C	-2.40884755	1.23789213	-0.36401488
C	-1.78484367	-0.87000438	0.64857851
C	-3.72028763	0.79982684	-0.42904541
H	-2.20133911	2.24415117	-0.72718137
C	-3.10094165	-1.27837206	0.59333635
H	-1.05896395	-1.57459232	1.06146449
C	-4.10282381	-0.46000125	0.04707245
N	-5.48327169	-0.90554452	-0.02134818

O	-5.93197747	-1.51219470	0.93335252
O	-6.11431130	-0.64858897	-1.02969997
F	-4.64428035	1.62105539	-0.92140183
F	-3.41677913	-2.49593842	1.02821007

*

constrD6

N	1.03747999	1.70492533	-1.51594955
O	2.01568156	1.35587997	-0.59473520
C	-0.16277086	2.03436186	-0.76919578
C	0.82760876	0.60635991	-2.44073395
H	-0.99434206	2.10330498	-1.48852381
C	-0.01295790	3.37603170	-0.05594037
H	1.76401711	0.42086195	-2.99041538
H	0.53636753	-0.33495996	-1.92832045
H	0.04369503	0.86935327	-3.17026304
H	0.18469314	4.17633374	-0.78765012
H	-0.92761978	3.62830293	0.50379716
H	0.82960882	3.33032056	0.65009167
C	0.41022891	0.39133580	1.00001810
C	-0.52940301	0.91175958	0.22658511
O	1.25067574	-0.07415344	1.69180939
Li	2.68207391	-0.18851891	0.03650416
O	2.42337440	-2.01594813	-0.54774800
O	4.52617608	-0.08991872	0.53537825
C	1.30821696	-2.83933101	-0.25767234
H	0.50740229	-2.70087185	-1.00651320
H	1.60547473	-3.90302215	-0.24232279
H	0.93470808	-2.55893243	0.73576663
C	3.07426045	-2.35007019	-1.76042546
H	2.37258567	-2.30549655	-2.61213937
H	3.88326126	-1.62316398	-1.92217856
H	3.50746888	-3.36473740	-1.70343287
C	5.38959324	-1.15788258	0.87082193
H	5.85693156	-0.99041482	1.85800636
H	4.78824917	-2.07670991	0.90690106
H	6.18535100	-1.27351462	0.11240543
C	5.16353400	1.17414440	0.46012744
H	5.97298663	1.15851748	-0.29221452
H	4.39664606	1.90163481	0.15888633
H	5.58802040	1.45664041	1.44031052
C	-1.92874123	0.42488834	0.37289477
C	-3.02251292	1.22895711	0.02865665
C	-2.20750845	-0.86788740	0.86443053
C	-4.34190422	0.77870895	0.16425759
H	-2.86115651	2.24230060	-0.34747814
C	-3.50995986	-1.32352987	1.01469044
H	-1.38267124	-1.53951345	1.12348594
C	-4.59328554	-0.50247493	0.66307969
H	-3.70934425	-2.32855204	1.39622661
H	-5.15803912	1.44532422	-0.11890590
O	-5.83520572	-1.03076951	0.83474002
C	-6.95783381	-0.23549971	0.49814574
H	-6.95415524	0.03465053	-0.57188474
H	-7.84673736	-0.84433473	0.71106964
H	-6.99692487	0.68451208	1.10659288

*

constrD8

N	1.05740617	1.89762904	-1.18679118
O	1.90729749	1.41027718	-0.20482047
C	-0.22746313	2.12641198	-0.55339793
C	0.97466134	0.94446747	-2.27843893
H	-0.97265758	2.27483174	-1.35058910
C	-0.19181790	3.37149774	0.32984978
H	1.97525015	0.82578377	-2.72340563
H	0.62223220	-0.05564307	-1.94862140
H	0.29189638	1.32067288	-3.05797540
H	0.05878335	4.25883387	-0.27389159
H	-1.16720780	3.53850894	0.81382180
H	0.57230787	3.25105411	1.11224620
C	0.20593105	0.26363173	1.03743423
C	-0.66746260	0.88520856	0.25256157
O	0.98288254	-0.28930885	1.73563914
Li	2.57256670	-0.21196475	0.21071830
O	2.34808625	-1.94429169	-0.62117298
O	4.37522825	-0.18934513	0.83926837
C	1.18629864	-2.75194435	-0.54302164
H	0.44407798	-2.45410727	-1.30566751
H	1.44416722	-3.81601310	-0.68695017
H	0.75501907	-2.62229092	0.45808793
C	3.08998930	-2.13426891	-1.81324171
H	2.46584776	-1.94225927	-2.70377915
H	3.93274650	-1.42807817	-1.80448473
H	3.48436594	-3.16475681	-1.86539475
C	5.21860898	-1.29647511	1.08975706
H	5.59434283	-1.27677655	2.12859670
H	4.62745192	-2.20981265	0.93660296
H	6.07939278	-1.29937407	0.39657657
C	5.00188636	1.07118984	1.01027408
H	5.87138058	1.16904070	0.33529611
H	4.25434791	1.83712380	0.76022012
H	5.33937549	1.20078065	2.05425390
C	-2.06774085	0.39946751	0.23618079
C	-3.12324429	1.23045487	-0.18278263
C	-2.38995404	-0.91357894	0.63860931
C	-4.43900048	0.77323491	-0.19535050
H	-2.92406876	2.25821780	-0.49350808
C	-3.70162509	-1.37148181	0.63590487
H	-1.59377043	-1.59829956	0.94686935
C	-4.74893828	-0.53255585	0.21548090
H	-3.92214358	-2.39440395	0.95236523
H	-5.24105362	1.44011007	-0.52278842
C	-6.11252428	-1.00558883	0.20371778
C	-7.25837238	-1.40172779	0.19401092
H	-8.27836546	-1.75429204	0.18563940

*

constrD9

N	0.97740399	1.81859475	-1.27403692
O	1.82262304	1.31900876	-0.29574551
C	-0.29800395	2.05292365	-0.62739796
C	0.87893851	0.86823236	-2.36756986
H	-1.05216628	2.19456530	-1.41705715
C	-0.25404533	3.30381209	0.24769546
H	1.87829866	0.73055287	-2.80982135
H	0.50762800	-0.12557475	-2.04009987
H	0.20556285	1.25896451	-3.14793213
H	-0.02070114	4.18892494	-0.36596664
H	-1.22122101	3.46833049	0.74861056
H	0.52475706	3.19185323	1.01656766
C	0.17609196	0.13638562	0.90710294
C	-0.72310037	0.81995499	0.19906411
O	0.97248414	-0.46903465	1.53419525
Li	2.58343279	-0.27958799	0.02749111
O	2.53707290	-1.94327016	-0.93692986
O	4.30057094	-0.18834100	0.85873643
C	1.41159053	-2.78578284	-1.10815676
H	0.97123503	-2.65628389	-2.11270861
H	1.69564332	-3.84492402	-0.97444420
H	0.66591581	-2.51537494	-0.34863149
C	3.56225517	-2.16910190	-1.88812926
H	3.18540575	-2.01647939	-2.91512776
H	4.37128355	-1.45169276	-1.69085805
H	3.95993934	-3.19581012	-1.79878841
C	5.12843696	-1.26183855	1.26146644
H	5.27572703	-1.25462569	2.35642916
H	4.63145713	-2.19800079	0.97166823
H	6.11431419	-1.20499252	0.76545129
C	4.80496078	1.09395693	1.19406827
H	5.78397347	1.26633929	0.71170713
H	4.07593631	1.83044622	0.82800190

H 4.91922675 1.19397009 2.28846467
C -2.13979050 0.41091891 0.30499073
C -3.17633333 1.23390238 -0.17890885
C -2.50382469 -0.82024296 0.89685574
C -4.50964708 0.84804488 -0.08143288
H -2.94821498 2.19952339 -0.63281249
C -3.83000993 -1.21123886 1.00252966
H -1.72999975 -1.49383535 1.27728854
C -4.84827101 -0.37713589 0.50970998
H -4.08392025 -2.16928154 1.46289985
H -5.29585760 1.50431653 -0.46294989
C -6.22856539 -0.77909724 0.61084970
N -7.33809548 -1.10308870 0.69266637
*

constrF14
Au 0.03570096 -2.18011027 0.16141819
C 1.32818046 0.21274011 1.39614536
C 0.15852249 -0.38555835 1.18173432
C 0.26582561 2.17908976 1.99911569
C 1.51224569 1.49417575 2.28822620
H 1.57781850 1.19434625 3.33356969
C -1.11768479 0.24344460 1.63856240
H -1.75023206 -0.45435470 2.19053944
H -1.68425754 0.57196434 0.76280712
C -0.90302975 1.52065927 2.56288083
P -0.11038293 -4.18694494 -0.96641883
C 1.07376957 -4.36145409 -2.32660874
C 0.17860303 -5.63913000 0.07824580
H 2.26483692 -0.12652765 0.96557077
C -1.72799678 -4.48427315 -1.72668887
H -1.94476477 -3.68942946 -2.44296392
H -1.73408995 -5.44848450 -2.24051170
H -2.49892974 -4.47957508 -0.95365062
H 0.91236032 -3.56445488 -3.05498006
H 0.94877552 -5.33112654 -2.81438728
H 2.09003180 -4.27934339 -1.93636715
H 1.17687420 -5.57878140 0.51600181
H 0.09545905 -6.55383290 -0.51350257
H -0.55720549 -5.65938532 0.88455257
H 2.41133610 2.02333317 1.98867911
H -1.81634970 2.10758608 2.54393056
H -0.68643533 1.16360647 3.56907002
C 0.16297775 3.26334246 1.04136918
C -1.00921225 4.03114968 0.97076763
C 1.23028648 3.55283552 0.17384051
C -1.11603476 5.06865098 0.06079916
H -1.83298930 3.84613644 1.64569167
C 1.12453854 4.57089196 -0.75244470
H 2.13615742 2.96267645 0.19838466
C -0.04784893 5.31002302 -0.78316914
H -2.00909208 5.67530116 0.00802148
H 1.93315421 4.79124043 -1.43510012
N -0.16028431 6.41540312 -1.76510422
O -1.21241354 7.01990387 -1.81510014
O 0.80624937 6.65051972 -2.46191110
*

constrF15
Au -0.04270362 -2.11463757 0.18076673
C 1.52301048 0.30433037 0.95420058
C 0.32282997 -0.26745737 1.03443820
C 0.62770822 2.37188377 1.63327019
C 1.90353743 1.66405862 1.62561195
H 2.25373582 1.47685325 2.64183795
C -0.81058535 0.43655390 1.71538037
H -1.31952929 -0.21120834 2.43200111
H -1.55187347 0.73614510 0.96844722
C -0.36209884 1.73581338 2.49866631
P -0.43233725 -4.17726678 -0.77696460
C 0.68026174 -4.56823549 -2.15299201
C -0.24844453 -5.56948187 0.36866569
H 2.34743875 -0.12367995 0.39275458
C -2.09622895 -4.37216742 -1.46797804
H -2.26163347 -3.61481459 -2.23669333
H -2.21036112 -5.36642696 -1.90639165
H -2.83654307 -4.23660615 -0.67714274
H 0.56794768 -3.81552835 -2.93576200
H 0.44749168 -5.55474133 -2.56087370
H 1.71331794 -4.55533256 -1.80014827
H 0.76568507 -5.57630557 0.77281326
H -0.44106850 -6.51330127 -0.14702961
H -0.95388993 -5.45552910 1.19400144
H 2.69064739 2.13709898 1.04739239
H -1.23585520 2.34005443 2.72323746
H 0.11817391 1.41089575 3.42143635
C 0.29862233 3.42302027 0.71241426
C -1.01842314 3.91944576 0.65437561
C 1.27716549 3.94638717 -0.15536459
C -1.33296470 4.89772719 -0.25385921
H -1.80303923 3.55456217 1.30192958
C 0.93103617 4.92520447 -1.05103623
H 2.30289551 3.60653916 -0.14335332
C -0.36985059 5.41208309 -1.11397333
F -2.57536320 5.38655599 -0.33583003
F -0.68591645 6.36094137 -1.97920363
F 1.83657930 5.44260846 -1.88860994
*

constrF19
Au -0.15806818 -2.83762875 0.17406212
C 1.16132227 -0.33312371 1.19433590
C 0.06216564 -1.09912315 1.25414109
C 0.17831124 1.50676384 1.88891055
C 1.34382697 0.76665322 2.27838708
H 1.24450951 0.31782789 3.26772623
C -0.98418799 -0.65124780 2.25016001
H -0.69111846 -1.05277427 3.22955056
H -1.96685520 -1.06747099 2.03189907
C -1.10588179 0.88580170 2.27523264
P -0.41037420 -4.78616152 -1.03805371
C -0.95812969 -4.51770444 -2.74360081
C 1.11030705 -5.75856572 -1.18990847
H 1.93724767 -0.37805954 0.44000894
C -1.62408903 -5.93375776 -0.33769420
H -2.59974484 -5.44639430 -0.28942429
H -1.69560649 -6.83127287 -0.95655722
H -1.31999190 -6.21218600 0.67310797
H -1.92097252 -4.00328818 -2.73982620
H -1.05822563 -5.47390768 -3.26256957
H -0.22928315 -3.89511534 -3.26598918
H 1.87749699 -5.15886641 -1.68337173
H 0.92288192 -6.66240654 -1.77433396
H 1.46636278 -6.03529100 -0.19572087
H 2.29334907 1.28055669 2.17560601
H -1.91888693 1.21374164 1.63051340
H -1.32506827 1.23720309 3.29475843
C 0.24832887 2.72775566 1.08359267
C -0.86500452 3.56397001 0.99968659
C 1.42524100 3.08508639 0.42280644
C -0.79907130 4.75813003 0.28913712
H -1.79101566 3.31775071 1.50178077
C 1.48160631 4.24548047 -0.33848876
H 2.30614332 2.45917918 0.46588698
C 0.37070518 5.08114843 -0.38663153
N 0.43441484 6.32830396 -1.17220234
O 0.89478028 6.24691080 -2.28973887
O 0.02060814 7.33351135 -0.63788417
C -1.97085353 5.59478160 0.24372143
N -2.93589080 6.21556542 0.22695771
C 2.71345859 4.56303049 -1.01450285
N 3.72173347 4.77674440 -1.51915500

*

constrF20
Au -0.17663057 -3.34000406 -0.03210871
C -1.68500763 -0.99955611 1.04044766
C -1.36719788 -1.65031584 -0.08004032
C -2.35379504 0.82727040 -0.08591258
C -2.78065556 0.12962181 1.10169759
H -3.77122213 -0.30844512 1.00528989
C -1.81964126 -1.14746386 -1.40938257
H -2.32879344 -1.91519970 -1.99531278
H -0.93163118 -0.84857746 -1.97382771
C -2.73833562 0.14288891 -1.31952629
P 1.15221680 -5.22536532 0.01601086
C 2.33005401 -5.25630532 1.39195630
C 0.22362391 -6.77345913 0.16870287
H -1.21054177 -1.17100205 2.00065843
C 2.16954565 -5.44214714 -1.46704327
H 2.82606042 -4.57813433 -1.58676996
H 2.77324023 -6.34883100 -1.38258450
H 1.52302142 -5.51617276 -2.34355570
H 2.98929364 -4.38864566 1.32601771
H 2.92729370 -6.17054030 1.35811730
H 1.78559240 -5.21263996 2.33721674
H -0.36017591 -6.75786867 1.09101721
H 0.90833009 -7.62471580 0.18553613
H -0.45904857 -6.87252337 -0.67744951
H -2.68710538 0.66483733 2.04024722
H -2.61509991 0.72454488 -2.22730545
H -3.77181994 -0.19192250 -1.22989411
C -1.43586053 1.96283730 -0.05927823
C -0.93724388 2.45291249 1.15287732
C -1.03018138 2.57074421 -1.24910451
C -0.04673941 3.50908609 1.18006617
H -1.22553701 2.00202364 2.09105198
C -0.14396758 3.63555746 -1.24020819
H -1.40726268 2.22849953 -2.20100486
C 0.34189896 4.09011201 -0.02294570
C 0.46572951 3.99942514 2.52242071
C 0.27018916 4.24693852 -2.56712286
N 1.29830379 5.22225078 -0.00313042
F 0.33742380 3.05395077 3.46439969
F -0.21127377 5.07872648 2.94716721
F 1.76722842 4.33352790 2.46774979
F -0.64647351 4.00640701 -3.51574988
F 1.43594266 3.74050523 -3.00148452
F 0.41472967 5.58101381 -2.48358417
O 0.89518797 6.26801080 0.45263176
O 2.40383033 5.00537896 -0.44474570
*

constrF21
Au -0.50847408 -2.38979206 0.14893487
C 0.64961296 -0.09105385 1.65484509
C -0.49674572 -0.63874742 1.24803841
C -0.32373664 1.87431355 2.11039894
C 0.72330623 1.08216147 2.70355506
H 0.47300464 0.71056743 3.69422116
C -1.80180900 0.02526629 1.52952888
H -2.51429163 -0.64388651 2.01607693
H -2.23993552 0.31848279 0.57153516
C -1.66643296 1.36140227 2.37570688
P -0.51776926 -4.34141239 -1.08130160
C -1.45754378 -4.22992258 -2.62594501
C 1.13085254 -4.89827666 -1.58483567
H 1.62842037 -0.38529989 1.29147665
C -1.23597181 -5.75635809 -0.20735284
H -2.27024558 -5.53145654 0.05993084
H -1.20897410 -6.64543826 -0.84182039
H -0.66994156 -5.94457640 0.70692293
H -2.49558147 -3.97646449 -2.40234892
H -1.42240535 -5.18295363 -3.15906243
H -1.03205363 -3.44562945 -3.25490136
H 1.60485553 -4.12632784 -2.19410032
H 1.05722332 -5.82314408 -2.16180544
H 1.74311594 -5.07065702 -0.69762945
H 1.71721305 1.51686205 2.68736218
H -2.47428683 2.02915581 2.09347919
H -1.74492762 1.09558423 3.43023447
C -0.05716970 2.95481900 1.16106279
C -1.08603169 3.83062927 0.80320375
C 1.22485293 3.11576016 0.61727755
C -0.82028236 4.85604600 -0.08024264
H -2.08448746 3.75795829 1.21007045
C 1.44609137 4.12237980 -0.29123073
H 2.04773230 2.45811409 0.86197028
C 0.43450450 5.00710265 -0.64484538
F -1.79584370 5.69849729 -0.42450624
F 2.65866723 4.27972667 -0.82587203
N 0.69253262 6.08576847 -1.60585312
O 1.11378556 5.76036937 -2.69487568
O 0.46345636 7.21759022 -1.23789719
*

constrF24
Au -0.26244120 -2.90164344 0.05980136
C 0.21633631 -0.64002080 1.94715342
C -0.65848542 -1.14714443 1.07983894
C -0.87030427 1.40086941 1.94025323
C -0.05475243 0.61716491 2.84979557
H -0.60354126 0.31289741 3.74048945
C -1.93766719 -0.43433875 0.77684876
H -2.80449969 -1.09328508 0.85656400
H -1.90236889 -0.07032920 -0.25440048
C -2.19028781 0.82112908 1.70812945
P 0.18562522 -4.85215408 -1.08756192
C 1.67882298 -4.77534619 -2.11100918
C 0.43589476 -6.29747853 -0.02351796
H 1.21230563 -1.04360075 2.09699056
C -1.12833896 -5.34930133 -2.23192317
H -1.28591925 -4.56013718 -2.96967706
H -0.85531120 -6.27567048 -2.74292995
H -2.05593592 -5.50038473 -1.67640859
H 1.57557894 -3.97469632 -2.84593494
H 1.83289573 -5.72579102 -2.62738912
H 2.54220799 -4.56135705 -1.47806162
H 1.27550706 -6.11290371 0.64935343
H 0.64335253 -7.18226456 -0.63004819
H -0.46145628 -6.46848033 0.57429465
H 0.89906129 1.05841326 3.12069241
H -2.90365380 1.48971747 1.23498205
H -2.59146036 0.45223092 2.65199445
C -0.34772876 2.49146718 1.15010567
C -1.05744236 2.95328641 0.03294382
C 0.88332285 3.09037642 1.47088607
C -0.53703407 3.96869877 -0.75033678
H -2.00865358 2.51405593 -0.23673709
C 1.37926763 4.10935756 0.68564977
H 1.43917715 2.77180638 2.34086703
C 0.68016862 4.55150870 -0.43412989
C -1.31051079 4.41832279 -1.96604860
C 2.70954140 4.74532009 1.00681568
H 1.08148529 5.35066445 -1.04585306
F -0.85396323 5.58067674 -2.45887120
F -2.61787527 4.58662030 -1.68752800
F -1.23978634 3.50933966 -2.96103480
F 3.10956617 4.48284596 2.26230263
F 2.66704780 6.08406042 0.87071136
F 3.68021297 4.30069475 0.18136086
*

constrF4
Au 0.10202840 -2.81443208 0.48172237
C 1.13808767 -0.33599534 1.78751255
C 0.03740730 -0.93030212 1.33087459
C 0.02045752 1.73010467 1.92829826
C 1.16414213 1.04814259 2.51838098
H 1.02658853 0.88619433 3.58875327
C -1.28726371 -0.23484172 1.39369737
H -2.06225843 -0.87435292 1.82088779
H -1.60696235 0.02636876 0.38060610
C -1.25409974 1.09588472 2.24867309
P 0.17188366 -4.91898720 -0.46050472
C 1.27532629 -5.05415389 -1.89185300
C 0.73486814 -6.21651615 0.67250538
H 2.13106006 -0.75994459 1.67752963
C -1.43202394 -5.50760389 -1.06479266
H -1.80836732 -4.82086004 -1.82544966
H -1.33137760 -6.50695113 -1.49488106
H -2.14323995 -5.53806151 -0.23708343
H 0.95145696 -4.35795548 -2.66791465
H 1.25943031 -6.07263078 -2.28714636
H 2.29295401 -4.79646030 -1.59204391
H 1.74078239 -5.98041204 1.02497974
H 0.74494488 -7.18421490 0.16526129
H 0.06428393 -6.26371100 1.53272249
H 2.12257222 1.52204098 2.32867793
H -2.12953558 1.69950729 2.02630899
H -1.25356033 0.80152021 3.29849188
C 0.14870634 2.77155627 0.94254167
C -0.93306325 3.08131640 0.09543492
C 1.35261208 3.48181514 0.80221576
C -0.80042195 4.04711834 -0.87977342
H -1.86986706 2.54798901 0.18255814
C 1.47685759 4.46878177 -0.15915632
H 2.18767436 3.28511367 1.46006796
C 0.40457936 4.73554249 -0.99952883
H -1.62809949 4.26901706 -1.54256227
H 2.40265659 5.02090832 -0.25370537
C 0.50971019 5.81065418 -2.05761963
F 0.08106242 5.36640506 -3.25496485
F -0.24983229 6.87966617 -1.74158000
F 1.76619665 6.25256666 -2.22348904
*

constrF9
Au -0.15269974 -1.88546366 0.13323543
C 1.04690367 0.51057820 1.45903071
C -0.10049846 -0.04319243 1.07080469
C 0.01928166 2.57067521 1.77128894
C 1.15696970 1.83528903 2.29491987
H 1.04723259 1.60134016 3.35387457
C -1.40137507 0.66615229 1.27442026
H -2.14563721 0.02672013 1.75324752
H -1.80388411 0.95427072 0.29886203
C -1.26734600 1.98569710 2.13751182
P -0.23490318 -3.94552774 -0.90183624
C 0.02886847 -3.88445313 -2.69341771
C 0.99843724 -5.12774728 -0.29806396
H 2.02067121 0.09578838 1.21997803
C -1.82071101 -4.79959104 -0.70287056
H -2.61993212 -4.18549273 -1.12231777
H -1.79754366 -5.76506165 -1.21385542
H -2.01882747 -4.95511161 0.35940847
H -0.73445880 -3.25255437 -3.15155244
H -0.02717825 -4.88922202 -3.11891230
H 1.01081209 -3.45534610 -2.90201704
H 1.99954643 -4.72355885 -0.46009192
H 0.90068223 -6.07956512 -0.82554937
H 0.85428639 -5.28854361 0.77206906
H 2.12537259 2.28557289 2.09940831
H -2.12279655 2.63121260 1.95930128
H -1.23448844 1.68355979 3.18461281
C 0.14708949 3.63065923 0.79848340
C -0.95017185 3.98164102 -0.00788113
C 1.36401098 4.31582833 0.64288579
C -0.82938514 4.97109357 -0.96220855
H -1.89463281 3.46457673 0.09414574
C 1.48436932 5.32406133 -0.29303891
H 2.21189203 4.08142378 1.27138785
C 0.38801031 5.64006064 -1.09486068
H -1.66635294 5.23337093 -1.59684009
H 2.41519089 5.86507905 -0.40706298
C 0.51306513 6.68765383 -2.07873611
N 0.61144139 7.52204444 -2.86244539
*

TS-DoeringLaFlamme(FigS6)
C 0.9693882 -1.4058616 2.0075758
C 2.4129403 -0.3962333 2.0838058
C 1.3557126 -0.3222646 1.1386433
H 0.9031352 -2.3981063 1.5207759
H 2.3001776 -0.1727507 3.1555287
O 0.2244492 1.6192240 -2.4807586
C -1.1450946 2.1153703 -2.3773486
C 1.0624449 2.5914063 -3.1787135
C -1.0557451 3.5942278 -2.7447576
H -1.7705558 1.5457132 -3.0918611
H -1.4904863 1.9346470 -1.3460692
C 0.0811902 3.5965385 -3.7801586
H 1.7350068 3.0641138 -2.4374799
H 1.6670391 2.0411992 -3.9177724
H -0.7678827 4.1856409 -1.8549886
H -2.0105846 3.9852887 -3.1397912
H 0.5421473 4.5905758 -3.9217139
H -0.2885525 3.2371835 -4.7598329
O -0.4500837 -0.9374779 -1.3684159
C -0.7553976 -1.4294013 -2.7077812
C -1.4986884 -1.3459219 -0.4486237
C -2.2164077 -1.9192235 -2.6473599
H -0.5966741 -0.5852001 -3.3989724
H -0.0405301 -2.2354409 -2.9543208
C -2.7481100 -1.3354127 -1.3214812
H -1.2702061 -2.3648947 -0.0745240
H -1.4925154 -0.6387735 0.3977406
H -2.2488928 -3.0239078 -2.6157043
H -2.7981043 -1.5837394 -3.5242144
H -3.5707236 -1.9335607 -0.8900513
H -3.1010452 -0.2959253 -1.4599816
Mg 1.0435567 0.4282146 -0.9199518
Cl 2.9591197 -0.1807128 -2.1377926
Cl 0.4420125 2.1453160 0.6858011
C 3.8511613 -0.5272427 1.6302877
H 3.9179260 -0.7209268 0.5446021
H 4.3880449 0.4179421 1.8572684
H 4.3623731 -1.3394093 2.1861344
N 0.3782803 -1.3790281 3.3023705
C -0.7499748 -0.5906459 3.5277956
O -1.3144265 0.0179148 2.6228247
C -1.2562209 -0.5416994 4.9625514
H -2.0020447 0.2685178 5.0246301
H -1.7511091 -1.4932641 5.2418472
H -0.4422936 -0.3534024 5.6878400
C 0.9446590 -2.2951479 4.2955841
H 0.1843077 -2.5984684 5.0325417
H 1.3021880 -3.2018117 3.7721093
H 1.8050886 -1.8331783 4.8221648
*

TS-HAT(FigS7)
C -0.96708232 -0.07668326 -0.03171210
H -0.27388617 1.02017562 -0.02381447
H -1.51495231 -0.04300864 -0.98566922

H	-1.63927692	0.02241139	0.83411876
C	-0.06487713	-1.18912535	0.06987816
N	0.71490919	-2.06042155	0.15427038
C	0.46830470	2.24224970	-0.01730079
H	-0.30144832	3.01814400	-0.12305827
H	0.96996076	2.21191598	0.95877732
H	1.13241445	2.12187387	-0.88319473

*
