# Supplementary material for: Recovering the causal kinetic–thermodynamic relationship in asynchronous reactions
Source: Chem Sci. 2026 Sep 22. Online ahead of print. doi: 10.1039/d6sc05228b (PMC13595108; doi:10.1039/d6sc05228b)
Supplement: Supplementary file 1 [file SC-OLF-D6SC05228B-s001.pdf]

Supplementary Information (SI) for Chemical Science.
This journal is © The Royal Society of Chemistry 2026

Supporting Information

Recovering the Causal Kinetic–Thermodynamic Relationship in Asynchronous Reactions

Eduardo Garcia-Padilla* and Guanqi Qiu*

*Eduardo Garcia-Padilla − Max-Planck-Institut für Kohlenforschung,
Kaiser-Wilhelm-Platz 1,
45470, Mülheim an der Ruhr, Germany;
orcid.org/0000-0001-9715-7992; Email: garciapadilla@kofo.mpg.de

*Guanqi Qiu − Max-Planck-Institut für Kohlenforschung,
Kaiser-Wilhelm-Platz 1,
45470, Mülheim an der Ruhr, Germany;
orcid.org/0000-0003-3818-3896; Email: gqiu@kofo.mpg.de

Table of Contents

1. Methods and Additional Calculations 3

1.1. General Methods 3

1.2. Constrained Optimisations 3

1.3. Asynchronicity without Gradient Minima and Mass-Dependent Features . 7

1.4. Reaction Energies and Activation Energies 9

2. Computed Structures and Energies 13

3. References 19

4. Coordinates of Stationary Points 20

1. Methods and Additional Calculations

1.1. General Methods

All calculations were carried out with ORCA 6.1.[1] Unless otherwise specified, calculations were run with TightSCF and, in the case of geometries, TightOpt convergence criteria. Single point calculations were performed at ωB97X-D3(BJ)/def2-TZVPPD with the tightened grid DefGrid3. The RIJCOSX approximation[2] was used and, in all cases, def2/J was used as auxiliary basis set. All stationary points were confirmed as minima or transition states through frequency analysis. Gibbs energies are reported at 298 K and 1 atm.

Geometry optimisations of the 1,2-hydride shifts on α-benzylbenzyl cations were performed with the ωB97X-D3(BJ) functional[3] and the def2-TZVP(-f) basis set.[4]

Reactions with lithium ynolates were optimised with ωB97X-D3(BJ) using ma-def2-SVP on oxygen and def2-SVP on all other atoms, a mixed basis set approach to minimise geometry error due to treatment of charge. The solvent, tetrahydrofuran, was modelled both in the optimisations and single point calculations with the implicit SMD model.[5] In some cases, where small imaginary frequencies were present, frequencies were also recalculated using a tightened grid (DefGrid3) to confirm the nature of the stationary points.

Gold(I)-mediated cyclisations were optimised with ωB97X-D3(BJ), with the def2-TZVP(-f) basis set on all non-metals and def2-TZVP (with def2-ECP) on gold.[6] The SCF convergence criteria, both in the optimisation and in the single point calculations, were increased to VeryTightSCF. The SMD model for dichloromethane was used both in the optimisations and subsequent single point calculations.

IRC calculations on the (3+2) cyclisations were carried out in ORCA 6.0 (current version as of January 2026) with the same level of theory as the aforementioned geometry optimisations using the local quadratic approximation algorithm following mass-weighted reaction coordinates.[7,8] The step size was 0.07 Bohr amu$^{1/2}$, the LQA Hessian updated with the Bofill method,[9] which is known to perform well,[10] a Bulirsch-Stoer corrector step with a threshold of 7×10^{-8} and tightened convergence criteria (tolerance of RMS gradient = 1×10^{-5}; tolerance of maximum gradient = 8×10^{-5}, strict convergence enforced). Solvation (SMD, tetrahydrofuran) was used.

IRC calculations on the gold(I)-mediated cyclisations were carried out on separately optimised TS geometries, at a ωB97X-D3(BJ)/def2-SVP + def2-TZVP[Au] level of theory with def2-TZVP(-f) on the 4 carbon atoms initially part of the alkene or alkyne, to ensure geometric proximity to the original calculation. The IRC calculation, performed in ORCA 6.1 (current version as of January 2026), used the local quadratic approximation algorithm following mass-weighted reaction coordinates. The step size was 0.19 Bohr amu$^{1/2}$, the LQA Hessian updated with the Bofill method, a Bulirsch-Stoer corrector step with a threshold of 2×10^{-6} and tightened convergence criteria (tolerance of RMS gradient = 5×10^{-6}; tolerance of maximum gradient = 7×10^{-5}, strict convergence enforced). Solvation (SMD, dichloromethane) was used.

All computational data has been included in a collection in ioChem-BD and is openly accessible at https://doi.org/10.19061/iochem-bd-6-615.[11]

1.2. Constrained Optimisations

Constrained optimisations were carried out at the same level of theory as the corresponding unconstrained geometries.

For the 1,2-hydride shifts (from protonated stilbenes), the Ph-C_{sp3}-C_{sp2} angles of well-behaved benzyl cations were found to reach around 108.5°, with some marginal variation presumably due to changes in hyperconjugation. Hence, we performed a constrained optimisation on all reactants and products of the reaction set keeping this angle fixed at 108.5°, which prevents spirocyclisation. This allows the extraction of the kinetic–thermodynamic relationship (Figure S1).

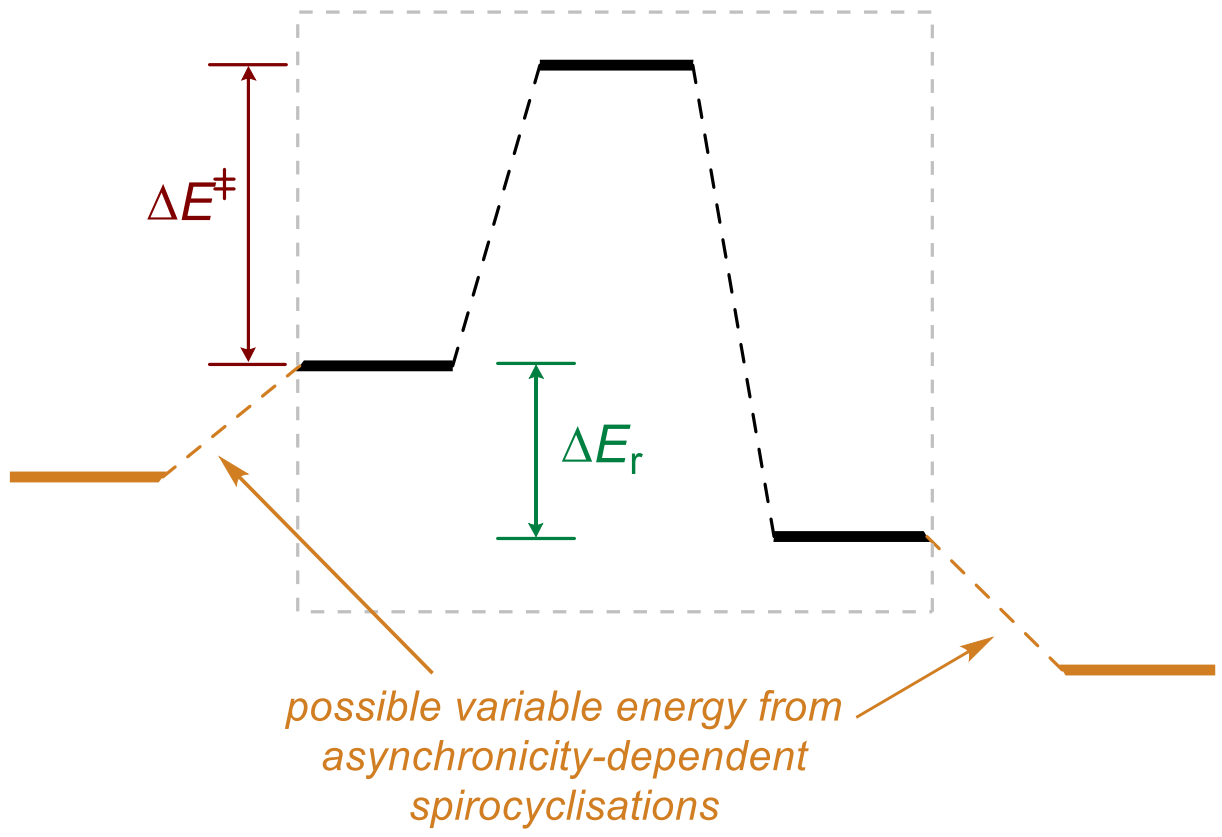

Figure S1. Conceptual representation of the main objective of decoupling: isolating the single synchronous process at the TS to study the kinetic–thermodynamic relationship; the energy changes of other processes are variable and independent from the transition state.

With the 6-*endo*-dig cyclisations, we aimed to obtain energies from the decoupled asynchronous reactions that were directly comparable to the synchronous ones (and so can be plotted together). In the manuscript, we show the better approximation for these non-stationary geometry references, which is also discussed below in more detail. The simplest way of accomplishing this is to constrain the key CCC angle (benzylic cation, CH_2, CH) to 102.1°, that of the least favoured true minimum with *meta*-CF_3 substitution. This would be analogous to the approach for the1,2-hydride shifts and indeed allows the optimisation of all 8 missing benzylic cation structures and the determination of the KTR (Figure 2). However, since the angles are seen to change significantly for all the different substitution pattern in the real minima, this is probably a worse approximation than the one used in the manuscript as it does not account for the variation in the CCC angle.

However, for all practical purposes, this alternative decoupling may have additional errors but is, however, successful in approximating the KTR by removing the secondary process (cyclopropane ring formation). The most important concept is not the evaluation of which approximation method is best, itself a worthwhile pursuit that may bear interest. Instead, it is that spurious energy responses from asynchronicity can be corrected by decoupling through intuitive constraints, and that this strategy is robust even when the constraints are not optimal.

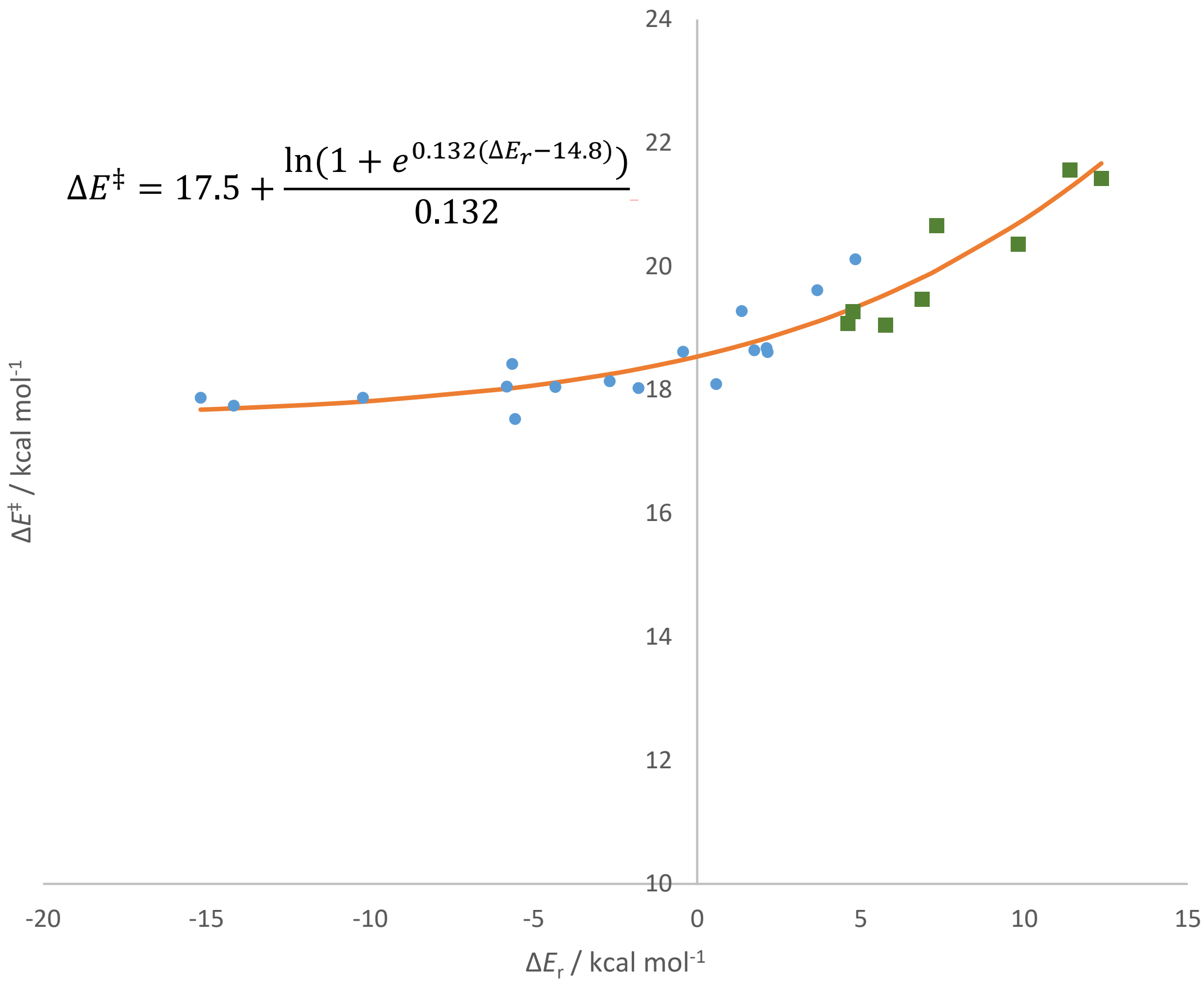

Figure S2. Plot of the kinetic-thermodynamic relationship of the initial 6-*endo*-dig cyclisation including both true minima (circles) and energies extracted from decoupling the concerted asynchronous reactions by freezing all CCC angles to 102.1° (squares). $R^2 = 0.89$.

For the IRC-based determination of the hidden intermediate (benzyl cation) angles in the eight concerted asynchronous 6-*endo*-dig cyclisations, IRC calculation were performed with the LQA algorithm at a slightly lower level of theory as detailed in the Computational Methods section. After carrying out the IRCs, plotting the key bond angle against the mass-weighted reaction coordinate showed that different reactions showed a more or less pronounced asynchronicity with large variations in the spatial extent of the hidden intermediate "plateau" (Figure S3). This is characteristic of reactions with rapidly changing degrees of asynchronicity (as is, in this case, the change between stepwise and concerted asynchronous). Note that the energies of IRC points cannot be directly plotted together with the real reactions because this would omit the minor reorganisational energy associated (from slight angle or torsional relaxations); while this is mainly thermodynamic-independent, the reaction energies would all appear higher than they should actually be.

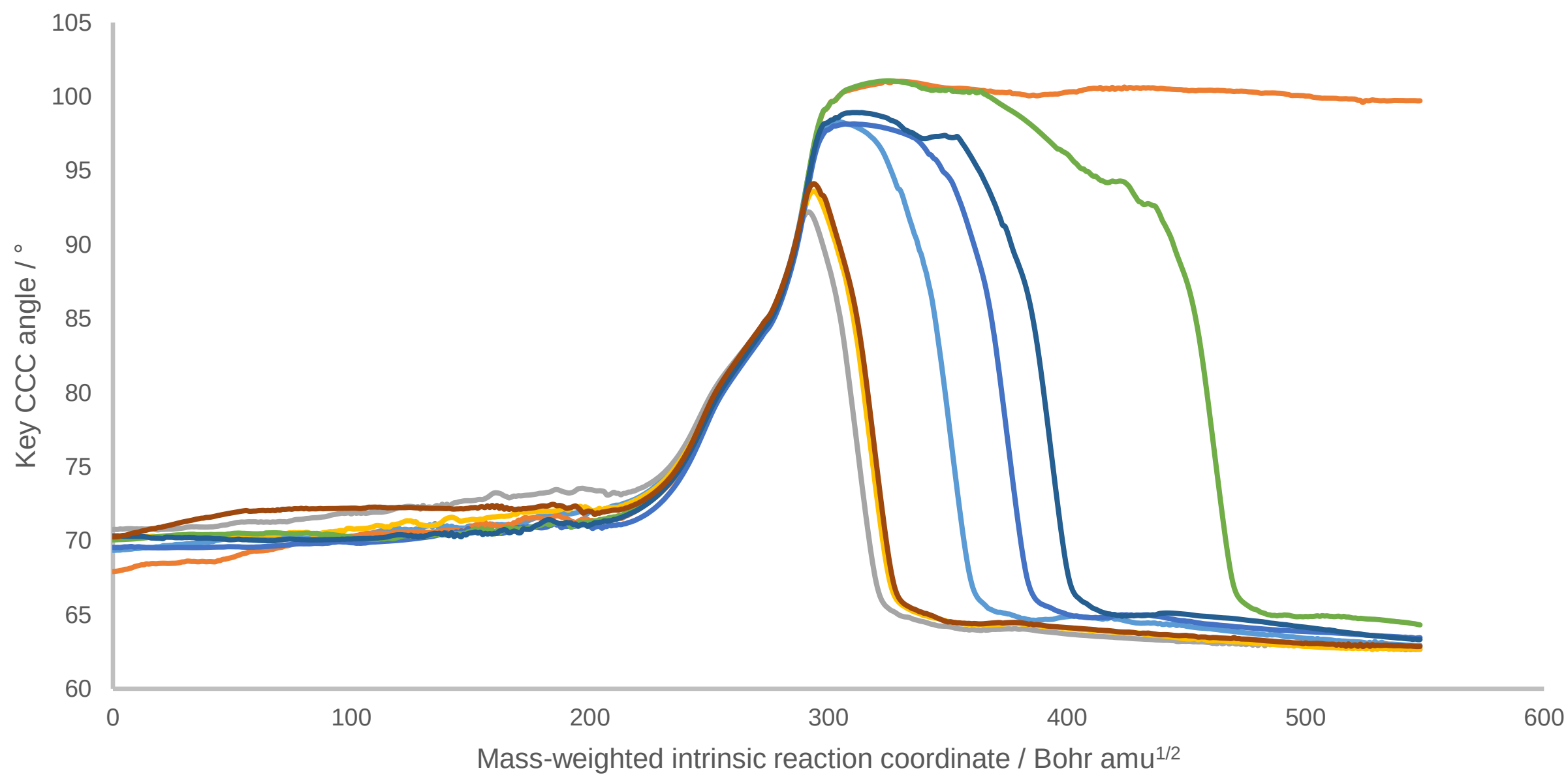

Figure S3. Change in the key bond angle over the IRC for different substitution patterns. Slowest change, orange: 3,4,5-trifluorophenyl (**TSEF15**); fastest change, grey: 3,5-dicyano-4-nitrophenyl (**TSEF19**).

The largest angles reached immediately after the transition state were selected. In order to compensate for systematic changes in the bond angles between the original triple-zeta optimisations and the IRC mixed basis set approach, we also optimised all real benzyl cation minima with the latter level of theory and compared the resulting bond angles to obtain a linear correction (HLT = 0.8602 LLT + 15.292) (Figure S4). Note that for all real minima, the discrepancies were < 1.5° in all cases.

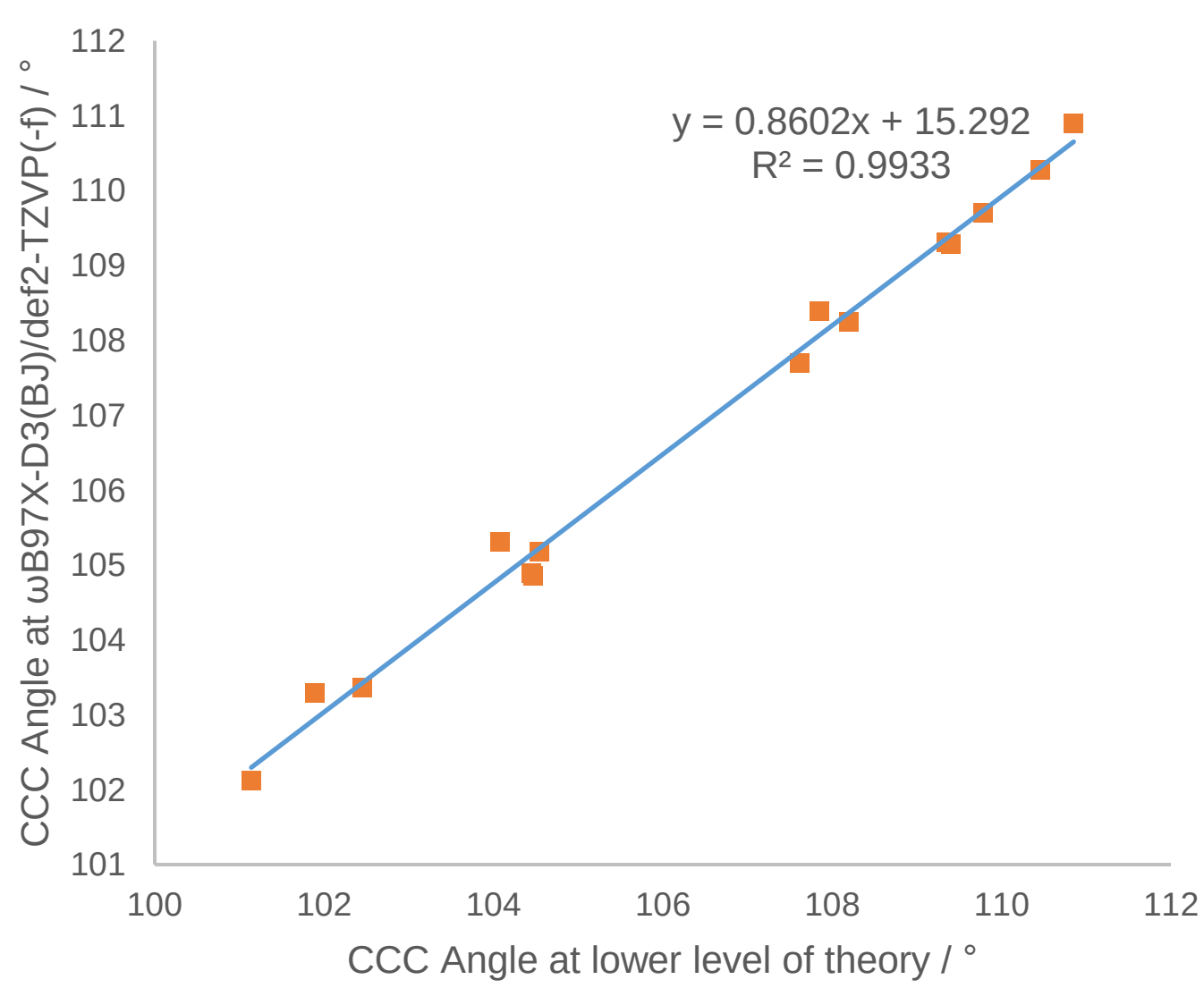

Figure S4. Linear correction used for angles extracted from the IRC.

The resulting angles used for the optimisation, obtained after the linear correction, are listed in Table S1. The angles were then constrained at the corrected value and optimised at the same level of theory as all unconstrained geometries of the 6-*endo*-dig cyclisations.

Table S1. Angles extracted from the IRC analysis and angles used for the constrained optimisations of benzyl cationic geometries.

	IRC angle / °	Corrected angle / °
**constrF4**	101.0579	102.2265
**constrF9**	98.9220	100.3891
**constrF14**	98.2957	99.8503
**constrF15**	101.0281	102.2009
**constrF19**	92.1964	94.6034
**constrF20**	94.1151	96.2540
**constrF21**	93.6110	95.8203
**constrF24**	98.1541	99.7285

For all (3+2) cycloadditions, the geometry was constrained to a linear ketene, with the CCO angle frozen at 180°. Choosing smaller angles would lead to increasingly larger reaction progression towards the final (3+2)-cyclised product. In order to evaluate the sensitivity of the observed linear energy relationship to the ketene angle, KTR plots were also obtained for 179.5°, 179.0°, 178.5° and 178.0°. The overlaid plots are shown in Figure S5.

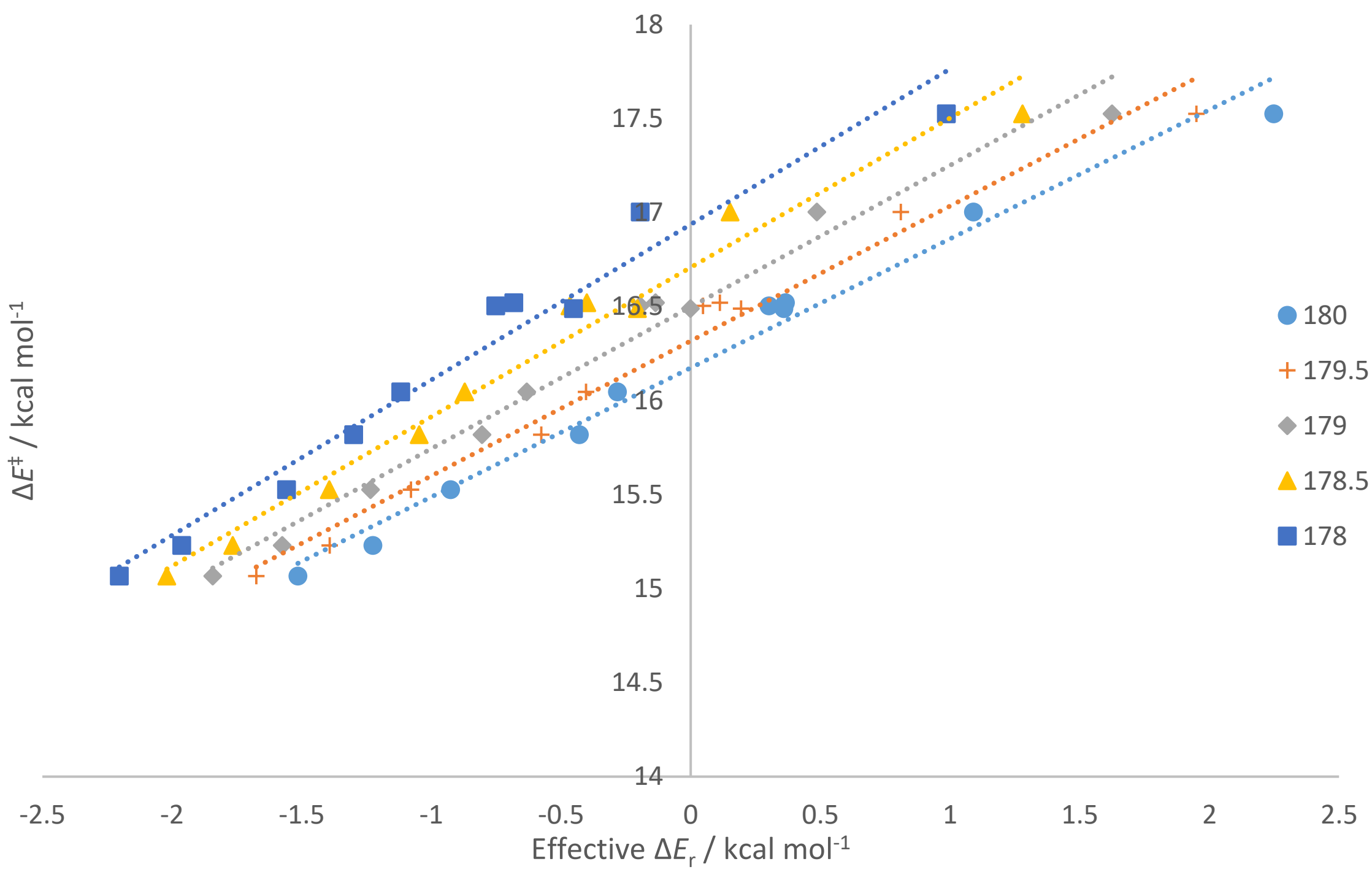

Figure S5. Linear fitting of KTR for the initial attack of the ynolate onto the nitrone, freezing the CCO angle of the ketene at one of five values.

Table S2. Variation of slopes and intercepts with ketene angle.

CCO Angle	Slope	Intercept / kcal mol^{-1}	R^2 of linear fit
180.0	0.6864	16.174	0.9826
179.5	0.7176	16.317	0.9810
179.0	0.7533	16.498	0.9795
178.5	0.7944	16.708	0.9756
178.0	0.8270	16.937	0.9650

The response of the slope is around 0.07 degree^{-1}, intercept around -0.28 kcal mol^{-1} degree^{-1} and R^2 sensitivity around 0.0028 degree^{-1}. The approximation correctly recovers the expected positive kinetic–thermodynamic response. The variation for this particular reaction is greater with geometric variation than other cases. For example, comparing with the local slopes with 6-*endo*-dig process: Figure S2: 0.323x+17.58 (R^2 = 0.86); Figure 6d: 0.383x + 17.24 (R^2 = 0.87). Both are very close in spite of the large differences in many of the used angles in the two different constrained approximations. The reason for the higher sensitivity in this (3+2) reaction is that the second process results in a rapid stabilisation upon distortion. More exothermic cyclisations (second process) lead to a decreased asynchronicity and thus to a greater change in energy with respect to geometric variation (Figure S6), which makes the KTR slope increase.

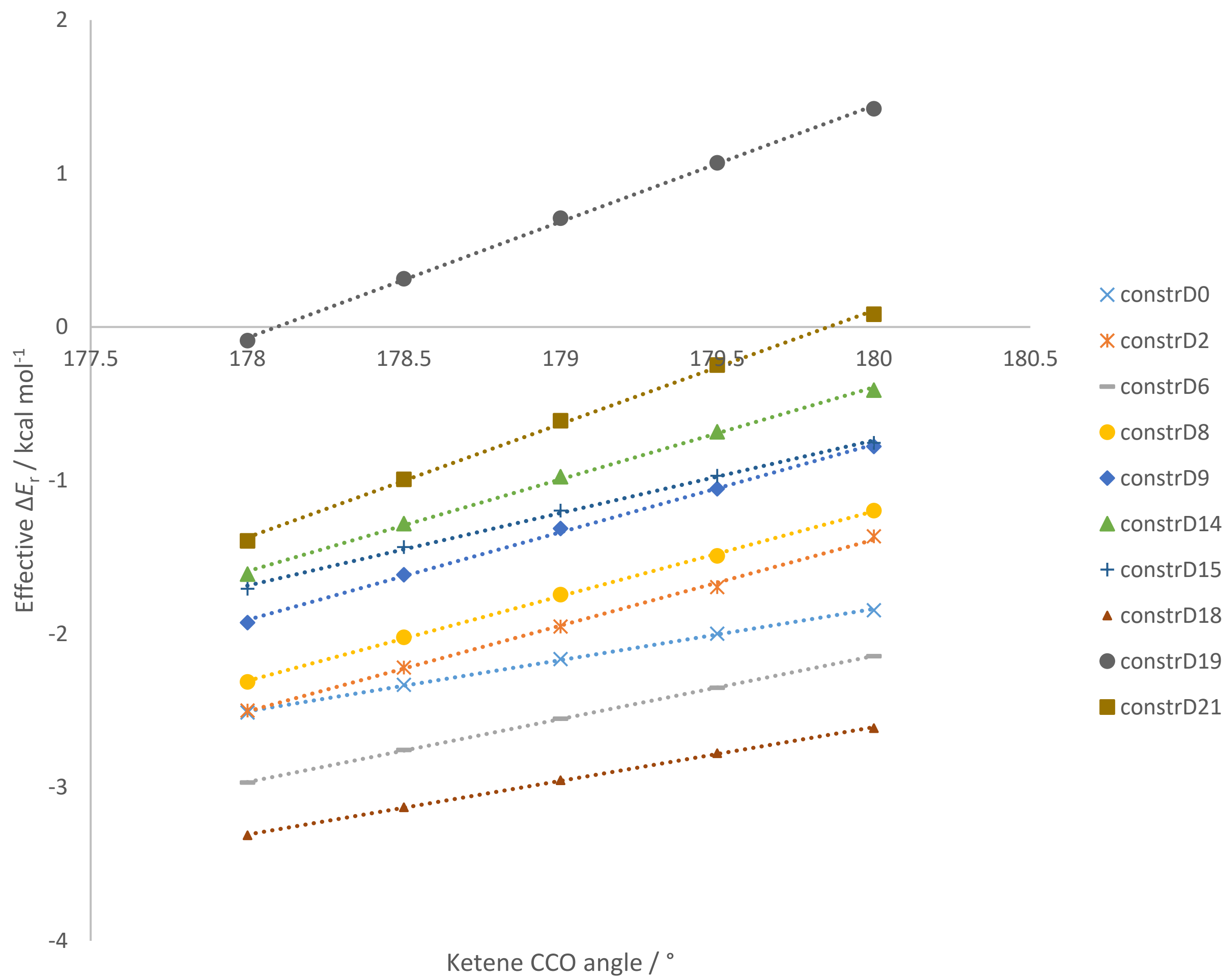

Figure S6. Variation of effective reaction energies for different frozen ketene CCO angles.

Table S3. Slopes and intercepts of effective reaction energy as a function of CCO angle as shown in Figure S6.

Pseudominimum	Slope	Intercept
**constrD0**	0.3336	-61.883
**constrD2**	0.5591	-102.030
**constrD6**	0.4110	-76.132
**constrD8**	0.5526	-100.680
**constrD9**	0.5715	-103.640
**constrD14**	0.5996	-108.320

**constrD15**	0.4734	-85.953
**constrD18**	0.3500	-65.616
**constrD19**	0.7562	-134.670
**constrD21**	0.7395	-133.000

The intercepts listed in Table S3 are closely linearly proportional to the slope (intercept = -172.69*slope – 4.863, R^2 = 0.9996). This is because of the energy-dependent degree of asynchronicity[12] and because all reactions respond in a similar way (i.e. they belong to the same reaction family). The angle-dependent equation is therefore effectively dependent on a single parameter, the slope listed in Table S3. With this slope as *s*, the energy at a given angle for a particular reaction can be linearly correlated in the following way:

$$E(\alpha) = s(\alpha - 172.69) - 4.863 \qquad \text{(Eq. S1)}$$

As the slope *s* is itself dependent on asynchronicity and the exothermicity of the second process changes the degree of asynchronicity (see Figure 5 in the manuscript), the reason for this angular dependence can be attributed to the degree to which the stabilisation cause by a Hammond-like interpolation from the final product has reached the distorted ketene at a given angle. While this is a correlational feature, it is therefore indicative of the influence of the secondary process KTR, as expected from how decreasing the angle enables the partial progression of the cyclisation. Although Equation S1 should not be linearly extrapolated because it is not analytically linear outside of this range, it would result in all reactions having an energy of -4.863 kcal mol^{-1} at 172.69°. Although the real geometric dependence is not linear, the common crossing point in Figure 5 (in the manuscript) between all IRC plots likely arises from a KT balancing phenomenon that is similar in nature.

1.3. Asynchronicity without Gradient Minima and Mass-Dependent Features

The presence of a gradient minimum is invariably linked to classical asynchronicity: there is a non-stationary region with positive curvature, a feature characteristic of minima. These cases reach a positive local maximum in curvature (d^2E/ds^2), followed by a negative local minimum, crossing the x-axis. However, there are some cases where classical asynchronous reactions do not need a gradient minimum and, yet, are separated into chemically independent reaction stages that compromise the interpolative assumption of KTR. A good example is the concerted halide abstraction—ring-opening step in the Doering-LaFlamme allene synthesis (Figure S5), calculated at TPSS-D3(BJ)/def2-SV(P), SMD (THF). There is no gradient minimum in the IRC, but clear wobbliness in the force—energy plot, corresponding to the chemically intuitive stages, shows there is some asynchronicity.

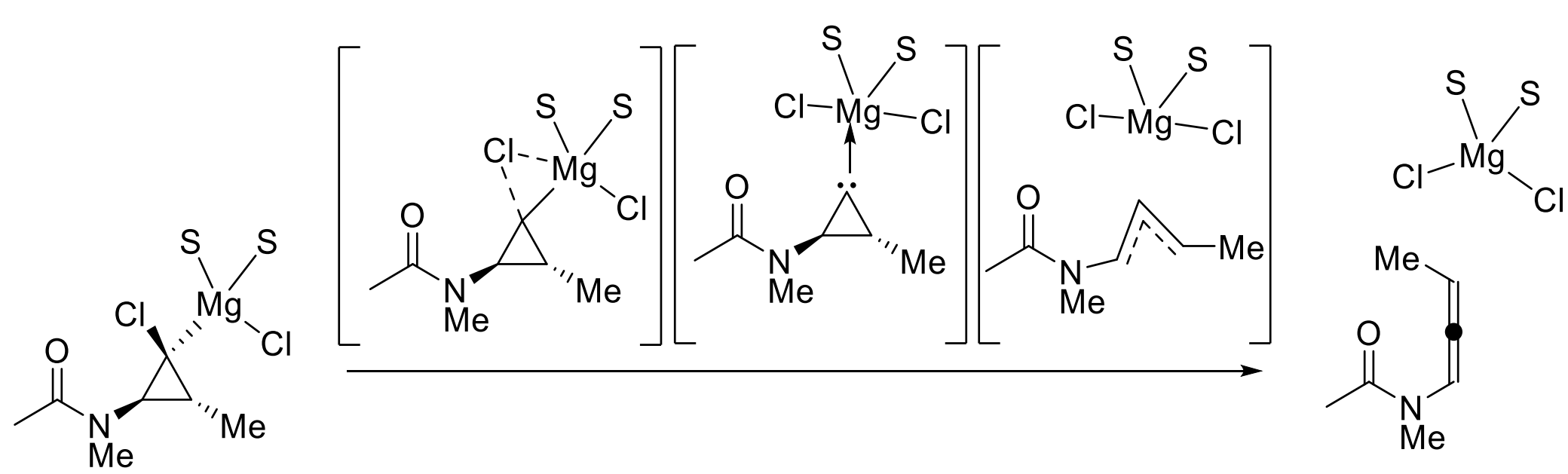

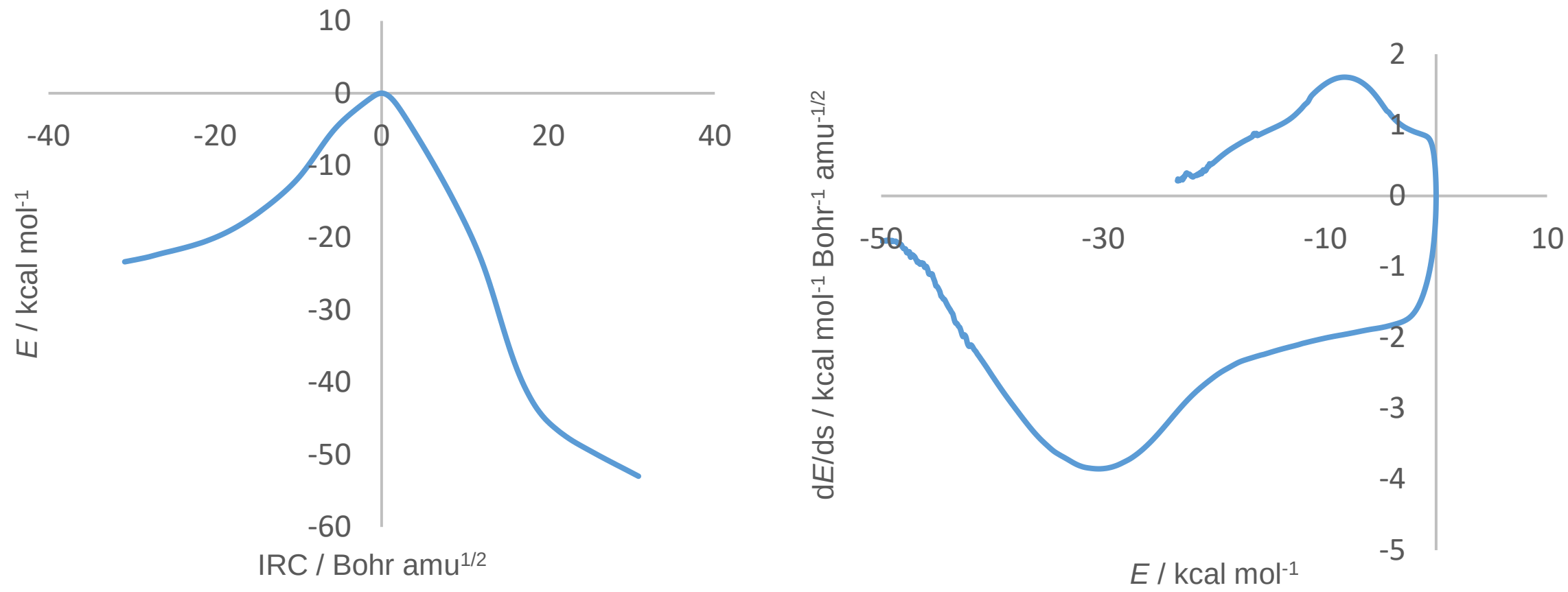

Figure S7. IRC and mass-weighted reaction coordinate force—energy plot for a Doering-LaFlamme allene synthesis ring opening step. S = THF.

When inspecting the curvature, the origin becomes clearer: two additional minima for the reaction force constant, usually attributable to a TS, appear on either side of the main TS region (the latter centred between approximately –2 and 3 Bohr amu$^{1/2}$) (see Figure 6). This is indicative of more reaction stages, which, in this particular reaction, are chemically independent and likely show different responsiveness patterns to substitution. If the curvature had increased slightly more, enough to cross the x-axis, a gradient minimum would have formed.

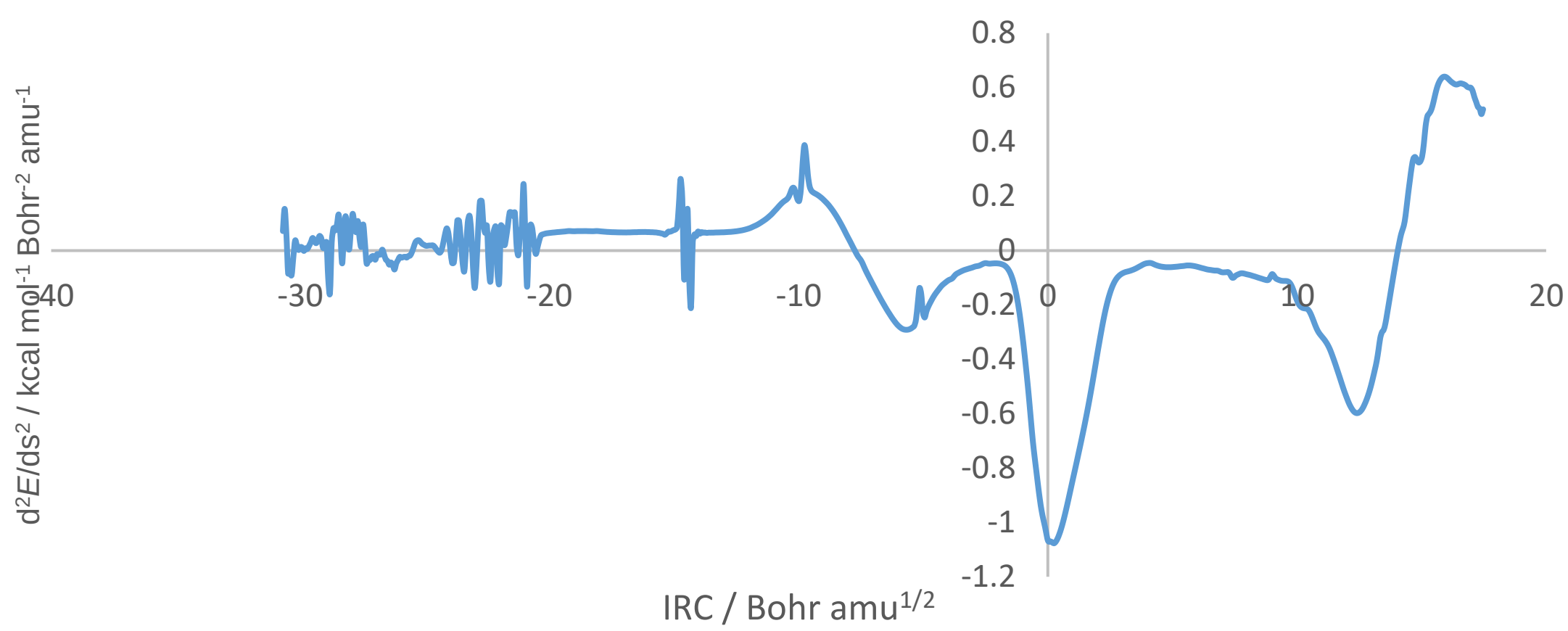

Figure S8. Evolution of reaction force constant (d^2E/ds^2) along a section of the IRC, showing additional curvature minima near –4.9 Bohr amu$^{1/2}$ and 12.4 Bohr amu$^{1/2}$.

Conversely, for reactions with strictly no gradient minima nor more than one curvature minimum, mass can become a defining feature of non-classical asynchronous processes, where the degree of asynchronicity can only be seen in an unusually rapid change in the curvature, indicating a change in the type of chemical relaxation. Most commonly, this happens before reaching a minimum, although its extent can be limited in strained systems. As detailed in the manuscript, it consists of processes minimally affected by the substitution pattern (hindered rotations, torsions, partial reorientations…) and so mostly captured in thermodynamic-independent components E_{min} and E_{eq}. In the very rare case that the reorganisation energy significantly depended on the given substitutions without breaking the validity of the reaction family, it could weaken Hammond's postulate enough to worsen the KTR correlation or could even hypothetically warrant asynchronicity analysis; however, in our experience, we have not observed this pattern in any studied systems.

The mass-related separation of the main process and the reorganisation is best visualised by plotting the IRC spatial derivative of energy against the energy. When Cartesian coordinates are used to track the relaxation of the TS, which is practical for this matter but an otherwise discouraged choice, the features practically disappear, suggesting that mass differences are key, as can be seen for an HAT reaction on acetonitrile (Figure S7). This has consequences on the kinetic–thermodynamic response, as it causes the TS to respond much less strongly to any structures that require rearrangement of polyatomic fragments, quickly reaching its "earliest" or "latest" TS character (generally high θ). Notice the features on the mass-weighted reaction coordinate plot near (–7.1, –8.8) and, slightly less visible, near (–1.1, 3.7), whereas no well-defined features can be observed for the IRC following Cartesian coordinates.

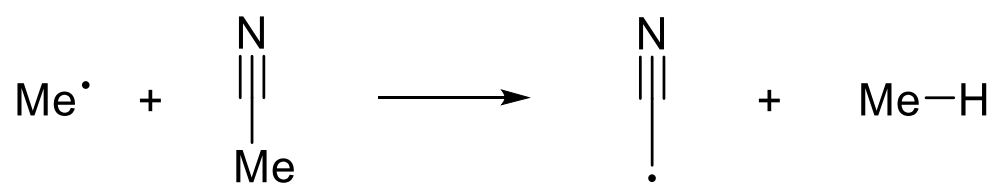

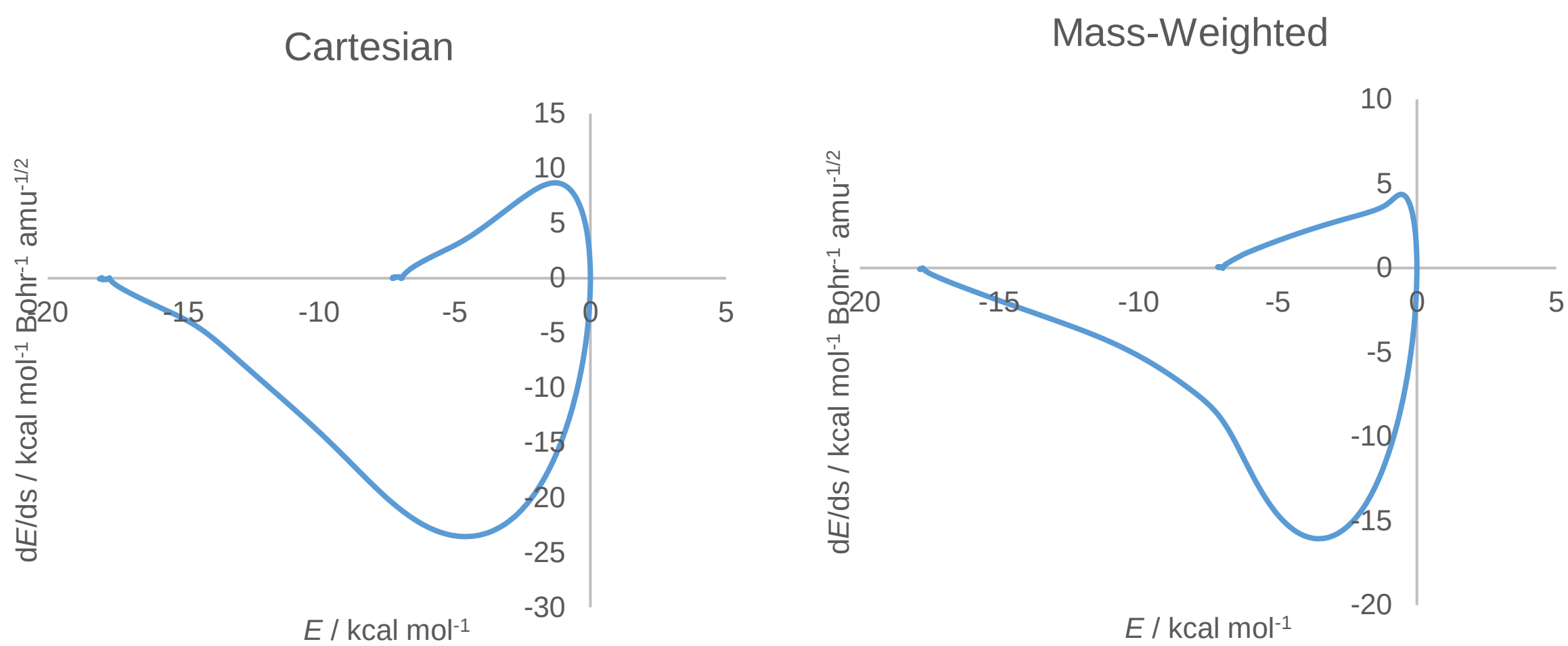

Figure S9. Comparison of the IRC reaction coordinate force—energy plot when following Cartesian coordinates and mass-weighted coordinates for an HAT reaction of a methyl radical with MeCN.

1.4. Reaction Energies and Activation Energies

The energies of all reactions shown in the figures of the manuscript, as well as the Gibbs energies where relevant, are tabulated here.

Table S4. Reaction energies and activation energies for the 1,2-hydride shifts on protonated stilbenes.

Reactant	ΔE_r / kcal mol^{-1}	$\Delta E^‡$ / kcal mol^{-1}	ΔG_r / kcal mol^{-1}	$\Delta G^‡$ / kcal mol^{-1}
**A0**	0.0	15.4	0.0	13.0
**A1**	-3.1	14.8	-3.3	12.3
**A2**	-1.2	15.5	-1.2	13.1
**A3**	2.4	16.5	2.5	14.2
**A4**	3.1	16.8	3.1	14.3
**A5**	-4.9	16.8	-5.4	13.7
**A6**	-4.9	18.3	-5.5	15.3
**A7**	-4.8	14.2	-4.7	11.9
**A8**	-1.5	15.4	-1.6	13.0
**A9**	3.7	17.3	3.7	14.9
**A10**	-0.8	15.2	-0.7	12.8
**A11**	1.4	15.8	1.3	13.4

**A12**	-5.0	15.6	-5.4	12.8
**A13**	1.4	16.2	1.4	13.8
**A14**	4.7	17.9	4.9	15.5
**A15**	2.7	17.1	2.8	14.7
**A16**	-5.4	17.5	-6.0	14.1
**A17**	-5.5	18.9	-6.2	15.8
**A18**	-5.4	25.6	-6.1	22.2
**A19**	5.9	22.0	6.4	19.4
**A20**	5.3	20.1	5.7	17.6
**A21**	5.2	19.7	5.5	17.0
**A22**	-5.4	27.3	-6.3	23.7
**A23**	-5.5	24.2	-6.3	20.5
**A24**	4.8	18.3	5.2	15.9

Table S5. Reaction energies and activation energies for the 1,2-hydride shifts on protonated stilbenes with constrained CCC angles (prevention of barrierless spirocyclisation).

Reactant	ΔE_r / kcal mol^{-1}	$\Delta E^‡$ / kcal mol^{-1}
**Constrained A0**	0.0	14.3
**Constrained A1**	-4.2	13.2
**Constrained A2**	-1.5	14.4
**Constrained A3**	3.4	15.8
**Constrained A4**	4.3	16.2
**Constrained A5**	-9.1	12.4
**Constrained A6**	-11.1	12.0
**Constrained A7**	-5.3	13.3
**Constrained A8**	-2.0	14.3
**Constrained A9**	5.1	16.8
**Constrained A10**	-1.2	14.0
**Constrained A11**	1.8	14.9
**Constrained A12**	-7.6	12.7
**Constrained A13**	2.1	15.4
**Constrained A14**	7.0	17.5
**Constrained A15**	3.7	16.6
**Constrained A16**	-10.7	12.0
**Constrained A17**	-12.0	12.3
**Constrained A18**	-20.5	10.3
**Constrained A19**	14.1	22.0
**Constrained A20**	10.6	19.9
**Constrained A21**	10.4	19.6
**Constrained A22**	-22.5	10.1
**Constrained A23**	-18.2	11.4
**Constrained A24**	6.9	17.8

Table S6. Reaction energies and activation energies for the (3+2) cyclisation of lithium ynolates with nitrones, and reaction energies when using the non-stationary ketene hidden intermediate. All energies in kcal mol^{-1}.

Reactant	ΔE_r	$\Delta E^‡$	ΔG_r	$\Delta G^‡$	ΔE_r(ketene)	ΔG_r(ketene)
**C0**	-33.2	15.5	-28.8	16.9	-0.9	1.1
**C2**	-33.5	15.8	-32.3	13.8	-0.4	-0.9
**C6**	-32.5	15.2	-29.3	14.7	-1.2	0.5
**C8**	-33.8	16.0	-31.4	14.7	-0.3	1.3
**C9**	-34.7	16.5	-31.2	16.1	0.3	2.4

**C14**	-35.3	16.5	-30.4	17.4	0.4	3.8
**C15**	-34.4	16.5	-29.9	17.1	0.4	3.1
**C18**	-32.2	15.1	-27.9	14.9	-1.5	0.6
**C19**	-36.9	17.5	-33.7	17.7	2.2	3.8
**C21**	-36.3	17.0	-32.4	16.4	1.1	2.1

In the gold(I)-mediated 6-*endo*-dig cyclisation of a 1,5-enyne, some of the initial reactions lead to a benzyl cation intermediate whereas other reactions are concerted asynchronous leading directly to the α-cyclopropylcarbene complex. For clarity, in Table S4, we show the energies corresponding to the benzyl cations, also for the concerted asynchronous reactions (where applicable, the values are indicated as constrained geometries, obtained from the IRC-derived angles). Table S5, shows the energies corresponding to the second process, with relative energies referenced to the corresponding benzyl cation.

Table S7. Reaction energies and activation energies for the initial 6-*endo*-dig cyclisation; products used for energies are benzyl cations in all cases (constrained are marked).

Reactant	ΔE_r / kcal mol^{-1}	$\Delta E^‡$ / kcal mol^{-1}	ΔG_r / kcal mol^{-1}	$\Delta G^‡$ / kcal mol^{-1}
**E0**	0.6	18.1	2.4	17.6
**E1**	-1.8	18.0	0.1	17.2
**E2**	1.7	18.6	4.2	18.6
**E3**	4.8	20.1	5.3	18.7
**E4**[a]	4.6	19.1	7.2	18.4
**E5**	-5.6	17.5	-3.2	17.0
**E6**	-5.8	18.1	-3.7	17.3
**E7**	-0.4	18.6	1.7	17.7
**E8**	2.1	18.7	4.0	18.5
**E9**[a]	5.4	19.1	6.9	18.9
**E10**	1.4	19.3	3.0	18.1
**E11**	2.2	18.6	4.0	18.1
**E12**	-2.7	18.1	-0.2	18.4
**E13**	3.7	19.6	4.5	17.9
**E14**[a]	6.9	19.5	7.8	18.0
**E15**[a]	4.8	19.3	7.2	19.0
**E16**	-4.3	18.0	-2.0	17.4
**E17**	-5.7	18.4	-3.8	17.6
**E18**	-14.2	17.7	-11.6	16.8
**E19**[a]	10.5	21.4	11.4	20.2
**E20**[a]	10.9	21.6	12.1	20.2
**E21**[a]	9.5	20.4	12.3	19.8
**E22**	-15.2	17.9	-12.4	16.9
**E23**	-10.2	17.9	-8.0	17.2
**E24**[a]	7.3	20.7	8.1	19.7

[a]These reactions are concerted asynchronous; the energies used for the product benzyl cation consequently correspond to the IRC-derived constrained geometry optimisation as explained in the previous section.

Table S8. Reaction energies using the alternative constrained products, with the CCC angle fixed at 102.1° for all product geometries.

Product	ΔE_r / kcal mol^{-1}	ΔG_r / kcal mol^{-1}	$\Delta\alpha^a$ / °	$\Delta\Delta E_r^a$ / kcal mol^{-1}
**alt-constrF4**	4.6	6.6	-0.13	0.0
**alt-constrF9**	5.8	7.7	1.71	0.4
**alt-constrF14**	6.9	8.1	2.25	0.0
**alt-constrF15**	4.8	6.9	-0.10	-0.1

**alt-constrF19**	12.4	12.7	7.50	1.9
**alt-constrF20**	11.4	12.1	5.85	0.5
**alt-constrF21**	9.8	11.4	6.28	0.4
**alt-constrF24**	7.3	8.5	2.37	0.0

[a]Difference with respect to angles and reaction energies of the IRC-derived constrained model of the hidden intermediate.

Table S9. Reaction energies and activation energies for the subsequent formation of the cyclopropane; reactants used for energies are benzyl cations in all cases (constrained are marked).

**Product**	**ΔE_r / kcal mol^{-1}**	**$\Delta E^‡$ / kcal mol^{-1}**	**ΔG_r / kcal mol^{-1}**	**$\Delta G^‡$ / kcal mol^{-1}**
**G0**	-6.0	—[a]	-6.5	—[a]
**G1**	-3.5	0.5	-3.6	0.1
**G2**	-6.6	—[a]	-7.0	—[a]
**G3**	-8.3	—[a]	-8.2	—[a]
**G4[b]**	-9.3	—[b]	-10.5	—[b]
**G5**	-0.2	2.5	-0.7	1.6
**G6**	0.6	3.1	-0.1	2.5
**G7**	-4.3	—[a]	-4.6	—[a]
**G8**	-7.0	—[a]	-6.9	—[a]
**G9[b]**	-10.1	—[b]	-9.7	—[b]
**G10**	-5.5	—[a]	-6.1	—[a]
**G11**	-7.0	—[a]	-7.1	—[a]
**G12**	-2.5	1.1	-2.6	1.0
**G13**	-7.6	—[a]	-7.8	—[a]
**G14[b]**	-11.3	—[b]	-11.5	—[b]
**G15[b]**	-9.4	—[b]	-10.0	—[b]
**G16**	-0.8	2.2	-1.5	1.7
**G17**	0.6	3.2	0.1	2.8
**G18**	9.1	9.4	8.1	8.3
**G19[b]**	-13.7	—[b]	-13.5	—[b]
**G22[b]**	-13.6	—[b]	-13.9	—[b]
**G21[b]**	-13.3	—[b]	-14.5	—[b]
**G22**	10.3	13.2	8.7	12.0
**G23**	5.0	7.8	4.3	6.7
**G24[b]**	-10.5	—[b]	-10.3	—[b]

[a]Low-lying torsional TS not found. [b]These reactions come from the barrierless concerted asynchronous process; reaction energies are referenced to the constrained benzyl cations

2. Computed Structures and Energies

Table S10. Hydride shifts on unconstrained protonated stilbenes. Low level of theory potential and free energies reported at ωB97X-D3(BJ)/def2-TZVP(-f). HLT energies reported at ωB97X-D3/def2-TZVPPD.

Code	E / Ha	G / Ha	E_{HLT} / Ha
A0	-541.48262	-541.29036	-541.50065
A1	-580.84043	-580.62294	-580.85979
A2	-1001.09365	-1000.91273	-1001.11463
A3	-878.69813	-878.50618	-878.72403
A4	-878.69777	-878.50561	-878.72366
A5	-616.75353	-616.55681	-616.77584
A6	-656.09377	-655.87045	-656.11650
A7	-618.94605	-618.72358	-618.96727
A8	-617.68088	-617.48184	-617.70153
A9	-633.77098	-633.58264	-633.79173
A10	-580.83957	-580.62181	-580.85890
A11	-616.74799	-616.55285	-616.76995
A12	-658.29598	-658.04356	-658.31922
A13	-654.86862	-654.66989	-654.88990
A14	-746.08236	-745.89106	-746.10921
A15	-839.30495	-839.14064	-839.33006
A16	-692.01853	-691.81876	-692.04459
A17	-770.69111	-770.43748	-770.71799
A18	-675.59273	-675.32988	-675.61713
A19	-930.64021	-930.45702	-930.67249
A20	-1420.49323	-1420.30072	-1420.53559
A21	-944.62094	-944.44858	-944.65236
A22	-753.09528	-752.79517	-753.12149
A23	-774.86095	-774.60698	-774.88742
A24	-1215.91349	-1215.72156	-1215.94723
B0	-541.48262	-541.29036	-541.50065
B1	-580.84547	-580.62816	-580.86481
B2	-1001.09537	-1000.91448	-1001.11650
B3	-878.69428	-878.50212	-878.72027
B4	-878.69275	-878.50059	-878.71871
B5	-616.76181	-616.56594	-616.78361
B6	-656.10220	-655.87975	-656.12434
B7	-618.95420	-618.73169	-618.97485
B8	-617.68308	-617.48421	-617.70389
B9	-633.76499	-633.57657	-633.78590
B10	-580.84085	-580.62302	-580.86015
B11	-616.74579	-616.55073	-616.76779
B12	-658.30447	-658.05271	-658.32713
B13	-654.86625	-654.66753	-654.88759
B14	-746.07433	-745.88278	-746.10168
B15	-839.30063	-839.13612	-839.32581
B16	-692.02766	-691.82884	-692.05320
B17	-770.70043	-770.44789	-770.72680
B18	-675.60188	-675.34025	-675.62570
B19	-930.63022	-930.44621	-930.66309
B20	-1420.48419	-1420.29110	-1420.52710
B21	-944.61207	-944.43920	-944.64408
B22	-753.10444	-752.80576	-753.13010
B23	-774.87024	-774.61750	-774.89614

**B24**	-1215.90531	-1215.71271	-1215.93956
**TSAB0**	-541.45734	-541.26888	-541.47612
**TSAB1**	-580.81607	-580.60262	-580.83613
**TSAB2**	-1001.06819	-1000.89109	-1001.08996
**TSAB3**	-878.67113	-878.48286	-878.69778
**TSAB4**	-878.67025	-878.48206	-878.69691
**TSAB5**	-616.72636	-616.53468	-616.74901
**TSAB6**	-656.06440	-655.84587	-656.08730
**TSAB7**	-618.92321	-618.70444	-618.94461
**TSAB8**	-617.65551	-617.46032	-617.67694
**TSAB9**	-633.74265	-633.55815	-633.76418
**TSAB10**	-580.81460	-580.60076	-580.83464
**TSAB11**	-616.72214	-616.53081	-616.74485
**TSAB12**	-658.27101	-658.02303	-658.29437
**TSAB13**	-654.84211	-654.64715	-654.86414
**TSAB14**	-746.05317	-745.86569	-746.08074
**TSAB15**	-839.27695	-839.11644	-839.30284
**TSAB16**	-691.99022	-691.79576	-692.01674
**TSAB17**	-770.66070	-770.41213	-770.68783
**TSAB18**	-675.55175	-675.29429	-675.57637
**TSAB19**	-930.60440	-930.42534	-930.63744
**TSAB20**	-1420.46037	-1420.27195	-1420.50348
**TSAB21**	-944.58879	-944.42072	-944.62099
**TSAB22**	-753.05156	-752.75721	-753.07802
**TSAB23**	-774.82219	-774.57406	-774.84890
**TSAB24**	-1215.88361	-1215.69549	-1215.91812

Table S11. Hydride shifts on constrained protonated stilbenes. Low level of theory potential and free energies reported at ωB97X-D3(BJ)/def2-TZVP(-f). HLT energies reported at ωB97X-D3/def2-TZVPPD.

Code	E / Ha	E_{HLT} / Ha
**constrA0**	-541.48098	-541.49901
**constrA1**	-580.83793	-580.85726
**constrA2**	-1001.09201	-1001.11300
**constrA3**	-878.69716	-878.72303
**constrA4**	-878.69687	-878.72276
**constrA5**	-616.74674	-616.76874
**constrA6**	-656.08423	-656.10646
**constrA7**	-618.94516	-618.96580
**constrA8**	-617.67901	-617.69968
**constrA9**	-633.77023	-633.79098
**constrA10**	-580.83766	-580.85695
**constrA11**	-616.74660	-616.76859
**constrA12**	-658.29195	-658.31459
**constrA13**	-654.86750	-654.88880
**constrA14**	-746.08175	-746.10861
**constrA15**	-839.30416	-839.32933
**constrA16**	-692.01004	-692.03598
**constrA17**	-770.68108	-770.70752
**constrA18**	-675.56888	-675.59286
**constrA19**	-930.64004	-930.67230
**constrA20**	-1420.49283	-1420.53523
**constrA21**	-944.62058	-944.65202
**constrA22**	-753.06832	-753.09416

**constrA23**	-774.84117	-774.86716
**constrA24**	-1215.91281	-1215.94653
**constrB0**	-541.48098	-541.49901
**constrB1**	-580.84458	-580.86396
**constrB2**	-1001.09417	-1001.11532
**constrB3**	-878.69171	-878.71769
**constrB4**	-878.68998	-878.71593
**constrB5**	-616.76140	-616.78321
**constrB6**	-656.10191	-656.12408
**constrB7**	-618.95353	-618.97422
**constrB8**	-617.68201	-617.70284
**constrB9**	-633.76190	-633.78279
**constrB10**	-580.83946	-580.85881
**constrB11**	-616.74379	-616.76578
**constrB12**	-658.30402	-658.32669
**constrB13**	-654.86414	-654.88548
**constrB14**	-746.07050	-746.09739
**constrB15**	-839.29820	-839.32338
**constrB16**	-692.02737	-692.05292
**constrB17**	-770.70026	-770.72662
**constrB18**	-675.60185	-675.62564
**constrB19**	-930.61775	-930.65031
**constrB20**	-1420.47581	-1420.51826
**constrB21**	-944.60425	-944.63579
**constrB22**	-753.10440	-753.13005
**constrB23**	-774.87013	-774.89609
**constrB24**	-1215.90163	-1215.93552

Table S12. Lithium ynolate (3+2) cyclisations. Low level of theory potential and free energies reported at ωB97X-D3(BJ)/def2-SVP+ma-def2-SVP[O]. HLT energies reported at ωB97X-D3(BJ)/def2-TZVPPD. Implicit solvation (tetrahydrofuran, SMD) is included in all energies.

Code / Filename	E / Ha	G / Ha	E_{HLT} / Ha
**C0**	-948.92240	-948.60113	-949.95681
**C2**	-1408.38294	-1408.06792	-1409.57685
**C6**	-1063.39916	-1063.04621	-1064.56089
**C8**	-1025.04641	-1024.71503	-1026.16301
**C9**	-1041.12824	-1040.80924	-1042.26752
**C14**	-1153.32111	-1153.00104	-1154.58604
**C15**	-1246.39287	-1246.10023	-1247.79789
**C18**	-1082.86339	-1082.47297	-1084.04137
**C19**	-1337.70249	-1337.38713	-1339.17695
**C21**	-1351.62852	-1351.32480	-1353.13618
**D0**	-948.97945	-948.65116	-950.00975
**D2**	-1408.44047	-1408.12350	-1409.63027
**D6**	-1063.45520	-1063.09720	-1064.61268
**D8**	-1025.10449	-1024.76924	-1026.21692
**D9**	-1041.18750	-1040.86289	-1042.32283
**D14**	-1153.38152	-1153.05357	-1154.64236
**D15**	-1246.45186	-1246.15209	-1247.85264
**D18**	-1082.91884	-1082.52152	-1084.09273
**D19**	-1337.76517	-1337.44467	-1339.23575
**D21**	-1351.69079	-1351.38094	-1353.19396
**TSCD0**	-948.89886	-948.57533	-949.93207

**TSCD2**	-1408.35892	-1408.04704	-1409.55165
**TSCD6**	-1063.37603	-1063.02401	-1064.53662
**TSCD8**	-1025.02201	-1024.69284	-1026.13744
**TSCD9**	-1041.10312	-1040.78469	-1042.24121
**TSCD14**	-1153.29555	-1152.97404	-1154.55972
**TSCD15**	-1246.36793	-1246.07430	-1247.77162
**TSCD18**	-1082.84075	-1082.45059	-1084.01736
**TSCD19**	-1337.67540	-1337.35971	-1339.14902
**TSCD21**	-1351.60232	-1351.29955	-1353.10908
**constrD0**	-948.92534	-948.60082	-949.95829
**constrD2**	-1408.38511	-1408.07086	-1409.57754
**constrD6**	-1063.40258	-1063.04693	-1064.56285
**constrD8**	-1025.04832	-1024.71435	-1026.16346
**constrD9**	-1041.12948	-1040.80708	-1042.26704
**constrD14**	-1153.32177	-1152.99625	-1154.58546
**constrD15**	-1246.39407	-1246.09698	-1247.79732
**constrD18**	-1082.86756	-1082.47381	-1084.04378
**constrD19**	-1337.70022	-1337.38236	-1339.17337
**constrD21**	-1351.62838	-1351.32298	-1353.13444

Table S13. LLT electronic energies (in Ha) for distorted ketene pseudominima at different angles (other than 180.0°, listed in Table S12). Energies reported at ωB97X-D3(BJ)/def2-SVP+ma-def2-SVP[O]. Implicit solvation (tetrahydrofuran, SMD) is included in all energies.

Structure	179.5°	179.0°	178.5°	178.0°
**constrD0[a]**	-948.92534	-948.92559	-948.92585	-948.92612
**constrD2[a]**	-1408.38511	-1408.38564	-1408.38605	-1408.38648
**constrD6[a]**	-1063.40258	-1063.40291	-1063.40323	-1063.40356
**constrD8[a]**	-1025.04832	-1025.04879	-1025.04920	-1025.04964
**constrD9[a]**	-1041.12948	-1041.12992	-1041.13033	-1041.13081
**constrD14[a]**	-1153.32177	-1153.32220	-1153.32267	-1153.32316
**constrD15[a]**	-1246.39407	-1246.39442	-1246.39478	-1246.39516
**constrD18[a]**	-1082.86756	-1082.86782	-1082.86810	-1082.86838
**constrD19[a]**	-1337.70022	-1337.70079	-1337.70136	-1337.70199
**constrD21[a]**	-1351.62838	-1351.62891	-1351.62949	-1351.63010

[a]Named in repository suffixed with ketene angle using a hyphen instead of a point as decimal separator, e.g. constrD0_179-5.

Table S14. HLT electronic energies (in Ha) for distorted ketene pseudominima at different angles (other than 180.0°, listed in Table S12). Energies reported at ωB97X-D3(BJ)/def2-TZVPPD. Implicit solvation (tetrahydrofuran, SMD) is included in all energies.

Structure	179.5°	179.0°	178.5°	178.0°
**constrD0[a]**	-949.95829	-949.95853	-949.95878	-949.95903
**constrD2[a]**	-1409.57754	-1409.57777	-1409.57814	-1409.57852
**constrD6[a]**	-1064.56285	-1064.56311	-1064.56340	-1064.56371
**constrD8[a]**	-1026.16346	-1026.16365	-1026.16402	-1026.16440
**constrD9[a]**	-1042.26704	-1042.26744	-1042.26783	-1042.26826
**constrD14[a]**	-1154.58546	-1154.58586	-1154.58626	-1154.58668
**constrD15[a]**	-1247.79732	-1247.79758	-1247.79790	-1247.79822
**constrD18[a]**	-1084.04378	-1084.04404	-1084.04430	-1084.04458
**constrD19[a]**	-1339.17337	-1339.17384	-1339.17436	-1339.17491
**constrD21[a]**	-1353.13444	-1353.13488	-1353.13540	-1353.13593

[a]Named in repository suffixed with ketene angle using a hyphen instead of a point as decimal separator, e.g. constrD0_179-5.

Table S15. Gold(I)-catalysed 6-*endo*-dig cyclisations. Low level of theory potential and free energies reported at ωB97X-D3(BJ)/def2-TZVP(-f). HLT energies reported at ωB97X-D3(BJ)/def2-TZVPPD. Implicit solvation (dichloromethane, SMD) is included in all energies. Geometries labelled “alt-constr” correspond to those with the angle constrained to a constant 102.1° (instead of the preferable variable constrain extracted from the IRC). Note that transition states referred to as **TSEG** in the manuscript are listed here as **TSEF** as no disambiguation is needed.

Code	E / Ha	G / Ha	E_{HLT} / Ha
**E0**	-1061.80424	-1061.53235	-1061.82906
**E1**	-1101.16039	-1100.86297	-1101.18649
**E2**	-1521.42043	-1521.16028	-1521.44828
**E3**	-1399.02927	-1398.75595	-1399.06203
**E4**	-1399.02715	-1398.75500	-1399.06001
**E5**	-1137.07270	-1136.79773	-1137.10115
**E6**	-1176.40687	-1176.10468	-1176.43577
**E7**	-1139.27089	-1138.96849	-1139.29820
**E8**	-1138.00721	-1137.72867	-1138.03464
**E9**	-1154.10764	-1153.83951	-1154.13536
**E10**	-1101.16211	-1100.86383	-1101.18818
**E11**	-1137.07352	-1136.79894	-1137.10197
**E12**	-1178.61772	-1178.28744	-1178.64705
**E13**	-1175.20227	-1174.92179	-1175.23072
**E14**	-1266.41870	-1266.14623	-1266.45265
**E15**	-1359.63511	-1359.39136	-1359.66731
**E16**	-1212.33946	-1212.06173	-1212.37152
**E17**	-1291.00437	-1290.67185	-1291.03733
**E18**	-1195.88929	-1195.54831	-1195.91979
**E19**	-1451.00003	-1450.73469	-1451.03967
**E20**	-1940.83968	-1940.56581	-1940.88928
**E21**	-1464.96203	-1464.70934	-1465.00070
**E22**	-1273.38992	-1273.01262	-1273.42251
**E23**	-1295.16555	-1294.83323	-1295.19808
**E24**	-1736.24939	-1735.97636	-1736.29019
**F0**	-1061.80447	-1061.52967	-1061.82814
**F1**	-1101.16439	-1100.86398	-1101.18934
**F2**	-1521.41873	-1521.15474	-1521.44550
**F3**	-1399.02254	-1398.74841	-1399.05432
**constrF4**	-1399.02089	-1398.74465	-1399.05268
**F5**	-1137.08272	-1136.80392	-1137.11003
**F6**	-1176.41726	-1176.11169	-1176.44504
**F7**	-1139.27269	-1138.96696	-1139.29889
**F8**	-1138.00489	-1137.72342	-1138.03126
**constrF9**	-1154.10013	-1153.82956	-1154.12676
**F10**	-1101.16105	-1100.86024	-1101.18600
**F11**	-1137.07100	-1136.79342	-1137.09854
**F12**	-1178.62316	-1178.28892	-1178.65131
**F13**	-1175.19757	-1174.91586	-1175.22486
**constrF14**	-1266.40885	-1266.13482	-1266.44171
**constrF15**	-1359.62839	-1359.38091	-1359.65963
**F16**	-1212.34725	-1212.06576	-1212.37844
**F17**	-1291.01427	-1290.67881	-1291.04634
**F18**	-1195.91314	-1195.56805	-1195.94237
**constrF19**	-1450.98433	-1450.71750	-1451.02293

**constrF20**	-1940.82322	-1940.54747	-1940.87190
**constrF21**	-1464.94794	-1464.69065	-1464.98564
**F22**	-1273.41558	-1273.03385	-1273.44671
**F23**	-1295.18291	-1294.84709	-1295.21436
**constrF24**	-1736.23863	-1735.96430	-1736.27855
**G0**	-1061.81351	-1061.53948	-1061.83771
**G1**	-1101.16945	-1100.86924	-1101.19490
**G2**	-1521.42883	-1521.16537	-1521.45607
**G3**	-1399.03531	-1398.76102	-1399.06757
**G4**	-1399.03526	-1398.76092	-1399.06752
**G5**	-1137.08223	-1136.80431	-1137.11029
**G6**	-1176.41569	-1176.11118	-1176.44414
**G7**	-1139.27902	-1138.97371	-1139.30575
**G8**	-1138.01558	-1137.73399	-1138.04237
**G9**	-1154.11584	-1153.84453	-1154.14291
**G10**	-1101.16926	-1100.86951	-1101.19473
**G11**	-1137.08167	-1136.80416	-1137.10975
**G12**	-1178.62650	-1178.29248	-1178.65525
**G13**	-1175.20922	-1174.92781	-1175.23703
**G14**	-1266.42632	-1266.15262	-1266.45970
**G15**	-1359.64280	-1359.39628	-1359.67459
**G16**	-1212.34760	-1212.06725	-1212.37967
**G17**	-1291.01265	-1290.67790	-1291.04541
**G18**	-1195.89802	-1195.55449	-1195.92788
**G19**	-1451.00561	-1450.73847	-1451.04469
**G20**	-1940.84442	-1940.56911	-1940.89360
**G21**	-1464.96866	-1464.71324	-1465.00689
**G22**	-1273.39841	-1273.01914	-1273.43033
**G23**	-1295.17451	-1294.83977	-1295.20642
**G24**	-1736.25498	-1735.98024	-1736.29531
**TSEF0**	-1061.77627	-1061.50509	-1061.80023
**TSEF1**	-1101.13246	-1100.83634	-1101.15776
**TSEF2**	-1521.39157	-1521.13142	-1521.41857
**TSEF3**	-1398.99791	-1398.72684	-1399.02998
**TSEF4**	-1398.99753	-1398.72641	-1399.02962
**TSEF5**	-1137.04534	-1136.77125	-1137.07321
**TSEF6**	-1176.37880	-1176.07787	-1176.40701
**TSEF7**	-1139.24205	-1138.94108	-1139.26853
**TSEF8**	-1137.97831	-1137.70013	-1138.00487
**TSEF9**	-1154.07817	-1153.81031	-1154.10500
**TSEF10**	-1101.13222	-1100.83577	-1101.15746
**TSEF11**	-1137.04446	-1136.77068	-1137.07231
**TSEF12**	-1178.58966	-1178.25888	-1178.61815
**TSEF13**	-1175.17189	-1174.89421	-1175.19946
**TSEF14**	-1266.38849	-1266.11840	-1266.42162
**TSEF15**	-1359.60509	-1359.36179	-1359.63661
**TSEF16**	-1212.31093	-1212.03421	-1212.34276
**TSEF17**	-1290.97542	-1290.64421	-1291.00798
**TSEF18**	-1195.86181	-1195.52236	-1195.89151
**TSEF19**	-1450.96676	-1450.70334	-1451.00552
**TSEF20**	-1940.80599	-1940.53427	-1940.85491
**TSEF21**	-1464.93034	-1464.67859	-1464.96825
**TSEF22**	-1273.36221	-1272.98651	-1273.39403
**TSEF23**	-1295.13785	-1294.80659	-1295.16960
**TSEF24**	-1736.21717	-1735.94563	-1736.25726

**TSFG1**	-1101.16361	-1100.86370	-1101.18862
**TSFG5**	-1137.07844	-1136.80113	-1137.10599
**TSFG6**	-1176.41218	-1176.10758	-1176.44015
**TSFG12**	-1178.62118	-1178.28716	-1178.64952
**TSFG16**	-1212.34340	-1212.06268	-1212.37488
**TSFG17**	-1291.00894	-1290.67413	-1291.04128
**TSFG18**	-1195.89764	-1195.55436	-1195.92735
**TSFG22**	-1273.39365	-1273.01397	-1273.42562
**TSFG23**	-1295.17013	-1294.83605	-1295.20197
**alt-constrF4**	-1399.02086	-1398.74557	-1399.05267
**alt-constrF9**	-1154.09952	-1153.82830	-1154.12617
**alt-constrF14**	-1266.40886	-1266.13446	-1266.44170
**alt-constrF15**	-1359.62848	-1359.38126	-1359.65973
**alt-constrF19**	-1450.98137	-1450.71554	-1451.01997
**alt-constrF20**	-1940.82245	-1940.54750	-1940.87113
**alt-constrF21**	-1464.94737	-1464.69221	-1464.98507
**alt-constrF24**	-1736.23859	-1735.96373	-1736.27853

3. REFERENCES

[1] F. Neese, "Software update: the ORCA program system, version 6.0," *WIREs Comput. Molec. Sci.* 15 (2025): e70019.

[2] F. Neese, "An improvement of the resolution of the identity approximation for the formation of the Coulomb matrix," *J. Comp. Chem.* 24 (2003): 1740–1747.

[3] Y.-S. Lin, G.-D. Li, S.-P. Mao, and J.-D. Chai, "Long-Range Corrected Hybrid Density Functionals with Improved Dispersion Corrections." *J. Chem. Theory Comput.* 9 (2013): 263–272.

[4] F. Weigend and R. Ahrlichs, "Balanced basis sets of split valence, triple zeta valence and quadruple zeta valence quality for H to Rn: Design and assessment of accuracy," *Phys. Chem. Chem. Phys.* 7 (2005): 3297–3305.

[5] A. V. Marenich, C. J. Cramer, and D. G. Truhlar, "Universal Solvation Model Based on Solute Electron Density and on a Continuum Model of the Solvent Defined by the Bulk Dielectric Constant and Atomic Surface Tensions," *J. Phys. Chem. B* 113 (2009): 6378–6396.

[6] D. Andrae, U. Haeussermann, M. Dolg, H. Stoll, and H. Preuss, "Energy-adjusted ab initio pseudopotentials for the second and third row transition elements," *Theor. Chim. Acta*, 77 (1990): 123-141.

[7] M. Page and J. W. McIver Jr., "On evaluating the reaction path Hamiltonian," *J. Chem. Phys.* 88 (1988): 922–935.

[8] M. Page, C. Doubleday, and J. W. McIver Jr., "Following steepest descent reaction paths. The use of higher energy derivatives with ab initio electronic structure methods," *J. Chem. Phys.* 93 (1990): 5634–5642.

[9] J. M. Bofill, *J. Comput. Chem.* 19 (1998): 349–362.

[10] H. P. Hratchian and H. B. Schlegel, "Using Hessian Updating To Increase the Efficiency of a Hessian Based Predictor-Corrector Reaction Path Following Method," *J. Chem. Theory Comput.* 1 (2005): 61–69.

[11] M. Álvarez-Moreno, C. de Graaf, N. Lopez, F. Maseras, J. M. Poblet, and C. Bo, "Managing the Computational Chemistry Big Data Problem: The ioChem-BD Platform," *J. Chem. Inf. Model.* 55 (2015): 95−103.

[12] C. Barrales-Martínez and P. Jaque, "A deeper analysis of the role of synchronicity on the Bell–Evans–Polanyi plot in multibond chemical reactions: a path-dependent reaction force constant," *Phys. Chem. Chem. Phys.* (2022) 24: 14772–14779.

4. Coordinates of Stationary Points

A0
C -1.03260921 -3.66921585 0.54092896
C -0.27181064 -3.73837875 -0.62157022
C 0.63253976 -2.73079701 -0.91879540
C 0.78970199 -1.65945280 -0.03998068
C 0.00624523 -1.57771317 1.10959821
C -0.89277920 -2.59077786 1.40691413
H -1.73777008 -4.45910354 0.77213180
H -0.37899876 -4.58214363 -1.29285863
H 1.23765549 -2.79145267 -1.81773373
H 0.10929619 -0.73209715 1.78201512
H -1.48580658 -2.53845122 2.31224719
C 1.79532268 -0.54187838 -0.36102416
H 2.27270059 -0.15474826 0.53487707
H 2.54556242 -0.90886557 -1.06129089
H 0.52672223 0.08001702 -1.99847185
C 0.27779459 1.53375378 -0.47697762
C -0.72313799 2.18722208 -1.24189327
C 0.66909409 2.08233829 0.77259601
C -1.31215291 3.34021231 -0.77508083
H -1.01783517 1.76445278 -2.19638585
C 0.07638657 3.23378946 1.22901737
H 1.44036412 1.60181460 1.36087591
C -0.91121398 3.85703525 0.45679375
H -2.07648030 3.84259432 -1.35454148
H 0.37131309 3.66419833 2.17793305
H -1.37291804 4.76706137 0.82517568
C 0.86090666 0.38134968 -1.00743884
*

A1
C -0.55823592 -3.33383015 0.29422651
C 0.29626541 -3.26275953 -0.80922753
C 1.13950669 -2.18129557 -0.98770005
C 1.15920912 -1.14890800 -0.04697661
C 0.28293270 -1.19152842 1.03755567
C -0.55163235 -2.28095528 1.21184727
H 0.30120141 -4.06993702 -1.53349982
H 1.80624523 -2.15016085 -1.84379190
H 0.26233702 -0.37763493 1.75487135
H -1.21419980 -2.31616579 2.06957760
C 2.10720649 0.04879535 -0.23138242
H 2.43702701 0.46066564 0.71772333
H 2.95809648 -0.23241042 -0.84940002
H 0.94913741 0.55176178 -1.98513958
C 0.31803771 1.88852189 -0.45878011
C -0.69508529 2.40325045 -1.30260244
C 0.50645864 2.45297372 0.82655816
C -1.49617537 3.43980875 -0.87545796
H -0.83432796 1.96991216 -2.28723184
C -0.29579051 3.48834607 1.24374353
H 1.28797974 2.08061398 1.47676146
C -1.29490682 3.97644631 0.39490633
H -2.27273104 3.83568200 -1.51792373
H -0.15455472 3.93055827 2.22219878
H -1.92288155 4.79407032 0.73227794
C 1.12405908 0.85085791 -0.95403104
C -1.44730778 -4.52830842 0.49864977
H -0.90621822 -5.30475070 1.04732306
H -2.33464795 -4.26866905 1.07724304
H -1.76192510 -4.95464267 -0.45491258
*

A10
C -0.98029800 -3.12337196 -1.09831636
C 0.13650133 -3.37041152 -0.29388995
C 0.49226692 -2.41139775 0.64498592
C -0.26092739 -1.24541006 0.79574058
C -1.35211655 -1.00173116 -0.03227606
C -1.71888256 -1.95653447 -0.97046194
H 1.34819701 -2.58684964 1.29022598
H -1.92159997 -0.08322673 0.06208030
H -2.58009429 -1.78756851 -1.60610159
C 0.14720547 -0.20151462 1.84964297
H -0.71472736 0.28120596 2.30129723
H 0.76824429 -0.66777316 2.61377825
H 1.89050683 0.23273677 0.64868311
C 0.55985263 1.85920708 0.36232189
C 1.42896644 2.42078876 -0.60687410
C -0.62555917 2.55177689 0.71951499
C 1.12348801 3.62512403 -1.20006284
H 2.33474757 1.88810969 -0.87600443
C -0.92122351 3.75343452 0.12314778
H -1.29216451 2.14122782 1.46721398
C -0.04928588 4.28471552 -0.83415564
H 1.78382951 4.05768387 -1.94117868
H -1.82082023 4.29272151 0.39271247
H -0.29097989 5.23464850 -1.29904631
C 0.93006668 0.64545007 0.95098285
H -1.27141777 -3.86145462 -1.83858681
C 0.91306239 -4.65224819 -0.43714385
H 1.82411842 -4.63655097 0.16210098
H 1.18922531 -4.82307638 -1.47977657
H 0.30827459 -5.50253495 -0.11262813
*

A11
C -0.97043118 -3.25390159 -1.06559502
C 0.09612640 -3.46879452 -0.19139423
C 0.44412517 -2.48779248 0.73158089
C -0.28841256 -1.30795024 0.78517063
C -1.33566650 -1.07496148 -0.10345095
C -1.67922571 -2.06352561 -1.01547673
H 1.26307307 -2.68042173 1.41567510
H -1.88971009 -0.14345979 -0.07354690
H -2.50577308 -1.90574539 -1.69819342
C 0.09697501 -0.22204604 1.80076989
H -0.77685546 0.26231760 2.22785200
H 0.70926824 -0.65823810 2.59074730
H 1.85959772 0.21209521 0.62600855
C 0.55307406 1.85635608 0.35162800
C 1.45287300 2.43515137 -0.58189224
C -0.64192057 2.54663073 0.68817668
C 1.16918605 3.65235751 -1.15728079
H 2.36396681 1.90384232 -0.83532332
C -0.91528382 3.76098153 0.10900238
H -1.33258377 2.12135090 1.40505290
C -0.01216098 4.30834563 -0.81047210
H 1.85157943 4.09855587 -1.86973499
H -1.82085628 4.29837959 0.36163568
H -0.23676580 5.26902895 -1.26187362
C 0.89989155 0.63207430 0.92180142
H -1.24363645 -4.02029372 -1.78405250
O 0.83023146 -4.60542689 -0.18685207
H 0.48949322 -5.22915344 -0.83756690
*

A12
C -0.31101955 -2.48908612 0.30993008
C 0.91461347 -2.38153957 -0.38570649
C 1.64499082 -1.23096789 -0.32214770
C 1.18929866 -0.11529770 0.43517310
C -0.04923307 -0.23027917 1.12421277
C -0.76996298 -1.38734684 1.06604739
H 1.28080345 -3.21514052 -0.97130659
H 2.58956070 -1.15543317 -0.85179865
H -0.42496752 0.62184335 1.67914715
H -1.71274512 -1.46747726 1.59366118

C	2.23680967	0.94423922	0.91670141
H	1.98997234	1.37143756	1.87945540
H	3.26024976	0.60828229	0.81257057
H	1.98494008	1.19145391	-1.21018540
C	0.43059945	2.38240305	-0.25983039
C	-0.39794845	2.46051533	-1.37973417
C	0.23307735	3.25930306	0.80503893
C	-1.42366568	3.39032353	-1.42608111
H	-0.23837921	1.78674600	-2.21573244
C	-0.79294778	4.19321507	0.75520876
H	0.89116916	3.23991233	1.66632697
C	-1.62437442	4.25555418	-0.35554754
H	-2.06411132	3.44621027	-2.29840644
H	-0.93543798	4.88050473	1.58084570
H	-2.42302627	4.98728171	-0.39308099
C	1.51538002	1.37551899	-0.25104032
C	-1.12164207	-3.70228345	0.26326992
C	-1.16192962	-4.54330484	-1.00782474
C	-0.44494468	-5.06583078	0.17526728
H	-2.03796731	-3.65938616	0.83763744
H	-0.58690205	-4.18467912	-1.85225343
H	-2.12682177	-4.96372698	-1.25927519
H	-0.90538780	-5.85355583	0.75701360
H	0.63767759	-5.07762184	0.16888537

*

A13

C	-1.28606121	-2.95711719	-0.57905077
C	-0.05396416	-3.15739480	0.04204408
C	0.51671319	-2.14747639	0.80295238
C	-0.14985514	-0.93418996	0.95793402
C	-1.37000095	-0.73050749	0.31778853
C	-1.94420609	-1.74553722	-0.43800707
H	1.47079480	-2.31899791	1.29448808
H	-1.88567981	0.21928065	0.42002757
H	-2.90320283	-1.58619748	-0.91631730
C	0.47153201	0.19446530	1.79205025
H	-0.27795876	0.72787578	2.37088110
H	1.23106911	-0.21644163	2.45853928
H	1.96236011	0.53059498	0.25810703
C	0.64865421	2.18584672	0.17098798
C	1.34490474	2.69678673	-0.95841720
C	-0.44709376	2.91872335	0.70485738
C	0.96052380	3.88643474	-1.53135853
H	2.18033989	2.13428515	-1.36126707
C	-0.82173714	4.10483466	0.12580104
H	-0.97951125	2.54771487	1.57131259
C	-0.11972047	4.58322845	-0.98824484
H	1.48651981	4.28055579	-2.39176472
H	-1.65141602	4.67416191	0.52595995
H	-0.42330188	5.52270901	-1.43824655
C	1.08967372	0.98694579	0.72238384
H	-1.70856250	-3.76439207	-1.16622411
C	0.65457750	-4.46590046	-0.10789360
O	0.23241976	-5.36979651	-0.77735822
H	1.61157334	-4.55339466	0.44431206

*

A14

C	0.04032073	-2.96110502	0.25614276
C	1.20218660	-2.80648555	-0.47953952
C	1.83729663	-1.57416066	-0.46142360
C	1.31167376	-0.53009714	0.29561842
C	0.13235020	-0.70919816	1.01494781
C	-0.50921060	-1.93800387	1.00779992
H	1.59408957	-3.64069283	-1.04574207
H	2.75597303	-1.43694159	-1.02139566
H	-0.28448883	0.10600758	1.59707359
H	-1.41818052	-2.11167238	1.56764479
C	2.00064282	0.83817584	0.31078035
H	2.01065492	1.26843415	1.30926716
H	3.02621783	0.73293613	-0.04974308
H	1.31241290	1.28257548	-1.69674512
C	0.29118474	2.61832987	-0.41985041
C	-0.43958794	3.13701626	-1.52728427
C	0.06719151	3.15957453	0.87895588
C	-1.35296292	4.14710699	-1.34418680
H	-0.26514013	2.72145909	-2.51399176
C	-0.84655389	4.16731255	1.04928675
H	0.62121835	2.78195308	1.72880974
C	-1.55226050	4.65590757	-0.05917234
H	-1.91232445	4.54580603	-2.18107851
H	-1.02391264	4.58974001	2.03044689
H	-2.27258448	5.45428115	0.08681985
C	1.20673734	1.60608799	-0.66220436
N	-0.64706346	-4.27740968	0.23605423
O	-0.13941433	-5.15304088	-0.42952118
O	-1.66612976	-4.37507500	0.88486706

*

A15

C	-0.60580713	-3.11729635	0.12996652
C	0.77264774	-3.11583563	-0.05388292
C	1.53414727	-2.00011078	0.24097679
C	0.90478556	-0.86613462	0.74515304
C	-0.47631324	-0.84014823	0.91743543
C	-1.21576176	-1.96957626	0.61982338
H	2.60708403	-2.04908259	0.09749418
H	-0.99529337	0.03339122	1.29433137
C	1.72020984	0.38932778	1.06523061
H	1.40161070	0.84730238	1.99827566
H	2.77889211	0.13140288	1.12914566
H	1.93059648	0.85042510	-1.04306272
C	0.65965498	2.33661133	-0.24228724
C	0.47913626	2.89978049	-1.53755942
C	0.01932802	2.94171242	0.87671074
C	-0.30800834	4.01362362	-1.70651695
H	0.96936821	2.43541490	-2.38652350
C	-0.76364209	4.05276766	0.69600906
H	0.15434800	2.53087293	1.86913946
C	-0.92561582	4.58304356	-0.59135478
H	-0.45038904	4.44638401	-2.68871508
H	-1.25378924	4.52372000	1.53894281
H	-1.54739522	5.46251676	-0.72329326
C	1.47036720	1.21652041	-0.12663460
F	-2.53513243	-1.96970407	0.79298063
F	-1.32243563	-4.19147181	-0.15737652
F	1.35239003	-4.21717797	-0.51990170

*

A16

C	-0.33114248	-2.95973744	-0.37208266
C	0.99985357	-2.85263765	-0.80822804
C	1.71788086	-1.74567790	-0.46568507
C	1.14324191	-0.70020778	0.31502765
C	-0.21969100	-0.83122236	0.74414466
C	-0.93660265	-1.93223104	0.41429163
H	1.41691487	-3.65413324	-1.40439924
H	2.74922817	-1.64878816	-0.78851664
H	-0.67249562	-0.02488210	1.31153171
C	2.10980925	0.19052511	1.16356542
H	1.67972463	0.50261202	2.10577231
H	3.12048462	-0.19071973	1.22401925
H	2.31890219	0.75291045	-0.91340595
C	0.62541654	1.87930639	-0.12304315
C	-0.04177421	2.10086215	-1.32790462
C	0.32701974	2.66986521	0.98350885
C	-1.00885714	3.08865926	-1.41923918
H	0.19811140	1.49479219	-2.19605081
C	-0.64086674	3.66271781	0.88952390
H	0.86518825	2.53864579	1.91559161
C	-1.31242411	3.86887867	-0.30768108

H	-1.52256985	3.25693215	-2.35853004
H	-0.85975211	4.28360640	1.75042513
H	-2.06403881	4.64634783	-0.38044824
C	1.65256043	0.80760833	-0.06047644
O	-1.02815345	-4.01985565	-0.70575386
H	-1.92667959	-3.95963679	-0.33837561
O	-2.22868509	-2.19846790	0.73897004
H	-2.63044485	-1.48291581	1.24411009

*

A17

C	-0.87964466	-2.23481495	0.07746070
C	0.54568945	-2.31968940	-0.12848201
C	1.31621103	-1.24910784	0.20036619
C	0.73430318	-0.04741824	0.73935245
C	-0.67482778	0.00114615	0.92276518
C	-1.46441519	-1.06368814	0.60454299
H	2.39001826	-1.28252676	0.06560432
H	-1.12183523	0.91349669	1.30066944
H	-2.53432981	-1.00884538	0.74471611
C	1.61867516	0.83665496	1.67963595
H	1.07171418	1.29426038	2.49279991
H	2.55264342	0.36779088	1.95929236
H	2.24712521	1.13511743	-0.36737310
C	0.59653616	2.51714936	-0.00780179
C	0.15490304	2.66659021	-1.32250678
C	0.24762443	3.46915377	0.94503801
C	-0.64036780	3.74492659	-1.67438174
H	0.43545015	1.93224519	-2.07132989
C	-0.54820459	4.55243226	0.59052554
H	0.61310904	3.38844195	1.96262546
C	-0.99649246	4.68854226	-0.71552104
H	-0.97927610	3.85534084	-2.69789624
H	-0.80823283	5.29528389	1.33561951
H	-1.61443836	5.53510036	-0.99147633
C	1.45006496	1.34758877	0.33600554
O	-1.54881518	-3.30000549	-0.26473215
C	-2.98240400	-3.33939588	-0.10237598
H	-3.24320925	-3.21903274	0.94997436
H	-3.44924756	-2.56654279	-0.71457397
H	-3.27767094	-4.32436994	-0.45194859
O	0.96425339	-3.48696253	-0.63723992
C	2.36682382	-3.63836101	-0.85807009
H	2.72630592	-2.89829746	-1.57916339
H	2.91739552	-3.54989670	0.08308873
H	2.49511479	-4.63804932	-1.26477194

*

A18

C	-0.08231981	-2.53400372	0.04014075
C	1.18552000	-2.30594268	-0.60736707
C	1.80996148	-1.11770387	-0.47968010
C	1.25239752	-0.03118450	0.28785834
C	-0.02155314	-0.27726813	0.91893433
C	-0.65869810	-1.46016389	0.81010952
H	1.64204842	-3.08905662	-1.19392482
H	2.76744215	-0.96270054	-0.96742491
H	-0.47843062	0.52884245	1.48218790
H	-1.61290247	-1.59709221	1.29642196
C	2.23255795	0.95328533	0.97868911
H	1.90046643	1.30035538	1.94815676
H	3.27654655	0.67456017	0.92256887
H	2.12528216	1.41630300	-1.13823168
C	0.44820263	2.43927139	-0.18180237
C	-0.47842266	2.46154360	-1.22381967
C	0.33078588	3.35663287	0.85658273
C	-1.51596831	3.37983419	-1.21919591
H	-0.38452709	1.75337176	-2.04148262
C	-0.70980797	4.27935785	0.86063092
H	1.06165567	3.37253687	1.65741888
C	-1.63511678	4.28949727	-0.17283641
H	-2.23116944	3.39171467	-2.03359171
H	-0.78943802	4.99613416	1.66975858
H	-2.44410067	5.01078064	-0.17025991
C	1.55298728	1.43706778	-0.21711984
N	-0.70273428	-3.69599765	-0.07384762
C	-1.98913390	-3.93429622	0.59370934
C	-0.12481367	-4.78687131	-0.86947072
H	-2.30634510	-4.95201401	0.38702021
H	-1.88946093	-3.81465122	1.67365393
H	-2.75094789	-3.24927565	0.21751029
H	-0.80202322	-5.63521877	-0.84283392
H	0.00364562	-4.47866936	-1.90829910
H	0.83692052	-5.09658094	-0.45686632

*

A19

C	-0.24646144	-2.43353278	0.03887501
C	1.14178315	-2.36961451	0.07390845
C	1.75778567	-1.16968779	0.42745960
C	0.99396524	-0.06264985	0.77014534
C	-0.39193318	-0.14451165	0.73541677
C	-1.02426305	-1.32789834	0.36232690
H	2.84073400	-1.12795404	0.44286663
H	-1.00848355	0.70659845	1.00184347
C	1.65471287	1.25892975	1.15607854
H	1.22014643	1.65941057	2.06949802
H	2.72335251	1.09470403	1.32040595
H	2.02808537	1.84054457	-0.90674188
C	0.69230603	3.27124750	-0.12782963
C	0.63672750	3.92499034	-1.39786359
C	-0.07399649	3.79377927	0.95976087
C	-0.14706037	5.03781256	-1.57114432
H	1.22058038	3.52561958	-2.22034785
C	-0.85232031	4.90420877	0.77283005
H	-0.03806044	3.31638764	1.93071771
C	-0.88729541	5.52020629	-0.48805753
H	-0.19621825	5.53747767	-2.53038454
H	-1.43824308	5.31086948	1.58759347
H	-1.50871099	6.39955444	-0.62450815
C	1.48770500	2.15050423	-0.01309631
N	-0.90834347	-3.69477347	-0.35486755
O	-0.55864708	-4.68796937	0.23715269
O	-1.73598597	-3.60644684	-1.23253740
C	-2.46406099	-1.35642686	0.34931300
N	-3.60985621	-1.29131532	0.38019119
C	1.97382956	-3.49005817	-0.28109347
N	2.69984458	-4.32852916	-0.57725545

*

A2

C	-0.17758476	-3.09245579	0.25076785
C	0.90637078	-2.97487380	-0.61246708
C	1.64705911	-1.80569168	-0.61351290
C	1.31957280	-0.76459940	0.25568967
C	0.21365296	-0.88817969	1.09548476
C	-0.52888636	-2.05614433	1.10817165
H	1.16467491	-3.79662103	-1.26812411
H	2.50207302	-1.71609927	-1.27571983
H	-0.06459027	-0.07541390	1.75822810
H	-1.37613502	-2.17016921	1.77215907
C	2.14060771	0.53274653	0.25420786
H	2.21534068	0.96780315	1.24695610
H	3.13423298	0.34617184	-0.15258939
H	1.37897647	0.96935001	-1.72372352
C	0.35608320	2.26988393	-0.40052072
C	-0.45360648	2.74209775	-1.46619261
C	0.19680687	2.82775321	0.89549359
C	-1.38853122	3.72758498	-1.24483655
H	-0.32726614	2.31405460	-2.45486852
C	-0.73766922	3.81148223	1.10575854
H	0.81699093	2.48787968	1.71518722

C	-1.52742105	4.25610530	0.03836728
H	-2.00933402	4.09062299	-2.05426159
H	-0.86367706	4.24782557	2.08884707
H	-2.26270306	5.03416024	0.21450101
C	1.30250037	1.28355073	-0.68452105
Cl	-1.10384447	-4.54627764	0.25500803

*

A20

C	-0.23889865	-1.85681015	0.05859774
C	1.12576490	-1.84863062	0.32254417
C	1.71380975	-0.67974191	0.78498095
C	0.94772136	0.45735674	0.99953188
C	-0.41307375	0.43079656	0.72643488
C	-1.01943888	-0.72390111	0.25595548
H	2.77785653	-0.67311938	0.98742819
H	-1.02450956	1.31032532	0.88867240
C	1.58785638	1.75244887	1.49964361
H	1.00340679	2.19356969	2.30394994
H	2.59491564	1.54077049	1.86737118
H	2.38838352	2.26439951	-0.45502379
C	0.93243716	3.72522989	-0.01719172
C	1.13962005	4.32987388	-1.29371519
C	-0.02937292	4.28917367	0.87434809
C	0.41853359	5.43894214	-1.66079706
H	1.87168233	3.89741023	-1.96740505
C	-0.74313753	5.39502031	0.49549891
H	-0.19287990	3.84784590	1.84922008
C	-0.51828949	5.96417650	-0.76706823
H	0.56859229	5.90235469	-2.62772481
H	-1.47561530	5.83352503	1.16160535
H	-1.08958524	6.84031082	-1.05666836
C	1.67986818	2.60678904	0.29817901
C	2.00700454	-3.07895007	0.13591074
F	3.29339084	-2.70844290	0.00347124
F	1.66955132	-3.76367434	-0.96090909
F	1.92691376	-3.89167361	1.19133986
C	-2.51190049	-0.68528310	-0.04672316
F	-3.09708774	0.31570961	0.63929401
F	-3.12107966	-1.82070646	0.29719733
F	-2.73050017	-0.45657862	-1.34657001
N	-0.87603328	-3.09641188	-0.44922220
O	-1.25241022	-3.06454612	-1.59700432
O	-0.95662411	-4.01212362	0.33281995

*

A21

C	-0.21384113	-2.61418539	0.00674894
C	1.16351633	-2.49209179	-0.09083484
C	1.80137099	-1.30415081	0.22340439
C	1.03791837	-0.23143124	0.66710253
C	-0.34628314	-0.33202551	0.76805539
C	-0.95779970	-1.52787283	0.43710638
H	2.87973689	-1.25082574	0.13601914
H	-0.96334206	0.48986082	1.11133758
C	1.70596184	1.09939701	1.01146948
H	1.35635694	1.48080581	1.96816646
H	2.78894929	0.95900108	1.05940013
H	1.83644562	1.68348226	-1.07578415
C	0.54242237	3.07466736	-0.15954430
C	0.32849775	3.71937884	-1.41445588
C	-0.11090286	3.57906890	1.00451754
C	-0.49839217	4.81217561	-1.50059072
H	0.82687472	3.33165057	-2.29650689
C	-0.93407123	4.66965748	0.90507462
H	0.04516791	3.10504027	1.96512072
C	-1.12503866	5.28055230	-0.34311697
H	-0.66638227	5.30626800	-2.44920722
H	-1.43504283	5.06369430	1.78045235
H	-1.77919402	6.14390952	-0.41009685
C	1.38570695	1.97964833	-0.12947671
N	-0.87902465	-3.88025655	-0.34644580
O	-0.61248422	-4.83604327	0.34279397
O	-1.62891026	-3.83976580	-1.29480653
F	-2.27600213	-1.64228637	0.54638003
F	1.87764938	-3.52730316	-0.51124080

*

A22

C	0.29285908	-1.90787238	0.10890335
C	1.51940829	-1.57130575	-0.57308781
C	2.01522147	-0.32154724	-0.49611246
C	1.35447444	0.72860377	0.24533493
C	0.12144914	0.37029800	0.90988059
C	-0.38486802	-0.87616582	0.85625186
H	2.04906128	-2.33136446	-1.12916225
H	2.94635120	-0.08292470	-1.00088240
H	-0.41212893	1.14538929	1.44875462
H	-1.31728472	-1.10204336	1.35334935
C	2.23616252	1.82686559	0.89060340
H	1.88354872	2.17229919	1.85345819
H	3.30311947	1.65941007	0.82458051
H	2.04993744	2.21084281	-1.23863766
C	0.28985908	3.08306984	-0.28148132
C	-0.67019745	2.95725431	-1.28510029
C	0.11652617	4.03931389	0.71277952
C	-1.79364724	3.76834446	-1.28589731
H	-0.53462123	2.21764834	-2.06840132
C	-1.01031009	4.85468958	0.71147505
H	0.86894695	4.16875846	1.48286623
C	-1.96698652	4.71804502	-0.28361285
H	-2.53445853	3.66510162	-2.07044654
H	-1.13357254	5.60261579	1.48624747
H	-2.84363533	5.35541213	-0.28553352
C	1.49044761	2.19631568	-0.30948078
N	-0.19795625	-3.12488832	0.04606120
C	-1.40351352	-3.57827572	0.77380423
C	0.38366086	-4.22052073	-0.76007486
H	-1.35859899	-3.25349807	1.81304465
H	-2.29093863	-3.14605696	0.30281920
C	-1.36506723	-5.09661339	0.62208331
H	0.61212803	-3.86658944	-1.76515269
H	1.30623540	-4.56528331	-0.28460296
C	-0.69929808	-5.29589515	-0.73976319
H	-0.75181247	-5.53462148	1.41326232
H	-2.36074676	-5.53415010	0.68189612
H	-1.41689815	-5.11731017	-1.54419042
H	-0.28127348	-6.29297032	-0.87205447

*

A23

C	0.02247589	-2.43681840	-0.10440747
C	1.38970976	-2.15822909	-0.46954047
C	1.96384408	-0.96934789	-0.19046226
C	1.24789355	0.10298402	0.44750093
C	-0.13914132	-0.14924914	0.75485138
C	-0.70500874	-1.33688906	0.49679480
H	1.98142463	-2.93728674	-0.92542390
H	3.00880588	-0.81367247	-0.43910530
H	-0.75571129	0.64434167	1.16172004
C	2.04652711	1.06740528	1.36396442
H	1.50132409	1.39371444	2.23952471
H	3.07556064	0.78393680	1.54139680
H	2.42333100	1.57817387	-0.71180687
C	0.57156063	2.58614482	-0.13558255
C	-0.08499073	2.64760627	-1.36463481
C	0.20826487	3.46585144	0.87832121
C	-1.10039395	3.56746624	-1.57067919
H	0.20221805	1.96918077	-2.16223632
C	-0.80975556	4.39032199	0.67079840
H	0.73095501	3.45258663	1.82821159
C	-1.46700706	4.43926361	-0.54980590

H -1.60545163 3.60949577 -2.52878597
H -1.08152922 5.07775188 1.46344507
H -2.25908905 5.16116423 -0.71145641
C 1.65729348 1.58160148 0.05577419
N -0.50901839 -3.62331357 -0.33098142
C -1.77651786 -4.10105922 0.25325909
C 0.19707911 -4.61870564 -1.15001669
H -1.69477296 -5.18137704 0.35805828
H -1.93777948 -3.66956973 1.23477319
H -2.61718278 -3.86334639 -0.39944318
H -0.53540987 -5.33960942 -1.50570281
H 0.65767035 -4.14744947 -2.01612358
H 0.95422661 -5.14183381 -0.56080664
F -2.01969083 -1.49284713 0.73349266
*

A24
C -0.54015388 -2.16829420 -0.23612192
C 0.83305498 -2.25174065 -0.01850866
C 1.52603178 -1.19385730 0.54073442
C 0.84012547 -0.03421267 0.90101975
C -0.52372333 0.06129047 0.67103992
C -1.21062303 -1.00982909 0.11095880
H 2.59293515 -1.27902861 0.71041274
H -1.06716184 0.96377158 0.93140782
C 1.58773951 1.15652633 1.50884046
H 1.03775599 1.58638970 2.34254448
H 2.57154716 0.83162541 1.85344201
H 2.42927784 1.72544136 -0.40901426
C 1.03899163 3.23291381 0.09192420
C 1.27327270 3.88042665 -1.15576456
C 0.10991703 3.80290777 1.01029854
C 0.60696133 5.03957682 -1.47157246
H 1.98153920 3.44244349 -1.85092345
C -0.54977372 4.95899428 0.68342398
H -0.07211500 3.32795486 1.96589098
C -0.30090808 5.57123114 -0.55319282
H 0.77862749 5.53627225 -2.41817288
H -1.25767887 5.40285002 1.37217173
H -0.82870606 6.48618044 -0.80199386
C 1.73415353 2.06442162 0.35784846
C 1.54748091 -3.53341046 -0.39280850
F 2.87979037 -3.40853014 -0.28523000
F 1.26767381 -3.88246475 -1.65839878
F 1.16382227 -4.54416339 0.40111836
H -1.07521499 -3.00035476 -0.67701616
C -2.69983554 -0.86398349 -0.10921523
F -2.96046131 0.21444053 -0.87707045
F -3.33903070 -0.67709347 1.05894757
F -3.23093280 -1.93283453 -0.70742522
*

A3
C -1.70378427 -2.23894052 0.26671551
C -0.33563519 -2.49262460 0.24791156
C 0.56547019 -1.52110754 0.64777974
C 0.09285643 -0.28375571 1.08049742
C -1.27265972 -0.01819625 1.07588585
C -2.17091350 -1.00143909 0.68335192
H 1.62792600 -1.73535831 0.64219167
H -1.64124531 0.95155373 1.39480484
H -3.23542910 -0.80162096 0.70013754
C 1.08251890 0.80551021 1.51499283
H 0.68345731 1.40907717 2.32591450
H 2.02356121 0.34708674 1.82250545
H 1.80758301 0.99296850 -0.51738552
C 0.69714603 2.74943209 -0.12789101
C 0.88721449 3.18240239 -1.46910647
C -0.02091937 3.57885752 0.77770689
C 0.37848921 4.38947097 -1.88771422
H 1.43612705 2.54652150 -2.15545147
C -0.52330069 4.78146480 0.34965682
H -0.16300495 3.26775703 1.80477469
C -0.32389874 5.18148284 -0.97836137
H 0.51926993 4.72460462 -2.90759663
H -1.06734116 5.42395763 1.03072273
H -0.72441947 6.13503186 -1.30650738
C 1.24544295 1.52775913 0.24656096
C 0.14490406 -3.85281084 -0.20785859
F 1.48584715 -3.92279890 -0.24571325
F -0.31333788 -4.13353330 -1.43976683
F -0.29289676 -4.81905554 0.61407563
H -2.39888923 -3.00952131 -0.04616118
*

A4
C 0.21209490 -2.58557587 0.22181767
C 1.32147739 -2.36219399 -0.58547977
C 1.93190282 -1.11849722 -0.58541925
C 1.44046705 -0.10378168 0.23254562
C 0.31764751 -0.32861437 1.02354614
C -0.29147595 -1.57441050 1.02851445
H 1.71243544 -3.16176683 -1.20274932
H 2.80730380 -0.94826932 -1.20316583
H -0.07717350 0.46248121 1.65264759
H -1.15187885 -1.76030498 1.65895489
C 2.10328139 1.27944597 0.23332616
H 2.14001967 1.70653423 1.23223499
H 3.11364861 1.20133059 -0.17178699
H 1.31534572 1.69615838 -1.74114301
C 0.25844710 2.95406716 -0.41151936
C -0.55531763 3.42224457 -1.48056738
C 0.06204301 3.47946401 0.89641532
C -1.52445967 4.37013215 -1.25166092
H -0.40124709 3.01870476 -2.47560365
C -0.90723202 4.42550892 1.11307330
H 0.68055580 3.14055980 1.71763829
C -1.69616216 4.86574694 0.04185816
H -2.14773932 4.73001816 -2.06053086
H -1.06367117 4.83614880 2.10278120
H -2.45970103 5.61492307 0.22394776
C 1.23066126 2.00454131 -0.70027756
C -0.47614209 -3.93290719 0.17917375
F -1.15469554 -4.17925592 1.30951777
F 0.40513384 -4.92794856 0.00018849
F -1.35437778 -3.98246346 -0.83998649
*

A5
C -0.78208209 -3.07685302 0.01606371
C 0.35942935 -3.08104598 -0.81686146
C 1.21814795 -2.02891788 -0.75913856
C 0.98236938 -0.92352626 0.11616711
C -0.18521932 -0.95035044 0.93644352
C -1.04729427 -2.00167407 0.89582097
H 0.52018625 -3.92431991 -1.47628574
H 2.10395887 -2.01932044 -1.38632384
H -0.39071007 -0.10402120 1.58208535
H -1.93598625 -2.01880565 1.51728003
C 2.20957561 -0.07239244 0.58600401
H 2.11290411 0.28873588 1.60117607
H 3.16354020 -0.52446907 0.34828630
H 1.79581981 0.41543500 -1.47583132
C 0.50768480 1.68845774 -0.26170317
C -0.45449583 1.94733981 -1.23811912
C 0.58719011 2.50893465 0.86067908
C -1.33894789 3.00234830 -1.08412159
H -0.50990148 1.31785787 -2.12087199
C -0.29818479 3.56880059 1.01182973
H 1.35434655 2.34706527 1.60955536
C -1.26374264 3.81246765 0.04456439
H -2.08371138 3.19924487 -1.84630734

H -0.22444214 4.21119253 1.88148987
H -1.95138033 4.64180365 0.16285819
C 1.43680109 0.54824902 -0.46167579
O -1.58344641 -4.12054699 -0.08107115
H -2.34034898 -4.05376100 0.51774891
*

A6
C -0.21124780 -2.82022453 -0.12674880
C 1.02687696 -2.64452354 -0.80236754
C 1.71723099 -1.49034420 -0.63930913
C 1.21902750 -0.43737287 0.19682481
C -0.03300049 -0.64250219 0.85337990
C -0.73473026 -1.79890398 0.70720464
H 1.38572466 -3.44952327 -1.43095775
H 2.66740835 -1.35021297 -1.14488922
H -0.43490046 0.15808331 1.46450380
H -1.68139262 -1.92859325 1.21169278
C 2.25410555 0.53131969 0.85881695
H 1.97289241 0.84570557 1.85489161
H 3.28113165 0.20835368 0.75101436
H 2.07076711 1.03718831 -1.23382959
C 0.47724908 2.10004722 -0.18852722
C -0.39290847 2.25832222 -1.26721040
C 0.33103835 2.90722112 0.93589387
C -1.40752262 3.20010632 -1.21384762
H -0.27314580 1.63964942 -2.15127163
C -0.68558680 3.85335889 0.98727530
H 1.02250211 2.82466581 1.76688033
C -1.55733114 3.99698858 -0.08305413
H -2.07913184 3.31922412 -2.05603129
H -0.78757366 4.48616707 1.86115252
H -2.34732702 4.73789937 -0.04310672
C 1.55535870 1.07999268 -0.28099371
O -0.80726808 -3.96478994 -0.34218877
C -2.06890733 -4.25960438 0.29595432
H -1.95110209 -4.24897637 1.38027574
H -2.82661200 -3.54372242 -0.02533733
H -2.32987202 -5.25810245 -0.04252042
*

A7
C -0.23858122 -2.87116583 -0.11813418
C 0.95793870 -2.67496672 -0.83401525
C 1.70693861 -1.54325236 -0.64685583
C 1.28504705 -0.54703252 0.26583221
C 0.06955117 -0.74001518 0.96940245
C -0.66608799 -1.87679266 0.78722630
H 1.28659696 -3.43411898 -1.53381778
H 2.63425509 -1.40548086 -1.19389838
H -0.28314049 0.03493750 1.64002221
H -1.59099905 -2.00139272 1.33388056
C 2.31000752 0.52155614 0.75547563
H 2.10936350 0.86154235 1.76304634
H 3.33673758 0.23150734 0.57109302
H 1.94908842 0.94840202 -1.32572032
C 0.43391216 2.02545743 -0.20758631
C -0.37344509 2.25206533 -1.32448063
C 0.19073627 2.72903885 0.97367535
C -1.42164977 3.15480784 -1.25646844
H -0.17834213 1.71511312 -2.24728591
C -0.85587658 3.63549480 1.03760183
H 0.83028960 2.59615569 1.83872237
C -1.66526532 3.84421825 -0.07363509
H -2.04528493 3.32685058 -2.12561592
H -1.03393758 4.18970494 1.95153958
H -2.48113168 4.55583398 -0.02092026
C 1.54221837 1.06820490 -0.32870403
C -0.99537919 -4.10204147 -0.35563304
C -2.09743715 -4.46417012 0.29918802
H -0.59496823 -4.75689008 -1.12328024
H -2.59241240 -5.39949861 0.06674103
H -2.54364779 -3.86356346 1.08325379
*

A8
C -0.28755397 -3.17026714 0.19065386
C 0.80723833 -3.03848663 -0.67023975
C 1.57100044 -1.88594846 -0.65221099
C 1.26392811 -0.85684208 0.23902760
C 0.15587573 -0.97369770 1.07753410
C -0.60568651 -2.12776649 1.06689095
H 1.05382849 -3.84907369 -1.34466152
H 2.42735978 -1.79760973 -1.31295333
H -0.10490303 -0.16714405 1.75480034
H -1.45338075 -2.23283288 1.73245638
C 2.11201441 0.42379868 0.26183527
H 2.18130816 0.84823194 1.25943546
H 3.10582039 0.22278654 -0.13685147
H 1.37512819 0.88383917 -1.71944611
C 0.34421042 2.17769479 -0.39408453
C -0.45412738 2.66132173 -1.46215791
C 0.17809131 2.72719009 0.90382495
C -1.38570303 3.65051513 -1.24124042
H -0.32266440 2.23930341 -2.45274176
C -0.75277019 3.71482511 1.11387056
H 0.79017410 2.37847905 1.72587418
C -1.53200359 4.17118604 0.04408683
H -1.99812821 4.02213978 -2.05315543
H -0.88356220 4.14489941 2.09910026
H -2.26437731 4.95199436 0.21989368
C 1.28921859 1.18782189 -0.67807549
C -1.08032505 -4.36527617 0.17176098
C -1.74110096 -5.36469790 0.15537300
H -2.32804425 -6.25575203 0.14194211
*

A9
C -0.20844606 -3.31663649 0.25978054
C 0.90271906 -3.19025777 -0.57348658
C 1.61169853 -2.00077998 -0.59364412
C 1.22254860 -0.94320199 0.22489897
C 0.10178567 -1.06852786 1.04208224
C -0.60908614 -2.25646376 1.07161154
H 1.20713911 -4.02368371 -1.19450709
H 2.48385053 -1.90693961 -1.23183745
H -0.21276440 -0.24278869 1.67176633
H -1.47196804 -2.36866559 1.71630005
C 1.99456925 0.38076940 0.19994915
H 2.08767772 0.80761695 1.19541724
H 2.98756863 0.21722027 -0.22295902
H 1.21109619 0.86280585 -1.76285879
C 0.30474431 2.22015296 -0.42202885
C -0.47630036 2.76368040 -1.48111433
C 0.17739999 2.76339865 0.88814871
C -1.34682500 3.79977262 -1.24074520
H -0.37532425 2.34663970 -2.47740523
C -0.69427224 3.79707108 1.11611457
H 0.77221981 2.36800563 1.70158512
C -1.45233915 4.30994326 0.05447153
H -1.94417059 4.21729726 -2.04143986
H -0.79771148 4.22143123 2.10699059
H -2.13847383 5.12857180 0.24569368
C 1.17517087 1.18182948 -0.72229083
C -0.95240801 -4.55107694 0.27918233
N -1.55041432 -5.53198273 0.29201177
*

B1
C -0.33988926 -3.64057811 0.39830280
C 0.90700901 -3.15082538 0.81796828
C 1.35326224 -1.93279458 0.37558627

C	0.56277099	-1.14837322	-0.51157411
C	-0.70203406	-1.65623536	-0.92863550
C	-1.13412000	-2.87205465	-0.47954602
H	1.51172377	-3.74272372	1.49453820
H	2.31545484	-1.54979041	0.69878830
H	-1.32018126	-1.08130617	-1.60634920
H	-2.09479903	-3.26089035	-0.79712788
C	1.06705240	0.06628096	-0.94366370
H	2.05576850	0.34104655	-0.58017305
H	1.09208098	1.48879276	-2.50688396
H	-0.50480020	0.74573620	-2.25318940
C	0.10214856	2.16828494	-0.71301278
C	-0.99956588	2.02562794	0.12691785
C	0.95016981	3.26355606	-0.56845616
C	-1.27531644	2.99499135	1.08058728
H	-1.65353218	1.16471818	0.02753789
C	0.67519687	4.22873462	0.38919954
H	1.81065038	3.37581368	-1.22026649
C	-0.43713630	4.09548498	1.21266164
H	-2.14361350	2.89163243	1.72068798
H	1.32537111	5.09000829	0.48826252
H	-0.65177387	4.85230695	1.95814141
C	0.40105117	1.09159445	-1.76116186
C	-0.82487461	-4.97849938	0.85288084
H	-1.88402681	-4.93900285	1.11476676
H	-0.72221650	-5.69315632	0.02918278
H	-0.25322118	-5.35109725	1.70179648

*

B10

C	-0.81394454	-3.55803422	-0.35693896
C	-0.06316473	-3.23285780	0.78167208
C	0.62864926	-2.04032844	0.75967525
C	0.58184162	-1.17742688	-0.36853221
C	-0.18818812	-1.54724293	-1.50075642
C	-0.87874228	-2.73429429	-1.48418250
H	1.22312385	-1.74235872	1.61761063
H	-0.22545677	-0.90717948	-2.37262647
H	-1.46942363	-3.03894665	-2.33928419
C	1.32337944	0.00123637	-0.32134841
H	1.91617660	0.16750645	0.57636245
H	2.33737569	1.51081923	-1.41087224
H	0.89923916	0.86955709	-2.25025957
C	0.46845143	2.10773690	-0.49939394
C	-0.92023914	2.00732026	-0.54759341
C	1.07079120	3.08553002	0.29064607
C	-1.70447054	2.91262637	0.15170688
H	-1.39127183	1.23222380	-1.14366470
C	0.28392058	3.98512016	0.99352626
H	2.15261596	3.16123823	0.33325889
C	-1.10250154	3.89981445	0.92306728
H	-2.78467272	2.84722059	0.09668441
H	0.75204506	4.75829744	1.59121975
H	-1.71540831	4.60555095	1.47161871
C	1.33227293	1.10806271	-1.28256826
H	-1.36639520	-4.49330752	-0.36345910
C	-0.02738988	-4.16096287	1.96555288
H	0.59560684	-3.75918913	2.76463475
H	-1.03341169	-4.31747987	2.36079921
H	0.37219286	-5.13548422	1.67653688

*

B11

C	-0.82367007	-3.52483984	-0.22123191
C	-0.01116115	-3.14299771	0.85002097
C	0.69044292	-1.95296312	0.76426868
C	0.57724438	-1.14419977	-0.38848576
C	-0.24884840	-1.54918869	-1.46434129
C	-0.93767457	-2.73565437	-1.36671920
H	1.32248725	-1.64947584	1.59119353
H	-0.32925353	-0.94521519	-2.35790342
H	-1.57044935	-3.07043821	-2.17938857
C	1.33393819	0.03627714	-0.42849955
H	1.99531819	0.21848882	0.41612631
H	2.25322765	1.54627174	-1.61015428
H	0.77413484	0.85722553	-2.34512925
C	0.43446878	2.07357664	-0.55348122
C	-0.95429237	1.95399911	-0.53170597
C	1.06733816	3.03663080	0.23345007
C	-1.71078630	2.83276084	0.22799650
H	-1.44512426	1.18507698	-1.11918623
C	0.30776897	3.90853110	0.99695741
H	2.14915655	3.12293417	0.22369237
C	-1.07991826	3.80774598	0.99259490
H	-2.79151199	2.75560179	0.22736409
H	0.79651071	4.67137915	1.59123108
H	-1.67184667	4.49137812	1.59005675
C	1.27348307	1.11203651	-1.41499017
H	-1.37534576	-4.45897688	-0.16412887
O	0.13251510	-3.87296260	1.97492567
H	-0.38741215	-4.68388088	1.93616253

*

B12

C	-0.10339053	-2.99879209	-0.28856563
C	0.70424183	-2.52956663	0.77273971
C	1.19054772	-1.25598420	0.75502622
C	0.90049745	-0.36933818	-0.32876015
C	0.08314761	-0.85536361	-1.39543758
C	-0.39907765	-2.12940187	-1.36800995
H	0.93781973	-3.18213820	1.60424965
H	1.80837667	-0.89525380	1.57057844
H	-0.15229435	-0.21018404	-2.23222497
H	-1.01665401	-2.49660626	-2.17897599
C	1.42312932	0.90354842	-0.29967159
H	2.03850945	1.16038894	0.56093787
H	2.13691567	2.49700581	-1.48341771
H	0.70733253	1.70039553	-2.16959903
C	0.30610681	2.97963841	-0.45429069
C	-1.05069178	2.70554440	-0.30319811
C	0.85625009	4.10716914	0.14812763
C	-1.85950838	3.57088866	0.41958676
H	-1.48192952	1.82070740	-0.76175865
C	0.04679003	4.96953740	0.87448638
H	1.91327039	4.32450197	0.03251259
C	-1.31034878	4.70258722	1.01007690
H	-2.91815979	3.36266802	0.52186587
H	0.47514525	5.85515772	1.32914632
H	-1.94129329	5.37898930	1.57485902
C	1.18755425	2.01234933	-1.24456771
C	-0.64196864	-4.35177265	-0.30900749
C	0.16758698	-5.50190607	0.28686784
C	-1.03806159	-5.03352185	0.99914674
H	-1.24585008	-4.59097836	-1.17461557
H	1.14287235	-5.25742137	0.68832493
H	0.12015354	-6.43362512	-0.26145833
H	-1.93670286	-5.63468064	0.95373025
H	-0.92024994	-4.45615601	1.90736358

*

B13

C	-0.81339660	-3.19993723	-0.49645536
C	0.13823070	-2.88606208	0.48045406
C	0.81869409	-1.69235029	0.40205328
C	0.55484897	-0.78763414	-0.65635690
C	-0.41133354	-1.13178408	-1.63528727
C	-1.08579482	-2.32920487	-1.55146854
H	1.55983218	-1.43418575	1.15327671
H	-0.61267084	-0.45815058	-2.45866147
H	-1.81997876	-2.59825711	-2.30065541
C	1.28770722	0.40527744	-0.70715520
H	2.05571667	0.53523165	0.05274057

H 2.02744808 2.02237375 -1.87449317
H 0.47281032 1.36183646 -2.46463674
C 0.35677866 2.42164071 -0.54905419
C -1.01827613 2.27908424 -0.36692378
C 1.07808972 3.32242436 0.23591108
C -1.67871316 3.07602199 0.55481584
H -1.57629378 1.55934152 -0.95692379
C 0.41495865 4.11241974 1.16129481
H 2.14975832 3.42710314 0.09976583
C -0.96196086 3.99032238 1.31924497
H -2.75090835 2.98322635 0.67993681
H 0.96987802 4.82874633 1.75533106
H -1.47839019 4.61002962 2.04307357
C 1.08710257 1.55259157 -1.58941890
H -1.33383753 -4.14882328 -0.41148385
C 0.40099053 -3.85474236 1.59598642
O -0.18543504 -4.89685686 1.69134434
H 1.17187951 -3.54585854 2.32746045
*

B14
C -0.17090399 -2.61599883 0.19091151
C 0.38025095 -1.92600818 1.25651743
C 0.93010026 -0.67864172 1.01490859
C 0.91465134 -0.13759350 -0.27277771
C 0.36484219 -0.86626041 -1.32984643
C -0.18348230 -2.11722864 -1.10014501
H 0.37516313 -2.36784125 2.24372000
H 1.37043854 -0.11885167 1.83296662
H 0.38259948 -0.47941653 -2.34157217
H -0.60822790 -2.70933025 -1.89950471
C 1.52503232 1.18399049 -0.47886347
H 2.30219210 1.45348885 0.22651742
H 2.18045303 2.70164222 -1.88297584
H 0.82460855 1.64101745 -2.48627786
C 0.44221468 2.57885245 -0.51292690
C -0.93304684 2.25761704 -0.52706098
C 0.90837739 3.63682808 0.29939944
C -1.81552420 2.99541795 0.22561247
H -1.28268525 1.42232895 -1.12256349
C 0.02073788 4.36292470 1.06053248
H 1.96691793 3.87622645 0.31002835
C -1.33676216 4.04168104 1.01883714
H -2.87290735 2.76230315 0.21298305
H 0.37054522 5.17682768 1.68316054
H -2.03537849 4.61221777 1.62143194
C 1.38154309 2.01364589 -1.63640830
N -0.76701900 -3.95525232 0.44111019
O -0.73188875 -4.36620994 1.57969270
O -1.24475138 -4.52886250 -0.51274303
*

B15
C -0.47871818 -2.97234556 0.00649377
C -0.33766097 -2.42410457 1.28440758
C 0.28450949 -1.21323766 1.45024017
C 0.78098924 -0.52175132 0.31988437
C 0.64070030 -1.07919187 -0.97376791
C 0.01741517 -2.28861642 -1.11397124
H 0.38377297 -0.80023443 2.44728659
H 1.02290024 -0.58609546 -1.85752724
C 1.42841487 0.70481333 0.53618791
H 1.55375174 1.00923887 1.57271506
H 2.78739445 2.18472306 -0.16976242
H 1.93773805 1.30402787 -1.47699203
C 0.65793886 2.60989076 -0.26384603
C -0.51538469 2.38834758 -0.98550719
C 0.72630839 3.65313620 0.66193459
C -1.59300730 3.24454985 -0.82797015
H -0.57988730 1.56110510 -1.68463590
C -0.35679301 4.50205690 0.82189178
H 1.63601852 3.81844130 1.23040555
C -1.51401133 4.29866815 0.07654149
H -2.49555802 3.09018307 -1.40709200
H -0.29795964 5.32617270 1.52271958
H -2.35990320 4.96440273 0.20280238
C 1.86894500 1.67681777 -0.45920182
F -0.81650048 -3.10124870 2.31638370
F -1.06832457 -4.12791828 -0.14810049
F -0.13367209 -2.85212134 -2.30288101
*

B16
C 0.15378533 -3.44727912 0.51160995
C 1.30790483 -2.85236794 1.04477374
C 1.64060215 -1.59158102 0.64554473
C 0.84101420 -0.87347271 -0.29880870
C -0.33627849 -1.50173837 -0.82940549
C -0.66928780 -2.75492380 -0.43490511
H 1.89901315 -3.41355801 1.75713454
H 2.52670181 -1.11119676 1.04498430
H -0.95578087 -0.97676220 -1.54648790
C 1.24222500 0.38926245 -0.65460233
H 2.15214969 0.76140769 -0.18816441
H 1.27547815 1.77312188 -2.24298124
H -0.27039693 0.92188629 -2.09385491
C 0.06980181 2.46330179 -0.59808260
C -1.03018828 2.23331501 0.22432904
C 0.73950110 3.68092672 -0.53178843
C -1.47445650 3.22394421 1.08844412
H -1.55031086 1.28069658 0.18401438
C 0.29607931 4.67038664 0.33531046
H 1.59681170 3.86545284 -1.17113547
C -0.81004638 4.44312127 1.14497597
H -2.33872352 3.04545722 1.71759595
H 0.81185720 5.62278702 0.37383027
H -1.15602514 5.21785589 1.81924564
C 0.55475021 1.35848796 -1.53377580
O -0.17235085 -4.65953579 0.88881019
H -0.98681559 -4.94467522 0.43991862
O -1.73880312 -3.48524148 -0.83814939
H -2.27240620 -3.02125886 -1.49295869
*

B17
C -0.02788626 -2.87312031 -0.52577615
C -0.01672164 -2.43858577 0.84941241
C 0.39756492 -1.17304892 1.12883274
C 0.81751499 -0.27671405 0.08397022
C 0.78935605 -0.73769419 -1.26616916
C 0.37778611 -2.00261273 -1.56310765
H 0.41852333 -0.81269795 2.14891519
H 1.09908711 -0.08002063 -2.06739768
H 0.36223203 -2.34419343 -2.58815044
C 1.23266316 0.98594135 0.41447130
H 1.21941446 1.24581832 1.47163513
H 2.58385468 2.50405670 -0.13089270
H 1.75734607 1.78458198 -1.51896259
C 0.54532113 3.15381516 -0.34767481
C -0.69433053 2.94610322 -0.94685312
C 0.76657462 4.30839315 0.39574955
C -1.69770033 3.89676148 -0.82357707
H -0.87569198 2.04246406 -1.52154009
C -0.23894594 5.25778260 0.52191265
H 1.73151064 4.47675538 0.86314685
C -1.47064265 5.05323266 -0.08729604
H -2.65667885 3.73603680 -1.30244181
H -0.05729365 6.16181980 1.09148217
H -2.25329396 5.79670161 0.00926197
C 1.63351392 2.09353516 -0.48265228
O -0.43208336 -4.09742011 -0.71337645
C -0.48332526 -4.65241195 -2.04513285

H	-1.17989168	-4.08218157	-2.66120143
H	0.51511259	-4.66211424	-2.48433744
H	-0.84473134	-5.66786003	-1.91197546
O	-0.42991714	-3.36254254	1.72515097
C	-0.44484002	-2.99588880	3.10637368
H	0.56324617	-2.74615977	3.44933649
H	-1.12309807	-2.15440507	3.27394132
H	-0.80726096	-3.87124947	3.63886439

*

B18

C	0.08522652	-3.04514493	0.00163017
C	1.10929515	-2.46840553	0.83330352
C	1.47763123	-1.18079035	0.66343934
C	0.87879455	-0.33534424	-0.33915755
C	-0.14856280	-0.92613790	-1.15947903
C	-0.53001661	-2.21107371	-1.00213253
H	1.58581653	-3.06556083	1.59624834
H	2.24980196	-0.75624048	1.29621735
H	-0.62946070	-0.32680204	-1.92201885
H	-1.30382995	-2.61786554	-1.63602387
C	1.29849740	0.95234162	-0.46403950
H	2.07545761	1.28572363	0.22155650
H	1.64062410	2.43391486	-1.91948342
H	0.09078177	1.61361843	-2.12091279
C	0.13747313	3.08065476	-0.52775591
C	-1.09482167	2.83144116	0.07100784
C	0.77102345	4.29928360	-0.31085462
C	-1.69580588	3.79779548	0.86495784
H	-1.59343192	1.87982406	-0.08974864
C	0.17117341	5.26607384	0.48620722
H	1.73020420	4.50200057	-0.77663836
C	-1.06188915	5.01681940	1.07438692
H	-2.66004470	3.60114201	1.31950697
H	0.66602946	6.21767346	0.64253548
H	-1.53079372	5.77298500	1.69329302
C	0.79047844	2.00336978	-1.38269256
N	-0.28359095	-4.30638060	0.15290127
C	0.33897862	-5.15690178	1.17561625
C	-1.32564178	-4.89827489	-0.69619154
H	-0.11027335	-6.14432990	1.12944463
H	1.41079930	-5.25371275	0.99445279
H	0.17102547	-4.74505546	2.17213927
H	-1.46160167	-5.93653708	-0.40897340
H	-2.27379178	-4.37437148	-0.56373497
H	-1.03100582	-4.86470703	-1.74634036

*

B19

C	0.06729735	-2.20925995	0.06307848
C	0.31187459	-1.42869401	1.18946965
C	0.76364343	-0.12474967	1.01987113
C	0.97857407	0.38977834	-0.25539166
C	0.75615876	-0.41065534	-1.36758045
C	0.28558811	-1.71507607	-1.21585028
H	0.93920197	0.48403285	1.89958649
H	0.95756312	-0.05336783	-2.36980157
C	1.48000100	1.78295572	-0.39254405
H	2.25214204	2.06443227	0.31399896
H	2.08718188	3.34875939	-1.78217153
H	0.83335680	2.18946453	-2.43830221
C	0.34756539	2.99995612	-0.41537633
C	-1.01266815	2.61191210	-0.54534703
C	0.67979207	4.13475640	0.37214271
C	-1.99508517	3.33873632	0.07727577
H	-1.26414202	1.73554022	-1.13209493
C	-0.30856456	4.85081788	1.00039779
H	1.72036990	4.42848493	0.46762797
C	-1.63990801	4.45089087	0.84941836
H	-3.03529218	3.05447684	-0.02066242
H	-0.06382152	5.71581380	1.60386281

H	-2.42013059	5.01667567	1.34819505
C	1.35177069	2.58301138	-1.57368983
N	-0.43362106	-3.58911567	0.23291797
O	0.22701660	-4.31039829	0.94147445
O	-1.45702199	-3.85487845	-0.35354584
C	0.07334470	-1.91093568	2.52520305
N	-0.12487355	-2.21783093	3.61346098
C	0.06927756	-2.51080816	-2.39618031
N	-0.06757425	-3.08907458	-3.37839921

*

B2

C	-0.23810449	-3.16439018	0.17132770
C	0.37755393	-2.63029245	1.30936829
C	0.93572235	-1.38125638	1.22831971
C	0.89226528	-0.63868401	0.01549920
C	0.26001494	-1.21638581	-1.12120988
C	-0.29754344	-2.46299793	-1.04449303
H	0.40318513	-3.20503522	2.22583973
H	1.41669314	-0.94490080	2.09708442
H	0.22349872	-0.67454118	-2.05754525
H	-0.77842873	-2.92067843	-1.89916862
C	1.48675982	0.61659247	-0.01109175
H	1.97529322	0.94310814	0.90509454
H	2.41971694	2.11275154	-1.18640714
H	1.13488781	1.22901354	-2.05012745
C	0.42725817	2.58994805	-0.49279960
C	-0.93386793	2.32860913	-0.63359834
C	0.85270366	3.70744096	0.22306409
C	-1.86654291	3.20652751	-0.10158256
H	-1.26823821	1.44835269	-1.17368439
C	-0.08224963	4.58015769	0.75905527
H	1.91279840	3.91010358	0.33747466
C	-1.44064942	4.33077154	0.59604999
H	-2.92509055	3.01358936	-0.22975886
H	0.24877413	5.45979961	1.29835227
H	-2.16940336	5.01553070	1.01389884
C	1.45423697	1.61564493	-1.08574337
Cl	-0.93479838	-4.71497566	0.25875112

*

B20

C	0.00502609	-1.64703659	-0.00680793
C	0.18177284	-0.93078661	1.17337614
C	0.70623519	0.34836400	1.10452466
C	1.03250598	0.91842184	-0.12230761
C	0.86258969	0.18167239	-1.28730006
C	0.35045605	-1.10718231	-1.23929543
H	0.85469062	0.90526237	2.02198896
H	1.13993995	0.58939964	-2.25040215
C	1.58119533	2.29412897	-0.14551912
H	2.22818656	2.54727568	0.68608075
H	2.40747128	3.89676743	-1.36299492
H	1.24999557	2.78971127	-2.24327346
C	0.47076563	3.56316477	-0.30537771
C	-0.85923312	3.19306770	-0.62810307
C	0.70181548	4.64242443	0.58471117
C	-1.91321754	3.88970350	-0.09196071
H	-1.03478664	2.35267882	-1.28999687
C	-0.35874051	5.32757290	1.12649673
H	1.72179795	4.92185502	0.82888510
C	-1.66002200	4.94981520	0.78509844
H	-2.93226234	3.61799737	-0.33720122
H	-0.19071947	6.15178604	1.80822552
H	-2.49689388	5.48958257	1.21590271
C	1.63192278	3.14512788	-1.29512940
C	-0.19826287	-1.48773916	2.54099045
F	0.47489036	-0.83867092	3.50655175
F	0.09064168	-2.78599210	2.64469397
F	-1.50372966	-1.31732934	2.77601659
C	0.20117983	-1.87756833	-2.54687933

F 0.16021730 -1.01828251 -3.58341232
F -0.92843222 -2.58998215 -2.57017507
F 1.23305001 -2.69870851 -2.74352312
N -0.56442780 -3.01558008 0.05346085
O -1.73379979 -3.08475346 0.34872609
O 0.19430968 -3.91907872 -0.19864337
*

B21
C 0.03012241 -2.38250686 0.08074631
C 0.53916626 -1.61601057 1.11835418
C 0.98636906 -0.32769244 0.89658377
C 0.92420957 0.19733601 -0.39250036
C 0.43442207 -0.56420574 -1.44991126
C -0.01243028 -1.84945071 -1.19647285
H 1.36975059 0.24583454 1.73201644
H 0.41648457 -0.20727907 -2.47145209
C 1.41744727 1.57760174 -0.60937983
H 2.26458308 1.86623235 0.00136061
H 1.83657772 3.14829690 -2.05469362
H 0.50364097 1.99648477 -2.54317142
C 0.27956187 2.82943714 -0.48957170
C -1.07826539 2.43117024 -0.41230684
C 0.72815840 3.94308314 0.26432438
C -1.95444785 3.13576916 0.37547333
H -1.41211894 1.56223361 -0.96773515
C -0.15219525 4.63577983 1.05976826
H 1.76929639 4.24337884 0.20192200
C -1.48871014 4.23082020 1.11026867
H -2.99509915 2.84285611 0.43603943
H 0.18250715 5.48699391 1.63918979
H -2.18252592 4.77705198 1.74076783
C 1.12743039 2.38937456 -1.75110434
N -0.45665246 -3.75034970 0.33319471
O 0.37490271 -4.56123455 0.66359917
O -1.64430239 -3.92406611 0.18563020
F -0.46930036 -2.59304499 -2.19549361
F 0.57482963 -2.12483430 2.34233200
*

B22
C 0.23987206 -2.36189380 -0.01882911
C 1.13708786 -1.76648387 0.93899476
C 1.47650297 -0.46589306 0.83050813
C 0.97670829 0.37460425 -0.23206602
C 0.07882052 -0.23881747 -1.18150327
C -0.27798055 -1.53523063 -1.08356122
H 1.52180124 -2.36195478 1.75442085
H 2.14395639 -0.02209657 1.56160662
H -0.31711907 0.35409557 -1.99616442
H -0.94236269 -1.96887265 -1.81729441
C 1.36193712 1.67589227 -0.28990893
H 2.03473148 2.02577376 0.49090988
H 1.83202081 3.18453231 -1.68124449
H 0.34730147 2.31828758 -2.07846905
C 0.14596967 3.77179853 -0.48743328
C -1.13934458 3.47514960 -0.04107469
C 0.70169588 5.01314994 -0.19862465
C -1.86760733 4.41579619 0.67305112
H -1.57798666 2.50558860 -0.25900991
C -0.02575020 5.95462213 0.51875844
H 1.70160689 5.25289372 -0.54612908
C -1.31012735 5.65744164 0.95490610
H -2.87135622 4.18161908 1.00885742
H 0.41047721 6.92375197 0.73183209
H -1.87828434 6.39364279 1.51163761
C 0.93633866 2.72261469 -1.25616777
N -0.10024350 -3.62825119 0.07308947
C -1.07082517 -4.30186018 -0.81725331
C 0.44080433 -4.56619026 1.08091019
H -1.96689148 -3.69133273 -0.92647106
H -0.61500603 -4.44661410 -1.80085488
C -1.33850837 -5.63150038 -0.11703015
H 1.52490350 -4.47074286 1.13791085
H 0.00987257 -4.33033666 2.05810155
C -0.01106410 -5.93462517 0.57817879
H -2.13579536 -5.51068965 0.62029075
H -1.64110046 -6.40509200 -0.82159572
H 0.71397358 -6.32596476 -0.13948204
H -0.10604308 -6.65266088 1.39167783
*

B23
C -0.17990690 -2.85748295 -0.09990691
C 1.10179802 -2.30372506 0.28051525
C 1.44558093 -1.03287868 0.01416409
C 0.55007908 -0.14287046 -0.67929263
C -0.71475839 -0.68950768 -1.08994015
C -1.05336538 -1.97102666 -0.83090149
H 2.43091914 -0.68949484 0.30889105
H -1.42369457 -0.05848318 -1.60993217
H -2.02453283 -2.33124395 -1.13462479
C 0.93371804 1.13981050 -0.92272795
H 1.91461007 1.43909027 -0.55828644
H 0.76418100 2.64368969 -2.38439182
H -0.77834955 1.87406910 -2.00616393
C -0.09155838 3.29917663 -0.52556774
C -1.04383910 3.08290763 0.46710609
C 0.64458087 4.47893032 -0.52487168
C -1.27023758 4.04472908 1.44133144
H -1.61935371 2.16163072 0.47494930
C 0.42066510 5.44065527 0.45206778
H 1.38609853 4.65588422 -1.29750766
C -0.53621410 5.22487546 1.43526967
H -2.02071179 3.87503744 2.20486784
H 0.99067452 6.36248591 0.44077182
H -0.71290476 5.97740867 2.19496840
C 0.15827255 2.22537590 -1.57540143
N -0.55378672 -4.09267125 0.18043340
C 0.12312957 -4.97371246 1.15070582
C -1.76089883 -4.66888318 -0.42882739
H -0.63023038 -5.64829580 1.55304057
H 0.90667446 -5.55545412 0.66380327
H 0.54403176 -4.39848189 1.96780370
H -1.68200110 -5.75178162 -0.36754144
H -2.65855784 -4.34685645 0.10468215
H -1.83111938 -4.39301944 -1.47924161
F 2.00416066 -3.10981018 0.86416159
*

B24
C -0.20290096 -2.11598020 -0.01356231
C -0.06521058 -1.43267442 1.19281722
C 0.52441903 -0.18529366 1.22817393
C 0.98047373 0.40394211 0.04455720
C 0.85694561 -0.28144088 -1.15841476
C 0.26260582 -1.53661800 -1.18098405
H 0.62729857 0.33595104 2.17278048
H 1.23213016 0.13831610 -2.08426894
C 1.60982654 1.73134594 0.12051225
H 2.08720458 1.96679714 1.06432767
H 2.71097901 3.29535512 -0.90315223
H 1.66843516 2.26434457 -1.98852473
C 0.59984402 3.12058384 -0.24183909
C -0.67609509 2.81084440 -0.76480448
C 0.73471960 4.14802697 0.71961459
C -1.77462122 3.52908472 -0.35756888
H -0.78238977 2.00059104 -1.47690717
C -0.37204362 4.85351077 1.13316806
H 1.71642356 4.37899721 1.12066596
C -1.62059420 4.54346755 0.59179878
H -2.75449282 3.30529993 -0.76042741

H -0.27627587 5.64250771 1.86850679
H -2.49319702 5.09770192 0.92063642
C 1.88427345 2.60080724 -0.98267726
H -0.66468708 -3.09578604 -0.03630399
C -0.59293719 -2.07968152 2.45652331
F -0.26566806 -1.37192318 3.54863019
F -0.10339722 -3.31808930 2.60167073
F -1.93234693 -2.17308084 2.41439836
C 0.16001815 -2.25799988 -2.50830507
F -0.34295397 -1.43634076 -3.45125463
F -0.62811056 -3.33354021 -2.43437606
F 1.37112816 -2.65386643 -2.93080192
*

B3
C -0.85692603 -2.46911995 -1.16279377
C -0.07812832 -2.27672176 -0.02055084
C 0.64682266 -1.11764748 0.12503622
C 0.59490775 -0.12280250 -0.87977188
C -0.19725122 -0.33553703 -2.03196534
C -0.91426785 -1.50324435 -2.16737265
H 1.25451446 -0.95991716 1.00845942
H -0.23156067 0.41022380 -2.81609151
H -1.51735294 -1.67848945 -3.04937770
C 1.37342289 1.03435080 -0.69768630
H 2.01763961 1.05032812 0.17877148
H 2.33766219 2.73013143 -1.55596941
H 0.88454646 2.19173454 -2.45678759
C 0.47956531 3.02468426 -0.46690180
C -0.91077017 2.93708824 -0.54424917
C 1.08056868 3.77044933 0.54965151
C -1.69633212 3.64391310 0.35137935
H -1.37835329 2.33232920 -1.31420585
C 0.29087132 4.46913084 1.44810698
H 2.16236936 3.82803529 0.61623143
C -1.09556082 4.40712974 1.34750378
H -2.77620925 3.59781686 0.27725009
H 0.75473135 5.06644569 2.22387803
H -1.71148763 4.95443137 2.05165241
C 1.35686136 2.26392342 -1.48169208
H -1.42238912 -3.38916302 -1.26957276
C -0.05343510 -3.36233367 1.03681145
F 0.72959616 -3.02411513 2.06940075
F 0.40521018 -4.51161359 0.52178241
F -1.28933913 -3.58869799 1.50544478
*

B4
C -0.03598319 -2.42483866 0.13338203
C 0.51466424 -1.83863426 1.26934231
C 1.05384120 -0.57322545 1.17385303
C 1.04060142 0.11759223 -0.05819312
C 0.48133772 -0.50371353 -1.19820981
C -0.05081754 -1.76812570 -1.09949584
H 0.51278068 -2.37097511 2.21185746
H 1.48624313 -0.09767973 2.04737195
H 0.47986850 0.00293592 -2.15478353
H -0.48052526 -2.25569421 -1.96521772
C 1.62645299 1.39916960 -0.10520208
H 2.12029988 1.73890944 0.80245243
H 2.47735057 2.97114679 -1.27046678
H 1.27226224 1.98081016 -2.15682995
C 0.44845116 3.18447144 -0.51496634
C -0.88692585 2.83848906 -0.72739636
C 0.77284262 4.24709603 0.33242305
C -1.89372296 3.59308757 -0.14855127
H -1.13871461 1.99234049 -1.35790321
C -0.23748979 4.99383397 0.91529896
H 1.81216595 4.50672539 0.50676065
C -1.56866718 4.66801898 0.67309085
H -2.93151513 3.34258923 -0.33276317
H 0.01064105 5.83298700 1.55402883
H -2.35774151 5.25356873 1.13053899
C 1.57337962 2.37079092 -1.18946830
C -0.59293995 -3.83825963 0.22540062
F -1.56623695 -4.03357829 -0.67144574
F -1.08835764 -4.08346979 1.44318680
F 0.38359951 -4.72389199 -0.01637023
*

B5
C -0.38166636 -3.68816661 0.14893845
C 0.28370878 -3.18109973 1.28690743
C 0.83756514 -1.94107998 1.22394595
C 0.75908604 -1.14738330 0.03237939
C 0.07631727 -1.69438140 -1.10086464
C -0.48094157 -2.93347709 -1.04652457
H 0.33311796 -3.79502827 2.17714347
H 1.35149740 -1.53207443 2.08716405
H 0.00340888 -1.11887189 -2.01469869
H -0.99661669 -3.35247496 -1.90394354
C 1.34311479 0.09616295 0.02481775
H 1.84224792 0.40711345 0.94095373
H 2.31786412 1.52311394 -1.18275188
H 0.94495106 0.74585486 -1.99229079
C 0.40184275 2.21040893 -0.47646897
C -0.97859142 2.02907040 -0.49311507
C 0.94208451 3.36169138 0.08849038
C -1.81597835 3.00674111 0.02494104
H -1.40480449 1.12791067 -0.92406178
C 0.10349045 4.33700584 0.61028733
H 2.01744234 3.50776742 0.10274291
C -1.27460773 4.16061943 0.57854521
H -2.89058109 2.86899370 -0.00358775
H 0.52676857 5.23935806 1.03583745
H -1.92769172 4.92482343 0.98354311
C 1.31275301 1.11985994 -1.03891934
O -0.90100384 -4.89487660 0.26038331
H -1.33896535 -5.18017673 -0.55387625
*

B6
C 0.00073948 -3.30729632 0.27819433
C 0.85357881 -2.72447408 1.25293440
C 1.29626832 -1.45537412 1.07726236
C 0.92061112 -0.68778215 -0.07758354
C 0.05895819 -1.30387533 -1.04122592
C -0.39274381 -2.57640047 -0.87552883
H 1.12565304 -3.31949027 2.11544872
H 1.94653145 -0.99846873 1.81541985
H -0.24028906 -0.74987941 -1.92192489
H -1.04077624 -3.02528335 -1.61467914
C 1.39848347 0.58991451 -0.21177712
H 2.04793681 0.95461310 0.58224548
H 2.01665660 2.03400293 -1.61550499
H 0.55369776 1.16618823 -2.10974122
C 0.24962169 2.65015529 -0.55063770
C -1.08976878 2.39519490 -0.26841359
C 0.82318824 3.85277381 -0.15021229
C -1.85815836 3.34726651 0.38623539
H -1.54022375 1.45435193 -0.57092383
C 0.05474018 4.80318119 0.50841539
H 1.86639513 4.05699474 -0.36920382
C -1.28517670 4.55160807 0.77656933
H -2.90396503 3.15029895 0.59158555
H 0.50177655 5.74441174 0.80643400
H -1.88430264 5.29600879 1.28792271
C 1.08568118 1.58667675 -1.25843628
O -0.37717329 -4.53421962 0.52713184
C -1.24365198 -5.23413498 -0.39264704
H -2.19670846 -4.71092607 -0.48011003
H -0.75642261 -5.33265015 -1.36352701

H -1.39388721 -6.21364851 0.05188119
*

B7
C -0.06736139 -3.33882216 0.29102271
C 0.76286033 -2.74493907 1.26389219
C 1.24738874 -1.47915283 1.08466455
C 0.92264106 -0.73509897 -0.08718388
C 0.08027245 -1.34606109 -1.06503884
C -0.39748666 -2.60875463 -0.87643871
H 1.01324324 -3.30527497 2.15673853
H 1.88496571 -1.02413092 1.83513060
H -0.17967053 -0.80529186 -1.96609923
H -1.03426175 -3.05360600 -1.62897004
C 1.44257974 0.53645392 -0.22694349
H 2.08549511 0.89195387 0.57642261
H 2.09272949 2.01010943 -1.59391680
H 0.66629569 1.10986125 -2.15432644
C 0.27774777 2.53990499 -0.55528693
C -1.07185410 2.25740542 -0.36007336
C 0.82031783 3.71872748 -0.05087391
C -1.88331988 3.16601234 0.30400727
H -1.49557625 1.33229028 -0.73887977
C 0.00811088 4.62390968 0.61722196
H 1.87205416 3.94120064 -0.20000927
C -1.34291976 4.34868537 0.79424278
H -2.93689999 2.95139667 0.43949577
H 0.42886950 5.54881745 0.99396814
H -1.97597190 5.05843528 1.31392318
C 1.16500994 1.52626294 -1.28210510
C -0.55332671 -4.69456092 0.53445784
C -1.30911156 -5.40175674 -0.30650414
H -0.25471641 -5.13732470 1.47935504
H -1.62427338 -6.40592452 -0.04877198
H -1.63795441 -5.02508858 -1.26790527
*

B8
C -0.18408575 -3.34700615 0.12421077
C 0.42471797 -2.80088869 1.26906151
C 0.96230081 -1.54249162 1.20960794
C 0.91115135 -0.78482054 0.00589010
C 0.29318299 -1.35765618 -1.14162393
C -0.24285639 -2.61372029 -1.08110754
H 0.45927984 -3.38279058 2.18093587
H 1.43261932 -1.10796881 2.08509411
H 0.25158005 -0.80263842 -2.06995856
H -0.71206394 -3.06267361 -1.94709548
C 1.48287499 0.48019277 -0.00075652
H 1.95735984 0.80500953 0.92345742
H 2.40482989 1.99748328 -1.15518984
H 1.13708501 1.10750624 -2.03555938
C 0.40282925 2.45508169 -0.48054889
C -0.95422122 2.17288923 -0.61929661
C 0.81113250 3.58431908 0.22628895
C -1.90005293 3.04029795 -0.09292598
H -1.27534048 1.28349459 -1.15243251
C -0.13675746 4.44683217 0.75639416
H 1.86806382 3.80366027 0.33901531
C -1.49128476 4.17594184 0.59618614
H -2.95558451 2.82999080 -0.21888205
H 0.18130165 5.33530760 1.28904014
H -2.23012681 4.85265316 1.00942899
C 1.44283096 1.49108834 -1.06547174
C -0.74498819 -4.65424426 0.17783397
C -1.21756784 -5.75559864 0.22204767
H -1.63797146 -6.73712591 0.26208428
*

B9
C -0.28978832 -3.18597158 0.20925627
C 0.31457990 -2.60531434 1.32748369
C 0.90988909 -1.36976102 1.19703258
C 0.90434789 -0.69910436 -0.04582222
C 0.29292096 -1.30978776 -1.16445335
C -0.29810509 -2.54379093 -1.03780590
H 0.30995226 -3.12745353 2.27575626
H 1.38355233 -0.90262745 2.05346332
H 0.29827664 -0.81935008 -2.12941239
H -0.76447901 -3.02889006 -1.88594245
C 1.55309452 0.55268854 -0.12651751
H 2.08485236 0.87943780 0.76410565
H 2.45138706 2.06876927 -1.33243826
H 1.18571672 1.11998684 -2.18078424
C 0.44941887 2.36986951 -0.53077369
C -0.90437643 2.08704070 -0.72344206
C 0.83624754 3.41574255 0.31226401
C -1.86532932 2.88869811 -0.13116601
H -1.20496353 1.25393479 -1.34984746
C -0.12891318 4.20948570 0.90892290
H 1.88918896 3.62546418 0.47154836
C -1.47734357 3.94703992 0.68535426
H -2.91645544 2.68842290 -0.30053473
H 0.16757437 5.03576221 1.54376368
H -2.23122146 4.56928544 1.15364485
C 1.52560337 1.50745503 -1.22544863
C -0.91518986 -4.47633913 0.33602056
N -1.41746468 -5.50485077 0.43550529
*

C0
N 0.15945808 1.05541705 -1.47912925
O 1.43329686 0.91216751 -1.25100117
C -0.50732238 2.08384136 -1.07846558
C -0.47569427 -0.05342427 -2.19424396
H -1.57293378 2.09609411 -1.31696198
C 0.15172922 3.16440523 -0.30305891
H 0.07628046 -0.21578363 -3.12970851
H -0.40137212 -0.94773015 -1.56080117
H -1.52759768 0.17820315 -2.40040065
H 1.01785831 3.56305604 -0.85637813
H -0.55643974 3.97600760 -0.09058796
H 0.53626796 2.75472743 0.64660037
C -0.25293552 0.20651163 1.62259043
C -1.39984163 -0.14021619 1.32448734
O 0.91157794 0.53691548 1.90925105
Li 2.07770829 0.20133670 0.41376555
O 2.16029823 -1.73890209 0.23973371
O 3.91200392 0.86302469 0.56029287
C 1.74978216 -2.61122889 1.27530410
H 0.76532655 -3.05763423 1.04590947
H 2.48749281 -3.42139760 1.42052240
H 1.67038629 -2.01881539 2.19632519
C 2.31701902 -2.37284059 -1.01340284
H 1.37746117 -2.86203489 -1.32934054
H 2.58372495 -1.60198962 -1.74898163
H 3.11629537 -3.13538843 -0.97151397
C 4.91966519 0.18158428 -0.16269223
H 5.92296089 0.46342484 0.20465837
H 4.77080328 -0.89592366 -0.00904202
H 4.85721908 0.40994176 -1.24248170
C 4.01695897 2.27268013 0.47480922
H 3.94413860 2.61279074 -0.57338394
H 3.19107749 2.70617562 1.05567280
H 4.97630461 2.61808026 0.90074734
C -2.71420558 -0.40373180 0.80935523
C -3.69032755 0.61651980 0.73799285
C -3.07490796 -1.68171058 0.32534833
C -4.95654174 0.37058322 0.20661594
H -3.43841734 1.61477903 1.10804560

C -4.34200948 -1.92293969 -0.20577374
H -2.33933846 -2.49040193 0.36943097
C -5.29285636 -0.89991824 -0.27002031
H -5.68990794 1.18237900 0.16444877
H -4.58995252 -2.92355401 -0.57437505
H -6.28608263 -1.09113175 -0.68687866
*

C1
N -1.65398067 -0.88948271 -1.27452320
O -0.39308214 -0.77096874 -1.58494049
C -2.46859305 0.11026413 -1.27940631
C -2.07421965 -2.23864598 -0.89091405
H -3.50212775 -0.10083533 -0.99792227
C -1.99831160 1.47478925 -1.62927655
H -3.14931141 -2.25556577 -0.67450345
H -1.83496118 -2.91856839 -1.71966903
H -1.49894956 -2.52132934 0.00107889
H -1.51540873 1.47465320 -2.62031982
H -2.83546400 2.18472585 -1.62429225
H -1.24049879 1.80520143 -0.90024525
C -1.36892644 0.35783093 1.82646695
C -2.27774413 1.13177434 2.13473423
H -3.07669742 1.78974594 2.42416708
O -0.44540134 -0.41759485 1.51046988
Li 0.62437266 -0.25043206 -0.03764007
O 2.21073293 -1.38438063 0.10072847
O 1.24527640 1.58716737 -0.13499996
C 3.05941502 -1.08259960 1.19217818
H 2.62865587 -1.44577239 2.14268948
H 4.05616899 -1.53854625 1.05175380
H 3.16709180 0.01025435 1.23902791
C 1.96609571 -2.76999880 -0.05631787
H 1.48419422 -3.19185605 0.84466889
H 1.29564357 -2.89036513 -0.91811274
H 2.90862192 -3.31494827 -0.24594118
C 1.67426710 2.14160642 -1.36196261
H 2.72810409 2.46927977 -1.29841003
H 1.58317120 1.36263589 -2.13167122
H 1.04889063 3.00712508 -1.64733199
C 1.26887253 2.49362553 0.95015621
H 0.58796328 3.34492192 0.76793254
H 0.93364910 1.95342544 1.84531388
H 2.29039302 2.88248987 1.11515683
*

C14
N 1.32691861 1.28489542 -1.50880307
O 2.54703474 0.85370436 -1.36144314
C 0.93557697 2.43063277 -1.06310983
C 0.41918239 0.36489340 -2.19971233
H -0.11139307 2.68747219 -1.23815665
C 1.86805739 3.32051299 -0.32677162
H 0.84961287 0.13669533 -3.18459892
H 0.35746451 -0.55454134 -1.60310060
H -0.57381534 0.81868887 -2.30541989
H 2.77912363 3.50014455 -0.92067612
H 1.38492902 4.27715864 -0.08853191
H 2.18501331 2.82793488 0.60848743
C 0.65351269 0.37881768 1.52785879
C -0.55778943 0.23618325 1.32704110
O 1.86706516 0.50560534 1.71633799
Li 2.96967200 -0.08394392 0.25229318
O 2.53011229 -1.96080017 -0.00553382
O 4.89978286 0.09269201 0.47076452
C 1.93706943 -2.76913506 0.99399093
H 0.88580307 -2.99661401 0.74180468
H 2.49294294 -3.71588150 1.11248197
H 1.96696922 -2.20980201 1.93792233
C 2.59200213 -2.57382198 -1.27767661
H 1.58343324 -2.85697716 -1.62879319
H 3.02534027 -1.84795586 -1.97956079
H 3.22328493 -3.47965192 -1.24734768
C 5.72955464 -0.78039314 -0.27292741
H 6.75859744 -0.77854952 0.12891082
H 5.31287341 -1.79324225 -0.18652585
H 5.76056869 -0.48658407 -1.33816396
C 5.35790361 1.43326473 0.47538111
H 5.40949391 1.83819798 -0.55085294
H 4.64744355 2.02872217 1.06547189
H 6.35758547 1.50044643 0.94109205
C -1.92282858 0.06977565 0.97357254
C -2.93162181 0.94529035 1.46344885
C -2.32485492 -0.97600617 0.10058894
C -4.26072482 0.79104498 1.10011903
H -2.64894845 1.75794694 2.13883945
C -3.64994212 -1.13944657 -0.26766101
H -1.56813586 -1.66706312 -0.28637537
C -4.61303565 -0.25179572 0.23382310
H -3.94626097 -1.94799371 -0.93973582
H -5.02749860 1.47192972 1.48139398
N -6.00745357 -0.41795724 -0.15495845
O -6.29023002 -1.33339068 -0.91552304
O -6.83509221 0.36523451 0.29403834
*

C15
N 1.30552781 1.21089406 -1.69546017
O 2.55693756 0.94739527 -1.44708062
C 0.76586282 2.34760715 -1.41149537
C 0.54387302 0.11339104 -2.29661533
H -0.29196378 2.45948862 -1.65894594
C 1.55461358 3.42075669 -0.75699675
H 1.04695498 -0.18065840 -3.22746587
H 0.55483962 -0.72608741 -1.58898636
H -0.48729990 0.42975797 -2.49472156
H 2.43386362 3.68186780 -1.36884582
H 0.93756725 4.31514389 -0.60038752
H 1.93138660 3.05724590 0.21375289
C 0.38233032 0.51683964 1.34619382
C -0.79692535 0.21572429 1.13946766
O 1.56859377 0.81392885 1.55339951
Li 2.86824289 0.19458334 0.29864632
O 2.65440535 -1.73324329 0.13882087
O 4.71477349 0.62597237 0.75838558
C 1.99063683 -2.52620884 1.10501698
H 1.00839708 -2.86525657 0.72899031
H 2.59880495 -3.41002681 1.37037840
H 1.83979114 -1.90750923 1.99967697
C 2.95813341 -2.42416491 -1.05697765
H 2.04327045 -2.84138353 -1.51617161
H 3.41240589 -1.70618803 -1.75327623
H 3.66633772 -3.25068639 -0.86410586
C 5.70116008 0.00715638 -0.04679673
H 6.70699314 0.15277093 0.38617172
H 5.48014957 -1.06860956 -0.07993693
H 5.68990107 0.41520777 -1.07377712
C 4.92580427 2.01574660 0.93503551
H 4.91853014 2.54478417 -0.03444948
H 4.11168189 2.39821154 1.56599653
H 5.89095177 2.20163292 1.43952674
C -2.14255874 -0.15098245 0.82239005
C -3.24051648 0.47636739 1.45381753
C -2.41378902 -1.15209333 -0.13847721
C -4.53712788 0.10946301 1.12755954
H -3.08760547 1.25492967 2.20379170
C -3.72241679 -1.49507351 -0.44147715
H -1.60426162 -1.66973900 -0.65652557
C -4.80428692 -0.87617432 0.17994875
F -3.97647968 -2.44407594 -1.35340248
F -6.06261374 -1.21835892 -0.12403285
F -5.57786910 0.70503311 1.72558975

*

C18

N	1.36859104	1.26271648	-1.49372229
O	2.61645200	0.92162790	-1.34163417
C	0.87589506	2.34193046	-0.98791419
C	0.54837053	0.31336366	-2.24876206
H	-0.18488012	2.52439344	-1.17219042
C	1.71520631	3.25028072	-0.16761644
H	1.02276038	0.15302618	-3.22623604
H	0.52765585	-0.63090205	-1.68763362
H	-0.46960281	0.70340182	-2.36953438
H	2.62301165	3.54295454	-0.71991608
H	1.15054702	4.14682074	0.12019005
H	2.04618766	2.71685900	0.74028445
C	0.75095663	0.22255755	1.53472548
C	-0.44988854	0.06022028	1.30482792
O	1.97095929	0.37325249	1.75543759
Li	3.08393969	-0.03741673	0.25994337
O	2.81978788	-1.93574343	-0.11704184
O	5.00233540	0.26809487	0.49259192
C	2.25704781	-2.82897469	0.82461196
H	1.23350144	-3.11983261	0.52656067
H	2.87675895	-3.73929492	0.92013156
H	2.21681667	-2.31010051	1.79142722
C	2.94542829	-2.47696601	-1.41597109
H	1.96439877	-2.80706907	-1.80390915
H	3.34191626	-1.68903920	-2.07052107
H	3.63491200	-3.34117776	-1.41834649
C	5.89826003	-0.48506810	-0.30221743
H	6.92596835	-0.42045435	0.09864563
H	5.56910051	-1.53284917	-0.27647148
H	5.89953223	-0.12692236	-1.34808496
C	5.34423091	1.63976317	0.57609565
H	5.35766838	2.10694938	-0.42458896
H	4.58723465	2.13647713	1.19863207
H	6.33612032	1.76595365	1.04658445
C	-1.84123090	-0.03489275	0.94018215
C	-2.64514967	1.11018400	0.76464813
C	-2.47801939	-1.27396946	0.72592375
C	-3.98637908	1.03076597	0.39443713
H	-2.20348688	2.09750616	0.93206615
C	-3.81786938	-1.36994689	0.35576400
H	-1.90223000	-2.19509579	0.85896761
C	-4.61227601	-0.21593870	0.16526016
H	-4.24458888	-2.36367071	0.21203173
H	-4.54774479	1.95961507	0.28279212
N	-5.94089719	-0.30360927	-0.22799638
C	-6.76930399	0.88327605	-0.18299619
C	-6.59506120	-1.59545613	-0.21374692
H	-7.77488340	0.63439651	-0.54796401
H	-6.86742002	1.30309277	0.83819983
H	-6.37033445	1.67756788	-0.83548816
H	-7.63077070	-1.47868414	-0.56017560
H	-6.09861096	-2.30628153	-0.89512338
H	-6.61934557	-2.05537352	0.79436803

*

C19

N	1.94394213	0.95316460	-1.76439682
O	3.14533830	0.54150959	-1.47787856
C	1.57319908	2.17882858	-1.60651245
C	1.03555591	-0.07911963	-2.27013039
H	0.54117991	2.41274412	-1.87659494
C	2.50878744	3.19460160	-1.06233005
H	1.48967430	-0.52156281	-3.16695766
H	0.93366100	-0.84726023	-1.49202837
H	0.05765218	0.35715846	-2.50735831
H	3.44736827	3.20248701	-1.63993374
H	2.05151919	4.19243504	-1.08189110
H	2.77193660	2.93506384	-0.02282270
C	1.15781176	0.72242744	1.37567498
C	-0.05133925	0.53182370	1.18578625
O	2.35751916	0.91384425	1.55956254
Li	3.53158434	0.00299779	0.31291393
O	3.08866375	-1.87895232	0.49935188
O	5.45036542	0.21649533	0.56068690
C	2.54580357	-2.44857674	1.67544734
H	1.49988856	-2.76542678	1.51242342
H	3.14035684	-3.32247155	1.99776422
H	2.57194874	-1.68229163	2.46169400
C	3.13919891	-2.76958859	-0.59801995
H	2.12946196	-3.14188855	-0.85032140
H	3.54491006	-2.21885535	-1.45748891
H	3.78928206	-3.63472780	-0.37351780
C	6.28065997	-0.82160676	0.07186851
H	7.29929253	-0.73652051	0.49069639
H	5.84155036	-1.77779201	0.38746027
H	6.34259489	-0.79311786	-1.03133692
C	5.93893857	1.51249351	0.26136492
H	6.02439259	1.66158582	-0.82954747
H	5.22889766	2.24261097	0.67390488
H	6.92844575	1.67151418	0.72607516
C	-1.40243556	0.28456016	0.86141559
C	-2.42810933	1.20342867	1.19099626
C	-1.79053598	-0.89442505	0.17905515
C	-3.76165359	0.96109153	0.86579704
H	-2.17119803	2.12896375	1.70904545
C	-3.11717719	-1.13732208	-0.17117514
H	-1.03350337	-1.63646625	-0.07918336
C	-4.11627943	-0.21097732	0.17508267
N	-5.50812302	-0.46399236	-0.18035706
O	-5.72738686	-1.11837293	-1.18217175
O	-6.37121627	-0.00651515	0.54416235
C	-4.71878981	1.98612453	1.22082698
N	-5.40808941	2.86406761	1.52275188
C	-3.39698731	-2.38739023	-0.84362780
N	-3.52630719	-3.41395345	-1.35996825

*

C2

N	1.11299886	1.21197342	-1.60582777
O	2.36840029	0.94630220	-1.38271695
C	0.56252309	2.32158101	-1.24583568
C	0.35984124	0.14819357	-2.27423340
H	-0.49863582	2.43548733	-1.47743058
C	1.34106376	3.35939281	-0.52521757
H	0.86336281	-0.08105621	-3.22301517
H	0.37924408	-0.73564750	-1.62276658
H	-0.67468022	0.46678535	-2.45118458
H	2.25328250	3.61838013	-1.08696086
H	0.73363990	4.26018647	-0.36737855
H	1.66411831	2.95874145	0.45089534
C	0.39009717	0.41848550	1.46981394
C	-0.78179276	0.10624025	1.23910937
O	1.57291410	0.72681202	1.69459104
Li	2.80931843	0.19686385	0.33080964
O	2.64750866	-1.73871085	0.18987634
O	4.69203631	0.63188097	0.62740999
C	2.17903277	-2.54680421	1.25246850
H	1.18855714	-2.97465543	1.01410653
H	2.88664920	-3.37023508	1.45951571
H	2.09087253	-1.90914526	2.14206012
C	2.82594815	-2.43659095	-1.02579441
H	1.87919869	-2.90131065	-1.35720564
H	3.15480566	-1.71163620	-1.78266692
H	3.58908782	-3.22905640	-0.91749719
C	5.64697420	-0.15737858	-0.05713057
H	6.65694074	-0.00063179	0.36262370
H	5.36459718	-1.21112439	0.07185613
H	5.66548348	0.08733318	-1.13493129
C	4.96985348	2.01971393	0.57121760

H 4.99335161 2.37731350 -0.47346098
H 4.17307494 2.54372545 1.11702483
H 5.94025975 2.24206951 1.05052722
C -2.12966713 -0.22373674 0.87917783
C -3.21871396 0.58658520 1.27268049
C -2.42407696 -1.36825422 0.10322125
C -4.52831121 0.27640200 0.91175049
H -3.02673126 1.47900832 1.87450143
C -3.73001045 -1.68718602 -0.26337858
H -1.60585859 -2.01948672 -0.21631734
C -4.77783638 -0.86088532 0.14329517
H -3.93655152 -2.57660509 -0.86414384
H -5.35668580 0.91671332 1.22546380
Cl -6.41598403 -1.25429647 -0.31323377
*

C21
N 1.63909628 1.05049440 -1.54963974
O 2.87484285 0.69213208 -1.34979110
C 1.18952538 2.21119351 -1.21097409
C 0.78402778 0.03272947 -2.16547392
H 0.13482379 2.40310178 -1.41997689
C 2.06929334 3.20350686 -0.54431835
H 1.24907434 -0.27494678 -3.11162043
H 0.73613475 -0.82500011 -1.48109491
H -0.22041881 0.43643567 -2.34124762
H 2.98212909 3.37055661 -1.13899957
H 1.54100120 4.15483555 -0.39915040
H 2.39155796 2.81125880 0.43537863
C 1.01285649 0.39309150 1.55327411
C -0.19646527 0.23753001 1.34195729
O 2.22011708 0.53771018 1.75452551
Li 3.34681219 -0.11204068 0.32412225
O 2.98314293 -2.01671441 0.19717291
O 5.26119694 0.17364673 0.53075089
C 2.39235083 -2.77676916 1.23450190
H 1.35936403 -3.06626914 0.96943041
H 2.98174485 -3.68959512 1.43559286
H 2.37151961 -2.15138001 2.13681264
C 3.10120197 -2.71441431 -1.02710800
H 2.11267913 -3.05682589 -1.38373971
H 3.52984157 -2.02454721 -1.76662944
H 3.76174757 -3.59358291 -0.91638760
C 6.14128593 -0.70376677 -0.14738793
H 7.16650439 -0.61639249 0.25486908
H 5.78060119 -1.72927643 0.01144503
H 6.16186357 -0.48557961 -1.23076412
C 5.64383408 1.53474373 0.43724865
H 5.67710355 1.86548239 -0.61584414
H 4.89835555 2.13056712 0.98194065
H 6.63596146 1.69202870 0.89713631
C -1.55295624 0.11867367 0.95131115
C -2.43257205 1.22827334 1.01723615
C -2.06577964 -1.10921097 0.46230545
C -3.74784179 1.10226544 0.61779542
H -2.08418310 2.19903532 1.37383894
C -3.38038279 -1.20478632 0.05344356
H -1.43517436 -1.99805500 0.40644385
C -4.25834891 -0.10964255 0.12243498
N -5.63866815 -0.22484930 -0.30394110
O -5.87641056 -0.89260017 -1.29479709
O -6.48889284 0.35202045 0.35007337
F -3.83537336 -2.38008444 -0.37935456
F -4.53886435 2.17338657 0.66250969
*

C6
N 1.13967918 1.24213112 -1.65798724
O 2.39224787 0.98479691 -1.40917769
C 0.57615387 2.35082036 -1.31622622
C 0.40431939 0.16817071 -2.32958400
H -0.48114913 2.45677993 -1.56821026
C 1.33446194 3.39786722 -0.58747270
H 0.92898940 -0.07141159 -3.26423617
H 0.41191879 -0.70739394 -1.66680563
H -0.62728454 0.48085833 -2.53221111
H 2.25192259 3.66463026 -1.13700638
H 0.71628110 4.29323394 -0.44079246
H 1.64695259 3.00165728 0.39391970
C 0.36424558 0.50231165 1.42128178
C -0.80337868 0.19435128 1.16891427
O 1.54742728 0.80770102 1.66894432
Li 2.79464461 0.26069226 0.32653101
O 2.64024763 -1.67934563 0.20672897
O 4.67613211 0.69890273 0.64362191
C 2.13484844 -2.46790163 1.26703206
H 1.14600452 -2.88770868 1.00840298
H 2.82720657 -3.29651889 1.50316734
H 2.03071448 -1.81694899 2.14514475
C 2.83971843 -2.39486684 -0.99504834
H 1.89605858 -2.85349167 -1.34339486
H 3.19479488 -1.68383123 -1.75325500
H 3.59102289 -3.19431454 -0.85839459
C 5.64121835 -0.09900513 -0.01565289
H 6.64504892 0.06467931 0.41620578
H 5.35784869 -1.15106346 0.12446629
H 5.67536337 0.13012114 -1.09653198
C 4.95223756 2.08601701 0.56947991
H 4.98667120 2.42787286 -0.48016693
H 4.14845995 2.61664838 1.09833069
H 5.91688389 2.31753734 1.05617110
C -2.14903948 -0.13819109 0.78222105
C -3.24731446 0.66258279 1.14642065
C -2.43133841 -1.28544185 0.00268434
C -4.55635655 0.35155845 0.76239593
H -3.07317697 1.55837437 1.74956218
C -3.72659115 -1.60471415 -0.38592075
H -1.60693884 -1.93750415 -0.30052180
C -4.80385671 -0.78817267 -0.00954102
H -3.92526184 -2.49559911 -0.98845979
H -5.36982183 1.00935809 1.07350504
O -6.04010085 -1.17618811 -0.43440755
C -7.15444087 -0.38397274 -0.06966102
H -7.07142893 0.64059411 -0.47218724
H -8.03892088 -0.86860445 -0.50489388
H -7.27419953 -0.33499230 1.02663946
*

C8
N 0.98511264 1.16751337 -1.44091441
O 2.20954615 0.76686982 -1.25019710
C 0.53505031 2.27859382 -0.96518633
C 0.14091736 0.25085873 -2.21009182
H -0.50961168 2.51187514 -1.18157555
C 1.39925087 3.15853961 -0.13974247
H 0.62931275 0.06654151 -3.17639648
H 0.06927958 -0.68925283 -1.64696384
H -0.85623935 0.68369005 -2.35497468
H 2.32463596 3.41097307 -0.68292924
H 0.86699218 4.07900161 0.13349946
H 1.69940130 2.62183241 0.77652038
C 0.26502486 0.17623266 1.57271948
C -0.93772027 0.07684981 1.31000675
O 1.47689277 0.26800281 1.82498635
Li 2.60642398 -0.21722694 0.34801194
O 2.23184958 -2.09626547 -0.00518952
O 4.52880881 0.00201638 0.61587507
C 1.61688198 -2.95240428 0.93878836
H 0.58324716 -3.19615878 0.63316817
H 2.19087192 -3.89040147 1.04882282
H 1.59256608 -2.42410408 1.90101248
C 2.35545503 -2.66058825 -1.29496123

H 1.36724656 -2.95168520 -1.69567913
H 2.79905699 -1.90056880 -1.95238121
H 3.00513840 -3.55468498 -1.27340507
C 5.40274719 -0.81707141 -0.13776984
H 6.42222965 -0.79594979 0.28766444
H 5.01827274 -1.84532053 -0.09538265
H 5.44805070 -0.48579563 -1.19137365
C 4.94183131 1.35551936 0.67661326
H 4.99919189 1.79828446 -0.33349653
H 4.20063120 1.90579269 1.27247203
H 5.92962379 1.43918619 1.16462636
C -2.30636998 0.03374800 0.89673481
C -3.24250991 0.99664086 1.34199043
C -2.77665701 -0.96354409 0.00954642
C -4.56744538 0.96831492 0.92216655
H -2.90989656 1.77930398 2.02923706
C -4.10084972 -0.99332835 -0.41241401
H -2.07894507 -1.72501175 -0.34951963
C -5.01846107 -0.02662435 0.03645239
H -4.43650958 -1.77491786 -1.09982750
H -5.26896192 1.72659717 1.28118776
C -6.39251179 -0.05551168 -0.40257792
C -7.54579905 -0.07637169 -0.77775605
H -8.57837036 -0.09792424 -1.08978492
*

C9
N 0.99520484 1.16078193 -1.45390786
O 2.21918708 0.76522478 -1.25128216
C 0.53977963 2.27481066 -0.99002917
C 0.15943580 0.23717749 -2.22408115
H -0.50378776 2.50454348 -1.21551593
C 1.39785172 3.16336090 -0.16716439
H 0.65617602 0.04829162 -3.18521593
H 0.08568586 -0.69986231 -1.65605560
H -0.83738362 0.66701501 -2.37988484
H 2.32844577 3.40760616 -0.70515450
H 0.86506510 4.08801383 0.09043698
H 1.68956119 2.63796267 0.75828738
C 0.23642415 0.16868980 1.55002815
C -0.96743339 0.07676686 1.28466421
O 1.44568034 0.25640304 1.80151133
Li 2.60400928 -0.22667112 0.34239193
O 2.23532719 -2.10513306 -0.01089269
O 4.51964982 0.00222211 0.63519994
C 1.61380380 -2.96357023 0.92678680
H 0.58396883 -3.21024551 0.61118457
H 2.18927167 -3.90001049 1.04174322
H 1.57913826 -2.43632053 1.88928909
C 2.37773088 -2.67062304 -1.29852995
H 1.39610038 -2.96810218 -1.71052898
H 2.82480065 -1.90913122 -1.95193072
H 3.03232873 -3.56076158 -1.26766770
C 5.40641783 -0.81611633 -0.10446731
H 6.41960034 -0.79155699 0.33546405
H 5.02401554 -1.84528239 -0.06565497
H 5.46570970 -0.48653340 -1.15789167
C 4.92821547 1.35710563 0.69907086
H 4.99780283 1.79818086 -0.31096246
H 4.17768134 1.90647144 1.28401981
H 5.90911019 1.44412726 1.20013901
C -2.33404448 0.05047705 0.87898193
C -3.26466568 1.00187530 1.36564844
C -2.81071755 -0.91830426 -0.03920840
C -4.59087926 0.99181031 0.95654038
H -2.92440502 1.75905037 2.07653196
C -4.13574498 -0.93359473 -0.45258925
H -2.11615322 -1.66736148 -0.42772626
C -5.03902734 0.02356337 0.04131736
H -4.48152844 -1.68967975 -1.16257901
H -5.29041759 1.73724407 1.34394256
C -6.41346947 0.01213696 -0.38777379
N -7.51955835 0.00299535 -0.73495432
*

D0
N 0.17654626 1.40700199 -1.67027934
O 0.84172082 1.09305886 -0.44400369
C -1.25117741 1.53900008 -1.27559009
C 0.41007300 0.28660624 -2.57302795
H -1.85546778 1.17077095 -2.12158824
C -1.59506381 3.00483392 -1.01334686
H 1.49176845 0.17505362 -2.73557178
H -0.00245644 -0.66572093 -2.18948958
H -0.06294418 0.52365102 -3.53747666
H -1.46540481 3.61223514 -1.92430159
H -2.63860679 3.10245262 -0.67441166
H -0.94207128 3.41143727 -0.22412567
C -0.08781597 0.46474636 0.43242210
C -1.35958627 0.64849740 -0.05251088
O 0.47047864 -0.11107265 1.40286749
Li 2.14523770 -0.24371832 0.42376247
O 2.23900950 -1.89207038 -0.52930505
O 3.88583574 0.45519968 0.69603905
C 1.22840486 -2.88037160 -0.40843650
H 0.68732396 -3.00379186 -1.36320232
H 1.66738674 -3.85091487 -0.11590395
H 0.52752567 -2.54464256 0.36753902
C 3.18132647 -2.16018955 -1.55139074
H 2.67771557 -2.27747006 -2.52727173
H 3.87575372 -1.31023806 -1.60473769
H 3.75022883 -3.08049139 -1.32843435
C 5.01487519 -0.33282116 1.03290552
H 5.36583565 -0.09549285 2.05267720
H 4.71460285 -1.38922386 0.99207732
H 5.83844114 -0.16069607 0.31743986
C 4.13360918 1.85235957 0.71549560
H 4.92718815 2.11520253 -0.00611730
H 3.20150129 2.35963093 0.43095183
H 4.43680042 2.18052238 1.72532798
C -2.59414675 0.13142558 0.51593956
C -3.83959337 0.36507441 -0.11159653
C -2.60598376 -0.63461620 1.70722597
C -5.02723693 -0.14063023 0.41913905
H -3.87913070 0.95194240 -1.03359042
C -3.79633976 -1.13639819 2.23033823
H -1.65877161 -0.82892668 2.21529488
C -5.01958072 -0.89649446 1.59429641
H -5.97257897 0.06031306 -0.09571555
H -3.76861440 -1.72529506 3.15333153
H -5.95142959 -1.29128122 2.00982138
*

D1
N -1.95946963 -0.44025999 -0.79956226
O -0.99997341 0.37583262 -0.12796703
C -2.99545415 -0.71248730 0.23910236
C -1.25764614 -1.64565294 -1.22223691
H -3.36484914 -1.73784890 0.06167285
C -4.16487656 0.26096305 0.09120989
H -0.44296580 -1.36619916 -1.90615180
H -0.83837534 -2.21679552 -0.37196741
H -1.96771538 -2.28206889 -1.77131192
H -4.65739800 0.14995103 -0.88961164
H -4.91433763 0.08064752 0.87930013
H -3.80988460 1.29976816 0.19143461
C -1.07967560 0.11029769 1.28396200
C -2.22504358 -0.57664930 1.53268579
H -2.56302036 -0.90289555 2.51514992
O -0.07565798 0.56100904 1.90903788
Li 0.98134017 0.59782673 0.28164151
O 1.98829653 -1.00736720 0.05351805

O	1.78761444	1.99023670	-0.73358162
C	1.96225110	-2.02413591	1.04148502
H	1.44135833	-2.92216906	0.66421465
H	2.98885158	-2.30332613	1.33950112
H	1.42152294	-1.62547351	1.91041437
C	2.60298655	-1.40187337	-1.15828906
H	2.10985412	-2.29847430	-1.57435546
H	2.50605356	-0.57363911	-1.87396701
H	3.67419584	-1.62223989	-1.00126955
C	3.18400292	2.19320430	-0.85949523
H	3.48460093	3.14546866	-0.38766945
H	3.69411895	1.36293076	-0.35130447
H	3.48273758	2.20758128	-1.92285946
C	1.00290651	2.99965996	-1.34841108
H	1.21841873	3.05327757	-2.43033492
H	-0.05296693	2.73463901	-1.20092516
H	1.20329742	3.98378993	-0.88901368

*

D14

N	1.39843670	1.72607067	-1.44787988
O	1.88205822	1.00554700	-0.30757827
C	0.01475076	2.11042281	-1.06237554
C	1.43730410	0.80014332	-2.57356028
H	-0.59514224	2.08966138	-1.98028946
C	0.00006924	3.52073982	-0.47538946
H	2.47421152	0.47177513	-2.73313577
H	0.79093062	-0.08503571	-2.42314964
H	1.10374642	1.34466444	-3.46897856
H	0.33213724	4.26187205	-1.22053028
H	-1.01513443	3.78936600	-0.14338110
H	0.66931376	3.57624440	0.39834105
C	0.78828050	0.44781269	0.38344186
C	-0.38360510	1.02830099	-0.07699816
O	1.11569438	-0.42848996	1.21316718
Li	2.81076821	-0.76401727	0.29326208
O	2.57881940	-2.12401832	-1.01538228
O	4.62711960	-0.53588957	0.76252800
C	1.37407756	-2.86314376	-1.14198939
H	0.89626792	-2.66519722	-2.11758825
H	1.57185200	-3.94596024	-1.05124789
H	0.70101254	-2.54448951	-0.33489383
C	3.52041661	-2.38704515	-2.04077599
H	3.08376996	-2.18176213	-3.03402883
H	4.38584847	-1.72820596	-1.88451360
H	3.85371180	-3.43943544	-2.00527299
C	5.54570341	-1.60426846	0.92498877
H	5.87179942	-1.68039290	1.97710324
H	5.03742051	-2.53526175	0.63726693
H	6.43043015	-1.45809452	0.28062690
C	5.15579269	0.73344059	1.11637138
H	6.03464462	0.97407962	0.49279981
H	4.37115933	1.48233197	0.94151753
H	5.44876948	0.74979857	2.18077354
C	-1.71635331	0.69277607	0.32642137
C	-2.84005994	1.35083860	-0.24806067
C	-1.98010158	-0.31358288	1.30042851
C	-4.13557498	1.02697244	0.11643812
H	-2.68545694	2.13033624	-0.99741238
C	-3.27272033	-0.64080384	1.66685874
H	-1.13924863	-0.83303604	1.76240009
C	-4.35360105	0.02906764	1.07496469
H	-3.45729671	-1.41502112	2.41354680
H	-4.98478136	1.54130282	-0.33656172
N	-5.70711492	-0.31243456	1.45863369
O	-5.87462116	-1.19099858	2.29334307
O	-6.63386854	0.28954484	0.93361658

*

D15

N	1.07231706	1.66958912	-1.55307668
O	1.65176969	1.17881041	-0.33923167
C	-0.36708253	1.84109880	-1.21827594
C	1.29844508	0.64661568	-2.56687290
H	-0.94240733	1.59962116	-2.12777002
C	-0.64975947	3.28700546	-0.81338096
H	2.38041500	0.49236590	-2.68692764
H	0.81685821	-0.31786047	-2.31787606
H	0.89286045	1.01869156	-3.51920214
H	-0.43926322	3.97908294	-1.64519135
H	-1.70445794	3.40660817	-0.51882331
H	-0.02199377	3.56986228	0.04706046
C	0.64829726	0.51830592	0.41194905
C	-0.58593053	0.83181307	-0.10884826
O	1.10999009	-0.19480142	1.33789065
Li	2.84300014	-0.32514617	0.45473039
O	2.90785149	-1.84383741	-0.68696883
O	4.58347336	0.26382209	0.91098872
C	1.84625440	-2.78297351	-0.75013245
H	1.37151644	-2.76784880	-1.74705137
H	2.21717777	-3.80276152	-0.54391161
H	1.10661151	-2.50019612	0.01092887
C	3.90918983	-2.04601332	-1.66792382
H	3.47199743	-2.02950445	-2.68190662
H	4.64184800	-1.23183961	-1.57889372
H	4.42000226	-3.01301957	-1.51258547
C	5.66396162	-0.60345137	1.21158722
H	5.96719800	-0.49647298	2.26793944
H	5.32904773	-1.63542268	1.03623632
H	6.53046985	-0.38671199	0.56215124
C	4.87814093	1.63822792	1.10822456
H	5.71894899	1.95052956	0.46406798
H	3.98147995	2.21306798	0.83880801
H	5.13629875	1.83352715	2.16389820
C	-1.86271461	0.32606933	0.34767188
C	-3.06291983	0.69648546	-0.30219497
C	-1.95402710	-0.56290586	1.44682521
C	-4.27989915	0.19434632	0.13437789
H	-3.06814255	1.37737977	-1.15547873
C	-3.18666933	-1.04394042	1.85275293
H	-1.05835225	-0.87662871	1.98490315
C	-4.37095559	-0.68073289	1.21286603
F	-3.26983618	-1.88636063	2.89415335
F	-5.55650218	-1.15547905	1.62473209
F	-5.41436729	0.54886913	-0.48778862

*

D18

N	1.57438068	1.76415653	-1.49030595
O	2.00590114	1.04793234	-0.33163650
C	0.15254169	2.08846747	-1.19795250
C	1.72367671	0.85908719	-2.62302293
H	-0.39096712	2.05537337	-2.15760525
C	0.03987413	3.49389899	-0.60739568
H	2.78156059	0.57475252	-2.71952221
H	1.10763295	-0.05470375	-2.52515023
H	1.42609501	1.40046567	-3.53325877
H	0.38522788	4.25647655	-1.32508245
H	-1.00435047	3.71702489	-0.33705274
H	0.65094121	3.56930167	0.30664696
C	0.86574617	0.43421727	0.27417412
C	-0.27237690	0.98244744	-0.25155305
O	1.18325137	-0.45742290	1.11086776
Li	2.91779010	-0.69071503	0.27697564
O	2.86157642	-2.05965239	-1.04667547
O	4.70717640	-0.42215376	0.85333289
C	1.68539376	-2.81973391	-1.27202079
H	1.27406473	-2.61345206	-2.27596361
H	1.89873015	-3.90024668	-1.18635481
H	0.95135642	-2.52765734	-0.50902542

C 3.87667205 -2.27682313 -2.00875171
H 3.50296764 -2.06643681 -3.02659067
H 4.70937616 -1.59614487 -1.78342892
H 4.23898215 -3.31968022 -1.96823666
C 5.63819485 -1.46417264 1.08893757
H 5.88229762 -1.53402711 2.16370986
H 5.18014825 -2.40811739 0.76180069
H 6.56775955 -1.29363791 0.51734993
C 5.17060458 0.86131046 1.24251538
H 6.08816320 1.12667889 0.68823132
H 4.37991492 1.58613954 1.00484053
H 5.38048349 0.88800793 2.32644503
C -1.65314591 0.62720048 0.06975038
C -2.74496806 1.24417535 -0.57114072
C -1.97668888 -0.36179235 1.02311423
C -4.06875701 0.90712300 -0.28375743
H -2.56486317 2.01323932 -1.32826544
C -3.29423281 -0.70717006 1.31647655
H -1.16404934 -0.87546489 1.54285904
C -4.38558550 -0.07193780 0.68172645
H -3.46777168 -1.48487574 2.06208701
H -4.86118402 1.42670080 -0.82486423
N -5.70589274 -0.39259839 0.99791214
C -6.76140617 0.07921344 0.12665703
C -5.96360027 -1.58983060 1.77031467
H -7.73151155 -0.24803695 0.52557067
H -6.78153481 1.18033992 0.08063287
H -6.67022460 -0.29987293 -0.91193490
H -7.04494246 -1.68215052 1.94133124
H -5.61383817 -2.51501737 1.26812821
H -5.47920437 -1.54167783 2.75908105
*

D19
N 2.02327606 2.34161343 -0.20994273
O 2.39478680 1.12878947 0.46249214
C 0.56771797 2.47093463 0.06632127
C 2.32031429 2.13884228 -1.62321993
H 0.10762748 2.92786023 -0.82465352
C 0.33268164 3.36778526 1.27985809
H 3.39506854 1.94006071 -1.73871341
H 1.74224125 1.30709802 -2.06832631
H 2.08028780 3.07203097 -2.15304455
H 0.70617018 4.38716307 1.09198121
H -0.74235043 3.42626775 1.51240793
H 0.85069503 2.95939231 2.16239408
C 1.27759545 0.29857607 0.57228880
C 0.13044680 1.03432279 0.27257041
O 1.53934096 -0.87678369 0.88809069
Li 3.40303668 -0.71501055 0.26223909
O 3.49312504 -1.18704274 -1.57143701
O 5.07628724 -0.75665881 1.12525439
C 2.41149167 -1.77927807 -2.27459674
H 2.04210407 -1.10096665 -3.06364349
H 2.72468775 -2.73231580 -2.73605267
H 1.60695271 -1.97035315 -1.55198455
C 4.59160680 -0.84448328 -2.39967629
H 4.27479487 -0.15662240 -3.20308752
H 5.34640090 -0.34643303 -1.77535042
H 5.03464097 -1.74838206 -2.85377608
C 6.08250847 -1.71935891 0.85254682
H 6.27039446 -2.34710619 1.74098911
H 5.72826056 -2.35627683 0.03000735
H 7.02252084 -1.22453233 0.55145256
C 5.40178904 0.12707785 2.18839316
H 6.32220574 0.69035777 1.95510409
H 4.56644094 0.83095887 2.30656548
H 5.54488587 -0.43289996 3.12893174
C -1.19250914 0.52578256 0.22068800
C -2.29144197 1.36323011 -0.11464292
C -1.49227027 -0.84085083 0.48594080
C -3.59256748 0.87904728 -0.17750493
H -2.12372079 2.41795665 -0.33801121
C -2.78732489 -1.33355754 0.40772683
H -0.68413706 -1.51861639 0.76174461
C -3.86459136 -0.48108933 0.07909557
N -5.21632935 -0.99081506 0.00746877
O -5.37131688 -2.19309018 -0.12666423
O -6.13676623 -0.19423559 0.08372590
C -4.60717197 1.83547649 -0.56716806
N -5.32250910 2.67967970 -0.90408078
C -2.95035867 -2.73597535 0.72859525
N -2.96096119 -3.85578783 1.01810523
*

D2
N 1.03562486 1.61495017 -1.57417876
O 1.61051897 1.12567961 -0.35894616
C -0.39455446 1.83253718 -1.22804821
C 1.22167170 0.57106185 -2.57469039
H -0.98495084 1.59966409 -2.13011103
C -0.62901000 3.29096961 -0.83669931
H 2.29753715 0.38301096 -2.70220986
H 0.71477879 -0.37523291 -2.30665749
H 0.81821744 0.94008254 -3.52916660
H -0.40778735 3.96793944 -1.67826549
H -1.67580465 3.44565545 -0.53064261
H 0.01725735 3.56378232 0.01326418
C 0.59188533 0.50054810 0.40977926
C -0.63572659 0.84226156 -0.10497749
O 1.04820708 -0.21345307 1.33980504
Li 2.76071422 -0.39810208 0.43383382
O 2.77015572 -1.94565388 -0.67609312
O 4.53032205 0.15798160 0.82490884
C 1.67513345 -2.84689782 -0.71473286
H 1.19439996 -2.83360025 -1.70883620
H 2.01049031 -3.87539690 -0.49133960
H 0.95172956 -2.52283387 0.04537584
C 3.75455562 -2.19941086 -1.66180745
H 3.30888950 -2.18517927 -2.67219463
H 4.51549881 -1.40963951 -1.59300305
H 4.23323114 -3.18075095 -1.49415792
C 5.59319651 -0.72232131 1.14822397
H 5.92218421 -0.56975659 2.19126083
H 5.22723113 -1.75228902 1.03327381
H 6.45122891 -0.56325432 0.47134460
C 4.86835768 1.53200098 0.93629954
H 5.70082340 1.78238181 0.25523028
H 3.98132067 2.11640447 0.65601264
H 5.15914425 1.77889752 1.97253037
C -1.93110165 0.38263668 0.36144569
C -3.12451214 0.78802903 -0.27932236
C -2.06036806 -0.49651522 1.46456660
C -4.37475172 0.34359800 0.14893770
H -3.07863574 1.46638915 -1.13524137
C -3.30554137 -0.94480431 1.89656866
H -1.15865213 -0.82710461 1.98424558
C -4.46168632 -0.52324166 1.23713021
H -3.38230797 -1.62348520 2.75026876
H -5.28329142 0.67066305 -0.36349545
Cl -6.02536364 -1.08362875 1.78031047
*

D21
N 1.81135283 2.14921128 -0.83104283
O 2.22080352 1.15147203 0.11466769
C 0.37273334 2.36394133 -0.52013712
C 2.02949102 1.57871507 -2.15518709
H -0.13677311 2.57946385 -1.47344781
C 0.20500445 3.55119384 0.42594697
H 3.09743683 1.34726439 -2.27454583
H 1.43381450 0.66322709 -2.33189621

H 1.75345522 2.34018628 -2.89905971
H 0.56687282 4.48117750 -0.04164237
H -0.85530792 3.68348993 0.69300146
H 0.77191300 3.38063551 1.35529139
C 1.10541222 0.38731374 0.49085459
C -0.05108513 1.03630104 0.07625028
O 1.39393140 -0.67553173 1.07808700
Li 3.20935055 -0.68289389 0.33157596
O 3.23756644 -1.62391224 -1.31448508
O 4.92756279 -0.50749947 1.09374482
C 2.11989164 -2.35563531 -1.79395089
H 1.72901760 -1.90524162 -2.72335863
H 2.39956881 -3.40545556 -1.99213515
H 1.34290346 -2.32380488 -1.01843878
C 4.30202494 -1.53233478 -2.24533547
H 3.95562017 -1.07848284 -3.19049189
H 5.08433133 -0.89763645 -1.80631673
H 4.72171078 -2.53143008 -2.45859145
C 5.91903475 -1.52120997 1.05370914
H 6.15821039 -1.87164415 2.07301407
H 5.52093358 -2.36182022 0.46816278
H 6.84012933 -1.14659016 0.57350898
C 5.31134013 0.64166278 1.83429562
H 6.21454000 1.10107063 1.39563922
H 4.48119662 1.35995879 1.78979677
H 5.51173550 0.37669242 2.88716091
C -1.38344200 0.55005404 0.22108474
C -2.48788630 1.29774341 -0.27132026
C -1.66138813 -0.69787504 0.84665218
C -3.77390623 0.82241363 -0.13932311
H -2.35087054 2.25597628 -0.77475353
C -2.95395050 -1.15631983 0.94717061
H -0.85638285 -1.30465169 1.26164113
C -4.05696833 -0.41853573 0.46621057
N -5.40612683 -0.91025837 0.58664832
O -5.59116676 -2.11428837 0.50601970
O -6.30284812 -0.10076539 0.76283203
F -3.16695695 -2.31967209 1.56490279
F -4.77163058 1.54672385 -0.64740355
*

D6
N 1.06263881 1.71418086 -1.60673656
O 1.62259087 1.21965943 -0.38750285
C -0.38051922 1.89048540 -1.29218860
C 1.29568571 0.69261047 -2.62007457
H -0.94291652 1.65080581 -2.21066364
C -0.66540077 3.33868142 -0.89549535
H 2.37805215 0.52812608 -2.72319058
H 0.80119583 -0.26791870 -2.38156126
H 0.90930764 1.07004739 -3.57843502
H -0.44258168 4.02933557 -1.72559296
H -1.72342288 3.46132709 -0.61430750
H -0.04807431 3.62166416 -0.02750817
C 0.59536180 0.56400308 0.35316863
C -0.62340937 0.88446860 -0.18411907
O 1.05099924 -0.15398721 1.28554131
Li 2.75964684 -0.32377109 0.38812353
O 2.78575046 -1.85688500 -0.74366876
O 4.53953185 0.18922738 0.80213912
C 1.68249404 -2.74599077 -0.81252565
H 1.21210240 -2.70784845 -1.81095460
H 2.00535696 -3.78219377 -0.60706028
H 0.95465248 -2.42992328 -0.05323800
C 3.77780579 -2.09865922 -1.72425626
H 3.34548028 -2.04961446 -2.73937225
H 4.55023033 -1.32344964 -1.62394927
H 4.23897562 -3.09181684 -1.57839741
C 5.55707323 -0.72666421 1.16940316
H 5.77649244 -0.65524265 2.24937793
H 5.19876610 -1.74033824 0.94175329
H 6.48124917 -0.53113479 0.59729030
C 4.86689542 1.54556654 1.06266075
H 5.77862356 1.83670667 0.51188807
H 4.02388136 2.16207403 0.72157577
H 5.02712631 1.70772410 2.14337514
C -1.92910402 0.40016972 0.25091646
C -3.10915665 0.75535436 -0.42717411
C -2.07447803 -0.46035140 1.36739777
C -4.36897571 0.28730772 -0.03080153
H -3.05720375 1.41768508 -1.29580941
C -3.31901294 -0.93024803 1.76661795
H -1.18145594 -0.75761550 1.92191663
C -4.48188037 -0.56238365 1.07209536
H -3.41103296 -1.59409136 2.63130736
H -5.24936224 0.59755665 -0.59663000
O -5.66168024 -1.07030272 1.53950247
C -6.84838524 -0.74777132 0.84375959
H -6.81266810 -1.09978306 -0.20209387
H -7.66877647 -1.25966305 1.36509891
H -7.04196445 0.33905811 0.85295337
*

D8
N 1.07926876 2.08175178 -0.80461594
O 1.48494450 1.14304612 0.19649901
C -0.38491337 2.22331494 -0.58522414
C 1.40306801 1.48384729 -2.09404570
H -0.84660692 2.37876392 -1.57459864
C -0.67387141 3.43415435 0.30089724
H 2.48685016 1.30598986 -2.14558877
H 0.86748858 0.53234033 -2.27300561
H 1.13207415 2.20473187 -2.87937905
H -0.34237723 4.36773112 -0.18271528
H -1.75289523 3.51211343 0.50772738
H -0.14974383 3.33270657 1.26501582
C 0.37598765 0.32504665 0.53431698
C -0.77433639 0.89514004 0.03505884
O 0.70284736 -0.70621700 1.17252404
Li 2.52597592 -0.62041322 0.48488841
O 2.67775907 -1.64024527 -1.11417946
O 4.21983136 -0.37169089 1.29428537
C 1.60101653 -2.42067090 -1.60898396
H 1.23537701 -2.01637594 -2.56947341
H 1.91715217 -3.46865082 -1.75701136
H 0.79365692 -2.38217816 -0.86541867
C 3.77568931 -1.55188208 -2.00415828
H 3.45622621 -1.14667318 -2.98054863
H 4.52007421 -0.87611584 -1.56049375
H 4.23407260 -2.54470420 -2.16031022
C 5.22109776 -1.37325938 1.35872705
H 5.40480014 -1.67392272 2.40525563
H 4.86552250 -2.24459337 0.79106095
H 6.16517195 -1.01041699 0.91518758
C 4.54902049 0.81565015 1.99879582
H 5.46943906 1.26716367 1.58832274
H 3.71380952 1.51874102 1.87582123
H 4.69533689 0.60356893 3.07251793
C -2.11434639 0.35783651 0.12238295
C -3.21316524 1.03667641 -0.45900723
C -2.39074392 -0.86938784 0.77830074
C -4.50371854 0.52438306 -0.39222940
H -3.05113959 1.98631925 -0.97539851
C -3.68014086 -1.37974753 0.84319072
H -1.56607007 -1.41662948 1.23926550
C -4.76313816 -0.69391785 0.26010173
H -3.86146551 -2.32879694 1.35629155
H -5.33003518 1.07418963 -0.85209938
C -6.10170839 -1.22473300 0.33281327
C -7.22847902 -1.67121900 0.39538672
H -8.23038514 -2.06771373 0.45087901
*

D9
N 1.05814158 2.07616323 -0.82124523
O 1.46805588 1.13872901 0.18060445
C -0.40475576 2.21924475 -0.59478982
C 1.37659276 1.47466961 -2.11046267
H -0.87107220 2.37364554 -1.58189751
C -0.68813804 3.43021327 0.29261009
H 2.46022931 1.29781752 -2.16637338
H 0.84081028 0.52234360 -2.28440693
H 1.10126620 2.19359687 -2.89597880
H -0.35722561 4.36282898 -0.19296490
H -1.76620171 3.50975644 0.50368665
H -0.16019793 3.32846443 1.25457025
C 0.36583344 0.32281182 0.52533469
C -0.79115895 0.89169200 0.02900335
O 0.69144036 -0.70461790 1.16500189
Li 2.52307993 -0.61947313 0.48546608
O 2.67867866 -1.63603499 -1.11376626
O 4.20872510 -0.35929629 1.30405634
C 1.61305849 -2.43143623 -1.60897199
H 1.24069355 -2.03089334 -2.56842306
H 1.94447823 -3.47434881 -1.75888620
H 0.80553820 -2.40643825 -0.86499859
C 3.77636803 -1.53426246 -2.00311469
H 3.45256785 -1.13272358 -2.97954257
H 4.51231952 -0.84982103 -1.55867313
H 4.24641904 -2.52154296 -2.15920188
C 5.22065597 -1.35096889 1.36102039
H 5.41113237 -1.65335509 2.40578103
H 4.87187401 -2.22388495 0.79158510
H 6.15915377 -0.97663373 0.91533799
C 4.52904812 0.82864262 2.01202514
H 5.44220339 1.29173562 1.59837268
H 3.68587061 1.52356415 1.89710075
H 4.68349526 0.61327328 3.08389171
C -2.12217130 0.35070490 0.12311470
C -3.22762848 1.02956354 -0.45314488
C -2.38868549 -0.88124667 0.78046105
C -4.51370128 0.51475282 -0.38207841
H -3.07020949 1.98028613 -0.96800664
C -3.67246775 -1.39738650 0.85191204
H -1.55923479 -1.42485424 1.23614473
C -4.75417734 -0.70685030 0.27181409
H -3.85035819 -2.34740929 1.36345066
H -5.34609302 1.05951266 -0.83589814
C -6.08623475 -1.24207732 0.34842405
N -7.16112125 -1.67418036 0.40998890
*

E0
C -1.35636869 -2.76439278 1.99803170
H -0.28381097 -2.74349907 1.79729303
H -1.62626172 -3.79548808 2.24016122
C -2.09019725 -2.39971717 0.77936344
C -2.85141479 -2.16182652 -0.14431420
H -3.64597382 -2.10748683 -0.86319374
C -2.31438089 0.52871432 2.77713911
H -2.08788294 1.57577457 2.60795666
H -3.36123524 0.25625526 2.86452135
C -1.35946435 -0.39326932 2.89947195
C -1.72312662 -1.83334059 3.16555981
H -1.21077707 -2.20439219 4.05558391
H -2.79521139 -1.90371840 3.35466372
Au -1.09090235 -0.89324169 -0.65510248
P 0.32897855 0.40749761 -1.89232313
C 0.02390199 0.16168050 -3.65748314
C 0.18896925 2.18905401 -1.63415849
C 2.06683170 -0.01180313 -1.63946835
H 2.22478688 -1.05749555 -1.90928992
H 2.69283094 0.62680041 -2.26702836
H 2.33143781 0.13012848 -0.59135634
H 0.14919465 -0.89335596 -3.90619518
H 0.72868043 0.76138306 -4.23820810
H -0.99680042 0.46534131 -3.89643975
H -0.84460299 2.49941500 -1.79612524
H 0.84041427 2.70631440 -2.34251401
H 0.48515434 2.44549401 -0.61760099
C 0.08431224 -0.04903208 2.75492132
C 1.07285425 -0.77009998 3.42914951
C 0.48279460 1.01324122 1.93806747
C 2.41383030 -0.42256106 3.31274209
H 0.80100698 -1.60373195 4.06592293
C 1.82029476 1.36588438 1.82615942
H -0.26454866 1.56370084 1.37910441
C 2.79344515 0.64961266 2.51486262
H 3.16345186 -0.99188289 3.85204390
H 2.10579533 2.19601427 1.18867203
H 3.83969586 0.92110693 2.42412883
*

E1
C -2.82404109 -1.26075564 0.54645727
H -1.95102939 -1.79860463 0.91934932
H -3.63203730 -1.98711854 0.42778014
C -2.52909063 -0.71667804 -0.78534832
C -2.46617207 -0.23701871 -1.90566728
H -2.62277035 0.13279395 -2.90040467
C -2.42695570 2.16505584 1.32733944
H -1.69979640 2.95688620 1.47085585
H -3.37407030 2.43886101 0.87369049
C -2.19277190 0.90904022 1.70795441
C -3.25188065 -0.14965638 1.51979925
H -3.49657868 -0.62491588 2.47250367
H -4.16350201 0.31957402 1.14722226
Au -0.34030655 -0.14234453 -1.23813441
P 1.93305122 0.09088490 -1.36234977
C 2.51114471 -0.27420780 -3.03612975
C 2.54981359 1.74876134 -1.00096534
C 2.83500855 -1.04403446 -0.28599920
H 2.57226848 -2.07041474 -0.54803517
H 3.90930712 -0.89589251 -0.41852421
H 2.56461936 -0.85997703 0.75379738
H 2.22932351 -1.29360394 -3.30483029
H 3.59798026 -0.17135450 -3.07962139
H 2.05057111 0.42009477 -3.74090629
H 2.08013590 2.46216370 -1.68037654
H 3.63316449 1.77130235 -1.14038759
H 2.30960705 2.02253091 0.02577634
C -0.89024954 0.49703175 2.30291310
C -0.80507943 -0.57714464 3.19383716
C 0.28931744 1.17447533 1.98886864
C 0.40697280 -0.94187624 3.76213162
H -1.69568638 -1.13393834 3.46153731
C 1.49840694 0.81366568 2.56826839
H 0.26534685 1.98964228 1.27463927
C 1.58098553 -0.25120506 3.46230232
H 0.44125086 -1.77857944 4.45376793
H 2.39968213 1.36004881 2.30829534
C 2.89731378 -0.66801344 4.06358596
H 2.83710283 -0.70234867 5.15468796
H 3.18095124 -1.66775741 3.72125904
H 3.69367580 0.02495660 3.78542715
*

E10
C -2.86939498 -0.95230265 1.45719194
H -2.18158038 -1.79846989 1.47746833
H -3.84183348 -1.31436310 1.80172557
C -3.03589597 -0.53443261 0.06139870
C -3.29219051 -0.18898862 -1.07952554
H -3.72952799 0.07979747 -2.02117041

C	-1.16684106	1.93902686	1.16880575
H	-0.28717414	2.42845512	0.76838106
H	-2.12579140	2.38537502	0.92761130
C	-1.10399183	0.84315956	1.93251014
C	-2.39384804	0.19210807	2.37760463
H	-2.29656153	-0.21868973	3.38418551
H	-3.17654097	0.95132108	2.40503677
Au	-1.04297630	-0.10464204	-1.04322463
P	1.13730480	-0.01870482	-1.72508258
C	1.32949106	-0.34170604	-3.49303462
C	1.93985881	1.57393201	-1.43962119
C	2.15957061	-1.24855201	-0.88674599
H	1.78978933	-2.24720817	-1.12577884
H	3.19707623	-1.15341342	-1.21587426
H	2.10106090	-1.09620935	0.19124964
H	0.92127198	-1.32561772	-3.72965670
H	2.38844672	-0.31007354	-3.76014554
H	0.78686762	0.41473736	-4.06258579
H	1.39045259	2.35709460	-1.96483374
H	2.96754710	1.53723108	-1.80849910
H	1.94416469	1.79484116	-0.37257237
C	0.19572675	0.20830113	2.29708185
C	0.25629649	-1.10524177	2.76598501
C	1.39674295	0.91538166	2.16918248
C	1.48107143	-1.69229885	3.06630498
H	-0.64607252	-1.68446534	2.91051340
C	2.62857614	0.33693431	2.44872417
H	1.37762389	1.95222528	1.85437339
C	2.66106575	-0.98275615	2.90262572
H	1.50930412	-2.71416033	3.42986754
H	3.61401218	-1.44949614	3.13219134
C	3.90367111	1.10284174	2.21154231
H	4.29480064	0.87857377	1.21318240
H	3.73535013	2.18021111	2.26880019
H	4.67287456	0.82957559	2.93707631

*

E11

C	-2.66788612	-1.08516871	1.25896591
H	-1.79949177	-1.74449514	1.30832758
H	-3.56056666	-1.70698865	1.36459024
C	-2.73072936	-0.45919231	-0.06787683
C	-2.94979035	0.10529918	-1.12734957
H	-3.35362890	0.57036848	-2.00554458
C	-1.54267838	2.15516886	2.00978108
H	-0.69640157	2.83195485	1.97055297
H	-2.52343046	2.57981049	1.82158865
C	-1.40862890	0.86092216	2.29961370
C	-2.62804322	-0.02540554	2.37231498
H	-2.67063598	-0.55064885	3.32848197
H	-3.52302880	0.59454710	2.30418025
Au	-0.72247186	-0.02599101	-1.11028751
P	1.45185473	-0.00323522	-1.82094630
C	1.55007298	-0.31839372	-3.59838239
C	2.32859999	1.55361367	-1.56928506
C	2.44538521	-1.29795753	-1.04733024
H	2.04582722	-2.27065895	-1.34039434
H	3.48321647	-1.21395211	-1.37778782
H	2.39402904	-1.20815470	0.03712614
H	1.08300285	-1.27789618	-3.82597265
H	2.59708180	-0.33737884	-3.90993408
H	1.02394494	0.47112984	-4.13768270
H	1.77524333	2.35935939	-2.05476848
H	3.32641899	1.47967893	-2.00842472
H	2.41870421	1.76545529	-0.50497226
C	-0.07148461	0.24436646	2.54383570
C	0.06319333	-0.89707680	3.33883441
C	1.07442263	0.81199023	1.98425320
C	1.32064401	-1.43593201	3.58212933
H	-0.80572822	-1.36365353	3.78509829
C	2.32825878	0.27371294	2.24334333
H	1.00147484	1.67857895	1.33920973
C	2.46109925	-0.85876590	3.04230080
H	1.41500119	-2.31845239	4.20582828
H	3.44355858	-1.28096368	3.23105415
O	3.40625942	0.88573539	1.66790832
H	4.21125715	0.40986290	1.91563973

*

E12

C	-0.85618406	-1.25118850	2.79806118
H	-0.17114687	-1.69922966	2.07650728
H	-1.28390845	-2.06351536	3.39071153
C	-1.95775859	-0.59912637	2.07681343
C	-2.93412237	-0.02101990	1.62771831
H	-3.87753832	0.45012542	1.43086935
C	-0.04832665	2.17548274	3.32619232
H	0.32205483	3.07674048	2.84976112
H	-0.78452144	2.29888965	4.11398617
C	0.38941452	0.96290077	2.98677441
C	-0.12497482	-0.25626810	3.71461192
H	0.69654919	-0.79610690	4.19196616
H	-0.80429863	0.06554574	4.50518787
Au	-1.56149912	-0.11170602	-0.13335348
P	-0.71246521	-0.03985555	-2.25460203
C	-1.87109123	-0.71216558	-3.46682751
C	-0.31764947	1.62416148	-2.83135447
C	0.79791647	-1.01406680	-2.42026435
H	0.57907022	-2.05891703	-2.19339711
H	1.18208098	-0.93107970	-3.43957558
H	1.54065498	-0.64373565	-1.71518305
H	-2.10480715	-1.74682958	-3.21058249
H	-1.42370456	-0.67377243	-4.46287634
H	-2.79149075	-0.12590392	-3.45715980
H	-1.22365961	2.23262358	-2.82441057
H	0.08192658	1.57458121	-3.84695561
H	0.42047260	2.07588869	-2.16873991
C	1.37762853	0.76661622	1.88979780
C	2.26316148	-0.31431326	1.89337908
C	1.46118542	1.66758718	0.82524510
C	3.21243572	-0.47217230	0.89325710
H	2.22862457	-1.04205437	2.69612659
C	2.42050726	1.51740134	-0.16504328
H	0.76331156	2.49538330	0.76560656
C	3.31468685	0.44653126	-0.15179420
H	3.88014909	-1.32631570	0.93628197
H	2.46729001	2.23602915	-0.97723911
C	4.29278893	0.29471552	-1.26335405
C	5.61765697	-0.39393801	-1.05684966
C	4.55152828	-1.06733091	-1.86392577
H	4.29356113	1.12478963	-1.96056051
H	5.82781724	-0.78213383	-0.06708059
H	6.47386889	0.01430429	-1.58044302
H	4.67847664	-1.12517114	-2.93850670
H	4.03042623	-1.90503622	-1.41429466

*

E13

C	-3.25769703	-0.89749779	-0.61709489
H	-2.72027771	-1.76833259	-0.24004961
H	-4.26136426	-1.23059375	-0.89475505
C	-2.59594442	-0.45490203	-1.84872862
C	-2.15264871	-0.08755401	-2.92357473
H	-1.97325922	0.20895432	-3.93854278
C	-1.62500778	1.93136966	0.19816504
H	-0.66557945	2.39425787	0.39689428
H	-2.24983362	2.40316736	-0.55274882
C	-2.04616237	0.83648512	0.83840015
C	-3.36786032	0.21567925	0.44665505
H	-3.86763734	-0.21409071	1.31684684
H	-4.01423848	0.99917336	0.04894660
Au	-0.32119991	-0.08636309	-1.61544717

P	1.84899953	-0.05197689	-0.90053532
C	3.03283030	-0.36713138	-2.22855120
C	2.34781038	1.52299073	-0.17097978
C	2.17058052	-1.30543440	0.35862743
H	1.98445632	-2.29567253	-0.06064139
H	3.20888296	-1.23722641	0.69171024
H	1.50294023	-1.14741330	1.20635964
H	2.82825480	-1.34193799	-2.67428826
H	4.04892601	-0.35335705	-1.82726014
H	2.93257747	0.40289600	-2.99538358
H	2.20680846	2.32336983	-0.89935021
H	3.39942458	1.47474916	0.12139417
H	1.73629126	1.72748622	0.70818109
C	-1.21902009	0.18392805	1.89273352
C	-1.41144560	-1.15132113	2.25167656
C	-0.22017799	0.89587951	2.56417872
C	-0.61606910	-1.76793281	3.21622820
H	-2.19262778	-1.73547586	1.78299389
C	0.57433077	0.28590677	3.52514533
H	-0.06281182	1.94836488	2.35541748
C	0.38516673	-1.05761126	3.85289864
H	-0.78953112	-2.80905032	3.46460535
H	1.01427134	-1.52044904	4.60447191
C	1.62861410	1.09176038	4.18542627
O	2.41094535	0.65665838	5.00179742
H	1.66797791	2.15339453	3.87628097
*			

E14

C	-1.78327614	-1.31687668	2.78128526
H	-1.16890650	-1.89922550	2.09291724
H	-2.59725131	-1.96446970	3.11467740
C	-2.37018647	-0.18366036	2.05118681
C	-2.89175848	0.81968981	1.59342580
H	-3.45627523	1.70631435	1.37872475
C	0.01303204	1.40974552	4.34522933
H	0.71915675	2.20568356	4.13330746
H	-0.68139315	1.56510598	5.16463888
C	0.00805133	0.27235466	3.65336852
C	-0.95568211	-0.84062268	3.98894905
H	-0.41819673	-1.71019356	4.37676946
H	-1.62756632	-0.49432141	4.77531678
Au	-1.63644472	0.09427148	-0.11422020
P	-0.74306598	-0.25082845	-2.19043608
C	-2.03499455	-0.37733165	-3.44689130
C	0.36173488	1.05643389	-2.76107435
C	0.19419545	-1.79076171	-2.29896647
H	-0.46343603	-2.63000487	-2.06613683
H	0.59133679	-1.90574473	-3.31020041
H	1.01582840	-1.77488414	-1.58246136
H	-2.70868531	-1.19815475	-3.19545615
H	-1.58053486	-0.56194917	-4.42311307
H	-2.60409561	0.55329473	-3.47917553
H	-0.16803697	2.01044898	-2.75154894
H	0.69400563	0.83229261	-3.77743679
H	1.22807457	1.12110563	-2.10306562
C	0.94173396	0.06853418	2.51234280
C	1.50980198	-1.18431691	2.26503715
C	1.25127426	1.13054604	1.65728193
C	2.39002556	-1.37135766	1.21193564
H	1.27607695	-2.02389285	2.90868491
C	2.13139340	0.96219175	0.60195185
H	0.78898057	2.09745682	1.81419289
C	2.68939095	-0.28982090	0.39924403
H	2.83721296	-2.33787151	1.02322475
H	2.37599432	1.78498452	-0.05559527
N	3.60841649	-0.48403002	-0.73215833
O	4.14288644	-1.57063676	-0.85649139
O	3.78590309	0.45196941	-1.49182132
*			

E15

C	-2.97805460	-1.04632727	-0.01766331
H	-2.17653932	-1.62277512	0.44753451
H	-3.82279727	-1.72264088	-0.17083278
C	-2.54268642	-0.58767683	-1.34310179
C	-2.35319753	-0.16681520	-2.47261942
H	-2.39781813	0.15908656	-3.49373775
C	-2.34533224	2.34928718	0.63548719
H	-1.57293976	3.08726812	0.82129182
H	-3.19340091	2.66345137	0.03630177
C	-2.28871757	1.11377323	1.13089001
C	-3.40710157	0.13606310	0.86745303
H	-3.79460609	-0.27392536	1.80250079
H	-4.22962791	0.66275097	0.38227969
Au	-0.29666569	-0.13805973	-1.60663663
P	1.98362731	-0.03648279	-1.44947154
C	2.75249527	-0.53990965	-3.00607096
C	2.65480303	1.60252724	-1.10207744
C	2.65601483	-1.15656195	-0.20249409
H	2.40057932	-2.18229357	-0.47477587
H	3.74227524	-1.04876954	-0.16152597
H	2.22780312	-0.93511120	0.77442786
H	2.44167551	-1.55586476	-3.25481688
H	3.83984386	-0.50382043	-2.90544267
H	2.43747813	0.13484533	-3.80376511
H	2.34016468	2.29024704	-1.88914639
H	3.74576588	1.54953195	-1.07879300
H	2.28843733	1.96687048	-0.14354503
C	-1.12276047	0.64969568	1.93659488
C	-1.26001015	-0.38763267	2.86034020
C	0.12807798	1.25281133	1.77751380
C	-0.17338494	-0.78599335	3.61323639
H	-2.20268938	-0.89286594	3.02532694
C	1.19115108	0.84087466	2.55065747
H	0.28909255	2.03751887	1.05065644
C	1.06349154	-0.18269185	3.47567619
F	2.40147971	1.41101649	2.40397190
F	2.11281724	-0.58468256	4.20311227
F	-0.29920194	-1.78125042	4.50812929
*			

E16

C	-2.85476970	-0.96940604	-0.40725319
H	-2.08687197	-1.64316918	-0.02596154
H	-3.50965623	-1.55851266	-1.05334554
C	-2.21001921	0.06121447	-1.23280920
C	-1.80965684	0.99249369	-1.91106833
H	-1.66604266	1.83292598	-2.56180008
C	-3.53889514	1.94979728	1.63551030
H	-3.06097282	2.75682175	2.18079519
H	-4.53445681	2.13523382	1.24499843
C	-2.94714228	0.76818646	1.46531650
C	-3.66652767	-0.34865278	0.74536281
H	-3.91091014	-1.15663229	1.44057313
H	-4.60815606	0.03993343	0.35512745
Au	0.09464638	0.10365285	-1.12469384
P	2.30059845	-0.44812291	-0.89682683
C	3.00629825	-0.91543231	-2.49412674
C	3.34050501	0.89315263	-0.28371666
C	2.60471594	-1.85832977	0.18942664
H	2.00242840	-2.70771745	-0.13658309
H	3.66417077	-2.12194880	0.14418699
H	2.33970923	-1.59962365	1.21391047
H	2.46847973	-1.77838539	-2.89060686
H	4.06204340	-1.16791032	-2.37009273
H	2.90950279	-0.08388371	-3.19412935
H	3.26754534	1.74527564	-0.96194917
H	4.37826395	0.55555198	-0.23130306
H	3.00279312	1.19950247	0.70644119
C	-1.56573393	0.51023672	1.95497400
C	-1.17980355	-0.74865484	2.41055841

C -0.61896961 1.54066677 1.96306367
C 0.10561307 -0.97034486 2.89308341
H -1.88837057 -1.56853888 2.41345811
C 0.65653024 1.32440221 2.44982897
H -0.87616145 2.52190513 1.57707042
C 1.02766925 0.06190493 2.92156529
H 0.40115707 -1.94802528 3.25771224
O 1.63054415 2.28705862 2.49308219
H 1.27050438 3.12965195 2.18328066
O 2.28867952 -0.16846056 3.38693589
H 2.78154585 0.66477416 3.37659493
*

E17
C -3.05162592 -0.52785258 -0.35720837
H -2.35261972 -1.20973813 0.12766700
H -3.81411564 -1.14212336 -0.84157646
C -2.33270668 0.21239470 -1.40300304
C -1.85082764 0.91133730 -2.27851663
H -1.63059746 1.57129632 -3.09468507
C -3.35186823 2.82361025 1.04736703
H -2.78535235 3.68330032 1.38851759
H -4.35931099 3.00420532 0.68603522
C -2.85062307 1.58886498 1.08518352
C -3.70853206 0.41552699 0.66875630
H -3.97093719 -0.19034264 1.54089915
H -4.64010768 0.80230651 0.25380908
Au -0.04618474 -0.05543249 -1.35442531
P 2.07114786 -0.85188292 -1.03943076
C 2.62668299 -1.78647784 -2.48349901
C 3.30016596 0.44693992 -0.79899194
C 2.24672693 -1.98299270 0.35682129
H 1.50958000 -2.78300385 0.27335268
H 3.25206991 -2.41117299 0.34168290
H 2.09733360 -1.44925952 1.29434729
H 1.96641980 -2.64161733 -2.63787538
H 3.64852895 -2.13843970 -2.32311764
H 2.59416882 -1.14807902 -3.36786439
H 3.32532585 1.08680480 -1.68278360
H 4.28476628 0.00120807 -0.63994646
H 3.02383567 1.04913485 0.06608854
C -1.45637893 1.33006409 1.53721400
C -1.12623080 0.12719957 2.17972200
C -0.45423133 2.27010015 1.34062236
C 0.15640251 -0.10729470 2.64615857
H -1.89257764 -0.61852324 2.34086990
C 0.83664932 2.05336869 1.82049453
H -0.66777832 3.18702903 0.80417571
C 1.15240794 0.87930288 2.48464252
H 1.59490236 2.80894991 1.66095508
O 2.37768522 0.57742666 2.98961133
C 3.33136557 1.63582854 3.05798293
H 3.63582775 1.97258163 2.06334662
H 2.93244080 2.48204671 3.62519033
H 4.19623874 1.22415699 3.57689949
O 0.55113648 -1.24547707 3.27593367
C -0.38334114 -2.31926314 3.32665672
H -0.71274560 -2.60311218 2.32223760
H 0.14537767 -3.15400855 3.78520558
H -1.25225805 -2.06117043 3.93958111
*

E18
C -1.67383245 -1.17752667 2.49184115
H -0.86637541 -1.70264421 1.97995663
H -2.35324401 -1.93440295 2.89122131
C -2.42113759 -0.37592412 1.51319806
C -3.13080291 0.34552810 0.83158088
H -3.89278371 0.96340818 0.39786260
C -0.72628168 2.12689157 3.49093020
H -0.16854297 3.00695832 3.18854777
H -1.60754666 2.28454201 4.10453219
C -0.34457920 0.89615802 3.14605411
C -1.12845186 -0.30014981 3.63217255
H -0.50410871 -0.93904405 4.26244189
H -1.96071097 0.04826748 4.24551807
Au -1.38133956 -0.02818979 -0.51162584
P -0.04820257 -0.15113713 -2.36464760
C -0.94808400 -0.79463350 -3.79362188
C 0.61372582 1.44289911 -2.89344910
C 1.36093104 -1.25756842 -2.14362568
H 0.99381077 -2.26283036 -1.92887385
H 1.96425093 -1.27464892 -3.05399397
H 1.96236727 -0.91446430 -1.30420443
H -1.32673436 -1.79246537 -3.56597631
H -0.27970689 -0.84494220 -4.65649522
H -1.78944563 -0.13825457 -4.02188495
H -0.21602510 2.10762159 -3.14112569
H 1.24558432 1.30561838 -3.77388285
H 1.19518296 1.88680333 -2.08599666
C 0.82996030 0.65305339 2.26942059
C 1.59607993 -0.50978679 2.36877549
C 1.22416598 1.58592769 1.30628368
C 2.71449845 -0.72331032 1.57848977
H 1.32857332 -1.27494875 3.08964212
C 2.34325311 1.39612700 0.51454674
H 0.63210088 2.48303782 1.15918212
C 3.13253960 0.23665029 0.63854848
H 3.26537942 -1.64578013 1.70191813
H 2.59387999 2.15254925 -0.21617308
N 4.25709784 0.04210435 -0.14135718
C 4.47602701 0.91909736 -1.27983074
C 4.81641384 -1.29701661 -0.23256619
H 5.41117072 0.63786869 -1.76219163
H 3.66885348 0.85745685 -2.02221555
H 4.56991676 1.95855236 -0.95805435
H 5.68676290 -1.26840260 -0.88663607
H 5.15038491 -1.64954910 0.74562831
H 4.09939928 -2.02432229 -0.63774125
*

E19
C -1.05936063 -1.04015040 3.01013427
H -0.32253788 -1.58707914 2.41818284
H -1.29929288 -1.65081076 3.88433547
C -2.30133620 -0.89739953 2.24029234
C -3.42233132 -0.77832445 1.77398194
H -4.47385882 -0.74435002 1.56358825
C -0.73730515 2.19750659 1.85761719
H -0.41335531 2.81444422 1.02667801
H -1.65940314 2.48215830 2.35277101
C -0.03259527 1.15392823 2.29436457
C -0.51313928 0.32676957 3.45810346
H 0.29017068 0.14714196 4.17408808
H -1.29748513 0.87126625 3.98456056
Au -2.15509828 -0.29547180 0.00284049
P -1.61117217 0.02679198 -2.19892798
C -3.01321199 -0.39155461 -3.26023606
C -1.15616632 1.70775374 -2.67837249
C -0.26543730 -1.05398302 -2.73059715
H -0.60065949 -2.09046942 -2.65791592
H 0.00766853 -0.82841749 -3.76369011
H 0.59999284 -0.92024395 -2.08308301
H -3.32015254 -1.42143673 -3.07203497
H -2.72258669 -0.28106615 -4.30769808
H -3.84831316 0.27595032 -3.04115111
H -1.97255495 2.38592986 -2.42371589
H -0.98038573 1.73625435 -3.75625378
H -0.25247934 2.02777735 -2.16158679
C 1.22775478 0.74626606 1.61298152
C 2.25880882 0.11056658 2.29976599
C 1.40115558 0.99470008 0.25351915

C 3.44765060 -0.23316892 1.65808418
H 2.16810591 -0.12121493 3.35238734
C 2.57184440 0.64231180 -0.40742700
H 0.61362536 1.46761523 -0.31318742
C 3.59995648 0.03280177 0.30289518
N 4.84233421 -0.34235552 -0.38681182
O 5.88261034 -0.18401419 0.21610326
O 4.73780417 -0.78256440 -1.51319726
C 4.46044885 -0.90016677 2.43631930
N 5.21178784 -1.44556574 3.11168639
C 2.65559666 0.95285297 -1.81192885
N 2.61997300 1.24051574 -2.92300071
*

E2
C -0.52469576 -1.28020489 2.83720967
H 0.08374747 -1.77743786 2.07982727
H -0.86973111 -2.04646769 3.53598190
C -1.71561128 -0.69905678 2.20337894
C -2.76297539 -0.18464549 1.84605318
H -3.75263176 0.21410731 1.73425479
C 0.32829462 2.14518028 2.84868677
H 0.67662228 2.96746286 2.23321765
H -0.38937967 2.37937365 3.62820040
C 0.77129204 0.89908855 2.68462286
C 0.28371359 -0.20805483 3.58665909
H 1.12220037 -0.71046431 4.07384641
H -0.33754656 0.22165016 4.37369966
Au -1.53210205 -0.09764423 -0.01215134
P -0.94032169 0.12980890 -2.20997172
C -2.32729197 -0.27433366 -3.29594275
C -0.43131518 1.79342565 -2.69135737
C 0.39364687 -0.98543306 -2.69597230
H 0.07203305 -2.01608161 -2.53599950
H 0.63069477 -0.83548535 -3.75180102
H 1.27804775 -0.79026842 -2.08965996
H -2.65417262 -1.29773583 -3.10511344
H -2.01590568 -0.17758720 -4.33873262
H -3.15610808 0.40743279 -3.09788252
H -1.24599451 2.49052538 -2.48758477
H -0.19745828 1.80691333 -3.75843054
H 0.44684090 2.09563032 -2.12174781
C 1.73638507 0.55251293 1.60317497
C 2.64387875 -0.49873482 1.75065209
C 1.75970973 1.27987370 0.40962750
C 3.56376917 -0.80270197 0.75549305
H 2.65442937 -1.09010589 2.65815004
C 2.67957012 0.99697207 -0.58812066
H 1.04252922 2.07634542 0.25072483
C 3.57431636 -0.04551993 -0.40374154
H 4.26607259 -1.61738173 0.88448867
H 2.69160766 1.56904455 -1.50773929
Cl 4.71788932 -0.42267187 -1.66754383
*

E20
C -1.06317208 -0.15692037 3.65356829
H -0.53612714 -1.00907233 3.22206303
H -1.14398684 -0.33823536 4.72865527
C -2.42795654 -0.14451812 3.11689727
C -3.60252059 -0.11843089 2.79064542
H -4.67283407 -0.09130262 2.72494582
C -1.16466983 2.40965460 1.47474177
H -1.19979685 2.70327955 0.43243032
H -1.93499416 2.81636238 2.12086282
C -0.23353228 1.59148013 1.97131941
C -0.30855734 1.16964109 3.42012203
H 0.68989398 1.05988338 3.84817072
H -0.81959625 1.95356588 3.97987195
Au -2.55970469 -0.23294102 0.80351116
P -2.13482951 -0.49444775 -1.42798316
C -3.48170391 -1.32586446 -2.29867018
C -1.88048701 1.06220996 -2.30728609
C -0.66264446 -1.49481230 -1.73154086
H -0.82119124 -2.49645461 -1.32786293
H -0.47017242 -1.55820139 -2.80484025
H 0.19349127 -1.04454580 -1.23087443
H -3.64951239 -2.30850358 -1.85485219
H -3.22167378 -1.43947330 -3.35372938
H -4.39422891 -0.73407093 -2.20911247
H -2.77192852 1.68368957 -2.20550256
H -1.69550148 0.85988965 -3.36467850
H -1.02826013 1.59196153 -1.88302927
C 0.83588811 1.02559643 1.10279088
C 1.55762413 -0.10147712 1.48716880
C 1.13997537 1.60493132 -0.12945732
C 2.51945973 -0.66568475 0.66009166
H 1.37731377 -0.56712502 2.44416857
C 2.08424379 1.05215595 -0.97708185
H 0.64113837 2.51231078 -0.43855078
C 2.76759511 -0.08984223 -0.57652551
N 3.75689416 -0.70713861 -1.48736199
O 4.92134149 -0.65633482 -1.15816746
O 3.32341865 -1.22067121 -2.49646155
C 3.25489251 -1.90233023 1.13800275
F 2.57732594 -2.53132801 2.11184685
F 4.46887797 -1.60486688 1.63242162
F 3.43304000 -2.79561150 0.14550530
C 2.30731524 1.69791156 -2.32925729
F 1.93570657 2.98744948 -2.32556686
F 1.58903275 1.08511414 -3.29037763
F 3.59762336 1.66037322 -2.70812331
*

E21
C -1.13587029 -1.21212232 2.85950199
H -0.45807068 -1.71660517 2.16838841
H -1.50498349 -1.96327620 3.56227350
C -2.29702706 -0.69994580 2.11948949
C -3.33310324 -0.24141632 1.66630696
H -4.32745462 0.10700034 1.46392938
C -0.37834526 2.22997080 2.76626875
H 0.00679181 3.03449470 2.14978436
H -1.18241487 2.47427362 3.45232327
C 0.11974625 0.99540356 2.72263918
C -0.42164168 -0.08547987 3.62516967
H 0.37955294 -0.53988800 4.21160482
H -1.12125624 0.36415098 4.33070641
Au -1.96436379 -0.15171207 -0.09322212
P -1.14721451 0.04725900 -2.22046831
C -2.36259893 -0.48473898 -3.44752171
C -0.70046761 1.73015985 -2.69522530
C 0.30278013 -0.99277165 -2.49973823
H 0.00275530 -2.03937049 -2.41955955
H 0.70892937 -0.80348743 -3.49576995
H 1.06244472 -0.78928935 -1.74707423
H -2.64269224 -1.52203995 -3.25759505
H -1.93324528 -0.39880443 -4.44855799
H -3.25181603 0.14394504 -3.37727640
H -1.59245908 2.35795890 -2.65189028
H -0.30495280 1.72846682 -3.71355165
H 0.04810418 2.13040254 -2.01331715
C 1.21724881 0.64475917 1.77751211
C 2.11741300 -0.37764271 2.08085141
C 1.35543656 1.34191600 0.57374290
C 3.14506746 -0.66626975 1.20792487
H 2.04876245 -0.95564817 2.99270253
C 2.38609497 1.02906718 -0.28203106
H 0.66420911 2.12350873 0.28893931
C 3.29917408 0.02371379 0.01407536
N 4.37043520 -0.31641101 -0.92061482
O 5.49699620 -0.39766854 -0.47621843

O 4.05818737 -0.49810690 -2.08119877
F 2.52286489 1.71429745 -1.42484093
F 3.99195596 -1.65932666 1.50220552
*

E22
C -2.72609289 -1.48560121 1.74651734
H -1.78668482 -1.95478667 1.45169800
H -3.47892120 -2.27489956 1.81218524
C -3.14466492 -0.55201209 0.69220364
C -3.61949646 0.26319669 -0.08124269
H -4.21890444 0.94705337 -0.64973821
C -2.24123712 1.64910421 3.42567572
H -1.64999514 2.55883693 3.41940287
H -3.26423087 1.72462727 3.78048259
C -1.73792193 0.47785934 3.03302858
C -2.58542547 -0.77102865 3.10229660
H -2.15365026 -1.49001425 3.80377533
H -3.57638038 -0.50735562 3.47499925
Au -1.53852085 0.00969573 -0.86530577
P 0.25420405 0.10187180 -2.28207856
C -0.21693138 -0.38681595 -3.95746466
C 0.95322962 1.75856967 -2.44736604
C 1.61730847 -0.99387101 -1.83414144
H 1.25671410 -2.02373139 -1.81094578
H 2.41227096 -0.90124331 -2.57769217
H 1.99758462 -0.72789875 -0.85008380
H -0.58925634 -1.41257035 -3.94518355
H 0.65074050 -0.32082847 -4.61817895
H -1.00520215 0.27342341 -4.32288196
H 0.18854439 2.42814750 -2.84562923
H 1.80600652 1.73262135 -3.12969922
H 1.27086666 2.12405163 -1.47123431
C -0.36133488 0.35061118 2.49076847
C 0.38955271 -0.81542977 2.65905671
C 0.24452750 1.39862607 1.79020671
C 1.68285222 -0.93248397 2.17748669
H -0.03582189 -1.65988538 3.19116090
C 1.53839368 1.30850717 1.30929575
H -0.31486525 2.31040345 1.60854733
C 2.29480929 0.13354694 1.48998788
H 2.22111690 -1.85916195 2.33087613
H 1.96610469 2.15175377 0.78245872
N 3.55884819 0.02129177 0.98877265
C 4.16012408 1.00483588 0.09050336
C 4.36493400 -1.18841793 1.11394020
H 3.43833868 1.34289463 -0.65801800
H 4.50604688 1.88370598 0.64818610
C 5.32628047 0.24490434 -0.54292849
H 4.43960383 -1.50317720 2.15794912
H 3.92274153 -2.01507313 0.54053002
C 5.71409286 -0.76992981 0.53411571
H 4.97993735 -0.27890091 -1.43853252
H 6.14505580 0.90709366 -0.82720560
H 6.31384472 -0.28417509 1.30891807
H 6.27701034 -1.61872445 0.14363040
*

E23
C -0.08205521 3.03848676 1.44413788
H -0.00364271 2.05375839 1.90744262
H -0.58111982 3.69496648 2.16052957
C -0.93308832 2.93273863 0.25015977
C -1.58308164 2.97526682 -0.78165146
H -2.14322156 3.22867731 -1.66065235
C 2.33743677 3.63241863 -1.14651379
H 2.83502539 3.18053053 -1.99784234
H 2.18023368 4.70588784 -1.17511773
C 1.95005895 2.91452236 -0.09256321
C 1.31016767 3.59340741 1.09462877
H 1.93611771 3.48252721 1.98426229
H 1.22979383 4.66081054 0.88483720
Au -1.46110524 0.78078691 -0.33558632
P -1.77140921 -1.47582870 -0.17918552
C -3.51744570 -1.93072079 -0.26676617
C -0.95338267 -2.45768908 -1.45275025
C -1.18629471 -2.08354372 1.41784263
H -1.76848363 -1.60563712 2.20808357
H -1.31185196 -3.16682234 1.47676070
H -0.13585060 -1.82313828 1.54652724
H -4.06867137 -1.41485783 0.52102711
H -3.62138706 -3.01092625 -0.13998412
H -3.92278335 -1.63678775 -1.23645248
H -1.32477898 -2.15256169 -2.43284607
H -1.16941665 -3.51667630 -1.29454933
H 0.12202843 -2.28950182 -1.41291875
C 2.12516477 1.43782505 -0.05730711
C 2.37587349 0.76471749 1.14137526
C 2.08013913 0.67700695 -1.22431020
C 2.61841353 -0.59070441 1.13424038
H 2.43623712 1.28922004 2.08809531
C 2.32176398 -0.68978190 -1.20888282
H 1.84587019 1.15632956 -2.16784314
C 2.61264876 -1.37642585 -0.02490760
H 2.27975321 -1.23601913 -2.14202825
N 2.94853920 -2.73921426 0.01991506
C 3.22316550 -3.37275059 -1.26087529
C 2.11400376 -3.59507451 0.86374898
H 3.64709107 -4.36018231 -1.06803680
H 2.32171330 -3.50349216 -1.87731820
H 3.95375312 -2.78974802 -1.82390154
H 1.15658586 -3.82690656 0.37782041
H 2.64303309 -4.53344065 1.04407848
H 1.91477371 -3.12532472 1.82343448
F 2.92812410 -1.17630647 2.32164223
*

E24
C -3.44185856 0.28264867 -0.85809810
H -3.16151104 -0.59346330 -0.27160736
H -4.52902693 0.26196715 -0.97152016
C -2.86714718 0.13406390 -2.19924789
C -2.50184577 0.03542640 -3.35842786
H -2.39740583 -0.02037160 -4.42445643
C -0.88530322 2.47237739 -0.99157299
H 0.19058891 2.60119845 -0.99371077
H -1.43263501 2.92871079 -1.80932045
C -1.53578572 1.79430668 -0.04196732
C -3.02946925 1.59868696 -0.16398083
H -3.50611305 1.61335833 0.81801551
H -3.44063301 2.42808714 -0.74052491
Au -0.58799343 -0.28033183 -2.22063183
P 1.56417016 -0.82987469 -1.67977721
C 2.35962122 -1.85974260 -2.93327819
C 2.64990931 0.59799756 -1.47085073
C 1.66122489 -1.76624372 -0.13867796
H 1.11427315 -2.70385232 -0.25307141
H 2.70566167 -1.97808012 0.10118148
H 1.20805305 -1.18885949 0.66688934
H 1.78810932 -2.77993526 -3.06442037
H 3.37742947 -2.10177746 -2.61828755
H 2.38940516 -1.32074719 -3.88176207
H 2.66762155 1.17651400 -2.39621845
H 3.66051304 0.25847772 -1.23278740
H 2.27823874 1.22932708 -0.66419536
C -0.81325565 1.16819370 1.10149252
C -1.39880068 0.14844705 1.85394016
C 0.47305286 1.58215598 1.45020997
C -0.70414268 -0.45394803 2.89262698
H -2.40465097 -0.18261717 1.64069992
C 1.15962442 0.96986754 2.48726355
H 0.94193965 2.40021438 0.92020424

C	0.58537262	-0.05959423	3.21726541
H	1.12639388	-0.53889846	4.02431288
C	2.57996445	1.37820985	2.77637569
F	2.82049209	2.66489747	2.46189053
F	3.45899536	0.63573182	2.06025209
F	2.90781582	1.21750041	4.07162268
C	-1.32502450	-1.57825328	3.68036720
F	-1.27440806	-1.34949818	5.00916291
F	-2.61623259	-1.78357896	3.36511547
F	-0.67998267	-2.74822933	3.46939033

*

E3

C	-3.18491452	-0.78787489	-1.66417733
H	-2.77239442	-1.69967026	-1.23057901
H	-4.10415456	-1.06329085	-2.18815290
C	-2.24327146	-0.29816705	-2.67614436
C	-1.55551874	0.11764988	-3.59308272
H	-1.13922395	0.46925084	-4.51682857
C	-1.71363371	1.91299558	-0.29616487
H	-0.81030544	2.32182803	0.14084121
H	-2.14602451	2.46391384	-1.12452579
C	-2.29489210	0.79322261	0.14480461
C	-3.50968270	0.26356406	-0.58166452
H	-4.21396083	-0.19284534	0.11636755
H	-4.01891956	1.09952074	-1.06278773
Au	-0.07022098	-0.03812664	-1.91001991
P	1.89154521	-0.14912491	-0.74194464
C	3.32618648	-0.40591830	-1.81048256
C	2.26679586	1.33610345	0.21411290
C	1.90260627	-1.52216867	0.43098804
H	1.80746926	-2.46075061	-0.11811648
H	2.83828655	-1.52160397	0.99460369
H	1.05963655	-1.42560004	1.11525473
H	3.19832792	-1.33038184	-2.37588333
H	4.23103667	-0.47045294	-1.20155660
H	3.41454864	0.42865881	-2.50822897
H	2.31941981	2.19462191	-0.45779525
H	3.22528067	1.21125875	0.72310300
H	1.48312679	1.50837855	0.95112157
C	-1.73836765	0.01775289	1.29037960
C	-2.09854413	-1.31188104	1.51626937
C	-0.82955902	0.60724933	2.17462764
C	-1.54837109	-2.04042987	2.56575408
H	-2.82040691	-1.80102341	0.87584001
C	-0.28043384	-0.12323125	3.21434353
H	-0.56019450	1.64849001	2.06262649
C	-0.62712747	-1.45557162	3.41851962
H	-1.84333605	-3.07281831	2.71493821
H	-0.19016652	-2.02049946	4.23351646
C	0.76481498	0.50476772	4.09461667
F	0.70728763	1.85051638	4.09761211
F	2.01678670	0.17581881	3.68694389
F	0.66738412	0.10332787	5.37784768

*

E4

C	-2.77415293	-1.29982885	-1.29504697
H	-2.12596060	-1.76688807	-0.55137026
H	-3.41386203	-2.08238190	-1.71072040
C	-1.95600166	-0.77543941	-2.39674271
C	-1.43236226	-0.31719087	-3.39926017
H	-1.16120431	0.02937731	-4.37761923
C	-2.87969208	2.15541105	-0.59792919
H	-2.32295989	2.98818234	-0.18238156
H	-3.51728540	2.36249825	-1.45120059
C	-2.81797932	0.92976630	-0.07884661
C	-3.63712247	-0.19201300	-0.66819031
H	-4.26478352	-0.65627277	0.09528955
H	-4.30218885	0.21581450	-1.43058968
Au	0.20012009	-0.14194908	-1.88905311
P	2.25767943	0.13756579	-0.92885559
C	3.57955744	-0.30325693	-2.07996615
C	2.62921637	1.82380535	-0.40226665
C	2.49432347	-0.92365446	0.51258222
H	2.42654950	-1.96733130	0.20035591
H	3.47630067	-0.73547189	0.95273790
H	1.71666731	-0.72160421	1.24794561
H	3.45352615	-1.33873798	-2.40019745
H	4.54778953	-0.18773582	-1.58708721
H	3.53528870	0.34833010	-2.95431571
H	2.55626761	2.49340969	-1.26096573
H	3.64220706	1.86143574	0.00509202
H	1.91908212	2.14260472	0.35998583
C	-1.93359040	0.62245365	1.08106060
C	-2.26219413	-0.38115189	1.99372045
C	-0.74986872	1.34062291	1.28208232
C	-1.45242342	-0.64433940	3.09070659
H	-3.16698872	-0.96243614	1.86599073
C	0.05712453	1.09180608	2.37832696
H	-0.45325480	2.09780346	0.56671102
C	-0.29723955	0.09854338	3.28573705
H	-1.72920039	-1.42334783	3.79072036
H	0.96933052	1.65979278	2.52106710
C	0.62121059	-0.19930560	4.43684971
F	1.68655176	-0.94588806	4.05215546
F	0.01354643	-0.88138793	5.42555650
F	1.13002477	0.92370997	4.98743566

*

E5

C	-2.68705267	-1.10518600	0.38456871
H	-1.78785591	-1.67381686	0.62502485
H	-3.40084031	-1.80231858	-0.06026142
C	-2.34780392	-0.09330766	-0.62583799
C	-2.20094143	0.81296602	-1.42891701
H	-2.28954698	1.61306051	-2.13786349
C	-3.15460527	1.89882142	2.35386905
H	-2.63516510	2.77235155	2.73385374
H	-4.22077050	1.99330620	2.17351487
C	-2.52287028	0.74660657	2.13314075
C	-3.28719759	-0.46350900	1.64930651
H	-3.31069144	-1.23595987	2.42307409
H	-4.31888144	-0.16922820	1.45167769
Au	-0.09636614	0.12413201	-1.06898922
P	2.14370761	-0.26581897	-1.29940855
C	2.55029730	-0.71466100	-3.00241100
C	3.19999138	1.14556218	-0.91140955
C	2.74073716	-1.63524112	-0.28496254
H	2.17062053	-2.53558384	-0.51977647
H	3.79914409	-1.80619704	-0.49549163
H	2.61283807	-1.39529011	0.77037340
H	1.99772212	-1.61285792	-3.28323147
H	3.62312041	-0.90318667	-3.08690678
H	2.26884873	0.09995007	-3.67182447
H	2.92859809	1.98821575	-1.54958656
H	4.24450167	0.87623153	-1.08450527
H	3.06317126	1.43125023	0.13122268
C	-1.05340077	0.61387145	2.32375729
C	-0.48193200	-0.57769014	2.77746082
C	-0.19828088	1.68176145	2.04371854
C	0.88549765	-0.69382248	2.97522278
H	-1.11375592	-1.43081848	2.99904632
C	1.17098729	1.58280644	2.24064214
H	-0.60839111	2.60612403	1.65256789
C	1.71501659	0.39172585	2.71162851
H	1.31849595	-1.61965696	3.33755615
H	1.81968633	2.42451317	2.01784770
O	3.05558870	0.22916019	2.91050131
H	3.50960134	1.06305668	2.72441987

*

E6

C	-2.74021321	-1.08464308	-0.95369181
H	-2.04636711	-1.65353096	-0.33311197
H	-3.24840366	-1.79494120	-1.61003237
C	-1.97577607	-0.15628818	-1.79837732
C	-1.49178498	0.68134243	-2.54182845
H	-1.26159902	1.41525232	-3.28936730
C	-3.63065016	2.04288319	0.57440703
H	-3.22764919	2.87003092	1.14953016
H	-4.45312566	2.25895933	-0.10015827
C	-3.15533209	0.80429623	0.69857049
C	-3.76482388	-0.33334065	-0.08570068
H	-4.21748822	-1.06618341	0.58769239
H	-4.55946480	0.05975217	-0.72163338
Au	0.25341854	0.09125500	-1.27202003
P	2.38730798	-0.17321612	-0.49530133
C	3.51033799	-0.69775301	-1.81038899
C	3.10886372	1.34079174	0.17261944
C	2.53497211	-1.43429986	0.78815774
H	2.22637691	-2.39703397	0.37667581
H	3.57343815	-1.49529286	1.12199901
H	1.89146365	-1.18918080	1.63159012
H	3.16961761	-1.64951841	-2.22126747
H	4.51973349	-0.81298319	-1.40834722
H	3.51718482	0.05076049	-2.60457024
H	3.15315793	2.09194427	-0.61829958
H	4.11749153	1.13701579	0.53940589
H	2.48953852	1.71858335	0.98520931
C	-1.99580791	0.49680249	1.57839393
C	-1.91239227	-0.71277078	2.27883779
C	-0.95056163	1.40484373	1.72934782
C	-0.84336282	-0.98859986	3.11075012
H	-2.70289669	-1.44935724	2.18528094
C	0.12921858	1.14762443	2.56849531
H	-0.96859665	2.33625403	1.17391997
C	0.18649158	-0.05712949	3.26301594
H	-0.78810838	-1.92627158	3.65291041
H	0.91515207	1.88458893	2.66118862
O	1.20835934	-0.42389857	4.08193532
C	2.29508338	0.48860318	4.22334982
H	2.78228077	0.67405721	3.26123545
H	1.96130011	1.43566262	4.65721791
H	3.00159088	0.00972546	4.90007793

*

E7

C	-2.72831050	-1.15849120	-0.85052857
H	-2.01047583	-1.67873606	-0.21425146
H	-3.26818199	-1.91662417	-1.42280963
C	-2.00062258	-0.31053995	-1.80566277
C	-1.54955872	0.44412955	-2.65194973
H	-1.35319143	1.09247608	-3.48368389
C	-3.43248286	2.07541735	0.48139689
H	-2.98597289	2.91607510	1.00186967
H	-4.22704121	2.29279724	-0.22533813
C	-3.04649190	0.82093884	0.71000617
C	-3.71116968	-0.32978499	-0.00578635
H	-4.17925078	-1.01114650	0.70910403
H	-4.50043042	0.06056928	-0.64997514
Au	0.22493254	0.05760936	-1.34959023
P	2.34821915	-0.06817939	-0.51228333
C	3.54540872	-0.58435564	-1.76324936
C	2.97376462	1.49624504	0.13559629
C	2.48533150	-1.27608449	0.82149595
H	2.24718588	-2.26669013	0.42980664
H	3.50308701	-1.27400156	1.21803555
H	1.77930075	-1.02821079	1.61270696
H	3.26179537	-1.56176841	-2.15685013
H	4.54027501	-0.64466412	-1.31556452
H	3.55612168	0.13942757	-2.57998090
H	3.02179245	2.22501930	-0.67565799
H	3.97231015	1.34785013	0.55311649
H	2.30348255	1.87059732	0.90844562
C	-1.93531832	0.51103358	1.65129607
C	-1.95456304	-0.63616273	2.44609664
C	-0.84251592	1.37593621	1.77221681
C	-0.93164718	-0.89440794	3.34857197
H	-2.78419312	-1.33095097	2.37819581
C	0.17533432	1.11969237	2.67561120
H	-0.79215117	2.26226862	1.14940330
C	0.15340164	-0.02611442	3.47441027
H	-0.97236000	-1.78807439	3.96361739
H	0.99494910	1.82305077	2.76650651
C	1.24907110	-0.35832292	4.40794712
C	2.49453641	0.10304627	4.32794210
H	0.99421817	-1.06532065	5.19426222
H	3.24418798	-0.19848232	5.05198669
H	2.81230139	0.78196524	3.54189424

*

E8

C	-0.45543020	-1.29992373	2.83805659
H	0.18270640	-1.79425503	2.10367892
H	-0.80339989	-2.06337102	3.53845894
C	-1.64143770	-0.75299469	2.16605760
C	-2.69331693	-0.27079568	1.77850734
H	-3.69172983	0.09534891	1.63705814
C	0.34590598	2.14184726	2.82116805
H	0.69906372	2.95805398	2.20045955
H	-0.39112555	2.38014557	3.58116097
C	0.80642445	0.89846893	2.68727416
C	0.31110594	-0.20158670	3.59372353
H	1.14354455	-0.68082434	4.11345734
H	-0.34073111	0.22979337	4.35470427
Au	-1.41626680	-0.13184820	-0.04372466
P	-0.81326989	0.14432021	-2.23380351
C	-2.20062170	-0.23877834	-3.32748237
C	-0.31024032	1.81999650	-2.67910441
C	0.52283144	-0.95461235	-2.75045185
H	0.20945631	-1.99001425	-2.60579032
H	0.74801535	-0.78435083	-3.80581839
H	1.41247791	-0.76408581	-2.15043479
H	-2.52378283	-1.26749975	-3.16031349
H	-1.89099210	-0.11671718	-4.36812419
H	-3.03159149	0.43521181	-3.11263242
H	-1.12373112	2.51104744	-2.45147292
H	-0.08608747	1.85946249	-3.74762493
H	0.57321466	2.10969616	-2.11122225
C	1.79825800	0.54571434	1.63312770
C	2.71440947	-0.49265629	1.81840316
C	1.83361528	1.24918825	0.42504500
C	3.64813843	-0.80660791	0.84277001
H	2.71481347	-1.06287930	2.73942013
C	2.76931551	0.94959686	-0.54967925
H	1.11116310	2.03547212	0.24138914
C	3.68605640	-0.08565851	-0.35137118
H	4.35335611	-1.61362177	1.00508773
H	2.78548021	1.50547887	-1.47993015
C	4.63791820	-0.41941036	-1.37830904
C	5.42451030	-0.70403612	-2.24018865
H	6.12842889	-0.95464109	-3.00520826

*

E9

C	-0.39008150	-1.29752648	2.82856660
H	0.22021510	-1.78999805	2.06942935
H	-0.71668380	-2.06457229	3.53529034
C	-1.59675240	-0.74103755	2.20217042
C	-2.65937451	-0.25050906	1.85650706
H	-3.65981878	0.12363093	1.75568597
C	0.43140578	2.13376876	2.77954992
H	0.77448083	2.94474634	2.14647678

H -0.28864377 2.37934287 3.55327512
C 0.88602074 0.88857678 2.64554373
C 0.40698677 -0.20772156 3.56468227
H 1.24858677 -0.69492845 4.06147513
H -0.21987164 0.22922208 4.34317145
Au -1.45261062 -0.12193584 -0.01377970
P -0.90638783 0.14666792 -2.21959711
C -2.32454701 -0.21984661 -3.27850348
C -0.39119235 1.81470692 -2.67928777
C 0.40327508 -0.96967465 -2.76560352
H 0.07974865 -2.00099126 -2.61431074
H 0.60814809 -0.80199095 -3.82550433
H 1.30811898 -0.79151454 -2.18477025
H -2.65768814 -1.24364302 -3.10110395
H -2.03790136 -0.10417528 -4.32641895
H -3.14083909 0.46600330 -3.04565659
H -1.18623810 2.51863765 -2.42732358
H -0.19892311 1.85076567 -3.75412879
H 0.51372851 2.09037329 -2.13872975
C 1.85994211 0.53408002 1.57513151
C 2.79509394 -0.48798062 1.76056848
C 1.85345761 1.22181534 0.35697677
C 3.71355216 -0.80300402 0.77234323
H 2.82243882 -1.04198562 2.69046781
C 2.77070834 0.92464715 -0.63451510
H 1.11372542 1.99282469 0.18023203
C 3.70236932 -0.09246947 -0.42632293
H 4.43778905 -1.59392058 0.92714159
H 2.76128163 1.46382783 -1.57418118
C 4.63891073 -0.42316188 -1.46748454
N 5.37990360 -0.68927687 -2.30627006
*

F0
C -0.99847226 -1.18119237 1.12677406
H -0.04133353 -1.68061240 1.30685758
H -1.59678017 -1.85619764 0.51113514
C -0.76257254 0.12930309 0.43143911
C -0.95200118 1.27465148 1.08574661
H -0.75062448 2.24288738 0.63733709
C -1.44908540 1.39135338 2.55255417
H -0.97689417 2.24980562 3.02035140
H -2.53369557 1.53328826 2.54737211
C -1.11713260 0.10606919 3.17757969
C -1.75131725 -1.02631363 2.49762275
H -1.72226606 -1.96010725 3.05132541
H -2.78499957 -0.76259923 2.26781110
Au -0.12198803 0.09237225 -1.53276185
P 0.58329567 0.05779645 -3.72990179
C -0.71276155 -0.39050988 -4.91519871
C 1.21171496 1.64944346 -4.32750041
C 1.92673902 -1.11268534 -4.06252860
H 1.59941637 -2.12318597 -3.81017390
H 2.21039487 -1.07525930 -5.11702634
H 2.79040059 -0.85957014 -3.44453509
H -1.09420461 -1.38562818 -4.67836929
H -0.31503732 -0.38602252 -5.93284854
H -1.53376653 0.32553842 -4.84273996
H 0.42767273 2.40452123 -4.24335386
H 1.52594384 1.56626555 -5.37065635
H 2.06166261 1.95734201 -3.71534165
C -0.13706090 -0.04443186 4.20042146
C 0.36274409 -1.32583992 4.52792368
C 0.36454085 1.07851436 4.89658496
C 1.33835502 -1.47039659 5.49139300
H 0.00091899 -2.20263243 4.00849495
C 1.32423396 0.92268755 5.87521761
H -0.01740679 2.06800841 4.68628359
C 1.81496290 -0.34817337 6.16698791
H 1.72875344 -2.45341993 5.72594236
H 1.69477958 1.78622189 6.41439094
H 2.57423214 -0.46650822 6.93271875
*

F1
C -0.74460489 -1.37540696 0.77466385
H 0.29642003 -1.59222853 1.03556231
H -1.08958446 -2.20347759 0.15154174
C -0.82476135 -0.06951402 0.02970146
C -1.34189218 1.00088815 0.63429026
H -1.40452862 1.96739689 0.14254428
C -1.87069930 1.03240056 2.08413143
H -1.60988438 1.98168306 2.54294190
H -2.96331029 0.94663352 2.06522057
C -1.32385630 -0.14282248 2.78616415
C -1.61725790 -1.39411042 2.07277465
H -1.42861631 -2.29713854 2.64538505
H -2.66252671 -1.37293678 1.75712561
Au -0.10467485 0.00619222 -1.90447639
P 0.69581867 0.09029727 -4.06769745
C -0.52985468 0.62391400 -5.29243444
C 2.08951780 1.22608385 -4.30166513
C 1.29863438 -1.49998230 -4.69551525
H 0.48808331 -2.23098812 -4.67162474
H 1.66030322 -1.39153659 -5.72083783
H 2.11110172 -1.85714612 -4.05966978
H -1.37610489 -0.06586179 -5.28413321
H -0.08619941 0.64314208 -6.29076279
H -0.88973191 1.62209782 -5.03553552
H 1.78341412 2.23754831 -4.02742006
H 2.41774158 1.21567437 -5.34377573
H 2.91717715 0.92398648 -3.65699253
C -0.48265308 -0.06695385 3.91941295
C 0.16988226 -1.22353853 4.42154517
C -0.25263836 1.16318357 4.58258642
C 1.00917674 -1.14183883 5.50384982
H 0.02503517 -2.18280288 3.94441635
C 0.57807342 1.22955930 5.67730300
H -0.74302167 2.06541377 4.24513921
C 1.22542496 0.08380145 6.15303951
H 1.50967860 -2.03177468 5.86924894
H 0.73522590 2.17733687 6.17928537
C 2.13768814 0.14941873 7.33743931
H 2.17799929 1.15439538 7.75758168
H 1.80156332 -0.54791685 8.10974800
H 3.14742467 -0.15425402 7.04611343
*

F10
C -0.81977296 -1.57323237 0.90913637
H 0.22933221 -1.81582855 1.10545300
H -1.20892231 -2.35688830 0.25551841
C -0.91248875 -0.22289613 0.25714068
C -1.41420693 0.81109409 0.93213531
H -1.45776756 1.81291160 0.51548095
C -1.96517417 0.74567212 2.38244984
H -1.74747454 1.68218002 2.88707200
H -3.04831771 0.59799269 2.34425863
C -1.31985042 -0.42620426 2.98611860
C -1.61715409 -1.66414559 2.25960036
H -1.35594318 -2.57388297 2.79212618
H -2.67866868 -1.67419279 2.00658938
Au -0.21320222 -0.02505990 -1.67706089
P 0.56859045 0.19664162 -3.83704121
C -0.03694061 1.67475958 -4.69396732
C 2.37344837 0.30639615 -3.96462211
C 0.12097576 -1.17723791 -4.93152794
H -0.96630654 -1.26093375 -4.98346958
H 0.52127657 -1.00996208 -5.93433709
H 0.52499065 -2.10843510 -4.52954072
H -1.12737681 1.64718042 -4.73914894
H 0.36815608 1.71949053 -5.70770247

H 0.26805161 2.56539846 -4.14108833
H 2.72624052 1.16970626 -3.39703620
H 2.67517323 0.41012822 -5.00961536
H 2.82275955 -0.59571754 -3.54479641
C -0.35771798 -0.34527871 4.03230144
C 0.46713518 -1.45250910 4.32624642
C -0.19653155 0.84507500 4.77886716
C 1.42768619 -1.35205693 5.31142705
H 0.37189411 -2.37338223 3.76737574
C 0.74682645 0.94222018 5.78392232
H -0.83735867 1.69526262 4.58647216
C 1.55996957 -0.16958916 6.03244465
H 2.07491309 -2.19361483 5.52792654
H 2.31158916 -0.10596030 6.81383130
C 0.90679694 2.19944120 6.59553797
H 0.70889567 2.00301848 7.65252370
H 1.92792858 2.58109286 6.51647424
H 0.22013774 2.97553190 6.25439167
*

F11
C -1.48515421 -1.01661149 0.79637803
H -0.68630276 -1.74128061 0.98117623
H -2.20565219 -1.49825897 0.13197236
C -0.90306790 0.21884216 0.17569962
C -0.85304382 1.35834238 0.86426444
H -0.37614952 2.25528938 0.48135663
C -1.43701242 1.56216329 2.29611267
H -0.84048716 2.30611174 2.81503049
H -2.46995341 1.90722302 2.21336099
C -1.37958619 0.22088922 2.87665062
C -2.22099575 -0.73924952 2.16132749
H -2.39024115 -1.67265927 2.69007535
H -3.17461762 -0.26580441 1.92403983
Au -0.14867337 0.07522119 -1.74306958
P 0.69640119 -0.09598564 -3.88324592
C -0.27380123 0.78158379 -5.13769971
C 2.37633557 0.55804540 -4.07083364
C 0.80107107 -1.79765239 -4.49882389
H -0.19453836 -2.24562688 -4.50033221
H 1.20702670 -1.80981174 -5.51313131
H 1.44751676 -2.38249903 -3.84162170
H -1.29113454 0.38579639 -5.15267365
H 0.17978398 0.65691185 -6.12387856
H -0.31480388 1.84357043 -4.88798991
H 2.38671830 1.61593301 -3.80130022
H 2.71513140 0.44249809 -5.10312531
H 3.05288782 0.02029900 -3.40379531
C -0.44138176 -0.16510765 3.88694951
C -0.17751540 -1.53008217 4.12160649
C 0.24149429 0.81150873 4.63715714
C 0.76457292 -1.89308152 5.06390129
H -0.68546735 -2.29732485 3.55447433
C 1.16316934 0.43081736 5.59589160
H 0.04386144 1.86634862 4.50536791
C 1.43093435 -0.92288958 5.80339465
H 0.98505489 -2.93970684 5.23654479
H 2.16054022 -1.21520094 6.55290528
O 1.77824132 1.41294777 6.30518733
H 2.39237721 1.02056388 6.94234448
*

F12
C -0.53880314 -1.57979236 0.33679017
H 0.49205905 -1.45965205 0.68646370
H -0.55702981 -2.48810191 -0.26970448
C -0.94552959 -0.38074775 -0.47986807
C -1.81280392 0.49511835 0.03079581
H -2.12635493 1.37901809 -0.51752844
C -2.43727860 0.40802002 1.43606283
H -2.50104492 1.40358027 1.86573631
H -3.45535899 0.00911727 1.35005739
C -1.64051351 -0.53873608 2.24178218
C -1.47500029 -1.83340849 1.56016286
H -1.07776151 -2.62547766 2.18748096
H -2.44732312 -2.13671537 1.16472088
Au -0.11244748 -0.14128584 -2.35409783
P 0.82350289 0.12209659 -4.44794643
C -0.24669247 -0.40391678 -5.81352894
C 1.26606457 1.83222438 -4.85571083
C 2.35849918 -0.81089315 -4.69300320
H 2.16406794 -1.87540870 -4.54819956
H 2.74691354 -0.64616028 -5.70087258
H 3.10075608 -0.48799015 -3.96035990
H -0.49373862 -1.46077145 -5.69525422
H 0.25703307 -0.25338629 -6.77139710
H -1.17171135 0.17568043 -5.79431487
H 0.37171924 2.45738934 -4.82017923
H 1.70610356 1.88355475 -5.85448089
H 1.98371285 2.20624096 -4.12289893
C -0.99255442 -0.20053237 3.44484167
C -0.10188931 -1.11082611 4.07658837
C -1.18803059 1.06245646 4.06330601
C 0.55743573 -0.77376453 5.22915493
H 0.07908603 -2.08434316 3.64224484
C -0.53589144 1.38598020 5.22582222
H -1.86721769 1.78365753 3.63054055
C 0.35338071 0.48162316 5.83089327
H 1.23736412 -1.48760525 5.67779015
H -0.70230468 2.35183374 5.68893964
C 1.03842135 0.87483177 7.07057414
C 1.35610932 -0.16440043 8.12961738
C 2.46320554 0.41852968 7.32789834
H 0.78004156 1.86246760 7.43072428
H 1.05407140 -1.18395935 7.92297798
H 1.23905753 0.15529891 9.15766197
H 3.11584054 1.14348925 7.79829339
H 2.92532901 -0.19767685 6.56608470
*

F13
C -1.75910718 -0.72841065 0.49338093
H -1.13340168 -1.59815385 0.71616741
H -2.51726242 -1.05510993 -0.22136043
C -0.90721122 0.37087866 -0.06862965
C -0.67094483 1.47037002 0.64565243
H -0.00486958 2.25865635 0.30886284
C -1.28257463 1.77077039 2.04895133
H -0.57864587 2.36941967 2.61883999
H -2.21599284 2.32278859 1.91899603
C -1.53870326 0.43963850 2.59779157
C -2.51625397 -0.31367235 1.81291577
H -2.91261034 -1.19635995 2.30608031
H -3.33049187 0.35114558 1.52298326
Au -0.09551528 0.10736464 -1.95114655
P 0.80760448 -0.18421826 -4.05440032
C -0.01993324 0.77168219 -5.35299604
C 2.55166000 0.29188786 -4.18231596
C 0.75908435 -1.89536609 -4.64942964
H -0.27691378 -2.23746711 -4.68866464
H 1.20236424 -1.96302127 -5.64574543
H 1.31351959 -2.53567424 -3.96062426
H -1.07118671 0.48165355 -5.40404678
H 0.45524515 0.58921682 -6.31981001
H 0.03860957 1.83533580 -5.11408097
H 2.66016015 1.34657833 -3.92203923
H 2.91568174 0.12889177 -5.19948892
H 3.14338899 -0.30373129 -3.48451379
C -0.75854048 -0.14727337 3.64372923
C -0.83391829 -1.53280212 3.90135704
C 0.11339361 0.63573589 4.42695810
C -0.05499505 -2.11531290 4.88453505

H -1.49242865 -2.16059714 3.31649205
C 0.87523639 0.05100404 5.41944578
H 0.18552816 1.70572828 4.27856998
C 0.79572892 -1.32616043 5.64553295
H -0.11460904 -3.18215156 5.06154685
H 1.40545500 -1.76617976 6.42708750
C 1.77692144 0.90455660 6.23731458
O 2.48721947 0.47832527 7.11821449
H 1.75993907 1.98036911 5.98540462
*

F16
C -0.36512617 -1.58536488 0.58121637
H 0.70289568 -1.54239067 0.82083393
H -0.50264754 -2.46049225 -0.05807901
C -0.77806039 -0.32771381 -0.14147597
C -1.52561587 0.57483484 0.49755146
H -1.84270761 1.49636499 0.01699576
C -1.98858982 0.46828386 1.95831590
H -1.92436764 1.44573192 2.42905380
H -3.04071839 0.15650420 1.98021973
C -1.19744806 -0.57596676 2.64733759
C -1.17942617 -1.83916106 1.88279528
H -0.77785851 -2.68893573 2.42656170
H -2.20774793 -2.06036563 1.58461777
Au -0.14179246 -0.05941441 -2.08698725
P 0.57257516 0.22925769 -4.26351514
C -0.39017796 1.43933601 -5.21002424
C 2.28673668 0.79998663 -4.41534928
C 0.51044784 -1.27959013 -5.26690963
H -0.51616130 -1.64938801 -5.30335060
H 0.85998147 -1.07658797 -6.28204769
H 1.14190864 -2.04504404 -4.81155017
H -1.43497457 1.12457975 -5.24572461
H -0.00074144 1.52372739 -6.22738984
H -0.33487583 2.41235629 -4.71793188
H 2.39656133 1.75988679 -3.90697166
H 2.55878534 0.91260708 -5.46756825
H 2.95368620 0.07596366 -3.94316715
C -0.45800776 -0.35713811 3.81651378
C 0.39790089 -1.36808692 4.33204562
C -0.52115834 0.88878697 4.51108267
C 1.16033857 -1.15259104 5.44962219
H 0.47114381 -2.32421015 3.83461365
C 0.22422282 1.09750371 5.63224018
H -1.17356952 1.68139416 4.16942938
C 1.08290072 0.07670763 6.10924520
H 1.82366605 -1.91459036 5.83998013
O 1.82015785 0.27779526 7.19431653
H 1.66450489 1.17403373 7.53909309
O 0.23491723 2.23521931 6.37932220
H -0.36605048 2.89526647 6.00408805
*

F17
C -0.11417040 -1.34727007 0.00488462
H 0.87963290 -1.01839148 0.32857464
H 0.04202497 -2.23934118 -0.60610117
C -0.77728606 -0.25861169 -0.80350756
C -1.77123460 0.44157186 -0.25132509
H -2.28044448 1.23153874 -0.79705698
C -2.28363390 0.27442393 1.18296267
H -2.46380784 1.25428958 1.61727980
H -3.24501240 -0.25635708 1.16115962
C -1.34156612 -0.55649088 1.97158084
C -0.95107611 -1.78063277 1.23933695
H -0.40932124 -2.50815569 1.83680656
H -1.86706600 -2.24560818 0.86322273
Au -0.10809720 0.09871213 -2.72279616
P 0.63360096 0.49272702 -4.87358305
C -0.60213793 0.15492236 -6.15683425
C 1.14688407 2.20367226 -5.18475300
C 2.06873568 -0.50326735 -5.35907067
H 1.81599683 -1.56300434 -5.28783436
H 2.36599286 -0.26868949 -6.38400472
H 2.90064829 -0.29596427 -4.68310171
H -0.90894592 -0.89126749 -6.09905931
H -0.18845013 0.35841716 -7.14746577
H -1.47804253 0.78559643 -5.99231769
H 0.30606920 2.87463312 -4.99817548
H 1.48162905 2.31916558 -6.21842774
H 1.96144844 2.46760450 -4.50741961
C -0.81274732 -0.18602513 3.21007711
C 0.18162374 -0.98743909 3.85876297
C -1.24790764 0.99273104 3.86753612
C 0.70618112 -0.62750847 5.06483622
H 0.53592926 -1.88530626 3.37837162
C -0.74016063 1.35760315 5.08806832
H -2.00575963 1.62056601 3.42190109
C 0.23969853 0.56628213 5.70372214
H -1.09561069 2.25550725 5.57318772
O 0.79116322 0.83627566 6.86892656
C 0.38115655 2.02274111 7.57725108
H -0.68131803 1.96947975 7.82046324
H 0.97336707 2.02925498 8.48940149
H 0.59836511 2.91181901 6.98287197
O 1.65338542 -1.30363922 5.74839707
C 2.15033086 -2.50214139 5.15034926
H 2.89570783 -2.89103548 5.84184645
H 1.34891643 -3.23583083 5.02351823
H 2.61952740 -2.29053316 4.18502073
*

F18
C -1.06002332 -1.17928351 0.23476482
H -0.06022819 -1.53527055 0.50991087
H -1.49771405 -1.96146530 -0.39073038
C -0.94207873 0.11070955 -0.54586013
C -1.27584200 1.25860308 0.05281966
H -1.21371059 2.20550236 -0.47825388
C -1.72560982 1.40243073 1.50362677
H -1.24007640 2.26915466 1.94671245
H -2.80667594 1.59769341 1.53177128
C -1.48606098 0.14547269 2.26958913
C -1.93380239 -1.04872409 1.50285352
H -1.91882452 -1.97287370 2.07430615
H -2.96317772 -0.86087369 1.18030791
Au -0.25869984 0.03907142 -2.49111426
P 0.50175450 -0.03952721 -4.67074209
C -0.74904015 0.39232449 -5.91090768
C 1.88360391 1.08160203 -5.02041483
C 1.10402336 -1.66851048 -5.19226701
H 0.29976996 -2.40082947 -5.09781872
H 1.44446199 -1.63442522 -6.22998294
H 1.93150488 -1.97312947 -4.54842275
H -1.59111587 -0.29825695 -5.83261916
H -0.32495989 0.33594978 -6.91633697
H -1.11015026 1.40580109 -5.72525166
H 1.57659604 2.10962555 -4.81796525
H 2.19188491 0.99339023 -6.06497095
H 2.72555341 0.83396595 -4.37094212
C -0.87143113 0.09341769 3.50119772
C -0.63186926 -1.15376829 4.17972695
C -0.43278061 1.27838247 4.19182867
C -0.02973666 -1.21966372 5.38961524
H -0.93671096 -2.08086885 3.71446027
C 0.16450604 1.23368020 5.40512266
H -0.58479233 2.24842896 3.73885788
C 0.39439724 -0.02317046 6.06324630
H 0.12837829 -2.18286247 5.85239254
H 0.46964779 2.15437131 5.88078077
N 0.97490608 -0.07662906 7.25120622

C 1.20220843 -1.36057423 7.91931221
C 1.40242570 1.14721124 7.93376918
H 1.68078053 -1.17450355 8.87662111
H 0.25560497 -1.87562941 8.09416139
H 1.85463056 -1.99677516 7.31804858
H 1.86018758 0.87626126 8.88096093
H 2.13564342 1.68659606 7.33114720
H 0.54699161 1.79689794 8.12934596
*

F2
C -1.11096894 -1.36144361 0.48661768
H -0.11332988 -1.67263936 0.81173586
H -1.48791135 -2.14440708 -0.17480210
C -1.02206230 -0.04057853 -0.22249813
C -1.50787171 1.05967828 0.35113157
H -1.42081143 2.04253953 -0.10200736
C -2.22601537 1.10183972 1.72950955
H -2.00260274 2.04510961 2.21863462
H -3.30492204 1.02467344 1.56906184
C -1.73954427 -0.08327139 2.44330006
C -2.05970461 -1.32750852 1.74072181
H -1.93172332 -2.22950636 2.33150871
H -3.08348095 -1.26958967 1.36840564
Au -0.10808680 0.00859028 -2.07512145
P 0.90575913 0.05680979 -4.14742120
C -0.20421147 0.52362915 -5.50224776
C 2.28863201 1.22174173 -4.27483986
C 1.60426490 -1.53424594 -4.66350774
H 0.81236479 -2.28505338 -4.69702410
H 2.06274197 -1.44483670 -5.65124781
H 2.35803619 -1.85230503 -3.94061193
H -1.03080822 -0.18753692 -5.55641416
H 0.33636908 0.52617014 -6.45179221
H -0.61061496 1.51907676 -5.31286634
H 1.93278363 2.23226501 -4.06472963
H 2.72006768 1.18954257 -5.27817032
H 3.05406602 0.96056781 -3.54153203
C -0.87806604 -0.02698887 3.57678128
C -0.21984942 -1.19078234 4.03567964
C -0.65076997 1.18886538 4.25895184
C 0.63924128 -1.14044454 5.11010581
H -0.36597516 -2.13888355 3.53688640
C 0.19265118 1.24340666 5.34634602
H -1.15240965 2.09494567 3.94981631
C 0.83224351 0.07758298 5.75623952
H 1.15378067 -2.02852229 5.45375852
H 0.35784233 2.17272540 5.87596790
Cl 1.89590832 0.14192023 7.11568952
*

F22
C -1.28039238 0.84146793 -0.37982005
H -1.50747213 -0.17346980 -0.03261125
H -2.11051104 1.13092847 -1.02933862
C 0.01593064 0.83339603 -1.15959759
C 1.11260567 1.34234921 -0.58858633
H 2.06037159 1.36523413 -1.12149555
C 1.20044278 1.89014036 0.83190426
H 2.11027820 1.52114551 1.30082370
H 1.28589074 2.98519890 0.79525232
C -0.02694804 1.56764959 1.61736507
C -1.26116056 1.81142589 0.82125097
H -2.17981695 1.73066211 1.39595177
H -1.19457734 2.83060791 0.42572490
Au 0.03260313 0.03393936 -3.06142248
P 0.05238428 -0.85129208 -5.19470951
C 0.82607459 0.21997552 -6.43681890
C 0.94228898 -2.42426929 -5.34488107
C -1.59332482 -1.20288504 -5.87073167
H -2.17755761 -0.28113384 -5.90344010
H -1.51088212 -1.61680683 -6.87860561
H -2.10619564 -1.91861692 -5.22519057
H 0.29046405 1.17031193 -6.48117757
H 0.80268458 -0.25697878 -7.41962147
H 1.86194060 0.41584777 -6.15278778
H 1.98085609 -2.28455929 -5.03838467
H 0.91270664 -2.78234213 -6.37678360
H 0.48145458 -3.16601802 -4.68951121
C -0.01448602 1.05454077 2.89357034
C -1.22971103 0.70376699 3.58592934
C 1.21129847 0.83480785 3.62052806
C -1.23060396 0.18979171 4.83626745
H -2.18205779 0.83975167 3.09221652
C 1.23119366 0.32528841 4.87244845
H 2.15598899 1.09662421 3.16396131
C 0.00572798 -0.02556777 5.53838854
H -2.16606036 -0.07463899 5.30913096
H 2.17389787 0.19268051 5.38459120
N 0.01595488 -0.52703440 6.75475066
C 1.24228418 -0.84489304 7.51219733
C -1.19680695 -0.84623566 7.53326421
H 1.94419372 -1.38840581 6.88056137
H 1.71008984 0.08546046 7.84605343
C 0.73086359 -1.67334647 8.68638339
H -1.90256361 -0.01735784 7.48743773
H -1.66846967 -1.73927019 7.11400872
C -0.66185455 -1.09701376 8.93970420
H 0.65689899 -2.72405722 8.39424855
H 1.39166877 -1.59864995 9.54994401
H -0.58556673 -0.15068971 9.48135231
H -1.30999721 -1.76830036 9.50295592
*

F23
C -0.41677754 -1.44090800 0.03525789
H 0.62423528 -1.32684931 0.35934033
H -0.44508099 -2.34258552 -0.58167856
C -0.83104249 -0.23538815 -0.77793799
C -1.65802163 0.65986117 -0.22886217
H -1.98659924 1.53485932 -0.78449433
C -2.19346535 0.61006955 1.20021287
H -2.15610317 1.60701502 1.63348980
H -3.25058346 0.31111964 1.18295532
C -1.46159401 -0.40661864 2.00645823
C -1.32164035 -1.68743880 1.26578956
H -0.93519140 -2.50722969 1.86537638
H -2.31463576 -1.96485610 0.89815314
Au -0.08551498 -0.01749704 -2.68955352
P 0.74939706 0.23558642 -4.82817350
C -0.18097072 -0.65550253 -6.10429607
C 0.78170477 1.95116674 -5.41487806
C 2.45656333 -0.33992478 -5.03520374
H 2.51478664 -1.40162795 -4.78736171
H 2.78727001 -0.18862061 -6.06554976
H 3.11105403 0.21312599 -4.35861061
H -0.17673673 -1.72352058 -5.87779334
H 0.26626100 -0.49041437 -7.08748649
H -1.21464827 -0.30410796 -6.11160950
H -0.23229116 2.35591728 -5.40938726
H 1.18707684 1.99985241 -6.42835816
H 1.40138170 2.55212938 -4.74642689
C -0.91326105 -0.16382695 3.24782345
C -0.19257700 -1.17745703 3.96859302
C -1.03102467 1.10537387 3.90730743
C 0.36580499 -0.92569629 5.16577914
H -0.09661739 -2.18291907 3.58216890
C -0.48001689 1.34893859 5.12037516
H -1.56840084 1.91167512 3.42847950
C 0.29095365 0.35759037 5.81835036
H -0.58167395 2.33201495 5.55499893
N 0.86438322 0.63231765 6.97747815

C 1.89908705 -0.19675798 7.61123431
C 0.55699078 1.87921924 7.68513481
H 2.53002401 0.46796318 8.19901641
H 1.45079705 -0.94091162 8.27182790
H 2.51700870 -0.68312724 6.86332805
H 0.82777155 1.74451593 8.73018635
H 1.13064409 2.71240705 7.27100733
H -0.50775387 2.09935632 7.63165315
F 0.96602602 -1.95111097 5.81507924
*

F3
C 0.45124112 -2.05559403 -0.15534106
H 1.44129164 -1.63432432 0.04404705
H 0.57596492 -2.81320540 -0.93171902
C -0.47235033 -0.95925000 -0.59155894
C -1.46989208 -0.56170625 0.19671147
H -2.10501887 0.28702077 -0.03648389
C -1.85614361 -1.23460929 1.55450049
H -2.31185280 -0.48822909 2.19728906
H -2.56107267 -2.04522821 1.36299641
C -0.57130364 -1.74705573 2.01890474
C -0.03205958 -2.79452016 1.15253167
H 0.79018678 -3.35883760 1.58276897
H -0.83954241 -3.46848857 0.86487987
Au -0.12545421 -0.07117517 -2.42576463
P 0.25900310 0.91017751 -4.47824219
C -1.22617952 1.09733916 -5.49982932
C 0.95737425 2.57981054 -4.38261521
C 1.41820488 -0.00937928 -5.52488272
H 1.02753576 -1.01274359 -5.70523879
H 1.55665832 0.50390746 -6.47941000
H 2.37972684 -0.09409018 -5.01480861
H -1.66940404 0.11615349 -5.68071730
H -0.97281673 1.56392311 -6.45476923
H -1.95341455 1.71853782 -4.97341740
H 0.27189642 3.22906292 -3.83447652
H 1.11548275 2.98247106 -5.38597399
H 1.90977776 2.54642424 -3.84998024
C 0.18880837 -1.13995450 3.07330246
C 1.55618917 -1.43842350 3.22743082
C -0.41478386 -0.22803397 3.96216595
C 2.30295374 -0.82932991 4.21879251
H 2.04125884 -2.13470913 2.55686951
C 0.33634786 0.35523011 4.95849959
H -1.46757820 0.00131471 3.88585832
C 1.69491519 0.06422787 5.08902760
H 3.35742997 -1.05308106 4.32160055
H 2.27433025 0.53357330 5.87691287
C -0.28855974 1.31625277 5.93712712
F 0.33910231 2.51097337 5.92645526
F -0.21373581 0.85431435 7.20333037
F -1.58615645 1.54855178 5.67765347
*

F5
C -1.15662242 -0.93603682 0.87326279
H -0.28541607 -1.55813752 1.10591410
H -1.82028350 -1.55224127 0.26231675
C -0.72201393 0.29293278 0.11420218
C -0.72681230 1.47446891 0.73551785
H -0.42215427 2.38610580 0.22864828
C -1.09745900 1.70063335 2.20735114
H -0.41051258 2.41926063 2.64593896
H -2.10624497 2.13061302 2.26264002
C -1.13126588 0.40241889 2.91888316
C -1.92543624 -0.60230082 2.18367190
H -2.13786318 -1.50650256 2.74602800
H -2.86968034 -0.13259279 1.89479215
Au -0.12446817 0.08629330 -1.85094444
P 0.53743042 -0.13788776 -4.05150158
C -0.41978117 -1.35468150 -4.99510807
C 0.40552430 1.38849094 -5.02092864
C 2.26086500 -0.65861770 -4.26522041
H 2.41090482 -1.62583954 -3.78152526
H 2.50421898 -0.74168890 -5.32717579
H 2.92234811 0.07290923 -3.79696098
H -0.32358404 -2.33564328 -4.52556770
H -0.05847143 -1.40760285 -6.02490793
H -1.47329894 -1.06827108 -4.99380076
H -0.63120427 1.73080669 -5.01963100
H 0.73012839 1.21579351 -6.04985325
H 1.02952442 2.16147969 -4.56801533
C -0.41043765 0.12597093 4.08604042
C -0.39269158 -1.18636168 4.64646422
C 0.33986501 1.13637928 4.75588653
C 0.32528974 -1.47259951 5.77121536
H -0.94519024 -1.98523213 4.17244908
C 1.05107342 0.86237862 5.88976886
H 0.34600824 2.14952969 4.37937703
C 1.05249760 -0.44747007 6.40227929
H 0.34713306 -2.47040377 6.19185367
H 1.61397210 1.63736209 6.39870308
O 1.73202690 -0.77298704 7.49745442
H 2.19901674 0.00204913 7.85167161
*

F6
C -1.04766828 -1.22859977 0.37847776
H -0.07169164 -1.64008571 0.65842280
H -1.51392856 -1.96491240 -0.28033837
C -0.86066391 0.08511370 -0.33950621
C -1.18372410 1.22000799 0.28523455
H -1.06220142 2.18792353 -0.19354321
C -1.70869473 1.32071382 1.72433152
H -1.26152904 2.18183033 2.21322062
H -2.79442482 1.47956282 1.70146742
C -1.45787588 0.04103465 2.42728680
C -1.94553620 -1.11290610 1.64332060
H -1.96200409 -2.05144757 2.18948031
H -2.95879043 -0.88108715 1.30443470
Au -0.07912061 0.06101180 -2.24997202
P 0.80483680 0.03115029 -4.38267465
C -0.37600679 0.46367762 -5.68884578
C 2.18603017 1.17978508 -4.62915997
C 1.46425538 -1.57992958 -4.88949278
H 0.66818387 -2.32626537 -4.85407735
H 1.86562483 -1.52436807 -5.90424298
H 2.25680947 -1.88093511 -4.20155484
H -1.20911819 -0.24185549 -5.67390514
H 0.11019855 0.43158022 -6.66685447
H -0.76501132 1.46788227 -5.50929964
H 1.84947762 2.19952950 -4.43187905
H 2.55926238 1.11218201 -5.65386380
H 2.99061479 0.93615766 -3.93257967
C -0.74518666 -0.07476926 3.62328181
C -0.43116356 -1.35381582 4.17965782
C -0.29407887 1.07313260 4.33599148
C 0.27962249 -1.46878268 5.33462684
H -0.74687406 -2.25691622 3.67614274
C 0.40881072 0.97122266 5.50525580
H -0.51491038 2.06326071 3.96258979
C 0.70765545 -0.30742807 6.01743577
H 0.52645762 -2.43654576 5.75401573
H 0.72760651 1.86497784 6.02262747
O 1.38244753 -0.51796630 7.13306058
C 1.84797264 0.61224174 7.89658913
H 1.00341603 1.21699941 8.23109132
H 2.36336333 0.18598149 8.75422396
H 2.54152382 1.21055138 7.30356475
*

F7
C -1.35622481 -0.90598817 0.56940878
H -0.51678831 -1.54509536 0.86250578
H -1.99732531 -1.51015682 -0.07629978
C -0.84670642 0.30797428 -0.16084443
C -0.87898844 1.49711200 0.44190587
H -0.50996743 2.39807444 -0.03981497
C -1.38458231 1.75087833 1.87646129
H -0.75075683 2.49010349 2.35722931
H -2.40366820 2.15314804 1.82724418
C -1.43596771 0.45793378 2.58334632
C -2.20228467 -0.54913533 1.83504882
H -2.44112194 -1.44608909 2.39807237
H -3.12527806 -0.08337171 1.48211694
Au -0.09881748 0.06596738 -2.07054876
P 0.73464311 -0.20378265 -4.20604622
C -0.18403858 0.70088330 -5.48042360
C 2.44964503 0.34740605 -4.41009239
C 0.74056382 -1.92073736 -4.78792520
H -0.27952167 -2.30972561 -4.78257998
H 1.14590729 -1.97726122 -5.80097653
H 1.35076340 -2.52955238 -4.11793570
H -1.22240785 0.36375854 -5.48776139
H 0.26092251 0.52998926 -6.46361126
H -0.16458716 1.76855942 -5.25328374
H 2.52199842 1.40836518 -4.16296866
H 2.77941864 0.19063242 -5.43988449
H 3.09514922 -0.21440470 -3.73215372
C -0.71488889 0.17359414 3.76486158
C -0.67130436 -1.14246955 4.28868559
C -0.00219864 1.18597295 4.45952546
C 0.05299733 -1.42415103 5.42110677
H -1.19780140 -1.94626186 3.79339240
C 0.70590101 0.89907215 5.59860240
H -0.02042929 2.20696133 4.10509754
C 0.75048772 -0.41283194 6.10054140
H 0.08605846 -2.43769296 5.80422685
H 1.23447356 1.69524997 6.10661117
C 1.49738298 -0.77503205 7.31350261
C 2.16633891 0.07626333 8.08868244
H 1.47690608 -1.83027381 7.57068725
H 2.68940249 -0.28182811 8.96891416
H 2.21828454 1.14070763 7.88673338
*

F8
C -1.06573325 -1.36579709 0.58285226
H -0.06644487 -1.67886079 0.90091908
H -1.44848765 -2.14713132 -0.07715083
C -0.98048635 -0.04366117 -0.12399054
C -1.45879685 1.05632689 0.45621040
H -1.37349000 2.04008533 0.00467060
C -2.16475188 1.09699520 1.84113619
H -1.93282589 2.03759017 2.33136861
H -3.24524958 1.02500870 1.68893210
C -1.67755949 -0.09202044 2.54730758
C -2.00663116 -1.33280674 1.84354554
H -1.87716028 -2.23694900 2.43065114
H -3.03230083 -1.27073955 1.47714693
Au -0.08734339 0.00853418 -1.98675230
P 0.89730706 0.06085007 -4.07292717
C -0.22614832 0.55550901 -5.40661492
C 2.29481847 1.20694700 -4.21093617
C 1.56483322 -1.53537706 -4.61327185
H 0.76167421 -2.27434225 -4.64141722
H 2.00979892 -1.44378087 -5.60697395
H 2.32457926 -1.87061622 -3.90453705
H -1.06346181 -0.14346907 -5.45438345
H 0.30009051 0.55887182 -6.36418504
H -0.61552442 1.55483154 -5.20251475
H 1.95687939 2.22043579 -3.98605947
H 2.71045733 1.17799258 -5.22100467
H 3.06735293 0.92777965 -3.49184212
C -0.80689483 -0.04219227 3.67555714
C -0.14862545 -1.20943312 4.12456316
C -0.57184598 1.17023837 4.36064853
C 0.71757102 -1.16227144 5.19243063
H -0.30210854 -2.15434531 3.62192365
C 0.28051791 1.21487527 5.44065818
H -1.07478856 2.07783740 4.05801522
C 0.93472924 0.05007789 5.86050286
H 1.22967267 -2.05661579 5.52456280
H 0.44909096 2.14473094 5.96916564
C 1.82764087 0.09664887 6.98267263
C 2.57552374 0.13392743 7.92030975
H 3.24129399 0.16854352 8.75740582
*

G0
C -1.82130930 -1.11713531 0.92192579
H -1.71910363 -2.06752562 0.39624452
H -2.81182266 -0.73035424 0.63969739
C -0.82679178 -0.12260109 0.42957490
C -0.40134111 0.69423446 1.48467810
H 0.47397674 1.32547257 1.43236620
C -1.56093492 1.24971653 2.39230811
H -1.27480205 2.19724750 2.83058444
H -2.55778446 1.20090476 1.97249185
C -0.90664810 0.04237982 2.84850522
C -1.61639052 -1.23979589 2.43963739
H -0.98307295 -2.10024265 2.66026011
H -2.55282895 -1.35434453 2.98925005
Au -0.15984633 -0.01581882 -1.48920475
P 0.58581058 0.11200049 -3.68117321
C -0.49218263 1.10836079 -4.74031021
C 2.22907809 0.85172133 -3.84738203
C 0.70358349 -1.49649995 -4.50230181
H -0.27744255 -1.97517694 -4.50620080
H 1.05093301 -1.36851162 -5.53018141
H 1.40406059 -2.13243925 -3.95785751
H -1.49375379 0.67454140 -4.75089807
H -0.09564178 1.13506863 -5.75793327
H -0.55158562 2.12431349 -4.34564084
H 2.21504773 1.86360987 -3.43828829
H 2.51946107 0.88779357 -4.89991236
H 2.95433867 0.25611486 -3.28991041
C 0.02507874 0.04664900 4.01311234
C 1.12701430 -0.80972383 4.02371075
C -0.20093307 0.87817080 5.10690606
C 1.99163275 -0.82859999 5.10861956
H 1.31174429 -1.45583110 3.17133649
C 0.66355174 0.85747510 6.19653320
H -1.06075133 1.53933479 5.11167849
C 1.76079038 0.00582271 6.19903701
H 2.84907372 -1.49297946 5.10419697
H 0.47616230 1.50683259 7.04495167
H 2.43608748 -0.00960647 7.04777282
*

G1
C -2.01895266 -1.12782993 0.51546205
H -1.86693859 -2.07200805 -0.00888751
H -2.99570032 -0.75379460 0.17403141
C -1.01095805 -0.11060249 0.10034775
C -0.69057877 0.71990760 1.17843139
H 0.17040449 1.37260550 1.19110180
C -1.91306641 1.22774290 2.02713414
H -1.67913874 2.17656837 2.49273645
H -2.88142313 1.15889709 1.54737272
C -1.26008365 0.03225680 2.51026908
C -1.91023010 -1.26102032 2.04182328
H -1.27286774 -2.10921459 2.29513297

H -2.87709944 -1.40329083 2.52904522
Au -0.18028965 -0.00522234 -1.75429854
P 0.76045642 0.11726926 -3.86979147
C -0.00462322 1.37541847 -4.92240806
C 2.52431627 0.52246919 -3.85164626
C 0.63838713 -1.42513377 -4.80855008
H -0.41243101 -1.69616662 -4.92661001
H 1.09647033 -1.30391903 -5.79292119
H 1.14899612 -2.22219668 -4.26488510
H -1.06620927 1.15206311 -5.04341432
H 0.47924704 1.38843181 -5.90180206
H 0.09866116 2.35497934 -4.45199229
H 2.67040589 1.48756690 -3.36300905
H 2.90904358 0.56887049 -4.87308038
H 3.06722109 -0.24173340 -3.29237127
C -0.39092556 0.04406506 3.72050840
C 0.70700292 -0.81039591 3.80003635
C -0.66390283 0.88453750 4.79821448
C 1.51571546 -0.81842191 4.92816526
H 0.93847379 -1.46770569 2.96771368
C 0.14545369 0.87076044 5.92604306
H -1.52114910 1.54821336 4.76058052
C 1.24872434 0.02146842 6.00912172
H 2.37026332 -1.48651699 4.97001902
H -0.08568506 1.52920298 6.75811068
C 2.12548099 0.02160120 7.23387030
H 2.89776134 -0.74667404 7.16517380
H 2.61770574 0.99044912 7.35823518
H 1.53549242 -0.16286353 8.13565723
*

G10
C -1.87271468 -1.21580906 0.59816383
H -1.76402529 -2.14387289 0.03544256
H -2.86190578 -0.81930065 0.32482629
C -0.87647588 -0.19886227 0.15658410
C -0.47147987 0.58242201 1.24493607
H 0.40022026 1.22054462 1.22713609
C -1.63961843 1.08922242 2.16943398
H -1.36299668 2.01943027 2.64894679
H -2.63334269 1.05138439 1.74112243
C -0.98102386 -0.13144163 2.57938552
C -1.67885142 -1.39979714 2.11106427
H -1.04135529 -2.26444828 2.30070660
H -2.61861530 -1.54343000 2.64815986
Au -0.17420615 -0.02428838 -1.74477083
P 0.62033506 0.18152630 -3.91332577
C 0.02648020 1.67164632 -4.75201933
C 2.42557100 0.26893082 -4.01069374
C 0.15369470 -1.19083804 -4.99687141
H -0.93450890 -1.25848545 -5.04966227
H 0.55814259 -1.03185241 -5.99919528
H 0.54563001 -2.12476716 -4.58971985
H -1.06398194 1.65364837 -4.79684806
H 0.43223012 1.71806233 -5.76528384
H 0.34215589 2.55389731 -4.19215108
H 2.77950911 1.12822457 -3.43812153
H 2.74054155 0.36956501 -5.05190536
H 2.85704294 -0.63949897 -3.58626329
C -0.05256903 -0.17147623 3.74606131
C 1.05015198 -1.02267017 3.72579827
C -0.28798030 0.61743081 4.87059639
C 1.90485258 -1.07229271 4.81904022
H 1.24453169 -1.63540524 2.85150383
C 0.55952902 0.57401953 5.97552052
H -1.15303571 1.27264888 4.89168639
C 1.66097419 -0.28065825 5.93462806
H 2.76770679 -1.72971129 4.80075827
C 0.29479550 1.42487463 7.19090309
H 2.33349010 -0.32585609 6.78610155
H 0.14208529 0.80124423 8.07608352
H 1.14316984 2.08313715 7.39729629
H -0.59320398 2.04424324 7.05202509
*

G11
C -2.11349888 -0.66940585 0.58853902
H -2.24487681 -1.58977510 0.01770760
H -2.96191432 -0.02732391 0.30876242
C -0.88213488 0.05288481 0.16205253
C -0.28806560 0.68076393 1.26423585
H 0.72204180 1.06410784 1.25798173
C -1.28801425 1.47765464 2.18245826
H -0.77813771 2.29783968 2.67121392
H -2.25258564 1.70640880 1.74739504
C -0.98157418 0.12192456 2.58442113
C -1.99074755 -0.90900730 2.10119438
H -1.61228119 -1.91474051 2.28844763
H -2.93956290 -0.79696316 2.62964175
Au -0.15727425 0.07260842 -1.73838045
P 0.66020602 0.10416197 -3.90806995
C -0.34831572 1.09771302 -5.03581000
C 2.33231841 0.78234706 -4.04518103
C 0.74934806 -1.53505712 -4.67005084
H -0.24683314 -1.98086147 -4.69088111
H 1.13413168 -1.45411595 -5.68935102
H 1.40973544 -2.17438584 -4.08127671
H -1.36315199 0.69687205 -5.06526797
H 0.08192050 1.07585011 -6.03978433
H -0.38591593 2.12831226 -4.67810054
H 2.34022203 1.80788954 -3.67137062
H 2.65724501 0.77197349 -5.08813371
H 3.01846652 0.18281512 -3.44408501
C -0.10673622 -0.17618411 3.75584148
C 0.71621159 -1.30392439 3.73497954
C -0.12170606 0.64996401 4.87264800
C 1.51800539 -1.58936938 4.82964129
H 0.73441046 -1.94528166 2.86070260
C 0.68545000 0.35299008 5.96886446
H -0.76279803 1.52339959 4.91177180
C 1.50962816 -0.76692068 5.95123870
H 2.16354534 -2.46096402 4.81493907
O 0.62397651 1.19827012 7.03698700
H 2.13704817 -0.99194306 6.80848986
H 1.22322108 0.88229175 7.72775213
*

G12
C -1.17603194 2.15256262 -0.14490731
H -2.12069868 1.90156994 -0.62876856
H -0.85620974 3.10398759 -0.59537714
C -0.11807364 1.15465993 -0.47361426
C 0.74737858 0.99271626 0.61249007
H 1.44287980 0.17064235 0.70135110
C 1.20819585 2.32390605 1.31158549
H 2.17581571 2.18820760 1.77754852
H 1.08111514 3.22909417 0.73126011
C 0.05674055 1.67084980 1.89134107
C -1.27674769 2.20096988 1.38689859
H -2.08501777 1.55153283 1.72595369
H -1.46123031 3.20575239 1.77264814
Au -0.00506752 0.13472085 -2.23096742
P 0.12748416 -1.02018755 -4.23693126
C 1.12814348 -0.17004131 -5.48308380
C 0.86549908 -2.66512924 -4.07780970
C -1.47784069 -1.29604424 -5.02593567
H -1.95336950 -0.33464983 -5.22882897
H -1.34611399 -1.84276094 -5.96253094
H -2.11838823 -1.87176395 -4.35527959
H 0.69650373 0.81119265 -5.68895536
H 1.15627636 -0.75656900 -6.40435397
H 2.14338942 -0.03659172 -5.10498071

H 1.87761148 -2.57248384 -3.67954010
H 0.90085176 -3.15625527 -5.05302287
H 0.26906588 -3.26607400 -3.38887996
C 0.13414164 0.94480277 3.18914168
C -0.65855339 -0.18366307 3.40797456
C 0.96888703 1.38038606 4.21271704
C -0.61203164 -0.86014581 4.61510952
H -1.31093378 -0.54135367 2.61755282
C 1.01096970 0.70291104 5.42602000
H 1.58472553 2.26178934 4.06920086
C 0.22552805 -0.42664804 5.64853415
H -1.23463118 -1.73805028 4.75105548
H 1.66438865 1.06012276 6.21578032
C 0.30010752 -1.12772130 6.95935510
C -0.92607099 -1.76587340 7.56488229
C 0.17685465 -2.62997922 7.03852864
H 0.99094403 -0.67269358 7.65968066
H -1.84451418 -1.71128296 6.99180931
H -1.04764522 -1.68724567 8.63864223
H 0.81084626 -3.14337536 7.75157127
H 0.00543671 -3.15986464 6.10857176
*

G13
C -2.14922957 -0.73750575 0.29138148
H -2.26163931 -1.64950749 -0.29679097
H -2.99496467 -0.09650129 0.00113121
C -0.91373004 -0.00197632 -0.09711095
C -0.34280548 0.60490538 1.03063477
H 0.66663967 0.98950486 1.05207857
C -1.36473273 1.38903337 1.93421986
H -0.86843191 2.20284734 2.44720258
H -2.32003837 1.62252030 1.48174734
C -1.06450467 0.02673530 2.32274842
C -2.06037893 -0.99931367 1.80279541
H -1.68375648 -2.00709780 1.98390149
H -3.02125074 -0.89729701 2.31094122
Au -0.15294548 0.06397143 -1.98196653
P 0.70999270 0.14884448 -4.13282607
C -0.13330070 1.35009918 -5.19186476
C 2.45948206 0.60684703 -4.19410133
C 0.59989847 -1.42545518 -5.01837885
H -0.44588232 -1.73025023 -5.08879955
H 1.01784331 -1.32084780 -6.02233334
H 1.15423001 -2.19009244 -4.47102342
H -1.19130687 1.09176331 -5.26469822
H 0.31258838 1.34656737 -6.18919988
H -0.04220262 2.34658705 -4.75577107
H 2.59375070 1.59054435 -3.74039527
H 2.80439069 0.63310289 -5.23032349
H 3.04635517 -0.12313441 -3.63350818
C -0.22117358 -0.28308203 3.51297997
C 0.61677060 -1.39809356 3.50001618
C -0.26677355 0.50997912 4.65438673
C 1.40065367 -1.71459673 4.60378506
H 0.66271647 -2.01960960 2.61145346
C 0.51559124 0.19423427 5.76250317
H -0.91882190 1.37716756 4.69279756
C 1.35289261 -0.91927037 5.73886447
H 2.05039296 -2.58201489 4.57296307
C 0.43601267 1.06366502 6.96075786
H 1.95710956 -1.15058697 6.60874962
O 1.07099594 0.88676488 7.97718065
H -0.26267150 1.91682693 6.87275678
*

G14
C -1.90778368 -1.44269174 -0.04250109
H -1.59923357 -2.39687435 -0.47332472
H -2.86967647 -1.20671694 -0.52123948
C -0.96701333 -0.35942340 -0.43623745
C -0.79470061 0.53447179 0.63473710
H 0.00832895 1.25557151 0.68499323
C -2.15323424 1.01635789 1.26802438
H -2.04727186 2.01334633 1.67631688
H -3.04843547 0.83134163 0.68827516
C -1.47033662 -0.08632460 1.91795764
C -1.96542984 -1.46117952 1.49329896
H -1.29885618 -2.23234268 1.88228921
H -2.96897892 -1.64892333 1.87940806
Au -0.05679112 -0.22059771 -2.24816287
P 0.96927729 -0.05093014 -4.32142201
C 0.00287399 0.90903492 -5.51303807
C 2.58881742 0.75445162 -4.26319347
C 1.26128156 -1.64763073 -5.12118262
H 0.30980759 -2.16374425 -5.26196503
H 1.74223531 -1.49822959 -6.09064909
H 1.90439134 -2.25978018 -4.48619291
H -0.96992046 0.43555373 -5.65787683
H 0.53049233 0.95822915 -6.46840907
H -0.14954607 1.91913080 -5.12843708
H 2.47852379 1.76358010 -3.86184041
H 3.01757445 0.80598813 -5.26671302
H 3.25500772 0.18622918 -3.61145574
C -0.77925846 0.08234125 3.22916246
C 0.39615014 -0.62614127 3.48389043
C -1.30472589 0.91912649 4.21029890
C 1.04612974 -0.49924686 4.69894888
H 0.81050407 -1.27476103 2.72016201
C -0.66764082 1.05604918 5.43542903
H -2.22218358 1.46425418 4.02254053
C 0.49819055 0.34359345 5.65457153
H 1.96082389 -1.03827284 4.90434672
H -1.06910490 1.70118860 6.20500859
N 1.18409279 0.48516048 6.95153700
O 0.68627680 1.22044671 7.78358805
O 2.21361317 -0.14074628 7.12330750
*

G15
C -1.68081688 -1.64883047 0.07536147
H -1.27509946 -2.53648851 -0.41254137
H -2.69704942 -1.53284901 -0.33001345
C -0.92872512 -0.42982418 -0.32939691
C -0.83219512 0.45493179 0.75713430
H -0.14119384 1.28495070 0.78856953
C -2.19476079 0.70330008 1.50212988
H -2.21005394 1.69052466 1.94579832
H -3.09191688 0.40311245 0.97609365
C -1.31461511 -0.30487756 2.05909958
C -1.63054737 -1.72361070 1.60934316
H -0.83347974 -2.39952483 1.92246565
H -2.56667099 -2.07066595 2.05069423
Au -0.12046645 -0.13340789 -2.17086721
P 0.80630086 0.19159848 -4.27216438
C -0.42120217 0.21473646 -5.60178052
C 1.71564600 1.74739779 -4.43665402
C 1.98484280 -1.10054151 -4.73812190
H 1.48086591 -2.06880482 -4.73765969
H 2.38651990 -0.89897965 -5.73391063
H 2.80170707 -1.12722111 -4.01450597
H -0.96192250 -0.73337175 -5.61371616
H 0.07390134 0.36428705 -6.56409779
H -1.13238273 1.02443161 -5.42838210
H 1.04270697 2.58439706 -4.24125091
H 2.12578790 1.83714754 -5.44520943
H 2.52921865 1.77180078 -3.70916311
C -0.56191116 -0.07300096 3.32578735
C 0.70013058 -0.64497842 3.48465805
C -1.11716572 0.68708987 4.34991505
C 1.38816981 -0.44419010 4.66130723
H 1.15687222 -1.23588087 2.69941306

C -0.40345910 0.86944700 5.51915331
H -2.10013637 1.13262313 4.25888472
C 0.85088577 0.31236411 5.69229066
F -0.92123450 1.59594295 6.52381345
F 1.53212470 0.49846388 6.82865306
F 2.60891559 -0.97744972 4.83708118
*

G16
C 2.00791322 1.19658359 0.24844552
H 1.86607298 2.12112401 -0.31205689
H 2.98942190 0.80798474 -0.06186388
C 1.00551466 0.16384451 -0.14242261
C 0.68252748 -0.63734235 0.95592787
H -0.17594975 -1.29299441 0.98135086
C 1.89233284 -1.10227270 1.84336856
H 1.65792208 -2.03520310 2.33988992
H 2.86804557 -1.04431002 1.37710222
C 1.22690846 0.10583043 2.27434813
C 1.87581808 1.38491830 1.76698417
H 1.22969065 2.23760749 1.97918307
H 2.83434327 1.55124484 2.26317777
Au 0.18212188 0.00359978 -1.99663389
P -0.74849429 -0.18413106 -4.11181993
C -0.01218485 -1.50904970 -5.10124434
C -2.52318359 -0.53872147 -4.08912835
C -0.57670107 1.31050060 -5.11774487
H 0.48206907 1.54677850 -5.23932239
H -1.03100085 1.15732929 -6.09942356
H -1.06848799 2.14536080 -4.61502371
H 1.05617888 -1.32122368 -5.22292373
H -0.48909166 -1.55200552 -6.08319535
H -0.14667626 -2.46344137 -4.58884156
H -2.69940131 -1.47701821 -3.55991596
H -2.90272683 -0.61907108 -5.11040704
H -3.04777426 0.26411715 -3.56779738
C 0.33753146 0.13314257 3.46844666
C -0.76025759 0.98867293 3.50380050
C 0.59985270 -0.68229080 4.57010480
C -1.58941325 1.02810043 4.61690193
H -0.98057112 1.62157412 2.65105258
C -0.22382335 -0.64148773 5.68238091
H 1.45739090 -1.34788265 4.57105189
C -1.32758666 0.21631494 5.71049628
H -2.45028992 1.68680583 4.64861537
O -0.03330034 -1.40456788 6.80251696
O -2.14603096 0.26570005 6.79632046
H 0.76188450 -1.94509900 6.69964299
H -1.82114089 -0.35871135 7.46113748
*

G17
C -1.61590132 1.86323459 -0.25010669
H -2.46675769 1.39720191 -0.74769327
H -1.54177916 2.87729385 -0.67054162
C -0.33777358 1.17145674 -0.58955456
C 0.54947755 1.23360616 0.48543833
H 1.43465462 0.61872266 0.56229724
C 0.63623802 2.60882216 1.23929793
H 1.60335539 2.71673303 1.71327185
H 0.27682783 3.46871212 0.68776005
C -0.30626208 1.66372478 1.78766373
C -1.72927990 1.84252773 1.28121106
H -2.34465601 0.99822991 1.59460203
H -2.16797720 2.75460537 1.69151720
Au 0.00944296 0.20741042 -2.34879662
P 0.39589103 -0.88204276 -4.35822175
C 0.59437943 0.24791141 -5.75847372
C 1.88935388 -1.90404228 -4.35853050
C -0.94321474 -2.00074642 -4.83916398
H -1.87221025 -1.43549118 -4.93408226
H -0.70828236 -2.47904773 -5.79292380
H -1.07252702 -2.76512901 -4.07063621
H -0.30828958 0.85166188 -5.86876724
H 0.76786031 -0.31964594 -6.67571319
H 1.44180168 0.91085035 -5.57452865
H 2.75845634 -1.27701386 -4.15097139
H 2.01284993 -2.38865596 -5.32980604
H 1.80926264 -2.66456294 -3.57955889
C -0.03883313 0.93306641 3.05548166
C -0.55075455 -0.36379571 3.22166861
C 0.68485533 1.51147579 4.08036459
C -0.33410179 -1.06508202 4.39167112
H -1.10974402 -0.81671667 2.41313823
C 0.90778405 0.81416985 5.27137811
H 1.07511808 2.51717425 3.97546182
C 0.40998288 -0.46631135 5.43871073
O -0.78296903 -2.32232538 4.63462111
H 1.47164412 1.28809277 6.06364853
O 0.57477471 -1.22378981 6.54937551
C -1.54027247 -2.95164584 3.60512963
C 1.31697412 -0.65140473 7.62385985
H -1.80713223 -3.93516415 3.98988726
H -2.45180272 -2.38764093 3.38421505
H -0.94706366 -3.06666812 2.69268808
H 0.83009709 0.25280886 8.00108939
H 1.33606932 -1.40726475 8.40791131
H 2.34101574 -0.41968873 7.31606441
*

G18
C -0.17720268 2.54509629 -0.21871020
H -1.11926360 2.67536267 -0.75055304
H 0.49952121 3.32515151 -0.59706690
C 0.46308835 1.22905768 -0.52943726
C 1.24511864 0.79500793 0.53080324
H 1.61542702 -0.21615873 0.62221092
C 2.02939167 1.86296211 1.36227415
H 2.86627434 1.40387035 1.87255519
H 2.24299043 2.78597031 0.83642679
C 0.69877600 1.62505404 1.85730384
C -0.34687566 2.58063221 1.30603686
H -1.34653346 2.23688032 1.57261235
H -0.20498513 3.57599746 1.73362037
Au 0.12554269 0.16868217 -2.23784930
P -0.26416674 -1.03324094 -4.17988052
C 0.50762491 -0.30328634 -5.64562846
C 0.35313265 -2.73372958 -4.11970145
C -2.01941876 -1.18349842 -4.59665335
H -2.44743010 -0.18817723 -4.72996880
H -2.13875111 -1.75818526 -5.51808020
H -2.54473630 -1.68708311 -3.78301710
H 0.12241500 0.70717012 -5.79444387
H 0.28796953 -0.91069571 -6.52674911
H 1.58814416 -0.25086527 -5.49963067
H 1.43044197 -2.72318762 -3.94404286
H 0.14262777 -3.24402727 -5.06250411
H -0.13196521 -3.26761373 -3.30040115
C 0.43932643 0.83321890 3.08175634
C -0.75412654 0.12277190 3.23045254
C 1.35534154 0.78127683 4.13113112
C -1.02229509 -0.61643499 4.36632663
H -1.48997462 0.13657087 2.43252499
C 1.10153335 0.05204265 5.28100839
H 2.28310822 1.33977324 4.06584638
C -0.09074926 -0.68432741 5.42492942
H -1.96059470 -1.15024913 4.42755391
H 1.83834171 0.06212389 6.07209593
N -0.33474343 -1.44710224 6.54565432
C -1.69128932 -1.91028961 6.79398058
C 0.51199412 -1.26297539 7.71366162
H -1.69672568 -2.50120084 7.70868602

H -2.40082959 -1.08008118 6.91013676
H -2.03950902 -2.55336775 5.98334650
H 0.19383792 -1.95881918 8.48865287
H 1.55495835 -1.48487956 7.47817174
H 0.45439429 -0.24234835 8.11548405
*

G19
C -2.30635955 -0.82537035 -0.67232291
H -2.26860679 -1.82642772 -1.10598178
H -3.14662184 -0.32566051 -1.17683884
C -1.08696822 -0.05499370 -1.03035702
C -0.68712080 0.73832343 0.06449114
H 0.29208979 1.18757683 0.14589975
C -1.85804912 1.59816028 0.66507045
H -1.47690553 2.51656233 1.09321839
H -2.75549597 1.68789171 0.06690783
C -1.54754893 0.33424732 1.31206377
C -2.41413647 -0.83280196 0.86121644
H -2.01630863 -1.76795218 1.25860412
H -3.43930204 -0.71765746 1.21692240
Au -0.12584837 -0.17406299 -2.81558081
P 0.97789904 -0.32594262 -4.85076728
C 0.15235664 0.58223848 -6.18045303
C 2.66719490 0.32060722 -4.80708406
C 1.13217261 -2.02377790 -5.45738411
H 0.13799564 -2.45669423 -5.58268129
H 1.65720408 -2.03238300 -6.41537858
H 1.68842974 -2.62055059 -4.73218178
H -0.85744105 0.19065500 -6.31617786
H 0.71401945 0.47291351 -7.11107867
H 0.08908693 1.63909665 -5.91511192
H 2.64632925 1.37341316 -4.51964300
H 3.13223957 0.22022277 -5.79053502
H 3.25009531 -0.23509343 -4.07024265
C -0.87432547 0.28290424 2.64124895
C -1.15951831 1.21688055 3.62668306
C 0.04079824 -0.73035548 2.90730427
C -0.52680494 1.15545392 4.86840058
H -1.88253553 2.00260268 3.44689152
C 0.66558100 -0.81694365 4.14670463
H 0.28487034 -1.46072736 2.14528296
C 0.38079995 0.13537082 5.12086616
N 1.04665435 0.05853224 6.43123277
O 1.42895889 1.10066456 6.91973834
O 1.16550070 -1.04214259 6.92665318
C -0.88239640 2.13810837 5.85999607
N -1.22418245 2.93930768 6.60783208
C 1.62669705 -1.87081803 4.34947373
N 2.39293855 -2.72127369 4.43666768
*

G2
C -2.05892685 -1.13433410 0.15317777
H -1.87384561 -2.08462168 -0.34984647
H -3.01627849 -0.77154626 -0.24962140
C -1.03364743 -0.12214355 -0.22543313
C -0.74640789 0.70354396 0.86995469
H 0.11920614 1.34841398 0.91773263
C -2.01188645 1.24397130 1.63226082
H -1.79411282 2.19775957 2.09569646
H -2.95282385 1.17617363 1.10113121
C -1.39595113 0.04779570 2.16647142
C -2.03427807 -1.24679809 1.68530003
H -1.42024209 -2.09709111 1.98545194
H -3.02774532 -1.37252195 2.12031588
Au -0.14930843 -0.00851896 -2.05334135
P 0.85324862 0.11699970 -4.14057912
C 0.09666039 1.35255698 -5.22529288
C 2.60824996 0.55336492 -4.07424923
C 0.78504112 -1.43480889 -5.06903051
H -0.25705216 -1.72528210 -5.21490080
H 1.26917026 -1.31297713 -6.04079095
H 1.29373841 -2.21839969 -4.50439833
H -0.95660484 1.10848855 -5.37553437
H 0.60907826 1.36646732 -6.19004860
H 0.16809666 2.33759724 -4.76048035
H 2.72343349 1.52444425 -3.58925816
H 3.02115763 0.59897256 -5.08468037
H 3.14797650 -0.19709053 -3.49369901
C -0.60334321 0.06748650 3.42895220
C 0.49508469 -0.78102059 3.56962374
C -0.95298106 0.90357929 4.48518408
C 1.23733092 -0.79367520 4.73982921
H 0.78103232 -1.43333994 2.75132068
C -0.22035774 0.90072843 5.66635271
H -1.81062665 1.56063572 4.39503659
C 0.86735900 0.05131045 5.77700470
H 2.09407764 -1.44776009 4.84652310
H -0.49517964 1.54933105 6.48924421
Cl 1.79648679 0.04099880 7.25442863
*

G20
C -2.39558505 -0.72995786 -1.43689296
H -2.39938180 -1.72873100 -1.87683948
H -3.20305650 -0.18590529 -1.94925852
C -1.13573588 -0.01332225 -1.76885489
C -0.72758774 0.76611976 -0.66922333
H 0.26662357 1.17702006 -0.56886401
C -1.87435717 1.66601927 -0.07939749
H -1.46431203 2.56683953 0.35926980
H -2.75671677 1.79369333 -0.69293012
C -1.62387269 0.38976909 0.56650775
C -2.52695121 -0.74059213 0.09456636
H -2.17094879 -1.69219457 0.49264316
H -3.55231983 -0.58844715 0.43587703
Au -0.13835161 -0.18469425 -3.53024835
P 1.00290811 -0.39353313 -5.53927503
C 0.22186938 0.50720980 -6.90057246
C 2.70360079 0.22038302 -5.47401427
C 1.13543354 -2.10416551 -6.11436852
H 0.13548752 -2.51967626 -6.25223665
H 1.67890727 -2.13929345 -7.06141347
H 1.66526656 -2.69928589 -5.36830767
H -0.79319704 0.13419315 -7.04875189
H 0.79860220 0.36963781 -7.81816467
H 0.17571524 1.56976047 -6.65512959
H 2.69836006 1.27781932 -5.20336568
H 3.18465254 0.09531650 -6.44689943
H 3.26166857 -0.33479923 -4.71778091
C -0.97212210 0.30755755 1.90489372
C -1.23992699 1.24535568 2.89118387
C -0.09536904 -0.73651491 2.17860773
C -0.63833914 1.15501890 4.14040640
H -1.93173946 2.05363483 2.69268336
C 0.51472132 -0.84690048 3.41747559
H 0.12524460 -1.46626060 1.40960637
C 0.23527074 0.10682792 4.39023999
C -0.96114486 2.21038489 5.18000294
C 1.46506279 -2.00291778 3.66025530
N 0.88327020 0.00049068 5.71750640
F -2.13028696 2.81414184 4.91382426
F -0.02284556 3.17247393 5.22446113
F -1.06114647 1.68884236 6.41712263
F 1.94487196 -2.49005579 2.50462058
F 0.86005988 -3.02225567 4.29509893
F 2.52664783 -1.63908079 4.40365037
O 1.68648506 0.85977006 6.00753732
O 0.56134430 -0.93838408 6.41223118
*

G21

C	-1.86225898	-1.45769686	-0.48756268
H	-1.51899544	-2.37315894	-0.97299803
H	-2.83452216	-1.23381045	-0.95090402
C	-0.96699186	-0.31922348	-0.82410384
C	-0.81468788	0.51365589	0.30117652
H	-0.03584579	1.25713860	0.39042108
C	-2.18848047	0.91578141	0.95604304
H	-2.11721163	1.89055587	1.42153544
H	-3.07931484	0.73062082	0.36989811
C	-1.46364685	-0.19852724	1.54083300
C	-1.91235076	-1.56540964	1.04494663
H	-1.21630044	-2.33222185	1.38839348
H	-2.90661856	-1.80983052	1.42267711
Au	-0.08370629	-0.04192346	-2.63237800
P	0.92032110	0.26857337	-4.70065620
C	-0.18624027	1.01334882	-5.92367203
C	2.36969661	1.35027165	-4.64674475
C	1.50105041	-1.26902227	-5.45752135
H	0.65743403	-1.94843915	-5.59252162
H	1.95944665	-1.05937987	-6.42676272
H	2.23456333	-1.74289706	-4.80258006
H	-1.05784574	0.37161841	-6.06444642
H	0.33636453	1.13270897	-6.87560547
H	-0.51990484	1.98919534	-5.56613741
H	2.07767853	2.33064749	-4.26584528
H	2.79390816	1.45934823	-5.64753769
H	3.11871927	0.92097100	-3.97867131
C	-0.78141691	-0.08063936	2.86259470
C	0.43666154	-0.72962933	3.05840797
C	-1.36803862	0.64402023	3.89405122
C	1.05964285	-0.63297423	4.28206610
H	0.90674687	-1.30215657	2.26792115
C	-0.73176044	0.70679199	5.11776886
H	-2.31340062	1.15592757	3.76454178
C	0.48668911	0.07941605	5.32959085
N	1.15675215	0.16680656	6.62834126
O	0.50758279	-0.12888481	7.61059399
O	2.31543989	0.52939558	6.63304383
F	-1.28493599	1.41171995	6.11032517
F	2.22499315	-1.25890383	4.48163493

*

G22

C	-2.46968395	-1.20405635	-0.92278515
H	-2.16480027	-2.09621823	-1.46818388
H	-3.43578462	-0.89351594	-1.34518365
C	-1.51177667	-0.06525824	-1.11441351
C	-1.56938732	0.82677457	-0.06436560
H	-0.82219922	1.58747259	0.11349823
C	-2.90163738	1.01201848	0.72321483
H	-2.88743008	1.94082438	1.27879698
H	-3.79723490	0.82538941	0.14148446
C	-2.16194115	-0.11029895	1.23277202
C	-2.52886627	-1.43678264	0.59198810
H	-1.80950762	-2.20480508	0.87485805
H	-3.51447043	-1.75117047	0.94515434
Au	-0.19789983	0.06103540	-2.67234543
P	1.28730864	0.19347424	-4.44376778
C	0.77452995	1.35854037	-5.73133493
C	2.94630949	0.73146165	-3.95818147
C	1.53744586	-1.37902163	-5.30527474
H	0.58296950	-1.73317862	-5.69926464
H	2.24556506	-1.24994710	-6.12714255
H	1.92540554	-2.12001353	-4.60381643
H	-0.19373461	1.05411104	-6.13297201
H	1.51322624	1.37767785	-6.53614793
H	0.67916135	2.35703255	-5.30062705
H	2.88703248	1.71894325	-3.49666426
H	3.59837703	0.77671491	-4.83357250
H	3.35908936	0.02794456	-3.23270869
C	-1.39218393	-0.05204480	2.48839710
C	-0.27699244	-0.87572613	2.68154807
C	-1.74557152	0.81264649	3.52789881
C	0.45731503	-0.84156210	3.84859093
H	0.04019199	-1.54367267	1.88714955
C	-1.03172709	0.85579566	4.71079306
H	-2.61545278	1.45237199	3.42438435
C	0.09588243	0.02805583	4.90398503
H	1.32504209	-1.48095780	3.94740306
H	-1.35547998	1.52328820	5.49892638
N	0.80713256	0.06421370	6.05771812
C	0.53511208	0.99703132	7.14900869
C	1.91126083	-0.84458893	6.35972230
H	0.43262440	2.01795521	6.77242567
H	-0.39523015	0.73017041	7.66614546
C	1.74897863	0.83420726	8.06243870
H	1.63949269	-1.87786273	6.12939979
H	2.79980768	-0.58672736	5.76996796
C	2.14890796	-0.62749872	7.85351835
H	2.55406009	1.49394756	7.72684832
H	1.51786459	1.07357934	9.10115481
H	1.48887743	-1.28140579	8.43034186
H	3.17966818	-0.83780623	8.14158302

*

G23

C	-1.21595730	2.17927043	-0.42957161
H	-2.14142329	1.91863950	-0.94452933
H	-0.88424431	3.12703032	-0.87922532
C	-0.14286258	1.18299383	-0.70961877
C	0.68243240	1.03447569	0.40969327
H	1.37597230	0.21510317	0.53258541
C	1.11331694	2.37265159	1.11259414
H	2.06343030	2.24500922	1.61524155
H	1.00382832	3.27304070	0.52136646
C	-0.05756193	1.72153691	1.65508128
C	-1.37272175	2.24394684	1.09699590
H	-2.19303288	1.59794773	1.41294065
H	-1.57162369	3.25260193	1.46488708
Au	0.03648893	0.14802574	-2.45232842
P	0.23784096	-1.02254489	-4.44350677
C	1.24296283	-0.16134301	-5.67825379
C	1.00926379	-2.64864607	-4.25408012
C	-1.34541867	-1.34166591	-5.26005699
H	-1.83875050	-0.39309256	-5.48002804
H	-1.18370720	-1.89297102	-6.18922832
H	-1.98517565	-1.92634232	-4.59641311
H	0.79350207	0.80802849	-5.90147554
H	1.30158300	-0.75546455	-6.59319420
H	2.24760936	-0.00182113	-5.28233864
H	2.01029626	-2.52925102	-3.83539562
H	1.07672026	-3.14742100	-5.22368719
H	0.41217599	-3.25703391	-3.57226795
C	-0.02388418	1.00823224	2.96073299
C	-0.84528833	-0.10337172	3.16505641
C	0.78125810	1.43600244	4.00698998
C	-0.83073376	-0.74449740	4.37922508
H	-1.50621031	-0.46883327	2.38674968
C	0.79047500	0.76392525	5.22603590
H	1.41388697	2.30737745	3.88094677
C	-0.01089225	-0.35723314	5.45094494
F	-1.68100122	-1.78875370	4.55170350
H	1.43637967	1.12430128	6.01567742
N	-0.06636235	-1.04785764	6.67255754
C	0.31415284	-2.46101234	6.60269942
C	0.57484295	-0.39017004	7.80193163
H	1.39901454	-2.57584431	6.46420178
H	0.03100849	-2.94879248	7.53754256
H	-0.19739615	-2.96335856	5.78505200
H	0.31963073	-0.94368415	8.70744890
H	1.67098429	-0.36285623	7.71546221

H 0.20314713 0.63049115 7.90635149
*

G24
C -2.40273577 -0.23747466 -0.88009418
H -2.62589845 -1.20204126 -1.33967251
H -3.09724465 0.47403235 -1.35061414
C -1.03158658 0.21130382 -1.24381637
C -0.43329370 0.85279749 -0.14469109
H 0.62704645 1.04792957 -0.07449577
C -1.35005365 1.95650070 0.50342178
H -0.74612291 2.73988142 0.94370266
H -2.20212581 2.28218622 -0.07937738
C -1.35108262 0.64149046 1.11764375
C -2.48300442 -0.26348645 0.65444151
H -2.31778745 -1.27843487 1.01987012
H -3.44334658 0.08665001 1.03723129
Au -0.16372285 -0.09288893 -3.05588295
P 0.81412528 -0.42959428 -5.13223063
C 0.48121067 0.91559936 -6.29635805
C 2.61699518 -0.57117904 -5.07112723
C 0.24575122 -1.93242713 -5.96470267
H -0.83550659 -1.88273954 -6.10575859
H 0.73612684 -2.03146771 -6.93587824
H 0.48155287 -2.80128845 -5.34743379
H -0.59621945 1.01176851 -6.44251919
H 0.96218868 0.70777851 -7.25502272
H 0.86766731 1.85205619 -5.88996542
H 3.03887887 0.34181261 -4.64688668
H 3.01509537 -0.72390436 -6.07696466
H 2.89456041 -1.41563187 -4.43753100
C -0.69907470 0.39547371 2.43626247
C -0.02798033 -0.80172030 2.66388387
C -0.77945873 1.33320128 3.45914599
C 0.55982290 -1.04760212 3.89640776
H 0.04015351 -1.54045323 1.87178120
C -0.19356987 1.07136407 4.69122543
H -1.30826407 2.26522619 3.29642824
C 0.48416141 -0.11618842 4.92232468
H 0.94172194 -0.31314675 5.88262098
C -0.27366509 2.11870014 5.77086513
F 0.62865849 3.10748747 5.57619700
F -0.03913820 1.61527208 6.99584147
F -1.48523449 2.70984662 5.81101162
C 1.27810939 -2.35625331 4.09677874
F 1.84770128 -2.45678683 5.30942433
F 2.25811296 -2.53314608 3.18351527
F 0.44170267 -3.40978410 3.96550754
*

G3
C -2.14224734 0.93684242 -0.33214281
H -2.83997162 0.40002318 -0.97676475
H -2.25196382 1.99741170 -0.60329860
C -0.73489740 0.56853092 -0.64878136
C 0.02804951 0.54036156 0.52865616
H 1.00256672 0.07865320 0.59538116
C -0.16518700 1.80502079 1.44553781
H 0.73444735 1.99927258 2.01552796
H -0.63198501 2.66509844 0.98275552
C -0.95986050 0.63582342 1.76375561
C -2.35749394 0.65424495 1.16289483
H -2.83053317 -0.32038192 1.29259309
H -2.98026705 1.40472602 1.65349079
Au -0.04703778 0.13424257 -2.51225760
P 0.74096429 -0.34954577 -4.63908830
C 0.86495863 1.10982642 -5.70239021
C 2.39444435 -1.08496696 -4.64973675
C -0.30228718 -1.51407860 -5.55052382
H -1.30661158 -1.09897664 -5.65317374
H 0.11937806 -1.69806940 -6.54143093
H -0.36375986 -2.45498398 -5.00058261
H -0.12000943 1.56807585 -5.80872679
H 1.24222805 0.82337171 -6.68684864
H 1.54421157 1.83382753 -5.24856075
H 3.10020888 -0.39831663 -4.17861608
H 2.70969683 -1.28362384 -5.67672133
H 2.38147249 -2.01939125 -4.08555775
C -0.67290910 -0.20748460 2.95996938
C -0.87914463 -1.58675263 2.90432462
C -0.22809553 0.36510730 4.14469165
C -0.63842868 -2.38179940 4.01386830
H -1.22388670 -2.03818030 1.97982880
C 0.00571844 -0.43730124 5.25696421
H -0.07500634 1.43614909 4.20638143
C -0.19223310 -1.80924121 5.20009948
H -0.79651202 -3.45275208 3.95915631
C 0.52033736 0.20026337 6.51873480
H -0.00509619 -2.42779368 6.06941634
F 1.86357718 0.37276194 6.48562379
F 0.25552221 -0.53513849 7.61535827
F -0.01285311 1.42257070 6.72726847
*

G4
C -2.02770427 -1.39889371 -0.42202064
H -1.73051522 -2.35737246 -0.85086327
H -2.97152433 -1.13685832 -0.92280383
C -1.05260979 -0.33351614 -0.78204748
C -0.89998494 0.55704194 0.29256464
H -0.08670533 1.26424304 0.36875268
C -2.26455207 1.05528589 0.89960026
H -2.15150451 2.04750268 1.31747842
H -3.14758969 0.89011516 0.29563220
C -1.61817662 -0.06202170 1.55915505
C -2.12522101 -1.42493220 1.11142441
H -1.48220852 -2.20984869 1.51239187
H -3.14135947 -1.59781048 1.47092824
Au -0.06937152 -0.21899912 -2.55776241
P 1.04524545 -0.08036381 -4.58683075
C 0.16901381 0.92284241 -5.81226262
C 2.69181318 0.65909382 -4.45604205
C 1.30672690 -1.68358199 -5.38428696
H 0.34189928 -2.15946319 -5.56926065
H 1.83468348 -1.54916313 -6.33120679
H 1.89619989 -2.32493678 -4.72648527
H -0.81544104 0.49033680 -6.00011854
H 0.73744663 0.95431601 -6.74469527
H 0.04189473 1.93678121 -5.42853399
H 2.60544314 1.66985668 -4.05297269
H 3.16440283 0.69802128 -5.44024177
H 3.30617731 0.06107458 -3.78045911
C -0.95473297 0.08730381 2.88680929
C 0.19696567 -0.65009584 3.17087698
C -1.47993418 0.93263944 3.85709055
C 0.81466426 -0.53799443 4.40316369
H 0.61476402 -1.30794698 2.41647855
C -0.86619513 1.04917826 5.09960360
H -2.37922844 1.50117928 3.64996090
C 0.27768679 0.31398214 5.36669464
H 1.71256428 -1.10715957 4.61773428
H -1.28478390 1.70794481 5.85022864
C 0.97903765 0.42817247 6.69238089
F 2.20213023 0.99420285 6.56655843
F 1.18038702 -0.78081878 7.26269407
F 0.29795023 1.17120562 7.58348875
*

G5
C -1.10485827 2.03404891 0.53452922
H -2.04474591 1.89234802 0.00034120
H -0.72486777 3.01604723 0.21545099

C	-0.08238243	1.03107101	0.11640009
C	0.75682645	0.71769430	1.18697921
H	1.41611512	-0.13837171	1.19439929
C	1.23644878	1.92554323	2.06875765
H	2.18240988	1.69391227	2.54131597
H	1.16241365	2.90210689	1.60638355
C	0.04318406	1.25638157	2.53064917
C	-1.25121958	1.90035576	2.05731191
H	-2.09604490	1.25147403	2.29178722
H	-1.40731991	2.85775006	2.55908588
Au	0.00937784	0.18953778	-1.73492983
P	0.11272465	-0.76256983	-3.84606957
C	1.37306426	-0.01614873	-4.90942506
C	0.49986452	-2.53044350	-3.82127686
C	-1.43304071	-0.62942099	-4.77788504
H	-1.69364370	0.42354092	-4.90026188
H	-1.32150789	-1.09397784	-5.76036725
H	-2.23273953	-1.12866795	-4.22759190
H	1.16031710	1.04714386	-5.03449651
H	1.37552816	-0.50497581	-5.88644684
H	2.35410506	-0.12746883	-4.44394533
H	1.46590908	-2.68411513	-3.33684777
H	0.53703198	-2.92059077	-4.84103679
H	-0.26696235	-3.06272164	-3.25541991
C	0.05207921	0.36335344	3.72058607
C	-0.80618819	-0.73779637	3.77732001
C	0.89074662	0.60910708	4.80400803
C	-0.82428459	-1.57420687	4.87892682
H	-1.46213299	-0.95057711	2.93924401
C	0.88081045	-0.21901415	5.91921421
H	1.55707159	1.46467146	4.78828970
C	0.02263190	-1.31341078	5.95613310
H	-1.48546422	-2.43264183	4.91815435
H	1.53494507	-0.01440442	6.76140170
O	-0.03175732	-2.16200211	7.01921988
H	0.60003588	-1.87149900	7.69217677

*

G6

C	-2.20445978	-0.95606251	0.10989702
H	-2.11035713	-1.89349967	-0.43873082
H	-3.14258461	-0.49829418	-0.23790216
C	-1.11369384	-0.00145251	-0.24525110
C	-0.78981452	0.79670152	0.85297245
H	0.10886505	1.39402145	0.90892113
C	-1.99767386	1.34291859	1.69541756
H	-1.72123049	2.26098442	2.19805838
H	-2.95643909	1.34566480	1.19184147
C	-1.43309858	0.09786954	2.15924447
C	-2.14336105	-1.14007476	1.63306746
H	-1.56308621	-2.03160671	1.87470676
H	-3.12859050	-1.23967376	2.09367695
Au	-0.18339976	0.04774825	-2.05531899
P	0.86936771	0.09030604	-4.12020708
C	0.21007025	1.35268614	-5.23731809
C	2.64506165	0.42592580	-4.02095765
C	0.73218442	-1.46671967	-5.03271110
H	-0.32155355	-1.69815391	-5.19914643
H	1.24399147	-1.38555723	-5.99447597
H	1.18155889	-2.27145609	-4.44786307
H	-0.85231689	1.16868225	-5.40751861
H	0.74258717	1.32343920	-6.19080355
H	0.32925750	2.33832783	-4.78365965
H	2.80564600	1.39604860	-3.54703160
H	3.08203401	0.43222235	-5.02223419
H	3.12772370	-0.34536814	-3.41787695
C	-0.59441167	0.02250553	3.38519390
C	0.44964401	-0.90614147	3.46755245
C	-0.83609314	0.84307591	4.47898766
C	1.22496984	-1.00303337	4.60451236
H	0.66167706	-1.55276753	2.62199147
C	-0.06701276	0.75538771	5.63714019
H	-1.64693042	1.56273348	4.44384861
C	0.97022309	-0.17090076	5.70167593
H	2.03859580	-1.71722433	4.66654885
H	-0.28928082	1.40661840	6.47184859
O	1.78188592	-0.34103335	6.77444620
C	1.54743364	0.48182707	7.91621808
H	2.29201475	0.18687912	8.65441759
H	1.67644775	1.54027959	7.67185541
H	0.54567108	0.31436237	8.32251267

*

G7

C	-0.95710737	2.14415671	0.19464200
H	-1.90651586	2.05716038	-0.33521497
H	-0.49442283	3.06977670	-0.17912968
C	-0.03121544	1.03532150	-0.17261195
C	0.75061869	0.67524532	0.93123057
H	1.32061466	-0.24120648	0.98226059
C	1.36847894	1.88059888	1.73098483
H	2.28838229	1.57909055	2.21545940
H	1.39226254	2.83063896	1.21218909
C	0.11386603	1.35953925	2.22733972
C	-1.11156066	2.10670648	1.72247667
H	-2.01614498	1.55902808	1.99057209
H	-1.16829163	3.10047176	2.17124086
Au	0.02503258	0.14488225	-2.00093698
P	0.08426268	-0.86264786	-4.08844051
C	1.32194015	-0.14352209	-5.19616112
C	0.46996949	-2.62979093	-4.02874122
C	-1.48097980	-0.74892640	-4.98981163
H	-1.74242108	0.30132861	-5.13197416
H	-1.39065735	-1.23722328	-5.96291357
H	-2.26966239	-1.23333383	-4.41111282
H	1.10696720	0.91661853	-5.34234582
H	1.30277011	-0.65598223	-6.16081166
H	2.31284396	-0.24459726	-4.74968541
H	1.44596390	-2.77321514	-3.56143671
H	0.48491483	-3.04449230	-5.03933806
H	-0.28521377	-3.14697065	-3.43396811
C	0.03745418	0.54919550	3.47552102
C	-0.90051022	-0.47534278	3.58609932
C	0.87792484	0.80968114	4.55707128
C	-0.99048343	-1.22224830	4.75038705
H	-1.55727128	-0.69680717	2.75092247
C	0.78420961	0.06490642	5.72298654
H	1.60800733	1.60899694	4.49030812
C	-0.15315264	-0.96410716	5.83943862
H	-1.72062089	-2.02229090	4.82153331
H	1.45078470	0.29139678	6.54724003
C	-0.29760996	-1.78774008	7.05853007
C	0.36342363	-1.62934710	8.20210751
H	-1.02832521	-2.58977942	6.98097139
H	0.17791584	-2.29309925	9.03997495
H	1.10026726	-0.84619847	8.35001760

*

G8

C	-2.02336469	-1.14998505	0.27818362
H	-1.83630578	-2.10063805	-0.22362008
H	-2.98153998	-0.79036560	-0.12540130
C	-1.00020390	-0.13717425	-0.10354886
C	-0.70716450	0.68567351	0.99314153
H	0.15889267	1.33006912	1.03887408
C	-1.97346833	1.23093554	1.75171633
H	-1.75446185	2.18545298	2.21304351
H	-2.91402221	1.16338780	1.21994543
C	-1.35843205	0.03502992	2.28869931
C	-1.99790664	-1.26017726	1.81055681
H	-1.38412519	-2.11017075	2.11202498
H	-2.99131406	-1.38445219	2.24602602

Au -0.13109761 -0.01557596 -1.93803307
P 0.85145227 0.12134425 -4.03409974
C 0.10019197 1.38045496 -5.09514263
C 2.61269365 0.53369725 -3.98061990
C 0.75285802 -1.41792535 -4.98038794
H -0.29452799 -1.69244311 -5.11906493
H 1.22880835 -1.29100169 -5.95552491
H 1.25640498 -2.21504332 -4.43026956
H -0.95783538 1.15250254 -5.23695029
H 0.60238663 1.39974745 -6.06516200
H 0.19000505 2.35845012 -4.61876185
H 2.74552953 1.49726294 -3.48525936
H 3.01597869 0.58609052 -4.99459445
H 3.14821830 -0.23074685 -3.41462206
C -0.56886786 0.05638088 3.55325176
C 0.53123471 -0.79011515 3.69677878
C -0.92467131 0.89196514 4.60833001
C 1.26618600 -0.79871786 4.86941876
H 0.81948317 -1.44081240 2.87792090
C -0.19513065 0.88737943 5.78890923
H -1.78386151 1.54631966 4.51300106
C 0.90643537 0.04251860 5.92725250
H 2.12364796 -1.45344434 4.97327995
H -0.47949739 1.53729432 6.60832997
C 1.66701076 0.03576347 7.15021033
C 2.30007693 0.03022122 8.17056746
H 2.86410366 0.02537696 9.07910948
*

G9
C -1.68248567 -1.64834589 0.40246233
H -1.27447435 -2.57090500 -0.01357568
H -2.69018669 -1.55541748 -0.02933325
C -0.91330650 -0.46628386 -0.07334247
C -0.82652440 0.49086946 0.95057145
H -0.12942350 1.31623064 0.93530741
C -2.20027199 0.80290497 1.65189397
H -2.21256199 1.81962084 2.02320250
H -3.09093511 0.47463731 1.13159104
C -1.33884065 -0.17016453 2.29390573
C -1.66004855 -1.61478315 1.93892770
H -0.87502557 -2.27304086 2.31396101
H -2.60715006 -1.92232059 2.38611697
Au -0.07541613 -0.29270662 -1.91734381
P 0.88478820 -0.08326705 -4.01825515
C 0.35447213 1.40260534 -4.90517682
C 2.69137198 0.01309500 -3.96690168
C 0.51360447 -1.46006249 -5.13219237
H -0.56652096 -1.53350904 -5.27187370
H 0.99629548 -1.30086322 -6.09921229
H 0.87659439 -2.39095845 -4.69255795
H -0.72841589 1.37889529 -5.04033125
H 0.84258138 1.44955689 -5.88138671
H 0.61844069 2.28718286 -4.32267864
H 2.99293659 0.87474569 -3.36845766
H 3.08928876 0.11487233 -4.97923500
H 3.09123861 -0.89288149 -3.50776421
C -0.60043496 0.14303449 3.55175277
C 0.66377941 -0.40975853 3.76279888
C -1.16383459 0.96256396 4.52627262
C 1.36156359 -0.14493748 4.92722659
H 1.10746170 -1.04406834 3.00331270
C -0.47637846 1.23503538 5.69965863
H -2.14953195 1.38626343 4.37339488
C 0.78636622 0.68071587 5.89540721
H 2.34591424 -0.56708716 5.09087357
H -0.91350841 1.87054105 6.46047454
C 1.50785041 0.96350105 7.10947947
N 2.08522115 1.18936268 8.07820481
*

TSAB0
C -0.24505336 -4.67608220 0.08929035
C 1.01572806 -4.19614479 -0.24507971
C 1.23785186 -2.83042447 -0.29604568
C 0.19406801 -1.94057759 -0.02122453
C -1.06985154 -2.43099241 0.32789784
C -1.28469634 -3.79569827 0.38041110
H -0.41892115 -5.74503025 0.13566099
H 1.82299927 -4.88538236 -0.46064252
H 2.22076257 -2.44885870 -0.55299210
H -1.87716720 -1.75545289 0.58698708
H -2.25851673 -4.17909783 0.65988809
C 0.48122286 -0.51131230 -0.09336455
H 1.52765393 -0.21650503 -0.05341212
H 0.08675746 0.00976251 -1.23621370
H -1.50449403 0.23115643 -0.26397722
C -0.18864397 1.95468424 -0.03933923
C -1.18353141 2.84628733 -0.45420720
C 1.01394306 2.44284966 0.48535948
C -0.97048288 4.21163806 -0.36742175
H -2.12078150 2.46636466 -0.84782626
C 1.21951790 3.80720251 0.57275677
H 1.77680596 1.76581016 0.85259464
C 0.23112220 4.68941359 0.14211066
H -1.73945310 4.90227976 -0.69132200
H 2.14457575 4.18883519 0.98755586
H 0.39697472 5.75805719 0.21647273
C -0.46277511 0.52587566 -0.15689670
*

TSAB1
C -0.11271710 -4.22336116 0.05107791
C 1.11871779 -3.69267271 -0.33081027
C 1.31153134 -2.32376847 -0.38926835
C 0.26697886 -1.45121135 -0.07356406
C -0.96986098 -1.97600670 0.32157248
C -1.15050849 -3.34217491 0.37906984
H 1.93491616 -4.36036088 -0.58190354
H 2.27720434 -1.92717657 -0.68657419
H -1.78572457 -1.32304576 0.61121870
H -2.10782933 -3.74253995 0.69435536
C 0.52111900 -0.01555859 -0.16013437
H 1.56194959 0.30039154 -0.15009327
H 0.09389873 0.47338444 -1.27259429
H -1.48504723 0.68645398 -0.22931953
C -0.20172580 2.43268264 -0.04260991
C -1.24376117 3.30247731 -0.38508805
C 1.01956261 2.94875636 0.41020087
C -1.06085998 4.67185405 -0.29852948
H -2.19378290 2.90089324 -0.72249655
C 1.19510318 4.31675912 0.49735115
H 1.82113365 2.28852789 0.72081967
C 0.15840891 5.17609174 0.13874655
H -1.86592376 5.34515688 -0.56629425
H 2.13449413 4.71972112 0.85590370
H 0.30138289 6.24810285 0.21284803
C -0.44695126 1.00303271 -0.15366746
C -0.32474104 -5.70783921 0.14236478
H -0.37234986 -6.01532220 1.19068860
H -1.26759049 -5.99479099 -0.32739883
H 0.48610877 -6.25691042 -0.33609824
*

TSAB10
C -0.72160334 -4.21653759 0.25088665
C 0.57624647 -3.86004311 -0.12262380
C 0.87648503 -2.50821680 -0.20929408
C -0.09260984 -1.53448527 0.05997205
C -1.38091503 -1.91292177 0.44455165
C -1.68768590 -3.25951480 0.53652727
H 1.87857340 -2.20134621 -0.49430991

H -2.13102210 -1.17379774 0.70100220
H -2.67974769 -3.56814961 0.84362861
C 0.30193699 -0.13314788 -0.05880785
H 1.36757019 0.08379344 -0.03244754
H -0.05811791 0.37298217 -1.20955055
H -1.62507308 0.74759920 -0.23278636
C -0.18610899 2.37574286 -0.06668386
C -1.12193598 3.32585937 -0.48963133
C 1.05792643 2.78994390 0.42467798
C -0.81021131 4.67409480 -0.44376624
H -2.09105727 3.00423010 -0.85710567
C 1.36233134 4.13758584 0.47089555
H 1.77690305 2.06927630 0.79724914
C 0.43172809 5.07713960 0.03256237
H -1.53356205 5.40968192 -0.77374065
H 2.31991056 4.46241789 0.85942616
H 0.67501080 6.13264569 0.07473360
C -0.56408877 0.96900119 -0.13908782
H -0.97669392 -5.26835707 0.33144009
C 1.60856006 -4.91664274 -0.41241557
H 2.56541738 -4.47360108 -0.69000872
H 1.76592909 -5.54935441 0.46391910
H 1.27963730 -5.56103818 -1.23072044
*

TSAB11
C -0.70913852 -4.24300776 0.16390412
C 0.58260219 -3.85660754 -0.18969380
C 0.89700590 -2.50370352 -0.25592488
C -0.08286488 -1.55002790 0.02150631
C -1.37640631 -1.93746759 0.38705115
C -1.67494473 -3.28689658 0.45399814
H 1.90585311 -2.21423437 -0.52870710
H -2.13119625 -1.20657852 0.64933140
H -2.66793815 -3.60664106 0.74603983
C 0.30792884 -0.14668444 -0.06834933
H 1.37328194 0.07213535 -0.04573667
H -0.07027436 0.39967011 -1.22389841
H -1.62132262 0.73004373 -0.24110625
C -0.18263140 2.36192341 -0.05641515
C -1.11013107 3.31671130 -0.48454006
C 1.05264430 2.76883590 0.45995261
C -0.79806117 4.66429781 -0.41838902
H -2.07323316 3.00043806 -0.87201382
C 1.35742460 4.11602979 0.52663419
H 1.76502363 2.04374643 0.83688105
C 0.43568415 5.06140818 0.08328182
H -1.51540564 5.40389211 -0.75257702
H 2.30867112 4.43500368 0.93510692
H 0.67895284 6.11612655 0.14126216
C -0.56014645 0.95236870 -0.15308662
H -0.95882397 -5.29773549 0.22491379
O 1.57125386 -4.73190195 -0.47553726
H 1.25754606 -5.63985148 -0.39661685
*

TSAB12
C -0.27857583 -3.42739433 0.12573938
C 0.80906740 -2.92808982 -0.59970669
C 1.01277020 -1.56869653 -0.72056420
C 0.12893288 -0.66147844 -0.12508240
C -0.95371733 -1.15147183 0.61268038
C -1.14916456 -2.51221025 0.73147681
H 1.50685131 -3.60931968 -1.07169537
H 1.86384962 -1.20125129 -1.28541149
H -1.63261662 -0.47656696 1.12235511
H -1.98397900 -2.88325741 1.31505552
C 0.38672364 0.76623744 -0.29445383
H 1.39903834 1.05344901 -0.57007231
H -0.30617952 1.25289020 -1.24786943
H -1.53858590 1.52273106 0.19890962
C -0.20796137 3.23140502 0.01960766
C -1.28138830 4.13025785 -0.01419328
C 1.10514921 3.71416989 0.10533374
C -1.04564544 5.49377972 0.01355435
H -2.29823596 3.75489553 -0.06653121
C 1.33389949 5.07646465 0.13554689
H 1.94406544 3.03160940 0.17656522
C 0.26059739 5.96395050 0.08465445
H -1.87550917 6.18918872 -0.01568320
H 2.34659164 5.45350955 0.21091454
H 0.44674896 7.03154495 0.11241271
C -0.51116429 1.81154238 -0.01251342
C -0.52371291 -4.87633949 0.28137225
C -0.18909645 -5.83289886 -0.84295004
C 0.63808190 -5.83997244 0.39565435
H -1.40234356 -5.11706045 0.86726928
H 0.23415023 -5.40710948 -1.74458914
H -0.87760484 -6.65125341 -1.00904036
H 0.52342274 -6.66318148 1.08882103
H 1.63505067 -5.41849898 0.35373836
*

TSAB13
C -0.97529506 -3.86094106 0.27770594
C 0.35339023 -3.53456287 0.01048125
C 0.73274236 -2.20595719 -0.07435040
C -0.21735056 -1.19323923 0.09765677
C -1.54687843 -1.53081558 0.38003079
C -1.92132498 -2.86121066 0.46717262
H 1.76985006 -1.95376215 -0.27925085
H -2.28756967 -0.75999601 0.56023971
H -2.94834244 -3.11968203 0.69435725
C 0.23081660 0.19006305 -0.00225954
H 1.30094884 0.36788404 0.08080334
H -0.05306807 0.72936466 -1.19334295
H -1.64497825 1.14614650 -0.31667782
C -0.14982475 2.71675348 -0.07530144
C -1.00534245 3.69948944 -0.58327828
C 1.06643019 3.08307116 0.51220004
C -0.63922581 5.03370778 -0.52536853
H -1.95453335 3.41549888 -1.02617620
C 1.42504291 4.41722962 0.57006739
H 1.72039403 2.33817004 0.95138844
C 0.57599581 5.38992051 0.04733090
H -1.30039750 5.79489169 -0.92118358
H 2.36079091 4.70549737 1.03343841
H 0.86102591 6.43445659 0.09887551
C -0.58201079 1.32292111 -0.16731011
H -1.24568841 -4.90899891 0.34481352
C 1.36580610 -4.62213019 -0.17818216
O 1.08625529 -5.78820218 -0.12290401
H 2.40133946 -4.28106312 -0.37452604
*

TSAB14
C 0.00127179 -3.44361641 0.01769597
C 1.24438747 -2.93939921 -0.31873079
C 1.40063009 -1.56440646 -0.35813867
C 0.31793316 -0.72417831 -0.07248565
C -0.92434191 -1.26885748 0.27895781
C -1.08670732 -2.64036127 0.32326982
H 2.05949032 -3.61561980 -0.53796196
H 2.36528851 -1.14067543 -0.61562500
H -1.75890086 -0.63167372 0.54547103
H -2.02844587 -3.09590057 0.59785874
C 0.54378393 0.71294747 -0.12768259
H 1.57912727 1.04805958 -0.12980164
H 0.04899957 1.28754944 -1.28913769
H -1.47434453 1.38293145 -0.25893610
C -0.21109243 3.15595621 -0.06013955
C -1.22987400 4.01901732 -0.47305032

C 0.97102645 3.67428012 0.47782276
C -1.06016915 5.38968592 -0.36938251
H -2.15234140 3.61539492 -0.87804404
C 1.13319831 5.04410437 0.58232641
H 1.75278038 3.01869101 0.84507415
C 0.12163362 5.90007882 0.15428663
H -1.84861991 6.05887863 -0.69155762
H 2.04285001 5.44888137 1.00915047
H 0.25308991 6.97241154 0.24187470
C -0.43929771 1.71289972 -0.20064839
N -0.17520050 -4.92253342 0.06733830
O -1.27524524 -5.32976500 0.36585721
O 0.79509181 -5.59576582 -0.19641985
*

TSAB15
C -0.12157022 -3.66652323 0.02724575
C 1.15357806 -3.14682139 -0.17186444
C 1.36212525 -1.78350955 -0.20226189
C 0.27410582 -0.92023783 -0.03617841
C -1.01291301 -1.42845363 0.17631340
C -1.19494086 -2.79291124 0.20343259
H 2.36834895 -1.41260690 -0.35897172
H -1.87367559 -0.79556577 0.35096290
C 0.53845892 0.50995015 -0.07790706
H 1.57523790 0.82049224 0.03120328
H 0.19671965 1.06700259 -1.26615424
H -1.44076912 1.22538309 -0.40623531
C -0.16629097 2.96734939 -0.08066711
C -1.13024174 3.84878809 -0.57974261
C 0.97788158 3.46557615 0.55213016
C -0.94225729 5.21614485 -0.46735282
H -2.02384463 3.46107973 -1.05824702
C 1.15832820 4.83202387 0.66411406
H 1.71304007 2.79656679 0.98524193
C 0.20256437 5.70536538 0.15034735
H -1.68760800 5.89930489 -0.85589186
H 2.03754571 5.22150413 1.16283117
H 0.34807596 6.77536583 0.24440212
C -0.41144278 1.53234245 -0.23360807
F -2.39981038 -3.30726081 0.41167719
F -0.31358996 -4.96767640 0.06322713
F 2.16658191 -3.98811774 -0.32909913
*

TSAB16
C 0.30136825 -3.93917188 -0.03942064
C 1.53313565 -3.34356767 -0.26756110
C 1.63524867 -1.96361838 -0.29488520
C 0.50979922 -1.16322785 -0.09981489
C -0.73831453 -1.76432507 0.14172414
C -0.84022821 -3.13467881 0.17218486
H 2.60127269 -1.50398388 -0.47357171
H -1.62294575 -1.16687146 0.33771178
C 0.68678539 0.28416908 -0.16363815
H 1.70135614 0.65213749 -0.03012732
H 0.38086809 0.73400876 -1.30333395
H -1.33866225 0.89126293 -0.41791634
C -0.17319225 2.68785641 -0.07445899
C -1.22609570 3.51089314 -0.49617408
C 0.97626673 3.25921649 0.49055533
C -1.12332747 4.88554529 -0.37693218
H -2.12045461 3.06724813 -0.92158967
C 1.07167601 4.63219290 0.60919461
H 1.78123152 2.63557465 0.86160802
C 0.02599198 5.44328595 0.17169467
H -1.93562333 5.52194278 -0.70600118
H 1.95406956 5.07699360 1.05290310
H 0.10600868 6.51984719 0.27095341
C -0.33216892 1.25485371 -0.22212961
O 0.20706580 -5.27329522 -0.00451041
H 2.39812646 -3.97783150 -0.41555615
H -0.70885106 -5.52922517 0.17475844
O -1.98369262 -3.83354669 0.41222350
H -2.73016354 -3.24869551 0.57618203
*

TSAB17
C -0.59603139 -3.22674575 0.08506432
C 0.75048050 -2.79468447 -0.09134486
C 1.02516407 -1.44503931 -0.11670766
C -0.01161539 -0.49906005 0.01960721
C -1.31831596 -0.92738067 0.20385532
C -1.60767746 -2.28547568 0.23761136
H 2.04344065 -1.10173132 -0.24817683
H -2.12365889 -0.21922463 0.36075564
H -2.62702112 -2.60619578 0.39989697
C 0.33987604 0.91737926 -0.02907125
H 1.35376581 1.18940321 0.25707479
H 0.26328164 1.36890357 -1.20548490
H -1.54805225 1.70104759 -0.61369548
C -0.29099304 3.39262917 -0.08661655
C -1.17319447 4.30324466 -0.68334615
C 0.79480585 3.86327239 0.66571684
C -0.96020245 5.66407386 -0.55147931
H -2.02114079 3.93755818 -1.25326095
C 1.00057275 5.22316398 0.79596791
H 1.45956700 3.17232471 1.17099078
C 0.12752849 6.12093742 0.18416162
H -1.64004295 6.36813238 -1.01544110
H 1.83289770 5.59096191 1.38361367
H 0.29248946 7.18682340 0.29346258
C -0.55813568 1.97847186 -0.25684517
O -0.77100857 -4.54838495 0.10444861
O 1.65682267 -3.77864866 -0.21601068
C -2.09430049 -5.05505391 0.28543107
H -2.49963920 -4.74215681 1.25126230
H -2.74927356 -4.72560324 -0.52546222
H -1.99756083 -6.13757677 0.26198148
C 3.02032511 -3.40593374 -0.38139985
H 3.15763257 -2.82271808 -1.29731797
H 3.37774947 -2.83821877 0.48331848
H 3.57483287 -4.33809901 -0.45816998
*

TSAB18
C 0.00780116 -3.38066684 0.09398398
C 1.27242355 -2.81183582 -0.19822318
C 1.42816306 -1.44910860 -0.26917118
C 0.35312350 -0.57450414 -0.05885729
C -0.89690684 -1.13048898 0.24735963
C -1.07306702 -2.48969233 0.32504232
H 2.13115417 -3.44561638 -0.36491996
H 2.41048440 -1.04581766 -0.49647985
H -1.74907960 -0.49307291 0.45929346
H -2.04855745 -2.87461701 0.58462775
C 0.58525012 0.86084715 -0.17485054
H 1.61280230 1.19679017 -0.05181617
H 0.30794235 1.26163222 -1.31054609
H -1.42715856 1.53587186 -0.36968901
C -0.19906147 3.29042676 -0.05928864
C -1.24596180 4.14522150 -0.43562371
C 0.99185343 3.83134110 0.45089720
C -1.09787783 5.51606727 -0.32674098
H -2.17104694 3.72582695 -0.81745959
C 1.13293214 5.20076859 0.55840363
H 1.79359360 3.18431841 0.78656033
C 0.09184598 6.04086721 0.16602876
H -1.90529399 6.17560273 -0.62036294
H 2.04737927 5.62135140 0.95872468
H 0.20790282 7.11494339 0.25694672
C -0.40670943 1.86818350 -0.19057654

N -0.16164262 -4.72014047 0.16429846
C -1.46916857 -5.28238087 0.47151945
C 0.96791779 -5.61458469 -0.04665305
H -1.39966795 -6.36709898 0.45445551
H -1.81192177 -4.97829046 1.46530385
H -2.21490727 -4.97838207 -0.26864624
H 0.62858679 -6.64235488 0.05470662
H 1.38944087 -5.49221642 -1.04869517
H 1.75609545 -5.44127016 0.69214632
*

TSAB19
C -0.03736449 -2.81813601 0.01300937
C 1.24360699 -2.31565950 -0.18999389
C 1.42332398 -0.93772799 -0.20839027
C 0.32991232 -0.08013242 -0.05453402
C -0.94896750 -0.60724868 0.15414783
C -1.13710320 -1.98126718 0.19517774
H 2.42260517 -0.54215002 -0.35146809
H -1.80767156 0.03262681 0.31383326
C 0.57770853 1.35167162 -0.08474250
H 1.61380255 1.67184912 0.01033603
H 0.17567980 1.98669590 -1.32792374
H -1.40927497 2.05281812 -0.42969610
C -0.14360598 3.80611811 -0.09477728
C -1.08610427 4.69078396 -0.62526474
C 0.96963521 4.29615927 0.59405625
C -0.90632516 6.05701142 -0.48550456
H -1.95672911 4.30906940 -1.14900100
C 1.14127135 5.66191916 0.73365007
H 1.68659564 3.62378798 1.05259880
C 0.20800383 6.54061922 0.18970875
H -1.63552814 6.74359612 -0.89794825
H 1.99572635 6.04534505 1.27803633
H 0.34606710 7.60941580 0.30526731
C -0.37678840 2.36467726 -0.28380667
N -0.23774316 -4.28151523 0.04853261
O -0.01617313 -4.86328719 -0.98542635
O -0.60148782 -4.73362846 1.10666510
C -2.45932249 -2.50719264 0.41147377
N -3.53489484 -2.87738497 0.56521380
C 2.37950306 -3.18359448 -0.35532227
N 3.31451836 -3.83853156 -0.47481429
*

TSAB2
C -0.04276950 -3.76311370 0.02343969
C 1.18503425 -3.24686030 -0.37624212
C 1.35167091 -1.87607608 -0.42735024
C 0.29815260 -1.01878369 -0.09041309
C -0.92639949 -1.55796101 0.32309997
C -1.09879712 -2.92584705 0.37937521
H 1.99340659 -3.91737017 -0.63745947
H 2.30774745 -1.46587358 -0.73563698
H -1.74382395 -0.91610759 0.63089587
H -2.03654075 -3.35527765 0.70753443
C 0.53341113 0.41833000 -0.16815328
H 1.57037913 0.74665560 -0.18403173
H 0.06183691 0.93185115 -1.27780493
H -1.48200456 1.09996607 -0.21156882
C -0.21055213 2.86012260 -0.04196226
C -1.25909838 3.72252807 -0.38077828
C 1.00709029 3.38296700 0.41077843
C -1.08581584 5.09309710 -0.29036957
H -2.20681264 3.31557760 -0.71834889
C 1.17290068 4.75225096 0.50201813
H 1.81390338 2.72817807 0.71978779
C 0.13003073 5.60524409 0.14705161
H -1.89623666 5.76107890 -0.55531369
H 2.10970194 5.16054213 0.86133124
H 0.26522012 6.67800264 0.22431478
C -0.44538355 1.42595212 -0.15929904
Cl -0.25983363 -5.46649782 0.09696426
*

TSAB20
C -0.05839849 -2.15353180 0.00193019
C 1.24273248 -1.67092774 -0.09300798
C 1.44012246 -0.30133367 -0.11323607
C 0.35488537 0.57470778 -0.04114919
C -0.94192297 0.06542377 0.07128426
C -1.15737857 -1.29885187 0.08553658
H 2.45005969 0.08324252 -0.19301247
H -1.79525057 0.72260700 0.17522996
C 0.62750584 2.00276132 -0.05269928
H 1.65811264 2.30678355 0.12047079
H 0.34202855 2.61823055 -1.29944018
H -1.31840426 2.73445519 -0.53179255
C -0.05406920 4.46729216 -0.10640879
C -0.95186839 5.36400281 -0.69189414
C 1.02407225 4.94420449 0.64530641
C -0.76135930 6.72796357 -0.54484677
H -1.79602591 4.99260214 -1.26406300
C 1.20657105 6.30765747 0.79180129
H 1.70362317 4.26269138 1.14516752
C 0.31868706 7.19770584 0.19293526
H -1.45563551 7.42360431 -1.00015710
H 2.03354937 6.68082370 1.38367398
H 0.46490899 8.26481693 0.31413852
C -0.29669952 3.03103804 -0.30313421
N -0.28460235 -3.62000807 0.02526904
O -0.79633346 -4.08513873 -0.96315392
O 0.06662924 -4.18909839 1.02872646
C -2.59250643 -1.81109604 0.18782459
F -3.38625030 -0.84955833 0.68957594
F -2.67618025 -2.87231356 0.99096182
F -3.06385369 -2.14017001 -1.01615363
C 2.46281112 -2.58709271 -0.15848502
F 3.50055405 -1.92407116 -0.69528057
F 2.22572854 -3.65984215 -0.91544330
F 2.82117631 -2.98792932 1.06184958
*

TSAB21
C -0.02628994 -3.05354706 0.00851318
C 1.22469247 -2.52062597 -0.25445357
C 1.41191007 -1.15182887 -0.29188423
C 0.31693393 -0.31321019 -0.06085624
C -0.94918288 -0.83949656 0.22090421
C -1.10331714 -2.20946922 0.24567901
H 2.40014636 -0.76116253 -0.50374924
H -1.80719344 -0.22069688 0.44907809
C 0.55412182 1.12143364 -0.10439158
H 1.59161201 1.44796512 -0.07183553
H 0.08733153 1.72359537 -1.30060559
H -1.45439375 1.80521534 -0.31454601
C -0.18535641 3.56993710 -0.06874864
C -1.17652422 4.44236322 -0.52601883
C 0.97485776 4.07602474 0.52483634
C -1.00016411 5.81124638 -0.40979597
H -2.08261301 4.04820369 -0.97497103
C 1.14345686 5.44421971 0.64147107
H 1.73339716 3.41326978 0.92661945
C 0.16024942 6.31013676 0.16977098
H -1.76727536 6.48796051 -0.76597504
H 2.03560436 5.83972875 1.11171215
H 0.29650087 7.38101554 0.26696615
C -0.41872779 2.12720301 -0.22749057
N -0.21452016 -4.51602112 0.04863545
O -0.80248603 -4.99433469 -0.89088027
O 0.23826944 -5.07909954 1.01507651
F -2.28535262 -2.73946593 0.52050279

F 2.24178077 -3.33700211 -0.48026404
*

TSAB22
C 0.09209055 -2.65924678 -0.01428412
C 1.36799180 -2.06738313 -0.18980177
C 1.50460143 -0.70195747 -0.22646636
C 0.39881375 0.15121143 -0.09600935
C -0.86424188 -0.42994254 0.09065168
C -1.02256360 -1.79234211 0.13307074
H 2.24643334 -2.69125232 -0.27826121
H 2.49549689 -0.27730879 -0.35786424
H -1.74329582 0.19077479 0.23040199
H -2.01001361 -2.20533157 0.28571416
C 0.61467608 1.59231992 -0.17250501
H 1.61988317 1.94298589 0.05242498
H 0.44218370 1.99803123 -1.31978981
H -1.38544476 2.23879914 -0.53022188
C -0.22278792 4.00469646 -0.07629896
C -1.24938438 4.85011633 -0.52451339
C 0.90986011 4.55520880 0.54530341
C -1.13669487 6.22048548 -0.37643838
H -2.13029004 4.42331510 -0.99280808
C 1.01566138 5.92409835 0.69134902
H 1.69162982 3.91478583 0.93607642
C -0.00345418 6.75462452 0.22722218
H -1.92795572 6.87253780 -0.72551310
H 1.88438682 6.35158277 1.17708748
H 0.08441108 7.82835678 0.34883752
C -0.39176002 2.58371740 -0.25200656
N -0.05708780 -3.99454044 0.02538938
C -1.32718174 -4.66866907 0.30728303
C 1.03646994 -4.94341751 -0.19972675
H -1.81173795 -4.22872500 1.18191376
H -2.00701615 -4.57624367 -0.54777008
C -0.91454614 -6.12106029 0.54339721
H 1.60141774 -4.67439531 -1.09509457
H 1.72328299 -4.94435706 0.65481367
C 0.31901516 -6.28455276 -0.34638317
H -0.64345988 -6.26230348 1.59271513
H -1.71473636 -6.82034491 0.30198715
H 0.01697147 -6.43059671 -1.38658439
H 0.95116361 -7.12308408 -0.05517776
*

TSAB23
C -0.22251740 -3.18302073 0.11987750
C 1.06989042 -2.61626207 -0.03273596
C 1.28856443 -1.26922175 -0.09820943
C 0.21830969 -0.36725317 0.00234103
C -1.06374212 -0.88820242 0.19657377
C -1.27676477 -2.24681121 0.25619338
H 2.31189375 -0.92526473 -0.20492094
H -1.91776708 -0.23176096 0.32107769
H -2.28489143 -2.60709422 0.40110584
C 0.50096392 1.05950653 -0.09121482
H 1.51931015 1.37140052 0.13100685
H 0.34304069 1.47509203 -1.25638095
H -1.45826749 1.77886402 -0.51916435
C -0.22482700 3.50901130 -0.09287580
C -1.19355556 4.38577879 -0.60237721
C 0.91007499 4.02363634 0.55271277
C -1.02041737 5.75362212 -0.49052305
H -2.07732467 3.98680206 -1.08949412
C 1.07608774 5.39027221 0.66281620
H 1.64628100 3.36016038 0.99103991
C 0.11508563 6.25288596 0.13785950
H -1.76716827 6.43056961 -0.88704470
H 1.94670250 5.79101500 1.16758382
H 0.24987174 7.32454211 0.23118429
C -0.45469027 2.08912766 -0.23569158
N -0.44374798 -4.51657279 0.17727151
C -1.74145547 -5.01857073 0.60736540
C 0.46671949 -5.50271721 -0.41122817
H -1.62940861 -6.06497125 0.88644500
H -2.09086747 -4.47592516 1.48677156
H -2.49635738 -4.94780763 -0.18444399
H -0.13229717 -6.33920680 -0.77078255
H 0.99742791 -5.08226875 -1.26231363
H 1.19206458 -5.87267596 0.31608358
F 2.14907689 -3.42353909 -0.05730336
*

TSAB24
C -0.16535347 -2.69119895 -0.02744358
C 1.13786396 -2.21615497 -0.08743638
C 1.38211450 -0.85489206 -0.08554589
C 0.31085030 0.04293865 -0.03356101
C -1.00231227 -0.43600858 0.04154912
C -1.23024990 -1.79756237 0.04045154
H 2.40197072 -0.49071236 -0.13329011
H -1.84463413 0.23939707 0.12049729
C 0.61596543 1.46565249 -0.03350481
H 1.64526360 1.74683668 0.17991609
H 0.40222621 2.06095531 -1.26442239
H -1.29629713 2.24033512 -0.56840357
C -0.01057471 3.94386799 -0.09884373
C -0.88025705 4.85807844 -0.70010849
C 1.06472004 4.40081701 0.66989191
C -0.66442287 6.21816756 -0.55263419
H -1.72243517 4.50232536 -1.28493290
C 1.27295923 5.76037258 0.81637793
H 1.72205883 3.70619249 1.18098926
C 0.41313403 6.66707288 0.20151358
H -1.33680497 6.92700540 -1.02028137
H 2.09815286 6.11803873 1.42019518
H 0.57949270 7.73125205 0.32280096
C -0.27956544 2.51509522 -0.29541073
C -2.63930226 -2.34745002 0.14657567
F -3.55542056 -1.38009351 -0.01893137
F -2.83963044 -2.90443097 1.34879611
F -2.85291870 -3.28704132 -0.78308892
C 2.28219503 -3.21030795 -0.12653204
F 3.44272469 -2.60678575 -0.42380403
F 2.05730592 -4.15902534 -1.04428642
F 2.42239965 -3.81309171 1.06265745
H -0.35429364 -3.75937017 -0.03021914
*

TSAB3
C -1.49393485 -3.09005438 0.30701061
C -0.14707960 -2.86755881 0.03907274
C 0.33913743 -1.57736815 -0.05230354
C -0.53175967 -0.49526629 0.11670731
C -1.88354791 -0.72176561 0.39963693
C -2.35919041 -2.01749571 0.49176987
H 1.39079978 -1.40939975 -0.25437758
H -2.56041589 0.10588160 0.57727112
H -3.40211841 -2.19798486 0.72080688
C 0.02901043 0.84553276 0.01431835
H 1.11042344 0.93429318 0.09495387
H -0.21336708 1.41321494 -1.18702977
H -1.76168151 1.94963694 -0.31184911
C -0.14187610 3.39465846 -0.07579522
C -0.90829912 4.44150753 -0.59700461
C 1.09513599 3.66201646 0.52076642
C -0.43322133 5.74113601 -0.54278700
H -1.87379329 4.23460737 -1.04738556
C 1.56265538 4.96240158 0.57509109
H 1.68158031 2.86822865 0.96968774
C 0.80216317 5.99899500 0.03939441
H -1.02546387 6.55201250 -0.94894579

H 2.51503387 5.17458293 1.04562412
H 1.17243124 7.01656708 0.08807328
C -0.68736562 2.03925073 -0.16566882
C 0.76906032 -4.05519219 -0.17036730
H -1.86391038 -4.10601656 0.38661781
F 0.58479380 -4.97796127 0.78380300
F 2.06062525 -3.69046292 -0.15226252
F 0.52442007 -4.63880584 -1.35436933
*

TSAB4
C 0.05217147 -3.04574409 -0.01215935
C 1.26373800 -2.50670458 -0.41583089
C 1.41032625 -1.12895166 -0.46010603
C 0.34373657 -0.29704425 -0.11016953
C -0.87201235 -0.85526735 0.30924971
C -1.01476770 -2.22620280 0.35536037
H 2.08560696 -3.15538616 -0.68907277
H 2.35606142 -0.69815201 -0.77137688
H -1.69596291 -0.22693239 0.62590648
H -1.94658086 -2.66873342 0.68660824
C 0.55610417 1.14262434 -0.17430301
H 1.58725714 1.48543157 -0.22974330
H 0.00899336 1.69703563 -1.28815048
H -1.47004512 1.79691461 -0.17581290
C -0.21341508 3.57779245 -0.04094684
C -1.26869351 4.43162702 -0.37514017
C 0.99910714 4.10750404 0.41293342
C -1.10677256 5.80357990 -0.27783785
H -2.21351199 4.01902253 -0.71411412
C 1.15365871 5.47839484 0.51147308
H 1.81205452 3.45870429 0.71915362
C 0.10436230 6.32466501 0.16155092
H -1.92343495 6.46543038 -0.53911954
H 2.08735592 5.89188125 0.87300849
H 0.23031408 7.39806685 0.24435885
C -0.43626166 2.13548887 -0.16821551
C -0.14784991 -4.54837175 0.05677184
F -1.15540462 -4.92240199 -0.74744940
F -0.46404301 -4.92136436 1.30652199
F 0.94955724 -5.21765277 -0.31228344
*

TSAB5
C -0.11609103 -4.22217726 0.05569478
C 1.11992410 -3.71244621 -0.35367061
C 1.30223066 -2.35009933 -0.41832452
C 0.26397861 -1.46702179 -0.08746407
C -0.96366545 -1.99006884 0.33356148
C -1.15629700 -3.35331232 0.40572112
H 1.91432114 -4.40291113 -0.60754451
H 2.26359313 -1.95716723 -0.73363621
H -1.77265861 -1.33525751 0.63781514
H -2.10537173 -3.75253579 0.74749855
C 0.51747322 -0.03305608 -0.18751206
H 1.55786913 0.28367558 -0.16206412
H 0.11982408 0.43341391 -1.29819652
H -1.48889566 0.67291206 -0.25926168
C -0.20736076 2.41261147 -0.04795019
C -1.25072172 3.28547197 -0.38325375
C 1.01536954 2.92710376 0.40624493
C -1.06783778 4.65386665 -0.28906165
H -2.20104095 2.88547971 -0.72166194
C 1.19074965 4.29416021 0.50072385
H 1.81710920 2.26477336 0.71157363
C 0.15271138 5.15515456 0.14888524
H -1.87306505 5.32908964 -0.55135510
H 2.13056533 4.69571499 0.85967386
H 0.29576190 6.22677322 0.22889165
C -0.45157706 0.98791067 -0.16577497
O -0.23787272 -5.55826679 0.10596117
H -1.11885701 -5.81441180 0.40326039
*

TSAB6
C 0.14475146 -3.77023426 -0.03726125
C 1.38219675 -3.19450600 -0.36659603
C 1.51510918 -1.82869872 -0.40299074
C 0.42202957 -0.99261247 -0.12131554
C -0.80126830 -1.57378908 0.21958678
C -0.94868297 -2.94695694 0.26386881
H 2.21659877 -3.84998880 -0.58258723
H 2.47654000 -1.39326000 -0.65639100
H -1.65258128 -0.95677543 0.48606625
H -1.90215799 -3.37051304 0.54615774
C 0.61824096 0.45238425 -0.18821409
H 1.63891690 0.81401533 -0.08342454
H 0.28451063 0.91096762 -1.31699879
H -1.40851618 1.06925636 -0.38758929
C -0.22654833 2.86019184 -0.06129747
C -1.28563980 3.69095257 -0.45084960
C 0.93931685 3.42202923 0.47891262
C -1.17303753 5.06430846 -0.32495481
H -2.19260550 3.25413934 -0.85608295
C 1.04466670 4.79371345 0.60420849
H 1.74972730 2.79158902 0.82584821
C -0.00759422 5.61272115 0.19838763
H -1.99026416 5.70682270 -0.62898653
H 1.93996682 5.23125527 1.02888665
H 0.08016955 6.68818409 0.30296896
C -0.39613281 1.42908073 -0.21535020
O 0.11160227 -5.10521493 -0.02350860
C -1.11666315 -5.75896297 0.30750993
H -0.90660806 -6.82374433 0.24582625
H -1.42844645 -5.50380783 1.32344431
H -1.89936129 -5.49593304 -0.40845626
*

TSAB7
C 0.14090832 -3.86748347 0.06947540
C 1.35588168 -3.28366848 -0.30076936
C 1.49206496 -1.91010757 -0.36707603
C 0.40955858 -1.08045792 -0.06236199
C -0.80443821 -1.65659123 0.33499640
C -0.93205642 -3.02686346 0.40091152
H 2.20037332 -3.91962022 -0.54056841
H 2.44260738 -1.47564203 -0.65979097
H -1.64385787 -1.03657515 0.62956639
H -1.86701510 -3.45437512 0.74003091
C 0.60280690 0.36386412 -0.15389032
H 1.62945574 0.72310992 -0.14567170
H 0.15574283 0.82820807 -1.26635197
H -1.43167371 0.98122662 -0.22036198
C -0.22259090 2.77961388 -0.04036950
C -1.30183122 3.60393567 -0.38081357
C 0.97776069 3.34826985 0.40593399
C -1.17650876 4.97998694 -0.29875121
H -2.23543161 3.16168958 -0.71317668
C 1.09579363 4.72263743 0.48849958
H 1.80775253 2.72348447 0.71514197
C 0.02225575 5.53644502 0.13193047
H -2.01032652 5.61799421 -0.56493068
H 2.01881397 5.16595039 0.84191009
H 0.12020147 6.61375127 0.20243632
C -0.40771817 1.34120850 -0.14646642
C 0.03859133 -5.33614882 0.11646530
C -1.09539981 -6.02645736 0.17783548
H 0.98337425 -5.87010099 0.07072915
H -1.08270702 -7.10946374 0.19747383
H -2.07026411 -5.55179615 0.19675232
*

TSAB8

C	-0.04288249	-3.93382387	0.03152121
C	1.18369976	-3.39662874	-0.37081146
C	1.35197063	-2.02679601	-0.42306286
C	0.29810284	-1.17133752	-0.08345693
C	-0.92549957	-1.71034329	0.33266614
C	-1.09387886	-3.07796065	0.38818714
H	1.99585025	-4.06173664	-0.63548094
H	2.30633528	-1.61397141	-0.73330987
H	-1.74173051	-1.06770629	0.64217346
H	-2.03345947	-3.50233740	0.71879984
C	0.53202871	0.26657600	-0.16356413
H	1.56871578	0.59556051	-0.18281323
H	0.05781590	0.77363076	-1.27085538
H	-1.48429675	0.94469783	-0.19791825
C	-0.21570037	2.70739023	-0.03711856
C	-1.26899672	3.56684352	-0.36881348
C	1.00427181	3.23409316	0.40491708
C	-1.09844198	4.93797950	-0.28193314
H	-2.21833930	3.15689533	-0.69804960
C	1.16746086	4.60390870	0.49258583
H	1.81520219	2.58166504	0.70797537
C	0.11963033	5.45379548	0.14481796
H	-1.91262951	5.60355058	-0.54135409
H	2.10619848	5.01508385	0.84347234
H	0.25281452	6.52701153	0.21924832
C	-0.44819728	1.27281498	-0.14976884
C	-0.22254593	-5.35385138	0.09328879
C	-0.37345258	-6.54129873	0.14475037
H	-0.50745025	-7.59928537	0.18967458

*

TSAB9

C	-0.03966022	-3.93058140	0.03217093
C	1.18709674	-3.40455842	-0.37117150
C	1.34999150	-2.03269844	-0.42156866
C	0.29039222	-1.18399792	-0.08080749
C	-0.93328289	-1.72331454	0.33683822
C	-1.09881905	-3.09218024	0.39193709
H	1.99907223	-4.06965704	-0.63666801
H	2.30229756	-1.61557334	-0.73106283
H	-1.74983782	-1.08187328	0.64607937
H	-2.03612375	-3.52286929	0.72100524
C	0.52071876	0.25172630	-0.15241332
H	1.55636871	0.58120633	-0.20619929
H	-0.01911147	0.81450322	-1.27843891
H	-1.49743563	0.93304803	-0.17478835
C	-0.21711142	2.69804358	-0.04345195
C	-1.25593346	3.56466834	-0.39557516
C	0.99771591	3.21303508	0.42080795
C	-1.07504279	4.93475420	-0.30552273
H	-2.20273937	3.16370449	-0.74290365
C	1.17112184	4.58221879	0.51207966
H	1.79763317	2.55478045	0.74100544
C	0.13845225	5.44125442	0.14433298
H	-1.87889665	5.60657012	-0.58071745
H	2.10650608	4.98449471	0.88181053
H	0.27912487	6.51324393	0.22138230
C	-0.45910781	1.25755481	-0.16541526
C	-0.21643713	-5.35944517	0.09213984
N	-0.35960126	-6.49824365	0.13862612

*

TSCD0

N	0.14316320	1.17047686	-1.59548764
O	1.43411759	1.03170860	-1.22059938
C	-0.68160949	1.88824197	-0.82949049
C	-0.37464335	0.07096613	-2.37962838
H	-1.69303979	1.96562452	-1.23982744
C	-0.13760092	3.10994016	-0.14432702
H	0.27914710	-0.09118002	-3.24953380
H	-0.39582745	-0.85420434	-1.77463432
H	-1.39381367	0.29715481	-2.72605867
H	0.19785779	3.83103137	-0.90906514
H	-0.91678500	3.58144083	0.47016358
H	0.72788606	2.86746787	0.48669835
C	-0.20934285	0.54213580	1.42707658
C	-1.25654978	0.69831356	0.71805722
O	0.85823550	0.52847894	1.99321317
Li	1.97493036	0.12910433	0.30723634
O	1.79045245	-1.80022208	0.16469958
O	3.83551246	0.63795834	0.64612337
C	1.47266178	-2.70424114	1.20447616
H	0.59851041	-3.32313046	0.93226387
H	2.32713480	-3.37117891	1.42149150
H	1.23606710	-2.11510116	2.10038064
C	2.06404123	-2.42625741	-1.07376700
H	1.19170477	-3.00973224	-1.41996073
H	2.28235069	-1.63627457	-1.80507916
H	2.93302497	-3.10425280	-0.98942816
C	4.73382982	0.10321881	-0.30941996
H	5.77945549	0.32337534	-0.02869836
H	4.59304726	-0.98643002	-0.32943601
H	4.53574915	0.51642238	-1.31481782
C	3.96506921	2.04067514	0.80572037
H	3.76157068	2.56659625	-0.14347078
H	3.23439522	2.35928444	1.56220237
H	4.98068788	2.29927181	1.15564707
C	-2.51642914	-0.01478470	0.51529942
C	-3.66678966	0.65355792	0.05657885
C	-2.61203708	-1.40198727	0.75574673
C	-4.86389683	-0.03573536	-0.15298965
H	-3.62555295	1.73161637	-0.12500172
C	-3.80852785	-2.08674538	0.55086499
H	-1.72430910	-1.93964232	1.10378796
C	-4.94275069	-1.40810916	0.09175326
H	-5.74487499	0.50891074	-0.50684905
H	-3.85586351	-3.16251399	0.74720126
H	-5.88080590	-1.94658393	-0.07229193

*

TSCD1

N	-1.78172032	-0.48122030	-1.29924720
O	-0.59273599	0.14452272	-1.21892580
C	-2.76997737	-0.11883404	-0.48768336
C	-1.71504216	-1.83130085	-1.81330724
H	-3.70052550	-0.66857909	-0.65576790
C	-2.88882022	1.32644128	-0.09673608
H	-1.21032879	-1.82213349	-2.79073732
H	-1.13684155	-2.47054662	-1.11989468
H	-2.72711343	-2.24542834	-1.92881873
H	-3.07931993	1.92923082	-1.00167817
H	-3.72325171	1.46540769	0.60393155
H	-1.96266297	1.69649663	0.36264216
C	-1.33476680	-0.54051757	1.80918422
C	-2.42284519	-1.00788209	1.35768281
H	-3.10710826	-1.81884632	1.57915395
O	-0.29730459	0.03055712	2.08290849
Li	0.54532823	0.14568394	0.25186694
O	1.82699642	-1.30317302	0.15872416
O	1.63884725	1.77583434	0.20963266
C	2.73459652	-1.59738232	1.20042616
H	2.68624621	-2.66674087	1.47593025
H	3.77053239	-1.35096683	0.90296750
H	2.45370986	-0.98900076	2.07113030
C	2.06173501	-2.02244531	-1.03537022
H	1.89657243	-3.10478788	-0.88185988
H	1.35396859	-1.64710775	-1.78636518
H	3.09654622	-1.86802318	-1.39270338
C	2.40031166	1.86762906	-0.98014764
H	3.17743937	2.64777090	-0.88862465
H	2.88497594	0.89452384	-1.14115796

H 1.75534655 2.09926111 -1.84656701
C 0.95527252 2.97402994 0.52784839
H 0.26121770 3.26266346 -0.28202510
H 0.38692280 2.79845154 1.45149352
H 1.67124713 3.79903521 0.69597245
*

TSCD14
N 1.20588017 1.41320464 -1.45210965
O 2.41724840 0.91428238 -1.12483895
C 0.57897166 2.20877573 -0.58075189
C 0.43581118 0.57938181 -2.34868496
H -0.37896484 2.58284465 -0.95469488
C 1.40161792 3.17352255 0.22691011
H 1.03290412 0.36944930 -3.24850407
H 0.18427806 -0.37966928 -1.85891514
H -0.49391878 1.09054416 -2.63895309
H 1.88763108 3.88926594 -0.45757585
H 0.76077138 3.72989312 0.92458700
H 2.19338274 2.65958170 0.78804202
C 0.74181958 0.50420397 1.47104624
C -0.23764561 1.00464435 0.81746212
O 1.76096675 0.16233081 2.00822228
Li 2.76218368 -0.28993837 0.23396538
O 2.10449795 -2.08310483 -0.10286681
O 4.68189867 -0.26927686 0.57855599
C 1.62975942 -3.01764113 0.84682481
H 0.62795439 -3.38879424 0.56436791
H 2.31813866 -3.87841344 0.92970806
H 1.56921767 -2.50877423 1.81798247
C 2.19136157 -2.59851928 -1.41795820
H 1.19830242 -2.92180763 -1.77897757
H 2.56698423 -1.79527870 -2.06619864
H 2.88193333 -3.46054818 -1.45836083
C 5.44089773 -0.87223484 -0.45465492
H 6.50314489 -0.95300801 -0.16237308
H 5.03964326 -1.88186753 -0.62036327
H 5.36786735 -0.29110916 -1.39153351
C 5.13880081 1.02901212 0.91997048
H 5.08055814 1.70914298 0.05223371
H 4.49301212 1.40887210 1.72396408
H 6.18110330 0.99025772 1.28415611
C -1.61299725 0.59722608 0.59035480
C -2.59722285 1.53385699 0.20902951
C -2.00258071 -0.75652759 0.72724571
C -3.91033299 1.14427000 -0.02865958
H -2.33054616 2.58901022 0.11294052
C -3.30965455 -1.15809623 0.49681232
H -1.25201588 -1.49848830 1.01310984
C -4.25434264 -0.20020987 0.11563680
H -3.60044959 -2.20408796 0.60427265
H -4.66582431 1.87614053 -0.31831367
N -5.63303128 -0.61712929 -0.13679895
O -5.91108409 -1.79773232 -0.01227703
O -6.44398582 0.23328630 -0.46151543
*

TSCD15
N 0.92616058 1.27243943 -1.56939056
O 2.19158218 0.96737961 -1.21209184
C 0.16929806 1.98473979 -0.73083725
C 0.30899533 0.31326279 -2.45850517
H -0.82406886 2.20692274 -1.13301403
C 0.82441616 3.06961722 0.07738661
H 0.94356704 0.18370721 -3.34787557
H 0.20449861 -0.66481094 -1.95221616
H -0.68520503 0.66693732 -2.76886258
H 1.23996811 3.82773843 -0.60794027
H 0.08836964 3.55164697 0.73539545
H 1.65263549 2.67861247 0.68331719
C 0.49098726 0.33471131 1.34979817
C -0.52793302 0.71340628 0.68217771
O 1.54209490 0.11592322 1.89799985
Li 2.73996277 -0.04927174 0.22764453
O 2.75991041 -1.94762459 -0.13030942
O 4.55086953 0.42432620 0.78281902
C 2.95361283 -2.92823076 0.86899410
H 2.15856753 -3.69484667 0.82709998
H 3.93525116 -3.42277627 0.75146416
H 2.91652936 -2.42469859 1.84477159
C 2.78716349 -2.45423009 -1.45051296
H 1.94710078 -3.15209043 -1.62111277
H 2.69569074 -1.59800069 -2.13190287
H 3.73625341 -2.98483096 -1.64972655
C 5.52354149 0.12330655 -0.20146200
H 6.54034578 0.16052979 0.22893910
H 5.32475222 -0.89434659 -0.56602611
H 5.46371803 0.83105081 -1.04772266
C 4.69428313 1.72234131 1.33172452
H 4.60032521 2.49660718 0.54927862
H 3.90004811 1.85901428 2.07861794
H 5.67617372 1.82967965 1.82700544
C -1.88797259 0.23345479 0.47306248
C -2.92211059 1.13194262 0.14991690
C -2.19564933 -1.13955301 0.57076699
C -4.21193291 0.66245551 -0.05983042
H -2.74209073 2.20652746 0.07652469
C -3.49352870 -1.57742772 0.36073324
H -1.42248537 -1.87440197 0.80622752
C -4.52045317 -0.69066243 0.04001084
F -3.79278312 -2.87921683 0.45372039
F -5.76703774 -1.12961600 -0.16395932
F -5.20019761 1.51454151 -0.35967650
*

TSCD18
N 1.26076037 1.42963430 -1.43926063
O 2.50063114 0.96444233 -1.16677199
C 0.64409755 2.20431958 -0.54399487
C 0.48609504 0.58709345 -2.32338961
H -0.33716057 2.55403570 -0.87895340
C 1.47045083 3.17740801 0.24850697
H 1.05554852 0.41596700 -3.24936036
H 0.28600584 -0.38940404 -1.84559037
H -0.47153230 1.07018295 -2.56738169
H 1.94281042 3.89532762 -0.44342222
H 0.83522511 3.73000695 0.95436555
H 2.27332672 2.67145549 0.80110707
C 0.86172755 0.54356824 1.51915858
C -0.14922352 0.97781109 0.88096544
O 1.92750209 0.25636094 2.02089098
Li 2.85916839 -0.18480782 0.25102253
O 2.25521073 -2.00398689 -0.08478492
O 4.80035924 -0.13280392 0.53089794
C 1.77649322 -2.93242382 0.86755318
H 0.78233259 -3.31679182 0.57542958
H 2.47159993 -3.78598447 0.97005890
H 1.69622755 -2.41250074 1.83143560
C 2.34740785 -2.52461091 -1.39629988
H 1.35875130 -2.86519153 -1.75409860
H 2.70823860 -1.71809550 -2.04879809
H 3.05073784 -3.37670705 -1.43443207
C 5.52439748 -0.69343867 -0.54915662
H 6.60407551 -0.73221478 -0.31781781
H 5.15711683 -1.71757101 -0.70369619
H 5.37386227 -0.10754356 -1.47363307
C 5.23134858 1.17434539 0.86927792
H 5.11888452 1.86179459 0.01263749
H 4.60678858 1.52559123 1.70245741
H 6.28838842 1.16385700 1.19122936
C -1.53126999 0.57103418 0.61990744
C -2.53537242 1.50525226 0.31831431

C -1.91831320 -0.78246651 0.63936803
C -3.84918241 1.12159436 0.04864087
H -2.28962249 2.57188461 0.30744647
C -3.22475514 -1.18345732 0.37645361
H -1.16545051 -1.54389321 0.86880605
C -4.23098899 -0.23929723 0.05813609
H -3.45856786 -2.24827688 0.41423620
H -4.58192798 1.89952581 -0.16987686
N -5.52701247 -0.63258247 -0.23016036
C -6.56346282 0.37180332 -0.34961539
C -5.91633698 -2.01050588 -0.01365160
H -7.51511689 -0.11953845 -0.59196464
H -6.70477328 0.95468275 0.58181364
H -6.34327245 1.08484637 -1.16191121
H -6.96423665 -2.13993486 -0.31533129
H -5.30986521 -2.70193574 -0.62235708
H -5.82237149 -2.32204847 1.04537963
*

TSCD19
N 1.69739693 1.66492041 -1.28215347
O 2.89710857 1.10059847 -1.03003951
C 1.10971675 2.37911234 -0.31600413
C 0.88649263 0.94703393 -2.24088963
H 0.15535585 2.81556538 -0.62759407
C 1.97506550 3.23995674 0.56228741
H 1.46079389 0.81145503 -3.16920043
H 0.61477025 -0.04892614 -1.84409318
H -0.03188079 1.51111484 -2.46056206
H 2.44405639 4.02246049 -0.05759017
H 1.36886964 3.72130428 1.34178842
H 2.78042115 2.65796922 1.02984775
C 1.30739551 0.46452798 1.54257108
C 0.32690701 1.05016337 0.95568295
O 2.31534303 0.04882077 2.03361873
Li 3.26553455 -0.26311132 0.15095732
O 2.51826973 -1.98730539 -0.31475874
O 5.18318720 -0.34702550 0.46053001
C 2.08385027 -3.01000563 0.56172722
H 1.06164058 -3.34056990 0.30281754
H 2.76199660 -3.88128423 0.51482784
H 2.08857316 -2.60419786 1.58204917
C 2.52616757 -2.37117099 -1.67786270
H 1.50946882 -2.64341158 -2.01390088
H 2.88339864 -1.51356900 -2.26396614
H 3.19720880 -3.23424134 -1.83889756
C 5.90933932 -0.84499854 -0.64999619
H 6.96104238 -1.03819913 -0.37315497
H 5.44271993 -1.79091525 -0.95921299
H 5.88330130 -0.13179043 -1.49336753
C 5.71248635 0.86368469 0.97568467
H 5.68353831 1.66323518 0.21465297
H 5.09633031 1.15804651 1.83671264
H 6.75454647 0.71914798 1.31248607
C -1.02669415 0.62134339 0.68051279
C -2.04279791 1.55120461 0.39112067
C -1.37192624 -0.74702685 0.66469436
C -3.34806622 1.14138881 0.10202877
H -1.82291507 2.62007837 0.40007507
C -2.66394447 -1.17306248 0.36063204
H -0.60997671 -1.49669245 0.88703817
C -3.66067830 -0.22440807 0.07777733
N -5.01950475 -0.66353081 -0.24435670
O -5.14321105 -1.70477422 -0.85831149
O -5.94096856 0.03859469 0.12109446
C -4.31443370 2.17184171 -0.20720145
N -5.02019846 3.05151609 -0.46163462
C -2.91586327 -2.59650385 0.39026879
N -3.03314906 -3.74521507 0.45044796
*

TSCD2
N 0.91027439 1.31751917 -1.51745403
O 2.18558156 1.06259519 -1.15120883
C 0.13653747 2.04413424 -0.70720191
C 0.31725629 0.30579388 -2.36385896
H -0.86443997 2.22166848 -1.11261712
C 0.76519668 3.17818961 0.05264321
H 0.96029056 0.14771804 -3.24238221
H 0.22822593 -0.64897629 -1.81330376
H -0.68190332 0.62445507 -2.69555970
H 1.15760008 3.91832266 -0.66527093
H 0.01923443 3.66713952 0.69420751
H 1.60625258 2.83365355 0.66894980
C 0.50396221 0.51536620 1.45330292
C -0.52566458 0.80842401 0.76101097
O 1.56387217 0.37100695 2.01314527
Li 2.66485690 0.01552547 0.30055229
O 2.36517208 -1.88481146 0.02106223
O 4.54622390 0.38357609 0.68648530
C 1.89178228 -2.82899354 0.96140061
H 0.95139894 -3.29232394 0.61145090
H 2.63957889 -3.62540692 1.12988654
H 1.70757811 -2.29956170 1.90554491
C 2.60697991 -2.42989152 -1.26185512
H 1.68614344 -2.87568513 -1.67960443
H 2.93857747 -1.61187592 -1.91563952
H 3.38972716 -3.20903892 -1.21709460
C 5.43762112 -0.14977314 -0.27603553
H 6.48561526 -0.02661653 0.05134761
H 5.22024027 -1.22230623 -0.37640282
H 5.30641033 0.34386448 -1.25583622
C 4.76468161 1.75999410 0.94574568
H 4.62735572 2.36136675 0.03012919
H 4.03302862 2.07703392 1.70197181
H 5.78358190 1.92367016 1.34051826
C -1.84503223 0.22729582 0.53100815
C -2.93626758 1.02734128 0.14492317
C -2.06482126 -1.15936684 0.66850763
C -4.19500250 0.47401358 -0.09441995
H -2.80412269 2.10805617 0.04250821
C -3.31658881 -1.72330834 0.43711488
H -1.22922466 -1.80340410 0.95825492
C -4.37667998 -0.89970076 0.05259391
H -3.47182035 -2.79922648 0.54892101
H -5.03414773 1.10880346 -0.38938704
Cl -5.94590620 -1.59996077 -0.24624625
*

TSCD21
N 1.54973370 1.56566080 -1.35427778
O 2.75148158 1.02196764 -1.06653485
C 0.93894815 2.30536014 -0.42381990
C 0.76302143 0.81158709 -2.30533921
H -0.01366689 2.72204277 -0.76500992
C 1.78002903 3.19701475 0.44670367
H 1.35404223 0.65649628 -3.22006764
H 0.49615594 -0.17551084 -1.88421307
H -0.15807671 1.35823234 -2.55541720
H 2.26389606 3.95968145 -0.18654599
H 1.15292495 3.70262720 1.19367736
H 2.57405957 2.63396586 0.95502793
C 1.09885708 0.45443286 1.50233780
C 0.12421830 1.01019070 0.88289217
O 2.10914193 0.06602835 2.01708059
Li 3.09606979 -0.28811884 0.18295393
O 2.38985844 -2.03984691 -0.24483921
O 5.01278828 -0.33656782 0.52403971
C 1.94896768 -3.03520633 0.65875435
H 0.94085608 -3.39475444 0.38416477
H 2.64384391 -3.89462043 0.66618093
H 1.91635689 -2.58896278 1.66150605

C	2.43717612	-2.47284099	-1.59182760
H	1.43228207	-2.76763010	-1.94414797
H	2.80133901	-1.63329832	-2.19943482
H	3.12035196	-3.33421885	-1.70439634
C	5.75907275	-0.87467681	-0.55355773
H	6.81369375	-1.02476328	-0.26121337
H	5.31957536	-1.84803519	-0.81311696
H	5.72023236	-0.20931654	-1.43475026
C	5.50993648	0.91162095	0.97791318
H	5.47437148	1.66705638	0.17348655
H	4.87579799	1.23949123	1.81343455
H	6.55010588	0.80821453	1.33532756
C	-1.24139730	0.59948738	0.62551176
C	-2.22673554	1.54636973	0.27992313
C	-1.61121552	-0.76345039	0.69460401
C	-3.52477894	1.13992339	0.02419727
H	-1.99936055	2.61189902	0.21843127
C	-2.90882055	-1.14821612	0.42361796
H	-0.88090659	-1.53187369	0.95665939
C	-3.89631937	-0.20968248	0.08286906
N	-5.25864245	-0.62625565	-0.20360685
O	-5.41599201	-1.63040395	-0.87275045
O	-6.16596489	0.05178170	0.24067955
F	-3.23657928	-2.43586863	0.51635357
F	-4.43127499	2.05349123	-0.31621101

*

TSCD6

N	0.86973654	1.36142833	-1.48741245
O	2.15933915	1.11174095	-1.16856201
C	0.11921832	2.07615254	-0.64573230
C	0.25461096	0.35451609	-2.32359101
H	-0.89575496	2.25189015	-1.01549013
C	0.76773997	3.20338687	0.10717747
H	0.86924614	0.20851419	-3.22434038
H	0.18872693	-0.60596820	-1.77978197
H	-0.75684800	0.67048318	-2.61861007
H	1.14278586	3.94971679	-0.61365590
H	0.03870354	3.68690168	0.77192989
H	1.62361553	2.85289350	0.69923768
C	0.54103909	0.53691387	1.49065633
C	-0.50924377	0.82332729	0.83131699
O	1.62440597	0.39684653	2.01233626
Li	2.66964139	0.06224323	0.27497899
O	2.38047539	-1.83836669	-0.02617012
O	4.56050591	0.44618033	0.61332311
C	1.90597859	-2.79201660	0.90370827
H	0.95856206	-3.24158472	0.55464346
H	2.64764956	-3.59771980	1.05458604
H	1.73384086	-2.27469079	1.85680509
C	2.60443553	-2.36526503	-1.31955420
H	1.67738042	-2.80311998	-1.73202895
H	2.92886267	-1.53838464	-1.96570106
H	3.38601421	-3.14662051	-1.29623041
C	5.42352438	-0.03731834	-0.39977035
H	6.48105374	0.10114178	-0.11126465
H	5.22684784	-1.11141358	-0.52366070
H	5.24069875	0.48161936	-1.35793808
C	4.75993895	1.81875482	0.90537023
H	4.57923814	2.44292999	0.01279949
H	4.04863166	2.09806896	1.69509303
H	5.78852557	1.99278414	1.26964610
C	-1.84320747	0.26098279	0.62069594
C	-2.93972715	1.07384542	0.30222827
C	-2.07458058	-1.13010103	0.70799919
C	-4.21672412	0.54249074	0.08015883
H	-2.80425822	2.15755204	0.23556870
C	-3.33499550	-1.67076317	0.49424902
H	-1.23633503	-1.79247193	0.94568401
C	-4.42004691	-0.83712326	0.17515532
H	-3.50125713	-2.74920661	0.56592290
H	-5.03812028	1.21916120	-0.16077860
O	-5.61848625	-1.45175133	-0.02326627
C	-6.74014252	-0.65015371	-0.34625647
H	-6.58559833	-0.10238693	-1.29194481
H	-7.59042534	-1.33532405	-0.46390273
H	-6.96687427	0.06975108	0.45908267

*

TSCD8

N	0.85513168	1.35477351	-1.47430146
O	2.08129111	0.88722701	-1.15294622
C	0.21801336	2.15015739	-0.61130411
C	0.09710839	0.49471039	-2.35622863
H	-0.75108583	2.49762207	-0.98206902
C	1.02610802	3.13970118	0.18029818
H	0.69158643	0.28943160	-3.25897666
H	-0.12897144	-0.46462622	-1.85522436
H	-0.84648130	0.98168988	-2.64331722
H	1.50529069	3.84945594	-0.51526338
H	0.37641350	3.69981842	0.86672022
H	1.82258308	2.64611386	0.75288202
C	0.40548767	0.49748190	1.47613447
C	-0.58629400	0.95272391	0.81492833
O	1.44329931	0.19421786	2.01084273
Li	2.43029928	-0.26873307	0.25387058
O	1.81227500	-2.08174081	-0.06730245
O	4.35644514	-0.21512902	0.58454630
C	1.33480925	-3.00693990	0.88951274
H	0.34204513	-3.39516685	0.59803794
H	2.03228786	-3.85780496	0.99670116
H	1.25275058	-2.48311766	1.85112011
C	1.91985697	-2.61357102	-1.37380450
H	0.93595472	-2.95956657	-1.73905796
H	2.28636895	-1.81218137	-2.02946146
H	2.62577443	-3.46389769	-1.39644743
C	5.10982963	-0.80050138	-0.46243366
H	6.18041094	-0.84793152	-0.19402822
H	4.73563273	-1.82260657	-0.61413043
H	4.99767927	-0.22693110	-1.40006169
C	4.78938631	1.09335042	0.91686462
H	4.70877313	1.76910791	0.04746296
H	4.14377371	1.46242985	1.72599928
H	5.83604825	1.07788832	1.27047381
C	-1.96327549	0.53411582	0.58058375
C	-2.95651220	1.46176107	0.21235191
C	-2.34191395	-0.82122343	0.69740836
C	-4.26782285	1.05845696	-0.02851966
H	-2.69925884	2.52113209	0.12783263
C	-3.64939760	-1.22825805	0.46266694
H	-1.58418258	-1.56071530	0.97320863
C	-4.63280010	-0.29207480	0.09257487
H	-3.91936227	-2.28327445	0.56076154
H	-5.02324271	1.79755226	-0.30882866
C	-5.99113843	-0.71318463	-0.15474249
C	-7.13206035	-1.06896419	-0.35973406
H	-8.14766200	-1.38539502	-0.54176103

*

TSCD9

N	0.84273321	1.37132213	-1.47354004
O	2.06638042	0.90501128	-1.14256855
C	0.19710166	2.16165338	-0.61165026
C	0.09267216	0.51119267	-2.36218044
H	-0.76959065	2.50838626	-0.98931905
C	0.99650130	3.15257194	0.18732799
H	0.69447647	0.30715821	-3.26025938
H	-0.13637638	-0.44884358	-1.86357821
H	-0.84891644	0.99767937	-2.65660658
H	1.47177386	3.86935938	-0.50362169
H	0.34143722	3.70450023	0.87525543
H	1.79540326	2.66138426	0.75857350

C 0.38767683 0.49152481 1.46488619
C -0.60156572 0.95548609 0.80223694
O 1.41771102 0.18314704 2.00581499
Li 2.42535720 -0.26779048 0.24327969
O 1.80708325 -2.07828226 -0.08100544
O 4.34483700 -0.20901969 0.59170246
C 1.34521389 -3.01214281 0.87534147
H 0.35448578 -3.40905495 0.58875313
H 2.05188041 -3.85631348 0.97438901
H 1.26455022 -2.49327465 1.83973176
C 1.91536954 -2.60549593 -1.38960737
H 0.93313964 -2.95767814 -1.75331920
H 2.27460786 -1.79972989 -2.04394676
H 2.62759334 -3.45033297 -1.41639134
C 5.11499699 -0.80144773 -0.43916217
H 6.17938419 -0.85682085 -0.14871928
H 4.73592794 -1.82076868 -0.59758118
H 5.02699851 -0.22813026 -1.37955262
C 4.77637181 1.09920474 0.92747625
H 4.70928117 1.77346513 0.05576224
H 4.12053050 1.47153915 1.72685514
H 5.81787470 1.08182262 1.29569905
C -1.97182341 0.52367145 0.57144891
C -2.97019700 1.44165515 0.18714213
C -2.33869417 -0.83475364 0.70840315
C -4.27640412 1.02763627 -0.05275048
H -2.72011031 2.50112450 0.08995146
C -3.63986711 -1.25694605 0.47573063
H -1.57566230 -1.56323342 0.99703379
C -4.61962818 -0.32551395 0.08862272
H -3.90502624 -2.31142061 0.58704660
H -5.03847511 1.75418274 -0.34592858
C -5.97137052 -0.75955939 -0.16060012
N -7.05772789 -1.10969254 -0.36083748
*

TSEF0
Au 0.10974070 -1.44924912 0.06995438
C 1.30523643 0.91804476 0.64973829
C 0.12022197 0.52079202 0.82727128
C 0.45790355 3.17459700 1.32763448
C 1.74789665 2.87233822 1.61500609
H 1.99696156 2.37052497 2.54300183
C -1.07021803 1.21990088 1.36816678
H -1.64431303 0.58327185 2.04196954
H -1.72032023 1.47742239 0.52766036
C -0.61433987 2.51066051 2.11762292
P -0.03737829 -3.59618415 -0.71668578
C 1.57072396 -4.37107943 -1.00815239
C -0.90635891 -4.70827894 0.41413275
H 2.30184710 0.83679323 0.25619635
C -0.92213033 -3.72485901 -2.28867483
H -0.40919919 -3.12786349 -3.04481976
H -0.95982583 -4.76825501 -2.61042791
H -1.93766176 -3.34393671 -2.16619649
H 2.12589039 -3.78946203 -1.74625579
H 1.43098699 -5.39020959 -1.37649246
H 2.13844478 -4.39463511 -0.07630406
H -0.37860113 -4.74064665 1.36897592
H -0.95104548 -5.71294717 -0.01277614
H -1.91930134 -4.33807275 0.58215134
H 2.56854392 3.28671093 1.04024261
H -1.49087010 3.14446475 2.24946836
H -0.23162444 2.22721965 3.09820367
C 0.08612370 3.98154528 0.13874234
C -1.05069225 4.79240387 0.15895398
C 0.86750850 3.94878792 -1.02053798
C -1.38777174 5.56509490 -0.94554658
H -1.66758371 4.84379473 1.04820932
C 0.52372940 4.71155465 -2.12669315
H 1.73892316 3.30553940 -1.06593700
C -0.60400843 5.52578568 -2.09173609
H -2.26717138 6.19882766 -0.90815419
H 1.13511767 4.66666457 -3.02149870
H -0.87154011 6.12374877 -2.95621269
*

TSEF1
Au 0.00544033 -1.67646189 0.21353575
C 1.14997038 0.59006782 1.14956408
C -0.06141235 0.24304165 1.09028678
C 0.27045352 2.85610074 1.77185070
C 1.47505900 2.49580278 2.27889512
H 1.53153756 1.94800505 3.21251157
C -1.30574495 0.96750218 1.44498159
H -2.01319166 0.32595219 1.97119264
H -1.78415650 1.28953753 0.51610636
C -0.94660459 2.20210890 2.32794806
P -0.06988690 -3.75821695 -0.73974544
C 0.92791167 -3.90031086 -2.24154571
C 0.51549551 -5.07595761 0.35174788
H 2.19947923 0.48634875 0.94416869
C -1.74065278 -4.24836758 -1.22836165
H -2.13379943 -3.53024690 -1.95018561
H -1.71973422 -5.24381718 -1.67808291
H -2.38875302 -4.25850607 -0.35021892
H 0.57768228 -3.17719770 -2.98029475
H 0.84330081 -4.91012019 -2.65004539
H 1.97234046 -3.68826209 -2.00595348
H 1.55259902 -4.88285876 0.63177755
H 0.44861637 -6.03939037 -0.15918776
H -0.09652072 -5.09849757 1.25519863
H 2.40167139 2.89768657 1.88490724
H -1.81091392 2.86577305 2.34152951
H -0.75100131 1.85923859 3.34419781
C 0.14505873 3.70815580 0.56490565
C -0.97668111 4.51271384 0.36719832
C 1.14851392 3.72417538 -0.41139908
C -1.08263420 5.32205361 -0.75829190
H -1.77180652 4.53250451 1.10304464
C 1.03235677 4.52286503 -1.53522078
H 2.01821993 3.08610250 -0.30263514
C -0.08445146 5.34100926 -1.72728944
H -1.96015024 5.94863261 -0.88253964
H 1.81886062 4.50962836 -2.28382893
C -0.19365589 6.21130880 -2.95071533
H -1.14224059 6.75082984 -2.96707041
H 0.61806968 6.94385494 -2.97775802
H -0.12233066 5.61154233 -3.86222077
*

TSEF10
Au -0.01751893 -1.58653659 0.22680800
C 1.23573076 0.74998710 0.79051301
C 0.06905129 0.35075100 1.05994703
C 0.46324579 2.98467317 1.62293919
C 1.77265566 2.67093105 1.78033941
H 2.10335323 2.13511442 2.66275441
C -1.06452679 1.03267960 1.72953475
H -1.58117198 0.37325715 2.42746218
H -1.78416106 1.32326273 0.95941433
C -0.53613726 2.29374178 2.48204559
P -0.24535201 -3.69670929 -0.63571778
C 0.27144660 -3.83017877 -2.36377302
C 0.71563549 -4.94696172 0.24977616
H 2.19257885 0.68095676 0.30654643
C -1.95250603 -4.29236649 -0.60028340
H -2.58491992 -3.61918195 -1.18177066
H -2.00425970 -5.29836767 -1.02311641
H -2.31012165 -4.31126953 0.43074906
H -0.32326405 -3.14876801 -2.97471216
H 0.13029628 -4.85460867 -2.71624586

H	1.32434169	-3.55614854	-2.45085490
H	1.77543400	-4.68976438	0.20554416
H	0.55855058	-5.92799976	-0.20474201
H	0.40149267	-4.97427210	1.29468382
H	2.53891920	3.10518546	1.14809510
H	-1.39377566	2.92461594	2.71420618
H	-0.06874933	1.97351445	3.41348588
C	-0.01304998	3.83394296	0.50282943
C	-1.12961935	4.65372492	0.66060633
C	0.65408011	3.82771962	-0.72746861
C	-1.55207001	5.46194302	-0.38936250
H	-1.65944530	4.68600363	1.60512296
C	0.23298044	4.62084107	-1.78712263
H	1.50712585	3.17286731	-0.86966479
C	-0.88041484	5.44460645	-1.60203572
H	-2.41365127	6.10751967	-0.25643663
H	-1.22217898	6.07243971	-2.41945195
C	0.95245498	4.59549418	-3.11083527
H	0.27079402	4.31671845	-3.91900778
H	1.35711853	5.58271492	-3.35072703
H	1.77827108	3.88181116	-3.09698400

*

TSEF11

Au	-0.04065978	-1.59067634	0.22649639
C	1.20779660	0.74235934	0.84191728
C	0.02895133	0.34560122	1.06133298
C	0.41744798	2.97645130	1.62855265
C	1.71628020	2.65267098	1.84368907
H	2.00445678	2.12199007	2.74391862
C	-1.12773982	1.03685256	1.68010669
H	-1.67537350	0.38433856	2.36070438
H	-1.81435163	1.32559611	0.87975903
C	-0.62534334	2.30088595	2.44621899
P	-0.25066404	-3.69738808	-0.64907931
C	0.24768843	-3.81067524	-2.38399324
C	0.73636129	-4.94258299	0.21486335
H	2.18219176	0.66448167	0.39536505
C	-1.94956958	-4.31529679	-0.59900051
H	-2.59759881	-3.64439393	-1.16575725
H	-1.99378061	-5.31752753	-1.03153207
H	-2.29426817	-4.34919166	0.43605397
H	-0.36280691	-3.13235048	-2.98268808
H	0.11577652	-4.83406450	-2.74300071
H	1.29584395	-3.52215662	-2.48103798
H	1.79241617	-4.67260497	0.15841627
H	0.58476382	-5.92218629	-0.24459183
H	0.43656859	-4.98096608	1.26362256
H	2.51207580	3.07684925	1.24175052
H	-1.48767556	2.94028055	2.63272159
H	-0.20341391	1.98512986	3.40052052
C	-0.00087591	3.82602759	0.48449567
C	-1.10776821	4.67042969	0.59909414
C	0.71567035	3.79302221	-0.71170906
C	-1.47199509	5.47982442	-0.46933168
H	-1.66998022	4.71927181	1.52317365
C	0.33388037	4.59694440	-1.77822838
H	1.56254881	3.12862807	-0.83710629
C	-0.76211637	5.44886296	-1.66122101
H	-2.32340763	6.14486309	-0.37382536
H	-1.05425255	6.07566335	-2.49832121
O	1.06714412	4.50641940	-2.92410309
H	0.70525001	5.11491735	-3.58353386

*

TSEF12

Au	-0.13843267	-2.10574470	0.12934486
C	0.87879785	-0.06071780	1.57071497
C	-0.31079657	-0.31831808	1.24053790
C	-0.02042533	2.15919772	2.32810615
C	1.04839685	1.66132526	2.99700474
H	0.90244725	0.99433500	3.83886108
C	-1.57279657	0.43006827	1.45485260
H	-2.39093472	-0.22889124	1.74718811
H	-1.85185736	0.89771509	0.50675977
C	-1.34574020	1.51672089	2.55077156
P	-0.08768806	-4.03203227	-1.10950774
C	0.90621555	-3.88963834	-2.61382689
C	0.59550660	-5.44966850	-0.21796153
H	1.94384672	-0.20073274	1.55214070
C	-1.72551866	-4.55969733	-1.66576277
H	-2.17217852	-3.77411128	-2.27784415
H	-1.64095587	-5.47682099	-2.25337800
H	-2.36402648	-4.73924311	-0.79897735
H	0.50120742	-3.09524696	-3.24328351
H	0.89005462	-4.83434871	-3.16238840
H	1.93485295	-3.64027132	-2.34737989
H	1.62060291	-5.22901567	0.08501117
H	0.58678373	-6.33304287	-0.86082551
H	-0.00336744	-5.64171336	0.67406470
H	2.04814075	2.05112630	2.84260147
H	-2.17161442	2.22552017	2.49339835
H	-1.36389206	1.03494567	3.52857543
C	0.12307512	3.16377867	1.24785358
C	-0.91421827	4.04974581	0.94530374
C	1.29795462	3.24960603	0.49642987
C	-0.77557762	4.99734978	-0.05759938
H	-1.83788016	4.02074324	1.51098083
C	1.42950194	4.18956884	-0.51295008
H	2.11197924	2.55858459	0.68395586
C	0.39814000	5.08371240	-0.80798335
H	-1.59925936	5.67578010	-0.25256525
H	2.34796178	4.22839309	-1.09018972
C	0.57862518	6.07853996	-1.89885213
C	-0.58054354	6.47153482	-2.78274234
C	-0.03315455	7.45465546	-1.79675307
H	1.54652270	6.02562058	-2.38363738
H	-1.54050636	6.01722117	-2.56573314
H	-0.37162290	6.62866631	-3.83422081
H	0.55170659	8.28604199	-2.17176584
H	-0.62259530	7.66720120	-0.91236655

*

TSEF13

Au	-0.01130922	-1.75244301	0.20801904
C	1.27869023	0.56255042	0.83510057
C	0.13981891	0.12235980	1.16219144
C	0.57285337	2.70536488	1.88465569
C	1.89126923	2.38829133	1.91204231
H	2.29224362	1.79758031	2.72749522
C	-0.93725660	0.74123206	1.97218910
H	-1.39093208	0.02796217	2.66086121
H	-1.72062695	1.07958088	1.28856552
C	-0.35349682	1.94982808	2.76951439
P	-0.29419543	-3.80306458	-0.77481653
C	0.26536339	-3.86307286	-2.49368037
C	0.59650248	-5.13361612	0.06592800
H	2.18879062	0.53575940	0.26394736
C	-2.02192293	-4.33593417	-0.81567501
H	-2.61423080	-3.60861618	-1.37373117
H	-2.09812436	-5.31422677	-1.29592625
H	-2.40744716	-4.39892178	0.20351263
H	-0.28860208	-3.12966043	-3.08232801
H	0.09926572	-4.86177537	-2.90437982
H	1.32900391	-3.62246796	-2.53871802
H	1.66595124	-4.91565330	0.06018725
H	0.41455661	-6.08325931	-0.44274546
H	0.25513094	-5.20265475	1.10034398
H	2.60404936	2.87111292	1.25300179
H	-1.19016681	2.55529262	3.11740698
H	0.18988561	1.56759357	3.63366778
C	0.01050887	3.62813127	0.86680966

C	-1.10590009	4.41415357	1.15554886
C	0.58464301	3.73082756	-0.40224746
C	-1.62760853	5.29412530	0.21081588
H	-1.56780106	4.36119994	2.13410331
C	0.05759746	4.59887600	-1.34907021
H	1.43747256	3.11600023	-0.67111168
C	-1.05182175	5.38876768	-1.04383940
H	-2.48844936	5.90249563	0.46400722
H	-1.45023156	6.06221078	-1.79405016
C	0.69230857	4.65995897	-2.68797553
O	0.31969517	5.38011441	-3.58765567
H	1.55832579	3.98571863	-2.82544833

*

TSEF14

Au	0.01325105	-2.11937632	0.12762093
C	1.19667631	0.07435103	1.27036919
C	-0.01087881	-0.29610891	1.18308152
C	0.33392342	2.20282167	2.18672785
C	1.54858517	1.76095363	2.60087415
H	1.62393592	1.10425155	3.45982252
C	-1.24978505	0.37533541	1.64486670
H	-1.94882329	-0.32487167	2.10287816
H	-1.74468590	0.80657888	0.77048795
C	-0.88188631	1.49769574	2.66782581
P	-0.09475940	-4.11144648	-1.00025699
C	1.10908640	-4.25638816	-2.34219841
C	0.19444832	-5.55018820	0.05708615
H	2.23593232	-0.00786812	1.00721238
C	-1.70604186	-4.39884203	-1.76976387
H	-1.91561567	-3.60064595	-2.48410486
H	-1.70619253	-5.36100985	-2.28726707
H	-2.48113775	-4.39822072	-1.00118275
H	0.94620490	-3.45622488	-3.06638635
H	0.99907212	-5.22450527	-2.83662270
H	2.11847495	-4.16683489	-1.93677210
H	1.18790896	-5.47941409	0.50359034
H	0.12399663	-6.46674498	-0.53332856
H	-0.55090163	-5.57145483	0.85428624
H	2.46650764	2.21000261	2.23821791
H	-1.74245098	2.16003512	2.75723077
H	-0.68439762	1.03556842	3.63495012
C	0.20025268	3.23576688	1.12830409
C	-0.85632223	4.14786934	1.17076632
C	1.12630346	3.31246252	0.08366658
C	-0.98523996	5.12713774	0.19853460
H	-1.57670624	4.11478299	1.97826207
C	1.00393693	4.27766226	-0.90055201
H	1.93980418	2.59996413	0.02345094
C	-0.05290501	5.17059941	-0.82319611
H	-1.79589643	5.84206924	0.23237074
H	1.71141692	4.33396413	-1.71649336
N	-0.19293542	6.19873205	-1.87027465
O	-1.11901352	6.98253705	-1.77904790
O	0.62461168	6.20932888	-2.77145126

*

TSEF15

Au	-0.05207771	-2.03842258	0.21055229
C	1.28148311	0.19013607	1.06657861
C	0.07254254	-0.16835276	1.17493356
C	0.56079880	2.38919319	1.93376201
C	1.82008941	1.97064387	2.21683179
H	2.00716829	1.37928834	3.10540822
C	-1.08696754	0.54584470	1.76231072
H	-1.71063102	-0.11419352	2.36575475
H	-1.70434824	0.91807264	0.94027654
C	-0.57427355	1.73482249	2.63585276
P	-0.31162085	-4.06865246	-0.82057569
C	0.62513886	-4.21839038	-2.36039712
C	0.21162977	-5.46891535	0.19741977
H	2.27453836	0.08169365	0.66896510
C	-2.02794447	-4.41972746	-1.26972843
H	-2.39225618	-3.64562216	-1.94744820
H	-2.09245157	-5.39385751	-1.76007745
H	-2.64646326	-4.42245758	-0.37042533
H	0.30220117	-3.44421724	-3.05878200
H	0.45798653	-5.20290323	-2.80371741
H	1.68879274	-4.08718909	-2.15371148
H	1.26889871	-5.36268682	0.44688545
H	0.05723534	-6.40336616	-0.34722507
H	-0.36988056	-5.48449114	1.12094502
H	2.68337337	2.37611639	1.70149704
H	-1.41165505	2.41086224	2.80538526
H	-0.23686246	1.34304545	3.59516244
C	0.28012915	3.32278879	0.81357794
C	-0.82317235	4.17457147	0.87217218
C	1.11783162	3.35587731	-0.30405643
C	-1.06159375	5.05074665	-0.16729562
H	-1.49340146	4.19220509	1.72167754
C	0.84653890	4.23364168	-1.33012395
H	1.96768736	2.69251783	-0.40259955
C	-0.23912373	5.09416564	-1.27920602
F	-2.11104616	5.88735574	-0.11973208
F	-0.48772154	5.94016889	-2.28328150
F	1.63131103	4.26760254	-2.41943763

*

TSEF16

Au	-0.06256493	-1.83509652	0.21079106
C	1.17798103	0.40182075	1.07962660
C	-0.02759014	0.04557678	1.17193679
C	0.36354257	2.63470036	1.93288388
C	1.62915454	2.26705659	2.25183711
H	1.81481892	1.67076714	3.13759963
C	-1.21871598	0.73055994	1.72842630
H	-1.83952834	0.05163967	2.31377789
H	-1.82388020	1.08829335	0.89106306
C	-0.75649372	1.92726415	2.61523454
P	-0.25572912	-3.87862534	-0.80639270
C	0.35468558	-3.89614684	-2.50865996
C	0.64496888	-5.19824166	0.04068746
H	2.19141774	0.32543691	0.73139518
C	-1.96714616	-4.45483138	-0.90528205
H	-2.56176881	-3.73473699	-1.47019107
H	-2.00389350	-5.42758958	-1.40119801
H	-2.38007082	-4.54269767	0.10126660
H	-0.20131840	-3.16764535	-3.10146180
H	0.22702023	-4.89190093	-2.93981247
H	1.41242363	-3.62720537	-2.51862902
H	1.70769113	-4.95155274	0.07205269
H	0.50484720	-6.14357804	-0.48882559
H	0.27354607	-5.29392263	1.06255110
H	2.49228321	2.70338974	1.76221346
H	-1.62101881	2.56837219	2.78398481
H	-0.41656795	1.53859397	3.57534212
C	0.06118320	3.54733805	0.80639241
C	-1.11630705	4.29222626	0.78178579
C	0.96070179	3.68146162	-0.26126028
C	-1.38789334	5.16384734	-0.26704778
H	-1.82845452	4.22331956	1.59409609
C	0.68828606	4.53961562	-1.30649545
H	1.87166442	3.09304803	-0.29432199
C	-0.49376936	5.29311033	-1.31494349
H	-2.29843150	5.75241878	-0.27818347
O	-0.76990279	6.13846786	-2.34141210
H	-0.03346614	6.11562166	-2.96986105
O	1.50655511	4.71573557	-2.38865622
H	2.31550670	4.19743442	-2.27802104

*

TSEF17

Au	0.04996608	-2.34223454	0.17187213
C	1.50180728	-0.24853789	1.05670748
C	0.27007253	-0.49216641	1.16759097
C	0.91799105	2.03175064	1.96980868
C	2.15192983	1.54437486	2.24902429
H	2.30442839	0.91245163	3.11635372
C	-0.83984440	0.29076420	1.76223614
H	-1.51348835	-0.33843268	2.34470510
H	-1.42165989	0.72486066	0.94460709
C	-0.24717727	1.41580967	2.66524742
P	-0.33206448	-4.34108699	-0.87973787
C	0.22391947	-4.36813325	-2.60048900
C	0.48986852	-5.74384614	-0.08782317
H	2.49661973	-0.41097665	0.68489521
C	-2.08493158	-4.78269501	-0.93645133
H	-2.63840574	-4.00784609	-1.46985296
H	-2.21099425	-5.73942725	-1.44862284
H	-2.47387516	-4.85929869	0.08048235
H	-0.29412939	-3.58869265	-3.16206573
H	0.00970046	-5.34270302	-3.04548286
H	1.29790174	-4.17761286	-2.63907835
H	1.56881400	-5.57905346	-0.08523328
H	0.26226269	-6.66391709	-0.63132858
H	0.14311234	-5.83373252	0.94317896
H	3.03760162	1.91312636	1.74422472
H	-1.04271516	2.13182148	2.87036776
H	0.07574748	0.97259521	3.60746268
C	0.67638580	2.98838198	0.86496834
C	1.51370719	3.03317730	-0.24052996
C	-0.42095065	3.86482151	0.91450963
C	1.27826605	3.93275035	-1.27823790
H	2.34863509	2.34786879	-0.32413451
C	-0.65858390	4.77022765	-0.10332439
H	-1.07794424	3.85146576	1.77200282
C	0.20393010	4.80765038	-1.22288368
H	1.94065390	3.93652102	-2.13362398
O	-0.10394482	5.71728310	-2.17620753
O	-1.68558276	5.65752310	-0.12168262
C	0.74883000	5.78123660	-3.31767099
H	0.33891558	6.56678145	-3.95104046
H	1.77205158	6.04113061	-3.03086327
H	0.74634867	4.83382612	-3.86462439
C	-2.57694712	5.64617949	0.98970810
H	-3.07738917	4.67753671	1.08542931
H	-2.05229643	5.88462421	1.92003705
H	-3.31833437	6.41756700	0.78491730

*

TSEF18

Au	-0.10021442	-2.10409805	0.12570348
C	1.09082343	-0.09625334	1.42753518
C	-0.13529634	-0.34293340	1.29757184
C	0.28059211	2.12517444	2.39451589
C	1.45101991	1.64010556	2.87788771
H	1.44830118	0.94173884	3.70668438
C	-1.35787115	0.37045586	1.73735218
H	-2.10274334	-0.31737454	2.13888765
H	-1.79786781	0.85677682	0.86260829
C	-0.97169298	1.42852177	2.81395078
P	-0.22737123	-4.00930496	-1.13916629
C	0.61241058	-3.87778131	-2.73545520
C	0.49318522	-5.45981280	-0.33498663
H	2.14393434	-0.20691970	1.24851025
C	-1.93025301	-4.47451133	-1.53237251
H	-2.40767788	-3.66716531	-2.09055164
H	-1.93772097	-5.38737285	-2.13256667
H	-2.48511685	-4.64154439	-0.60745004
H	0.17442693	-3.06003463	-3.31046359
H	0.50460448	-4.81314133	-3.28975573
H	1.67153957	-3.66952730	-2.57374808
H	1.54976846	-5.27602256	-0.13218780
H	0.39215341	-6.33345121	-0.98323206
H	-0.02160838	-5.64451610	0.60955790
H	2.41000522	2.05428913	2.58905506
H	-1.81516336	2.10784047	2.93431129
H	-0.80505533	0.91499372	3.76119398
C	0.21402602	3.15239023	1.33688934
C	-0.95352226	3.88141787	1.09381305
C	1.31228964	3.42861049	0.51243183
C	-1.02281966	4.85037740	0.10827707
H	-1.83938104	3.70870845	1.69325012
C	1.26078823	4.38379014	-0.48260947
H	2.22967366	2.86213612	0.62738938
C	0.09102951	5.14347528	-0.70292055
H	-1.95382054	5.38365360	-0.02567822
H	2.13649894	4.53562221	-1.09849085
N	0.04343525	6.12626358	-1.66242978
C	1.10818500	6.20063608	-2.65018493
C	-1.24935528	6.66933057	-2.04771614
H	0.91880410	7.05054633	-3.30407392
H	2.07563999	6.36161313	-2.17014066
H	1.17065700	5.29355136	-3.26601986
H	-1.09448154	7.44160922	-2.79961790
H	-1.91509972	5.90278033	-2.46609607
H	-1.74809703	7.13322424	-1.19405989

*

TSEF19

Au	-0.06776288	-2.61357975	0.13773977
C	1.21847109	-0.48827569	1.35594091
C	0.00475157	-0.86135945	1.29861923
C	0.44994451	1.56864135	2.38622758
C	1.67039505	1.07310144	2.72195247
H	1.76460004	0.39381927	3.56094789
C	-1.19903587	-0.19613646	1.85304172
H	-1.88117766	-0.90398648	2.32451242
H	-1.73627629	0.27199635	1.02381424
C	-0.76558885	0.88878075	2.89455078
P	-0.25792866	-4.52244544	-1.11809436
C	0.06923684	-4.26365782	-2.87805974
C	0.87116109	-5.84092159	-0.61003828
H	2.23441028	-0.57992366	1.01305106
C	-1.91052015	-5.25341170	-1.04149062
H	-2.64407953	-4.53320540	-1.40827983
H	-1.94785213	-6.15674254	-1.65485259
H	-2.14898510	-5.50539788	-0.00658017
H	-0.63154088	-3.52633112	-3.27374639
H	-0.04612693	-5.20489177	-3.42073494
H	1.08654347	-3.89018421	-3.00829500
H	1.90175275	-5.49370739	-0.70307784
H	0.72402313	-6.72041314	-1.24136831
H	0.67870276	-6.10344458	0.43175051
H	2.58341227	1.51143750	2.33412194
H	-1.60297097	1.57213964	3.03345069
H	-0.54365257	0.39598656	3.84050818
C	0.30727992	2.64445236	1.37112766
C	-0.68883945	3.60586426	1.50702091
C	1.16339284	2.70872765	0.27490168
C	-0.81667994	4.63526377	0.57736328
H	-1.36998441	3.58535940	2.34781897
C	1.02207100	3.70870835	-0.68176101
H	1.94133295	1.96947005	0.13498881
C	0.03409219	4.67375805	-0.51983179
N	-0.11740999	5.73905583	-1.52441610
O	-0.02955695	5.41452516	-2.68963597
O	-0.31908329	6.86157278	-1.11308190
C	-1.86894350	5.59898801	0.77628901
N	-2.73616557	6.32205470	0.98322167
C	1.93593384	3.71138955	-1.79537001
N	2.70523207	3.66393832	-2.64609566

*

TSEF2

Au	-0.13854133	-1.90523774	0.15710636
C	0.93001210	0.24452038	1.43016306
C	-0.26796339	-0.05595566	1.16419954
C	0.04296405	2.47182733	2.11150607
C	1.14689211	2.00923575	2.74908306
H	1.04531359	1.38850600	3.63173113
C	-1.52781799	0.69134185	1.39553156
H	-2.32622642	0.04315799	1.75781943
H	-1.85247444	1.10696421	0.43776776
C	-1.26638985	1.83742639	2.42260885
P	-0.13172984	-3.90833358	-0.95515873
C	0.52332095	-3.79605515	-2.63728197
C	0.85646430	-5.18526247	-0.13998611
H	1.99388285	0.10939663	1.35919909
C	-1.78410063	-4.62188305	-1.13175653
H	-2.42215935	-3.92943142	-1.68373400
H	-1.72473163	-5.57110845	-1.66944244
H	-2.21558240	-4.78929588	-0.14323158
H	-0.07823109	-3.09184130	-3.21476457
H	0.49429104	-4.77847375	-3.11453482
H	1.55347916	-3.43697605	-2.60369169
H	1.89341100	-4.85214043	-0.06855818
H	0.81022123	-6.11426613	-0.71316839
H	0.46999425	-5.35750883	0.86613617
H	2.13467716	2.39755474	2.52794494
H	-2.09893949	2.53780399	2.35949840
H	-1.24085344	1.40902898	3.42458293
C	0.13310469	3.43146856	0.98347892
C	-0.90763444	4.32447350	0.72285764
C	1.26048902	3.45886707	0.15730781
C	-0.82374603	5.23426704	-0.32211888
H	-1.79084717	4.33517262	1.34942403
C	1.35280261	4.35352547	-0.89621978
H	2.07075863	2.75829686	0.32049736
C	0.30664267	5.23627562	-1.12206741
H	-1.63015661	5.93223211	-0.51084468
H	2.22469363	4.36148734	-1.53884800
Cl	0.41549104	6.36680678	-2.44509681

*

TSEF20

Au	-0.13666774	-3.27873258	-0.02821514
C	-1.72377230	-1.16927320	0.76634070
C	-1.30325379	-1.56012970	-0.36588819
C	-2.51951038	0.85511006	-0.34155573
C	-3.19473253	0.38623853	0.74033135
H	-4.00814668	-0.31701114	0.60611473
C	-1.48711889	-0.93795662	-1.69944941
H	-1.71675899	-1.67513694	-2.46920467
H	-0.54606334	-0.45645253	-1.97884152
C	-2.63481079	0.12224078	-1.62660818
P	1.16046746	-5.15095957	0.23113806
C	2.41350832	-4.98514462	1.52504687
C	0.21415068	-6.62898947	0.66792278
H	-1.70676223	-1.22676383	1.84046262
C	2.07531904	-5.59373935	-1.26487052
H	2.73145834	-4.76868149	-1.54789875
H	2.67335992	-6.48986870	-1.08356221
H	1.37318434	-5.78261558	-2.07889208
H	3.07217556	-4.14758734	1.28827161
H	3.00135532	-5.90347439	1.59398807
H	1.92536459	-4.79181831	2.48197761
H	-0.30992292	-6.45982124	1.61031225
H	0.88581192	-7.48435171	0.77225188
H	-0.52004288	-6.83482581	-0.11304650
H	-3.10506456	0.85905873	1.71197466
H	-2.54265985	0.77540578	-2.49380095
H	-3.59212024	-0.39619121	-1.67066640
C	-1.52329772	1.95035339	-0.21772354
C	-0.77259334	2.10119048	0.94552614
C	-1.32604534	2.84372707	-1.26540516
C	0.15699056	3.12066697	1.06857722
H	-0.89279619	1.40400842	1.76326395
C	-0.40627597	3.87723465	-1.15850718
H	-1.90769013	2.75750285	-2.17283922
C	0.33162215	4.00346640	0.00964991
C	0.95172526	3.22752063	2.35589972
C	-0.24177635	4.82430889	-2.33178790
N	1.32814032	5.09230228	0.12857552
F	0.97337607	2.05784623	3.01461558
F	0.42779588	4.13992450	3.19227588
F	2.23234305	3.57542000	2.13040113
F	-1.33393810	4.82707879	-3.11172412
F	0.79798035	4.47756171	-3.11062637
F	-0.03972216	6.09350007	-1.93229925
O	1.09601686	5.96348872	0.93740525
O	2.30008469	5.02691860	-0.59151572

*

TSEF21

Au	-0.46329515	-2.34349510	0.10910390
C	0.62987446	-0.25671820	1.53917246
C	-0.55842780	-0.54669563	1.20130454
C	-0.22298537	1.90337876	2.29902689
C	0.84362483	1.35751453	2.93918908
H	0.68744958	0.70437301	3.78962641
C	-1.81071590	0.21547668	1.42695949
H	-2.63525311	-0.42923698	1.73228611
H	-2.09471854	0.68296743	0.48026377
C	-1.56555551	1.30943527	2.51687430
P	-0.46832509	-4.29244551	-1.09813612
C	-1.13067932	-4.09794456	-2.76983535
C	1.17755176	-5.01032573	-1.31694572
H	1.69067952	-0.42030066	1.46767393
C	-1.46258430	-5.59935947	-0.33980037
H	-2.49596356	-5.26104499	-0.24463542
H	-1.42892768	-6.49979589	-0.95763109
H	-1.07121480	-5.82404959	0.65403255
H	-2.15933313	-3.73719172	-2.71417614
H	-1.10793441	-5.05673620	-3.29313277
H	-0.52950855	-3.36953496	-3.31692424
H	1.81894826	-4.29737868	-1.83812006
H	1.10733033	-5.93191102	-1.89963631
H	1.61209896	-5.22883809	-0.33979823
H	1.85019054	1.72561372	2.77519550
H	-2.36768849	2.04295710	2.44125984
H	-1.60439982	0.83761525	3.49831769
C	-0.04718238	2.94348356	1.25176503
C	-1.01420483	3.93501591	1.09291737
C	1.08404980	2.93416489	0.43414693
C	-0.82806551	4.91274118	0.13801836
H	-1.90213157	3.97942748	1.70942821
C	1.22826694	3.90577210	-0.53041231
H	1.84876539	2.17326224	0.52003432
C	0.28366080	4.91153357	-0.69141872
F	-1.75631938	5.86186952	-0.01549399
F	2.31298402	3.89881590	-1.31178705
N	0.45468625	5.94239150	-1.71693362
O	0.67846111	5.56712639	-2.84950245
O	0.36042762	7.09948899	-1.36308473

*

TSEF22

Au	-0.11538415	-2.62136218	0.05014085
C	1.07704101	-0.75559836	1.52910839
C	-0.15113256	-0.97550846	1.38159069
C	0.27424539	1.38351034	2.70148712
C	1.44281410	0.85141744	3.13828599
H	1.43636749	0.07685535	3.89635488
C	-1.37033374	-0.30045255	1.88576737

H	-2.11752408	-1.01994404	2.22231167
H	-1.80865461	0.26621933	1.05996702
C	-0.97941748	0.65198503	3.05514169
P	-0.24409305	-4.39867855	-1.38846600
C	0.56344230	-4.09917887	-2.97856115
C	0.51147857	-5.90887626	-0.74176633
H	2.13017740	-0.85491366	1.34454654
C	-1.94771846	-4.84975602	-1.79420155
H	-2.44781526	-3.99884811	-2.26017582
H	-1.95389013	-5.69843436	-2.48217748
H	-2.48123425	-5.11701399	-0.88029214
H	0.10389616	-3.23548140	-3.46233530
H	0.45828227	-4.97670738	-3.62084445
H	1.62245784	-3.89142451	-2.81537092
H	1.56850807	-5.72849884	-0.53823599
H	0.41243034	-6.71636912	-1.47107579
H	0.01545460	-6.19360810	0.18788431
H	2.40435427	1.28629508	2.89136522
H	-1.82125700	1.31877687	3.23988526
H	-0.81308806	0.05210263	3.95024275
C	0.20740734	2.50484653	1.74653368
C	-0.96750834	3.24163092	1.55897534
C	1.31637355	2.87557306	0.97176000
C	-1.03666085	4.30309925	0.67533661
H	-1.85600901	3.00372626	2.13178635
C	1.26836752	3.92243211	0.07561579
H	2.24000515	2.31295469	1.04960286
C	0.08511081	4.67770956	-0.09307005
H	-1.96421634	4.85359947	0.58472435
H	2.14602619	4.15292435	-0.51476877
N	0.03059770	5.71459624	-0.96624620
C	-1.17813399	6.49432467	-1.21995871
C	1.19558629	6.24006731	-1.67592709
H	-2.02476273	5.83975941	-1.44221129
H	-1.43966295	7.10382248	-0.34539755
C	-0.78893715	7.36674342	-2.41190839
H	2.04923957	6.34254441	-1.00106150
H	1.48753006	5.57010463	-2.49415115
C	0.71025496	7.58979280	-2.20417019
H	-0.96027126	6.81793877	-3.34225519
H	-1.36152447	8.29438856	-2.44823654
H	0.87127038	8.36156958	-1.44627803
H	1.23191546	7.88768967	-3.11459371

*

TSEF23

Au	-0.03367014	-2.45329406	0.13747095
C	1.20753243	-0.36770933	1.25627011
C	-0.02017477	-0.63522963	1.22217212
C	0.44683851	1.90510265	2.17793251
C	1.62138849	1.38666401	2.61607357
H	1.63403201	0.71613502	3.46748424
C	-1.22548139	0.07367696	1.70432415
H	-1.89699739	-0.60213789	2.23523920
H	-1.76888476	0.44518779	0.83291584
C	-0.80826227	1.24764236	2.65066061
P	-0.20851267	-4.41639850	-1.02948259
C	0.62551707	-4.37602524	-2.63368702
C	0.49031652	-5.83969506	-0.16013803
H	2.24634051	-0.47901947	1.00688177
C	-1.92184790	-4.86694712	-1.39224135
H	-2.38694976	-4.07777507	-1.98569840
H	-1.94934843	-5.80660041	-1.94888561
H	-2.47503820	-4.97993610	-0.45818512
H	0.20042884	-3.57781548	-3.24473871
H	0.49734154	-5.33401988	-3.14325802
H	1.68913921	-4.18074298	-2.48584297
H	1.55118078	-5.66566954	0.02836753
H	0.36988330	-6.74116013	-0.76556181
H	-0.02225578	-5.96996956	0.79461777
H	2.58047889	1.74312651	2.25967133
H	-1.64221706	1.94719398	2.71385176
H	-0.63424037	0.82689238	3.64106780
C	0.38311087	2.96812621	1.15308793
C	-0.79915560	3.21513275	0.44579764
C	1.49090727	3.76100711	0.84806620
C	-0.83468408	4.18977507	-0.52192408
H	-1.70201748	2.64225035	0.61250446
C	1.42921068	4.75252131	-0.11859788
H	2.41893296	3.63198583	1.39112135
C	0.25768523	5.00720269	-0.84249765
H	2.30946805	5.35308872	-0.30498293
N	0.16383394	5.97031646	-1.85316345
C	1.42026803	6.55605932	-2.29802189
C	-0.86034670	6.99965295	-1.65803601
H	1.22067223	7.14798794	-3.19298976
H	1.87376557	7.21634420	-1.54479444
H	2.13319808	5.77154561	-2.55649282
H	-0.55567772	7.72077001	-0.88628488
H	-1.00253869	7.53704457	-2.59758866
H	-1.81015720	6.55792965	-1.36777495
F	-1.98992732	4.33334730	-1.22220809

*

TSEF24

Au	-0.19464141	-2.81881920	0.10196986
C	-0.05721744	-0.69155283	1.81918181
C	-0.90557079	-1.02035339	0.93799112
C	-1.23886419	1.46499030	1.96407761
C	-0.63157525	0.98604342	3.07912716
H	-1.17472735	0.33804282	3.75694957
C	-2.11806338	-0.30514654	0.47156853
H	-2.96483982	-0.97879324	0.33758877
H	-1.89404050	0.13653787	-0.50321219
C	-2.48960038	0.81235046	1.49892215
P	0.49603935	-4.76650690	-0.88885758
C	2.17693529	-4.67591761	-1.55081707
C	0.49719402	-6.18814825	0.22905878
H	0.88847401	-0.81260060	2.31643056
C	-0.54108293	-5.25791939	-2.28652368
H	-0.52658259	-4.47162847	-3.04346417
H	-0.16615464	-6.18820718	-2.71954756
H	-1.56785882	-5.40209194	-1.94549755
H	2.23196174	-3.87857845	-2.29422064
H	2.44673456	-5.62739541	-2.01516831
H	2.87501306	-4.45407755	-0.74163950
H	1.16741445	-5.99098339	1.06761715
H	0.83238963	-7.08098232	-0.30405989
H	-0.51176587	-6.34963389	0.61285134
H	0.30897110	1.39498299	3.43133067
H	-3.16789612	1.50738466	1.00436683
H	-3.00373286	0.35411529	2.34352585
C	-0.58446500	2.47595456	1.09496372
C	-1.34513361	3.42056056	0.41435334
C	0.80478004	2.49688272	0.94753549
C	-0.72537073	4.37585358	-0.38391642
H	-2.42338583	3.43525952	0.51843443
C	1.40622343	3.44636284	0.14074199
H	1.41467873	1.75723833	1.45022965
C	0.64944987	4.39797742	-0.53445207
C	-1.59433348	5.38860878	-1.08347408
C	2.89970800	3.41948340	-0.05882130
H	1.12731780	5.13740645	-1.16393894
F	-0.88622585	6.25562397	-1.82614606
F	-2.31683041	6.11720893	-0.20508187
F	-2.48396062	4.79549802	-1.90948753
F	3.54356157	2.84697375	0.97604626
F	3.41384150	4.65428699	-0.21228854
F	3.24224333	2.71809600	-1.16347891

*

TSEF3

Au	0.00027723	-2.11820041	0.23481171
C	1.29527326	0.16906184	0.96302714
C	0.20820070	-0.34460440	1.35572496
C	0.64795104	2.16961088	2.28506610
C	1.96842549	1.89424565	2.14015440
H	2.46933405	1.24384631	2.84778744
C	-0.79430745	0.15525651	2.32734359
H	-1.15673657	-0.63615341	2.98394627
H	-1.65204265	0.53060338	1.76266375
C	-0.16267572	1.30191728	3.17936440
P	-0.33400433	-4.06687649	-0.92320041
C	-0.04435061	-3.90901105	-2.70158548
C	0.73915682	-5.41831323	-0.38191489
H	2.14101682	0.22535011	0.30172019
C	-2.01939939	-4.70398229	-0.76836686
H	-2.72458013	-3.96376674	-1.15063528
H	-2.11988896	-5.63155897	-1.33670142
H	-2.24137571	-4.89453441	0.28317242
H	-0.71885113	-3.15690372	-3.11471762
H	-0.22107849	-4.86825657	-3.19392913
H	0.98606181	-3.59388851	-2.87561120
H	1.78315848	-5.13024661	-0.51783484
H	0.52935564	-6.31751221	-0.96597917
H	0.56299926	-5.62094437	0.67601555
H	2.59415149	2.46299161	1.46132798
H	-0.97521549	1.84177552	3.66502412
H	0.47520607	0.85782265	3.94352806
C	-0.04102420	3.16954537	1.43080148
C	-1.11588902	3.91250734	1.92722815
C	0.37250013	3.38529963	0.11798557
C	-1.74951406	4.85832592	1.13496304
H	-1.44927458	3.77006639	2.94799708
C	-0.27582197	4.32431688	-0.67282280
H	1.18779315	2.80638675	-0.29991022
C	-1.33615798	5.06861659	-0.17434569
H	-2.57338285	5.43517703	1.53889031
H	-1.83644368	5.80001302	-0.79651713
C	0.19726769	4.49947634	-2.09069564
F	0.07969406	3.35568343	-2.80455378
F	1.50363759	4.84512536	-2.14736747
F	-0.48590127	5.44640998	-2.75701422

*

TSEF4

Au	0.28007525	-2.69454650	0.49306748
C	1.33396066	-0.38266955	1.50143652
C	0.14454616	-0.80429774	1.41679218
C	0.35690042	1.78254853	2.21318421
C	1.57616152	1.44193930	2.70046413
H	1.65482689	0.86210576	3.61282060
C	-1.13366385	-0.15217372	1.79178173
H	-1.81537974	-0.84361375	2.28769273
H	-1.62097928	0.18534107	0.87306239
C	-0.83821930	1.06278144	2.72692596
P	0.29188027	-4.75740619	-0.50494691
C	1.94058162	-5.49047076	-0.63188791
C	-0.70881988	-5.97903848	0.37656014
H	2.38461208	-0.43616710	1.28055301
C	-0.35426600	-4.73765480	-2.19399807
H	0.25343631	-4.07032628	-2.80771040
H	-0.32850167	-5.74557574	-2.61481333
H	-1.38262804	-4.37198095	-2.18570848
H	2.58581780	-4.83436244	-1.21876123
H	1.87734658	-6.46826311	-1.11531715
H	2.36594045	-5.60430676	0.36684286
H	-0.32528844	-6.09972255	1.39128430
H	-0.67058473	-6.93829900	-0.14494880
H	-1.74277375	-5.63310988	0.42799888
H	2.48411239	1.90011662	2.32446221
H	-1.72859583	1.69063781	2.74926051
H	-0.64207609	0.68931706	3.73198005
C	0.20604119	2.70090414	1.05602756
C	-0.91070871	3.53520868	0.95925063
C	1.17276180	2.74786275	0.05031601
C	-1.04830248	4.40690851	-0.10829479
H	-1.66998529	3.52466161	1.73113071
C	1.03494922	3.61101147	-1.02624211
H	2.03343295	2.09139882	0.09282728
C	-0.07467105	4.44160380	-1.10013678
H	-1.91295991	5.05847368	-0.16816526
H	1.78905643	3.62976207	-1.80306977
C	-0.26229248	5.38293981	-2.25870643
F	-1.34065725	5.05113288	-3.00569001
F	-0.46472226	6.65418931	-1.84708971
F	0.79472506	5.40537214	-3.09071291

*

TSEF5

Au	-0.11293908	-1.64303456	0.18304449
C	0.98591153	0.57940174	1.24374929
C	-0.22332735	0.27576083	1.06066771
C	0.10235962	2.87954855	1.79704875
C	1.23752303	2.48376682	2.42388514
H	1.18100050	1.92383675	3.35019430
C	-1.47814859	1.02996714	1.29465187
H	-2.25012831	0.40265332	1.74138499
H	-1.85284023	1.37024496	0.32551504
C	-1.17914811	2.24781663	2.22147722
P	-0.15228121	-3.72119777	-0.77972424
C	0.49215173	-3.74472986	-2.46927194
C	0.81409414	-4.95642676	0.12081650
H	2.04838581	0.45081292	1.15011145
C	-1.82136777	-4.40814479	-0.89428534
H	-2.44778923	-3.74314090	-1.49146528
H	-1.78697993	-5.39492954	-1.36190206
H	-2.24909765	-4.49344765	0.10627797
H	-0.09603038	-3.06798054	-3.09161960
H	0.43506437	-4.75728049	-2.87566750
H	1.53111733	-3.41037454	-2.46716858
H	1.85765780	-4.63984658	0.16568182
H	0.74703389	-5.92242141	-0.38523652
H	0.42822610	-5.04934023	1.13754614
H	2.21126513	2.86410302	2.13735787
H	-2.02656544	2.93006546	2.16100585
H	-1.09172614	1.89118317	3.24793706
C	0.11982282	3.74013219	0.59242804
C	-0.98812052	4.51846910	0.24283550
C	1.24481360	3.79508578	-0.23712821
C	-0.97159463	5.33656247	-0.87499329
H	-1.87843289	4.50966622	0.85978490
C	1.27149852	4.59895255	-1.36345311
H	2.11053877	3.18129725	-0.01667343
C	0.16047791	5.37856859	-1.68317826
H	-1.83131518	5.94599079	-1.12969196
H	2.14933134	4.61939494	-2.00214534
O	0.13088938	6.18846977	-2.77476186
H	0.97838355	6.13203747	-3.23872719

*

TSEF6

Au	-0.03211209	-1.91078099	0.17728873
C	1.18958618	0.26271613	1.20799419
C	-0.02558704	-0.07103422	1.21567830
C	0.36356689	2.47690985	2.10511783
C	1.60334647	2.07582653	2.47940260
H	1.73063816	1.43915343	3.34733326
C	-1.23545483	0.61425197	1.72982487
H	-1.90224146	-0.07560638	2.24798968
H	-1.78321598	1.01845247	0.87424441
C	-0.80516885	1.76321067	2.69268830
P	-0.19592726	-3.91076155	-0.92773557

C 0.77380766 -3.97859131 -2.45269324
C 0.35462571 -5.32530972 0.05550808
H 2.22079273 0.18142238 0.91810961
C -1.89147112 -4.30188599 -1.42035810
H -2.26824539 -3.51867171 -2.08055706
H -1.91591614 -5.26170099 -1.94161382
H -2.52549913 -4.35229114 -0.53336822
H 0.44142523 -3.18983392 -3.12997653
H 0.64406290 -4.95138112 -2.93294990
H 1.82897362 -3.82431877 -2.22004239
H 1.40238118 -5.19186953 0.33063889
H 0.24308619 -6.24615979 -0.52177356
H -0.24443684 -5.39016859 0.96554274
H 2.49820450 2.51842263 2.05684701
H -1.66625684 2.41488106 2.83830984
H -0.52875085 1.32828733 3.65337592
C 0.13888574 3.43496102 0.99945947
C -1.01893267 4.22187944 0.94776474
C 1.06819159 3.57862383 -0.03107909
C -1.22535366 5.12506937 -0.07694336
H -1.76482899 4.14857957 1.73014427
C 0.87100549 4.47259167 -1.07464083
H 1.96096580 2.96362089 -0.04221769
C -0.28197446 5.25777455 -1.09910792
H -2.11771835 5.74049043 -0.10276592
H 1.61211370 4.54163394 -1.85972940
O -0.57279525 6.16100587 -2.06548911
C 0.37363189 6.32987800 -3.12048484
H 1.33876164 6.66772536 -2.73231039
H 0.50402126 5.40169726 -3.68431495
H -0.04241729 7.09535030 -3.77399803
*

TSEF7
Au -0.04641636 -1.86939228 0.14645921
C 1.15412012 0.33721594 1.17198354
C -0.05801157 -0.01385345 1.15183133
C 0.31081640 2.53188735 2.04148044
C 1.54149506 2.12709053 2.44215703
H 1.64886172 1.49769054 3.31804031
C -1.28071718 0.67122771 1.63575901
H -1.95825189 -0.01619259 2.14298744
H -1.80976560 1.07001864 0.76597772
C -0.87478989 1.82719793 2.60178346
P -0.18395862 -3.89189141 -0.92100314
C 0.79573493 -3.98081413 -2.43851608
C 0.37543226 -5.28045124 0.09393549
H 2.18859258 0.25684219 0.89261263
C -1.87220104 -4.31135415 -1.41557386
H -2.25339049 -3.54579247 -2.09370533
H -1.88318736 -5.28171543 -1.91740957
H -2.51103154 -4.35079582 -0.53147116
H 0.45783684 -3.21116889 -3.13478246
H 0.68081327 -4.96534158 -2.89817578
H 1.84750902 -3.80848997 -2.20320213
H 1.42027071 -5.13073527 0.37178040
H 0.27645062 -6.21403900 -0.46489165
H -0.22785313 -5.33292558 1.00199588
H 2.44548662 2.56639851 2.03597546
H -1.73720950 2.48300738 2.71708414
H -0.62596798 1.39874372 3.57278346
C 0.12059110 3.49890546 0.93417669
C -1.01097154 4.31244305 0.88181657
C 1.06892383 3.61790836 -0.08985812
C -1.17698841 5.22865557 -0.14788509
H -1.76413112 4.25371766 1.65821967
C 0.89626917 4.52343270 -1.11976799
H 1.94149311 2.97472064 -0.09486535
C -0.22942771 5.35396163 -1.16325721
H -2.05922240 5.86069883 -0.16371089
H 1.64241045 4.57510039 -1.90422649
C -0.45571176 6.34562111 -2.23469214
C 0.40118699 6.67364079 -3.19811970
H -1.42145883 6.84451163 -2.19721618
H 0.13569707 7.42138101 -3.93798561
H 1.38504382 6.22386111 -3.28623206
*

TSEF8
Au -0.12721225 -1.80549407 0.13950205
C 0.93931383 0.37664934 1.36229295
C -0.25936098 0.05460493 1.12555970
C 0.03344084 2.59449281 2.04603431
C 1.15874717 2.15186996 2.66042250
H 1.08712233 1.53663084 3.54987245
C -1.52517957 0.78477570 1.37797533
H -2.30524866 0.12780062 1.76360699
H -1.87726537 1.18686878 0.42417042
C -1.25932151 1.94361240 2.38973126
P -0.12069728 -3.82388351 -0.94464634
C 0.53895890 -3.73692917 -2.62648485
C 0.86198912 -5.09168869 -0.10874005
H 2.00313003 0.25701592 1.26640933
C -1.77459586 -4.53558726 -1.11553213
H -2.40804497 -3.85064025 -1.68212638
H -1.71613654 -5.49411394 -1.63654702
H -2.21083505 -4.68447678 -0.12609955
H -0.05491538 -3.03426976 -3.21376552
H 0.50252636 -4.72463008 -3.09219234
H 1.57221859 -3.38646775 -2.59444685
H 1.89842234 -4.75770114 -0.03320724
H 0.81991033 -6.02690152 -0.67203314
H 0.46803400 -5.25296523 0.89633861
H 2.13500890 2.55356812 2.41352976
H -2.10303643 2.63133989 2.33775565
H -1.20665489 1.52394762 3.39435842
C 0.08411133 3.55139442 0.91333537
C -0.97105324 4.43637684 0.68284660
C 1.18706817 3.58521891 0.05446860
C -0.92132986 5.34210814 -0.36511024
H -1.83428603 4.44016202 1.33674283
C 1.23786368 4.47873471 -1.00052910
H 2.00620090 2.89035968 0.19676151
C 0.18268413 5.36917778 -1.21796801
H -1.74134437 6.03211206 -0.52608230
H 2.09343719 4.48809108 -1.66575125
C 0.23157750 6.30288576 -2.31263080
C 0.26828609 7.08588044 -3.22230455
H 0.30205058 7.78167824 -4.03383777
*

TSEF9
Au -0.13836329 -1.79976586 0.14909686
C 0.92854537 0.39745415 1.37614435
C -0.26630885 0.06341639 1.12591927
C 0.02901542 2.60200382 2.03119481
C 1.14483694 2.15401839 2.66033817
H 1.05797631 1.55157457 3.55706656
C -1.53301601 0.79805188 1.36185563
H -2.31790736 0.14717068 1.74795340
H -1.87693982 1.19159748 0.40151510
C -1.27329774 1.96736845 2.36420635
P -0.12611303 -3.82051536 -0.93158092
C 0.53191323 -3.73258209 -2.61406283
C 0.86259005 -5.08344642 -0.09545258
H 1.99204803 0.27159311 1.28113220
C -1.77686895 -4.53968246 -1.10081053
H -2.41512006 -3.85644489 -1.66394090
H -1.71489640 -5.49649144 -1.62456632
H -2.20994844 -4.69349580 -0.11077584
H -0.06765361 -3.03570344 -3.20239630
H 0.50242144 -4.72148775 -3.07768815

H 1.56250361 -3.37443668 -2.58371521
H 1.89809089 -4.74599767 -0.02344864
H 0.82209526 -6.01997531 -0.65669500
H 0.47193438 -5.24393084 0.91101115
H 2.12659540 2.54645105 2.42040122
H -2.11007245 2.66163853 2.29233225
H -1.23717439 1.55920843 3.37412752
C 0.10470091 3.55443373 0.89424403
C -0.93074931 4.46292323 0.66546251
C 1.21109511 3.55517846 0.03907339
C -0.86117827 5.36460491 -0.38405435
H -1.79279547 4.48711738 1.31983173
C 1.28684528 4.44316195 -1.01828138
H 2.01230540 2.84135539 0.18590378
C 0.24785368 5.35074086 -1.22662708
H -1.66167850 6.07499417 -0.55151151
H 2.14093057 4.43416206 -1.68471598
C 0.31974908 6.27724071 -2.32672553
N 0.37676048 7.01681279 -3.20557662
*

TSFG1
C -2.12599860 -1.11588994 0.50011660
H -1.89571431 -2.01467781 -0.07047112
H -3.16972127 -0.86123668 0.27400636
C -1.26236643 0.05831559 0.09400046
C -1.21976640 1.05373128 1.00215413
H -0.52548077 1.88489252 0.98107946
C -2.22151920 1.05261720 2.19736526
H -2.12335646 1.96085850 2.77965170
H -3.24029736 0.86808240 1.86146924
C -1.51420693 -0.11324144 2.67411384
C -1.91106614 -1.38374030 1.99761400
H -1.17003076 -2.16616758 2.14325453
H -2.83361380 -1.71439142 2.49256007
Au -0.26990323 0.10985548 -1.70527461
P 0.83777536 0.15335276 -3.73171556
C 0.26714741 1.45629004 -4.85369194
C 2.62341983 0.42206680 -3.58613776
C 0.67870391 -1.37855342 -4.68554096
H -0.37636331 -1.57168334 -4.88896619
H 1.22194871 -1.29548238 -5.62984010
H 1.08219217 -2.21091041 -4.10574208
H -0.79544367 1.31748149 -5.06196275
H 0.82922069 1.42190976 -5.78998976
H 0.40754494 2.43014071 -4.38070248
H 2.80784992 1.37741174 -3.09108940
H 3.08627154 0.42954282 -4.57581472
H 3.06340728 -0.37579687 -2.98484273
C -0.51603624 -0.05351577 3.72455413
C 0.49522526 -1.02080521 3.82130540
C -0.55678834 0.97012245 4.68742685
C 1.43276509 -0.95935814 4.83645168
H 0.57225827 -1.80699915 3.08110863
C 0.37260119 1.01311158 5.70682200
H -1.33972675 1.71718484 4.65442329
C 1.38548956 0.05281404 5.79620915
H 2.21763033 -1.70630616 4.88695262
H 0.31714114 1.80071173 6.45120145
C 2.39288300 0.11673836 6.90831871
H 3.13778563 -0.67461318 6.81497378
H 2.90536135 1.08249131 6.90601614
H 1.89620764 0.01427997 7.87730300
*

TSFG12
C -1.36933392 2.18827347 -0.14096465
H -2.25576780 1.79925708 -0.64026985
H -1.22905426 3.21379273 -0.50692492
C -0.12524179 1.39734714 -0.48131963
C 0.89417864 1.57374439 0.38487843
H 1.78831212 0.96353079 0.42179397
C 0.82912370 2.72282066 1.43607907
H 1.76072832 2.79189415 1.98473318
H 0.52542079 3.66416550 0.98245096
C -0.23634069 1.95785459 2.03979863
C -1.56591652 2.14132452 1.38162443
H -2.25994976 1.34923314 1.65275553
H -1.97519923 3.08085399 1.77432411
Au -0.02697590 0.17596829 -2.13106651
P 0.07277971 -1.19235534 -3.98964328
C 1.41385435 -0.77439821 -5.13371413
C 0.33265500 -2.94140591 -3.59538929
C -1.42559989 -1.16753603 -5.00739680
H -1.60848465 -0.15081910 -5.36050294
H -1.31086756 -1.83536958 -5.86441112
H -2.27847683 -1.48957590 -4.40684587
H 1.28262509 0.24961923 -5.48867031
H 1.41150116 -1.45817142 -5.98590963
H 2.37068584 -0.84524345 -4.61292262
H 1.26962534 -3.05450432 -3.04670373
H 0.37417413 -3.53347795 -4.51268102
H -0.48659873 -3.29871842 -2.96859550
C -0.03819373 1.10305912 3.19403779
C -0.88068246 0.00498562 3.43784217
C 0.99129704 1.36669019 4.11054231
C -0.69465386 -0.79787952 4.54403240
H -1.66655085 -0.24356433 2.73578459
C 1.16069080 0.57001752 5.22669564
H 1.64378826 2.21904488 3.96845543
C 0.32766349 -0.52783291 5.46297225
H -1.35167130 -1.64714308 4.69336855
H 1.95045623 0.79886598 5.93406962
C 0.55045132 -1.36187456 6.66698668
C -0.61914182 -1.98316666 7.39646891
C 0.31250350 -2.85439891 6.62128375
H 1.37581509 -1.03092922 7.28583416
H -1.60613914 -1.80229496 6.98712322
H -0.56411409 -2.00492132 8.47807322
H 1.01039498 -3.47639331 7.16852615
H -0.03870479 -3.26866225 5.68350113
*

TSFG16
C -1.43437191 2.02051320 0.23427122
H -2.31409023 1.72685837 -0.33675045
H -1.24725260 3.07700048 0.00122643
C -0.20588599 1.23107670 -0.15719919
C 0.78278252 1.26818931 0.76430800
H 1.64944018 0.61871795 0.76447068
C 0.72815390 2.30357892 1.92408098
H 1.64652151 2.28640977 2.49791875
H 0.45520598 3.29627162 1.57358534
C -0.36399669 1.48273883 2.38730041
C -1.67644358 1.79233707 1.73307207
H -2.39741054 0.99194936 1.88132425
H -2.07169393 2.68493168 2.23309452
Au -0.08045895 0.19808188 -1.92767850
P 0.04989987 -0.95414729 -3.92623360
C 1.52692344 -0.56530097 -4.90010015
C 0.08509029 -2.75503015 -3.73326821
C -1.33729878 -0.63652245 -5.04712253
H -1.37848923 0.42902003 -5.28096756
H -1.21571341 -1.20738626 -5.97072214
H -2.27104939 -0.92573072 -4.56104544
H 1.53790949 0.50121391 -5.13295436
H 1.52969354 -1.14074532 -5.82885693
H 2.41922168 -0.80705802 -4.31950866
H 0.95032439 -3.03932875 -3.13130440
H 0.14739183 -3.23816446 -4.71124906
H -0.82172448 -3.08393627 -3.22211332
C -0.21645263 0.48656791 3.43156222

C	-1.09934118	-0.59672330	3.52572219
C	0.82130483	0.60066594	4.37673088
C	-0.95321253	-1.54289679	4.52381558
H	-1.88766080	-0.72716473	2.79634918
C	0.95700346	-0.33192562	5.37825066
H	1.50757733	1.43903944	4.34935553
C	0.06858904	-1.41778645	5.45611782
H	-1.62419827	-2.39119506	4.59307698
O	1.91660657	-0.29157689	6.34834464
O	0.19613813	-2.34007315	6.43034986
H	2.48145274	0.48416263	6.22455021
H	0.95268470	-2.11016086	6.99205190

*

TSFG17

C	-1.52288982	2.07252597	-0.27558847
H	-2.35967744	1.60262299	-0.79043196
H	-1.48561481	3.11410609	-0.62061717
C	-0.20067638	1.42018166	-0.61139036
C	0.78298476	1.69753600	0.27465747
H	1.73095192	1.17612467	0.32263294
C	0.60027157	2.83757783	1.31617857
H	1.51976688	3.00585011	1.86326181
H	0.18405607	3.73878291	0.87224309
C	-0.35539388	1.92105207	1.88711501
C	-1.70953689	1.97255219	1.24454017
H	-2.30311398	1.09624199	1.49512717
H	-2.22335545	2.84651944	1.66246716
Au	0.04406131	0.21411237	-2.25502292
P	0.30278526	-1.13349320	-4.11429830
C	0.34245051	-0.21439013	-5.67495271
C	1.83010982	-2.10724253	-4.11326497
C	-1.02018096	-2.35222662	-4.32979037
H	-1.97826656	-1.83682083	-4.42035608
H	-0.84144354	-2.94909805	-5.22729515
H	-1.05528993	-3.00814985	-3.45784883
H	-0.58952948	0.34170771	-5.79293259
H	0.46563985	-0.90140039	-6.51550476
H	1.17364265	0.49312681	-5.65838526
H	2.68927167	-1.43522226	-4.06882903
H	1.89058482	-2.71627550	-5.01829646
H	1.84651682	-2.75675835	-3.23595991
C	-0.05106526	1.06969772	3.02093781
C	-0.75100322	-0.14116547	3.21883434
C	0.91716701	1.44843284	3.94850824
C	-0.48026308	-0.94372187	4.30299172
H	-1.48215329	-0.45987229	2.49059379
C	1.18446005	0.65638474	5.05740185
H	1.45029418	2.38380273	3.83827376
C	0.50244932	-0.53920702	5.24633354
O	-1.08248840	-2.12822738	4.56267549
H	1.92576549	0.98120628	5.77446381
O	0.69510819	-1.37604314	6.27667767
C	-2.08145397	-2.56484063	3.64323121
C	1.67533357	-1.01520851	7.25476967
H	-2.44226532	-3.51809978	4.02671138
H	-2.91087173	-1.85271308	3.59672376
H	-1.66004184	-2.70956418	2.64381933
H	1.40922582	-0.07391296	7.74271877
H	1.66990170	-1.82120297	7.98648232
H	2.66673419	-0.93674515	6.80117436

*

TSFG18

C	-1.21124408	2.38019188	-0.28933462
H	-2.10927835	2.11479211	-0.84520077
H	-0.91297072	3.38129257	-0.62870010
C	-0.06838590	1.43695844	-0.56075752
C	0.88202345	1.47022990	0.41964811
H	1.65827331	0.72463663	0.52961568
C	0.99358520	2.68630870	1.37684626
H	1.91840502	2.64890843	1.93801607
H	0.78729277	3.63858937	0.89854101
C	-0.13034764	1.92895031	1.85739953
C	-1.44775830	2.31768606	1.22429105
H	-2.21794291	1.58192026	1.44805595
H	-1.76477204	3.27366284	1.65224395
Au	-0.01262296	0.17387610	-2.17109464
P	0.04448770	-1.24346889	-3.99831669
C	0.78857060	-0.49712769	-5.47120340
C	0.99382030	-2.75944956	-3.71556039
C	-1.58794457	-1.81087148	-4.53919388
H	-2.20182387	-0.94900284	-4.80757184
H	-1.49002395	-2.47170151	-5.40367207
H	-2.07444189	-2.34877339	-3.72330460
H	0.21834567	0.38861776	-5.75754257
H	0.78836961	-1.21302256	-6.29654935
H	1.81460441	-0.19820854	-5.24863096
H	2.02281790	-2.50064526	-3.45897351
H	0.98800193	-3.38177320	-4.61350163
H	0.55363745	-3.31476733	-2.88519920
C	-0.05199797	1.02911485	3.00502395
C	-0.96250215	-0.02578517	3.16265559
C	0.92785594	1.18524622	3.99485467
C	-0.90040617	-0.88493228	4.23732965
H	-1.72651516	-0.19637329	2.41257348
C	1.00018328	0.34410977	5.08517141
H	1.63658352	2.00298665	3.93047203
C	0.09249869	-0.73034075	5.23467346
H	-1.62233742	-1.68687957	4.30560304
H	1.76076293	0.52480700	5.83207942
N	0.16952854	-1.58212771	6.29308779
C	-0.87143031	-2.57551397	6.50321527
C	1.11395679	-1.32283329	7.36756707
H	-0.62707507	-3.15741199	7.38968514
H	-1.85398553	-2.11246475	6.65191765
H	-0.93662380	-3.26353678	5.65591219
H	1.05066778	-2.13177466	8.09278622
H	2.13918510	-1.28865871	6.98965465
H	0.90086336	-0.37777153	7.88123611

*

TSFG22

C	0.23246552	2.26035415	-0.93694796
H	-0.67314623	2.61663632	-1.43155250
H	1.05817873	2.79387160	-1.43048123
C	0.44196029	0.81374339	-1.21386144
C	0.94194334	0.17563323	-0.06553115
H	0.96722168	-0.89782093	0.04227605
C	2.18155895	0.93076087	0.54947160
H	2.84485491	0.23214521	1.04350874
H	2.65186579	1.67662373	-0.07939981
C	0.87369659	1.18175696	1.14070361
C	0.19943587	2.43187929	0.58982859
H	-0.83335860	2.47555861	0.93999123
H	0.71213426	3.33645934	0.92135750
Au	0.03759238	-0.08570111	-2.99155463
P	-0.41674370	-1.09894621	-5.02775427
C	-1.60020909	-0.18055933	-6.04324786
C	1.05030816	-1.29576122	-6.06935627
C	-1.11455192	-2.76138696	-4.87170369
H	-2.04946915	-2.71303433	-4.31026618
H	-1.30540798	-3.18188821	-5.86180691
H	-0.41248652	-3.40075750	-4.33337554
H	-2.54564022	-0.08846590	-5.50541489
H	-1.76784562	-0.70192995	-6.98851618
H	-1.20916542	0.81886936	-6.24268693
H	1.48843511	-0.31633391	-6.27007084
H	0.77718103	-1.77345020	-7.01316480
H	1.78600780	-1.91156903	-5.54878466
C	0.52746816	0.70185239	2.51343049
C	0.54686356	1.61394699	3.57016739

C 0.18182080 -0.61439826 2.80530528
C 0.23884978 1.23541651 4.86548391
H 0.80657627 2.65080803 3.37931584
C -0.12594417 -1.01632227 4.09719327
H 0.14416801 -1.35648413 2.01537872
C -0.10602515 -0.09865620 5.16444494
H 0.25507551 1.98053837 5.65098909
H -0.38508855 -2.05200483 4.27740845
N -0.41973047 -0.48011036 6.43644931
C -0.79283506 -1.84306436 6.79617949
C -0.22066234 0.37436712 7.60425007
H -1.61102472 -2.20372746 6.16692617
H 0.05549894 -2.53075434 6.67565426
C -1.20103201 -1.71595166 8.26227616
H 0.74297210 0.88936716 7.55348899
H -1.00703388 1.13682930 7.66948337
C -0.28763782 -0.60267091 8.77927446
H -2.24727068 -1.40384013 8.32775845
H -1.08654453 -2.65441533 8.80649172
H 0.70931954 -1.00408931 8.98173164
H -0.65804451 -0.12909886 9.68948622
*

TSFG23
C -1.21084708 2.40503409 -0.52321495
H -2.09835784 2.09284099 -1.07167900
H -0.95413890 3.41012297 -0.88302957
C -0.02971132 1.49844955 -0.77605934
C 0.93935158 1.60608705 0.16856781
H 1.75395485 0.90262875 0.28549844
C 0.95894140 2.80850674 1.14794800
H 1.87488654 2.82065460 1.72501773
H 0.72671459 3.75311186 0.66379997
C -0.15992978 2.06561698 1.66904654
C -1.46727992 2.37499514 0.98902724
H -2.22258638 1.62842158 1.22403420
H -1.81948877 3.33572526 1.38029732
Au 0.05220680 0.19005553 -2.35332124
P 0.13565833 -1.28370370 -4.13244758
C 0.88585531 -0.58419452 -5.62542981
C 1.09282904 -2.78344359 -3.79279709
C -1.48767457 -1.88240970 -4.66794976
H -2.10591865 -1.03511015 -4.97063189
H -1.37773330 -2.57154316 -5.50858329
H -1.97728719 -2.39594255 -3.83830843
H 0.31163525 0.28696224 -5.94658280
H 0.89691672 -1.32822102 -6.42546742
H 1.90802242 -0.27002224 -5.40603200
H 2.11870321 -2.50971887 -3.53911438
H 1.09629111 -3.43632614 -4.66879155
H 0.65112364 -3.31282302 -2.94643172
C -0.05983152 1.19000479 2.82150205
C -0.99103152 0.15735976 3.02937845
C 0.95082052 1.34836327 3.77828081
C -0.88249432 -0.66245325 4.11629165
H -1.80771259 -0.02259635 2.34152120
C 1.04546030 0.51724954 4.87530205
H 1.67584451 2.14629397 3.67737016
C 0.14380111 -0.54853151 5.08412897
F -1.85144266 -1.59935387 4.29388836
H 1.84677649 0.67839984 5.58290822
N 0.23287621 -1.37764479 6.16009116
C -0.18088079 -2.78052262 6.08731313
C 1.16159995 -1.05974704 7.23384615
H 0.55614526 -3.38061289 6.62362991
H -1.16075366 -2.94112729 6.54407239
H -0.20448967 -3.12880519 5.05626758
H 0.88779729 -1.65171847 8.10723710
H 2.20057150 -1.29027658 6.96645727
H 1.08827139 -0.00593854 7.50535428
*

TSFG5
C -2.05409614 -1.24613999 0.52807505
H -1.81560436 -2.15001928 -0.03070796
H -3.10393892 -1.00981357 0.31008857
C -1.21104956 -0.06685398 0.10008222
C -1.18239702 0.93784997 1.00558179
H -0.48883890 1.76915849 0.98010191
C -2.20610414 0.96328497 2.17560487
H -2.13413767 1.89204414 2.72793023
H -3.21536450 0.73072647 1.84415010
C -1.42623294 -0.15509108 2.64473233
C -1.80915400 -1.46718867 2.02692309
H -1.03741390 -2.21908916 2.17488986
H -2.70769040 -1.81187517 2.55262803
Au -0.20539586 -0.02167715 -1.68935676
P 0.91312505 0.02554115 -3.71071478
C 0.05514003 0.97320011 -4.99441723
C 2.56439043 0.76462270 -3.61701870
C 1.17971400 -1.60871742 -4.44509005
H 0.21602410 -2.09311556 -4.61384079
H 1.71067053 -1.51382709 -5.39522881
H 1.76670272 -2.22359047 -3.76018515
H -0.92919887 0.53578379 -5.17204376
H 0.63181313 0.96094819 -5.92232967
H -0.07411594 2.00427751 -4.65992565
H 2.48196426 1.79407449 -3.26312195
H 3.03885222 0.75476473 -4.60115044
H 3.17670251 0.19881427 -2.91232084
C -0.41065355 -0.03270151 3.67350329
C 0.63979320 -0.96190090 3.77588591
C -0.46544210 1.01182638 4.61018933
C 1.59517238 -0.85228573 4.76230946
H 0.72830528 -1.76493496 3.05511665
C 0.47726093 1.12365360 5.61137335
H -1.27302138 1.73208335 4.57574318
C 1.51448116 0.19154878 5.68812521
H 2.41199343 -1.56118204 4.83111361
H 0.41489713 1.92533071 6.34017687
O 2.46736939 0.25332918 6.64163896
H 2.30583602 1.01802162 7.21450819
*

TSFG6
C -2.51856098 -0.64347714 0.06527029
H -2.46143226 -1.54606049 -0.54146685
H -3.46888748 -0.15462364 -0.18647935
C -1.39819110 0.32536986 -0.23764412
C -1.20072013 1.24766224 0.73254205
H -0.33245746 1.89147184 0.79893946
C -2.26030166 1.44729895 1.85205533
H -2.01030229 2.30121908 2.46943130
H -3.27451843 1.47343335 1.46092003
C -1.78492246 0.15364701 2.27560722
C -2.42547444 -0.99678060 1.55593641
H -1.85853381 -1.91493029 1.69106744
H -3.40989926 -1.14821522 2.01444406
Au -0.27084733 0.21258890 -1.94992525
P 0.99431131 0.07782492 -3.87903698
C 0.19815989 0.82064142 -5.32669823
C 2.60296884 0.90270470 -3.76293555
C 1.38280300 -1.61781788 -4.38466468
H 0.45550751 -2.16771660 -4.55634642
H 1.97770718 -1.61226026 -5.30106724
H 1.94342941 -2.11432870 -3.59033653
H -0.75261797 0.31871091 -5.51593655
H 0.84244450 0.72309386 -6.20366351
H 0.00494750 1.87741932 -5.13273042
H 2.45390120 1.96031564 -3.53711860
H 3.14610290 0.80476741 -4.70588073
H 3.18635963 0.45062861 -2.95856908

C	-0.82278498	-0.02649725	3.34523053
C	-0.02169330	-1.18277870	3.41517658
C	-0.67679192	0.93788578	4.35105108
C	0.88121503	-1.35776792	4.43571405
H	-0.08734181	-1.93753178	2.64150118
C	0.21653353	0.76795690	5.39358144
H	-1.29176351	1.82918173	4.34260288
C	1.00666836	-0.38457961	5.43909959
H	1.50887017	-2.24013309	4.48206870
H	0.28759459	1.52568648	6.16177982
O	1.90810355	-0.64698041	6.39955528
C	2.08131749	0.31724728	7.44378468
H	2.85059617	-0.09122558	8.09667938
H	2.41560241	1.27479731	7.03662252
H	1.15441641	0.45034569	8.00738578

*

alt-constrF14

Au	0.02319123	-2.19363348	0.16477367
C	1.34679422	0.16779700	1.43049125
C	0.16863005	-0.39420328	1.17371405
C	0.28415864	2.18425394	2.00921967
C	1.52781652	1.47734407	2.27265541
H	1.60865744	1.20732561	3.32651992
C	-1.10303226	0.28161852	1.57641851
H	-1.78798058	-0.39712697	2.08811362
H	-1.61716633	0.65197939	0.68522160
C	-0.88530269	1.52052049	2.55668040
P	-0.14493338	-4.20188115	-0.95728083
C	1.01873929	-4.38030322	-2.33479914
C	0.15564687	-5.65528114	0.08248354
H	2.29168988	-0.22575907	1.06966910
C	-1.77408309	-4.49561389	-1.69424855
H	-1.99940727	-3.70010556	-2.40714917
H	-1.78991359	-5.45968135	-2.20814571
H	-2.53377649	-4.48929375	-0.91016774
H	0.84876956	-3.58328610	-3.06121373
H	0.88426149	-5.34990176	-2.82021941
H	2.04083261	-4.30048051	-1.95955632
H	1.16042532	-5.59759963	0.50548725
H	0.06161913	-6.56950542	-0.50838358
H	-0.56817142	-5.67422947	0.89959423
H	2.42516233	1.99958410	1.95495508
H	-1.79708044	2.11022779	2.55956874
H	-0.67049304	1.11578709	3.54478973
C	0.18458424	3.28798330	1.07742764
C	-0.98134003	4.06881351	1.03126689
C	1.24558363	3.57989130	0.20258830
C	-1.08737699	5.12075385	0.13897264
H	-1.79946028	3.88058064	1.71215037
C	1.13989750	4.61346009	-0.70681913
H	2.14561347	2.98070707	0.20764928
C	-0.02503183	5.36410553	-0.71253685
H	-1.97452882	5.73729266	0.10483481
H	1.94290923	4.83642203	-1.39518307
N	-0.13662721	6.48652359	-1.67598276
O	-1.18879062	7.09144357	-1.71633483
O	0.83058573	6.73306863	-2.36758187

*

alt-constrF15

Au	0.03065875	-2.13347321	0.20060552
C	1.56953727	0.29889221	0.99953242
C	0.37344208	-0.28363566	1.05826042
C	0.65374538	2.35915311	1.66791019
C	1.93131464	1.65861161	1.68471531
H	2.26866695	1.46607130	2.70429918
C	-0.77998919	0.40661833	1.71842443
H	-1.29119823	-0.24599861	2.42891785
H	-1.51292900	0.69127058	0.95758261
C	-0.36219539	1.71705952	2.49744465
P	-0.35286334	-4.19603500	-0.75950784
C	0.51880849	-4.45286291	-2.32732974
C	0.14516967	-5.59865850	0.27416011
H	2.40677104	-0.11805717	0.44909903
C	-2.09375247	-4.51419561	-1.15010897
H	-2.45561925	-3.75283436	-1.84380399
H	-2.20613107	-5.50199674	-1.60328360
H	-2.68677385	-4.46481986	-0.23481984
H	0.20615510	-3.69252607	-3.04568124
H	0.29510493	-5.44480772	-2.72696288
H	1.59442581	-4.36048111	-2.16495520
H	1.21340502	-5.53090955	0.48894375
H	-0.06371856	-6.54034574	-0.23908949
H	-0.40450666	-5.56876361	1.21692653
H	2.72198387	2.13426585	1.11271596
H	-1.23952817	2.32741880	2.69215506
H	0.09236304	1.40282831	3.43740019
C	0.35270139	3.42503374	0.75411070
C	-0.98875483	3.76799929	0.49638681
C	1.38896428	4.11840508	0.10122852
C	-1.26790062	4.75934919	-0.40909367
H	-1.81701187	3.25738008	0.96845055
C	1.07571027	5.12232890	-0.78026349
H	2.43300217	3.90937415	0.28838775
C	-0.24713474	5.44997048	-1.05288070
F	-2.53112430	5.09514416	-0.69358489
F	-0.53266553	6.41565388	-1.91088997
F	2.03647493	5.81426586	-1.40290800

*

alt-constrF19

Au	0.07258977	-2.71823588	0.17311926
C	1.80739643	-0.39321034	0.90281426
C	0.61382762	-0.94416096	1.09225005
C	1.06296103	1.59727482	1.90794640
C	2.30035381	0.88232687	1.68178934
H	2.75108202	0.55911420	2.62046567
C	-0.41374398	-0.28546545	1.94676237
H	-0.87069040	-0.97130837	2.66218180
H	-1.21148985	0.11688372	1.31735734
C	0.15901021	0.93966872	2.82151682
P	-0.55582272	-4.68924053	-0.84502520
C	-0.49385619	-4.63622991	-2.65467875
C	0.46460833	-6.11541992	-0.39087059
H	2.54642632	-0.75313323	0.19506070
C	-2.25197047	-5.19514679	-0.45818908
H	-2.94598770	-4.41731795	-0.78242200
H	-2.49237658	-6.13206078	-0.96626680
H	-2.35451494	-5.32978728	0.62029035
H	-1.15450645	-3.84678587	-3.01810106
H	-0.80942528	-5.59551361	-3.07175729
H	0.52561284	-4.41728188	-2.97791353
H	1.50247003	-5.92366268	-0.66988398
H	0.10958502	-7.01245424	-0.90378064
H	0.41391119	-6.26960893	0.68866436
H	3.03307038	1.41389873	1.08151019
H	-0.69394547	1.52369756	3.15447932
H	0.69960321	0.50072247	3.65821697
C	0.64649800	2.72127969	1.07451363
C	-0.40820586	3.54247168	1.47659408
C	1.29789988	2.98480240	-0.13442555
C	-0.79934359	4.62465638	0.69465770
H	-0.92352295	3.37819768	2.41257750
C	0.88890798	4.03807147	-0.94073041
H	2.11288364	2.36335686	-0.48004383
C	-0.15141281	4.85639949	-0.51122402
N	-0.56813691	5.99494844	-1.35161264
O	-0.73402280	5.77079922	-2.53015333
O	-0.71037455	7.06106739	-0.79518045
C	-1.89235927	5.44424252	1.15115649
N	-2.77778468	6.05546377	1.55018707

C 1.58927502 4.27302048 -2.17763489
N 2.18949373 4.42599303 -3.14363565
*

alt-constrF20
Au -0.06391436 -3.37093466 -0.26534515
C -2.06175963 -1.14966259 -0.30279305
C -0.99543525 -1.64480475 -0.92402387
C -1.82434667 0.87644269 -1.46964162
C -2.86127091 0.09841668 -0.81983019
H -3.61056159 -0.23813627 -1.53665333
C -0.39628338 -0.94331413 -2.09782872
H -0.21318029 -1.61671853 -2.93712201
H 0.56413744 -0.50616196 -1.81117103
C -1.30586287 0.24543351 -2.66746998
P 0.98716898 -5.28588336 0.47405913
C 1.32289599 -5.29773468 2.25453954
C 0.05017673 -6.80731682 0.17480389
H -2.46710340 -1.54572203 0.62220752
C 2.59950625 -5.58129976 -0.29776144
H 3.26213447 -4.73913213 -0.08922667
H 3.04377274 -6.49933711 0.09407360
H 2.47462275 -5.67041492 -1.37864063
H 1.96194112 -4.45020505 2.50976317
H 1.82084304 -6.22763802 2.53937360
H 0.38311980 -5.20617948 2.80245735
H -0.91323568 -6.74707608 0.68472059
H 0.60513291 -7.67207599 0.54634383
H -0.12618285 -6.92136242 -0.89648617
H -3.34920395 0.58351963 0.01984452
H -0.68659535 0.86795323 -3.30580541
H -2.11285043 -0.21479320 -3.23524903
C -1.16970309 2.00971772 -0.83504503
C -1.51088957 2.39245120 0.46752755
C -0.16885611 2.71799061 -1.50804405
C -0.86540507 3.44363061 1.09021950
H -2.27329829 1.86030162 1.01705851
C 0.48227586 3.77834360 -0.90209688
H 0.10487535 2.45802923 -2.51978765
C 0.12636917 4.12640471 0.39431499
C -1.27188996 3.81793671 2.50488009
C 1.56683198 4.50911823 -1.67391633
N 0.81997959 5.25883989 1.05374891
F -1.87540836 2.79217356 3.12107902
F -2.12926701 4.85116624 2.51652151
F -0.21006083 4.16488882 3.25353375
F 1.42148745 4.33416835 -2.99477467
F 2.79074875 4.06565142 -1.34442234
F 1.53976833 5.83314266 -1.44183279
O 0.16684642 6.25946567 1.24144191
O 1.98036478 5.08592635 1.34866358
*

alt-constrF21
Au -0.02693828 -2.52529811 0.18017804
C 1.37014793 -0.22311271 1.46894233
C 0.17439804 -0.74377981 1.21336062
C 0.36722604 1.80687488 2.10267826
C 1.58889361 1.06440302 2.34323091
H 1.66869063 0.76303882 3.38826755
C -1.07419660 -0.03985395 1.62869618
H -1.78117851 -0.70240960 2.13115496
H -1.57552603 0.36941105 0.74773463
C -0.81819426 1.17779348 2.64156109
P -0.25278519 -4.50973566 -0.97267685
C -1.74628177 -4.59763829 -1.99460348
C 1.10774857 -4.85382421 -2.11876637
H 2.30383058 -0.62636118 1.09098402
C -0.33607137 -5.97493165 0.08970194
H -1.19016893 -5.88791121 0.76402176
H -0.44237289 -6.87667866 -0.51800344
H 0.57595315 -6.04355897 0.68574923
H -2.62779734 -4.48235829 -1.36103142
H -1.79407518 -5.55792743 -2.51353634
H -1.73258316 -3.78868781 -2.72759875
H 1.17474260 -4.05106325 -2.85568369
H 0.93995735 -5.80450261 -2.63047680
H 2.04695731 -4.90021809 -1.56418629
H 2.49869087 1.56875150 2.03080920
H -1.71438591 1.79102422 2.65102608
H -0.61832910 0.74073255 3.61857426
C 0.29575955 2.92311121 1.17062592
C -0.82732909 3.75834515 1.17488104
C 1.34278507 3.14707672 0.26594589
C -0.88050917 4.81350326 0.29025517
H -1.64569966 3.62802894 1.86909438
C 1.23846876 4.18239910 -0.63214340
H 2.21925070 2.51495444 0.22578364
C 0.13849321 5.02977143 -0.62309454
F -1.93902018 5.62481281 0.28789922
F 2.21729926 4.40303640 -1.51089286
N 0.05776393 6.14688861 -1.57302521
O 0.10438778 5.86799780 -2.75117530
O -0.04937932 7.25873509 -1.10373360
*

alt-constrF24
Au -0.33260644 -2.96409610 0.03505977
C 0.08930227 -0.68186902 1.91514275
C -0.72190522 -1.16460998 0.97669661
C -0.95559112 1.42108548 1.80406851
C -0.19103826 0.61808985 2.74440111
H -0.78132962 0.35544445 3.62340896
C -1.93543018 -0.40199592 0.54780894
H -2.82878986 -1.02932985 0.53397512
H -1.78588105 -0.02970831 -0.47000851
C -2.25274582 0.84490315 1.47312383
P 0.09156647 -4.97383639 -1.01463146
C 1.74851150 -5.09563742 -1.73815776
C -0.03637481 -6.41684078 0.07415235
H 1.03351337 -1.14329970 2.18505241
C -1.03379940 -5.33556743 -2.38799015
H -0.95416781 -4.54816635 -3.13998225
H -0.78241308 -6.29770731 -2.84059534
H -2.06065666 -5.36513443 -2.01847558
H 1.88227011 -4.30295872 -2.47682980
H 1.88082549 -6.06750986 -2.21956092
H 2.49761402 -4.97378020 -0.95339660
H 0.67998689 -6.31831629 0.89205641
H 0.17251484 -7.33252552 -0.48411148
H -1.04281098 -6.46844246 0.49390442
H 0.76067620 1.04752141 3.04343239
H -2.93280336 1.52015017 0.96117864
H -2.71534471 0.45830007 2.38146945
C -0.39191564 2.53855285 1.08813013
C -0.98175154 2.98011552 -0.10401008
C 0.76212890 3.18857376 1.56305133
C -0.41197312 4.02153764 -0.81635245
H -1.86901984 2.49637283 -0.49149204
C 1.29863238 4.24042317 0.85308044
H 1.21681523 2.88631988 2.49540107
C 0.72337873 4.66017946 -0.34448633
C -1.06428500 4.45130006 -2.10784822
C 2.52148946 4.97049672 1.35166132
H 1.16002925 5.48332860 -0.89773762
F -0.38068439 5.42504639 -2.72829558
F -2.31518592 4.91101697 -1.89969313
F -1.17022781 3.42220093 -2.97287057
F 3.00417015 4.44451419 2.48877266
F 2.25490211 6.26994696 1.59455587
F 3.51732129 4.94269353 0.44335938
*

alt-constrF4

Au	-0.03756950	-2.50816702	0.15404252
C	1.27891036	-0.22609818	1.56112322
C	0.09932460	-0.75782201	1.24589576
C	0.25335138	1.80099520	2.16849171
C	1.46947502	1.04930890	2.44823480
H	1.52098526	0.74848066	3.49572102
C	-1.17413049	-0.07834117	1.64514175
H	-1.87134853	-0.76933811	2.12322095
H	-1.67000918	0.31375498	0.75223000
C	-0.95235829	1.12891814	2.64359059
P	-0.19609044	-4.45542401	-1.07308689
C	1.39614312	-5.09425179	-1.65677390
C	-0.94093892	-5.84014958	-0.17210245
H	2.22436750	-0.62868036	1.21197890
C	-1.20106904	-4.29571301	-2.57271346
H	-0.76698697	-3.53254577	-3.22155449
H	-1.24032799	-5.24791909	-3.10706789
H	-2.21312851	-3.98998049	-2.30047284
H	1.87963940	-4.34478171	-2.28637701
H	1.24927386	-6.01190537	-2.23128099
H	2.04065721	-5.30085527	-0.80017685
H	-0.34174706	-6.06264023	0.71293606
H	-0.98974048	-6.72557734	-0.81041257
H	-1.94844660	-5.56593520	0.14666356
H	2.39085511	1.54264925	2.15497841
H	-1.84119012	1.75280036	2.66803481
H	-0.76281394	0.70138919	3.62845585
C	0.21589118	2.95340955	1.30596894
C	-1.00935191	3.39505696	0.76868988
C	1.39397272	3.64417625	0.97541600
C	-1.04728013	4.47511636	-0.08727631
H	-1.93086243	2.88106237	1.00467548
C	1.35308024	4.74065308	0.13287844
H	2.34548161	3.34316344	1.39081257
C	0.13518119	5.14381793	-0.39638682
H	-1.98894986	4.80362866	-0.51101409
H	2.26330746	5.27389299	-0.10751296
C	0.05632877	6.32930977	-1.33192443
F	-0.40190344	5.96068959	-2.54550010
F	-0.79005676	7.26751741	-0.86292711
F	1.24503060	6.92277565	-1.51944107

*

alt-constrF9

Au	0.03339574	-1.89789668	0.15156737
C	1.31423221	0.54207739	1.30727619
C	0.14551649	-0.04605445	1.06470669
C	0.22732893	2.57517986	1.77149871
C	1.47383817	1.89139642	2.08596959
H	1.54097116	1.67521068	3.15322733
C	-1.13824757	0.63533298	1.41916786
H	-1.82073748	-0.02534506	1.95723402
H	-1.64678129	0.95040660	0.50370247
C	-0.94581194	1.92505518	2.33224901
P	-0.09638749	-3.96858711	-0.85655732
C	0.31261688	-3.95800452	-2.62180705
C	1.01364472	-5.20868085	-0.13948519
H	2.26664801	0.13826184	0.97863896
C	-1.74081546	-4.72526268	-0.77330762
H	-2.46611356	-4.07350020	-1.26421616
H	-1.73492156	-5.69974905	-1.26738132
H	-2.03159210	-4.84908142	0.27166393
H	-0.37253389	-3.29211008	-3.14994432
H	0.23179414	-4.96602222	-3.03546537
H	1.33183945	-3.59056791	-2.75628867
H	2.04647297	-4.86594659	-0.22710334
H	0.90173985	-6.16310706	-0.65940638
H	0.77665844	-5.34075515	0.91798382
H	2.37106831	2.40649241	1.75693584
H	-1.86187882	2.50665105	2.29113259
H	-0.74259120	1.57717603	3.34439745
C	0.13436033	3.64054418	0.79992748
C	-1.03615856	4.41218722	0.70773776
C	1.20755610	3.91031392	-0.06878070
C	-1.13257779	5.43128231	-0.22047677
H	-1.86303743	4.24240604	1.38318259
C	1.10667230	4.90751696	-1.01597314
H	2.11171513	3.31851266	-0.02711166
C	-0.06262800	5.66770207	-1.08133623
H	-2.02641191	6.03897119	-0.28276665
H	1.92308849	5.10587278	-1.69872113
C	-0.16305781	6.72439984	-2.05821922
N	-0.24221097	7.56536344	-2.83707041

*

constrA0

C	-0.36156559	-3.97407305	1.14602058
C	-0.46680322	-4.05207196	-0.23727173
C	-0.10757835	-2.96636781	-1.02253170
C	0.37242333	-1.80481030	-0.42452255
C	0.46245461	-1.72075377	0.96201682
C	0.10343337	-2.80951444	1.74535543
H	-0.64041495	-4.82313877	1.75906802
H	-0.82335576	-4.96163854	-0.70622026
H	-0.18027370	-3.03226803	-2.10318891
H	0.82781265	-0.81332384	1.43374229
H	0.18668772	-2.74881008	2.82415856
C	0.77580322	-0.61264448	-1.28764903
H	1.77306910	-0.26087998	-1.02816055
H	0.78980820	-0.93254965	-2.33475968
H	-1.23039429	0.16279756	-1.54489709
C	-0.14521698	1.66814205	-0.53816282
C	-1.30685163	2.49092876	-0.48967935
C	1.06996303	2.14227134	0.03392570
C	-1.25473510	3.73102531	0.09967520
H	-2.22994364	2.12305113	-0.92463143
C	1.10908412	3.38059197	0.62216046
H	1.95985936	1.52690051	0.00571827
C	-0.04907593	4.16881598	0.65197384
H	-2.13324579	4.36261281	0.13906547
H	2.02640918	3.75289749	1.06084037
H	-0.00677880	5.14769511	1.11840881
C	-0.24979556	0.42906624	-1.15180180

*

constrA1

C	-0.71742178	-3.62277912	0.11442588
C	0.49849546	-3.57114921	-0.56783652
C	1.26617309	-2.41840209	-0.57281096
C	0.83693819	-1.29089305	0.12184689
C	-0.38444984	-1.32070715	0.78737704
C	-1.14634291	-2.48015007	0.78864647
H	0.85067653	-4.44892701	-1.09916451
H	2.21325300	-2.40412902	-1.10258451
H	-0.74090559	-0.44395341	1.32029177
H	-2.09146916	-2.49762434	1.32062750
C	1.68278258	-0.02193858	0.12588545
H	1.83731930	0.34315767	1.14015653
H	2.66220838	-0.25862773	-0.30215752
H	1.02147697	0.71206748	-1.80159550
C	0.39703722	2.14547091	-0.38152508
C	-0.20567988	2.92271829	-1.41069986
C	0.32591280	2.59790661	0.96644378
C	-0.84631543	4.09995768	-1.10666814
H	-0.15064544	2.57142598	-2.43547340
C	-0.31927131	3.77283892	1.25849473
H	0.77816648	2.01589432	1.75897744
C	-0.90029959	4.51827223	0.22442483
H	-1.30552118	4.69691297	-1.88451359
H	-0.38070843	4.12759309	2.27973889

H	-1.40686221	5.44698139	0.46604583
C	1.03911416	0.96927940	-0.74309872
C	-1.52991546	-4.88923646	0.14165426
H	-2.58303610	-4.68056465	0.33421230
H	-1.44914579	-5.42879359	-0.80335237
H	-1.16929112	-5.55143537	0.93376529

*

constrA10

C	-1.24894163	-3.47646796	-0.64138217
C	0.07084397	-3.66046615	-0.22398595
C	0.73343335	-2.58288720	0.35114435
C	0.09340999	-1.35764491	0.52373213
C	-1.21340426	-1.18235479	0.08609056
C	-1.88526602	-2.25403722	-0.49091366
H	1.75621413	-2.70925353	0.69341756
H	-1.71546559	-0.22743970	0.20785650
H	-2.90984123	-2.13280115	-0.82303605
C	0.83734057	-0.20276246	1.18895788
H	0.26951176	0.19604017	2.02810642
H	1.79028324	-0.58152347	1.57228722
H	1.83385154	0.49287583	-0.60374490
C	0.59118803	2.07847193	0.03043093
C	1.00210196	2.86494299	-1.08302741
C	-0.34944886	2.61557811	0.95451138
C	0.49885633	4.13109155	-1.26179455
H	1.71665285	2.44922961	-1.78531992
C	-0.84740018	3.87951924	0.76406641
H	-0.67271719	2.02771881	1.80381636
C	-0.42226381	4.63120777	-0.33903313
H	0.80785300	4.73534355	-2.10540124
H	-1.56358940	4.29929422	1.45935152
H	-0.82114922	5.63051603	-0.47969831
C	1.13386283	0.80901095	0.16889349
H	-1.78398026	-4.30648382	-1.09110776
C	0.74462018	-4.99735475	-0.38444488
H	1.78260335	-4.96351085	-0.05162315
H	0.72949548	-5.31383682	-1.42987142
H	0.22459796	-5.76128134	0.19838102

*

constrA11

C	-1.26070877	-3.57667290	-0.39806909
C	0.10356229	-3.70312566	-0.14123409
C	0.82295130	-2.61331724	0.34140908
C	0.17128692	-1.41156628	0.57716382
C	-1.18704184	-1.27039462	0.30555063
C	-1.89605125	-2.36491185	-0.17350027
H	1.87998833	-2.73721185	0.54794435
H	-1.69334505	-0.32756501	0.48342147
H	-2.95688561	-2.27454570	-0.37596311
C	0.95876819	-0.22093366	1.11346279
H	0.48950778	0.18859840	2.00643539
H	1.96432322	-0.56147392	1.38319599
H	1.67256449	0.42664407	-0.82508780
C	0.55885907	2.04158675	-0.04270200
C	0.81167563	2.80311872	-1.21932816
C	-0.22635812	2.60989909	1.00072936
C	0.30729563	4.07510091	-1.34442942
H	1.40776921	2.36363113	-2.01187929
C	-0.72699524	3.87947264	0.86323600
H	-0.42952068	2.04115790	1.89880973
C	-0.45844331	4.60602358	-0.30423615
H	0.49675988	4.66061096	-2.23523065
H	-1.32559412	4.32298525	1.64906463
H	-0.85825889	5.61006782	-0.40218411
C	1.09994206	0.76718443	0.03696120
H	-1.82349924	-4.42633176	-0.77150310
O	0.78889441	-4.85426880	-0.33586244
H	0.19664490	-5.54808413	-0.64613461

*

constrA12

C	-0.32909397	-2.94957644	0.17821064
C	0.92372591	-2.79990601	-0.42385189
C	1.58774418	-1.58675451	-0.39594416
C	1.02300389	-0.49092807	0.25264358
C	-0.23282066	-0.61935175	0.83763636
C	-0.89271592	-1.83828948	0.80763635
H	1.39372591	-3.64327330	-0.91669674
H	2.56401595	-1.49883251	-0.86190648
H	-0.69553763	0.22765744	1.33594540
H	-1.86373595	-1.93205712	1.28139662
C	1.75700819	0.84378229	0.29305032
H	1.82016390	1.22772204	1.31022400
H	2.77718407	0.68820113	-0.07280813
H	1.15308066	1.50588236	-1.67832523
C	0.32891971	2.89316348	-0.31451833
C	-0.27202127	3.61145731	-1.38614934
C	0.14286495	3.34860191	1.02099790
C	-1.02023908	4.73656145	-1.13439660
H	-0.12943542	3.25772160	-2.40158747
C	-0.60880341	4.47114133	1.26065831
H	0.59178167	2.81032845	1.84566726
C	-1.18495243	5.16009592	0.18579373
H	-1.47862632	5.28883515	-1.94504878
H	-0.75759031	4.82770973	2.27223576
H	-1.77620170	6.04766829	0.38598782
C	1.08672329	1.77136296	-0.62385235
C	-1.06167856	-4.23875919	0.16817466
C	-0.96438205	-5.16778856	-1.01935512
C	-0.31071553	-5.54607905	0.26807063
H	-2.02265439	-4.20334213	0.66745372
H	-0.33473634	-4.85371941	-1.84307090
H	-1.86528196	-5.69104413	-1.31308086
H	-0.75999318	-6.33041613	0.86350675
H	0.76929510	-5.49140401	0.33237671

*

constrA13

C	-1.25132932	-3.23789513	-0.40174380
C	0.05893987	-3.35656402	0.05495130
C	0.70718708	-2.26000251	0.60568417
C	0.04589968	-1.04060656	0.71442816
C	-1.25888538	-0.92137013	0.24540495
C	-1.90887296	-2.02142004	-0.30472595
H	1.72614348	-2.36731757	0.96782655
H	-1.78095955	0.02806613	0.32064121
H	-2.92928447	-1.92412331	-0.65600954
C	0.75657548	0.16315917	1.32591352
H	0.15489285	0.61459369	2.11308495
H	1.69732821	-0.17718448	1.77106852
H	1.80136506	0.73330129	-0.48630482
C	0.57008740	2.37048996	0.01871542
C	1.01952939	3.07935589	-1.13360162
C	-0.38093903	2.98190611	0.88696691
C	0.54366209	4.33949227	-1.40242108
H	1.74160627	2.60817540	-1.79182571
C	-0.85026377	4.23909883	0.60666190
H	-0.73213079	2.45550315	1.76506287
C	-0.38737344	4.91197683	-0.53262835
H	0.88132868	4.88451335	-2.27487501
H	-1.57255379	4.71528396	1.25787237
H	-0.76455189	5.90727361	-0.74427253
C	1.08798772	1.10668511	0.24766786
H	-1.73611957	-4.10944391	-0.82618845
C	0.77030343	-4.66757477	-0.04273136
O	0.27848652	-5.65080579	-0.52806560
H	1.80052240	-4.67946222	0.36629683

*

constrA14
C -0.10820510 -3.08341301 0.07255423
C 1.20709428 -2.91146826 -0.31797792
C 1.78733842 -1.66371942 -0.14802952
C 1.05533005 -0.62110288 0.41338453
C -0.27106056 -0.81836179 0.78759678
C -0.86210834 -2.06239246 0.62409153
H 1.75628752 -3.74280023 -0.73904302
H 2.82135636 -1.51198664 -0.43768197
H -0.84649470 -0.00771650 1.22292837
H -1.88682131 -2.25001855 0.91491696
C 1.70206154 0.74753053 0.60282950
H 1.54519804 1.11481197 1.61547326
H 2.78054436 0.65175273 0.43807955
H 1.41363798 1.38168783 -1.45171937
C 0.38145144 2.78083032 -0.25894124
C -0.04191351 3.48061516 -1.42807899
C -0.02222957 3.25733876 1.02377277
C -0.82794588 4.60122929 -1.31942294
H 0.26659936 3.11294909 -2.40088521
C -0.80828668 4.37574986 1.11871146
H 0.29105173 2.73770562 1.92005562
C -1.20683329 5.04216461 -0.04902304
H -1.15287054 5.13787605 -2.20182907
H -1.12194356 4.74844430 2.08578229
H -1.82876678 5.92725961 0.03819040
C 1.17442452 1.66022241 -0.42588538
N -0.73869116 -4.41543782 -0.10804940
O -0.05167881 -5.28862064 -0.59113820
O -1.89454505 -4.52898721 0.23899197
*

constrA15
C -0.52355645 -3.26353242 -0.11706529
C 0.86167578 -3.19155300 -0.04709208
C 1.49991996 -2.01880570 0.31373951
C 0.73780684 -0.89782326 0.62281728
C -0.65044551 -0.94295443 0.54376470
C -1.26595978 -2.12803710 0.18237843
H 2.58195536 -2.01289572 0.36835562
H -1.27254297 -0.08545183 0.77339685
C 1.42878924 0.39812646 1.02812935
H 1.02828680 0.77758419 1.96667644
H 2.49518686 0.19657216 1.17257300
H 1.80394897 1.06817632 -1.00148415
C 0.59208600 2.54754297 -0.11165217
C 0.58702984 3.29204359 -1.32872221
C -0.12669867 3.04732199 1.01473333
C -0.10038554 4.47759784 -1.41388389
H 1.13376084 2.90642155 -2.18259218
C -0.81109217 4.23046382 0.91606780
H -0.13331710 2.49390383 1.94491943
C -0.79535072 4.93975610 -0.29343656
H -0.10832660 5.04855609 -2.33375290
H -1.36153242 4.62090755 1.76287206
H -1.34039768 5.87583225 -0.36010721
C 1.30274914 1.36145605 -0.07968327
F -2.59371980 -2.19563370 0.11284416
F -1.12593389 -4.39146233 -0.46086293
F 1.57190414 -4.27868129 -0.33182290
*

constrA16
C -0.09917660 -3.50952441 -0.29591452
C 1.26644308 -3.27953744 -0.35103460
C 1.78556931 -2.06150111 0.06447982
C 0.94408090 -1.07011672 0.55108716
C -0.43731444 -1.28473034 0.58385055
C -0.95331361 -2.49985712 0.17831829
H 1.91169791 -4.07222178 -0.70960241
H 2.85769670 -1.90076109 0.03298870
H -1.10977512 -0.51233381 0.94826022
C 1.51436303 0.26335142 1.01408884
H 1.06249184 0.58297477 1.95194700
H 2.58862794 0.13789848 1.18487101
H 1.90586088 1.02899397 -0.97181368
C 0.51040858 2.35516330 -0.10110235
C 0.47567173 3.12480805 -1.29757377
C -0.28272828 2.75786573 1.00981073
C -0.31342705 4.24766891 -1.37859366
H 1.08098688 2.81208832 -2.14161629
C -1.06751533 3.87939248 0.91728718
H -0.26417458 2.18313192 1.92677608
C -1.07992169 4.61896450 -0.27263703
H -0.34300855 4.83843743 -2.28547447
H -1.67306055 4.19809336 1.75657037
H -1.70256250 5.50546259 -0.33480715
C 1.34205905 1.24314668 -0.06457165
O -0.59244148 -4.69787644 -0.69398945
H -1.55101122 -4.70143143 -0.56948385
O -2.27985698 -2.82524250 0.18858317
H -2.80055254 -2.14564930 0.62694195
*

constrA17
C 0.01809837 -2.74511685 0.84955518
C 0.04495362 -2.78322088 -0.57342757
C 0.30878063 -1.62505100 -1.27871432
C 0.57185205 -0.42589007 -0.60076600
C 0.51933266 -0.38416532 0.77971627
C 0.25564753 -1.54809838 1.50476587
H 0.34576459 -1.64605615 -2.35989893
H 0.70431804 0.54196055 1.31396339
H 0.23805664 -1.50644649 2.58491360
C 0.87335188 0.83684810 -1.39041548
H 1.84775063 1.24801019 -1.12917226
H 0.89688689 0.58392805 -2.45561768
H -1.18380011 1.48329869 -1.58532116
C -0.19257948 2.98884844 -0.47672472
C -1.39904774 3.73458143 -0.37479664
C 0.99182730 3.49486712 0.12572573
C -1.41810929 4.93694060 0.29164867
H -2.30060729 3.34143834 -0.83217360
C 0.96018958 4.69443617 0.79293524
H 1.91521842 2.93378121 0.06325476
C -0.24001097 5.41064067 0.87202998
H -2.33289813 5.51088638 0.36982264
H 1.85583096 5.08898872 1.25639639
H -0.25443082 6.35835758 1.40010889
C -0.22011980 1.78874964 -1.18018800
O -0.24129402 -3.91694733 1.44784297
C -0.26405963 -3.94759700 2.87194103
H 0.70625457 -3.65493791 3.28341747
H -1.04984841 -3.29525063 3.26386569
H -0.47864300 -4.97924186 3.14063492
O -0.18814967 -3.98804426 -1.12064188
C -0.16616037 -4.08846550 -2.53950655
H -0.93755269 -3.45521129 -2.98872954
H 0.81763914 -3.81846193 -2.93543107
H -0.37521078 -5.13144047 -2.76564633
*

constrA18
C -0.09310080 -2.93847193 0.05524684
C 1.24458398 -2.72278931 -0.35265028
C 1.83818159 -1.48909417 -0.20408535
C 1.15223235 -0.41962944 0.37472444
C -0.17947261 -0.61110223 0.74528805
C -0.79150133 -1.83889909 0.60705497
H 1.82332446 -3.53125938 -0.77597519
H 2.86990413 -1.36272836 -0.51738196
H -0.74905445 0.20989133 1.17149566

H	-1.81708528	-1.94980157	0.92872536
C	1.82051776	0.93206106	0.54616655
H	1.69502630	1.31128602	1.56015218
H	2.89397849	0.81246854	0.37017543
H	1.58381115	1.62884475	-1.49090263
C	0.38428741	2.89235473	-0.28830979
C	-0.04732040	3.60754755	-1.43740165
C	-0.09790496	3.27684496	0.99162932
C	-0.91760930	4.66531753	-1.31186716
H	0.32107892	3.30808883	-2.41266073
C	-0.97021156	4.33161693	1.10539733
H	0.21973135	2.73871113	1.87531880
C	-1.37535743	5.02186048	-0.04267312
H	-1.24719689	5.21541050	-2.18432420
H	-1.34369993	4.63266638	2.07626491
H	-2.06307674	5.85497035	0.05759401
C	1.28733241	1.84539818	-0.46557902
N	-0.68309815	-4.15515232	-0.07805903
C	-2.05480857	-4.35384373	0.35866574
C	0.05066085	-5.26492741	-0.66454472
H	-2.33256302	-5.39214004	0.19369154
H	-2.16818160	-4.13820235	1.42575514
H	-2.75108461	-3.72210006	-0.20265683
H	-0.59307809	-6.14092271	-0.68935959
H	0.36065057	-5.04234590	-1.69042471
H	0.93920054	-5.51320276	-0.07544851

*

constrA19

C	-0.24016560	-2.52017951	0.03251942
C	1.14606392	-2.42864430	0.04903939
C	1.73923036	-1.20275666	0.34537046
C	0.95873957	-0.09555671	0.65009256
C	-0.42498184	-0.20411343	0.63458689
C	-1.03573426	-1.41570176	0.31686384
H	2.82115203	-1.13918832	0.34707328
H	-1.05647278	0.64400776	0.87459106
C	1.61740294	1.23650053	0.98937937
H	1.24525089	1.62001128	1.93776683
H	2.69726790	1.08415171	1.08572857
H	1.85122485	1.86935518	-1.07916060
C	0.65403275	3.33230419	-0.15214457
C	0.55166627	4.04376023	-1.38864536
C	-0.01397686	3.84358107	1.00419351
C	-0.18042503	5.20143724	-1.46380780
H	1.05991020	3.65209827	-2.26337374
C	-0.74232926	4.99892548	0.91446221
H	0.05495873	3.32020456	1.94924636
C	-0.82331206	5.67185428	-0.31493778
H	-0.26392337	5.74537024	-2.39621080
H	-1.25447895	5.39718091	1.78140732
H	-1.40434733	6.58674215	-0.37397088
C	1.39509940	2.17052969	-0.13666396
N	-0.88209330	-3.80954026	-0.29804099
O	-0.50211361	-4.77019657	0.32838202
O	-1.72527643	-3.77507799	-1.16451595
C	-2.47478295	-1.47350270	0.32295853
N	-3.62111321	-1.43155391	0.37026866
C	1.99780889	-3.54592220	-0.26690927
N	2.73900823	-4.37971901	-0.53756300

*

constrA2

C	-0.11760374	-3.40335566	0.17276111
C	1.13238657	-3.23172554	-0.40700418
C	1.73801240	-1.98682209	-0.35405099
C	1.10759391	-0.92275541	0.28503243
C	-0.15254176	-1.10578185	0.84783688
C	-0.76610810	-2.34821942	0.80169439
H	1.62395817	-4.06828471	-0.88712149
H	2.71931609	-1.85380134	-0.79723011
H	-0.65876178	-0.28223339	1.34216775
H	-1.73951898	-2.50451284	1.24887277
C	1.78204937	0.44278267	0.34683646
H	1.79119523	0.83057890	1.36422509
H	2.82062857	0.33325681	0.01734926
H	1.19936678	1.05728627	-1.64801681
C	0.32300378	2.45121963	-0.32750837
C	-0.27152480	3.13663067	-1.42624898
C	0.10124691	2.93044475	0.99596893
C	-1.04724355	4.25004110	-1.21197413
H	-0.10136035	2.76568211	-2.43127556
C	-0.67689056	4.04135215	1.19741043
H	0.54533179	2.41928613	1.84033120
C	-1.24589147	4.69602415	0.09654972
H	-1.50108625	4.77674267	-2.04194600
H	-0.85292329	4.41618485	2.19793543
H	-1.85893639	5.57503215	0.26722479
C	1.10547060	1.34039149	-0.60014790
Cl	-0.87832682	-4.95281699	0.11241648

*

constrA20

C	-0.32542333	-1.93125436	0.03556254
C	1.06169891	-1.94626662	0.10722431
C	1.72644137	-0.77928519	0.45479786
C	1.01945143	0.37982920	0.74337935
C	-0.36556192	0.37691210	0.66340379
C	-1.05019947	-0.77688999	0.30999201
H	2.80816519	-0.79069721	0.50840307
H	-0.93365485	1.27129637	0.89009198
C	1.76067718	1.65357950	1.13349157
H	1.34088023	2.08093785	2.04238765
H	2.80943646	1.40675532	1.32708822
H	2.25015888	2.24584059	-0.89998409
C	1.06268286	3.79569504	-0.10929148
C	1.13235128	4.49527125	-1.35320739
C	0.31623606	4.36257011	0.96900318
C	0.48824936	5.69682234	-1.51016644
H	1.69968379	4.06042336	-2.16919942
C	-0.32323660	5.56132997	0.79828963
H	0.25544038	3.84754661	1.91913323
C	-0.23561503	6.22242759	-0.43632075
H	0.53523916	6.23258855	-2.44981454
H	-0.89345154	6.00217237	1.60638226
H	-0.74676903	7.17187401	-0.55981251
C	1.72190602	2.58748271	-0.01041191
C	1.88620650	-3.19855671	-0.16864837
F	3.15049750	-2.85989899	-0.47923712
F	1.39134722	-3.89579501	-1.19593098
F	1.93184095	-3.99006370	0.90497272
C	-2.56989265	-0.71632165	0.22153052
F	-3.03514809	0.31015063	0.95996363
F	-3.14075085	-1.83242698	0.67767627
F	-2.96957275	-0.51280639	-1.03843963
N	-1.04985133	-3.16761926	-0.34808846
O	-1.58457177	-3.15162114	-1.43155564
O	-1.03474143	-4.06590080	0.45802290

*

constrA21

C	-0.15372640	-2.76327823	0.02663951
C	1.21609022	-2.58474449	-0.07481750
C	1.79510456	-1.35192086	0.17138574
C	0.98260223	-0.28933108	0.54777814
C	-0.39548906	-0.44574012	0.65173558
C	-0.94844565	-1.68685539	0.38804032
H	2.87023984	-1.25428349	0.08328109
H	-1.05148846	0.36407008	0.94881490
C	1.60730233	1.07072892	0.83427566
H	1.31226509	1.43420878	1.81704873
H	2.69758327	0.96812288	0.82317215

H	1.59534142	1.70217708	-1.24338821
C	0.47233376	3.14145022	-0.19022637
C	0.22408696	3.85030508	-1.40502512
C	-0.07399202	3.63858915	1.03191865
C	-0.52975466	4.99726877	-1.39696855
H	0.63975949	3.46716349	-2.33085293
C	-0.82563729	4.78361555	1.02582207
H	0.10435089	3.11312148	1.96126361
C	-1.05005318	5.45717654	-0.18418215
H	-0.72268214	5.54040582	-2.31347146
H	-1.24623675	5.17202271	1.94491617
H	-1.64731042	6.36335357	-0.17680659
C	1.23847256	1.99474231	-0.25642269
N	-0.75967485	-4.07732190	-0.24952788
O	-0.44892232	-4.97744342	0.49439366
O	-1.51068171	-4.12876093	-1.19641245
F	-2.26078749	-1.85541326	0.50025176
F	1.97974328	-3.60836439	-0.43211513

*

constrA22

C	0.28957400	-2.24359589	0.08186114
C	1.52069104	-1.93048371	-0.54298805
C	2.02067777	-0.64888789	-0.50878931
C	1.34695728	0.37405725	0.16422801
C	0.11232721	0.08267542	0.74937961
C	-0.40572289	-1.19396306	0.72767674
H	2.09102145	-2.70744681	-1.03351751
H	2.97472764	-0.44389593	-0.98466995
H	-0.45010043	0.86611902	1.24940251
H	-1.35781887	-1.38788976	1.20250052
C	1.91475758	1.77970058	0.20845804
H	1.92563362	2.17316248	1.22489114
H	2.95129656	1.74497435	-0.14078566
H	1.29022105	2.40571538	-1.76850345
C	0.19357514	3.58883592	-0.39604553
C	-0.47996596	4.23849922	-1.46429288
C	-0.10508066	3.96051239	0.94186098
C	-1.40613512	5.22269332	-1.20681728
H	-0.25123627	3.94860519	-2.48417967
C	-1.03500144	4.94128774	1.18827914
H	0.39726117	3.47117889	1.76621711
C	-1.67995347	5.56908847	0.11692309
H	-1.91964211	5.72302365	-2.01824836
H	-1.26943863	5.23193856	2.20480332
H	-2.41117691	6.34397244	0.32157632
C	1.14658330	2.61976321	-0.71044164
N	-0.20265008	-3.50072710	0.06134034
C	-1.40396972	-3.91597216	0.78473702
C	0.42310201	-4.60759713	-0.66249096
H	-1.38455065	-3.54312208	1.81186953
H	-2.30426393	-3.52564933	0.29438944
C	-1.34761859	-5.44212698	0.72143077
H	0.65960648	-4.31515811	-1.68850311
H	1.35369944	-4.91310376	-0.16882442
C	-0.63184659	-5.71170463	-0.60374920
H	-0.75254156	-5.82438437	1.55473331
H	-2.33779466	-5.89439956	0.77513118
H	-1.32972056	-5.60132772	-1.43778893
H	-0.19065004	-6.70672515	-0.65933168

*

constrA23

C	0.06650083	-2.79960139	-0.09663819
C	1.43312858	-2.54234649	-0.32457539
C	1.99307318	-1.29905166	-0.09714922
C	1.22732440	-0.24963748	0.39948625
C	-0.13999946	-0.45749265	0.59339147
C	-0.68868361	-1.69166598	0.35166163
H	2.07302814	-3.34693472	-0.65868258
H	3.05542021	-1.16187231	-0.26968547
H	-0.80204442	0.33511399	0.92601008
C	1.84694867	1.11148179	0.66430757
H	1.60068489	1.47096486	1.66308338
H	2.93474029	1.01057248	0.60560244
H	1.83256897	1.84391432	-1.37616423
C	0.46917536	3.05218603	-0.30138333
C	0.14883126	3.77432471	-1.48393556
C	-0.17252028	3.39742331	0.92013805
C	-0.76850427	4.79765326	-1.44614390
H	0.63872566	3.50510232	-2.41347726
C	-1.09004090	4.41808256	0.94543323
H	0.06038567	2.85576226	1.82781403
C	-1.38382260	5.11371952	-0.23358918
H	-1.01536713	5.35179922	-2.34297651
H	-1.58547378	4.68868425	1.86951813
H	-2.10953089	5.91971237	-0.20289584
C	1.41222046	2.03352336	-0.38949255
N	-0.49585579	-4.02105742	-0.32863809
C	-1.62705722	-4.50687538	0.46490056
C	0.27282037	-5.03783625	-1.02673435
H	-1.48667490	-5.57360070	0.64369087
H	-1.66554118	-4.01068487	1.43335578
H	-2.57957875	-4.35776745	-0.04773752
H	-0.41202196	-5.81153670	-1.37221759
H	0.76116717	-4.61139627	-1.90414662
H	1.03154619	-5.50578712	-0.38606245
F	-2.02405565	-1.81901128	0.50363971

*

constrA24

C	-0.68690439	-2.34245048	-0.09275024
C	0.68745643	-2.46152544	0.07924581
C	1.44283560	-1.37749425	0.49048069
C	0.82088487	-0.15753023	0.74611440
C	-0.54790516	-0.02772761	0.56380087
C	-1.29674372	-1.12280228	0.14917592
H	2.51169575	-1.48997595	0.62826229
H	-1.04473195	0.91921553	0.74706041
C	1.64698445	1.03956137	1.20571804
H	1.21187024	1.49150970	2.09541869
H	2.65468389	0.69450701	1.45810346
H	2.32782287	1.61698870	-0.77377836
C	1.20309438	3.24351378	-0.04379845
C	1.41565605	3.95110926	-1.26492530
C	0.42347613	3.84905156	0.98694302
C	0.87586178	5.20039745	-1.44555452
H	2.00763958	3.48541968	-2.04556097
C	-0.11189429	5.09520324	0.79314096
H	0.25433838	3.32615874	1.91943594
C	0.11548667	5.76472334	-0.41806312
H	1.03211037	5.74348947	-2.36903948
H	-0.70656880	5.56588257	1.56599548
H	-0.31318904	6.75153148	-0.56043373
C	1.76366690	1.98655950	0.08191402
C	1.34420795	-3.79318862	-0.21697021
F	2.62299593	-3.81794003	0.19047812
F	1.33195473	-4.04379283	-1.53740781
F	0.69661620	-4.79672918	0.39135785
H	-1.27407497	-3.19371366	-0.41518844
C	-2.79451295	-0.96868660	0.00263689
F	-3.10457086	0.23885423	-0.51145981
F	-3.40500806	-1.05924456	1.19634370
F	-3.31708412	-1.90259099	-0.79873733

*

constrA3

C	-0.97649085	-2.52823178	1.32409246
C	-0.01062303	-2.67811895	0.33540454
C	0.78905053	-1.60957811	-0.03415269
C	0.62678564	-0.37868071	0.59462012
C	-0.35041781	-0.21942047	1.57051738

C	-1.14496507	-1.29747542	1.94073480
H	1.54631454	-1.74370239	-0.79770874
H	-0.48506337	0.74122426	2.05832599
H	-1.89655363	-1.17686381	2.71155109
C	1.50698643	0.80142557	0.19413886
H	1.87487088	1.32899751	1.07219116
H	2.37137434	0.42090078	-0.36068463
H	0.45578713	1.20339401	-1.65867217
C	0.29499569	2.96152246	-0.50421216
C	-0.47814042	3.57785176	-1.53143270
C	0.58326245	3.69219275	0.68549065
C	-0.93862582	4.86267837	-1.37709542
H	-0.69571961	3.01656123	-2.43382332
C	0.11676155	4.97320073	0.82837934
H	1.16966370	3.23804968	1.47369783
C	-0.63961721	5.55260613	-0.20000971
H	-1.52562412	5.33756478	-2.15307436
H	0.32916250	5.53979200	1.72637050
H	-1.00297812	6.56772063	-0.07665512
C	0.74081358	1.66785691	-0.71512937
C	0.16829396	-4.03394799	-0.31031462
F	1.00222368	-3.98081343	-1.36293186
F	-1.00656803	-4.51942290	-0.74502435
F	0.67156652	-4.92145923	0.56302358
H	-1.59476342	-3.37194046	1.60770780

*

constrA4

C	0.12206225	-2.74407297	0.13531800
C	1.36242062	-2.49593076	-0.43520077
C	1.90936512	-1.22368808	-0.35948613
C	1.22190341	-0.20613971	0.29344412
C	-0.02763870	-0.45790283	0.85237155
C	-0.57536194	-1.72964971	0.77990426
H	1.90405893	-3.29433560	-0.92679828
H	2.88518513	-1.03305765	-0.79316109
H	-0.57143124	0.33145408	1.36182228
H	-1.53970429	-1.93569134	1.22795352
C	1.82752480	1.19135888	0.38051543
H	1.79701682	1.56715526	1.40177271
H	2.87595730	1.13727125	0.06858499
H	1.23935055	1.78698830	-1.61931937
C	0.30236219	3.14637860	-0.30668409
C	-0.30655151	3.81020615	-1.41207265
C	0.04853373	3.61629371	1.01536442
C	-1.12662678	4.89238748	-1.20550461
H	-0.11166432	3.44671074	-2.41534613
C	-0.77345033	4.69602234	1.20867896
H	0.50334147	3.12232931	1.86424953
C	-1.35569484	5.32895337	0.10147116
H	-1.59193601	5.40211688	-2.03969792
H	-0.97454428	5.06354508	2.20718187
H	-2.00396262	6.18355987	0.26613735
C	1.12456508	2.06499923	-0.57210174
C	-0.50565804	-4.11555314	0.01782187
F	-1.23201077	-4.41846898	1.10548548
F	0.41902005	-5.07345738	-0.14074536
F	-1.33134367	-4.16931835	-1.04446368

*

constrA5

C	-0.61155404	-3.68267862	-0.03853808
C	0.73282536	-3.60646616	-0.40773090
C	1.43093678	-2.43262844	-0.20959666
C	0.81263903	-1.32420067	0.37140105
C	-0.53385382	-1.40067989	0.71480818
C	-1.24476054	-2.57468527	0.52380386
H	1.20917867	-4.47918026	-0.83724143
H	2.47866470	-2.38540669	-0.48837961
H	-1.03677102	-0.54458374	1.15464623
H	-2.28954340	-2.63193529	0.81152127
C	1.59096893	-0.03527874	0.59543528
H	1.46558128	0.33154893	1.61316567
H	2.65544455	-0.24235404	0.44347535
H	1.43284640	0.68139074	-1.44193235
C	0.44031948	2.10781642	-0.23981939
C	0.10997257	2.87193315	-1.39420258
C	0.02545082	2.56689976	1.04193247
C	-0.59779111	4.04391168	-1.27218968
H	0.42627300	2.51583129	-2.36875648
C	-0.68299234	3.73680848	1.15143447
H	0.26780873	1.99501569	1.92828152
C	-0.99041580	4.46994888	-0.00190495
H	-0.85037094	4.63103215	-2.14614025
H	-1.00332135	4.09700884	2.12109443
H	-1.54960988	5.39470762	0.09588767
C	1.17428798	0.94193777	-0.41623905
O	-1.24150995	-4.85845263	-0.25253321
H	-2.15506530	-4.81263979	0.05045272

*

constrA6

C	-0.07547596	-3.33982186	-0.07121024
C	1.26608815	-3.12449152	-0.41560532
C	1.84366059	-1.89181234	-0.21142623
C	1.10774652	-0.84727096	0.35666390
C	-0.22836063	-1.05702101	0.67283074
C	-0.82422065	-2.29627751	0.47604641
H	1.83159399	-3.94853690	-0.83329394
H	2.88651841	-1.74280790	-0.47202874
H	-0.82146073	-0.25300751	1.09890738
H	-1.86053433	-2.43681879	0.74995319
C	1.75273366	0.51144458	0.58990286
H	1.59044224	0.85738986	1.60982412
H	2.83254430	0.41182443	0.43901204
H	1.53553624	1.23113855	-1.44096828
C	0.37757591	2.52193301	-0.23225078
C	-0.02580804	3.25973929	-1.37987389
C	-0.09948492	2.91209876	1.05011458
C	-0.86352311	4.34199497	-1.25060966
H	0.33875098	2.95591760	-2.35526068
C	-0.93815375	3.99201181	1.16693468
H	0.19719866	2.35821305	1.93135311
C	-1.31530723	4.70222213	0.02025741
H	-1.17225971	4.90912245	-2.11971568
H	-1.30752856	4.29885311	2.13750349
H	-1.97727874	5.55572859	0.12363120
C	1.24071057	1.44808318	-0.41496635
O	-0.54563752	-4.57733331	-0.30417851
C	-1.89999164	-4.86058317	0.04024230
H	-2.06628129	-4.72207983	1.11221406
H	-2.58768438	-4.23089608	-0.53135941
H	-2.05916122	-5.90404363	-0.22130787

*

constrA7

C	-0.18255050	-3.39846472	-0.04066267
C	1.09733244	-3.18839778	-0.55825699
C	1.72240412	-1.95801564	-0.44158503
C	1.07898750	-0.90834767	0.20594858
C	-0.21207460	-1.09371796	0.69642745
C	-0.83140891	-2.32610311	0.57910628
H	1.61396439	-4.00703257	-1.04735948
H	2.72390494	-1.82306742	-0.83706855
H	-0.73632647	-0.27527587	1.18112866
H	-1.83767243	-2.45229483	0.95960585
C	1.76517989	0.44611038	0.34008831
H	1.72891355	0.80401059	1.36799788
H	2.81683809	0.33028149	0.05873072
H	1.30817453	1.14180300	-1.65971447
C	0.33127013	2.46932678	-0.33924378
C	-0.19973797	3.19664672	-1.44203151

C	0.01880348	2.88556272	0.98601777
C	-1.00208068	4.29192039	-1.22953778
H	0.03932810	2.87293870	-2.44932992
C	-0.78597900	3.97830762	1.18589575
H	0.41413689	2.34088497	1.83361266
C	-1.29108362	4.67653036	0.08129190
H	-1.40791791	4.85069726	-2.06339984
H	-1.03109054	4.30505168	2.18875124
H	-1.92524961	5.54064837	0.25050342
C	1.14520604	1.37819535	-0.60879584
C	-0.80200850	-4.73202592	-0.16739825
C	-1.82196320	-5.17939784	0.55641481
H	-0.34725469	-5.38820056	-0.90456674
H	-2.21316579	-6.17825010	0.40564738
H	-2.29243506	-4.58279421	1.33053267

*

constrA8

C	-0.28435169	-3.48025088	0.10874885
C	0.96145038	-3.31898593	-0.50235012
C	1.60602532	-2.09491212	-0.45684383
C	1.02391419	-1.02078433	0.21061394
C	-0.22537546	-1.17162615	0.80645195
C	-0.87262729	-2.39564794	0.76458598
H	1.42015128	-4.16115532	-1.00563603
H	2.57819343	-1.98352847	-0.92548574
H	-0.69212551	-0.33752627	1.32163764
H	-1.83784327	-2.52209357	1.23925447
C	1.73940515	0.32429820	0.26632415
H	1.78750121	0.70006604	1.28716578
H	2.76459028	0.18779384	-0.09337975
H	1.12431830	0.98136676	-1.70483959
C	0.31175003	2.37482068	-0.34309499
C	-0.29379745	3.08947868	-1.41642722
C	0.13268757	2.83808593	0.99211154
C	-1.03959936	4.21600472	-1.16666010
H	-0.15630819	2.73041828	-2.43072143
C	-0.61599768	3.96231550	1.22918969
H	0.58560982	2.30436281	1.81758561
C	-1.19693197	4.64600229	0.15269264
H	-1.50172830	4.76491248	-1.97745340
H	-0.75953231	4.32510324	2.23930017
H	-1.78633117	5.53522547	0.35135416
C	1.06241496	1.25073154	-0.65099282
C	-0.95471799	-4.74926612	0.06461538
C	-1.51427449	-5.80801904	0.02625998
H	-2.01003262	-6.75212629	-0.00580683

*

constrA9

C	-0.27889413	-3.51477903	0.05122830
C	1.01597830	-3.34340558	-0.43429958
C	1.63914242	-2.11393145	-0.29878692
C	0.98037132	-1.05995213	0.32777867
C	-0.31763362	-1.23175399	0.80032673
C	-0.94736755	-2.45921552	0.67034414
H	1.52778967	-4.17195035	-0.90811148
H	2.65125387	-1.98407447	-0.66649834
H	-0.83849633	-0.41300706	1.28657369
H	-1.95266492	-2.60646441	1.04531263
C	1.67302628	0.29031725	0.47989451
H	1.59732675	0.65418780	1.50308384
H	2.73470814	0.16864166	0.23975515
H	1.25772601	0.94538495	-1.54558550
C	0.34960050	2.36366961	-0.27641219
C	-0.13508169	3.08249114	-1.40931538
C	0.05201563	2.84324944	1.03358898
C	-0.87805926	4.22491841	-1.24026350
H	0.09269664	2.71215519	-2.40312586
C	-0.69194589	3.98363497	1.18905382
H	0.41284456	2.30892559	1.90294774
C	-1.15249588	4.66886330	0.05563266
H	-1.24877224	4.77635463	-2.09507482
H	-0.92478571	4.35922872	2.17759710
H	-1.74001918	5.57128414	0.19060895
C	1.09902851	1.22338261	-0.50411685
C	-0.93520895	-4.79069521	-0.08857365
N	-1.46256226	-5.80490715	-0.20374663

*

constrB1

C	-0.34082153	-3.78370778	0.08206382
C	0.26839834	-3.28563486	1.24601058
C	0.78352177	-2.01714721	1.26334677
C	0.71270955	-1.18645361	0.10548944
C	0.08921133	-1.70591844	-1.06969335
C	-0.42217301	-2.97150152	-1.07074650
H	0.32380430	-3.91219878	2.12803868
H	1.25234309	-1.62601101	2.16010335
H	0.01940360	-1.09488779	-1.96045366
H	-0.89789316	-3.36751273	-1.96057852
C	1.25270981	0.08128037	0.17357328
H	1.69227963	0.37356520	1.12640849
H	2.28596111	1.50001724	-0.95414819
H	0.94433770	0.76142956	-1.83528053
C	0.36968074	2.27610495	-0.39317403
C	-1.01382969	2.12338509	-0.38345818
C	0.93952086	3.46818731	0.04352193
C	-1.82368841	3.17071580	0.03478575
H	-1.46395916	1.19268691	-0.71628144
C	0.12842915	4.51316743	0.46257727
H	2.01787294	3.58955595	0.03721109
C	-1.25281894	4.36521826	0.45800141
H	-2.90125279	3.05491541	0.02947030
H	0.57550347	5.44563014	0.78675229
H	-1.88649523	5.18230257	0.78260726
C	1.26006974	1.12964932	-0.86027848
C	-0.88937459	-5.17173199	0.04365243
H	-1.87654866	-5.18262513	-0.42341472
H	-0.23515376	-5.79651341	-0.57391315
H	-0.95113340	-5.61408707	1.03680534

*

constrB10

C	-0.76722615	-3.74966841	-0.33343122
C	0.16122733	-3.46483236	0.68004092
C	0.76104580	-2.22581720	0.64900804
C	0.45509210	-1.27665747	-0.36966995
C	-0.49278470	-1.61070336	-1.37619454
C	-1.09492608	-2.84308713	-1.34684693
H	1.48474204	-1.95265883	1.41047815
H	-0.73750916	-0.90127711	-2.15566873
H	-1.81992005	-3.12045037	-2.10198788
C	1.10581021	-0.05400625	-0.33660006
H	1.80358080	0.10378591	0.48507262
H	1.92802127	1.42283674	-1.55389497
H	0.35162113	0.85943936	-2.12087555
C	0.28709818	2.23250374	-0.44196695
C	-1.05307580	2.14884612	-0.07521768
C	1.04295860	3.33986430	-0.06860376
C	-1.64170771	3.18577959	0.63626072
H	-1.64490163	1.28278399	-0.35652777
C	0.45270314	4.37396972	0.64340384
H	2.08821854	3.40586758	-0.35241899
C	-0.88902392	4.29741046	0.99595226
H	-2.68864443	3.12612871	0.90985607
H	1.03945609	5.24280001	0.91773384
H	-1.35043640	5.10629368	1.55033005
C	0.93317241	1.09819704	-1.23185285
H	-1.25414509	-4.72090756	-0.32912565
C	0.46878101	-4.48401212	1.74315778
H	1.20917916	-4.10820629	2.44922257

H -0.43508476 -4.74237796 2.29913062
H 0.85733177 -5.40063523 1.29410768
*

constrB11
C -0.08505135 -3.75536828 -0.93102657
C -0.63263255 -3.47823949 0.32705532
C -0.34845571 -2.26276884 0.91954783
C 0.48668940 -1.32501678 0.25893341
C 1.03158927 -1.63042705 -1.01734261
C 0.73636215 -2.84102481 -1.59503852
H -0.76900396 -2.03335232 1.89215271
H 1.66869724 -0.92211302 -1.52878612
H 1.13787176 -3.09903354 -2.56709818
C 0.74249173 -0.12215507 0.90771637
H 0.26551722 0.01900608 1.87698064
H 2.20658300 1.32108124 1.22787936
H 2.11373784 0.79154208 -0.45683327
C 0.56471830 2.18190923 0.15831323
C -0.29104178 2.13549585 -0.93850246
C 0.53094359 3.27146850 1.02384775
C -1.15420233 3.19448486 -1.18646733
H -0.27591815 1.28216404 -1.61000859
C -0.33344590 4.32717990 0.77408384
H 1.19524370 3.30687879 1.88106219
C -1.17595042 4.28899777 -0.33027864
H -1.81080238 3.16476877 -2.04808727
H -0.34510265 5.18272117 1.43910471
H -1.85014454 5.11468429 -0.52574437
C 1.52183105 1.02331895 0.42696730
H -0.30430175 -4.70805840 -1.40552300
O -1.42883371 -4.33315626 0.99833673
H -1.56675948 -5.15057806 0.50591428
*

constrB12
C -0.03835276 -3.07271736 -0.26605773
C 0.29256758 -2.61238081 1.03022129
C 0.71466639 -1.33043928 1.21643017
C 0.83683579 -0.42376815 0.11501972
C 0.49775027 -0.90173802 -1.19018975
C 0.07437439 -2.18410241 -1.36526913
H 0.20955217 -3.27922040 1.87887761
H 0.96635220 -0.97641419 2.21054487
H 0.57638407 -0.24101372 -2.04402594
H -0.18196781 -2.54399340 -2.35451689
C 1.27172265 0.85630793 0.35882820
H 1.48783052 1.11027540 1.39559635
H 2.42871508 2.39211563 -0.46270035
H 1.33813667 1.64688026 -1.63451802
C 0.39523565 3.03998351 -0.26806259
C -0.94990615 2.82165955 -0.55146332
C 0.79074534 4.23021177 0.33437278
C -1.88949989 3.80025860 -0.25669887
H -1.26633004 1.89213507 -1.01574588
C -0.14977779 5.20704630 0.63029550
H 1.83874752 4.40412022 0.55615861
C -1.48975136 4.99321233 0.33425160
H -2.93461086 3.63266098 -0.48983551
H 0.16567455 6.13820119 1.08637478
H -2.22412307 5.75704186 0.56199797
C 1.42837800 1.96943860 -0.59818501
C -0.49240964 -4.43355287 -0.50880361
C 0.05952176 -5.58156096 0.33608481
C -1.33779739 -5.15363732 0.54125126
H -0.71509191 -4.66501671 -1.54219454
H 0.80198948 -5.32668762 1.08155961
H 0.24998111 -6.50033358 -0.20297146
H -2.13502768 -5.77005411 0.14692034
H -1.59121157 -4.59491592 1.43324057
*

constrB13
C -0.72743843 -3.44292118 -0.73218597
C 0.18185335 -3.20787929 0.30869122
C 0.79079346 -1.98086255 0.40416546
C 0.50273087 -0.96029814 -0.54605277
C -0.42534938 -1.22992288 -1.59167654
C -1.03113349 -2.46101688 -1.67718493
H 1.49666728 -1.77784466 1.20467802
H -0.65560870 -0.46371068 -2.32088246
H -1.73764538 -2.67231166 -2.47010144
C 1.14658800 0.26225310 -0.41356882
H 1.82908972 0.36261415 0.42978124
H 1.97865656 1.83102948 -1.49804973
H 0.42029320 1.29794669 -2.14011794
C 0.30736723 2.54178671 -0.36470023
C -1.04179009 2.42466145 -0.04233482
C 1.04884656 3.61749638 0.11541620
C -1.65462678 3.40168730 0.73083504
H -1.62224833 1.58235171 -0.40717813
C 0.43394580 4.59113496 0.88871780
H 2.10112500 3.70835745 -0.13329174
C -0.91686171 4.48349580 1.19663038
H -2.70835749 3.31841089 0.96974261
H 1.00843787 5.43767515 1.24598195
H -1.39711605 5.24545377 1.79932689
C 0.98054755 1.47407775 -1.22324639
H -1.19415807 -4.42225298 -0.78228695
C 0.46886007 -4.30110510 1.29786802
O -0.05789061 -5.37618051 1.22907470
H 1.19627922 -4.05091859 2.09287569
*

constrB14
C 0.20295239 -3.13892783 0.02006461
C 0.31823890 -2.64615770 1.31187551
C 0.64724149 -1.31691658 1.46042283
C 0.86109975 -0.49700874 0.32087811
C 0.73146542 -1.05319598 -0.97960634
C 0.39959757 -2.37854985 -1.12920973
H 0.14937400 -3.30070176 2.15611652
H 0.74525494 -0.88674280 2.45101814
H 0.89132952 -0.43971955 -1.85645653
H 0.29007380 -2.84097543 -2.10077569
C 1.20125624 0.83988055 0.53300503
H 1.26325499 1.16469062 1.57126524
H 2.34600066 2.41026216 -0.20100262
H 1.51771181 1.50309120 -1.47391518
C 0.26796722 2.89170206 -0.36660302
C -0.99548300 2.54258761 -0.83573766
C 0.47988198 4.13903189 0.21457866
C -2.03616944 3.45860379 -0.75893626
H -1.16724580 1.56628096 -1.27912779
C -0.56248230 5.05032554 0.29050703
H 1.46355120 4.40872106 0.58455873
C -1.81980654 4.71025738 -0.19505086
H -3.01627895 3.19457084 -1.13825980
H -0.39197346 6.02885545 0.72364908
H -2.63348771 5.42360846 -0.13593081
C 1.41786886 1.89008020 -0.46109030
N -0.15783355 -4.58226259 -0.15009263
O -0.31118092 -5.22163512 0.86384577
O -0.26423381 -4.97980750 -1.28668018
*

constrB15
C -0.02026083 -3.36627481 -0.09665832
C 0.30109681 -2.89029890 1.18191603
C 0.72916403 -1.60157643 1.34297480
C 0.85084857 -0.75323077 0.20714906
C 0.52487515 -1.24312298 -1.08808918

C 0.09538570 -2.53145948 -1.22271943
H 0.97138915 -1.23975752 2.33557918
H 0.60648435 -0.62397206 -1.97138321
C 1.29412454 0.54602649 0.41460128
H 1.50486140 0.82746662 1.44539823
H 2.43496799 2.07419592 -0.41763945
H 1.39651130 1.26674890 -1.59776778
C 0.38542341 2.68258971 -0.29970905
C -0.94322251 2.43300922 -0.63179809
C 0.74131935 3.88637055 0.30185151
C -1.90777927 3.40325184 -0.39541542
H -1.22695250 1.49146665 -1.09278397
C -0.22520020 4.85262327 0.53847851
H 1.77635666 4.08008777 0.56371668
C -1.54884444 4.61141996 0.19043189
H -2.93962294 3.21616491 -0.66848824
H 0.05626457 5.79707528 0.98906985
H -2.30303226 5.36773915 0.37383572
C 1.44969768 1.62130764 -0.56939503
F 0.17450284 -3.71653176 2.20649505
F -0.43091617 -4.59249450 -0.24596076
F -0.22380434 -3.03829904 -2.40190653
*

constrB16
C 0.10611834 -3.54976835 0.44445366
C 1.20300510 -2.97879258 1.10992442
C 1.56218408 -1.70013482 0.80386545
C 0.84824485 -0.93854366 -0.17640288
C -0.27224656 -1.54359111 -0.84243769
C -0.63196089 -2.81431734 -0.53980982
H 1.72895499 -3.57257902 1.84652582
H 2.40441618 -1.23733377 1.30585458
H -0.82865780 -0.98480099 -1.58514546
C 1.27242719 0.33914409 -0.43196256
H 2.12598709 0.69329145 0.14301816
H 1.48371245 1.72140153 -1.98931828
H -0.08415016 0.91186749 -1.99504397
C 0.12312917 2.48551606 -0.51926141
C -1.01627154 2.29425882 0.25730999
C 0.75791444 3.72345455 -0.52064870
C -1.52981400 3.34237208 1.00885963
H -1.51301586 1.32830575 0.26905600
C 0.24448926 4.77064842 0.23218193
H 1.64615560 3.87693118 -1.12491439
C -0.89966066 4.58134081 0.99613751
H -2.42316505 3.19320624 1.60419477
H 0.73571310 5.73659180 0.21696341
H -1.30247978 5.39943063 1.58181084
C 0.68339200 1.33746711 -1.34936549
O -0.24561767 -4.77820867 0.73270228
H -1.01475252 -5.04357700 0.19921736
O -1.65583795 -3.52698477 -1.07169225
H -2.13561042 -3.03161197 -1.74498868
*

constrB17
C -0.03390246 -2.94745329 -0.58361490
C -0.05478719 -2.55413568 0.80433276
C 0.32321056 -1.28801019 1.12755080
C 0.73896293 -0.35084802 0.11642734
C 0.74256493 -0.77220783 -1.24782360
C 0.36583190 -2.03646767 -1.58859147
H 0.31921266 -0.95770370 2.15795411
H 1.04652802 -0.08292100 -2.02426457
H 0.37260503 -2.34709471 -2.62353980
C 1.11841265 0.90971172 0.49089216
H 1.07229142 1.13985946 1.55423340
H 2.49370707 2.41490517 0.01290129
H 1.67674958 1.75840729 -1.40527981
C 0.49612891 3.15047197 -0.23954195
C -0.75864132 2.99885454 -0.82276546
C 0.78994864 4.30999335 0.47081023
C -1.70371956 4.00995293 -0.71425824
H -0.99755869 2.09392884 -1.37402833
C -0.15619169 5.31995039 0.58118818
H 1.76764343 4.43377502 0.92541183
C -1.40281332 5.17140326 -0.01223555
H -2.67560113 3.89275783 -1.17962855
H 0.08366213 6.22610181 1.12529161
H -2.14067614 5.96098482 0.06988041
C 1.53536259 2.04447730 -0.36393985
O -0.40490248 -4.17427422 -0.81373158
C -0.42376505 -4.69095160 -2.16208425
H -1.12485633 -4.11903922 -2.77133355
H 0.58093976 -4.66435345 -2.58592858
H -0.76287923 -5.71796821 -2.06370619
O -0.45833688 -3.51399449 1.64492025
C -0.50219011 -3.19056247 3.03645612
H 0.49425944 -2.92603260 3.40154272
H -1.20434875 -2.37221002 3.21935793
H -0.84932349 -4.09090109 3.53648223
*

constrB18
C -0.00425022 -3.07169733 0.04368223
C 0.97971306 -2.51560574 0.93592909
C 1.37876978 -1.23389631 0.79401789
C 0.85308367 -0.37426018 -0.23746678
C -0.13505914 -0.94454598 -1.11858618
C -0.54658217 -2.22323733 -0.98969357
H 1.40133736 -3.12372494 1.72219522
H 2.12013260 -0.82510033 1.47238625
H -0.56184896 -0.33389781 -1.90400794
H -1.28961855 -2.61387012 -1.66897304
C 1.29948999 0.90681012 -0.33129786
H 2.03866714 1.22541209 0.40157178
H 1.75774407 2.37738724 -1.76119630
H 0.21206332 1.58383069 -2.06024612
C 0.17777606 3.06881857 -0.48637162
C -1.07600740 2.84772628 0.07668553
C 0.80150012 4.29933457 -0.30877810
C -1.70648962 3.85469147 0.79448551
H -1.56875205 1.88831320 -0.05275417
C 0.17217142 5.30645563 0.41108193
H 1.77779349 4.47907064 -0.74755725
C -1.08285288 5.08589215 0.96192208
H -2.68725847 3.68022155 1.22197040
H 0.66076187 6.26594683 0.53553221
H -1.57702553 5.87232781 1.52050781
C 0.86664070 1.96659839 -1.27778471
N -0.40222616 -4.32691044 0.16731595
C 0.14599066 -5.19205913 1.21989368
C -1.40357875 -4.89753852 -0.74314752
H -0.31677913 -6.17131155 1.14335742
H 1.22461052 -5.30680267 1.09996497
H -0.07174995 -4.78117175 2.20712415
H -1.57306011 -5.93457952 -0.46964190
H -2.34893473 -4.35829968 -0.66237453
H -1.04876434 -4.86451688 -1.77452320
*

constrB19
C 0.00957891 -2.52203923 -0.01815809
C 0.12799173 -2.07349592 1.29864541
C 0.55249972 -0.77497935 1.50971715
C 0.86285195 0.06622091 0.41434030
C 0.73438768 -0.42495977 -0.90722182
C 0.30427978 -1.71753944 -1.12561510
H 0.64631820 -0.40328917 2.52403821
H 0.96677437 0.20010751 -1.75933246
C 1.29612757 1.37101912 0.68768589

H 1.35023348 1.65051304 1.73954596
H 2.55257495 2.89517655 0.05568668
H 1.73255348 2.07635601 -1.28050436
C 0.51352430 3.49666569 -0.17698610
C -0.74002571 3.24077534 -0.72703898
C 0.76392932 4.70007595 0.47781855
C -1.72909791 4.21307548 -0.66362190
H -0.94249891 2.29802672 -1.22660761
C -0.22778517 5.66642663 0.53996458
H 1.73879306 4.89406468 0.91258839
C -1.47328073 5.42256689 -0.02866153
H -2.69940215 4.02524982 -1.10773986
H -0.02731577 6.61321030 1.02732976
H -2.24729495 6.17940805 0.02122847
C 1.61040728 2.43340640 -0.25887346
N -0.44897803 -3.90298728 -0.25538663
O 0.41539861 -4.74184824 -0.23660742
O -1.63351074 -4.02439566 -0.44037533
C -0.18946903 -2.93584318 2.40540768
N -0.44387312 -3.61391599 3.29583901
C 0.16349801 -2.22733312 -2.46375358
N 0.05651512 -2.61994655 -3.53697773
*

constrB2
C -0.00968179 -3.42457211 -0.04625780
C 0.56438362 -2.94707550 1.14028235
C 1.00108296 -1.65093277 1.18013361
C 0.87992962 -0.80061785 0.04046810
C 0.28915136 -1.32481154 -1.14809316
C -0.14968244 -2.61788839 -1.18990208
H 0.65075618 -3.60330245 1.99633152
H 1.44699221 -1.25494356 2.08613905
H 0.18486796 -0.69824389 -2.02438437
H -0.60012289 -3.03450498 -2.08136260
C 1.34523335 0.49709990 0.13713397
H 1.76808714 0.79235287 1.09659407
H 2.28843740 2.00275647 -0.95282756
H 0.99563612 1.20524439 -1.85500190
C 0.32833920 2.64547173 -0.37620567
C -1.04329103 2.40821408 -0.37379357
C 0.82527428 3.85784952 0.09287217
C -1.91554027 3.39384775 0.06830309
H -1.43622427 1.46109321 -0.73180391
C -0.04826954 4.84064379 0.53558912
H 1.89428855 4.04424806 0.09268498
C -1.41815958 4.60932721 0.52325878
H -2.98409729 3.21346120 0.05694597
H 0.34086864 5.79003797 0.88430855
H -2.10048911 5.37809915 0.86664928
C 1.28740851 1.56704330 -0.87013587
Cl -0.55668440 -5.03199744 -0.10773521
*

constrB20
C 0.07545592 -1.96428362 -0.01564955
C 0.36626693 -1.50695234 1.27217898
C 0.79615507 -0.20767286 1.41693321
C 0.93185369 0.64056522 0.29219066
C 0.62469368 0.13949767 -0.99558433
C 0.20072274 -1.15710940 -1.15479726
H 1.03231344 0.16960154 2.40512786
H 0.71816802 0.77119468 -1.86832884
C 1.36879294 1.95181526 0.50142091
H 1.56895508 2.23460566 1.53454178
H 2.48174194 3.50898016 -0.30465905
H 1.50028453 2.66303948 -1.50865504
C 0.41321544 4.05340954 -0.24422292
C -0.89308907 3.77069560 -0.63423943
C 0.71364030 5.25642477 0.38927068
C -1.89071512 4.71452646 -0.43059788
H -1.13284916 2.82836512 -1.11786108
C -0.28655289 6.19476513 0.59256034
H 1.73096521 5.47233420 0.69851471
C -1.58752577 5.92369256 0.18398632
H -2.90463506 4.50513361 -0.75026644
H -0.04962370 7.14083000 1.06472715
H -2.36808803 6.65844142 0.34304796
C 1.51698794 3.02160874 -0.48045843
C 0.20546092 -2.38032731 2.51745989
F 0.93647168 -1.87301020 3.51951929
F 0.62038144 -3.62676603 2.28803697
F -1.06843855 -2.41076564 2.90700563
C -0.09911153 -1.67001957 -2.56431417
F -0.32069126 -0.63125000 -3.38355946
F -1.18473603 -2.44453315 -2.57376453
F 0.93350901 -2.36182162 -3.04290254
N -0.38574037 -3.36316094 -0.18563912
O -1.50951026 -3.58500640 0.18665855
O 0.41727706 -4.11452175 -0.67681194
*

constrB21
C 0.05642967 -2.75634783 -0.00941366
C -0.19327479 -2.27886871 1.27204767
C 0.12185477 -0.98065194 1.59394657
C 0.70668499 -0.14814230 0.60680919
C 0.96162887 -0.64182606 -0.69736401
C 0.62896593 -1.93995414 -0.98511933
H -0.07931868 -0.61128724 2.59273158
H 1.40762834 -0.02956222 -1.46939252
C 1.02291970 1.16412940 0.97393444
H 0.77687787 1.45263299 1.99519851
H 2.39250912 2.69876796 0.69662674
H 1.97457505 1.86448861 -0.80573233
C 0.48876702 3.27815131 -0.08896494
C -0.56653273 3.00516951 -0.95530411
C 0.54198926 4.49047540 0.59401480
C -1.54515049 3.96616384 -1.17123499
H -0.61785260 2.05542602 -1.47928589
C -0.43861850 5.44608892 0.37661367
H 1.36148074 4.69937032 1.27369651
C -1.48173677 5.18384482 -0.50440874
H -2.35785947 3.76325954 -1.85859524
H -0.38579500 6.39866307 0.89038395
H -2.24717120 5.93206696 -0.67381450
C 1.57527634 2.22737778 0.14010500
N -0.28989634 -4.14930859 -0.34576394
O 0.57344643 -4.96199919 -0.13298940
O -1.39613254 -4.31133325 -0.79500148
F 0.84192472 -2.44870631 -2.18690354
F -0.73709767 -3.09120399 2.16155062
*

constrB22
C 0.21962515 -2.37619458 -0.04063197
C 1.19282809 -1.78804103 0.84490398
C 1.52127847 -0.48620725 0.72008431
C 0.93431106 0.36298553 -0.28996834
C -0.04149317 -0.24246030 -1.16504536
C -0.38810636 -1.54009878 -1.04910546
H 1.64406052 -2.39057814 1.62001894
H 2.24739398 -0.04770023 1.39639867
H -0.50577154 0.35795105 -1.93697588
H -1.11279967 -1.96809935 -1.72691716
C 1.31669478 1.66375358 -0.37121200
H 2.05314011 2.00681981 0.35323871
H 1.68115254 3.16365130 -1.79929879
H 0.15738547 2.31747922 -2.06344309
C 0.11309084 3.78522937 -0.47448292
C -1.11697674 3.51433126 0.11845111
C 0.69675789 5.03560231 -0.29953521

C -1.76351320 4.49071905 0.86368854
H -1.57840562 2.53921041 -0.00890385
C 0.05162228 6.01235295 0.44783271
H 1.65419679 5.25449192 -0.76152796
C -1.17985621 5.74175765 1.02854618
H -2.72590482 4.27694082 1.31452492
H 0.50921492 6.98728459 0.57032871
H -1.68671286 6.50435372 1.60844785
C 0.81928189 2.71659883 -1.29541804
N -0.10847801 -3.64453306 0.06574515
C -1.14817872 -4.31209270 -0.74805493
C 0.51849400 -4.59195407 1.01315774
H -2.05347196 -3.70586923 -0.77160887
H -0.77721170 -4.44007594 -1.76892484
C -1.34815558 -5.65316472 -0.04693734
H 1.60302335 -4.48987088 0.98297679
H 0.16714547 -4.37210583 2.02532506
C 0.03476094 -5.95643768 0.52996789
H -2.08122859 -5.54828989 0.75650289
H -1.70447331 -6.41906224 -0.73456546
H 0.69946127 -6.33181096 -0.25179979
H 0.01292673 -6.68680586 1.33771517
*

constrB23
C -0.19482645 -2.86843176 -0.06339957
C 1.13468432 -2.33518482 0.14416097
C 1.46141100 -1.07102225 -0.17027910
C 0.49780110 -0.16790565 -0.74601371
C -0.81740502 -0.69613034 -0.99029236
C -1.14044120 -1.97080837 -0.68262115
H 2.48182025 -0.74298223 -0.00644476
H -1.57729826 -0.05539225 -1.41833553
H -2.14823628 -2.31579736 -0.85731004
C 0.86367671 1.11058922 -1.03315581
H 1.88533322 1.39756023 -0.79107099
H 0.54410534 2.63064503 -2.44830048
H -0.94869320 1.85379351 -1.92375071
C -0.12875424 3.27969592 -0.51775781
C -0.92577114 3.04747164 0.59956073
C 0.53677908 4.49449139 -0.64523194
C -1.07047427 4.02938202 1.56991255
H -1.44518468 2.09977675 0.70960446
C 0.39438821 5.47626441 0.32651960
H 1.15755291 4.68241925 -1.51546274
C -0.41053259 5.24546490 1.43377065
H -1.70095662 3.84747066 2.43273044
H 0.90782343 6.42418816 0.21422586
H -0.52591774 6.01238068 2.19086650
C 0.02527529 2.20514112 -1.58452805
N -0.54986834 -4.09482057 0.27337240
C 0.23224106 -4.98146517 1.15555043
C -1.83600874 -4.65291835 -0.16823527
H -0.47404872 -5.64023324 1.65717038
H 0.93660968 -5.57990131 0.57662313
H 0.76456613 -4.40852168 1.90663869
H -1.76860399 -5.73663737 -0.10787606
H -2.65061698 -4.31092349 0.47486956
H -2.03780555 -4.38274298 -1.20295264
F 2.09134046 -3.15477164 0.61071769
*

constrB24
C -0.06550547 -2.44883353 -0.15496936
C 0.15066175 -2.04472038 1.16176537
C 0.60059896 -0.77131203 1.41652923
C 0.84744146 0.12235513 0.34106047
C 0.62337182 -0.30796810 -0.99354074
C 0.16911570 -1.58096820 -1.22644816
H 0.76926343 -0.44412518 2.43616174
H 0.80483817 0.35802106 -1.82627104
C 1.30685102 1.40284357 0.64444700
H 1.43109659 1.63418800 1.70192306
H 2.54557946 2.94130190 -0.00246956
H 1.62141571 2.19863265 -1.31470597
C 0.50359531 3.58010949 -0.06826382
C -0.78032830 3.37802433 -0.56684069
C 0.80614388 4.73932360 0.64083839
C -1.74951079 4.35639594 -0.39052678
H -1.02291569 2.47036383 -1.11153428
C -0.16550073 5.71297154 0.81687365
H 1.80588035 4.89405275 1.03313611
C -1.44236623 5.52155383 0.30213319
H -2.74458394 4.20860128 -0.79322878
H 0.07585476 6.62455862 1.35067963
H -2.20049719 6.28376372 0.43894219
C 1.57579537 2.51039838 -0.27234789
H -0.42265692 -3.45544711 -0.35322806
C -0.13341096 -3.02763555 2.28377498
F 0.22003868 -2.52180979 3.47060397
F 0.54317094 -4.16571288 2.08822673
F -1.43880798 -3.32326674 2.32137598
C -0.08502330 -2.08586578 -2.63568131
F 0.11747184 -1.12059905 -3.54034157
F -1.34683360 -2.51791552 -2.75341296
F 0.73083810 -3.10848638 -2.91806324
*

constrB3
C -0.73343283 -2.71170277 -1.42676995
C -0.01661449 -2.62109123 -0.22911402
C 0.60164567 -1.44321824 0.10393999
C 0.51261695 -0.32388911 -0.77000487
C -0.22215837 -0.43961792 -1.98147429
C -0.83762176 -1.62652133 -2.29959072
H 1.15856150 -1.35724696 1.02988547
H -0.29825738 0.40552605 -2.65331438
H -1.39984896 -1.72746819 -3.21929480
C 1.16268315 0.84658906 -0.39553945
H 1.68817897 0.82529599 0.55870390
H 2.22315930 2.47068080 -1.14415116
H 0.78411900 2.08750615 -2.09731143
C 0.40443498 3.14878381 -0.24177149
C -0.98404048 3.07479305 -0.17524657
C 1.08767276 4.12402077 0.47921270
C -1.68889962 3.99934898 0.58391141
H -1.52042054 2.30937447 -0.72829788
C 0.38049297 5.04503352 1.23768275
H 2.17019549 4.17976987 0.43041078
C -1.00686008 4.98285600 1.29034124
H -2.77073402 3.95154260 0.62442187
H 0.91253115 5.81554532 1.78313708
H -1.55882456 5.70380072 1.88197967
C 1.18081173 2.13983024 -1.08461195
H -1.21927064 -3.64905556 -1.67964319
C 0.05587695 -3.83916404 0.67168174
F 0.75087187 -3.58101589 1.78556645
F 0.64633922 -4.85796527 0.03245909
F -1.17539051 -4.23330087 1.02410879
*

constrB4
C 0.17106406 -2.74503012 0.00685521
C 0.62374575 -2.24929057 1.22683181
C 1.02284304 -0.93347216 1.29933394
C 0.97524150 -0.10224842 0.14879884
C 0.50917659 -0.64083842 -1.08246312
C 0.11114366 -1.95113687 -1.14613332
H 0.65386996 -2.89013708 2.09807668
H 1.37625145 -0.51938640 2.23735442
H 0.46569861 -0.02100630 -1.96840644
H -0.25009108 -2.37721541 -2.07398568

C 1.39487680 1.21807749 0.27978801
H 1.72237234 1.52674373 1.27206709
H 2.36789585 2.76785160 -0.70802570
H 1.22007145 1.90298682 -1.73868056
C 0.32956916 3.30912373 -0.34475036
C -1.02110589 2.99868560 -0.47646601
C 0.71648063 4.54627583 0.16281736
C -1.98209602 3.94077915 -0.13454811
H -1.32726465 2.03076055 -0.86214390
C -0.24627157 5.48436105 0.50428778
H 1.76914090 4.78686432 0.26912964
C -1.59466435 5.18188823 0.35638286
H -3.03351265 3.70559984 -0.25072904
H 0.05642647 6.45439232 0.88068753
H -2.34620030 5.91597397 0.62244045
C 1.38850304 2.27851398 -0.73059421
C -0.25450636 -4.20391182 -0.11473628
F -1.41957640 -4.29367497 -0.76635214
F -0.39235409 -4.78028540 1.08047101
F 0.67015418 -4.87898694 -0.80931165
*

constrB5
C -0.33260932 -3.80937482 0.06030370
C 0.32055496 -3.33286326 1.21939712
C 0.82649678 -2.07183924 1.21391575
C 0.71240244 -1.22490087 0.06054732
C 0.04131794 -1.74261997 -1.09517057
C -0.46939448 -3.00204292 -1.09728148
H 0.39785571 -3.98685473 2.07858231
H 1.33013667 -1.68505920 2.09328227
H -0.06291348 -1.12538820 -1.97814958
H -0.97789317 -3.39792846 -1.96983156
C 1.24834607 0.03675669 0.11077988
H 1.72556512 0.32755474 1.04545317
H 2.23751575 1.45317962 -1.06619987
H 0.84765918 0.73277679 -1.88109438
C 0.35772718 2.24374617 -0.40639139
C -1.02447579 2.10149779 -0.32390500
C 0.95589707 3.43260650 -0.00068782
C -1.80479665 3.15246110 0.13857573
H -1.49792049 1.17415890 -0.63339776
C 0.17492788 4.48205674 0.46326413
H 2.03311964 3.54775720 -0.06448249
C -1.20523147 4.34311958 0.53225916
H -2.88186155 3.04316282 0.19036691
H 0.64478282 5.41096041 0.76474971
H -1.81566071 5.16349548 0.89129222
C 1.21422869 1.09328951 -0.92077226
O -0.80528592 -5.03773227 0.11608526
H -1.23820184 -5.30081553 -0.70849238
*

constrB6
C 0.01704725 -3.41273550 0.27456360
C 0.86079908 -2.83970030 1.26389672
C 1.26993004 -1.55529637 1.12696283
C 0.86875219 -0.76033877 -0.00198408
C 0.01445012 -1.36713784 -0.97979704
C -0.40388141 -2.65476805 -0.85236715
H 1.15273003 -3.45529334 2.10522844
H 1.91330669 -1.10578188 1.87561689
H -0.30768307 -0.79161045 -1.83823952
H -1.04762343 -3.09534478 -1.60021166
C 1.31547923 0.53024910 -0.09890347
H 1.95197996 0.88871631 0.70870310
H 1.94956950 1.94297259 -1.50591832
H 0.46191766 1.12126704 -1.98047589
C 0.21031420 2.66745084 -0.48344512
C -1.11218151 2.46089740 -0.10167401
C 0.81000216 3.90172279 -0.25411224
C -1.83582607 3.49034425 0.48471982
H -1.58574733 1.49844324 -0.27292214
C 0.08638806 4.93008299 0.33394503
H 1.84037478 4.06775667 -0.55170492
C -1.23679013 4.72564857 0.70251625
H -2.86894707 3.32950580 0.77033452
H 0.55502243 5.89342580 0.49805301
H -1.80345908 5.52914433 1.15843782
C 1.00276102 1.54036395 -1.13288772
O -0.32707506 -4.65573071 0.48515957
C -1.17989638 -5.34946175 -0.45270226
H -2.14742953 -4.85022836 -0.51767035
H -0.69587769 -5.40245961 -1.42866276
H -1.30001720 -6.34663525 -0.03945796
*

constrB7
C -0.01798921 -3.47556980 0.24672778
C 0.78079544 -2.90201162 1.25927719
C 1.21207801 -1.61035714 1.15420167
C 0.86570284 -0.81573834 0.01911535
C 0.05283067 -1.40740810 -0.99798788
C -0.37336137 -2.69602512 -0.88173317
H 1.04785299 -3.50040945 2.12205877
H 1.82514591 -1.17058684 1.93362944
H -0.22888339 -0.82710383 -1.86717158
H -0.98995134 -3.12389271 -1.66038609
C 1.33017187 0.47904489 -0.04486087
H 1.93104259 0.82410952 0.79543528
H 2.03186039 1.91654527 -1.38781727
H 0.58554435 1.08172640 -1.96320477
C 0.22531694 2.60666538 -0.46484240
C -1.11975583 2.38481704 -0.18369360
C 0.80230972 3.83687185 -0.16471598
C -1.88922943 3.39937395 0.36961416
H -1.57393938 1.42437952 -0.40929684
C 0.03224109 4.84931562 0.39012357
H 1.85072588 4.01257700 -0.38295145
C -1.31347597 4.63154940 0.65654473
H -2.93948785 3.22887285 0.57586839
H 0.48218032 5.81083529 0.60813867
H -1.91596690 5.42302511 1.08681737
C 1.06926407 1.49612285 -1.07986448
C -0.44732165 -4.86065949 0.41151560
C -1.16995047 -5.54971801 -0.47327395
H -0.13440922 -5.34325845 1.33189623
H -1.44422217 -6.57905058 -0.27469843
H -1.51038459 -5.13301593 -1.41387762
*

constrB8
C -0.09039702 -3.54136077 0.03315348
C 0.43961700 -3.03523686 1.23631147
C 0.88856296 -1.74404142 1.28051679
C 0.82862756 -0.90940928 0.12441129
C 0.28743722 -1.44439461 -1.08289127
C -0.16243149 -2.73309454 -1.12406822
H 0.48224715 -3.67521221 2.10802891
H 1.29711106 -1.33801131 2.19958165
H 0.23086598 -0.82853610 -1.97103728
H -0.57528436 -3.15240770 -2.03252880
C 1.30154449 0.38497794 0.22500821
H 1.68189427 0.69002353 1.19905418
H 2.30777040 1.86917896 -0.83984430
H 1.05439267 1.06660754 -1.79162319
C 0.32667852 2.53274970 -0.36826713
C -1.04497893 2.30172558 -0.42162421
C 0.80824742 3.75189144 0.09914012
C -1.93079657 3.29911554 -0.03662716
H -1.42642775 1.34935877 -0.77805777
C -0.07880041 4.74665568 0.48469999

H 1.87717858 3.93366344 0.14259613
C -1.44794347 4.52100203 0.41680183
H -2.99871790 3.12276885 -0.09114814
H 0.29959030 5.70075493 0.83247289
H -2.14081119 5.29891053 0.71567269
C 1.30092687 1.44122060 -0.79926153
C -0.55992137 -4.88246258 -0.02005144
C -0.95626894 -6.01367528 -0.06532652
H -1.30890381 -7.02175135 -0.10524757
*

constrB9
C -0.02204069 -3.54774989 -0.02224001
C 0.42572197 -3.06909648 1.21565728
C 0.86448672 -1.77017044 1.30197690
C 0.86645311 -0.93087791 0.15466645
C 0.40749339 -1.44621819 -1.08840560
C -0.03305769 -2.74174932 -1.17351122
H 0.41874288 -3.72036169 2.08027825
H 1.21325245 -1.37327958 2.24903004
H 0.40252091 -0.81985606 -1.97081828
H -0.38697684 -3.15350170 -2.11010514
C 1.32654203 0.37507725 0.30010226
H 1.64320321 0.66874378 1.30040023
H 2.36906071 1.89746988 -0.65838263
H 1.21780357 1.07419565 -1.71839702
C 0.34142043 2.50122143 -0.33665690
C -1.01516203 2.23509960 -0.50156939
C 0.75607447 3.72205970 0.18851328
C -1.95310777 3.20583689 -0.17620661
H -1.34327109 1.27995885 -0.90085208
C -0.18380629 4.68866659 0.51351184
H 1.81311993 3.92792698 0.32094691
C -1.53745942 4.43077422 0.33201486
H -3.00851692 3.00563715 -0.31866798
H 0.14090525 5.64622313 0.90325207
H -2.27101187 5.18722365 0.58529486
C 1.37553144 1.43922104 -0.70468541
C -0.48643760 -4.90547883 -0.12032603
N -0.86088139 -5.98895516 -0.19955131
*

constrD0
N 0.11429047 1.48815702 -1.64611933
O 1.14819591 1.42574334 -0.72190230
C -1.11100348 1.72172898 -0.90377861
C 0.06951993 0.25416105 -2.40896434
H -1.95427092 1.55116219 -1.59145219
C -1.17626807 3.15733001 -0.38820152
H 1.01496555 0.14426596 -2.96344252
H -0.05945837 -0.64180023 -1.76563380
H -0.75755090 0.28878650 -3.13723512
H -1.13285341 3.86678742 -1.23058230
H -2.10901640 3.33153553 0.17139496
H -0.32409937 3.35289947 0.27971666
C -0.22884853 0.45637677 1.04254449
C -1.26214706 0.70734325 0.25098760
O 0.69294298 0.23249289 1.74868170
Li 2.08962708 0.10021221 0.04800894
O 2.09684200 -1.80869702 -0.26734512
O 3.90689600 0.54335338 0.43927977
C 1.12545137 -2.73197824 0.19160811
H 0.28517782 -2.81146101 -0.52170674
H 1.57549612 -3.73106573 0.33005292
H 0.75233407 -2.37189979 1.15915752
C 2.75408904 -2.22037879 -1.45268715
H 2.02876794 -2.40235054 -2.26532797
H 3.44108322 -1.41728370 -1.75621674
H 3.33494950 -3.14310096 -1.27522672
C 4.93773756 -0.33231114 0.85135830
H 5.39450106 0.01518261 1.79551983
H 4.49186896 -1.32396199 1.00904186
H 5.72352718 -0.40564577 0.07754231
C 4.33155940 1.87583584 0.20695127
H 5.11556477 1.90521862 -0.57139709
H 3.45171511 2.43732477 -0.13743790
H 4.72725051 2.32692402 1.13463799
C -2.55520766 0.03123344 0.53612368
C -3.77297479 0.57994860 0.09671971
C -2.60023457 -1.18171138 1.25214890
C -4.98574198 -0.06038815 0.36332912
H -3.78156463 1.52518914 -0.45121185
C -3.81222158 -1.81322439 1.52580824
H -1.66783714 -1.64775306 1.58716985
C -5.01533254 -1.25796949 1.07971002
H -5.91842368 0.38962425 0.00934771
H -3.81503596 -2.75462043 2.08375460
H -5.96644385 -1.75667661 1.28738340
*

constrD1
N -2.08951012 -0.29724176 -1.11338719
O -0.86058471 0.28359121 -0.84018857
C -2.99022086 0.03941819 -0.02357494
C -1.92178457 -1.72798325 -1.27535701
H -3.89094902 -0.58828561 -0.12516090
C -3.40855112 1.50722055 -0.09948639
H -1.28665737 -1.91630699 -2.15547926
H -1.43771804 -2.20248160 -0.39425670
H -2.89966613 -2.20818447 -1.44662673
H -3.90556666 1.71166490 -1.06205061
H -4.10441496 1.76087179 0.71618805
H -2.52089462 2.15226166 -0.01811901
C -1.10228413 -0.09534273 1.64173580
C -2.36901469 -0.28022956 1.34630842
H -3.00522972 -0.64880002 2.15720427
O 0.03979336 0.07135010 1.90809163
Li 0.76466334 0.04916479 -0.18605587
O 2.00337409 -1.42418124 -0.18865426
O 1.93312718 1.60608960 -0.15598741
C 2.93979483 -1.48769089 0.87208077
H 2.58379457 -2.16255532 1.67082647
H 3.91915375 -1.84436281 0.50592105
H 3.05386690 -0.47426465 1.28058543
C 1.77675248 -2.67009273 -0.82205834
H 1.33983300 -3.39880760 -0.11557554
H 1.07617623 -2.50329532 -1.65101348
H 2.72139056 -3.07924848 -1.22277010
C 3.00970990 1.68571735 -1.07154132
H 3.77588832 2.39626882 -0.71313542
H 3.45520040 0.68464152 -1.15305095
H 2.65815874 2.00861840 -2.06843775
C 1.27230590 2.84285682 0.04819601
H 0.82416144 3.21066224 -0.89200386
H 0.47602016 2.67841045 0.78643118
H 1.97706532 3.60012167 0.43578025
*

constrD14
N 1.37661975 1.88439944 -1.23554456
O 2.16875104 1.38097981 -0.21633523
C 0.06237362 2.08830264 -0.66088266
C 1.35872998 0.95118175 -2.34791080
H -0.64710600 2.23223437 -1.49036671
C 0.03650473 3.32283713 0.23769620
H 2.38450154 0.83675408 -2.73258734
H 0.98635102 -0.05376704 -2.05796438
H 0.72554163 1.34387329 -3.16025172
H 0.29017569 4.22255980 -0.34578927
H -0.95985291 3.46367632 0.68581292
H 0.77254347 3.20746946 1.04707923
C 0.48506839 0.14730468 0.86207276

C	-0.38659318	0.83288925	0.11845840
O	1.25484413	-0.45814474	1.51876932
Li	2.98050298	-0.18926383	0.11940699
O	3.10068657	-1.82245969	-0.88033111
O	4.58199751	-0.04010814	1.14941756
C	2.02466831	-2.68923079	-1.19257241
H	1.70212222	-2.55499485	-2.24020177
H	2.31847189	-3.74296748	-1.03906945
H	1.18834537	-2.44857584	-0.52240463
C	4.23855809	-2.01272420	-1.70285881
H	3.98539714	-1.85151031	-2.76580358
H	5.00201308	-1.28238373	-1.39978521
H	4.64479410	-3.03238120	-1.57885468
C	5.41909151	-1.08103890	1.61384691
H	5.44539128	-1.10008468	2.71821501
H	5.00929686	-2.03265382	1.24776708
H	6.44857517	-0.95749535	1.23154501
C	4.97666707	1.25567714	1.56983187
H	5.98903789	1.49481922	1.19721000
H	4.24893853	1.96412705	1.14961965
H	4.97191842	1.32579032	2.67245186
C	-1.79600580	0.39982620	0.14330603
C	-2.82042651	1.21315395	-0.38570862
C	-2.16965382	-0.84735470	0.69888679
C	-4.14957284	0.80542054	-0.36617107
H	-2.58603162	2.18903556	-0.81284680
C	-3.49090342	-1.26391533	0.72804232
H	-1.40621764	-1.51435488	1.10982825
C	-4.47549137	-0.43048724	0.19016587
H	-3.76083431	-2.22985911	1.15710009
H	-4.93112068	1.44639461	-0.77662763
N	-5.87146935	-0.86321665	0.21118706
O	-6.13202643	-1.94652280	0.70609904
O	-6.71329795	-0.12235595	-0.26691785

*

constrD15

N	0.93132891	1.61965138	-1.78518362
O	2.00907812	1.28971275	-0.97685505
C	-0.17592427	1.94321820	-0.90759413
C	0.63137601	0.51146378	-2.67162043
H	-1.08038595	2.00744018	-1.53268330
C	0.04505152	3.28634856	-0.21550162
H	1.48534321	0.35425808	-3.34921575
H	0.44870446	-0.43675204	-2.12160888
H	-0.25607470	0.74455066	-3.28305076
H	0.15233940	4.08723874	-0.96489816
H	-0.80183966	3.53332744	0.44422061
H	0.96202666	3.24642846	0.39082624
C	0.58694375	0.23416318	0.75210164
C	-0.42679645	0.82377680	0.12576375
O	1.48330955	-0.28718305	1.30591994
Li	3.03026888	-0.10816537	-0.58507993
O	3.12787177	-1.93936927	-1.12973267
O	4.52771184	0.28625878	0.54385444
C	2.97845451	-2.98334190	-0.18324088
H	1.97305664	-3.43531266	-0.25517222
H	3.74091993	-3.76613994	-0.34398855
H	3.10713628	-2.55385425	0.81919638
C	3.01610495	-2.37027039	-2.47493310
H	2.01384979	-2.79021465	-2.67306590
H	3.17923778	-1.49886182	-3.12364834
H	3.77916024	-3.13630096	-2.70056288
C	5.52328705	-0.59875364	1.01811105
H	5.28373756	-0.95315442	2.03722919
H	5.56423354	-1.45808449	0.33442484
H	6.51090770	-0.10379584	1.03420860
C	4.38726015	1.46607179	1.31875846
H	5.32025862	2.05726073	1.29865635
H	3.56948451	2.04904451	0.87242327
H	4.13899367	1.21892550	2.36631851
C	-1.80043201	0.42495701	0.51402181
C	-2.90034487	1.24730607	0.21167832
C	-2.04049797	-0.78474537	1.19774661
C	-4.18159069	0.86323232	0.58618647
H	-2.79047820	2.20308758	-0.30210733
C	-3.32857777	-1.13640211	1.56675410
H	-1.23361829	-1.47959158	1.44445473
C	-4.42148845	-0.32508217	1.26730940
F	-3.55451267	-2.28577936	2.21334476
F	-5.66048054	-0.68146688	1.62085136
F	-5.22651554	1.64776379	0.29762132

*

constrD18

N	1.49110093	1.76985932	-1.47616861
O	2.41861052	1.30325750	-0.55396514
C	0.29244940	2.13560586	-0.74265010
C	1.23699255	0.74270666	-2.46926953
H	-0.50907721	2.30652037	-1.47906244
C	0.51140769	3.41823220	0.05600446
H	2.17483117	0.52705433	-3.00544390
H	0.87054441	-0.20501937	-2.02093190
H	0.49270116	1.09852853	-3.20104197
H	0.78753365	4.24463961	-0.61918545
H	-0.40128789	3.70108863	0.60417368
H	1.32554449	3.27146084	0.78143246
C	0.68625620	0.35298707	0.93725311
C	-0.18552429	0.98470374	0.16989681
O	1.46782257	-0.21335778	1.62520132
Li	2.94864214	-0.31473471	0.02697983
O	2.61263854	-2.08549034	-0.68626469
O	4.77852660	-0.35187236	0.58485039
C	1.43067828	-2.84463486	-0.50735939
H	0.67937716	-2.59606980	-1.27870275
H	1.65275085	-3.92552123	-0.55732575
H	1.02859082	-2.60826055	0.48638740
C	3.29802063	-2.37962575	-1.89012833
H	2.64099859	-2.23506425	-2.76601550
H	4.15553726	-1.69575953	-1.96710490
H	3.66626841	-3.42119261	-1.88457893
C	5.56925225	-1.48240615	0.89226078
H	6.00062919	-1.39711931	1.90602909
H	4.92000289	-2.36777924	0.84944663
H	6.39070727	-1.59836633	0.16177527
C	5.48251477	0.87828048	0.61188050
H	6.32423951	0.86176565	-0.10417712
H	4.76787294	1.66104617	0.32071477
H	5.87507687	1.08149404	1.62453016
C	-1.62091415	0.58923045	0.23294490
C	-2.64742359	1.49922108	-0.06329993
C	-2.01662393	-0.70869319	0.59902572
C	-3.99317431	1.13999523	-0.00072626
H	-2.40171832	2.52822789	-0.33981366
C	-3.35557799	-1.08159554	0.67745153
H	-1.25419697	-1.46240314	0.82391385
C	-4.38920827	-0.16655099	0.36624872
H	-3.59222960	-2.10493696	0.97157052
H	-4.74100673	1.89731127	-0.23925640
N	-5.72175400	-0.53451450	0.41549131
C	-6.74516845	0.47920059	0.26515970
C	-6.08571475	-1.81676589	0.98198309
H	-7.73349153	0.00264814	0.30786158
H	-6.70136953	1.25251814	1.05702480
H	-6.66651900	0.99081000	-0.70890948
H	-7.17600918	-1.93640914	0.93146131
H	-5.63523565	-2.65129555	0.41831198
H	-5.77774049	-1.91950267	2.04096397

*

constrD19

N	1.95147873	2.18855163	-0.69036123

O	2.69641345	1.40189127	0.17033116
C	0.62814714	2.24786511	-0.10634183
C	1.94915021	1.59706454	-2.01770339
H	-0.06786652	2.60433049	-0.88163159
C	0.58389098	3.20201042	1.08526170
H	2.98326753	1.56720641	-2.39525991
H	1.54890221	0.56188603	-2.02252766
H	1.34795047	2.21522156	-2.70377760
H	0.84093268	4.22369583	0.76300291
H	-0.41962889	3.21947555	1.53940486
H	1.30769608	2.87774026	1.84737938
C	1.06314546	-0.03191878	0.85192062
C	0.18599610	0.82892296	0.31387167
O	1.83351733	-0.78722603	1.32521852
Li	3.57410664	-0.16930764	0.02689171
O	3.63221728	-1.47506185	-1.37209864
O	5.19949276	-0.30216721	1.01260783
C	2.54009271	-2.24389601	-1.84508918
H	2.15749391	-1.83929862	-2.79882858
H	2.84262563	-3.29540966	-1.99598596
H	1.74426548	-2.20449375	-1.08909128
C	4.72177728	-1.42222580	-2.27708104
H	4.40714530	-0.99207518	-3.24445507
H	5.49934176	-0.78320260	-1.83511919
H	5.13700854	-2.43118842	-2.44869754
C	6.06700073	-1.41608043	1.10752332
H	6.15722329	-1.75360746	2.15548761
H	5.63990561	-2.22883077	0.50397085
H	7.07163397	-1.16512800	0.72199082
C	5.60945679	0.81982324	1.77785590
H	6.59935488	1.17915283	1.44372929
H	4.85850514	1.60719826	1.62329902
H	5.66216882	0.56090915	2.85045935
C	-1.21942541	0.41563918	0.24359815
C	-2.23918173	1.33643576	-0.06494917
C	-1.61096667	-0.91990732	0.48117831
C	-3.57960897	0.94855564	-0.14256018
H	-2.00625524	2.38675595	-0.23759329
C	-2.94546519	-1.31732092	0.43282104
H	-0.86299987	-1.68469537	0.70288286
C	-3.94040657	-0.38040471	0.11182757
N	-5.34325212	-0.79117141	0.04387866
O	-5.71641856	-1.64880246	0.81922398
O	-6.04906206	-0.25041444	-0.78384447
C	-4.55155595	1.98045006	-0.43073934
N	-5.26808802	2.86160470	-0.64663496
C	-3.23207841	-2.71597598	0.66334634
N	-3.37856257	-3.84906648	0.83983045

*

constrD2

N	1.00448656	1.70746080	-1.45470183
O	1.98485156	1.31817558	-0.55367377
C	-0.16404468	2.07703263	-0.67860126
C	0.73382840	0.62156126	-2.37902235
H	-1.01337550	2.16411342	-1.37451112
C	0.04238300	3.41764437	0.02227711
H	1.65057690	0.40371340	-2.94933968
H	0.41800476	-0.31017495	-1.86391965
H	-0.05463006	0.91782374	-3.09044766
H	0.23845703	4.20863847	-0.71974291
H	-0.84804476	3.69665424	0.60781108
H	0.90403324	3.35321037	0.70337861
C	0.42820311	0.41325253	1.07086570
C	-0.52546134	0.96952201	0.33551745
O	1.27821393	-0.08255597	1.72628893
Li	2.65084785	-0.24881294	0.02190570
O	2.29823508	-2.06257251	-0.55332510
O	4.51517123	-0.21286314	0.43808390
C	1.15788229	-2.83355740	-0.21878511
H	0.32474535	-2.63381058	-0.91685631
H	1.39787448	-3.91128302	-0.24419987
H	0.85606626	-2.55966926	0.80054437
C	2.87522016	-2.41504632	-1.79819379
H	2.13860677	-2.32557104	-2.61610362
H	3.71243253	-1.72864043	-1.99063052
H	3.25815459	-3.45083400	-1.77009685
C	5.35629173	-1.31297892	0.72396007
H	5.87036197	-1.17333069	1.69197665
H	4.72675760	-2.21197019	0.77537122
H	6.11459301	-1.44461130	-0.06932545
C	5.19115809	1.03014941	0.34742728
H	5.96509677	0.99635147	-0.44071193
H	4.43719567	1.78694051	0.08886992
H	5.66813367	1.28651069	1.31042902
C	-1.93006339	0.54412776	0.56069773
C	-3.00776180	1.37771567	0.21340986
C	-2.22985142	-0.71006326	1.12880798
C	-4.32892864	0.98081037	0.42478504
H	-2.82508505	2.36351455	-0.21993723
C	-3.54316818	-1.11324948	1.35432370
H	-1.41966954	-1.39826141	1.38926212
C	-4.58978019	-0.26220827	0.99702920
H	-3.75549579	-2.09005489	1.79549072
H	-5.15466013	1.64177983	0.15054299
Cl	-6.23790893	-0.76240848	1.26562530

*

constrD21

N	1.78226830	2.09952816	-0.88099062
O	2.50613527	1.42670678	0.08853507
C	0.43771657	2.20915359	-0.35290395
C	1.82878583	1.35886511	-2.13023335
H	-0.23454423	2.45480826	-1.18921014
C	0.33782452	3.30728107	0.70344085
H	2.87618772	1.29109758	-2.46361835
H	1.42891976	0.32814244	-2.03205078
H	1.25316379	1.89159310	-2.90440061
H	0.59565490	4.28343255	0.26216673
H	-0.68301311	3.36612281	1.11328070
H	1.03512511	3.09639055	1.52790439
C	0.85996983	0.08003046	0.90309063
C	-0.00544926	0.85146928	0.23121340
O	1.62174348	-0.59901375	1.49449774
Li	3.35379681	-0.16775841	0.16203434
O	3.39266755	-1.62541840	-1.08591349
O	5.00259904	-0.20554453	1.12056034
C	2.29324868	-2.43938438	-1.45483365
H	1.90138235	-2.14639911	-2.44493132
H	2.59106424	-3.50260880	-1.48634758
H	1.50535822	-2.30892377	-0.70082073
C	4.46431278	-1.67077073	-2.01174578
H	4.12933300	-1.35203719	-3.01477461
H	5.24757805	-0.98499887	-1.65918326
H	4.88085956	-2.69159608	-2.07834671
C	5.85223929	-1.31674521	1.33338744
H	5.96459216	-1.52240593	2.41301582
H	5.39448584	-2.18906890	0.84679068
H	6.85080949	-1.13721026	0.89557070
C	5.45803544	0.99639384	1.72042567
H	6.44299785	1.28682103	1.31252929
H	4.71764105	1.77423592	1.48716029
H	5.54089117	0.87806267	2.81564643
C	-1.40455578	0.40665222	0.17182868
C	-2.40884755	1.23789213	-0.36401488
C	-1.78484367	-0.87000438	0.64857851
C	-3.72028763	0.79982684	-0.42904541
H	-2.20133911	2.24415117	-0.72718137
C	-3.10094165	-1.27837206	0.59333635
H	-1.05896395	-1.57459232	1.06146449
C	-4.10282381	-0.46000125	0.04707245
N	-5.48327169	-0.90554452	-0.02134818

O	-5.93197747	-1.51219470	0.93335252
O	-6.11431130	-0.64858897	-1.02969997
F	-4.64428035	1.62105539	-0.92140183
F	-3.41677913	-2.49593842	1.02821007

*

constrD6

N	1.03747999	1.70492533	-1.51594955
O	2.01568156	1.35587997	-0.59473520
C	-0.16277086	2.03436186	-0.76919578
C	0.82760876	0.60635991	-2.44073395
H	-0.99434206	2.10330498	-1.48852381
C	-0.01295790	3.37603170	-0.05594037
H	1.76401711	0.42086195	-2.99041538
H	0.53636753	-0.33495996	-1.92832045
H	0.04369503	0.86935327	-3.17026304
H	0.18469314	4.17633374	-0.78765012
H	-0.92761978	3.62830293	0.50379716
H	0.82960882	3.33032056	0.65009167
C	0.41022891	0.39133580	1.00001810
C	-0.52940301	0.91175958	0.22658511
O	1.25067574	-0.07415344	1.69180939
Li	2.68207391	-0.18851891	0.03650416
O	2.42337440	-2.01594813	-0.54774800
O	4.52617608	-0.08991872	0.53537825
C	1.30821696	-2.83933101	-0.25767234
H	0.50740229	-2.70087185	-1.00651320
H	1.60547473	-3.90302215	-0.24232279
H	0.93470808	-2.55893243	0.73576663
C	3.07426045	-2.35007019	-1.76042546
H	2.37258567	-2.30549655	-2.61213937
H	3.88326126	-1.62316398	-1.92217856
H	3.50746888	-3.36473740	-1.70343287
C	5.38959324	-1.15788258	0.87082193
H	5.85693156	-0.99041482	1.85800636
H	4.78824917	-2.07670991	0.90690106
H	6.18535100	-1.27351462	0.11240543
C	5.16353400	1.17414440	0.46012744
H	5.97298663	1.15851748	-0.29221452
H	4.39664606	1.90163481	0.15888633
H	5.58802040	1.45664041	1.44031052
C	-1.92874123	0.42488834	0.37289477
C	-3.02251292	1.22895711	0.02865665
C	-2.20750845	-0.86788740	0.86443053
C	-4.34190422	0.77870895	0.16425759
H	-2.86115651	2.24230060	-0.34747814
C	-3.50995986	-1.32352987	1.01469044
H	-1.38267124	-1.53951345	1.12348594
C	-4.59328554	-0.50247493	0.66307969
H	-3.70934425	-2.32855204	1.39622661
H	-5.15803912	1.44532422	-0.11890590
O	-5.83520572	-1.03076951	0.83474002
C	-6.95783381	-0.23549971	0.49814574
H	-6.95415524	0.03465053	-0.57188474
H	-7.84673736	-0.84433473	0.71106964
H	-6.99692487	0.68451208	1.10659288

*

constrD8

N	1.05740617	1.89762904	-1.18679118
O	1.90729749	1.41027718	-0.20482047
C	-0.22746313	2.12641198	-0.55339793
C	0.97466134	0.94446747	-2.27843893
H	-0.97265758	2.27483174	-1.35058910
C	-0.19181790	3.37149774	0.32984978
H	1.97525015	0.82578377	-2.72340563
H	0.62223220	-0.05564307	-1.94862140
H	0.29189638	1.32067288	-3.05797540
H	0.05878335	4.25883387	-0.27389159
H	-1.16720780	3.53850894	0.81382180
H	0.57230787	3.25105411	1.11224620
C	0.20593105	0.26363173	1.03743423
C	-0.66746260	0.88520856	0.25256157
O	0.98288254	-0.28930885	1.73563914
Li	2.57256670	-0.21196475	0.21071830
O	2.34808625	-1.94429169	-0.62117298
O	4.37522825	-0.18934513	0.83926837
C	1.18629864	-2.75194435	-0.54302164
H	0.44407798	-2.45410727	-1.30566751
H	1.44416722	-3.81601310	-0.68695017
H	0.75501907	-2.62229092	0.45808793
C	3.08998930	-2.13426891	-1.81324171
H	2.46584776	-1.94225927	-2.70377915
H	3.93274650	-1.42807817	-1.80448473
H	3.48436594	-3.16475681	-1.86539475
C	5.21860898	-1.29647511	1.08975706
H	5.59434283	-1.27677655	2.12859670
H	4.62745192	-2.20981265	0.93660296
H	6.07939278	-1.29937407	0.39657657
C	5.00188636	1.07118984	1.01027408
H	5.87138058	1.16904070	0.33529611
H	4.25434791	1.83712380	0.76022012
H	5.33937549	1.20078065	2.05425390
C	-2.06774085	0.39946751	0.23618079
C	-3.12324429	1.23045487	-0.18278263
C	-2.38995404	-0.91357894	0.63860931
C	-4.43900048	0.77323491	-0.19535050
H	-2.92406876	2.25821780	-0.49350808
C	-3.70162509	-1.37148181	0.63590487
H	-1.59377043	-1.59829956	0.94686935
C	-4.74893828	-0.53255585	0.21548090
H	-3.92214358	-2.39440395	0.95236523
H	-5.24105362	1.44011007	-0.52278842
C	-6.11252428	-1.00558883	0.20371778
C	-7.25837238	-1.40172779	0.19401092
H	-8.27836546	-1.75429204	0.18563940

*

constrD9

N	0.97740399	1.81859475	-1.27403692
O	1.82262304	1.31900876	-0.29574551
C	-0.29800395	2.05292365	-0.62739796
C	0.87893851	0.86823236	-2.36756986
H	-1.05216628	2.19456530	-1.41705715
C	-0.25404533	3.30381209	0.24769546
H	1.87829866	0.73055287	-2.80982135
H	0.50762800	-0.12557475	-2.04009987
H	0.20556285	1.25896451	-3.14793213
H	-0.02070114	4.18892494	-0.36596664
H	-1.22122101	3.46833049	0.74861056
H	0.52475706	3.19185323	1.01656766
C	0.17609196	0.13638562	0.90710294
C	-0.72310037	0.81995499	0.19906411
O	0.97248414	-0.46903465	1.53419525
Li	2.58343279	-0.27958799	0.02749111
O	2.53707290	-1.94327016	-0.93692986
O	4.30057094	-0.18834100	0.85873643
C	1.41159053	-2.78578284	-1.10815676
H	0.97123503	-2.65628389	-2.11270861
H	1.69564332	-3.84492402	-0.97444420
H	0.66591581	-2.51537494	-0.34863149
C	3.56225517	-2.16910190	-1.88812926
H	3.18540575	-2.01647939	-2.91512776
H	4.37128355	-1.45169276	-1.69085805
H	3.95993934	-3.19581012	-1.79878841
C	5.12843696	-1.26183855	1.26146644
H	5.27572703	-1.25462569	2.35642916
H	4.63145713	-2.19800079	0.97166823
H	6.11431419	-1.20499252	0.76545129
C	4.80496078	1.09395693	1.19406827
H	5.78397347	1.26633929	0.71170713
H	4.07593631	1.83044622	0.82800190

H	4.91922675	1.19397009	2.28846467
C	-2.13979050	0.41091891	0.30499073
C	-3.17633333	1.23390238	-0.17890885
C	-2.50382469	-0.82024296	0.89685574
C	-4.50964708	0.84804488	-0.08143288
H	-2.94821498	2.19952339	-0.63281249
C	-3.83000993	-1.21123886	1.00252966
H	-1.72999975	-1.49383535	1.27728854
C	-4.84827101	-0.37713589	0.50970998
H	-4.08392025	-2.16928154	1.46289985
H	-5.29585760	1.50431653	-0.46294989
C	-6.22856539	-0.77909724	0.61084970
N	-7.33809548	-1.10308870	0.69266637

*

constrF14

Au	0.03570096	-2.18011027	0.16141819
C	1.32818046	0.21274011	1.39614536
C	0.15852249	-0.38555835	1.18173432
C	0.26582561	2.17908976	1.99911569
C	1.51224569	1.49417575	2.28822620
H	1.57781850	1.19434625	3.33356969
C	-1.11768479	0.24344460	1.63856240
H	-1.75023206	-0.45435470	2.19053944
H	-1.68425754	0.57196434	0.76280712
C	-0.90302975	1.52065927	2.56288083
P	-0.11038293	-4.18694494	-0.96641883
C	1.07376957	-4.36145409	-2.32660874
C	0.17860303	-5.63913000	0.07824580
H	2.26483692	-0.12652765	0.96557077
C	-1.72799678	-4.48427315	-1.72668887
H	-1.94476477	-3.68942946	-2.44296392
H	-1.73408995	-5.44848450	-2.24051170
H	-2.49892974	-4.47957508	-0.95365062
H	0.91236032	-3.56445488	-3.05498006
H	0.94877552	-5.33112654	-2.81438728
H	2.09003180	-4.27934339	-1.93636715
H	1.17687420	-5.57878140	0.51600181
H	0.09545905	-6.55383290	-0.51350257
H	-0.55720549	-5.65938532	0.88455257
H	2.41133610	2.02333317	1.98867911
H	-1.81634970	2.10758608	2.54393056
H	-0.68643533	1.16360647	3.56907002
C	0.16297775	3.26334246	1.04136918
C	-1.00921225	4.03114968	0.97076763
C	1.23028648	3.55283552	0.17384051
C	-1.11603476	5.06865098	0.06079916
H	-1.83298930	3.84613644	1.64569167
C	1.12453854	4.57089196	-0.75244470
H	2.13615742	2.96267645	0.19838466
C	-0.04784893	5.31002302	-0.78316914
H	-2.00909208	5.67530116	0.00802148
H	1.93315421	4.79124043	-1.43510012
N	-0.16028431	6.41540312	-1.76510422
O	-1.21241354	7.01990387	-1.81510014
O	0.80624937	6.65051972	-2.46191110

*

constrF15

Au	-0.04270362	-2.11463757	0.18076673
C	1.52301048	0.30433037	0.95420058
C	0.32282997	-0.26745737	1.03443820
C	0.62770822	2.37188377	1.63327019
C	1.90353743	1.66405862	1.62561195
H	2.25373582	1.47685325	2.64183795
C	-0.81058535	0.43655390	1.71538037
H	-1.31952929	-0.21120834	2.43200111
H	-1.55187347	0.73614510	0.96844722
C	-0.36209884	1.73581338	2.49866631
P	-0.43233725	-4.17726678	-0.77696460
C	0.68026174	-4.56823549	-2.15299201
C	-0.24844453	-5.56948187	0.36866569
H	2.34743875	-0.12367995	0.39275458
C	-2.09622895	-4.37216742	-1.46797804
H	-2.26163347	-3.61481459	-2.23669333
H	-2.21036112	-5.36642696	-1.90639165
H	-2.83654307	-4.23660615	-0.67714274
H	0.56794768	-3.81552835	-2.93576200
H	0.44749168	-5.55474133	-2.56087370
H	1.71331794	-4.55533256	-1.80014827
H	0.76568507	-5.57630557	0.77281326
H	-0.44106850	-6.51330127	-0.14702961
H	-0.95388993	-5.45552910	1.19400144
H	2.69064739	2.13709898	1.04739239
H	-1.23585520	2.34005443	2.72323746
H	0.11817391	1.41089575	3.42143635
C	0.29862233	3.42302027	0.71241426
C	-1.01842314	3.91944576	0.65437561
C	1.27716549	3.94638717	-0.15536459
C	-1.33296470	4.89772719	-0.25385921
H	-1.80303923	3.55456217	1.30192958
C	0.93103617	4.92520447	-1.05103623
H	2.30289551	3.60653916	-0.14335332
C	-0.36985059	5.41208309	-1.11397333
F	-2.57536320	5.38655599	-0.33583003
F	-0.68591645	6.36094137	-1.97920363
F	1.83657930	5.44260846	-1.88860994

*

constrF19

Au	-0.15806818	-2.83762875	0.17406212
C	1.16132227	-0.33312371	1.19433590
C	0.06216564	-1.09912315	1.25414109
C	0.17831124	1.50676384	1.88891055
C	1.34382697	0.76665322	2.27838708
H	1.24450951	0.31782789	3.26772623
C	-0.98418799	-0.65124780	2.25016001
H	-0.69111846	-1.05277427	3.22955056
H	-1.96685520	-1.06747099	2.03189907
C	-1.10588179	0.88580170	2.27523264
P	-0.41037420	-4.78616152	-1.03805371
C	-0.95812969	-4.51770444	-2.74360081
C	1.11030705	-5.75856572	-1.18990847
H	1.93724767	-0.37805954	0.44000894
C	-1.62408903	-5.93375776	-0.33769420
H	-2.59974484	-5.44639430	-0.28942429
H	-1.69560649	-6.83127287	-0.95655722
H	-1.31999190	-6.21218600	0.67310797
H	-1.92097252	-4.00328818	-2.73982620
H	-1.05822563	-5.47390768	-3.26256957
H	-0.22928315	-3.89511534	-3.26598918
H	1.87749699	-5.15886641	-1.68337173
H	0.92288192	-6.66240654	-1.77433396
H	1.46636278	-6.03529100	-0.19572087
H	2.29334907	1.28055669	2.17560601
H	-1.91888693	1.21374164	1.63051340
H	-1.32506827	1.23720309	3.29475843
C	0.24832887	2.72775566	1.08359267
C	-0.86500452	3.56397001	0.99968659
C	1.42524100	3.08508639	0.42280644
C	-0.79907130	4.75813003	0.28913712
H	-1.79101566	3.31775071	1.50178077
C	1.48160631	4.24548047	-0.33848876
H	2.30614332	2.45917918	0.46588698
C	0.37070518	5.08114843	-0.38663153
N	0.43441484	6.32830396	-1.17220234
O	0.89478028	6.24691080	-2.28973887
O	0.02060814	7.33351135	-0.63788417
C	-1.97085353	5.59478160	0.24372143
N	-2.93589080	6.21556542	0.22695771
C	2.71345859	4.56303049	-1.01450285
N	3.72173347	4.77674440	-1.51915500

*

constrF20
Au -0.17663057 -3.34000406 -0.03210871
C -1.68500763 -0.99955611 1.04044766
C -1.36719788 -1.65031584 -0.08004032
C -2.35379504 0.82727040 -0.08591258
C -2.78065556 0.12962181 1.10169759
H -3.77122213 -0.30844512 1.00528989
C -1.81964126 -1.14746386 -1.40938257
H -2.32879344 -1.91519970 -1.99531278
H -0.93163118 -0.84857746 -1.97382771
C -2.73833562 0.14288891 -1.31952629
P 1.15221680 -5.22536532 0.01601086
C 2.33005401 -5.25630532 1.39195630
C 0.22362391 -6.77345913 0.16870287
H -1.21054177 -1.17100205 2.00065843
C 2.16954565 -5.44214714 -1.46704327
H 2.82606042 -4.57813433 -1.58676996
H 2.77324023 -6.34883100 -1.38258450
H 1.52302142 -5.51617276 -2.34355570
H 2.98929364 -4.38864566 1.32601771
H 2.92729370 -6.17054030 1.35811730
H 1.78559240 -5.21263996 2.33721674
H -0.36017591 -6.75786867 1.09101721
H 0.90833009 -7.62471580 0.18553613
H -0.45904857 -6.87252337 -0.67744951
H -2.68710538 0.66483733 2.04024722
H -2.61509991 0.72454488 -2.22730545
H -3.77181994 -0.19192250 -1.22989411
C -1.43586053 1.96283730 -0.05927823
C -0.93724388 2.45291249 1.15287732
C -1.03018138 2.57074421 -1.24910451
C -0.04673941 3.50908609 1.18006617
H -1.22553701 2.00202364 2.09105198
C -0.14396758 3.63555746 -1.24020819
H -1.40726268 2.22849953 -2.20100486
C 0.34189896 4.09011201 -0.02294570
C 0.46572951 3.99942514 2.52242071
C 0.27018916 4.24693852 -2.56712286
N 1.29830379 5.22225078 -0.00313042
F 0.33742380 3.05395077 3.46439969
F -0.21127377 5.07872648 2.94716721
F 1.76722842 4.33352790 2.46774979
F -0.64647351 4.00640701 -3.51574988
F 1.43594266 3.74050523 -3.00148452
F 0.41472967 5.58101381 -2.48358417
O 0.89518797 6.26801080 0.45263176
O 2.40383033 5.00537896 -0.44474570
*

constrF21
Au -0.50847408 -2.38979206 0.14893487
C 0.64961296 -0.09105385 1.65484509
C -0.49674572 -0.63874742 1.24803841
C -0.32373664 1.87431355 2.11039894
C 0.72330623 1.08216147 2.70355506
H 0.47300464 0.71056743 3.69422116
C -1.80180900 0.02526629 1.52952888
H -2.51429163 -0.64388651 2.01607693
H -2.23993552 0.31848279 0.57153516
C -1.66643296 1.36140227 2.37570688
P -0.51776926 -4.34141239 -1.08130160
C -1.45754378 -4.22992258 -2.62594501
C 1.13085254 -4.89827666 -1.58483567
H 1.62842037 -0.38529989 1.29147665
C -1.23597181 -5.75635809 -0.20735284
H -2.27024558 -5.53145654 0.05993084
H -1.20897410 -6.64543826 -0.84182039
H -0.66994156 -5.94457640 0.70692293
H -2.49558147 -3.97646449 -2.40234892
H -1.42240535 -5.18295363 -3.15906243
H -1.03205363 -3.44562945 -3.25490136
H 1.60485553 -4.12632784 -2.19410032
H 1.05722332 -5.82314408 -2.16180544
H 1.74311594 -5.07065702 -0.69762945
H 1.71721305 1.51686205 2.68736218
H -2.47428683 2.02915581 2.09347919
H -1.74492762 1.09558423 3.43023447
C -0.05716970 2.95481900 1.16106279
C -1.08603169 3.83062927 0.80320375
C 1.22485293 3.11576016 0.61727755
C -0.82028236 4.85604600 -0.08024264
H -2.08448746 3.75795829 1.21007045
C 1.44609137 4.12237980 -0.29123073
H 2.04773230 2.45811409 0.86197028
C 0.43450450 5.00710265 -0.64484538
F -1.79584370 5.69849729 -0.42450624
F 2.65866723 4.27972667 -0.82587203
N 0.69253262 6.08576847 -1.60585312
O 1.11378556 5.76036937 -2.69487568
O 0.46345636 7.21759022 -1.23789719
*

constrF24
Au -0.26244120 -2.90164344 0.05980136
C 0.21633631 -0.64002080 1.94715342
C -0.65848542 -1.14714443 1.07983894
C -0.87030427 1.40086941 1.94025323
C -0.05475243 0.61716491 2.84979557
H -0.60354126 0.31289741 3.74048945
C -1.93766719 -0.43433875 0.77684876
H -2.80449969 -1.09328508 0.85656400
H -1.90236889 -0.07032920 -0.25440048
C -2.19028781 0.82112908 1.70812945
P 0.18562522 -4.85215408 -1.08756192
C 1.67882298 -4.77534619 -2.11100918
C 0.43589476 -6.29747853 -0.02351796
H 1.21230563 -1.04360075 2.09699056
C -1.12833896 -5.34930133 -2.23192317
H -1.28591925 -4.56013718 -2.96967706
H -0.85531120 -6.27567048 -2.74292995
H -2.05593592 -5.50038473 -1.67640859
H 1.57557894 -3.97469632 -2.84593494
H 1.83289573 -5.72579102 -2.62738912
H 2.54220799 -4.56135705 -1.47806162
H 1.27550706 -6.11290371 0.64935343
H 0.64335253 -7.18226456 -0.63004819
H -0.46145628 -6.46848033 0.57429465
H 0.89906129 1.05841326 3.12069241
H -2.90365380 1.48971747 1.23498205
H -2.59146036 0.45223092 2.65199445
C -0.34772876 2.49146718 1.15010567
C -1.05744236 2.95328641 0.03294382
C 0.88332285 3.09037642 1.47088607
C -0.53703407 3.96869877 -0.75033678
H -2.00865358 2.51405593 -0.23673709
C 1.37926763 4.10935756 0.68564977
H 1.43917715 2.77180638 2.34086703
C 0.68016862 4.55150870 -0.43412989
C -1.31051079 4.41832279 -1.96604860
C 2.70954140 4.74532009 1.00681568
H 1.08148529 5.35066445 -1.04585306
F -0.85396323 5.58067674 -2.45887120
F -2.61787527 4.58662030 -1.68752800
F -1.23978634 3.50933966 -2.96103480
F 3.10956617 4.48284596 2.26230263
F 2.66704780 6.08406042 0.87071136
F 3.68021297 4.30069475 0.18136086
*

constrF4
Au 0.10202840 -2.81443208 0.48172237
C 1.13808767 -0.33599534 1.78751255
C 0.03740730 -0.93030212 1.33087459
C 0.02045752 1.73010467 1.92829826
C 1.16414213 1.04814259 2.51838098
H 1.02658853 0.88619433 3.58875327
C -1.28726371 -0.23484172 1.39369737
H -2.06225843 -0.87435292 1.82088779
H -1.60696235 0.02636876 0.38060610
C -1.25409974 1.09588472 2.24867309
P 0.17188366 -4.91898720 -0.46050472
C 1.27532629 -5.05415389 -1.89185300
C 0.73486814 -6.21651615 0.67250538
H 2.13106006 -0.75994459 1.67752963
C -1.43202394 -5.50760389 -1.06479266
H -1.80836732 -4.82086004 -1.82544966
H -1.33137760 -6.50695113 -1.49488106
H -2.14323995 -5.53806151 -0.23708343
H 0.95145696 -4.35795548 -2.66791465
H 1.25943031 -6.07263078 -2.28714636
H 2.29295401 -4.79646030 -1.59204391
H 1.74078239 -5.98041204 1.02497974
H 0.74494488 -7.18421490 0.16526129
H 0.06428393 -6.26371100 1.53272249
H 2.12257222 1.52204098 2.32867793
H -2.12953558 1.69950729 2.02630899
H -1.25356033 0.80152021 3.29849188
C 0.14870634 2.77155627 0.94254167
C -0.93306325 3.08131640 0.09543492
C 1.35261208 3.48181514 0.80221576
C -0.80042195 4.04711834 -0.87977342
H -1.86986706 2.54798901 0.18255814
C 1.47685759 4.46878177 -0.15915632
H 2.18767436 3.28511367 1.46006796
C 0.40457936 4.73554249 -0.99952883
H -1.62809949 4.26901706 -1.54256227
H 2.40265659 5.02090832 -0.25370537
C 0.50971019 5.81065418 -2.05761963
F 0.08106242 5.36640506 -3.25496485
F -0.24983229 6.87966617 -1.74158000
F 1.76619665 6.25256666 -2.22348904
*

constrF9
Au -0.15269974 -1.88546366 0.13323543
C 1.04690367 0.51057820 1.45903071
C -0.10049846 -0.04319243 1.07080469
C 0.01928166 2.57067521 1.77128894
C 1.15696970 1.83528903 2.29491987
H 1.04723259 1.60134016 3.35387457
C -1.40137507 0.66615229 1.27442026
H -2.14563721 0.02672013 1.75324752
H -1.80388411 0.95427072 0.29886203
C -1.26734600 1.98569710 2.13751182
P -0.23490318 -3.94552774 -0.90183624
C 0.02886847 -3.88445313 -2.69341771
C 0.99843724 -5.12774728 -0.29806396
H 2.02067121 0.09578838 1.21997803
C -1.82071101 -4.79959104 -0.70287056
H -2.61993212 -4.18549273 -1.12231777
H -1.79754366 -5.76506165 -1.21385542
H -2.01882747 -4.95511161 0.35940847
H -0.73445880 -3.25255437 -3.15155244
H -0.02717825 -4.88922202 -3.11891230
H 1.01081209 -3.45534610 -2.90201704
H 1.99954643 -4.72355885 -0.46009192
H 0.90068223 -6.07956512 -0.82554937
H 0.85428639 -5.28854361 0.77206906
H 2.12537259 2.28557289 2.09940831
H -2.12279655 2.63121260 1.95930128
H -1.23448844 1.68355979 3.18461281
C 0.14708949 3.63065923 0.79848340
C -0.95017185 3.98164102 -0.00788113
C 1.36401098 4.31582833 0.64288579
C -0.82938514 4.97109357 -0.96220855
H -1.89463281 3.46457673 0.09414574
C 1.48436932 5.32406133 -0.29303891
H 2.21189203 4.08142378 1.27138785
C 0.38801031 5.64006064 -1.09486068
H -1.66635294 5.23337093 -1.59684009
H 2.41519089 5.86507905 -0.40706298
C 0.51306513 6.68765383 -2.07873611
N 0.61144139 7.52204444 -2.86244539
*

TS-DoeringLaFlamme(FigS6)
C 0.9693882 -1.4058616 2.0075758
C 2.4129403 -0.3962333 2.0838058
C 1.3557126 -0.3222646 1.1386433
H 0.9031352 -2.3981063 1.5207759
H 2.3001776 -0.1727507 3.1555287
O 0.2244492 1.6192240 -2.4807586
C -1.1450946 2.1153703 -2.3773486
C 1.0624449 2.5914063 -3.1787135
C -1.0557451 3.5942278 -2.7447576
H -1.7705558 1.5457132 -3.0918611
H -1.4904863 1.9346470 -1.3460692
C 0.0811902 3.5965385 -3.7801586
H 1.7350068 3.0641138 -2.4374799
H 1.6670391 2.0411992 -3.9177724
H -0.7678827 4.1856409 -1.8549886
H -2.0105846 3.9852887 -3.1397912
H 0.5421473 4.5905758 -3.9217139
H -0.2885525 3.2371835 -4.7598329
O -0.4500837 -0.9374779 -1.3684159
C -0.7553976 -1.4294013 -2.7077812
C -1.4986884 -1.3459219 -0.4486237
C -2.2164077 -1.9192235 -2.6473599
H -0.5966741 -0.5852001 -3.3989724
H -0.0405301 -2.2354409 -2.9543208
C -2.7481100 -1.3354127 -1.3214812
H -1.2702061 -2.3648947 -0.0745240
H -1.4925154 -0.6387735 0.3977406
H -2.2488928 -3.0239078 -2.6157043
H -2.7981043 -1.5837394 -3.5242144
H -3.5707236 -1.9335607 -0.8900513
H -3.1010452 -0.2959253 -1.4599816
Mg 1.0435567 0.4282146 -0.9199518
Cl 2.9591197 -0.1807128 -2.1377926
Cl 0.4420125 2.1453160 0.6858011
C 3.8511613 -0.5272427 1.6302877
H 3.9179260 -0.7209268 0.5446021
H 4.3880449 0.4179421 1.8572684
H 4.3623731 -1.3394093 2.1861344
N 0.3782803 -1.3790281 3.3023705
C -0.7499748 -0.5906459 3.5277956
O -1.3144265 0.0179148 2.6228247
C -1.2562209 -0.5416994 4.9625514
H -2.0020447 0.2685178 5.0246301
H -1.7511091 -1.4932641 5.2418472
H -0.4422936 -0.3534024 5.6878400
C 0.9446590 -2.2951479 4.2955841
H 0.1843077 -2.5984684 5.0325417
H 1.3021880 -3.2018117 3.7721093
H 1.8050886 -1.8331783 4.8221648
*

TS-HAT(FigS7)
C -0.96708232 -0.07668326 -0.03171210
H -0.27388617 1.02017562 -0.02381447
H -1.51495231 -0.04300864 -0.98566922

H	-1.63927692	0.02241139	0.83411876
C	-0.06487713	-1.18912535	0.06987816
N	0.71490919	-2.06042155	0.15427038
C	0.46830470	2.24224970	-0.01730079
H	-0.30144832	3.01814400	-0.12305827
H	0.96996076	2.21191598	0.95877732
H	1.13241445	2.12187387	-0.88319473

*
